# Photochemical deracemization of α-chiral carboxamides mediated by inter- and intramolecular hydrogen bonding

**DOI:** 10.1039/d6sc05947c

**Published:** 2026-09-09

**Authors:** Maximilian Iglhaut, Biki Ghosh, Thorsten Bach

**Affiliations:** a Technische Universität München, School of Natural Sciences, Department of Chemistry and Catalysis Research Center Lichtenbergstrasse 4 85747 Garching Germany thorsten.bach@ch.tum.de https://www.ch.nat.tum.de/en/oc1/home/

## Abstract

Photochemical deracemization allows for the direct generation of enantiomerically pure or enriched compounds from their racemates employing a single chiral catalyst. In the present study, carboxylic acid amides (carboxamides) with a substituent in the α-position were investigated in a photochemical deracemization mediated by a chiral aromatic ketone. The catalyst displays a hydrogen bonding site which allows for a differentiation between the two enantiomers and operates by a reversible hydrogen atom transfer. It was found that α-alkoxy or α-amino substituents enable an efficient deracemization (up to 99.5 : 0.5 e.r.) due to a second intramolecular hydrogen bonding interaction locking a distinct substrate conformation.

## Abstract 2

Acyclic α-chiral carboxamides undergo a photocatalytic deracemization which is assisted by an intramolecular hydrogen bond that locks the substrate in a defined conformation.

## Graphical abstract

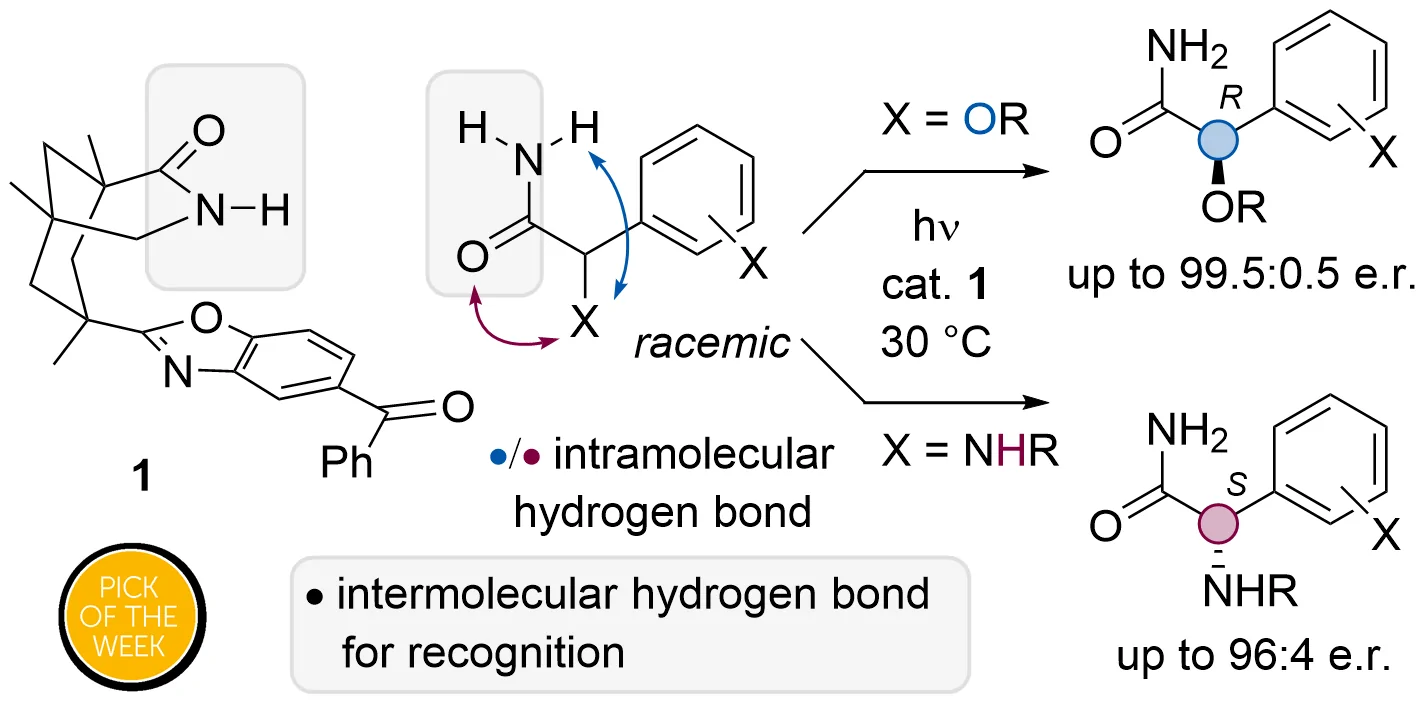

## Introduction

**Scheme 1:**
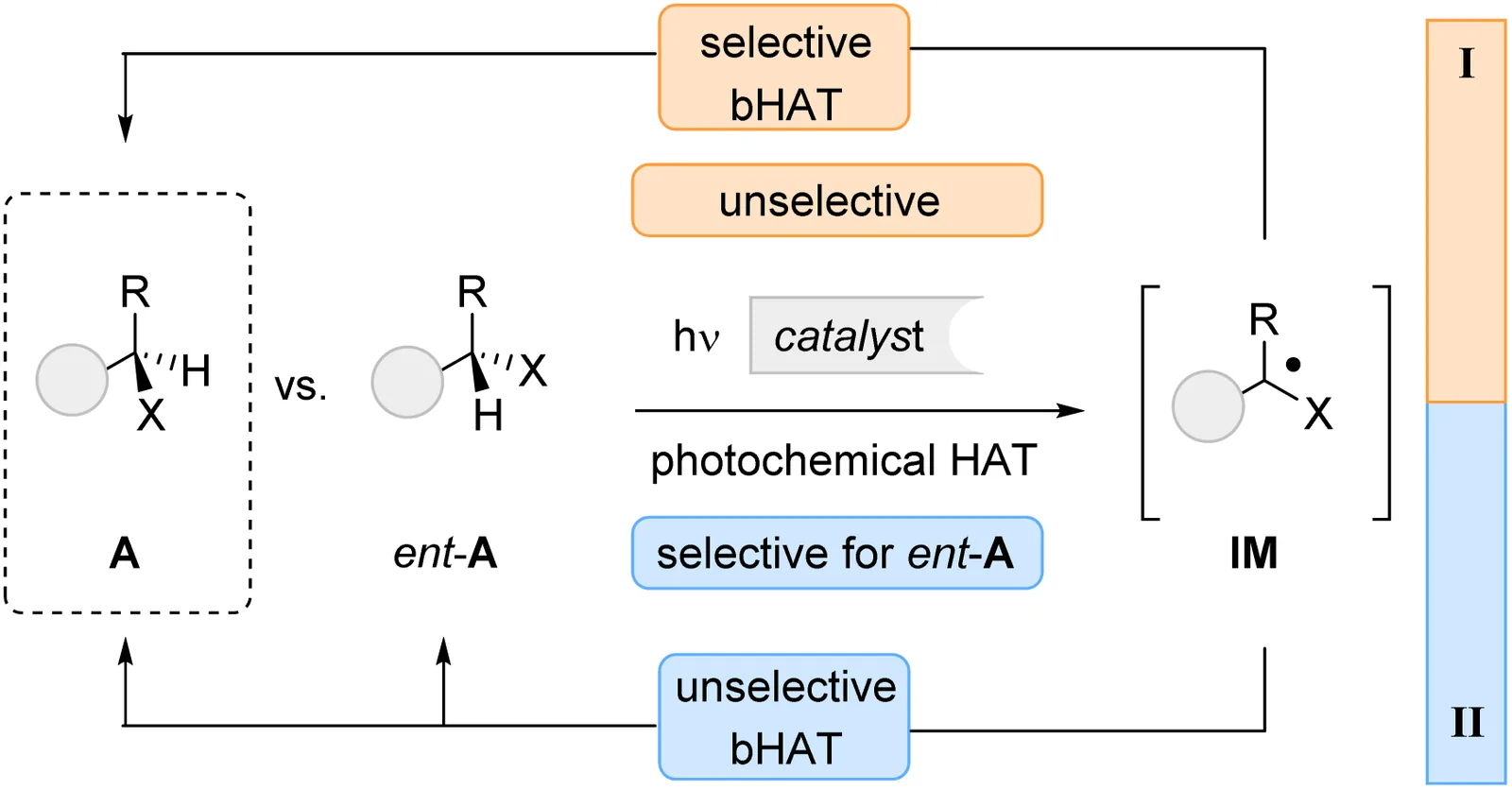
Modes of action of a photochemical deracemization at a stereogenic carbon center towards enantiomer A by reversible hydrogen atom transfer (HAT). Pathway I comprises an unselective HAT and a selective back HAT (bHAT) to the radical intermediate IM typically mediated by a chiral HAT donor; pathway II relies on a selective HAT mediated by a chiral photocatalyst.

An sp^3^-hybridized carbon atom with four different substituents is the most frequently encountered structural motif that confers chirality to a given molecule. If the atom is the only stereogenic element within a molecule, only two stereoisomers are possible which are non-superimposable mirror images to each other and which are commonly referred to as enantiomers. The fact that enantiomers have different physiological properties^1^ has triggered intensive efforts to make chiral compounds available as single enantiomers but not as racemates.^2^ Recent work has shown that it is possible to convert a racemate, *i.e.* a 1 : 1 mixture of enantiomers, into a single enantiomer of the same compound by photochemical methods.^3,4^ In a situation in which a compound with a stereogenic carbon atom A and its enantiomer *ent*-A are to be deracemized (Scheme 1), a viable pathway for deracemization involves the reversible removal of a hydrogen atom at the stereogenic center. If the hydrogen atom transfer (HAT) to the catalyst occurs photochemically and the back HAT (bHAT) thermally, the principle of microscopic reversibility^5^ is not violated because the latter reaction is not the reverse of the former. In both steps, a chiral discrimination is possible. In scenario I, an achiral photocatalyst processes both enantiomers unselectively to form an achiral radical intermediate IM. The removal of the hydrogen atom can occur by a direct HAT or by an electron transfer-deprotonation pathway.^4*b*,*c*,*f*,6^ Selectivity is achieved in the thermal bHAT step, which is promoted by a chiral hydrogen atom donor,^7^ favouring one enantiomer, *e.g.*A. In the second scenario II, the photocatalyst is chiral and differentiates between the substrate enantiomers. Ideally, it processes only one of them, *e.g. ent*-A, forming the achiral radical intermediate IM which undergoes unselective bHAT to reform A and *ent*-A. Since only *ent*-A is processed, enantiomer A will prevail in the photostationary state.^4*d*,8^ Both selectivity levels can be combined by using a chiral photocatalyst and a chiral HAT donor, thus enhancing the enantioselectivity of two moderately selective events.^4*b*^

**Scheme 2:**
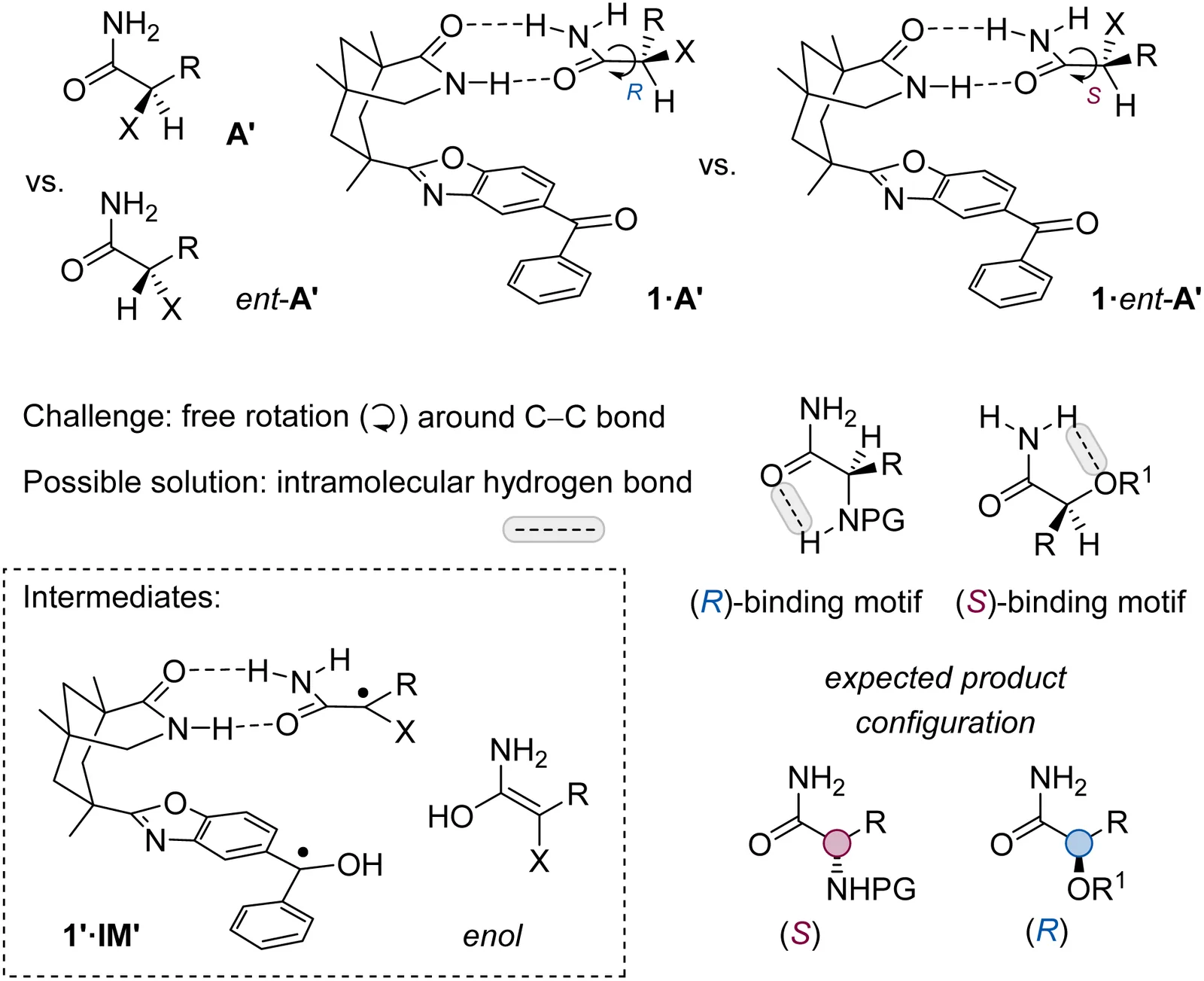
Deracemization of α-branched carboxamides *rac*-A′ by chiral photocatalyst 1: if the rotation around the C–C bond is prevented by an intramolecular hydrogen bond, a preference toward a single substrate enantiomer can be expected by reversible HAT, as shown for α-amino- (PG = protecting group) and α-alkoxy-substituted substrates. The other enantiomer is not processed and will prevail in the photostationary state.

Work in our group has revolved around the development of chiral benzophenone-based photocatalysts which can act both in the HAT and the bHAT event (scenario II).^9^ It has been found that the bHAT step is unselective if it does not proceed immediately to the carbon atom but to an adjacent C=O or C=N bond. The so formed enol and enamine derivatives subsequently tautomerize to the desired products. The concept offers the possibility to use a single chiral catalyst for the process without the requirement for an additional hydrogen atom donor. Although in all cases a chiral 1,5,7-trimethyl-3-azabicyclo[3.3.1]nonan-2-one backbone has served as the recognition element for one of the two substrate enantiomers, an optimisation of the steric and electronic properties has been required to guarantee optimal performance of the catalyst. So far benzophenone catalyst 1 ^10^ and its enantiomer *ent*-1 have been most frequently used in photochemical deracemization reactions (Scheme 2). For the HAT step to be enantioselective, it is necessary that the benzophenone can approach the C–H bond at the stereogenic carbon atom of the substrate only for one but not for the other enantiomer. This task turns out to be challenging, if the substrate enantiomers are acyclic, *e.g.* for amides A′ and *ent*-A′. A distinction between the two enantiomers is impossible unless the free rotation around the indicated C–C bond is prevented and the conformation is locked. A HAT process is facile upon excitation of benzophenone within both diastereomeric complexes 1·A′ and 1·*ent*-A′.

Since coordination of the primary amide to the lactam binding motif of catalyst 1 leaves a lone pair at the oxygen atom and a hydrogen atom at the N–H bond available for additional hydrogen bonds, we hypothesized that they could be exploited to lock possible substrates for a selective HAT. Specifically, a protected amino group (NHPG) in the α-position of the carboxamide should be available for a hydrogen bond with the oxygen atom at the amide C=O bond.^11^ Alternatively, a Lewis basic oxygen atom in the α-position might form a hydrogen bond with the free N–H atom of the carboxamide.^12^ In the former situation, the (*R*)-enantiomer of the substrate would display an abstractable hydrogen atom for a HAT to benzophenone, while in the latter case, a HAT from the (*S*)-enantiomer would be favoured. Upon HAT, a complex of the radical intermediate IM′ with the protonated ketyl radical 1′ would form in which bHAT would lead to an achiral enol species. Tautomerization reforms compounds A′ and *ent*-A′, and it was expected that the non-processed enantiomer would eventually prevail, *i.e.* the (*S*)-enantiomer for the α-amino, and the (*R*)-enantiomer for the α-alkoxy case. In this article we describe the realization of a photochemical deracemization based on the presented concept. The desired compounds were obtained upon proper optimisation of the reaction conditions in enantiomerically pure or enriched forms. The process was employed for stereochemical editing in compounds with an additional stereogenic center, and other substrates with various substituents X at the stereogenic center were probed. A by-product was identified which originates from dimerization of the catalyst and which is likely responsible for a decrease in catalyst efficiency upon prolonged irradiation.

## Results and discussion

The study commenced with the commercially available mandelic acid derivative *rac*-2a, which displays a methoxy group in the α-position. Irradiation was performed with fluorescent lamps (see the SI for details) with an emission maximum at *λ* = 366 nm and *λ* = 350 nm (Table 1). Absorption of benzophenone catalyst 1 in this wavelength region is due to a forbidden n–π* transition (*λ*_max_ = 355 nm, *ε* = 132 M^−1^ cm^−1^) which populates the first excited singlet state S_1_. Intersystem crossing (ISC) is rapid and occurs efficiently on a ps time scale populating the reactive triplet state (T_1_) which also has n–π* character.^8*b*,13^

**Table 1:** Reaction optimisation for the photochemical deracemization of racemic *O*-methyl mandelamide (*rac*-2a) catalysed by chiral photocatalyst 1

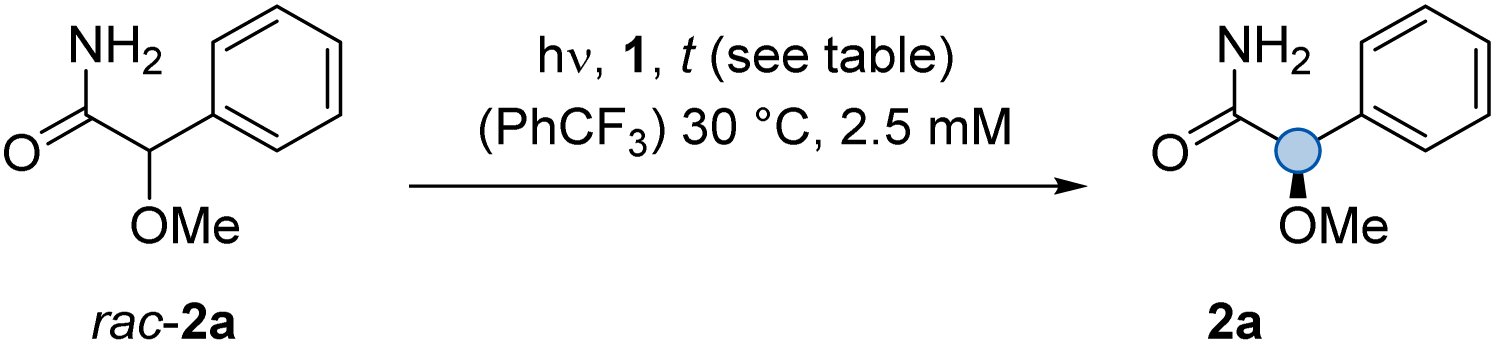					
entry^a^	1 [mol%]	*λ* [nm]	*t* [h]	Yield	e.r.^b^
1	10	366	13	97%	84 : 16
2	10	350	13	Quant.	81 : 19
3	15	366	13	91%	89.5 : 10.5
4	10	366	18	95%	90.5 : 9.5
5	10	366	24	91%	90.5 : 9.5
6^c^	10	366	18	79%	85 : 15
7	5	366	18	92%	76.5 : 23.5
8	7.5	366	18	Quant.	79.5 20.5
9	12.5	366	18	87%	90.5 : 9.5
10	15	366	24	87%	92.5 : 7.5
11	7.5 + 7.5	366	18 + 18	95%	95 : 5

a Reactions were performed under deaerated conditions at a substrate concentration of *c* = 2.5 mM by irradiation with fluorescent lamps.

b The enantiomeric ratio (e.r.) was determined by chiral-phase HPLC analysis.

c Thiophenol (20 mol%) was used as the additive.

**Fig. 1:**
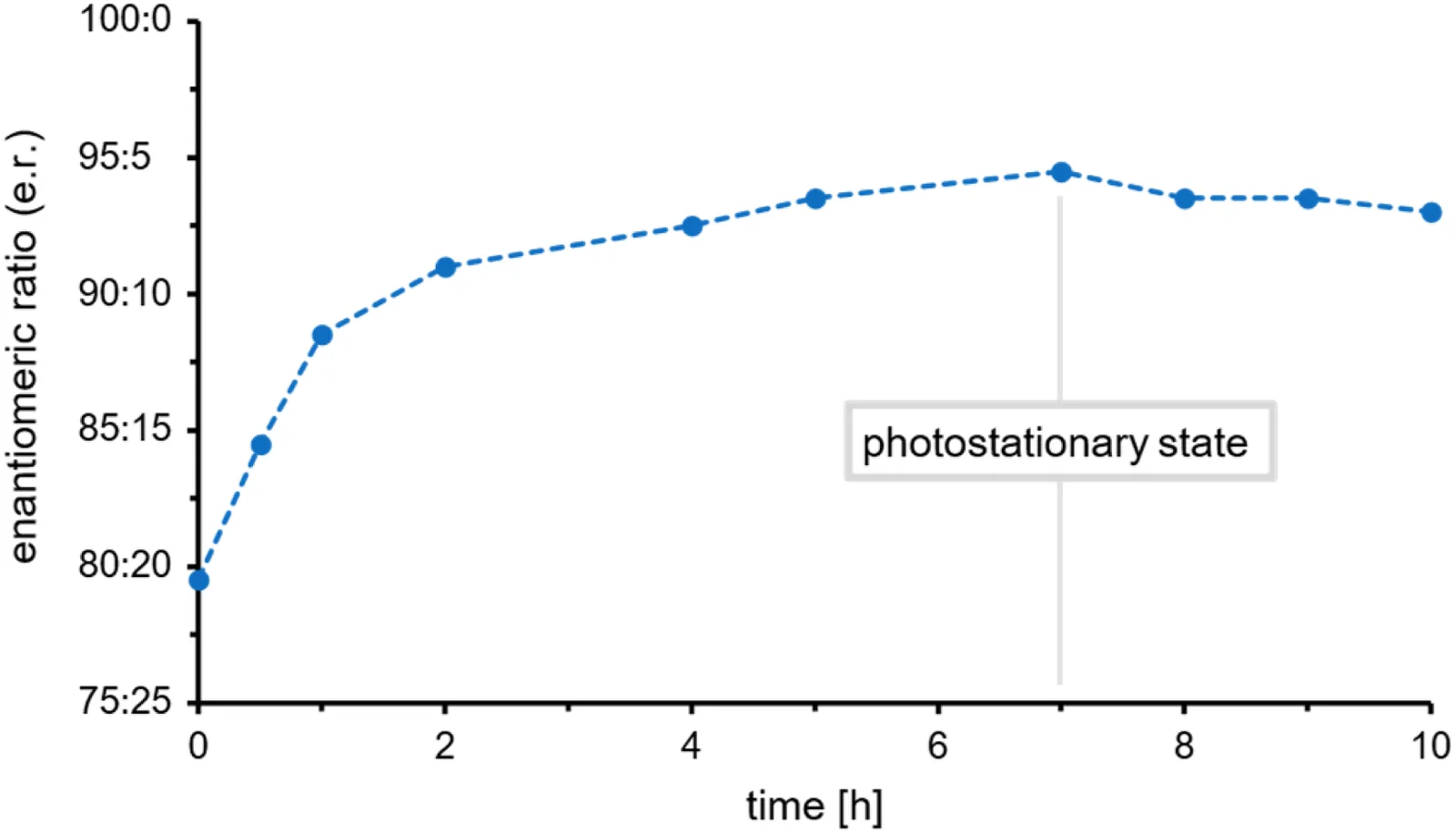
Time-dependent progression of the enantiomeric ratio (e.r.) for an enantiomeric enriched sample of product 2a (79.5 : 20.5 e.r., *cf.*Table 1, entry 8) when irradiated in the presence of additional 7.5 mol% catalyst 1: The photostationary state is reached after approx. seven hours.

If the subsequent deracemization follows the pathway described in Scheme 2, it was expected that the (*R*)-enantiomer 2a was the product. Under conditions which had previously been used in the deracemization of lactams,^4*d*,8*a*,9^ the yield was 97% and the ratio of enantiomers (e.r.) was 84 : 16 (entry 1). At a shorter wavelength (entry 2) the enantioselectivity was even less pronounced. A higher catalyst loading (entry 3) and longer reaction times (entries 4, 5) improved the enantioselectivity. Addition of a potential hydrogen atom shuttle, which had turned out to be beneficial for the deracemization of chromanes,^8*e*^ led to a decrease in selectivity (entry 6). A more detailed variation of the catalyst loading at a constant irradiation time of *t* = 18 h (entries 7–9) revealed the substrate to be essentially stable as long as the loading was 7.5 mol% or below. Higher loadings gave a better enantioselectivity at the expense of yield (entries 9, 10). Since catalyst decomposition appeared to be an issue (*vide infra*), the catalyst was added in two portions which improved the enantioselectivity to 95 : 5 e.r (entry 11). Still, the irradiation time after the addition of the second catalyst portion seemed not perfectly adjusted, which is why we studied the time course of the e.r. in more detail (Fig. 1).). Irradiation of amide *rac*-2a under the conditions of entry 8 resulted in a quantitative yield and 79.5 : 20.5 e.r. We used this result as a starting point and – instead of isolating the deracemization product after 18 h – we added another 7.5 mol% of the catalyst to the reaction solution, and irradiation was continued. The photostationary state was reached after an irradiation time *t* = 7 h. The e.r. was not optimal under the conditions because the repeated removal of samples introduced oxygen to the reaction mixture. When the experiment was repeated without taking aliquots, *i.e.* by running the reaction for a total of 25 hours (18 + 7) with consecutive addition of two batches of the photocatalyst (7.5 + 7.5 mol%), the desired product 2a was isolated in 97% yield and 96.5 : 3.5 e.r.

**Scheme 3:**
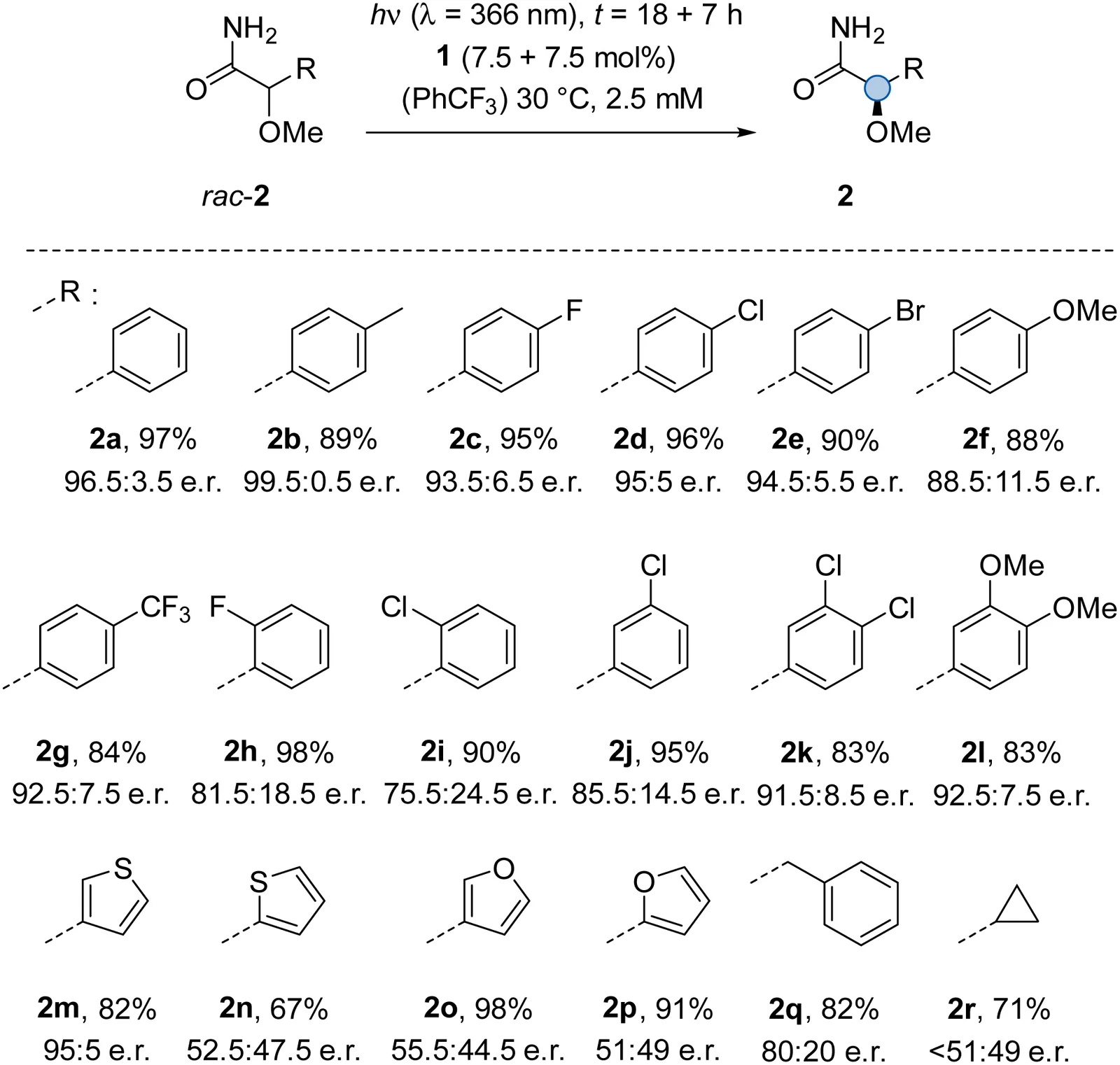
Photochemical deracemization reactions of various α-methoxy-substituted carboxamides *rac*-2 delivering enantioenriched amides 2.

The absolute configuration of product 2a was confirmed by HPLC comparison with an authentic sample of the (*R*)-configuration (see the SI for details). The assignment of all other products with an α-alkoxy substituent was based on the analogy to 2a. The optimised reaction conditions were applied to a series of α-methoxy carboxamides *rac*-2 (Scheme 3). The compounds were prepared by methylation and amide bond formation from the respective α-hydroxy carboxylic acids^14^ if the latter were commercially available. If not, the α-methoxy carboxylic acid was obtained from the aldehyde (RCHO) by reaction with bromoform in basic methanol solution^15^ (see the SI for details). The photochemical deracemization of *para*-substituted *O*-methyl mandelamides proceeded smoothly and produced the desired products 2a–2g in high yields and enantioselectivity. On a 1 mmol scale, product 2a was obtained in 94% yield and 95.5 : 4.5 e.r. (*t* = 20 + 10 h). If the substituent was located in the *ortho* (2h, 2i) or *meta* position (2j), the enantioselectivity suffered, potentially because the substituent compromised the delicate binding situation.

Disubstitution at the phenyl core was compatible with deracemization, and products 2k and 2l were obtained with high enantioselectivity. The same applied to the 3-thienyl-substituted product 2m which was obtained in 95 : 5 e.r. Other heterocyclic substrates *rac*-2n to *rac*-2p failed to provide a meaningful differentiation between the two enantiomers, which may be related for *rac*-2n to decomposition of the catalyst and substrate under the reaction conditions and for the furyl derivatives *rac*-2o and *rac*-2p to competitive binding. Since detection of compounds without a chromophore was difficult by HPLC analysis, only two aliphatic compounds *rac*-2q and *rac*-2r were investigated. The amide with a benzyl group at the stereogenic center delivered the desired enantiomer 2q with moderate enantioselectivity, while cyclopropane *rac*-2r remained essentially racemic.

There are several fundamentally different reasons why a photochemical deracemization with catalyst 1 may fail. Most obviously, the catalyst may not be competent to perform the desired HAT in its excited state, either because the C–H bond dissociation energy (BDE) of the substrate is too high or because the distance between the oxygen atom of the benzophenone and the target hydrogen atom is too large. Our current hypothesis for a successful HAT,^9^ supported by literature precedence, assumes that the BDE^16^ should be below 400 kJ mol^−1^ and that the O–H distance^17^ should be below 300 pm. A second potential reason for a low enantioselectivity is an insufficient differentiation between the two substrate enantiomers, *e.g.* because the suggested hydrogen bond (Scheme 2) is not stable or compromised by another heteroatom in the substrate. Other labile C–H bonds in the immediate vicinity of the α-carbon atom might also cause a lack of selectivity (*e.g.* the benzylic C–H bonds in substrate *rac*-2q). Thirdly, the catalyst is typically not recovered and delivers various side products (*vide infra*). Some of the side products might still be productive, and others, particularly those stemming from oxidation or reduction, manifest an irreversible decomposition. This situation is frequently indicated by a low product yield, as seen in the case of substrates *rac*-2n and *rac*-2r.

**Scheme 4:**
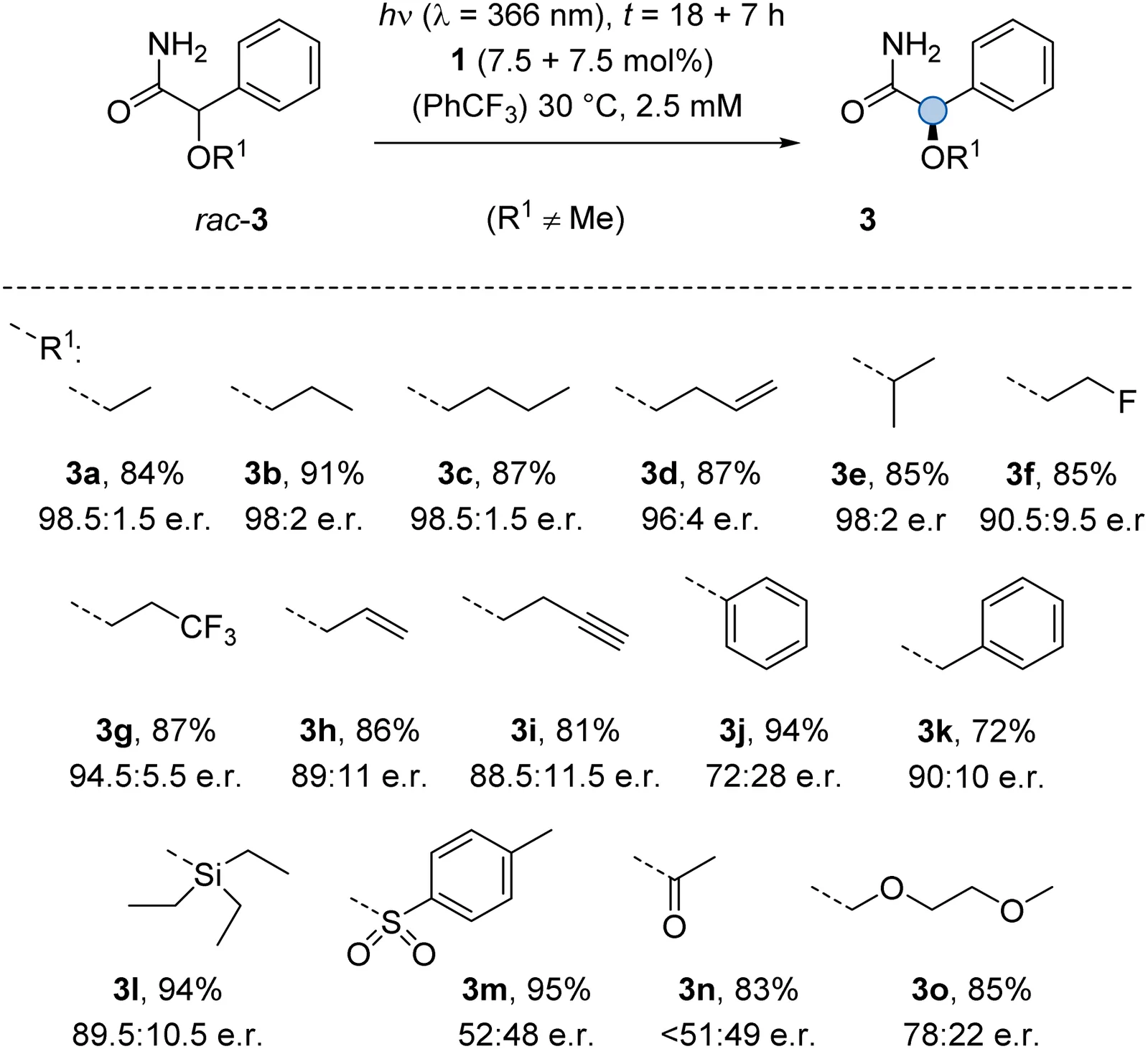
Photochemical deracemization reactions of various α-alkoxy-substituted mandelamides *rac*-3 delivering enantioenriched amides 3.

In a second series of experiments, we investigated the role of the substituent *R*^1^ at the oxygen atom in the α-position of mandelamide (Scheme 4). All alkoxy substituents were fully compatible and gave good to excellent enantioselectivities (products 3a–3g). Fluorine (3f and 3g) and olefin (3d) substitution was tolerated, but allylic ether *rac*-3h reacted less selectively. A lower selectivity was also recorded for butynyl (*rac*-3i) and phenyl substitution (*rac*-3j) at the oxygen atom. The benzyl ether *rac*-3k gave 90 : 10 e.r. and silyl ether *rac*-3l 89.5 : 10.5 e.r. with the triethylsilyl (TES) substitution resulting in a significantly better yield (94%).

Other possible leaving or protecting groups at the α-oxygen atom such as *para*-toluenesulfonyl (Ts, *rac*-3m), acetyl (Ac, *rac*-3n) and methoxyethoxymethyl (Mem, *rac*-3o) led to low enantioselectivities. It is likely that the electron-withdrawing Ts and Ac groups render the oxygen atom in the α-position too electron deficient, and intramolecular hydrogen bonding becomes unfeasible. Attempts to use α-hydroxy-substituted mandelamide under the optimised reactions conditions failed due to its insolubility in trifluorotoluene.

**Scheme 5:**
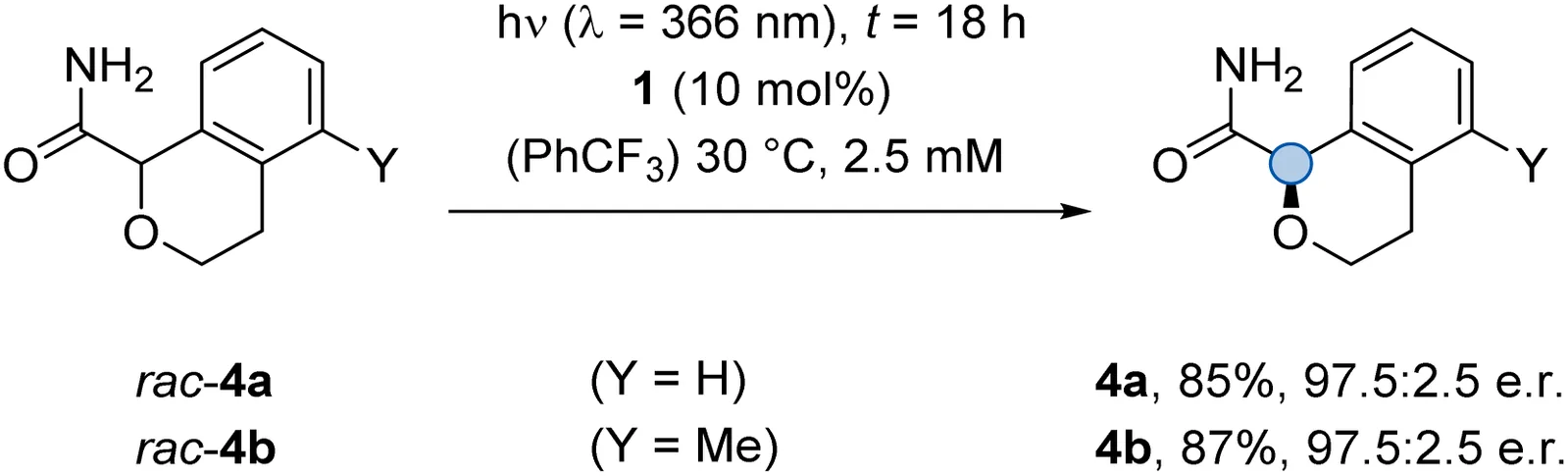
Photochemical deracemization of isochromane-1-carboxamides *rac*-4 delivering enantioenriched products 4.

Isochromane-1-carboxamides^18^*rac*-4 represent a special class of mandelic acid derivatives in which the alkoxy-substituent is linked to the phenyl group. Since the stereogenic center with the labile C–H bond is embedded in a ring system, there is less conformational freedom, and the reversible HAT was expected to proceed smoothly. In fact, products 4a and 4b were obtained with excellent enantioselectivity by applying only 10 mol% of the catalyst and terminating the reaction after a reaction time of 18 hours (Scheme 5).

**Scheme 6:**
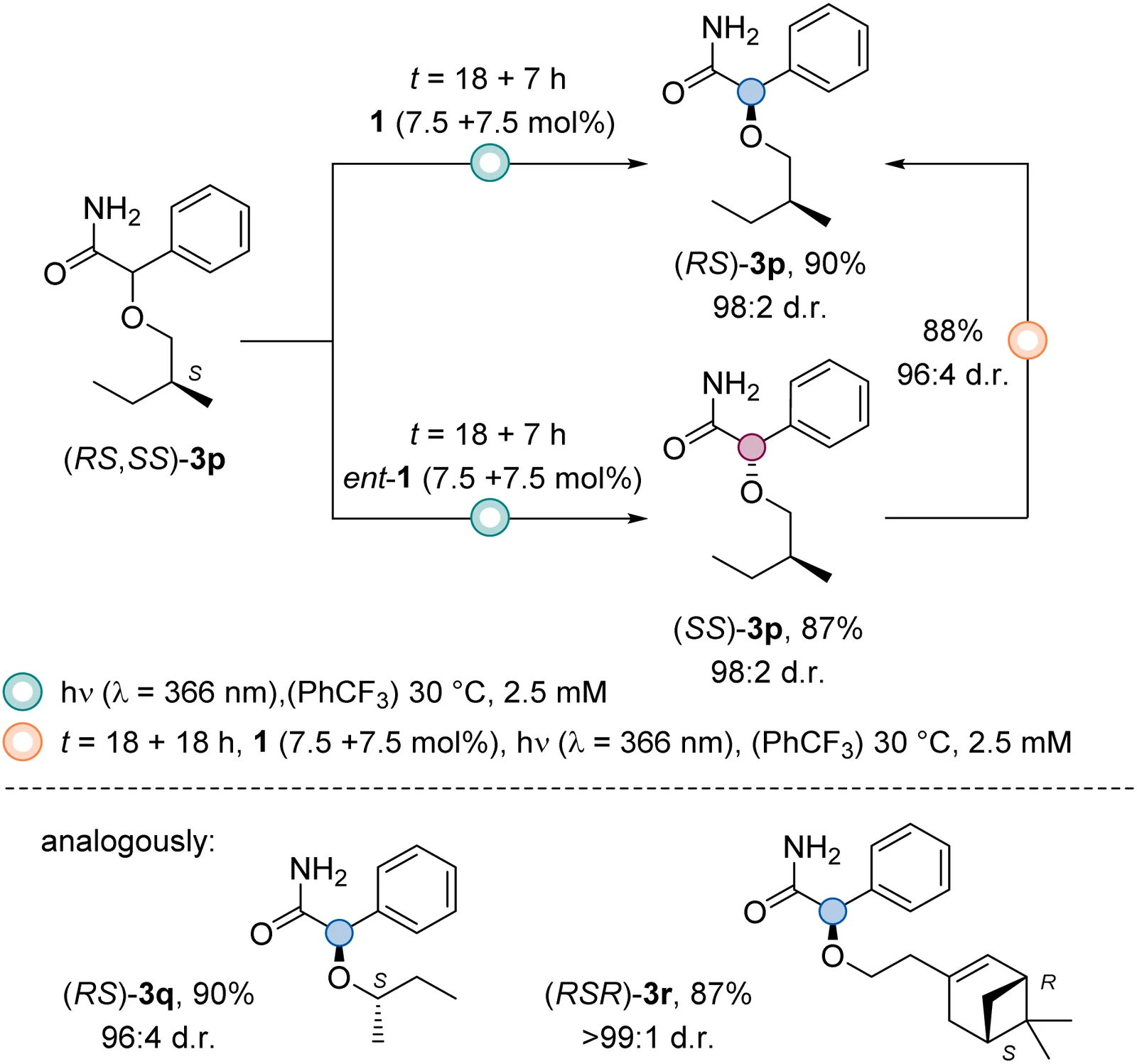
Stereochemical editing of the α-stereogenic center in mandelamides bearing a substituent with a defined absolute configuration (d.r. = diastereomeric ratio).

The site selectivity of the photochemical deracemization is only critical if a C–H bond with a low BDE is in the immediate vicinity of the targeted stereogenic center. In any other situation, the process shows a high fidelity towards the stereogenic center displayed to the catalyst by hydrogen bonding. Site-selective stereochemical editing of substrates with multiple stereogenic centers is consequently facile and was demonstrated for compounds 3p to 3r (Scheme 6).

For the diastereomeric mixture of compound 3p that carries a defined stereogenic center within the alkoxy substituent, it was shown that the α-stereogenic center of the mandelamide can be established at will by employing either catalyst 1 or its enantiomer *ent*-1. In the former case, diastereoisomer (*RS*)-3p was the major product, and in the latter case, its epimer (*SS*)-3p was formed. In an analogous fashion a diastereomeric mixture of mandelamide (*RS*,*SS*)-3q derived from (2*S*)-butanol was converted into a single diastereoisomer (*RS*)-3q employing catalyst 1. The substrate for the preparation of product (*RSR*)-3q was obtained from (–)-nopol, and stereochemical editing was performed at the α-stereogenic center of the mandelamide with catalyst 1.

**Scheme 7:**
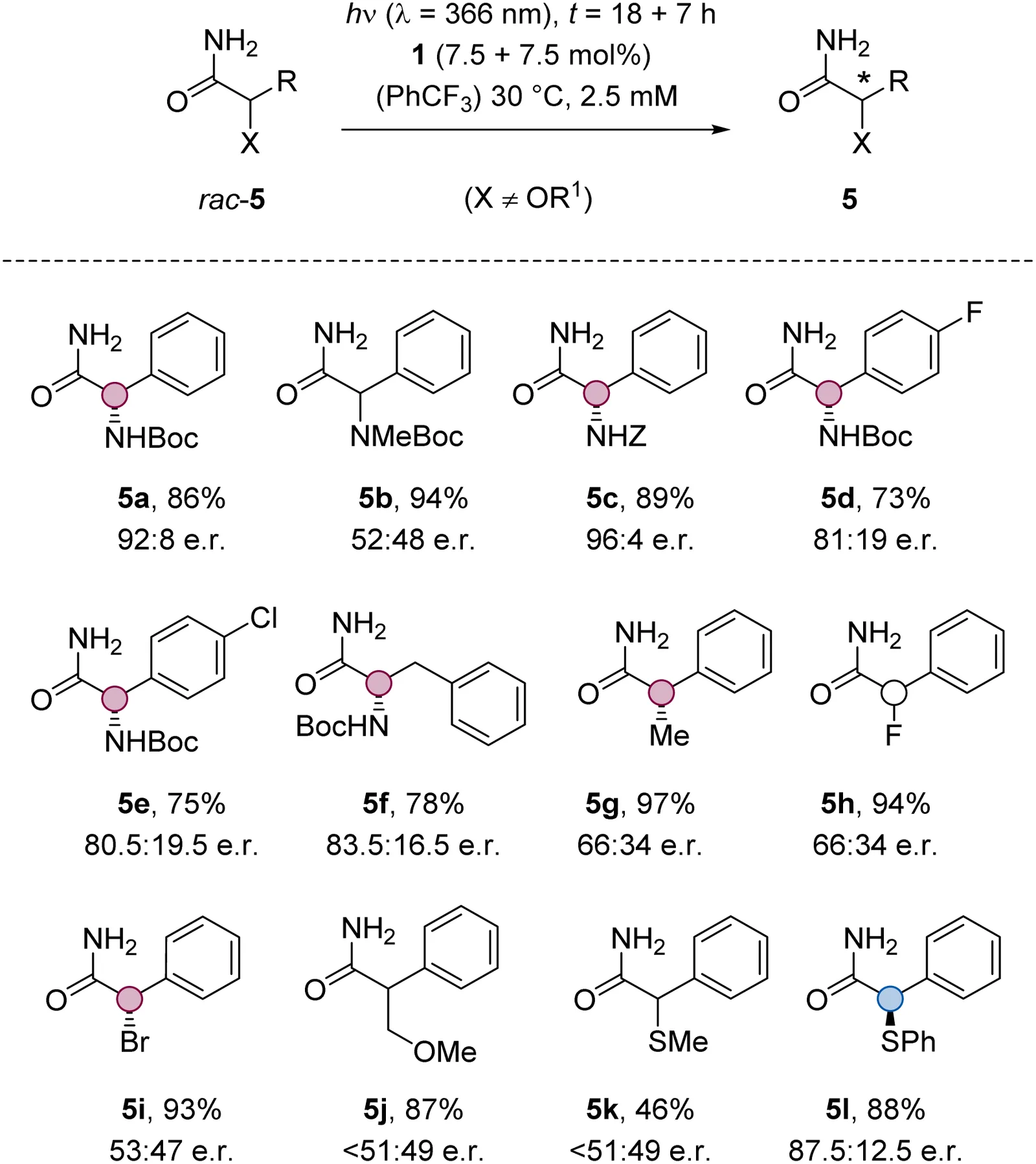
Photochemical deracemization attempts performed with various α-branched primary carboxyamides *rac*-5. The non-coloured circle (○) at the stereogenic center indicates that the absolute configuration of the major product was not determined.

In a final set of experiments the α-substituent of various carboxamides was varied, and it was investigated whether a notable enantioselectivity could be achieved if the substituent X was not an alkoxy group (OR^1^) (Scheme 7). The most pronounced differentiation was observed for phenylglycine derivatives 5a and 5c which were formed with up to 96 : 4 e.r. It was demonstrated by comparison with authentic materials that the major enantiomer 5a displayed the (*S*)-configuration. Thus, the mechanistic hypothesis provided in Scheme 2 appears to hold. Hydrogen bonding of the hydrogen atom at the NHBoc group to the amide oxygen atom restricts the conformational freedom and preferentially displays the α-hydrogen atom of the (*R*)-enantiomer *ent*-5a to the benzophenone part of catalyst 1. The importance of hydrogen bonding was underscored by the result obtained with *N*-methyl derivative *rac*-5b which does not display the required hydrogen atom at the amino group and which resulted in a low enantioselectivity.

Other amino acid derivatives 5d–5f were also successfully deracemized under the optimised conditions, although the results were less convincing than for phenylglycines 5a and 5c. The photochemical deracemization was not successful with compounds *rac*-5g to *rac*-5k carrying substituents X = Me, F, Br, CH_2_OMe, and SMe. Very likely, there is no stabilizing effect allowing for a differentiation of the substrate enantiomers. Only the phenylsulfanyl-substituted substrate^19^*rac*-5l resulted in a notable enantioselectivity upon attempted deracemization. The configuration at the stereogenic center of major enantiomer 5l was shown to be (*R*) by comparison with authentic materials (see the SI for details). The result indicates that intramolecular hydrogen bonding to the amide hydrogen atom by the sulfur atom is feasible.

**Scheme 8:**
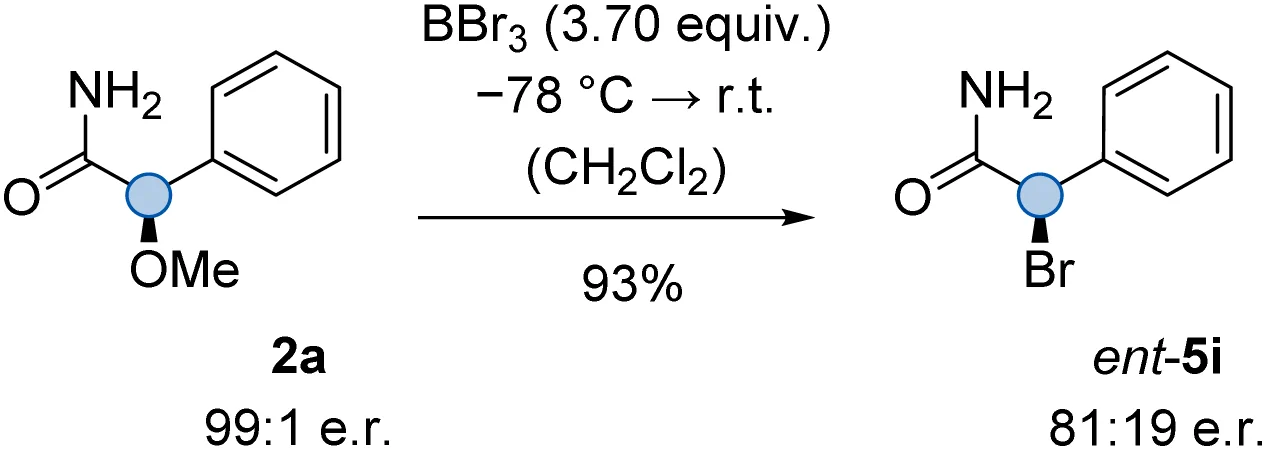
Formation of enantioenriched product *ent*-5i by nucleophilic bromo-de-methoxylation of compound α-methoxy-substituted amide 2a.

Upon attempted deprotection of enantiomerically enriched, (*R*)-configured product 2a, it was found that the methoxy group was replaced by a bromine atom. The reaction proceeded with a partial retention of the configuration, and the α-bromo-substituted amide *ent*-5i was isolated in an 81 : 19 e.r. (Scheme 8). It is likely that the amide group facilitates the displacement by neighbouring group assistance.^20^

**Scheme 9:**
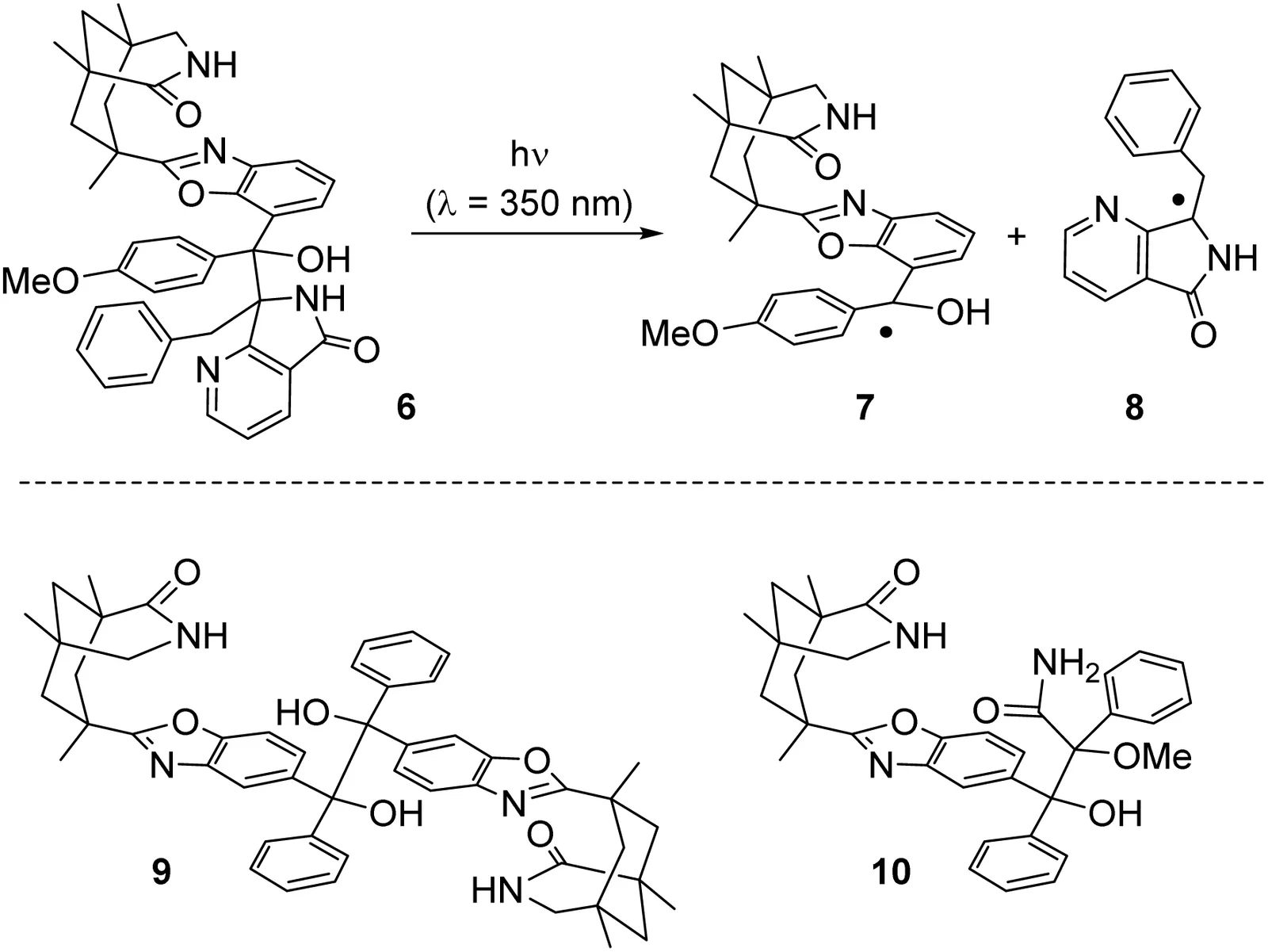
Photochemical cleavage of 1 : 1 adduct 6 to radicals 7 and 8 from which an active benzophenone catalyst can be restored by HAT^21^ and putative by-products 9 and 10 formed from catalyst 1 during the photochemical deracemization reaction of substrate *rac*-2a.

The mechanistic course of photochemical deracemization reactions mediated by catalysts 1 and *ent*-1 has been investigated in previous studies.^8*b*,*d*,*e*^ The bHAT occurs within complex 1′·IM′ (Scheme 2) from the protonated ketyl radical 1′ to the carbonyl oxygen atom of the amide. In recent work on aza-1-isoindolinones, we had seen that it is possible for radicals such as 1′ and IM′ to form a 1 : 1 adduct which acts as a resting state of the catalyst.^21^ In the case of a 4-aza-1-isoindolinone, the 1 : 1 adduct 6 was isolated and it was shown that the compound cleaves upon irradiation into the two components 7 and 8. The catalyst is reformed by a HAT of the protonated ketyl radical 7 to radical 8 (Scheme 9). The latter study alerted us to the important question of consecutive benzophenone products in reversible HAT processes. In earlier work on the photochemical deracemization of *N*-carboxyanhydrides,^22^ we had reisolated *ca.* 25% of benzophenone catalyst 1 after 120 min of irradiation but had not searched for possible consecutive products. In the present study, the formation of possible benzophenone side products was examined in a photochemical deracemization of compound *rac*-2a that was performed with an elevated catalyst loading of 25 mol%. HPLC analyses revealed the formation of two major products after an irradiation time of three hours. The major component displayed an exact mass *m*/*z* = 807.4126 [M + H^+^] by electrospray ionization (ESI) which correlates well with the calculated mass for pinacol 9 (calcd. *m*/*z* = 807.4116). The structure of the 1 : 1 adduct 10 was assigned to the second component based on its exact mass (calcd for [M + H^+^]: *m*/*z* = 568.2806, found: *m*/*z* = 568.2804). The latter compound corresponds to adduct 6 isolated in the 4-aza-1-isoindolinone experiments and is also likely to act in the present case as a resting state of the catalyst. Pinacol 9, which is formed by coupling of two protonated ketyl radicals, requires an oxidant^23^ to restore catalyst 1. Attempts to isolate the compound cleanly were not successful, and traces of benzophenone 1 remained detectable in the material.

Given the relatively large catalyst loading required for the photochemical deracemization of α-branched amides and given the beneficial effect of adding the catalyst in two portions (*cf.*Fig. 1), we hypothesized that catalyst deactivation occurs mainly by formation of pinacol 9. In order to substantiate this hypothesis we initially studied the previously performed deracemization *rac*-2a → 2a (Table 1, entry 1) with the material isolated from the above-mentioned experiment using approx. 10 mol% of pinacol 9 as the potential catalyst. After 13 h of irradiation the enantiomerically enriched product was isolated in an e.r. of only 59 : 41, compared to an e.r. of 84 : 16 if the reaction was performed with catalyst 1.

**Scheme 10:**
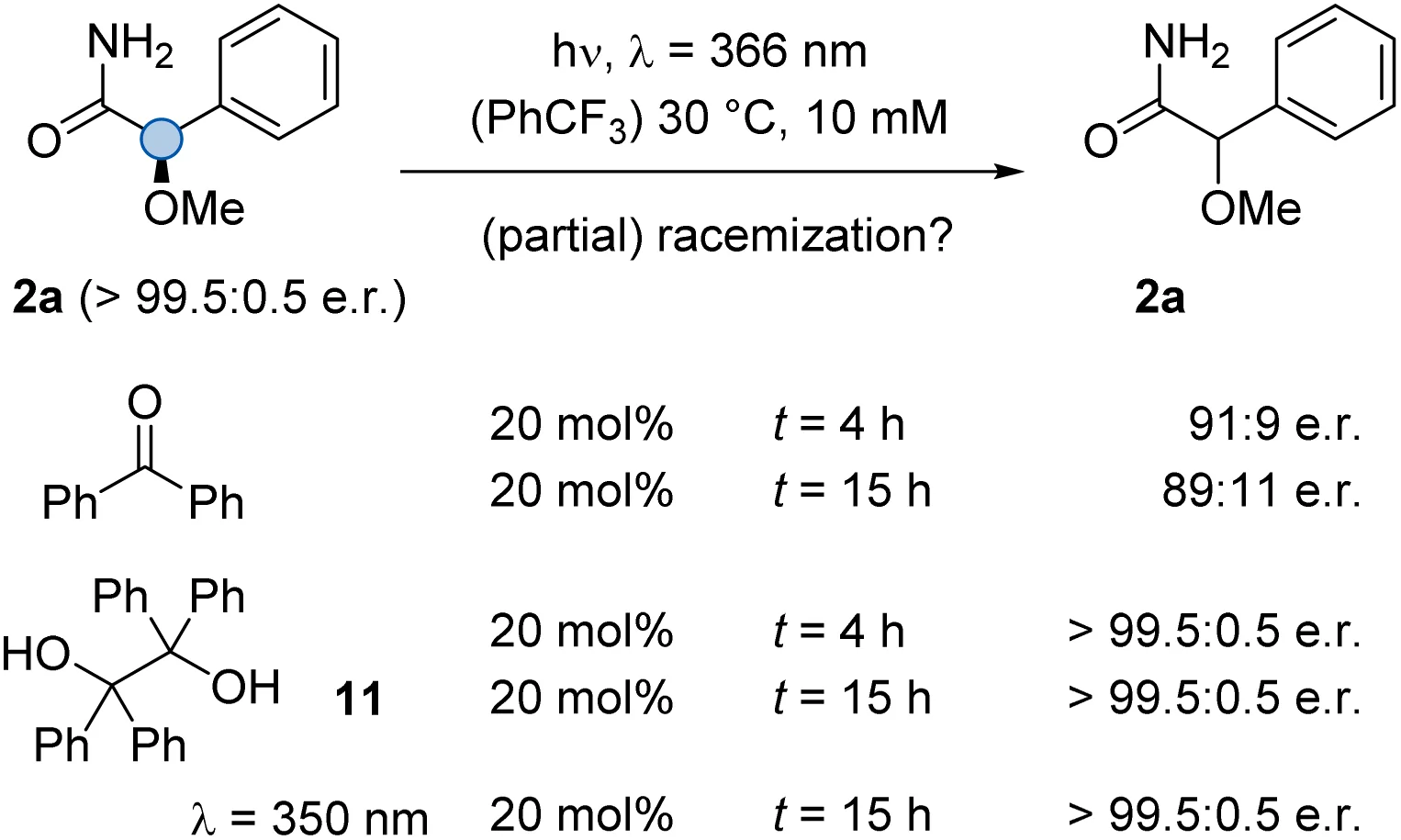
Photochemical racemization of enantiopure substrate 2a mediated by achiral additives: a decrease in e.r. indicates a reversible HAT (see the narrative for details).

Since it appeared as if the minimal turnover in the former reaction was due to the residual benzophenone catalyst, we investigated the influence of parent benzophenone and its corresponding pinacol 11 on the racemization of compound 2a. It was confirmed that no racemization occurs when irradiating the compound at *λ* = 366 nm in trifluorotoluene solution in the absence of any additive. Addition of benzophenone (20 mol%) led to a minor, yet detectable racemization indicating a reversible HAT occurring at the stereogenic center (Scheme 10). If pinacol served as a benzophenone precursor, it should also induce a racemization. However, in the presence of the same quantity of pinacol 11, no racemization was detected. Since the absorption of pinacol 11 is blue-shifted by *ca.* 15 nm ^24^ relative to compound 9, the same experiment was performed at a shorter wavelength (*λ* = 350 nm). There was no detectable racemization, indicating that the pinacol is not competent to regenerate the active benzophenone catalyst under the selected conditions.

Taken together, the results with pinacols 9 and 11 indicate that pinacol 9 is not a resting state of the photocatalyst but rather an undesired by-product of the reaction. Since pinacol formation was less pronounced in the photochemical deracemization of aza-1-isoindolinones, it is likely that in the present case the dissociation of complex 1′·IM′ (Scheme 2) successfully competes with bHAT or formation of 1 : 1 adduct 10. Once the two radicals are separated, pinacol coupling can occur, leading to irreversible catalyst deactivation. Attempts to isolate the corresponding radical coupling products of the intermediate radicals IM′ were not successful.

## Conclusions

In summary, we have developed a protocol for the photochemical deracemization of α-branched chiral carboxamides. The compound class is of immense importance with 117 out of the top selling 200 small-molecule drugs in 2023 containing at least one amide bond.^25^ The deracemization protocol complements the existing arsenal of methods which relies on the preparation of enantiomerically pure carboxamides from achiral^26^ or chiral^4*j*^ starting materials. Key to a high enantioselectivity was the successive addition (2 × 7.5 mol%) of the chiral HAT catalyst 1 to the initially racemic substrates. The best enantioselectivities were obtained for arylacetamides with an alkoxy or an amino substituent in the α-position. In the former case, the alkoxy substituent is likely responsible for intramolecular hydrogen bond formation to the amide NH proton, which is not involved in binding to the catalyst. Consequently, the respective (*R*)-enantiomeric products are formed with high enantioselectivity (up to 99.5 : 0.5 e.r.). In the latter case, the NH proton of the amino group can form a hydrogen bond to the oxygen atom of the amide, rendering the reaction enantioselective towards the corresponding (*S*)-enantiomers (up to 96 : 4 e.r.). The study has shown that a temporary fixation of a specific conformation by hydrogen bonding can render the photochemical deracemization of acyclic substrates by a single chiral photocatalyst possible. Mechanistically, the identification of pinacol by-product 9 resulting from the reductive dimerization of catalyst 1 suggests this pathway to be largely responsible for the deactivation of the catalyst. The observation stresses the relevance of an optimal trajectory for bHAT in photochemical deracemization reactions mediated by single benzophenone catalysts.

## Author contributions

M. I., B. G. and T. B. developed the project. Funding was acquired by T. B. M. I. and B. G. designed and performed the synthetic experiments. M. I. and B. G. generated and validated the experimental data. T. B. administered the project and supervised the research. All authors wrote, reviewed, and edited the manuscript.

## Conflicts of interest

There are no conflicts to declare.

## Supplementary Material

SC-OLF-D6SC05947C-s001

## Data Availability

The data that supports the findings of this study are available in the supplementary information (SI) of this article. Primary research data are openly available in the repository RADAR4Chem at https://doi.org/10.22000/7mw8v2p8dv5su25e. Supplementary information: synthetic procedures and full characterization for all compounds (2a–2r, 3a–3r, and 5a–5l) and spectroscopic data. See DOI: https://doi.org/10.1039/d6sc05947c.
