# Supplementary material for: Photochemical deracemization of α-chiral carboxamides mediated by inter- and intramolecular hydrogen bonding
Source: Chem Sci. 2026 Sep 9;17(41):19746–53. doi: 10.1039/d6sc05947c (PMC13555699; doi:10.1039/d6sc05947c)
Supplement: Supplementary file 1 [file SC-017-D6SC05947C-s001.pdf]

Supplementary Information (SI) for Chemical Science.
This journal is © The Royal Society of Chemistry 2026

Electronic Supplementary Information

Photochemical deracemization of α-chiral carboxamides mediated by inter- and intramolecular hydrogen bonding

Maximilian Iglhaut,[#a] Biki Ghosh[#a] and Thorsten Bach[*a]

Technische Universität München, School of Natural Sciences, Department of Chemistry and Catalysis Research Center, Lichtenbergstrasse 4, 85747 Garching, Germany

[#]These authors contributed equally to this work.

Corresponding author: Email: thorsten.bach@ch.tum.de

Table of Contents

S1. General Information 3
S2. Analytical Methods 7
S3. General Synthetic Procedures 9
S5. Optimization of the Reaction Conditions and Profile of the Deracemization Reaction 15
S6. Determination of the Absolute Configuration of Deracemization Products 18
S9. Synthesis and Characterization of Deracemization Substrates 26
S10. Photochemical Deracemization Reactions 56
S11. Stereochemical Editing 83
S12. Conversion of **2a** to *ent*-**5i** 87
S13. Detection and Attempted Isolation of Catalyst Coupling Products **9** and **10** 88
S14. Deracemization of *rac*-**2a** with **9** 91
S15. Racemization Studies 92
S16. NMR Spectra 94
S17. HPLC Traces 154
S18. List of References 207

S1. General Information

All reactions sensitive to air or moisture, were carried out in flame-dried glassware under positive pressure of argon using standard Schlenk techniques.

Commercially available chemicals were used without further purification, if not mentioned otherwise. For moisture sensitive reactions, dichloromethane (CH_2Cl_2), diethyl ether (Et_2O) and tetrahydrofuran (THF) were purified using a MBSPS 800 *MBraun* solvent purification system. The following columns were used:

CH_2Cl_2: 2 × MB-KOL-A type (aluminum oxide)

Et_2O: 1 × MB-KOL-A type 2 (aluminum oxide), 1 × MB-KOL-M type 2 (3 Å molecular sieve)

THF: 2 × MB-KOL-M type 2 (3 Å molecular sieve)

Anhydrous *α,α,α*-trifluorotoluene ($PhCF_3$) and a 3 M solution of HCl in methanol (MeOH) were purchased from *Sigma Aldrich (Merck)*. $PhCF_3$ was additionally stored over 3 Å molecular sieves. Anhydrous acetonitrile (MeCN), dimethyl sulfoxide (DMSO), MeOH and ethanol (EtOH) were purchased from *Thermo Fisher Scientific* and stored over 3 Å molecular sieves.

Technical solvents for column chromatography [dichloromethane (CH_2Cl_2), ethyl acetate (EtOAc), methanol (MeOH), hexanes, *n*-pentane (pentane)] were used after simple distillation.

Normal-phase flash column chromatography (FCC) was performed on silica 60 (*Merck*, 230-400 mesh) with the indicated eluent mixture.

Commercially available starting materials were purchased either from *Sigma Aldrich (Merck)*, *TCI Chemicals*, *ABCR* or *BLDpharm*.

Unless water was used, round bottom flasks, vials and phototubes were dried with a heat gun at approximately 600 °C under vacuum.

Unless otherwise stated, photochemical reactions at λ = 366 nm and λ = 350 nm were carried out in Duran phototubes (∅ = 1 cm, 10 mL) under argon atmosphere in a positive geometry setup with a cylindrical array of 16 fluorescent light tubes, UV-A, λ_{max} = 366 nm and λ_{max} = 350 nm (Figure S1, S2).

Prior to the start of a photoreaction, each reaction mixture was degassed by being sparged with argon under ultrasonication for 15 min.

Lehrstuhl OC 1 - TUM

200 nm | 250 nm | 300 nm | 350 nm | 400 nm | 450 nm | 500 nm | 550 nm | 600 nm | 650 nm

Datasheet FLT024 — Philipps-BLB-365

Basic Information

Type	Fluorescent light tube
Description	Philipps TL 8W BLB
Manufacturer / Supplier	Philipps / Beleuchtungdirekt.de
Order number / Date of purch.	n/a / 02/2021
Internal lot / serial number	2021-02 / FLT024

Specification Manufacturer

Type / size	T5 tube, G5 socket
Mechanical specification	16 mm diameter, 288 mm length
Electrical specification	8 W
Wavelength (range, typ.)	350 - 400 nm
Spectral width (FWHM)	~ 16 nm
Datasheet	n/a

Characterization

Description of measurement	Measured with Ocean-optics USB4000 spectrometer using a calibrated setup (cosine corrector/fibre). The cosine corrector was placed at 20 mm distance from a single fluorescent tube at half height.	
Measured dominant wavelength / Int.	365 nm	168 µW/mm²nm
Measured spectral width (FWHM)	16 nm	
Integral Reference intensity / range	3059 µW/cm²	300-450 nm

Spectrum

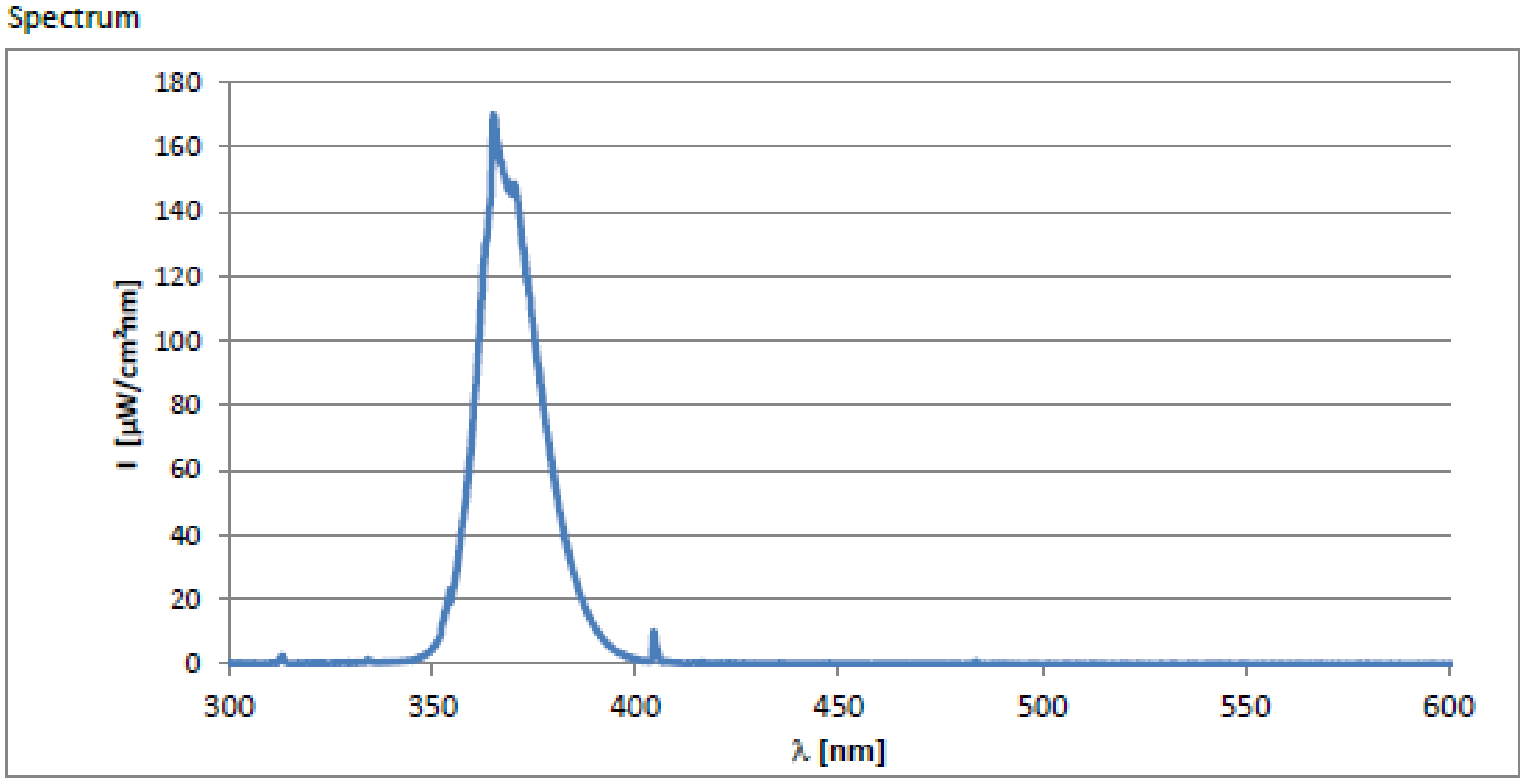

Figure S1: Emission spectrum of the 366 nm fluorescent light tube.

Lehrstuhl OC 1 - TUM

200 nm | 250 nm | 300 nm | 350 nm | 400 nm | 450 nm | 500 nm | 550 nm | 600 nm | 650 nm

Datasheet FLT021 — LZC-UVA

Basic Information

Type	Fluorescent light tube
Description	Luzchem LZC-UVA
Manufacturer / Supplier	Hitachi / Luzchem
Order number / Date of purch.	LZC-UVA / 09/2015
Internal lot / serial number	2015-09 / FLT021

Specification Manufacturer

Type / size	T5 tube, G5 socket
Mechanical specification	16 mm diameter, 288 mm length
Electrical specification	8 W
Wavelength (range, typ.)	300 - 400 nm, 350m nm, UV-A
Spectral width (FWHM)	~ 40 nm
Datasheet	

Characterization

Description of measurement	Measured with Ocean-optics USB4000 spectrometer using a calibrated setup (cosine corrector/fibre). The cosine corrector was placed at 20 mm distance from a single fluorescent tube at half height.	
Measured dominant wavelength / Int.	350 nm	115 µW/mm²nm
Measured spectral width (FWHM)	40 nm	
Integral Reference intensity / range	5017 µW/cm²	300-425 nm

Spectrum

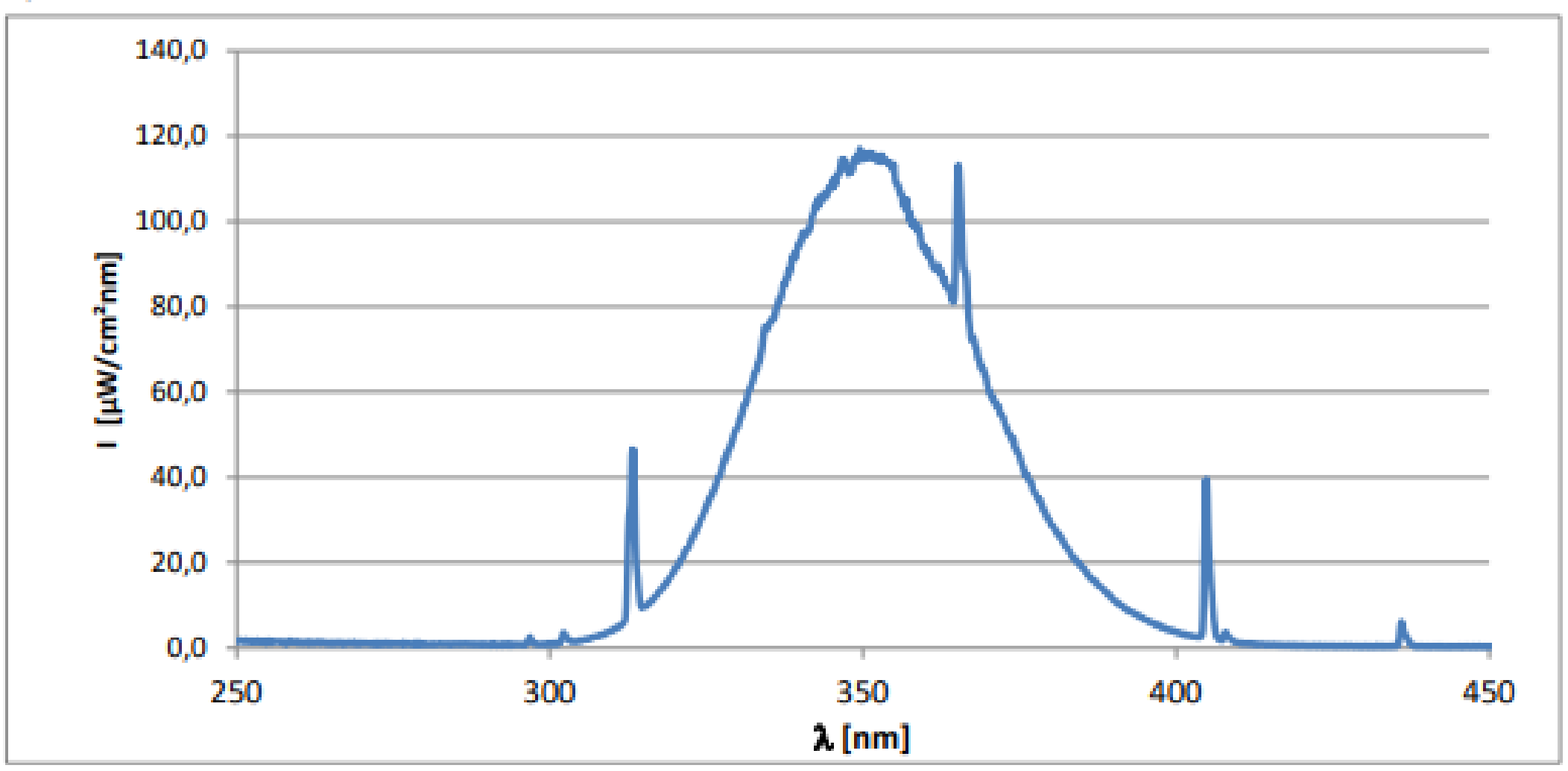

Figure S2: Emission spectrum of the 350 nm fluorescent light tube.

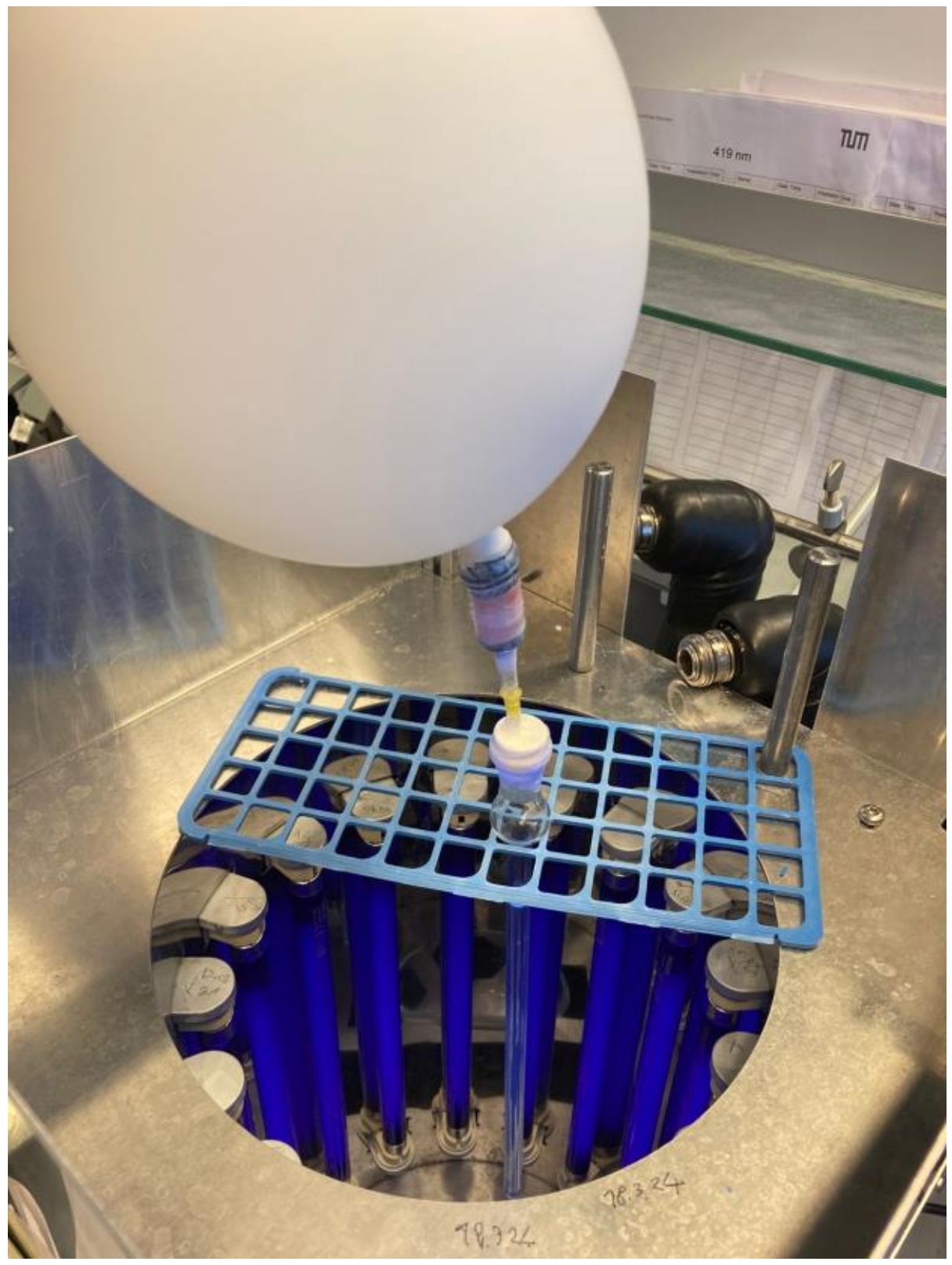

Figure S3: Typical setup for a photochemical deracemization reaction.

S2. Analytical Methods

Thin layer chromatography (TLC) was performed on silica coated glass plates (silica gel 60 F_{254}) with detection by UV-light (λ = 254 nm) and potassium permanganate stain [$KMnO_4$].

Infrared spectra (IR) were recorded on a Perkin Elmer Frontier IR-FTR spectrometer or on an Agilent Cary 630 FTIR spectrometer by ATR technique. The signal intensity is assigned using the following abbreviations: s (strong), m (medium), w (weak). The following abbreviations were used: aliph = aliphatic, arom = aromatic.

$KMnO_4$-staining solution: Potassium permanganate (3.00 g), potassium carbonate (20.0 g), aqueous NaOH solution (5%, 5.0 mL), water (300 mL).

Melting points (M.p.) were determined using a Kofler ("Thermopan", Fs *Reichert*, Wien) apparatus.

Nuclear magnetic resonance (NMR) (^{1}H, ^{13}C and ^{19}F-NMR) spectra were recorded at room temperature (r.t.) on either a *Bruker* AVHD-400, AVHD-500, or a *Bruker* AV-II-500 equipped with cryo probe head. Chemical shifts of the NMR spectra are reported relative to $CHCl_3$ (^{1}H-NMR: δ = 7.26 ppm, ^{13}C-NMR: δ = 77.16 ppm), MeOH (^{1}H-NMR: δ = 3.31 ppm, ^{13}C-NMR: δ = 49.00 ppm) or DMSO (^{1}H-NMR: δ = 2.50 ppm, ^{13}C-NMR: δ = 128.06 ppm). The data are reported as follows: chemical shift (δ) [multiplicity, coupling constant *J* (Hz), relative integral, number of protons] where multiplicity is defined as: m = multiplet, s = singlet, d = doublet, t = triplet, q = quartet, sept = septet, br broad. Apparent multiplets which occur as a result of coupling constant equality between magnetically non-equivalent protons are marked as virtual (*virt.*).

Mass spectrometry (MS) and **high-resolution mass spectrometry (HRMS)** were measured on a *Thermo Scientific* LTQ-FT Ultra [electrospray ionization (ESI)].

Specific Rotation was determined using an ADP440+ polarimeter (Fa *Bellingham+Stanley*) and is reported as follows: $[\alpha]_D^T$ (c in g per 100 mL solvent). The polarimeter has a variance of ± 0.001 which translates to a variance of ± 2 of the measured rotation for c = 1.0 and a cuvette path length of 0.5 cm.

High Performance Liquid Chromatography (HPLC) was performed using a chiral stationary phase [ChiralPak AD-H (250 x 4.6 mm), Chiralpak IC (250 x 4.6 mm), ChiralPak AS-H (250 x 4.6 mm), ChiralPak IA (250 x 4.6 mm), ChiralPak OJ-RH (150 x 4.6 mm), *Daicel Chemical Industries*] with UVD 340 Photodiode Array Detector, P580 Pump and an ASI-100

Automated Sample Injector at 20 ºC. For normal-phase HPLC a *Daicel* ChiralPak AD-H, ChiralPak IC, ChiralPak AS-H and ChiralPak IA were used as the stationary phase with a mixture of *n*-heptane/*i*-propanol as the mobile phase or a *Daicel* ChiralPak OJ-RH were used as the stationary phase with a mixture of MeCN/water as the mobile phase.

S3. General Synthetic Procedures

General Procedure A (GP A): Synthesis of racemic 2-substituted phenylacetamides (*rac*-2/*rac*-5) from 2-substituted phenylacetic acids

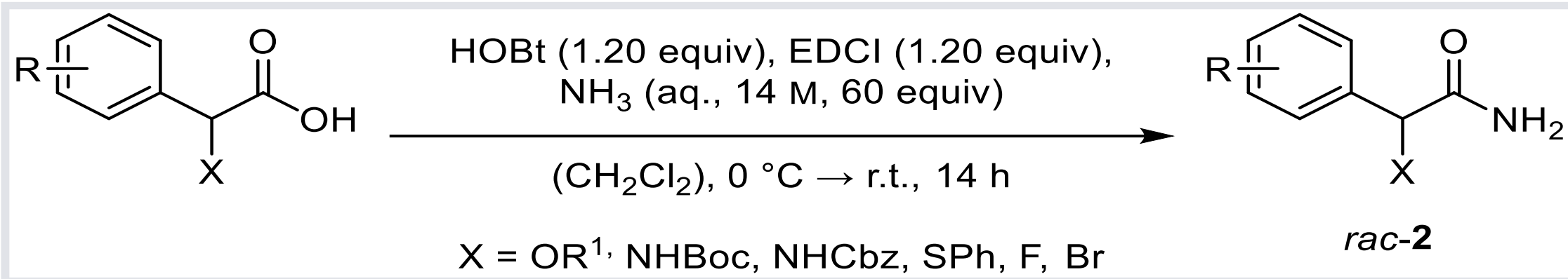

Following a procedure by Plaza *et al.,*[1] 1-hydroxybenzotriazole hydrate (HOBt · H_2O) (1.20 equiv.) was added to a solution of the respective 2-substituted phenylacetic acid (1.00 equiv.) in CH_2Cl_2 (0.03 M) at 0 °C. After 10 min, *N*-(3-dimethylaminopropyl)-*N'*-ethylcarbodiimide-hydrochloride (EDCI · HCl) was added. After stirring the resulting mixture at 0 °C for another 30 min, aq. ammonia (14 M, 60.0 equiv.) was added before the reaction mixture was allowed to warm to r.t. and stirred vigorously for 14 h. Then, water was added, the layers were separated and the aqueous layer was extracted thrice with CH_2Cl_2. The combined organic layers were washed with brine and dried over Na_2SO_4. The solvents were removed under reduced pressure, and the crude product was subjected to flash column chromatography (FCC) (SiO_2, EtOAc/hexanes) to yield the racemic 2-substituted phenylacetamides (*rac*-**2**/*rac*-**5**) as white solids.

General Procedure B (GP B): Two-step synthesis of 2-alkoxy-2-arylacetamides from their corresponding arylaldehydes

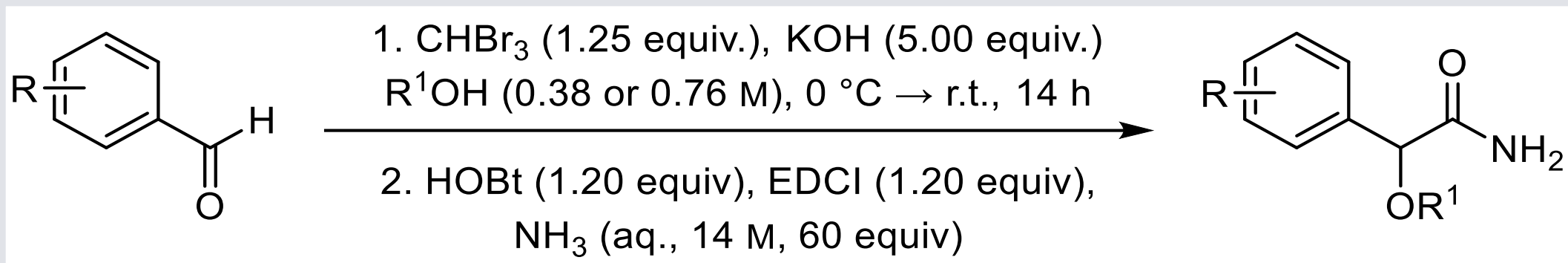

rac-**2**/*rac*-**3**

Following a modified procedure by Woods *et al.,*[2] KOH pellets (5.00 equiv.) were added in portions over a span of 10 min to a solution of the respective aldehyde and bromoform (1.25 equiv.) in the respective alcohol (R^1OH) at 0 °C. The resulting suspension was stirred at 0 °C for 30 min, before it was allowed to warm to r.t. and stirred at this temperature for another 14 h. Subsequently, the solvent was removed under reduced pressure, water was added and the resulting solution was washed thrice with Et_2O before the pH value of the aqueous layer was adjusted to 1 by addition of aq. HCl (2.0 M). The resulting suspension was then extracted thrice with EtOAc and the organic layer was washed with brine and dried over Na_2SO_4.

Following a procedure by Plaza *et al.,*[1] 1-hydroxybenzotriazole hydrate (HOBt · H_2O) (1.20 equiv.) was added to a solution of the respective 2-alkoxy-2-arylacetic acid (1.00 equiv.) in CH_2Cl_2 (0.03 M) at 0 °C. After 10 min, *N*-(3-dimethylaminopropyl)-*N'*-ethylcarbodiimide-hydrochloride (EDCI · HCl) was added. After stirring the resulting mixture at 0 °C for another 30 min, aq. ammonia (14 M, 60.0 equiv.) was added before the reaction mixture was allowed to warm to r.t. and stirred vigorously for 14 h. Then, water was added, the layers were separated and the aqueous layer was extracted thrice with CH_2Cl_2. The combined organic layers were washed with brine and dried over Na_2SO_4. The solvents were removed under reduced pressure, and the crude product was subjected to flash column chromatography (FCC) (SiO_2, EtOAc/hexanes) to yield the racemic 2-alkoxy-2-arylacetamides (*rac*-**2**/*rac*-**3**) as white solids.

General Procedure C (GP C): Two-step synthesis of racemic 2-methoxy-2-phenylacetamides (*rac*-2) from 2-hydroxy-2-phenylacetic acids

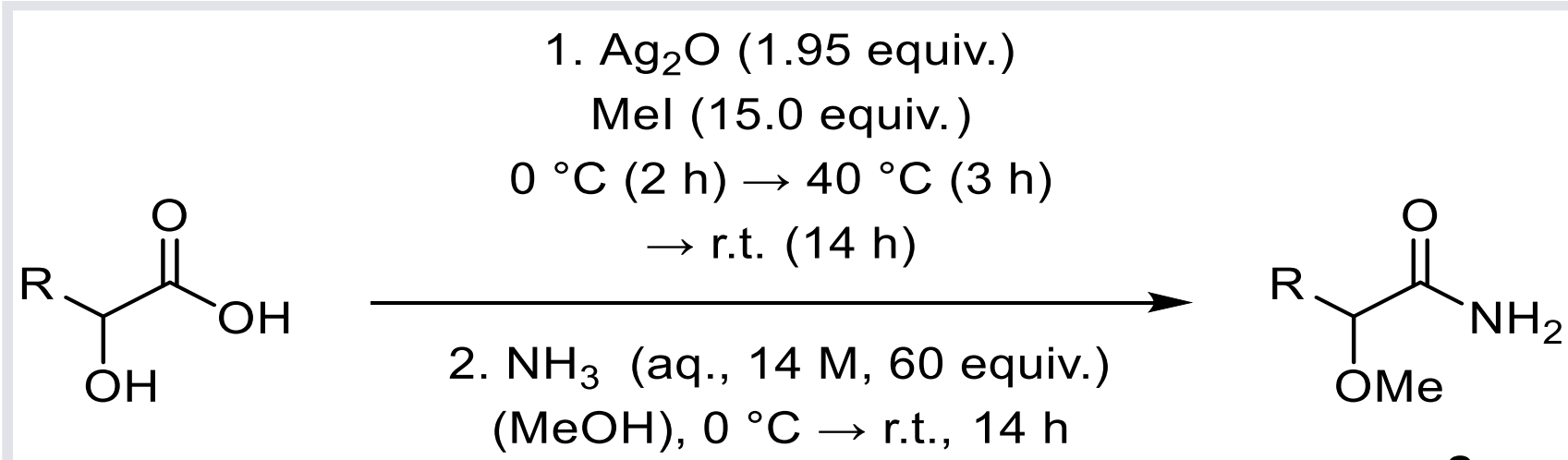

rac-**2**

Following a procedure by McKenzie[3] and Watson *et al.,*[4] a solid mixture of silver oxide (2.00 equiv.) and the respective 2-hydroxy-substituted carboxylic acid (1.00 equiv.) was added in portions to MeI (15.0 equiv.) at 0 °C. After 2 h the resulting mixture was heated to 40 °C for 3 h and cooled to ambient conditions. After stirring at r.t. for another 14 h, Et_2O (5 mL) was added and filtered through cotton. The filtrate was concentrated under reduced pressure. Subsequently the mixture was taken in MeOH (0.50 M) at 0 °C followed by the addition of aq. ammonia (14 M, 60.0 equiv.). The resulting mixture was stirred at r.t. for 14 h. All the volatiles were removed under reduced pressure. Water (10 mL) and CH_2Cl_2 (10 mL) were added, the layers were separated and the aqueous layer was extracted thrice with CH_2Cl_2 (10 mL). The combined organic layers were washed with brine and dried over Na_2SO_4. The solvents were removed under reduced pressure, and the crude product was subjected to flash column chromatography (FCC) (SiO_2, EtOAc/hexanes) to yield the racemic 2-methoxy-substituted primary amides (*rac*-**2**) as white solids.

General Procedure D (GP D): Synthesis of racemic 2-alkoxy-2-phenylacetamides (*rac*-3) from methyl 2-hydroxy-2-phenylacetate

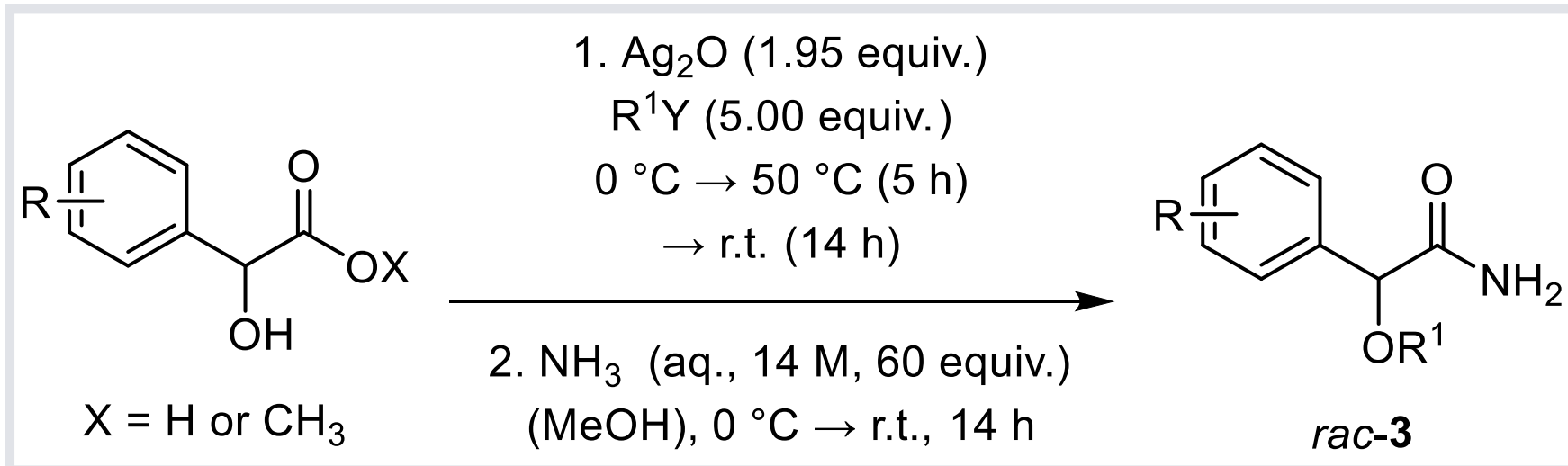

Following a procedure by McKenzie[3] and Watson *et al.,*[4] and Ghosh *et.al.*,[5] a solid mixture of silver oxide (2.00 equiv.) and the respective 2-hydroxy-2-phenylacetic acid (1.00 equiv.) was added in portions to R^1X (5.00 equiv.) at 0 °C. The resulting mixture was heated to 50 °C for 5 h and cooled to ambient conditions and stirred for additional 14 h. Then, Et_2O (5 mL) was added and filtered through cotton. The filtrate was concentrated under reduced pressure. Subsequently the mixture was taken in MeOH (0.50 M) at 0 °C followed by the addition of aq. ammonia (14 M, 60.0 equiv.). The resulting mixture was stirred at r.t. for 14 h. All the volatiles were removed under reduced pressure. Water (10 mL) and CH_2Cl_2 (10 mL) were added, the layers were separated and the aqueous layer was extracted thrice with CH_2Cl_2 (10 mL). The combined organic layers were washed with brine and dried over Na_2SO_4. The solvents were removed under reduced pressure, and the crude product was subjected to flash column chromatography (FCC) (SiO_2, EtOAc/hexanes) to yield the racemic 2-alkoxy-2-phenylacetamides (*rac*-**3**) as white solids.

General Procedure E (GP E): Photochemical deracemizations of amides (*rac*-2/*rac*-3)

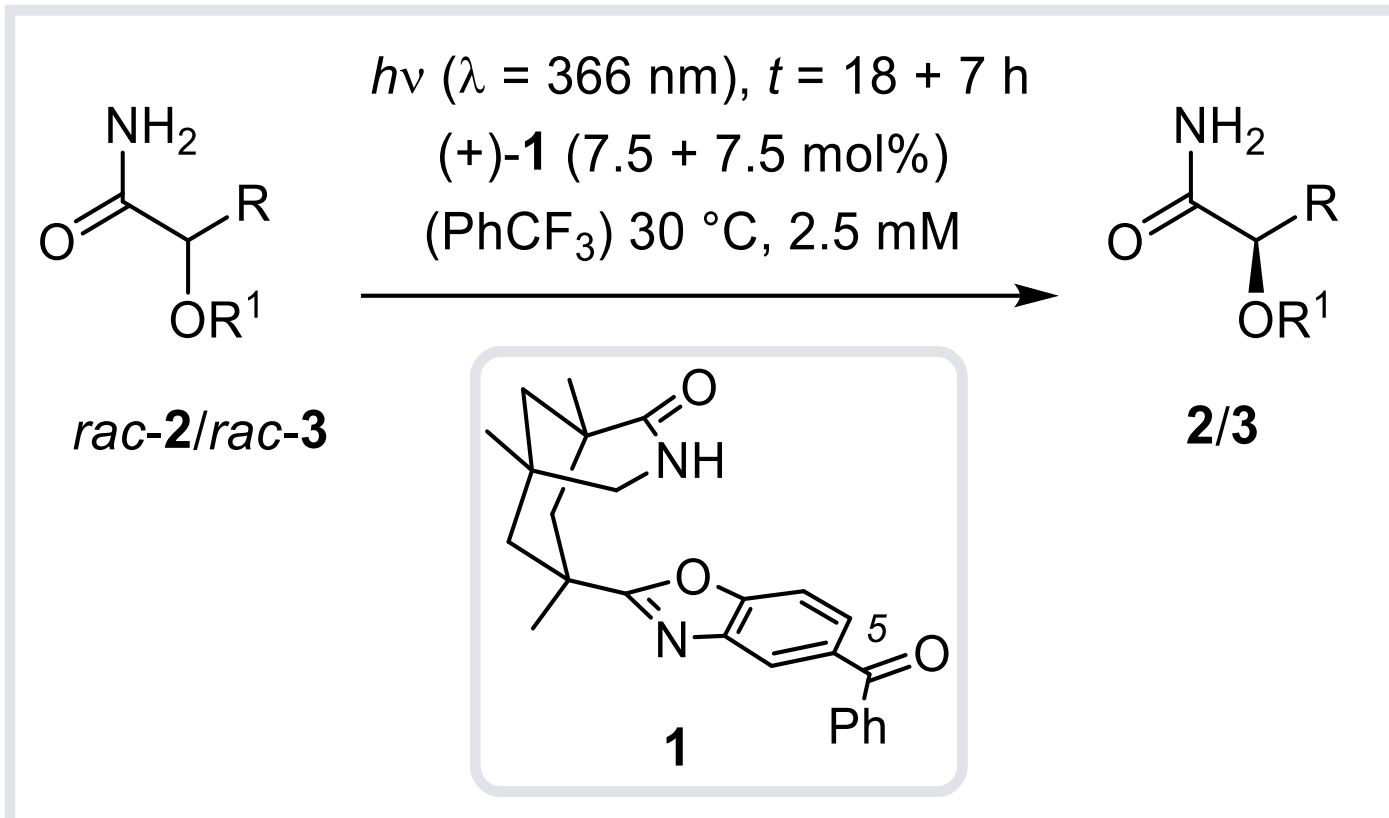

A solution of the corresponding racemic amide *rac*-**2** or *rac*-**3** (25.0 µmol, c = 2.5 mM, 1.00 equiv.) and enantiomerically pure (+)-benzophenone **1** (7.50 mol%) in dry $PhCF_3$ was degassed by being sparged with argon under ultrasonication for 15 minutes. The solution was irradiated at λ = 366 nm at 30 °C for 18 h. Another portion of catalyst **1** (7.50 mol%) was added and the solution was again degassed for 15 min and then irradiated at λ = 366 nm for 7 h. After irradiation, all the volatile compounds were removed under reduced pressure and the crude products were subjected to FCC (SiO_2, EtOAc/hexanes) to afford the enantioenriched (*R*)-amides (**2**/**3**).

General Procedure F (GP F): Photochemical deracemizations of amides (*rac*-5)

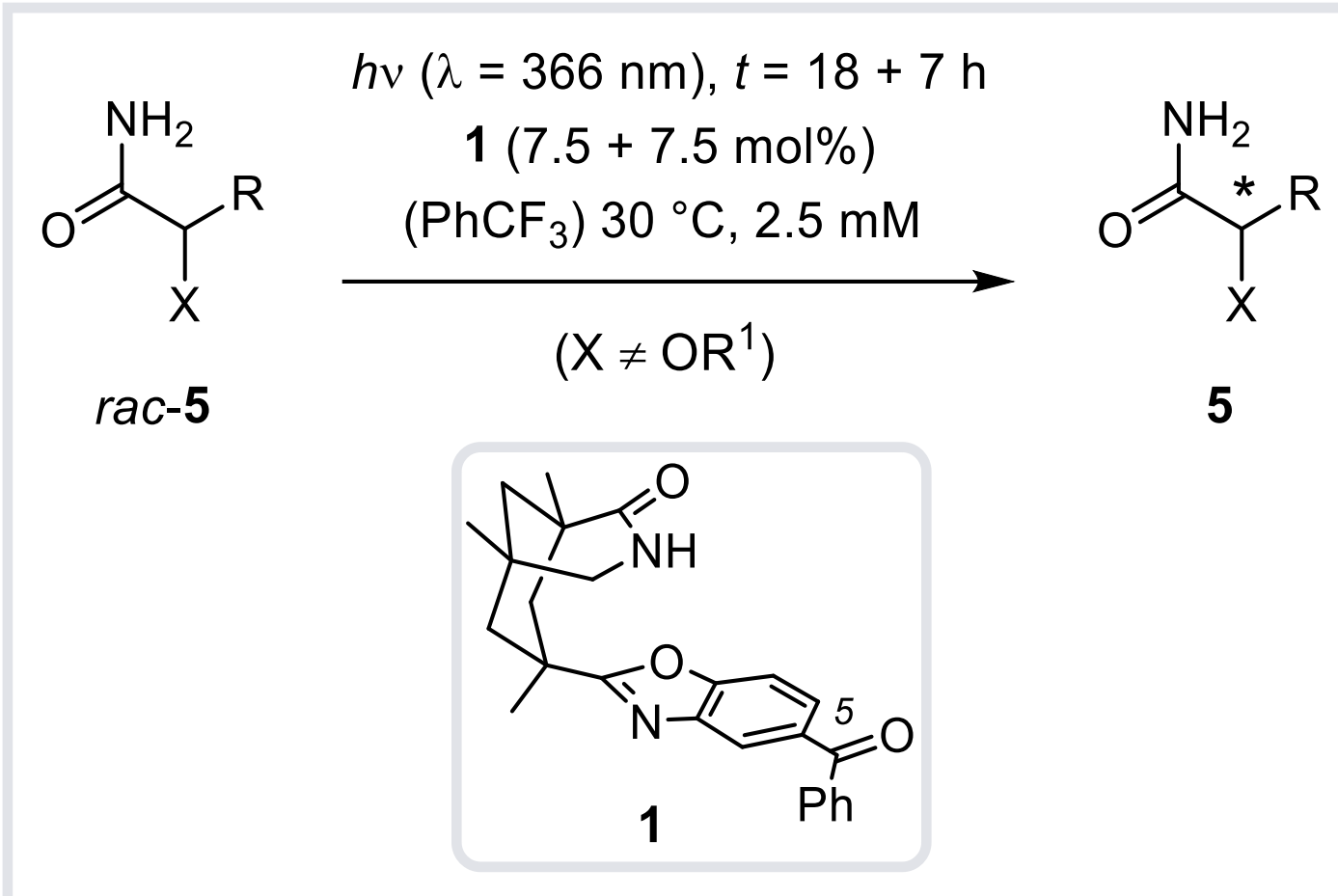

A solution of the corresponding racemic amide *rac*-**5** (25.0 µmol, c = 2.5 mM, 1.00 equiv.) and enantiomerically pure (+)-benzophenone **1** (7.50 mol%) in dry $PhCF_3$ was degassed by being sparged with argon under ultrasonication for 15 minutes. The solution was irradiated at λ = 366 nm at 30 °C for 18 h. Another portion of catalyst **1** (7.50 mol%) was added and the solution was again degassed for 15 min and then irradiated at λ = 366 nm for 7 h. After irradiation, all the volatile compounds were removed under reduced pressure and the crude products were subjected to FCC (SiO_2, EtOAc/hexanes) to afford the enantioenriched amides (**5**).

S5. Optimization of the Reaction Conditions and Profile of the Deracemization Reaction

The reaction conditions were first optimized for 2-methoxy-2-phenylacetamide (*rac*-**2a**) using catalyst (+)-**1** (Table **S1**) as well as thiols **S-1a** and S-**1b** as potential hydrogen atom shuttle additives.

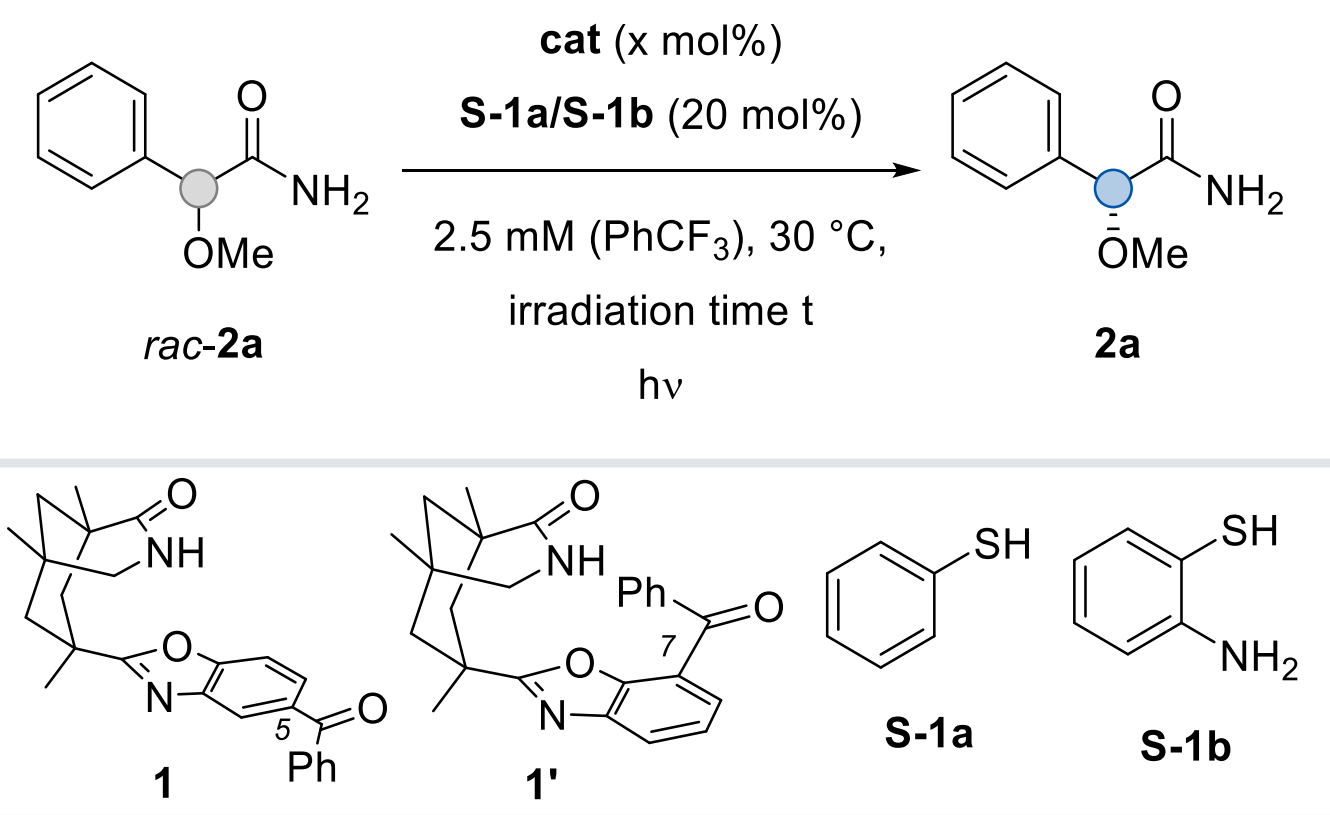

Table S1: Optimization of reaction conditions for the deracemization of *rac*-**2a**. All reactions were performed on a 25.0 µmol scale.

Entry	cat	x [mol%]	S	λ [nm]	t [h]	yield [%]	e.r.
1	**1**	10.0	-	366	13	97	84:16
2	**1′**	10.0	-	366	13	94	59:41
3	**1**	10.0	-	350	13	quant.	81:19
4	**1**	15.0	-	366	13	91	89.5:10.5
5	**1**	10.0	-	366	18	95	90.5:9.5
6	**1**	7.5	-	366	18	quant.	79.5:20.5
7	**1**	10.0	-	366	24	91	90.5:9.5
8	**1**	10.0	**S-1a**	366	18	79	85:15
9	**1**	10.0	**S-1b**	366	18	88	87.5:12.5
10	**1**	5.0	**-**	366	18	92	76.5:23.5
11	**1**	12.5	**-**	366	18	87	90.5:9.5
12	**1**	15.0	**-**	366	18	88	93:7
13	**1**	15.0	**-**	366	24	87	92.5:7.5
14	**1**	7.5+7.5	**-**	366	18+18	95	95:5

Given the improved e.r. for a portionwise addition of 15 mol% of **1** (entry 14) compared to entry 12, we investigated the rate profile of the deracemization of *rac*-**2a** after the addition of the second portion of **1**. For this, aliquots of the reaction mixture were taken at different time points and the enantiomeric ratio (e.r.) of **2a** was determined by chiral HPLC analysis (table S**2**).

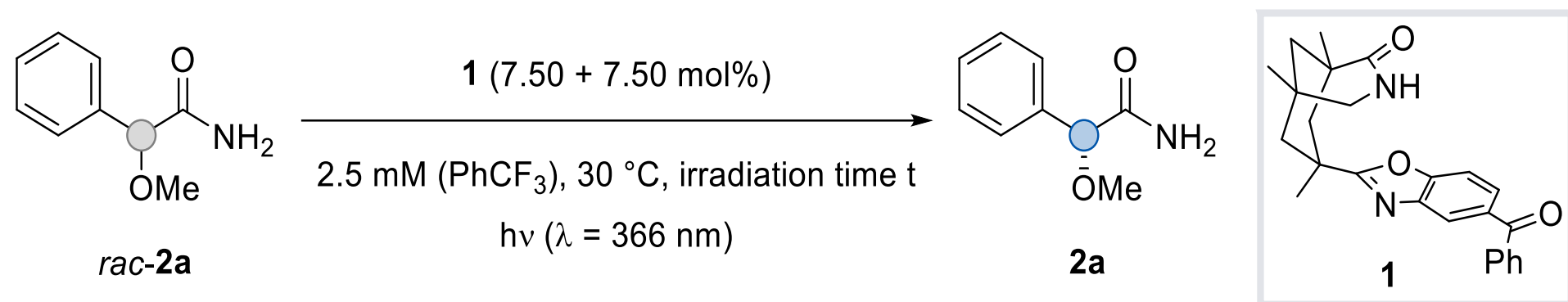

Table S2: Rate profile of the deracemization of *rac*-**2a** with chiral photocatalyst **1**.

t [h]	e.r.
18	79.5:20.5
18.5	89.5:10.5
19	88.5:11.5
20	91:9
22	92.5:7.5
23	93.5:6.5
24	93:7
25	94.5:5.5
26	93.5:6.5
27	93.5:6.5
28	93:7
36	92:8

The rate profile of the deracemization of *rac*-**2a** showed a maximum e.r. after a total irradiation time of 25 h (7 h after the addition of the second portion of **1**). After that, a drop in e.r. was observed. We assume that the overall lower enantioselectivity observed in the determination of the rate profile compared to e.r.s observed for the initial reaction optimization (table S**2** can be explained by the oxygen sensitivity of the photoreaction and the introduction of oxygen into the phototube by repeatedly taking aliquots of the reaction mixture. Based on these results, the highest e.r. of **2a** could then be obtained by adding catalyst **1** in two portions with irradiation times of 18 and seven hours (25 h in total).

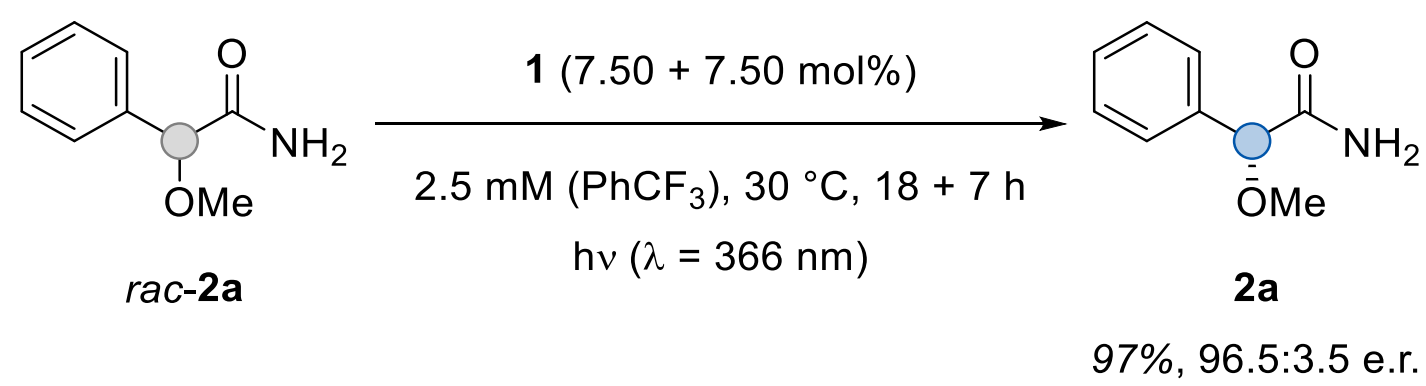

S6. Determination of the Absolute Configuration of Deracemization Products

The absolute configuration of 2-methoxy-2-phenylacetamide (**2a**) was determined by comparison of the retention times of **2a** with authentic **2a**, which was prepared from commercially available (*R*)-2-methoxy-2-phenylacetic acid following GP **A**, on chiral HPLC.

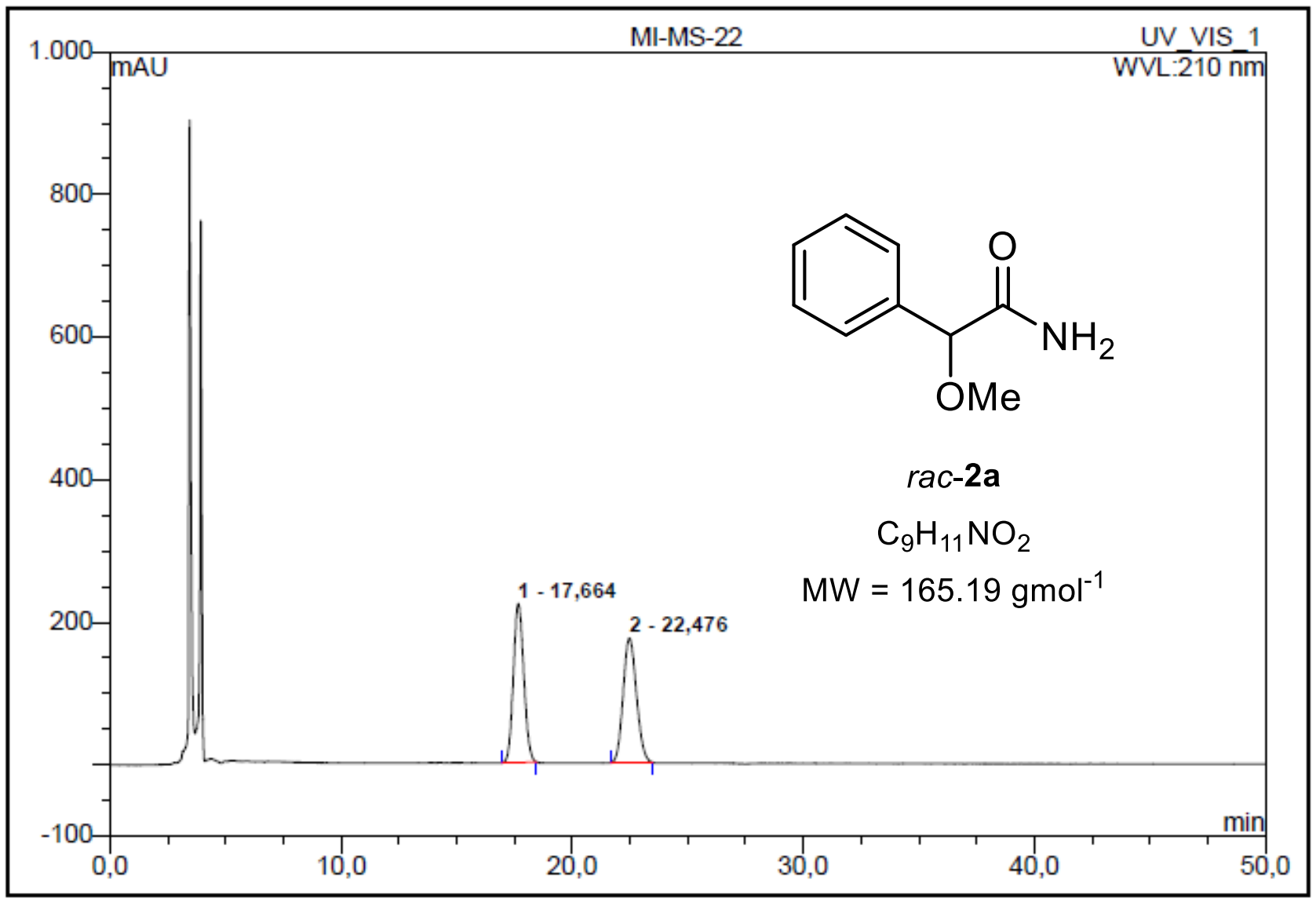

No.	Ret.Time min	Peak Name	Height mAU	Area mAU*min	Rel.Area %	Amount	Type
1	17,66	n.a.	222,715	116,866	50,09	n.a.	BMB
2	22,48	n.a.	174,568	116,454	49,91	n.a.	BMB
Total:			397,283	233,320	100,00	0,000	

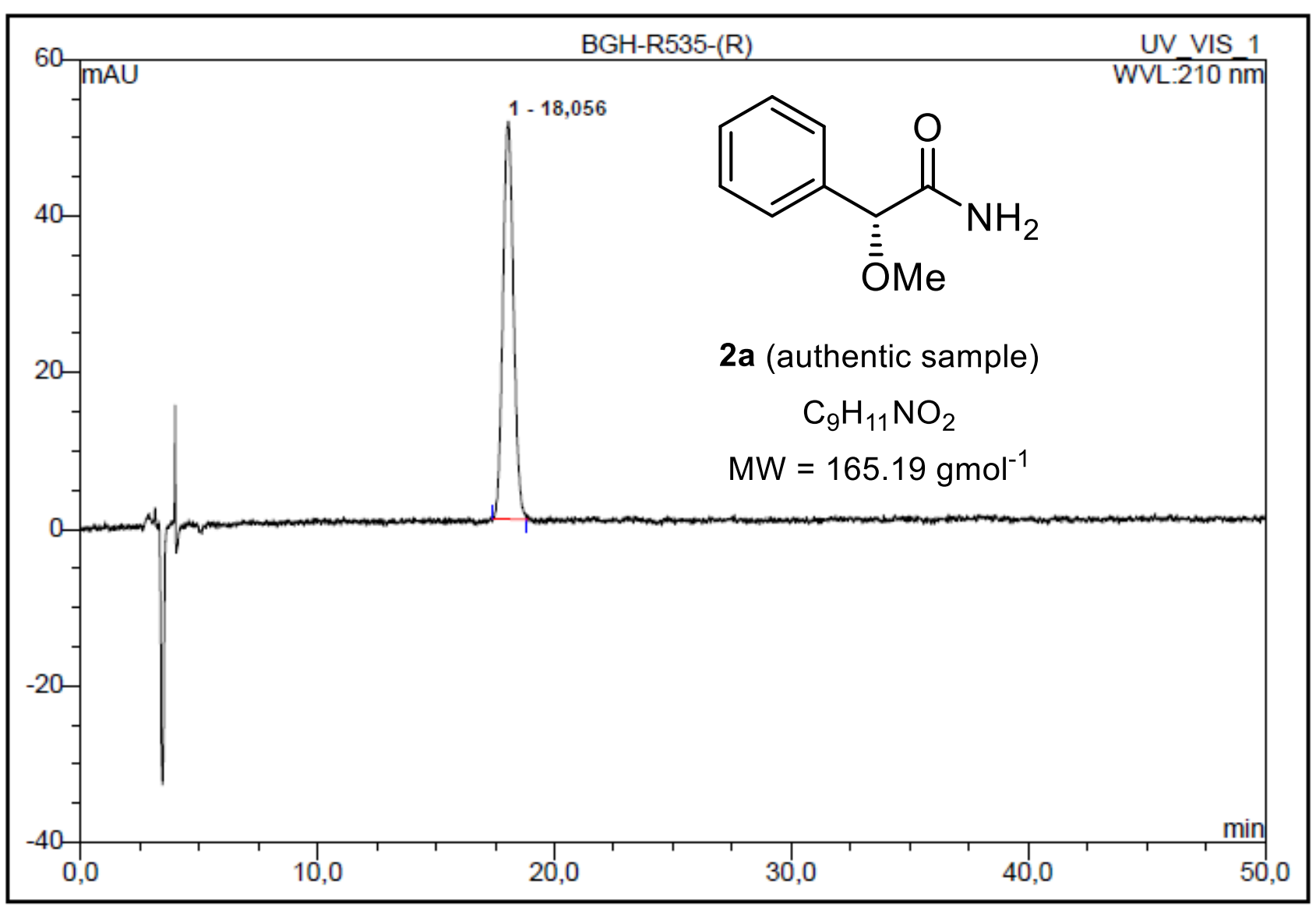

No.	Ret.Time min	Peak Name	Height mAU	Area mAU*min	Rel.Area %	Amount	Type
1	18,06	n.a.	50,788	26,332	100,00	n.a.	BMB*
Total:			50,788	26,332	100,00	0,000	

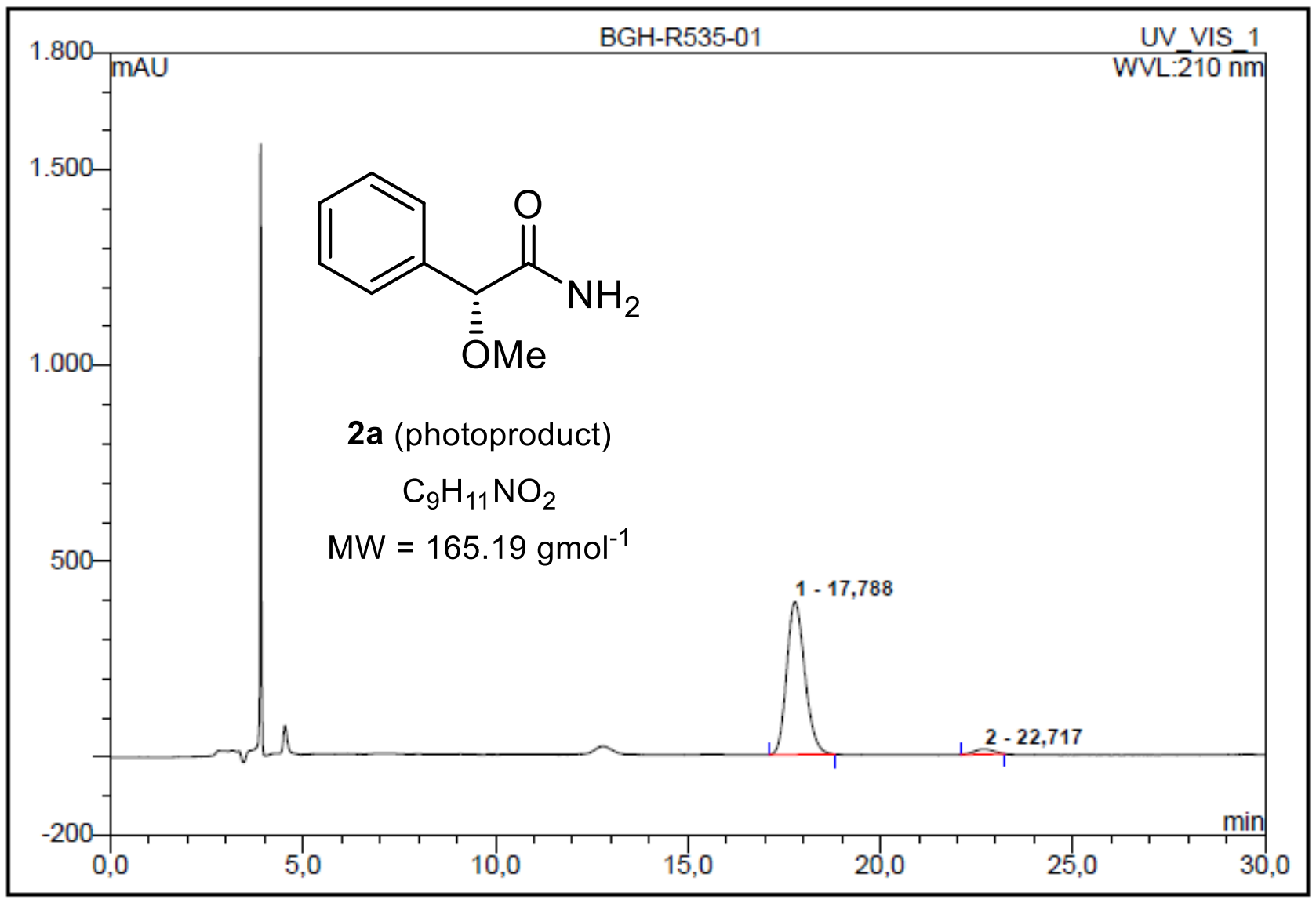

No.	Ret.Time min	Peak Name	Height mAU	Area mAU*min	Rel.Area %	Amount	Type
1	17,79	n.a.	390,353	213,907	96,50	n.a.	BMB
2	22,72	n.a.	13,206	7,759	3,50	n.a.	BMB*
**Total:**			403,559	221,666	100,00	0,000	

The absolute configuration of *tert*-butyl (2-amino-2-oxo-1-phenylethyl)carbamate (**5a**) was determined as (*S*) by comparison of the retention times of **5a** with *ent*-**5a**, which was prepared from commercially available (*R*)-*tert*-butyl (2-amino-2-oxo-1-phenylethyl)carbamate following GP **A**, on chiral HPLC.

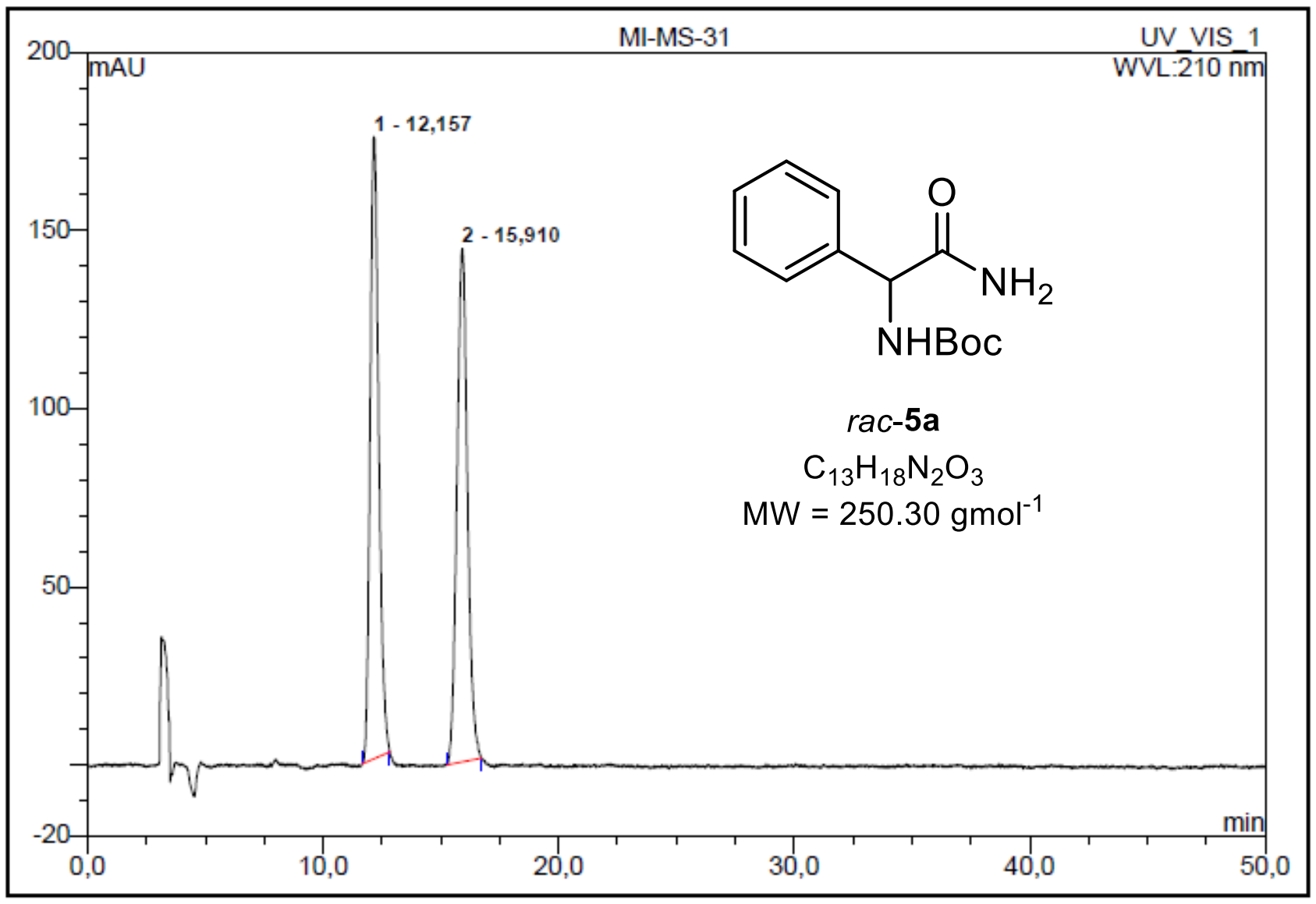

No.	Ret.Time min	Peak Name	Height mAU	Area mAU*min	Rel.Area %	Amount	Type
1	12,16	n.a.	174,579	73,678	49,69	n.a.	BMB
2	15,91	n.a.	143,976	74,608	50,31	n.a.	BMB
Total:			318,554	148,287	100,00	0,000	

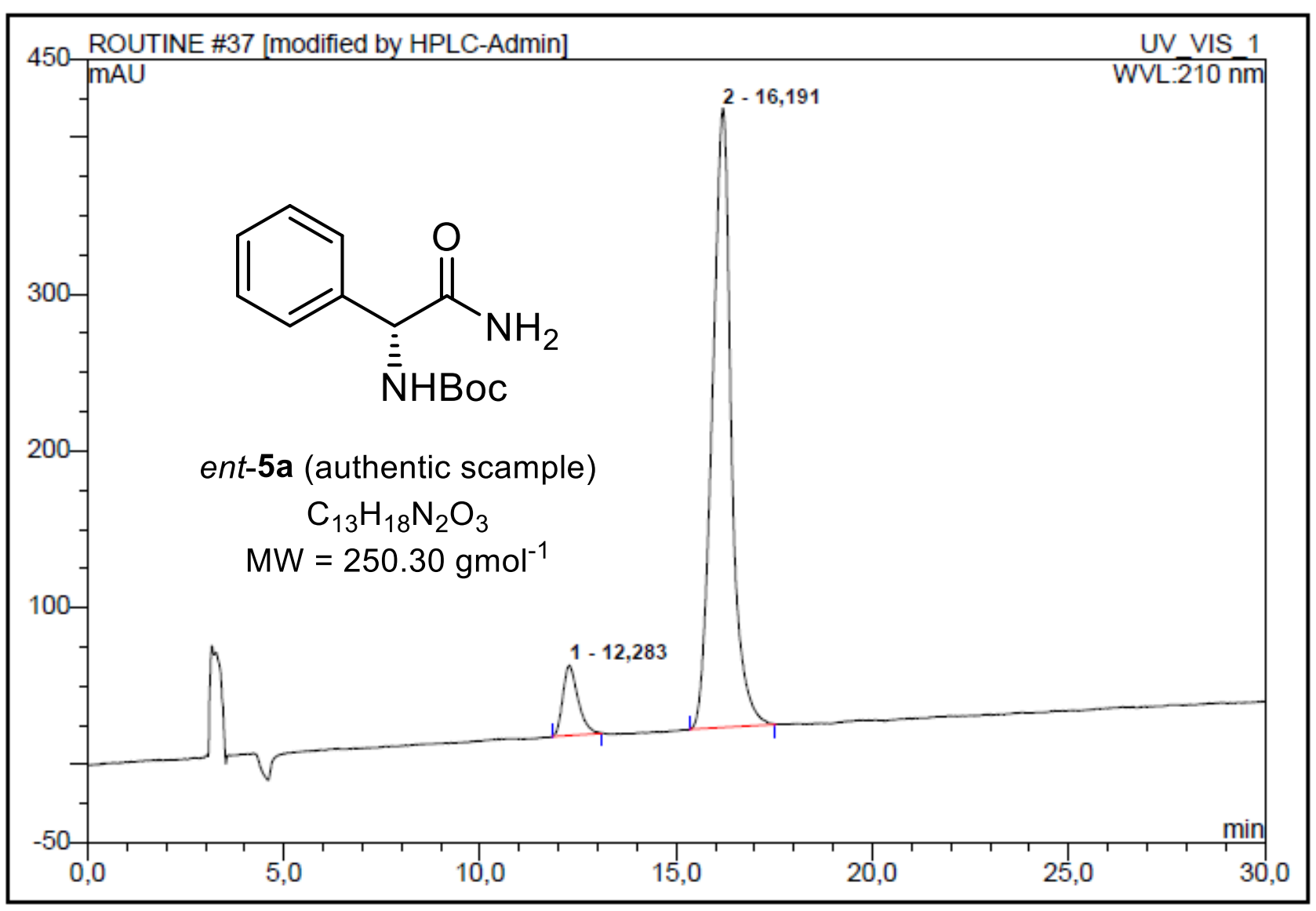

No.	Ret.Time min	Peak Name	Height mAU	Area mAU*min	Rel.Area %	Amount	Type
1	12,28	n.a.	44,672	20,034	8,30	n.a.	BMB*
2	16,19	n.a.	394,687	221,415	91,70	n.a.	BMB
Total:			439,359	241,449	100,00	0,000	

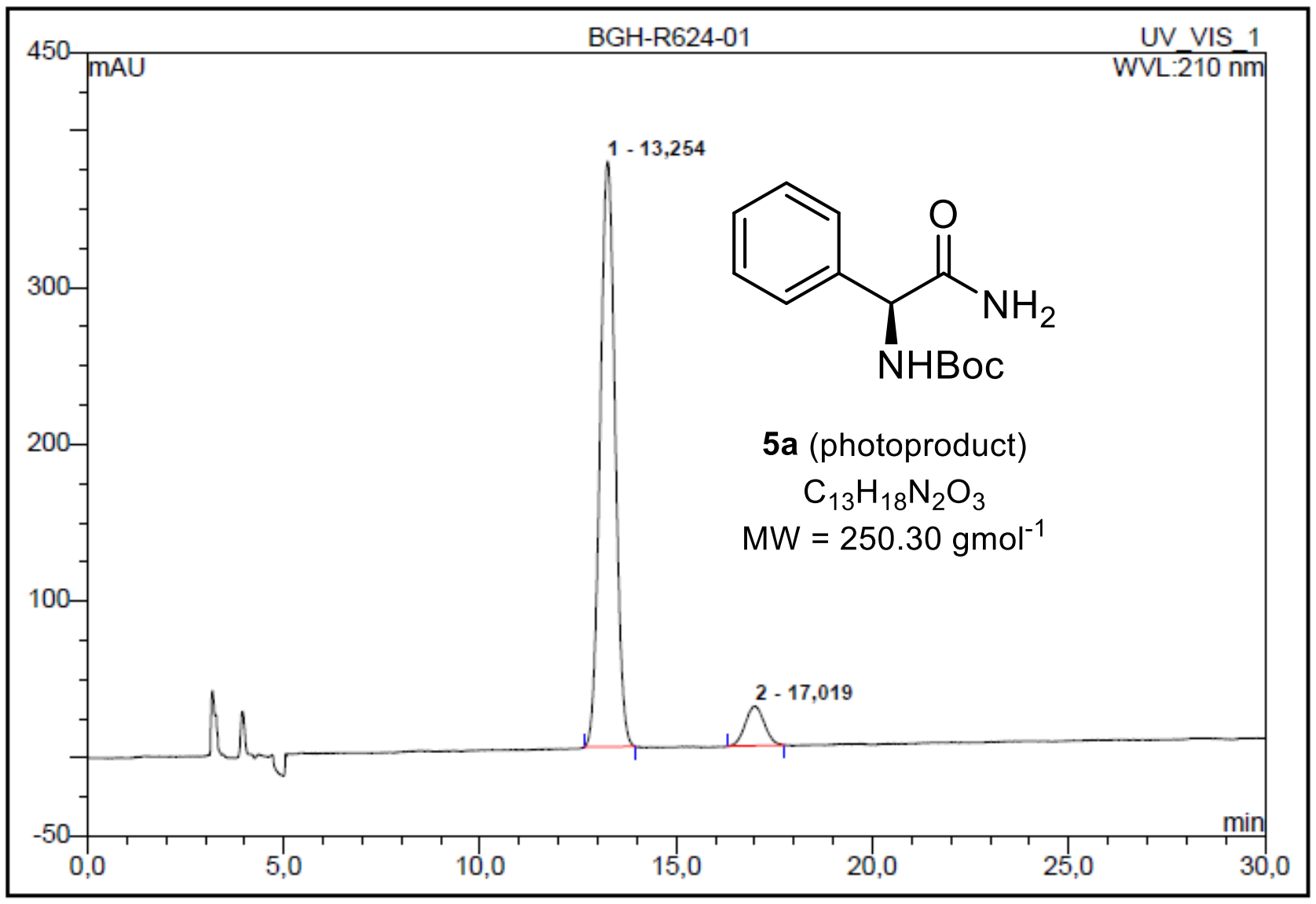

No.	Ret.Time min	Peak Name	Height mAU	Area mAU*min	Rel.Area %	Amount	Type
1	13,25	n.a.	373,242	164,486	92,03	n.a.	BMB
2	17,02	n.a.	25,228	14,239	7,97	n.a.	BMB*
Total:			398,470	178,725	100,00	0,000	

The absolute configuration of 2-phenyl-2-(phenylthio)acetamide (**5l**) was determined as (*R*) by comparison of the retention times on chiral HPLC. The (*R*)-2-phenyl-2-(phenylthio)acetamide (**5l**) was prepared from the enantioenriched methyl (*R*)-2-phenyl-2-(phenylthio)acetate[6] ((*R*)-**S-1**) following a modified GP C.

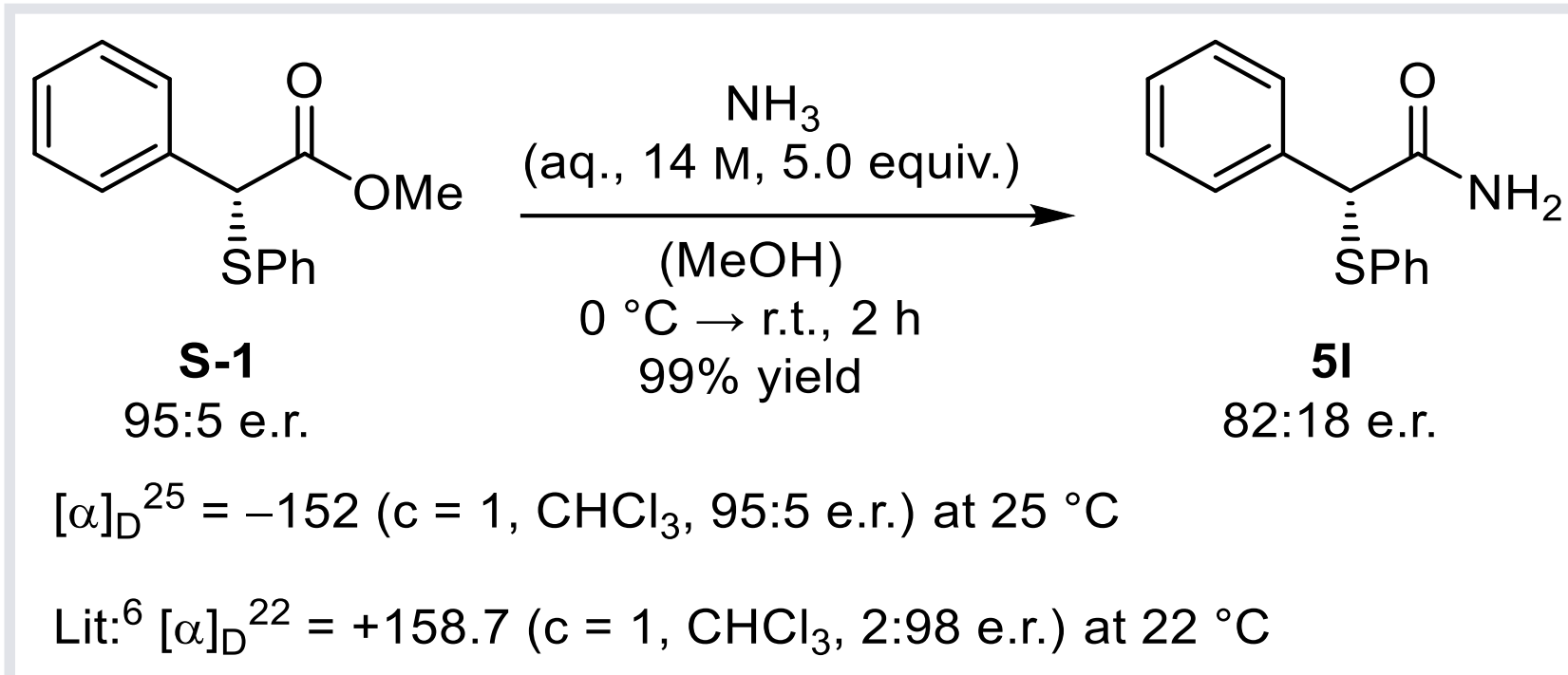

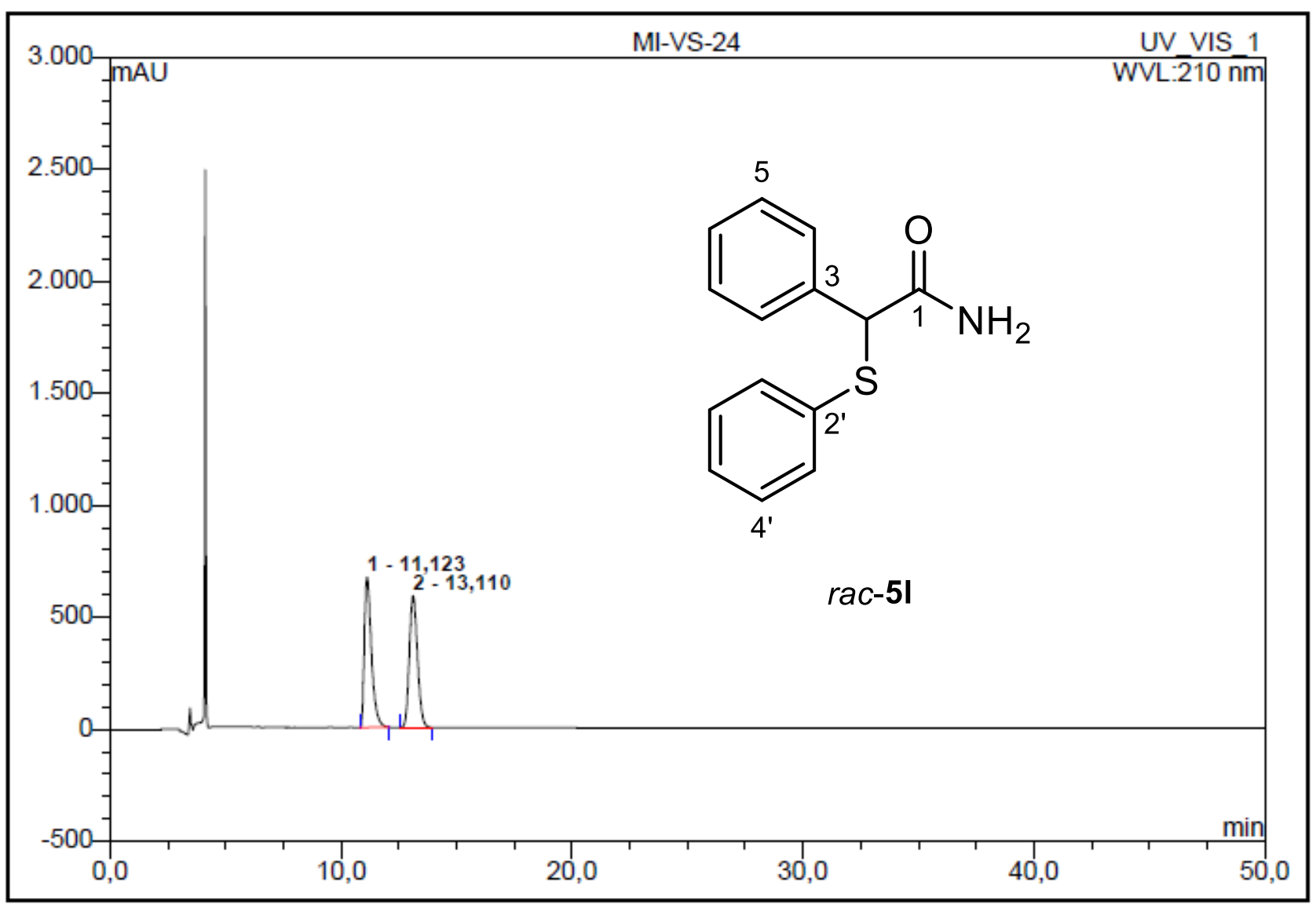

No.	Ret.Time min	Peak Name	Height mAU	Area mAU*min	Rel.Area %	Amount	Type
1	11,12	n.a.	669,866	240,973	49,92	n.a.	BMB
2	13,11	n.a.	587,008	241,770	50,08	n.a.	BMB
Total:			1256,874	482,743	100,00	0,000	

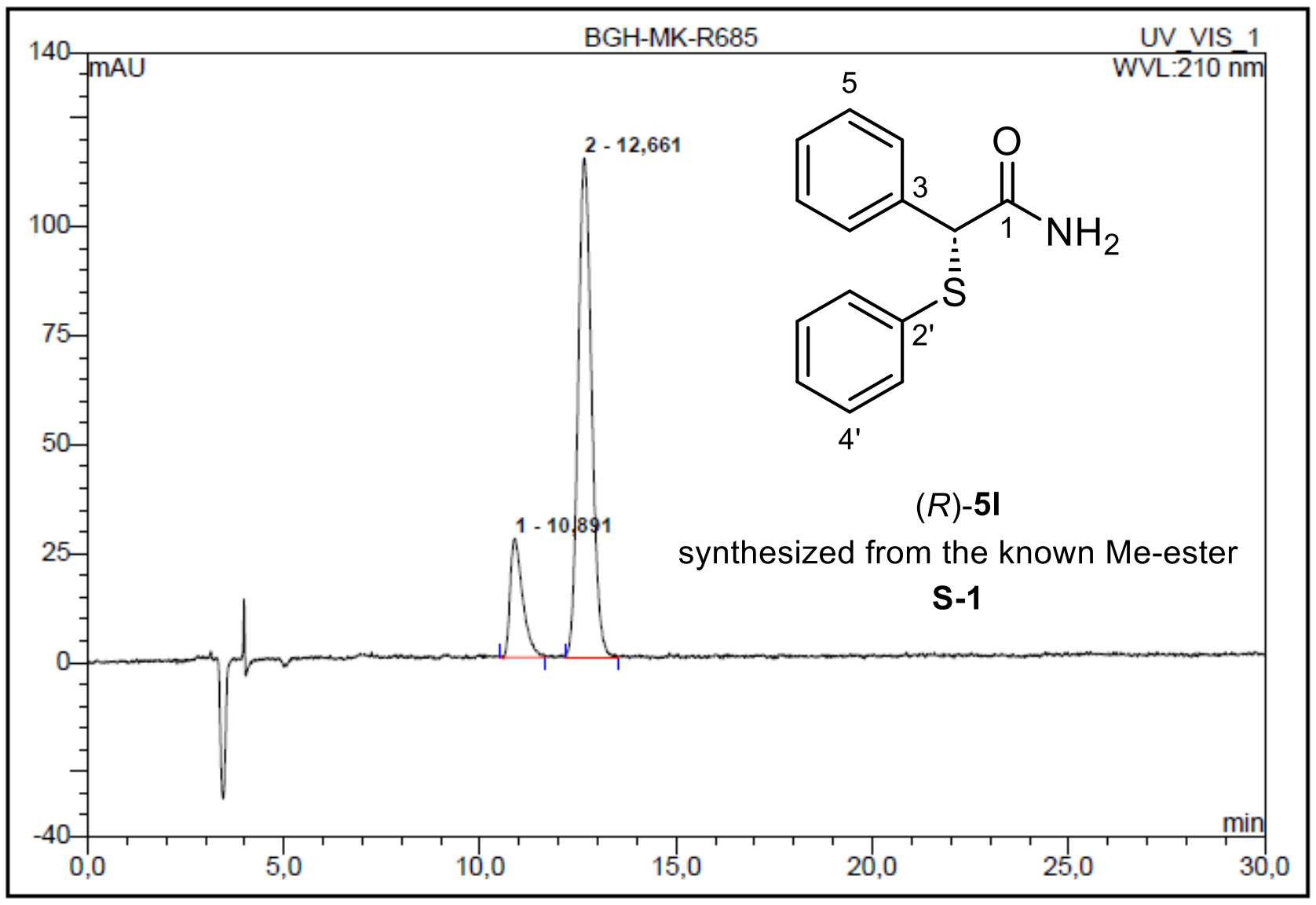

No.	Ret.Time min	Peak Name	Height mAU	Area mAU*min	Rel.Area %	Amount	Type
1	10,89	n.a.	27,270	10,140	18,18	n.a.	BMB*
2	12,66	n.a.	114,525	45,647	81,82	n.a.	BMB*
Total:			141,795	55,787	100,00	0,000	

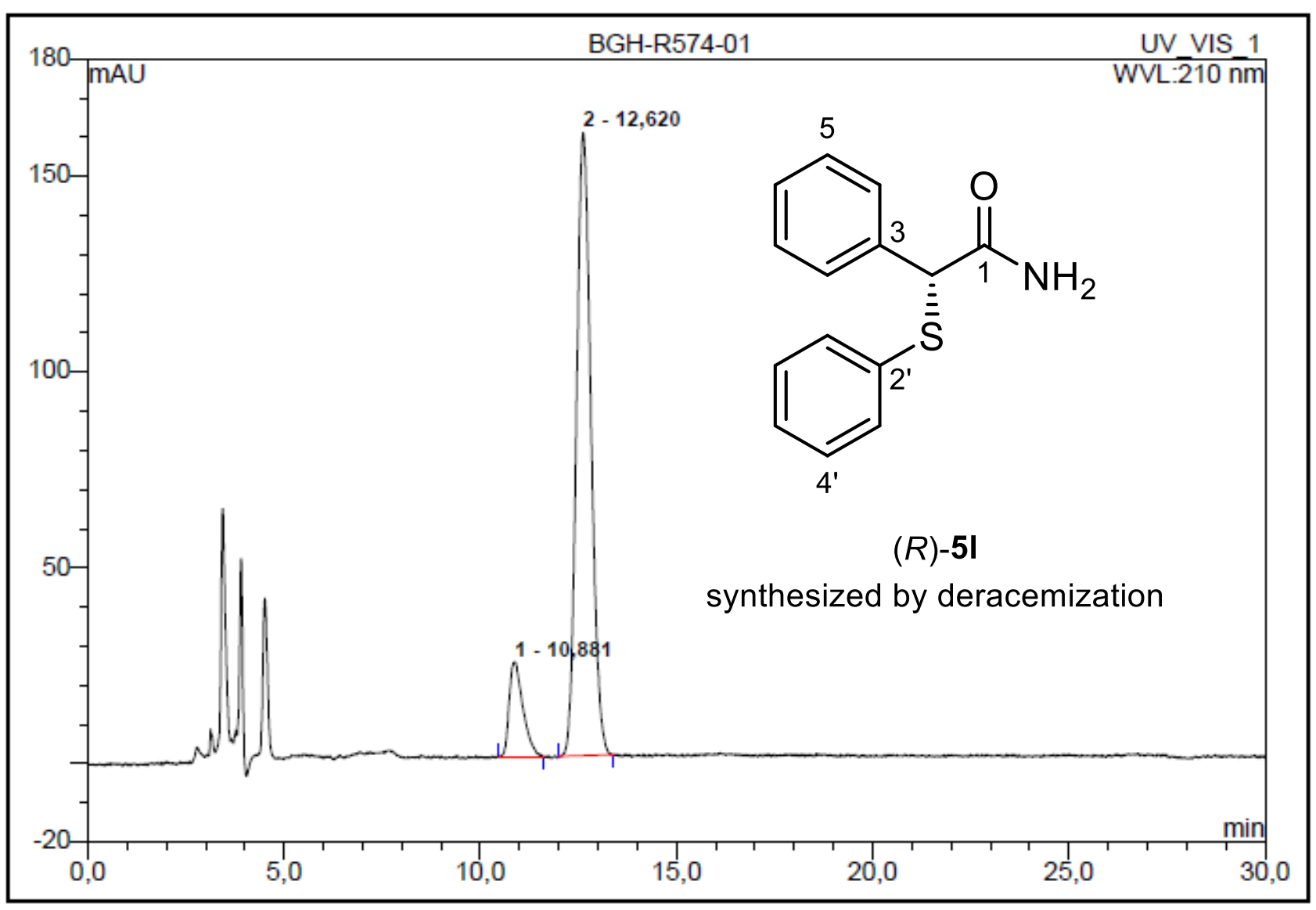

No.	Ret.Time min	Peak Name	Height mAU	Area mAU*min	Rel.Area %	Amount	Type
1	10,88	n.a.	24,098	9,812	12,61	n.a.	BMB*
2	12,62	n.a.	159,152	68,017	87,39	n.a.	BMB*
Total:			183,250	77,829	100,00	0,000	

Methyl 2-phenyl-2-(phenylthio)acetate (*rac*-**S-1**)

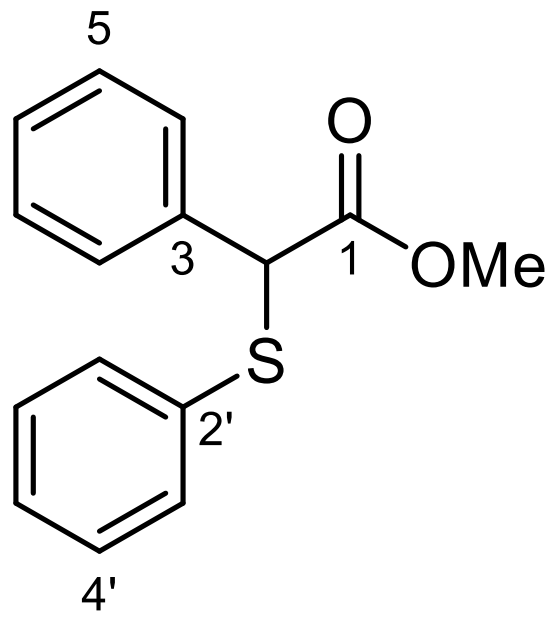

rac-**S-1**
$C_{15}H_{14}O_2S$
MW = 258.33 gmol^{-1}

A solution of 2-phenyl-2-(phenylthio)acetic acid (80.0 mg, 328 µmol, 1.00 equiv.) in methanol (1.0 mL) and CH_2Cl_2 (4.0 mL) was cooled to 0 °C. Subsequently, trimethylsilyldiazomethane (2.0 M in hexane) (327 µL, 655 µmol, 2.00 equiv.) was added dropwise. The resulting yellow solution was stirred at r.t. for 4 h. Acetic acid was added to quench the remaining trimethylsilyldiazomethane. The resulting colorless solution was evaporated to dryness to obtain the methyl 2-phenyl-2-(phenylthio)acetate (r*ac*-**S-1**) (71 mg, 275 µmol, 84%) as a colorless oil. The two enantiomers were then separated by chiral preparative HPLC (OD-H 250 × 20 mm, n-heptane/iPrOH = 90/10) to obtain (*R*)-**S-1** and (*S*)-**S-1**.

Specific Rotation: $[\alpha]_D^{25}$: −152 (*c* = 1.0, $CHCl_3$) [95:5 e.r.] for (*R*)-**S-1** (enantiomer 1).[6]

^{1}H-NMR (400 MHz, $CDCl_3$, 300 K): δ [ppm] = 7.40 – 7.20 (m, 10H, H4, H5, H6, H3', H4', H5'), 4.87 (s, 1H, H2), 3.63 (s, 3H, CH_3).

^{13}C-NMR (101 MHz, $CDCl_3$, 300 K): δ [ppm] = 171.0 (C1), 135.8 (C3), 133.8 (C2'), 132.8 (C_{aryl}), 129.1 (C_{aryl}), 128.8 (C_{aryl}), 128.7 (C_{aryl}), 128.5 (C_{aryl}), 128.2 (C_{aryl}), 56.5 (C2), 52.9 (CH_3).

IR (film) $\tilde{\nu}_{max}$/cm^{-1} = 3004 (w, CH_{arom}), 2953 (w, CH_{aliph}), 1717 (s, C=O), 1581 (m, C=C), 1455 (m, CH_{arom}), 1157 (m, C–O), 745 (s, C–S), 728 (s, C–S).

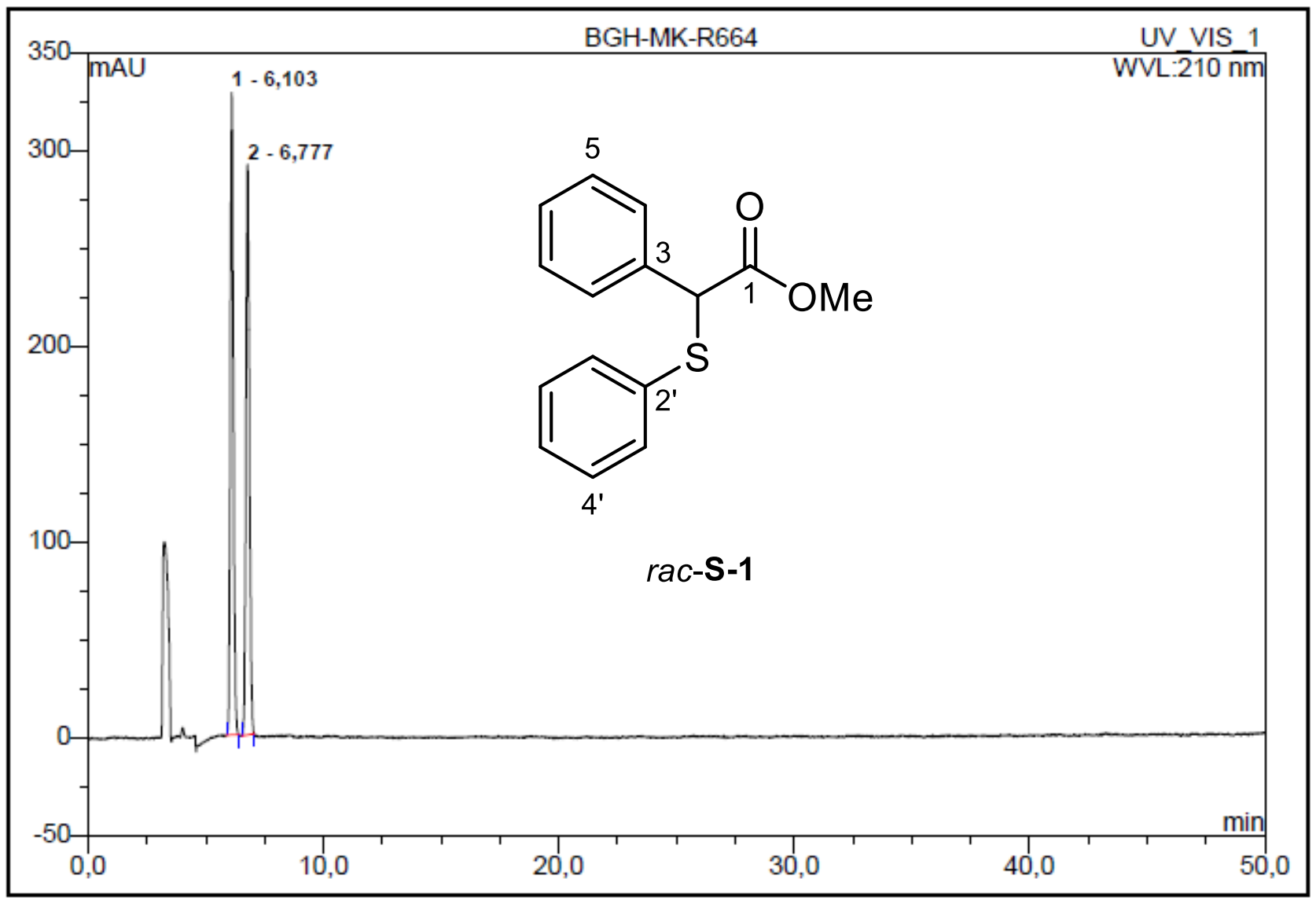

No.	Ret.Time min	Peak Name	Height mAU	Area mAU*min	Rel.Area %	Amount	Type
1	6,10	n.a.	328,015	51,169	50,12	n.a.	BMB
2	6,78	n.a.	291,262	50,921	49,88	n.a.	BMB
Total:			619,277	102,089	100,00	0,000	

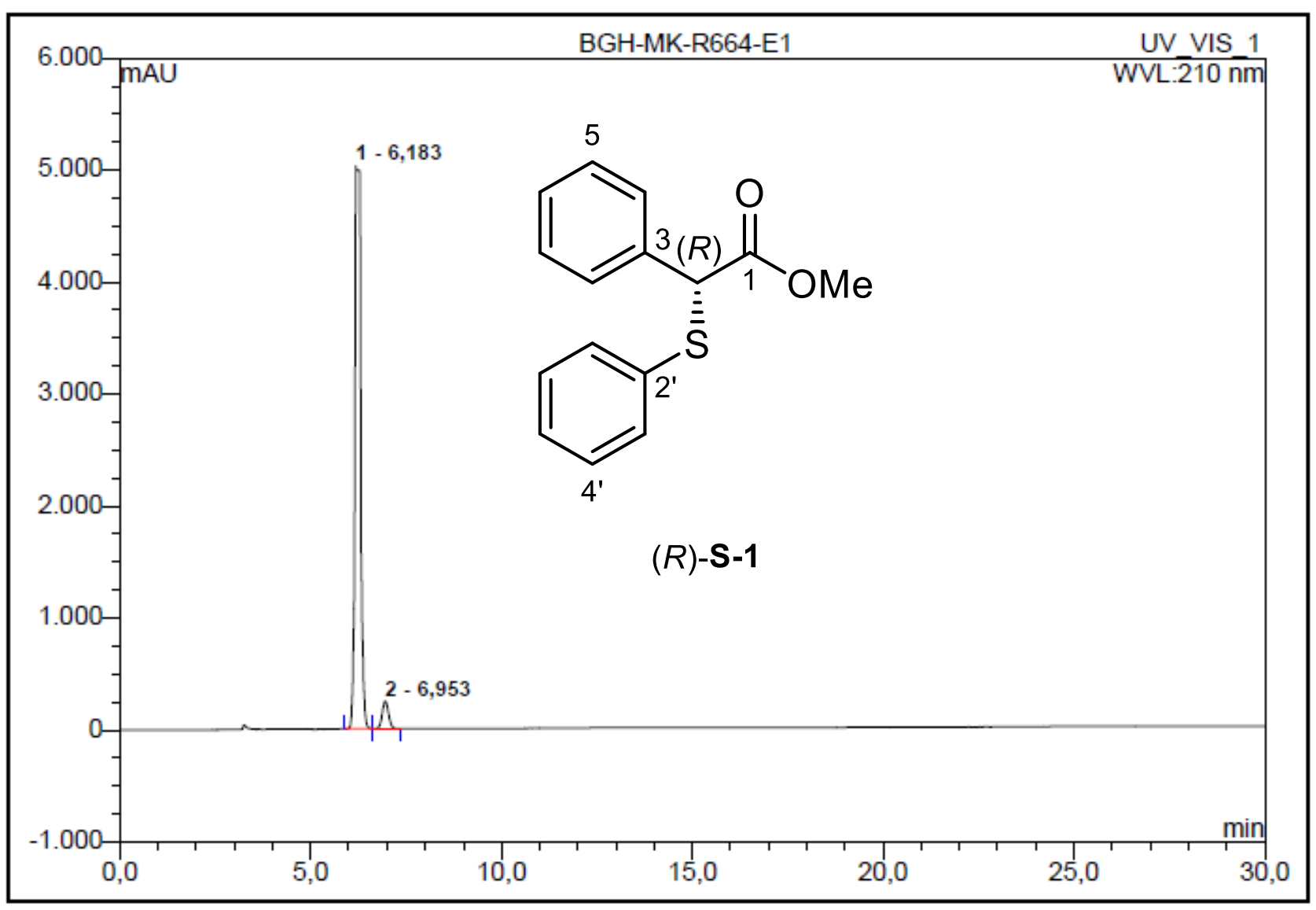

No.	Ret.Time min	Peak Name	Height mAU	Area mAU*min	Rel.Area %	Amount	Type
1	6,18	n.a.	5027,232	889,461	94,96	n.a.	BMB*
2	6,95	n.a.	246,164	47,177	5,04	n.a.	BMB*
Total:			5273,396	936,637	100,00	0,000	

S9. Synthesis and Characterization of Deracemization Substrates

2-Methoxy-2-phenylacetamide (*rac*-2a)

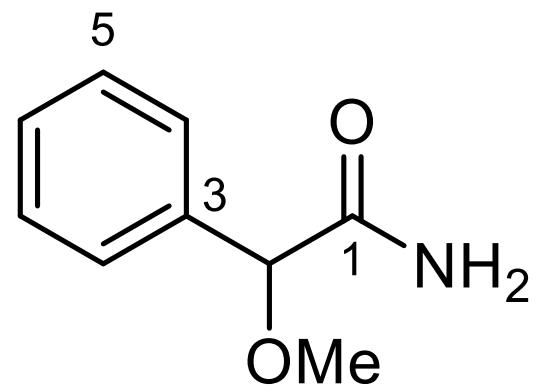

rac-**2a**
$C_9H_{11}NO_2$
MW = 165.19 gmol^{-1}

Following GP A, 2-methoxy-2-phenylacetamide (*rac*-**2a**) was obtained from 2-methoxy-2-phenylacetic acid (1.55 g, 9.33 mmol, 1.00 equiv.) as a white solid (1.37 g, 8.29 mmol, 89%).

TLC (70% EtOAc/hexanes): R_f = 0.27 [UV, $KMnO_4$].

^{1}H-NMR (400 MHz, $CDCl_3$, 300 K): δ [ppm] = 7.43 – 7.30 (m, 5H, H4, H5, H6), 6.66 (bs, 1H, NH_2^a), 5.95 (bs, 1H, NH_2^b), 4.61 (s, 1H, H2), 3.37 (s, 3H, OCH_3).

^{13}C-NMR (101 MHz, $CDCl_3$, 300 K): δ [ppm] = 173.6 (C1), 136.9 (C3), 128.8 (C5), 128.7 (C6), 127.2 (C4), 84.0 (C2), 57.4 (OCH_3).

The spectroscopic data matches the one reported in the literature.[7]

2-Methoxy-2-(p-tolyl)acetamide (*rac*-2b)

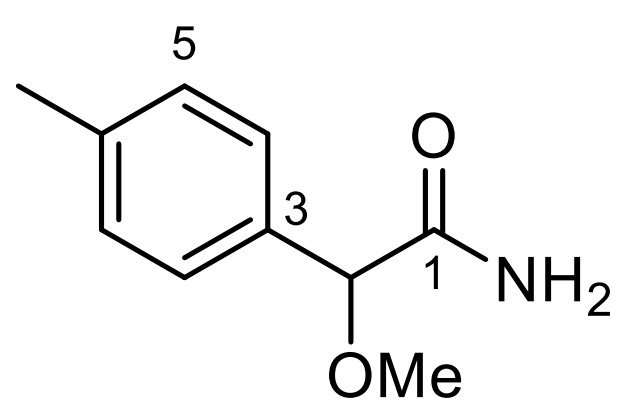

rac-**2b**
$C_{10}H_{13}NO_2$
MW = 179.22 gmol^{-1}

Following GP C, 2-methoxy-2-(*p*-tolyl)acetamide (*rac*-**2b**) was obtained from 2-hydroxy-2-(*p*-tolyl)acetic acid (300 mg, 1.81 mmol, 1.00 equiv.), as a white solid (152 mg, 848 μmol, 47% over two steps).

TLC (60% EtOAc/hexanes): R_f = 0.18 [UV, $KMnO_4$].

M.p.: 103-105 °C

^{1}H-NMR (400 MHz, $CDCl_3$, 300 K): δ [ppm] = 7.30 – 7.28 (m, 2H, H4), 7.19 – 7.17 (m, 2H, H5), 6.66 (bs, 1H, NH_2^a), 5.96 (bs, 1H, NH_2^b), 4.57 (s, 1H, H2), 3.35 (s, 3H, OCH_3), 2.34 (s, 3H, $C6CH_3$).

^{13}C-NMR (101 MHz, $CDCl_3$, 300 K): δ [ppm] = 173.8 (C1), 138.5 (C6), 133.9 (C3), 129.5 (C5), 127.2 (C4), 83.8 (C2), 57.2 (OCH_3), 21.3 (C6*C*H_3).

HRMS (ESI) *m/z* $[M+Na]^+$: calculated for $[C_{10}H_{13}NO_2Na]^+$: 202.0838; found: 202.0834.

IR (film) $\tilde{\nu}_{max}/cm^{-1}$ = 3379 (m, NH), 3168 (m, NH), 3051 (w, CH_{arom}), 2939 (w, CH_{aliph}), 2894 (w, CH_{aliph}), 2823 (w, CH_{aliph}), 1659 (s, C=O), 1198 (m, C–N), 1094 (s, C–O).

2-(4-Fluorophenyl)-2-methoxyacetamide (*rac*-2c)

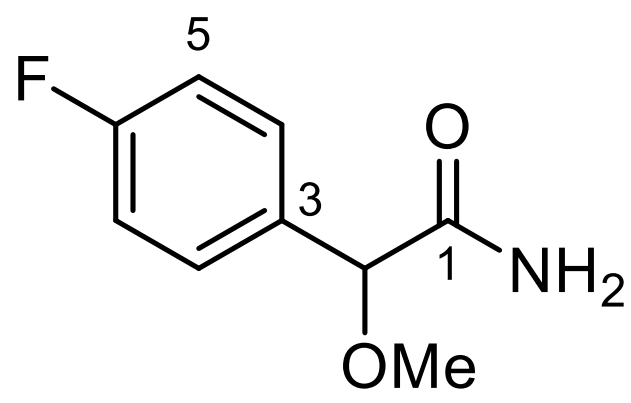

rac-**2c**
$C_9H_{10}FNO_2$
MW = 183.18 gmol^{-1}

Following GP C, 2-(4-fluorophenyl)-2-methoxyacetamide (*rac*-**2c**) was obtained from 2-(4-fluorophenyl)-2-hydroxyacetic acid (200 mg, 1.18 mmol, 1.00 equiv.) as a white solid (79.2 mg, 432 µmol, 37% over two steps).

TLC (60% EtOAc/hexanes): R_f = 0.16 [UV, $KMnO_4$].

M.p.: 115-116 °C

^{1}H-NMR (400 MHz, $CDCl_3$, 300 K): δ [ppm] = 7.41 – 7.36 (m, 2H, H4), 7.08 – 7.02 (m, 2H, H5), 6.66 (bs, 1H, NH_2^a), 5.96 (bs, 1H, NH_2^b), 4.59 (s, 1H, H2), 3.36 (s, 3H, OCH_3).

^{13}C-NMR (101 MHz, $CDCl_3$, 300 K): δ [ppm] = 173.4 (C1), 164.0 (d, $^1J_{CF}$ = 247 Hz, C6), 132.7 (d, $^4J_{CF}$ = 3.2 Hz, C3), 129.0 (d, $^3J_{CF}$ = 8.3 Hz, C4), 115.7 (d, $^2J_{CF}$ = 21.7 Hz, C5), 83.2 (C2), 57.4 (OCH_3).

^{19}F-NMR (376 MHz, $CDCl_3$, 300 K): δ [ppm] = −113.5 – −113.6 (m).

HRMS (ESI) *m/z* $[M+H]^+$: calculated for $[C_9H_{11}FNO_2]^+$: 184.0768; found: 184.0765.

IR (film) $\tilde{\nu}_{max}/cm^{-1}$ = 3459 (m, NH), 3165 (m, NH), 2995 (w, CH_{arom}), 2945 (w, CH_{aliph}), 2909 (w, CH_{aliph}), 2831 (w, CH_{aliph}), 1653 (m, C=O), 1605 (m, C=C), 1415 (m, CH_{arom}), 1228 (m, C–F), 1196 (m, C–N), 1087 (s, C–O).

2-(4-Chlorophenyl)-2-methoxyacetamide (*rac*-2d)

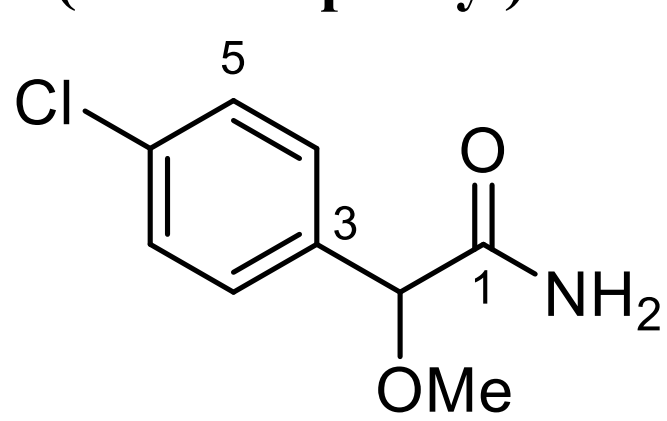

rac-**2d**
$C_9H_{10}ClNO_2$
MW = 199.63 gmol^{-1}

Following GP A, 2-(4-chlorophenyl)-2-methoxyacetamide (*rac*-**2d**) was obtained from 2-(4-chlorophenyl)-2-methoxyacetic acid (201 mg, 1.00 mmol, 1.00 equivas a white solid (190 mg, 951 µmol, 95%).

TLC (50% EtOAc/hexanes): R_f = 0.38 [UV, $KMnO_4$].

M.p.: 135-136 °C

^{1}H-NMR (400 MHz, $CDCl_3$, 300 K): δ [ppm] = 7.37 – 7.33 (m, 4H, H4, H5), 6.65 (bs, 1H, NH_2^a), 5.85 (bs, 1H, NH_2^b), 4.58 (s, 1H, H2), 3.37 (s, 3H, OCH_3).

^{13}C-NMR (101 MHz, $CDCl_3$, 300 K): δ [ppm] = 173.1 (C1), 135.4 (C3), 134.6 (C6), 129.0 (C5), 128.6 (C4), 83.2 (C2), 57.5 (OCH_3).

HRMS (ESI) *m/z* $[M+H]^+$: calculated for $[C_9H_{11}{}^{35}ClNO_2]^+$: 200.0473; found: 200.0472.

IR (film) $\tilde{\nu}_{max}/cm^{-1}$ = 3457 (m, NH), 3159 (m, NH), 2995 (w, CH_{arom}), 2935 (m, CH_{aliph}), 2904 (w, CH_{aliph}), 2827 (w, CH_{aliph}), 1653 (m, C=O), 1593 (m, C=C), 1487 (s, CH_{arom}), 1196 (m, C–N), 1083 (s, C–O), 978 (m, C–Cl).

2-(4-Bromophenyl)-2-methoxyacetamide (*rac*-**2e**)

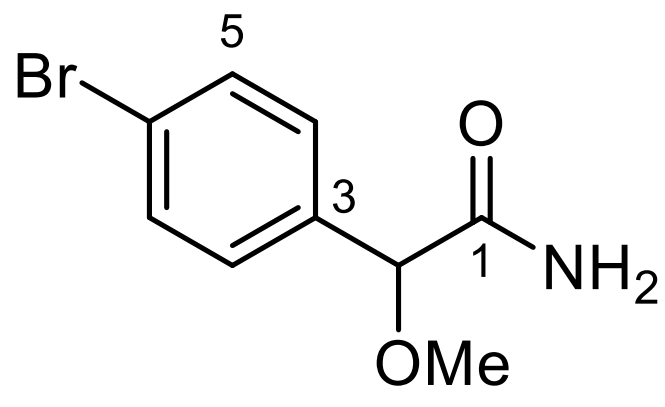

rac-**2e**
$C_9H_{10}BrNO_2$
MW = 244.09 $gmol^{-1}$

Following GP C, 2-(4-bromophenyl)-2-methoxyacetamide (*rac*-**2e**) was obtained from 2-(4-bromophenyl)-2-hydroxyacetic acid (620 mg, 2.68 mmol, 1.00 equiv.) as a white solid (304 mg, 1.25 mmol, 47% over two steps).

TLC (80% EtOAc/hexanes): R_f = 0.22 [UV, $KMnO_4$].

M.p.: 120-121 °C

^{1}H-NMR (400 MHz, $CDCl_3$, 300 K): δ [ppm] = 7.52 – 7.48 (m, 2H, H5), 7.31 – 7.28 (m, 2H, H4), 6.64 (bs, 1H, NH_2^a), 5.85 (bs, 1H, NH_2^b), 4.57 (s, 1H, H2), 3.37 (s, 3H, OCH_3).

^{13}C-NMR (101 MHz, $CDCl_3$, 300 K): δ [ppm] = 173.0 (C1), 135.9 (C3), 131.9 (C5), 128.9 (C4), 122.8 (C6), 83.3 (C2), 57.5 (OCH_3).

HRMS (ESI) *m/z* $[M+H]^+$: calculated for $[C_9H_{11}{}^{79}BrNO_2]^+$: 243.9968; found: 243.9964.

IR (film) $\tilde{\nu}_{max}/cm^{-1}$ = 3457 (m, NH), 3161 (m, NH), 2993 (w, CH_{arom}), 2933 (w, CH_{aliph}), 2898 (w, CH_{aliph}), 2825 (w, CH_{aliph}), 1653 (s, C=O), 1195 (m, C–N), 1087 (s, C–O), 1085 (s, C–Br).

2-Methoxy-2-(4-methoxyphenyl)acetamide (*rac*-**2f**)

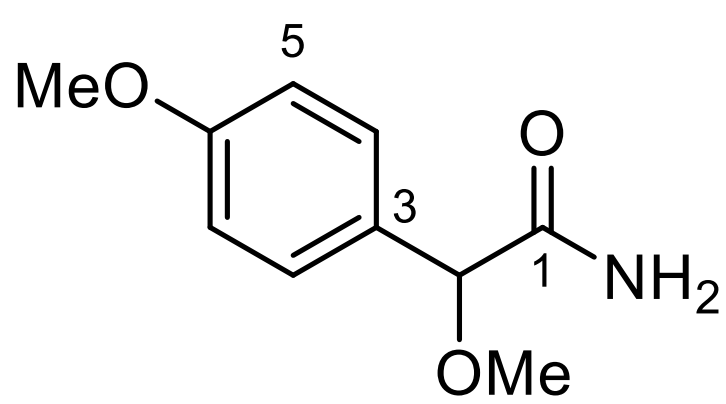

rac-**2f**
$C_{10}H_{13}NO_3$
MW = 195.22 $gmol^{-1}$

Following a modified GP C, 2-methoxy-2-(4-methoxyphenyl)acetamide (*rac*-**2f**) was obtained from 2-hydroxy-2-(4-hydroxyphenyl)acetic acid (150 mg, 892 µmol, 1.00 equiv.), Ag_2O (610 mg, 2.63 mmol, 2.95 equiv.) and MeI (3.04 g, 1.33 mL, 21.4 mmol, 24.0 equiv.) as a white solid (37.8 mg, 194 µmol, 22% over two steps).

TLC (67% EtOAc/hexanes): R_f = 0.13 [UV, $KMnO_4$].

M.p.: 172-173 °C

^{1}H-NMR (400 MHz, $CDCl_3$, 300 K): δ [ppm] = 7.34 – 7.30 (m, 2H, H4), 6.91 – 6.88 (m, 2H, H5), 6.66 (bs, 1H, NH_2^a), 5.87 (bs, 1H, NH_2^b), 4.56 (s, 1H, H2), 3.80 (s, 3H, C6O*C*H_3), 3.35 (s, 3H, C2O*C*H_3).

^{13}C-NMR (101 MHz, $CDCl_3$, 300 K): δ [ppm] = 173.9 (C1), 160.0 (C6), 129.0 (C3), 128.6 (C4), 114.2 (C5), 83.6 (C2), 57.1 (C2OCH$_3$), 55.4 (C6OCH$_3$).

HRMS (ESI) *m/z* $[M+H]^+$: calculated for $[C_{10}H_{14}NO_3]^+$: 196.0968; found: 196.0964.

IR (film) $\tilde{\nu}_{max}/cm^{-1}$ = 3379 (m, NH), 3148 (m, NH), 2952 (w, CH_{aliph}), 2924 (m, CH_{aliph}), 2853 (w, CH_{aliph}), 2825 (w, CH_{aliph}), 1657 (s, $C=O_{amide}$), 1608 (m, C=C), 1510 (m, CH_{arom}), 1176 (s, C–N), 1094 (s, C–O), 1025 (s, C–O).

2-Methoxy-2-(4-(trifluoromethyl)phenyl)acetamide (*rac*-2g)

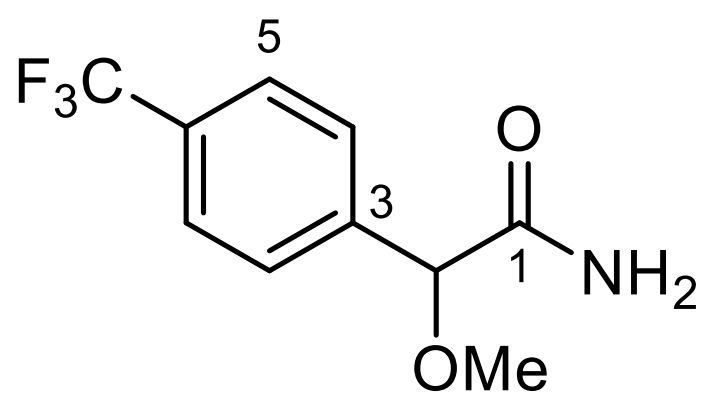

rac-**2g**
$C_{10}H_{10}F_3NO_2$
MW = 233.19 gmol^{-1}

Following GP C, 2-methoxy-2-(4-(trifluoromethyl)phenyl)acetamide (*rac*-**2g**) was obtained from 2-hydroxy-2-(4-(trifluoromethyl)phenyl)acetic acid (500 mg, 2.27 mmol, 1.00 equiv.) (185 mg, 793 μmol, 43% over two steps).

TLC (50% EtOAc/hexanes): R_f = 0.4 [UV, $KMnO_4$].

M.p.: 136-137 °C

^{1}H-NMR (400 MHz, $CDCl_3$, 300 K): δ [ppm] = 7.65 – 7.62 (m, 2H, H5), 7.57 – 7.54 (m, 2H, H4), 6.68 (bs, 1H, NH_2^a), 6.80 (bs, 1H, NH_2^b), 4.67 (s, 1H, H2), 3.41 (s, 3H, OCH_3).

^{13}C-NMR (101 MHz, $CDCl_3$, 300 K): δ [ppm] = 172.7 (C1), 140.8 (q, $^5J_{CF}$ = 0.8 Hz, C3), 130.9 (q, $^2J_{CF}$ = 32.4 Hz, C6), 127.5 (C4), 125.7 (q, $^3J_{CF}$ = 3.8 Hz, C5), 83.3 (C2), 57.8 (OCH_3).

^{19}F-NMR (376 MHz, $CDCl_3$, 300 K): δ [ppm] = −62.7.

HRMS (ESI) *m/z* $[M+H]^+$: calculated for $[C_{10}H_{11}F_3NO_2]^+$: 234.0736; found: 234.0734.

IR (film) $\tilde{\nu}_{max}/cm^{-1}$ = 3468 (m, NH), 3166 (m, NH), 3008 (w, CH_{arom}), 2939 (w, CH_{aliph}), 2833 (w, CH_{aliph}), 1696 (s, C=O), 1677 (s, C=C), 1327 (s, C–F), 1165 (s, C–N), 1103 (s, C–O).

2-(2-Fluorophenyl)-2-methoxyacetamide (*rac*-2h)

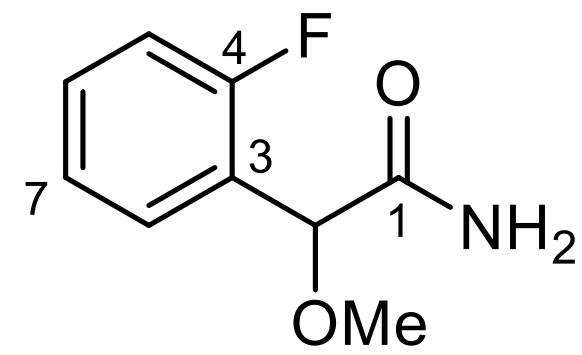

rac-**2h**
$C_9H_{10}FNO_2$
MW = 183.18 gmol^{-1}

Following GP A, 2-(2-fluorophenyl)-2-methoxyacetamide (*rac*-**2h**) was obtained from 2-(2-fluorophenyl)-2-methoxyacetic acid (200 mg, 1.09 mmol, 1.00 equiv.), as a white solid (178 mg, 971 µmol, 89%).

TLC (50% EtOAc/hexanes): R_f = 0.41 [UV, $KMnO_4$].

M.p.: 105-107 °C

^{1}H-NMR (400 MHz, $CDCl_3$, 300 K): δ [ppm] = 7.37 – 7.30 (m, 2H, H6, H8), 7.16 (*virt.* td, $^3J_{HH} \approx {}^3J_{HH}$ = 7.6 Hz, $^4J_{HH}$ = 1.2 Hz, 1H, H7), 7.09 (ddd, $^3J_{HF}$ = 9.6 Hz, $^3J_{HH}$ = 8.1 Hz, $^4J_{HH}$ = 1.2 Hz, 1H, H5), 6.77 (bs, 1H, $NH_2{}^a$), 6.07 (bs, 1H, $NH_2{}^b$), 4.96 (s, 1H, H2), 3.36 (s, 3H, OCH_3).

^{13}C-NMR (101 MHz, $CDCl_3$, 300 K): δ [ppm] = 172.9 (C1), 161.5 (d, $^1J_{CF}$ = 248 Hz, C4), 130.6 (d, $^3J_{CF}$ = 8.2 Hz, C8), 129.2 (d, $^3J_{CF}$ = 3.6 Hz, C6), 124.6 (d, $^4J_{CF}$ = 3.6 Hz, C7), 124.2 (d, $^2J_{CF}$ = 14.2 Hz, C3), 116.0 (d, $^2J_{CF}$ = 22.0 Hz, C5), 77.8 (C2), 57.4 (OCH_3).

^{19}F-NMR (376 MHz, $CDCl_3$, 300 K): δ [ppm] = −117.7 – −117.8 (m).

HRMS (ESI) *m/z* $[M+H]^+$: calculated for $[C_9H_{11}FNO_2]^+$: 184.0768; found: 184.0768.

IR (film) $\tilde{\nu}_{max}$/cm^{-1} = 3470 (m, NH), 3192 (m, NH), 3064 (w, CH_{arom}), 2943 (w, CH_{aliph}), 2917 (m, CH_{aliph}), 2840 (w, CH_{aliph}), 1677 (s, C=O), 1614 (m, C=C), 1485 (m, CH_{arom}), 1223 (m, C–F), 1111 (s, C–N), 1083 (s, C–O).

2-(2-Chlorophenyl)-2-methoxyacetamide (*rac*-2i)

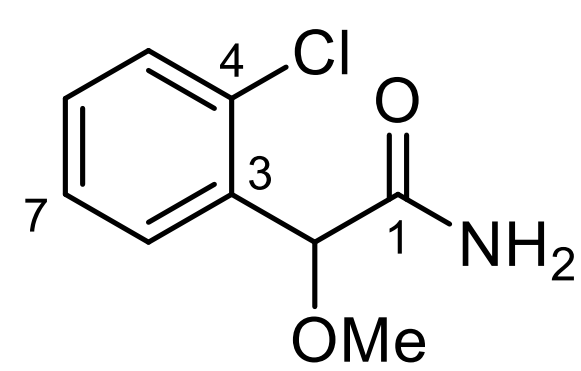

rac-**2i**
$C_9H_{10}ClNO_2$
MW = 199.63 gmol^{-1}

Following GP C, 2-(2-chlorophenyl)-2-methoxyacetamide (*rac*-**2i**) was obtained from 2-(2-chlorophenyl)-2-hydroxyacetic acid (500 mg, 2.68 mmol, 1.00 equiv.) as a white solid (128 mg, 641 µmol, 24%).

TLC (80% EtOAc/hexanes): R_f = 0.39 [UV, $KMnO_4$].

M.p.: 135-136 °C

^{1}H-NMR (400 MHz, $CDCl_3$, 300 K): δ [ppm] = 7.38 – 7.33 (m, 2H, H8, H5/H6/H7), 7.25 – 7.20 (m, 2H, H5/H6/H7), 6.69 (bs, 1H, $NH_2{}^a$), 6.15 (bs, 1H, $NH_2{}^b$), 5.13 (s, 1H, H2), 3.32 (s, 3H, OCH_3).

^{13}C-NMR (101 MHz, $CDCl_3$, 300 K): δ [ppm] = 172.8 (C1), 135.0 (C3/C4), 134.9 (C3/C4), 130.0 (C5/C6/C7), 130.0 (C5/C6/C7), 128.9 (C8), 127.4 (C5/C6/C7), 80.1 (C2), 57.5 (OCH_3).

HRMS (ESI) *m/z* $[M+H]^+$: calculated for $[C_9H_{11}{}^{35}ClNO_2]^+$: 200.0473; found: 200.0469.

IR (film) $\tilde{\nu}_{max}/cm^{-1}$ = 3465 (m, NH), 3172 (m, NH), 3060 (w, CH_{arom}), 2954 (m, CH_{aliph}), 2917 (m, CH_{aliph}), 2853 (w, CH_{aliph}), 2838 (w, CH_{aliph}), 1677 (s, C=O), 1597 (m, C=C), 1396 (m, CH_{arom}), 1202 (m, C–N), 1094 (s, C–O), 1047 (m, C–Cl).

2-(3-Chlorophenyl)-2-methoxyacetamide (*rac*-2j)

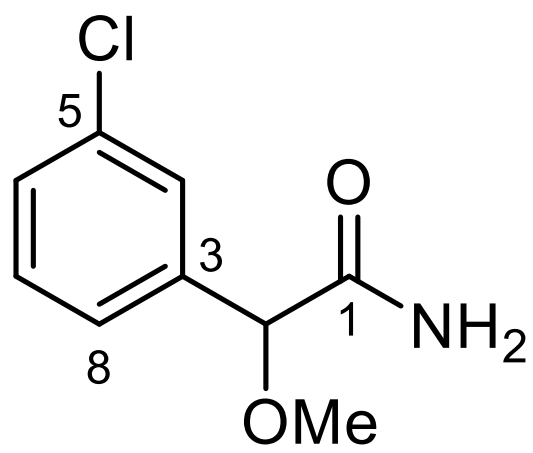

rac-**2j**
$C_9H_{10}ClNO_2$
MW = 199.63 gmol^{-1}

Following GP B, 2-(3-chlorophenyl)-2-methoxyacetamide (*rac*-**2j**) was obtained from 2-(3-chlorophenyl)-2-methoxyacetic acid (201 mg, 1.00 mmol, 1.00 equiv.) as a white solid (122 mg, 611 µmol, 61%).

TLC (50% EtOAc/hexanes): R_f = 0.37 [UV, $KMnO_4$].

M.p.: 130-131 °C

^{1}H-NMR (400 MHz, $CDCl_3$, 300 K): δ [ppm] = 7.42 – 7.41 (m, 1H, H4), 7.32 – 7.30 (m, 3H, H6, H7, H8), 6.66 (bs, 1H, $NH_2{}^a$), 5.85 (bs, 1H, $NH_2{}^b$), 4.58 (s, 1H, H2), 3.39 (s, 3H, OCH_3).

^{13}C-NMR (101 MHz, $CDCl_3$, 300 K): δ [ppm] = 172.9 (C1), 138.9 (C3), 134.8 (C5), 130.0 (C7), 128.9 (C6), 127.2 (C4), 125.5 (C8), 83.2 (C2), 57.6 (OCH_3).

HRMS (ESI) *m/z* $[M+H]^+$: calculated for $[C_9H_{11}{}^{35}ClNO_2]^+$: 200.0473; found: 200.0472.

IR (film) $\tilde{\nu}_{max}/cm^{-1}$ = 3459 (m, NH), 3168 (m, NH), 2997 (w, CH_{arom}), 2950 (m, CH_{aliph}), 2913 (m, CH_{aliph}), 2887 (w, CH_{aliph}), 2833 (w, CH_{aliph}), 1655 (s, C=O), 1595 (m, C=C), 1411 (s, CH_{arom}), 1191 (s, C–N), 1088 (s, C–O), 1087 (s, C–Cl).

2-(3,4-Dichlorophenyl)-2-methoxyacetamide (*rac*-2k)

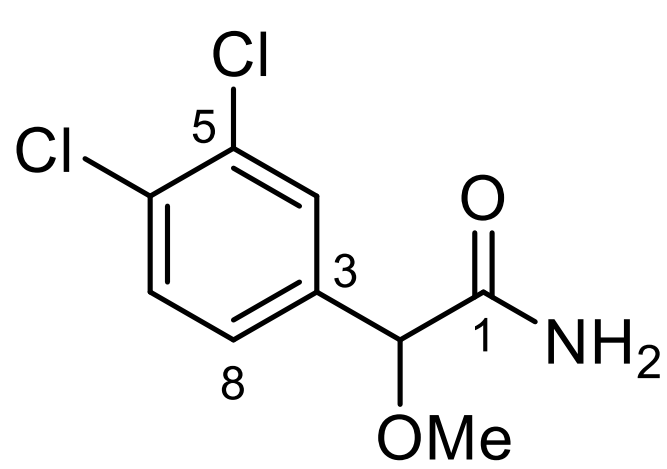

rac-**2k**
$C_9H_9Cl_2NO_2$
MW = 234.08 gmol^{-1}

Following GP A, 2-(3,4-dichlorophenyl)-2-methoxyacetamide (*rac*-**2k**) was obtained from 2-(3,4-dichlorophenyl)-2-methoxyacetic acid (300 mg, 1.28 mmol, 1.00 equiv.) as a white solid (205 mg, 876 µmol, 69%).

TLC (50% EtOAc/hexanes): R_f = 0.4 [UV, $KMnO_4$].

M.p.: 152-157 °C

^{1}H-NMR (400 MHz, $CDCl_3$, 300 K): δ [ppm] = 7.52 (d, ^4J = 2.1 Hz, 1H, H4), 7.44 (d, ^3J = 8.3 Hz, 1H, H7), 7.28 – 7.26 (m, 1H, H8), 6.65 (bs, 1H, $NH_2{}^a$), 5.91 (bs, 1H, $NH_2{}^b$), 4.56 (s, 1H, H2), 3.39 (s, 3H, OCH_3).

^{13}C-NMR (101 MHz, $CDCl_3$, 300 K): δ [ppm] = 172.6 (C1), 137.1 (C3), 133.0 (C5/C6), 132.8 (C5/C6), 130.7 (C7), 129.0 (C4), 126.6 (C8), 82.6 (C2), 57.8 (OCH_3).

HRMS (ESI) *m/z* $[M+H]^+$: calculated for $[C_9H_{10}{}^{35}Cl_2NO_2]^+$: 234.0083; found: 234.0082.

IR (film) $\tilde{\nu}_{max}/cm^{-1}$ = 3457 (m, NH), 3178 (m, NH), 2995 (w, CH_{arom}), 2950 (m, CH_{aliph}), 2913 (m, CH_{aliph}), 2853 (w, CH_{aliph}), 2831 (w, CH_{aliph}), 1653 (s, C=O), 1560 (m, C=C), 1465 (s, CH_{arom}), 1191 (m, C–N), 1085 (s, C–O), 1034 (m, C–Cl), 978 (m, C–Cl).

2-(3,4-Dimethoxyphenyl)-2-methoxyacetamide (*rac*-2l)

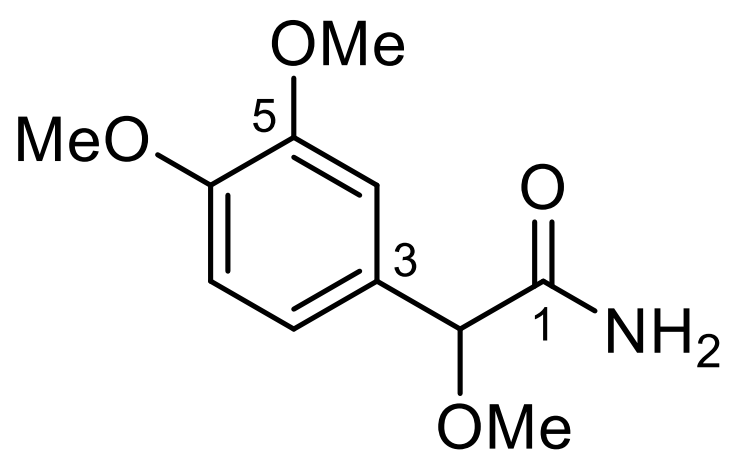

rac-2l
$C_{11}H_{15}NO_4$
MW = 225.24 $gmol^{-1}$

Following a modified GP C, 2-(3,4-dimethoxyphenyl)-2-methoxyacetamide (*rac*-**2l**) was obtained from 2-(3-methoxy-4-hydroxyphenyl)-2-hydroxyacetic acid (400 mg, 2.02 mmol, 1.00 equiv.), Ag_2O (1.38 g, 5.95 mmol, 2.95 equiv.) and MeI (6.88 g, 3.02 mL, 48.4 mmol, 24.0 equiv.) as a white solid (87.0 mg, 386 µmol, 19% over two steps).

TLC (80% EtOAc/hexanes): R_f = 0.12 [UV, $KMnO_4$].

M.p.: 130-131 °C

^{1}H-NMR (400 MHz, $CDCl_3$, 300 K): δ [ppm] = 6.97 (dd, ^3J = 8.2 Hz, ^4J = 2.0 Hz, 1H, H8), 6.90 (d, ^4J = 2.0 Hz, 1H, H4), 6.86 (d, ^3J = 8.2 Hz, 1H, H7), 6.64 (bs, 1H, $NH_2{}^a$), 5.80 (bs, 1H, $NH_2{}^b$), 4.55 (s, 1H, H2), 3.88 – 3.87 (m, 6H, C5OCH_3, C6OCH_3), 3.36 (s, 3H, C2OCH_3).

^{13}C-NMR (101 MHz, $CDCl_3$, 300 K): δ [ppm] = 173.7 (C1), 149.5 (*C*5OCH_3/ *C*6OCH_3), 149.4 (*C*5OCH_3/ *C*6OCH_3), 129.3 (C3), 120.1 (C8), 111.3 (C7), 110.0 (C4), 83.8 (C2), 57.2 (C2O*C*H_3), 56.1 (C5O*C*H_3/ C6O*C*H_3), 56.1 (C5O*C*H_3/ C6O*C*H_3).

HRMS (ESI) *m/z* $[M+H]^+$: calculated for $[C_{11}H_{16}NO_4]^+$: 226.1074; found: 226.1070.

IR (film) $\tilde{\nu}_{max}/cm^{-1}$ = 3401 (m, NH), 3163 (m, NH), 2952 (w, CH_{aliph}), 2922 (m, CH_{aliph}), 2853 (w, CH_{aliph}), 2837 (w, CH_{aliph}), 1679 (s, C=O), 1511 (m, CH_{arom}), 1230 (s, C–N), 1133 (s, C–O), 1092 (s, C–O), 1019 (s, C–O).

2-Methoxy-2-(thiophen-3-yl)acetamide (*rac*-2m)

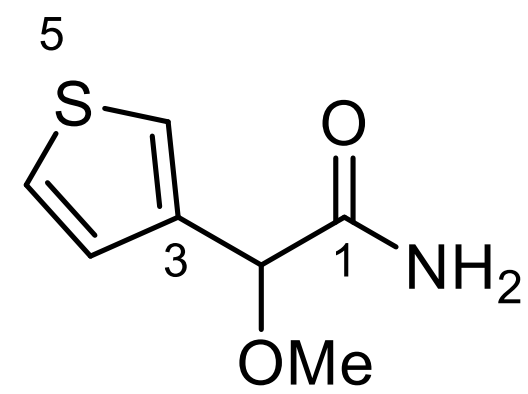

rac-**2m**
$C_7H_9NO_2S$
MW = 171.21 gmol^{-1}

Following GP B, 2-methoxy-2-(thiophen-3-yl)acetamide (*rac*-**2m**) was obtained from thiophene-3-carbaldehyde (130 mg, 107 µL, 1.16 mmol, 1.00 equiv.) and MeOH (0.38 M) as a white solid (148 mg, 864 µmol, 75% over two steps).

TLC (70% EtOAc/hexanes): R_f = 0.40 [UV, $KMnO_4$].

M.p.: 138-139 °C

^{1}H-NMR (400 MHz, $CDCl_3$, 300 K): δ [ppm] = 7.34 – 7.31 (m, 2H, H6, H4), 7.10 (dd, ^3J = 4.9 Hz, ^4J = 1.3 Hz, 1H, H7), 6.65 (bs, 1H, $NH_2{}^a$), 6.03 (bs, 1H, $NH_2{}^b$), 4.72 (s, 1H, H2), 3.40 (s, 3H, OCH_3).

^{13}C-NMR (101 MHz, $CDCl_3$, 300 K): δ [ppm] = 173.3 (C1), 137.7 (C3), 126.5 (C6), 126.0 (C7), 123.7 (C4), 80.3 (C2), 57.6 (OCH_3).

HRMS (ESI) *m/z* $[M+H]^+$: calculated for $[C_7H_{10}NO_2S]^+$: 172.0427; found: 172.0424.

IR (film) $\tilde{\nu}_{max}/cm^{-1}$ = 3394 (m, NH), 315 5(m, NH), 3110 (w, CH_{arom}), 3002 (w, CH_{arom}), 2933 (m, CH_{aliph}), 2887 (w, CH_{aliph}), 2820 (w, CH_{aliph}), 1659 (s, C=O), 1187 (m, C–N), 1092 (s, C–O), 773 (s, C–S).

2-Methoxy-2-(thiophen-2-yl)acetamide (*rac*-2n)

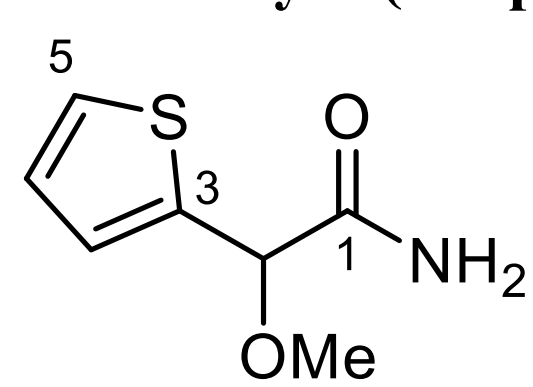

rac-**2n**
$C_7H_9NO_2S$
MW = 171.21 gmol^{-1}

Following GP B, 2-methoxy-2-(thiophen-2-yl)acetamide (*rac*-**2n**) was obtained from thiophene-2-carbaldehyde (130 mg, 107 µL, 1.16 mmol, 1.00 equiv.) and MeOH (0.38 M) as a white solid (142 mg, 829 µmol, 72% over two steps).

TLC (50% EtOAc/hexanes): R_f = 0.33 [UV, $KMnO_4$].

M.p.: 143-144 °C

^{1}H-NMR (400 MHz, $CDCl_3$, 300 K): δ [ppm] = 7.31 (dd, ^3J = 5.1 Hz, ^4J = 1.1 Hz, 1H, H5), 7.14 (*virt.* dd, ^3J = 3.6 Hz, $^4J \approx {}^4J$ = 1.0 Hz, 1H, H7), 7.00 (dd, ^3J = 5.1 Hz, ^3J = 3.6 Hz, 1H, H6), 6.66 (bs, 1H, $NH_2{}^a$), 5.84 (bs, 1H, $NH_2{}^b$), 4.88 (d, ^4J = 1.1, 1H, H2), 3.43 (s, 3H, CH_3).

^{13}C-NMR (101 MHz, $CDCl_3$, 300 K): δ [ppm] = 172.5 (C1), 139.5 (C3), 127.2 (C7), 126.7 (C6), 126.3 (C4), 79.9 (C2), 57.6 (CH_3).

HRMS (ESI) *m/z* $[M +H]^+$: calculated for $[C_7H_{10}NO_2S]^+$: 172.0427; found: 172.0419.

IR (film) $\tilde{\nu}_{max}$/cm^{-1} = 3459 (m, NH), 3157 (m, NH), 3103 (w, CH_{arom}), 3087 (w, CH_{arom}), 2956 (w, CH_{aliph}), 1659 (s, C=O), 1188 (m, C–N), 1085 (s, C–O), 785 (s, C–S).

2-(Furan-3-yl)-2-methoxyacetamide (*rac*-2o)

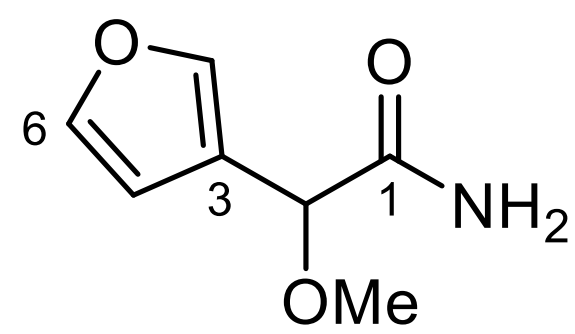

rac-**2o**
$C_7H_9NO_3$
MW = 155.15 gmol^{-1}

Following GP B, 2-(furan-3-yl)-2methoxyacetamide (*rac*-**2o**) was obtained from furan-3-carbaldehyde (150 mg, 134 µL, 1.56 mmol, 1.00 equiv.) and MeOH (0.38 M) as a white solid (167 mg, 1.08 mmol, 69% over two steps).

TLC (50% EtOAc/hexanes): R_f = 0.25 [UV, $KMnO_4$].

M.p.: 117-118 °C

^{1}H-NMR (400 MHz, $CDCl_3$, 300 K): δ [ppm] = 7.51 (m, 1H, H4), 7.40 (*virt.* t, $^3J \approx {}^4J$ = 1.7 Hz, 1H, H6), 6.65 (bs, 1H, NH_2^a) 6.40 (dd, ^3J = 1.9 Hz, ^4J = 0.9 Hz, 1H, H7), 6.13 (bs, 1H, NH_2^b), 4.60 (s, 1H, H2), 3.09 (s, 3H, CH_3).

^{13}C-NMR (101 MHz, $CDCl_3$, 300 K): δ [ppm] = 173.4 (C1), 143.8 (C6), 141.4 (C4), 121.6 (C3), 108.8 (C7), 76.8 (C2), 57.3 (CH_3).

HRMS (ESI) *m/z* $[M+H]^+$: calculated for $[C_7H_{10}NO_3]^+$: 156.0655; found: 156.0648.

IR (film) $\tilde{\nu}_{max}$/cm^{-1} = 3465 (m, NH), 3152 (w, NH), 3001 (w, CH_{arom}), 2948 (w, CH_{aliph}), 2910 (w, CH_{aliph}), 2836 (w, CH_{aliph}), 2836 (w, CH_{aliph}), 1655 (s, C=O), 1594 (m, C=C), 1414 (m, CH_{arom}), 1190 (m, C–N), 1092 (s, C–O), 1016 (s, C–O).

2-(Furan-2-yl)-2-methoxyacetamide (*rac*-2p)

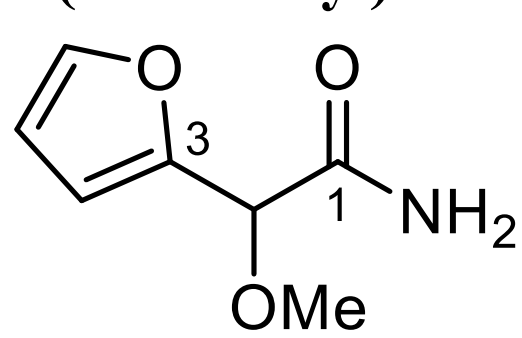

rac-**2p**
$C_7H_9NO_3$
MW = 155.15 gmol^{-1}

Following GP B, 2-(furan-2-yl)-2methoxyacetamide (*rac*-**2p**) was obtained from furfural (150 mg, 129 µL, 1.56 mmol, 1.00 equiv.) and MeOH (0.38 M) as a white solid (175 mg, 1.13 mmol, 72% over two steps).

TLC (50% EtOAc/hexanes): R_f = 0.25 [UV, $KMnO_4$].

M.p.: 120-121 °C

^{1}H-NMR (400 MHz, $CDCl_3$, 300 K): δ [ppm] = 7.43 (dd, ^3J = 1.8 Hz, ^4J = 0.8 Hz, 1H, H5), 6.74 (bs, 1H, NH_2^a), 6.47 (dd, ^3J = 3.3 Hz, ^4J = 0.8 Hz, 1H, H7), 6.37 (dd, ^3J = 3.3 Hz, ^3J = 1.8 Hz, 1H, H6), 5.29 (bs, 1H, NH_2^b), 4.70 (s, 1H, H2), 3.37 (s, 3H, CH_3).

^{13}C-NMR (101 MHz, $CDCl_3$, 300 K): δ [ppm] = 171.3 (C1), 149.3 (C3), 143.5 (C7), 111.0 (C7), 110.6 (C6), 77.2 (C2), 57.2 (CH_3).

HRMS (ESI) *m/z* $[M+H]^+$: calculated for $[C_7H_{10}NO_3]^+$: 156.0655; found: 156.0648.

IR (film) $\tilde{\nu}_{max}/cm^{-1}$ = 3383 (m, NH), 3153 (m, NH), 3005 (w, CH_{arom}), 2936 (w, CH_{aliph}), 2997 (w, CH_{aliph}), 2897 (w, CH_{aliph}), 2824 (w, CH_{aliph}), 1662 (s, C=O), 1418 (m, CH_{arom}), 1185 (m, C–N), 1094 (s, C–O).

2-Methoxy-3-phenylpropanamide (*rac*-2q)

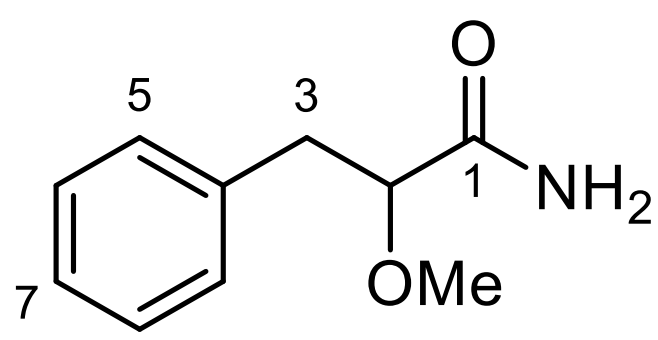

rac-**2q**
$C_{10}H_{13}NO_2$
MW = 179.22 gmol^{-1}

Following GP B, 2-methoxy-3-phenylpropanamide (*rac*-**2q**) was obtained from 2-hydroxy-3-phenylpropanoic acid (500 mg, 3.01 mmol, 1.00 equiv.) as a white solid (154 mg, 859 µmol, 29% over two steps).

TLC (50% EtOAc/hexanes): R_f = 0.41 [UV, $KMnO_4$].

M.p.: 94-95 °C

^{1}H-NMR (400 MHz, $CDCl_3$, 300 K): δ [ppm] = 7.33 – 7.22 (m, 5H, H5, H6, H7), 6.43 (bs, 1H, NH_2^a), 5.91 (bs, 1H, NH_2^b), 3.88 (dd, ^3J = 7.6 Hz, 3.8 Hz, 1H, H2), 3.36 (s, 3H, CH_3), 3.17 (dd, ^2J = 14.2 Hz, ^3J = 3.8 Hz, 1H, H3^a), 2.95 (dd, ^2J = 14.2 Hz, ^3J = 7.6 Hz, 1H, H3^b), 0.95 (t, ^3J = 7.4 Hz, 3H, H4’).

^{13}C-NMR (101 MHz, $CDCl_3$, 300 K): δ [ppm] = 175.1 (C1), 137.3 (C4), 129.7 (C6), 128.4 (C5), 126.7 (C7), 83.3 (C2), 58.8 (CH_3), 38.7 (C3).

IR (film) $\tilde{\nu}_{max}/cm^{-1}$ = 3392 (m, NH), 3184 (w, NH), 3030 (w, CH_{arom}), 2947 (m, CH_{aliph}), 2831 (w, CH_{aliph}), 1655 (s, C=O), 1109 (s, C–N), 1080 (m, C–O).

2-Cyclopropyl-2-methoxyacetamide (*rac*-2r)

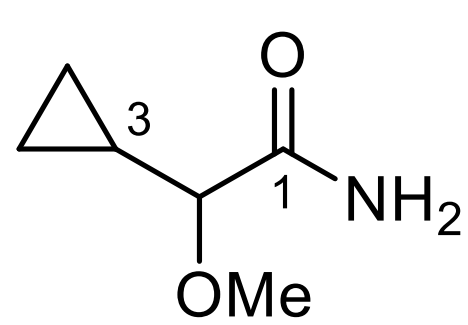

rac-**2r**
$C_6H_{11}NO_2$
MW = 129.16 gmol^{-1}

Following GP C, 2-cyclopropyl-2-methoxyacetamide (*rac*-**2r**) was obtained from 2-cyclopropyl-2-hydroxyacetic acid (232 mg, 2.00 mmol, 1.00 equiv.) as a white solid (124 mg, 960 µmol, 48% over two steps).

TLC (50% EtOAc/hexanes): R_f = 0.30 [UV, $KMnO_4$].

M.p.: 81-82 °C

^{1}H-NMR (400 MHz, $CDCl_3$, 300 K): δ [ppm] = 6.40 (bs, 1H, NH_2^a), 5.91 (bs, 1H, NH_2^b), 3.42 (s, 3H, CH_3), 3.19 (d, ^3J = 7.2 Hz, 1H, H2), 1.10 – 1.02 (m, 1H, H3), 0.60 – 0.50 (m, 3H, H4^a, H5^a, H5^a), 0.47 – 0.39 (m, 1H, H5^b).

^{13}**C-NMR** (101 MHz, $CDCl_3$, 300 K): δ [ppm] = 175.2 (C1), 84.8 (C2), 58.5 (CH_3), 13.7 (C3), 3.04 (C4/C5), 1.66 (C4/5).

HRMS (ESI) *m/z* $[M+H]^+$: calculated for $[C_6H_{12}NO_2]^+$: 130.0863; found: 130.0859.

IR (film) $\tilde{\nu}_{max}/cm^{-1}$ = 3372 (m, NH), 3182 (m, NH), 3013 (w, CH_{arom}), 2986 (w, CH_{aliph}), 2945 (w, CH_{aliph}), 2880 (w, CH_{aliph}), 2830 (w, CH_{aliph}), 1655 (s, C=O), 1421 (m, CH_{arom}), 1158 (m, C–N), 1088 (s, C–O).

2-Ethoxy-2-phenylacetamide (*rac*-3a)

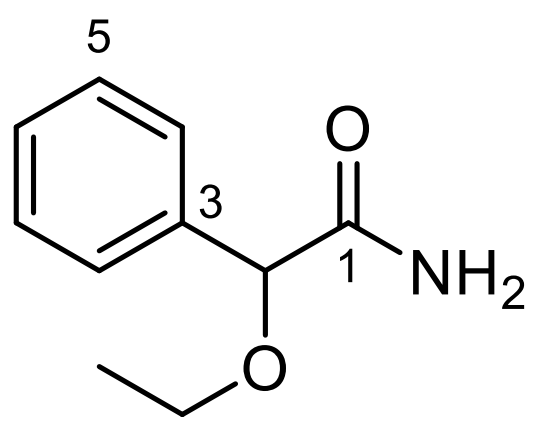

rac-**3a**
$C_{10}H_{13}NO_2$
MW = 179.22 $gmol^{-1}$

Following GP B, 2-ethoxy-2-phenylacetamide (*rac*-**3a**) was obtained from benzaldehyde (220 mg, 211 µL, 2.07 mmol, 1.00 equiv.) and EtOH (0.38 M) as a white solid (267mg, 1.49 mmol, 72% over two steps).

TLC (70% EtOAc/hexanes): R_f = 0.38 [UV, $KMnO_4$].

M.p.: 127-128 °C

^{1}**H-NMR** (400 MHz, $CDCl_3$, 300 K): δ [ppm] = 7.44 – 7.41 (m, 2H, H4), 7.38 – 7.29 (m, 3H, H5, H6), 6.73 (bs, 1H, NH_2^a), 5.95 (bs, 1H, NH_2^b), 4.71 (s, 1H, H2), 3.53 (q, ^3J = 7.0 Hz, 2H, OCH_2), 1.25 (t, ^3J = 7.0 Hz, 3H, CH_3).

^{13}**C-NMR** (101 MHz, $CDCl_3$, 300 K): δ [ppm] = 174.0 (C1), 137.5 (C3), 128.7 (C5), 128.5 (C6), 127.1 (C4), 82.1 (C2), 65.3 (OCH_2), 15.3 (CH_3).

HRMS (ESI) *m/z* $[M+H]^+$: calculated for $[C_{10}H_{14}NO_2]^+$: 180.1019; found: 180.1017.

IR (film) $\tilde{\nu}_{max}/cm^{-1}$ = 3383 (m, NH), 3166 (m, NH), 3032 (w, CH_{arom}), 2974 (w, CH_{aliph}), 2928 (w, CH_{aliph}), 2887 (w, CH_{aliph}), 2872 (w, CH_{aliph}), 1657 (s, C=O), 1497 (w, CH_{arom}), 1308 (m, C–N), 1070 (s, C–O).

2-Phenyl-2-propoxyacetamide (*rac*-3b)

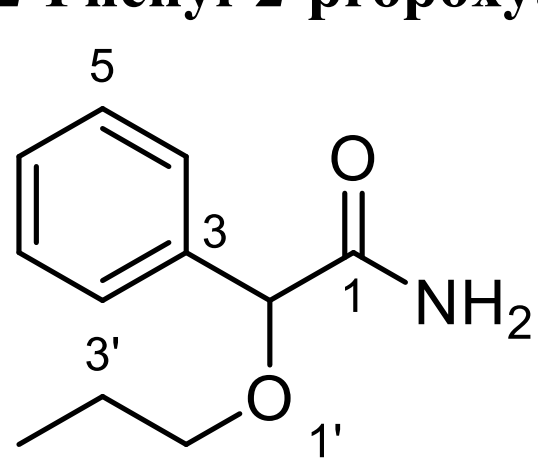

rac-**3b**
$C_{11}H_{15}NO_2$
MW = 193.25 $gmol^{-1}$

Following a modified GP D, 2-phenyl-2-propoxyacetamide (*rac*-**3b**) was obtained from 1-iodopropane and 2-methyl 3-hydroxy-2-phenylpropanoate (500 mg, 3.01 mmol, 1.00 equiv.) as a white solid (192 mg, 994 µmol, 33% over two steps).

TLC (50% EtOAc/hexanes): R_f = 0.40 [UV, $KMnO_4$].

M.p.: 77-73 °C

^{1}H-NMR (400 MHz, $CDCl_3$, 300 K): δ [ppm] = 7.44 – 7.42 (m, 2H, H4), 7.38 – 7.30 (m, 3H, H5, H6), 6.73 (bs, 1H, NH_2^a), 5.50 (bs, 1H, NH_2^b), 4.72 (s, 1H, H2), 3.43 (t, ^3J = 6.6 Hz, 2H, H2'), 1.65 (*virt.* sext, $^3J \approx {}^3J$ = 7.5 Hz, 2H, H3'), 0.95 (t, ^3J = 7.4 Hz, 3H, H4').

^{13}C-NMR (101 MHz, $CDCl_3$, 300 K): δ [ppm] = 173.8 (C1), 137.4 (C3), 128.7 (C5), 128.6 (C6), 127.1 (C4), 82.3 (C2), 71.5 (C2'), 23.1 (C3'), 10.7 (C4').

HRMS (ESI) *m/z* $[M+H]^+$: calculated for $[C_{11}H_{16}NO_2]^+$: 194.1176; found: 194.1169.

IR (film) $\tilde{\nu}_{max}/cm^{-1}$ = 3381 (m, NH), 3174 (m, NH), 3090 (w, CH_{arom}), 3066 (w, CH_{arom}), 3032 (w, CH_{arom}), 2963 (m, CH_{aliph}), 2932 (w, CH_{aliph}), 2876 (w, CH_{aliph}), 1657 (s, C=O), 1612 (m, C=C) 1396 (m, CH_{arom}), 1096 (s, C–N), 1073 (s, C–O).

2-Butoxy-2-phenylacetamide (*rac*-3c)

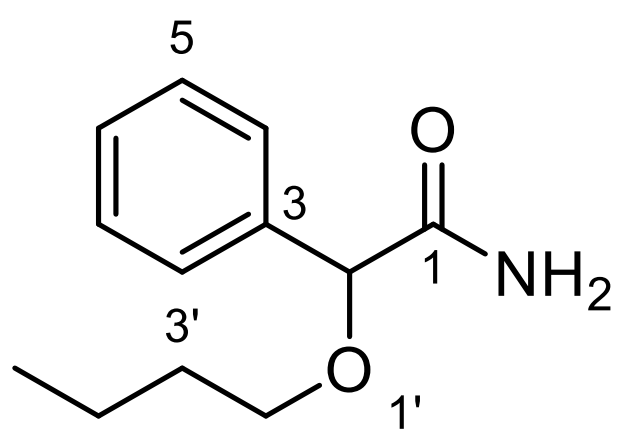

rac-**3c**
$C_{12}H_{17}NO_2$
MW = 207.27 $gmol^{-1}$

Following GP D, 2-butoxy-2-phenylacetamide (*rac*-**3c**) was obtained from 1-iodobutane and 2-methyl 3-hydroxy-2-phenylpropanoate (500 mg, 3.01 mmol, 1.00 equiv.) as a white solid (207 mg, 1.0 mmol, 33% over two steps).

TLC (50% EtOAc/hexanes): R_f = 0.55 [UV, $KMnO_4$].

M.p.: 120-123 °C

^{1}H-NMR (400 MHz, $CDCl_3$, 300 K): δ [ppm] = 7.43 – 7.29 (m, 5H, H4, H5, H6), 6.70 (bs, 1H, NH_2^a), 6.04 (bs, 1H, NH_2^b), 4.69 (s, 1H, H2), 3.46 (t, ^3J = 6.5 Hz, 2H, H2'), 1.64 – 1.56 (m, 2H, H3'), 1.44 – 1.34 (m, 2H, H4'), 0.91 (t, ^3J = 7.4 Hz, 3H, H5').

^{13}C-NMR (101 MHz, $CDCl_3$, 300 K): δ [ppm] = 174.1 (C1), 137.5 (C3), 128.7 (C5), 128.5 (C6), 127.1 (C4), 82.3 (C2), 69.6 (C2'), 31.9 (C3'), 19.5 (C4'), 14.0 (C5').

HRMS (ESI) *m/z* $[M+H]^+$: calculated for $[C_{12}H_{18}NO_2]^+$: 208.1332; found: 208.1330.

IR (film) $\tilde{\nu}_{max}/cm^{-1}$ = 3375 (m, NH), 3176 (m, NH), 2960 (m, CH_{arom}), 2936 (m, CH_{aliph}), 2868 (m, CH_{aliph}), 2779 (m, CH_{aliph}), 1657 (s, C=O), 1451 (m, CH_{arom}), 1098 (s, C–N), 1075 (s, C–O).

2-(But-3-en-1-yloxy)-2-phenylacetamide (*rac*-3d)

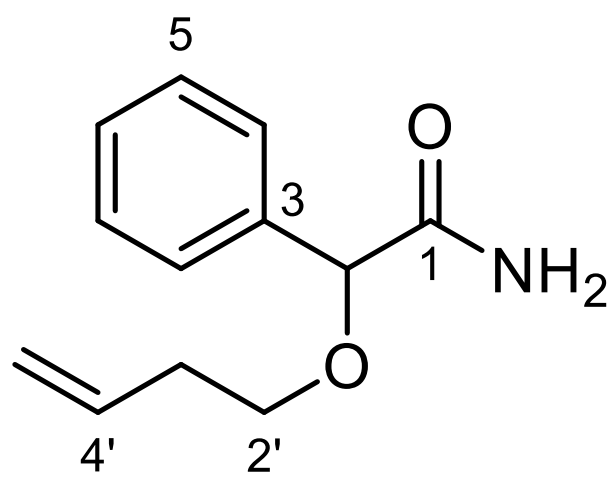

rac-3d

$C_{12}H_{15}NO_2$

MW = 205.26 gmol^{-1}

According to GP C, 2-(but-3-en-1-yloxy)-2-phenylacetamide (*rac*-**3d**) was obtained from benzaldehyde (300 mg, 288 µL, 2.83 mmol, 1.00 equiv.) and but-3-en-1-ol (0.76 M) as a white solid (179 mg, 872 µmol, 31% over two steps).

TLC (50% EtOAc/hexanes): R_f = 0.33 [UV, $KMnO_4$].

M.p.: 100-101 °C

^{1}H-NMR (400 MHz, $CDCl_3$, 300 K): δ [ppm] = 7.43 – 7.41 (m, 2H, H4), 7.38 – 7.30 (m, 3H, H5, H6), 6.72 (bs, 1H, NH_2^a), 5.86 – 5.75 (m, 2H, NH_2^b, H4'), 5.14 – 5.05 (m, 2H, H5'), 4.72 (s, 1H, H2), 3.58 – 3.48 (m, 2H, H2'), 2.38 (qt, $^3J \approx {}^3J$ = 6.5 Hz, $^4J \approx {}^4J$ = 1.4 Hz, 2H, H3').

^{13}C-NMR (101 MHz, $CDCl_3$, 300 K): δ [ppm] = 173.7 (C1), 137.2 (C3), 134.9 (C4'), 128.7 (C5), 128.6 (C6), 127.2 (C4), 117.2 (C5'), 82.3 (C2), 69.0 (C2'), 34.3 (C3').

HRMS (ESI) *m/z* $[M+H]^+$: calculated for $[C_{12}H_{16}NO_2]^+$: 206.1176; found: 206.1172.

IR (film) $\tilde{\nu}_{max}/cm^{-1}$ = 3371 (m, NH), 3174 (m, NH), 3069 (w, CH_{arom}), 3032 (w, CH_{arom}), 2980 (w, CH_{aliph}), 2905 (w, CH_{aliph}), 2866 (w, CH_{aliph}), 1659 (s, C=O), 1586 (m, C=C), 1452 (w, CH_{arom}), 1092 (s, C–N), 1073 (s, C–O).

2-Isopropoxy-2-phenylacetamide (*rac*-3e)

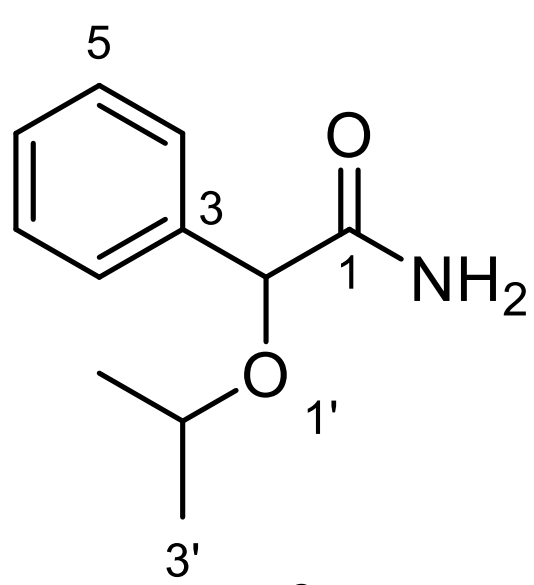

rac-**3e**

$C_{11}H_{15}NO_2$

MW = 193.25 gmol^{-1}

Following GP D, 2-isopropoxy-2-phenylacetamide (*rac*-**3e**) was obtained from 2-iodopropane and 2-methyl 3-hydroxy-2-phenylpropanoate (500 mg, 3.01 mmol, 1.00 equiv.) as a white solid (74 mg, 383 µmol, 13% over two steps).

TLC (50% EtOAc/hexanes): R_f = 0.40 [UV, $KMnO_4$].

M.p.: 107-109 °C

^{1}H-NMR (400 MHz, $CDCl_3$, 300 K): δ [ppm] = 7.45 – 7.42 (m, 2H, H4), 7.37 – 7.28 (m, 3H, H5, H6), 6.75 (bs, 1H, NH_2^a), 5.99 (bs, 1H, NH_2^b), 4.82 (s, 1H, H2), 3.68 (hept, ^3J = 6.1 Hz, 1H, H2'), 1.22 (d, ^3J = 6.1 Hz, 3H, H3'^{a}), 1.17 (d, ^3J = 6.1 Hz, 3H, H3'^{b}).

^{13}C-NMR (101 MHz, $CDCl_3$, 300 K): δ [ppm] = 174.6 (C1), 138.1 (C3), 128.6 (C5), 128.4 (C6), 127.1 (C4), 79.5 (C2), 70.9 (C2'), 22.9 (C3'^{b}), 21.7 (C3'^{a}).

HRMS (ESI) *m/z* $[M+H]^+$: calculated for $[C_{11}H_{16}NO_2]^+$: 194.1176; found: 194.1175.

IR (film) $\tilde{\nu}_{max}/cm^{-1}$ = 3401 (m, NH), 3170 (m, NH), 3032 (w, CH_{arom}), 2971 (m, CH_{aliph}), 2928 (w, CH_{aliph}), 2878 (w, CH_{aliph}), 1655 (s, C=O), 1605 (m, C=C) 1381 (m, CH_{arom}), 1081 (m, C–N), 1060 (s, C–O).

2-(2-Fluoroethoxy)-2-phenylacetamide (*rac*-3f)

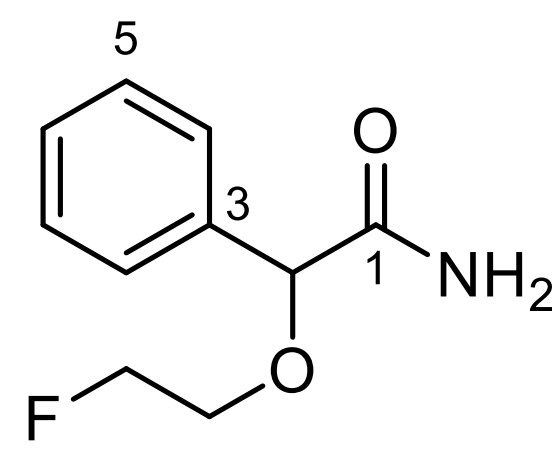

rac-**3f**
$C_{10}H_{12}FNO_2$
MW = 197.21 $gmol^{-1}$

Following GP B, 2-(2-fluoroethoxy)-2-phenylacetamide (*rac*-**3f**) was obtained from benzaldehyde (500 mg, 4.71 mmol, 1.00 equiv.) and 2-fluoroethanol (0.8 M) as a white solid (340 mg, 1.72 mmol, 37%).

TLC (50% EtOAc/hexanes): R_f = 0.43 [UV, $KMnO_4$].

M.p.: 110-113 °C

^{1}H-NMR (400 MHz, $CDCl_3$, 300 K): δ [ppm] = 7.44 – 7.33 (m, 5H, H4, H5, H6), 6.75 (bs, 1H, $NH_2{}^a$), 6.38 – 6.32 (m, 1H, $NH_2{}^b$), 4.78 (s, 1H, H2), 4.66 – 4.59 (m, 1H, CH_2F^a), 4.54 – 4.47 (m, 1H, CH_2F^b), 3.79 – 3.63 (m, 2H, OCH_2).

^{13}C-NMR (101 MHz, $CDCl_3$, 300 K): δ [ppm] = 173.4 (C1), 136.6 (C3), 128.8 (C6), 128.8 (C5), 127.2 (C4), 82.6 (d, $^1J_{CF}$ = 170 Hz, CH_2F), 82.5 (C2), 68.7 (d, $^2J_{CF}$ = 19.4 Hz, OCH_2)

^{19}F-NMR (376 MHz, $CDCl_3$, 300 K): δ [ppm] = −223.7.

HRMS (ESI) *m/z* $[M+H]^+$: calculated for $[C_{10}H_{13}FNO_2]^+$: 198.0925; found: 198.0924.

IR (film) $\tilde{\nu}_{max}/cm^{-1}$ = 3388 (m, NH), 3168 (m, NH), 3034 (w, CH_{arom}), 2982 (w, CH_{aliph}), 2917 (w, CH_{aliph}), 2868 (w, CH_{aliph}), 1657 (s, C=O), 1195 (w, C–N), 1094 (s, C–O), 1042 (s, C–F).

2-Phenyl-2-(3,3,3-trifluoropropoxy)acetamide (*rac*-3g)

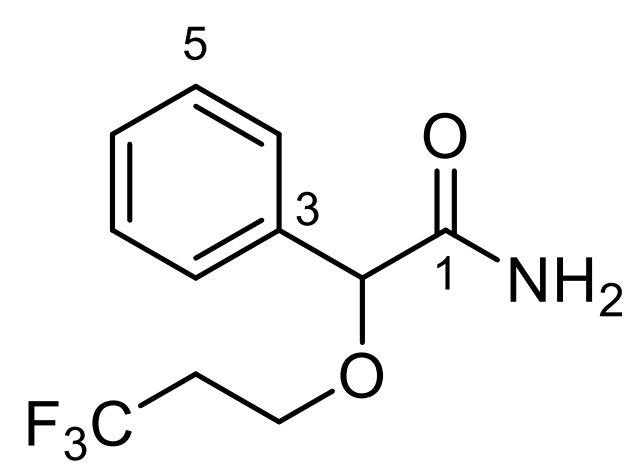

rac-**3g**
$C_{11}H_{12}F_3NO_2$
MW = 247.22 $gmol^{-1}$

Following GP D, 2-phenyl-2-(3,3,3-trifluoropropoxy)acetamide (*rac*-**3g**) was obtained from 1,1,1-trifluoro-3-iodopropane and methyl 3-hydroxy-2-phenylpropanoate (500 mg, 3.01 mmol, 1.00 equiv.) as a white semi-solid (24 mg, 105 μmol, 3% over two steps).

TLC (50% EtOAc/hexanes): R_f = 0.48 [UV, $KMnO_4$].

^{1}H-NMR (400 MHz, $CDCl_3$, 300 K): δ [ppm] = 7.43 – 7.32 (m, 5H, H4, H5, H6), 6.65 (bs, 1H, $NH_2{}^a$), 6.25 – 6.15 (m, 1H, $NH_2{}^b$), 4.72 (s, 1H, H2), 3.66 (t, ^3J = 6.1 Hz, 2H, OCH_2), 2.50 – 2.37 (m, 2H, CF_3CH_2).

^{13}C-NMR (101 MHz, $CDCl_3$, 300 K): δ [ppm] = 173.1 (C1), 136.3 (C3), 129.0 (C6), 128.9 (C5), 127.2 (C4), 126.2 (q, $^1J_{CF}$ = 277 Hz, CF_3), 82.6 (C2), 62.4 (q, $^3J_{CF}$ = 3.4 Hz, OCH_2), 34.3 (q, $^2J_{CF}$ = 28.8 Hz, CF_3CH_2)

^{19}F-NMR (376 MHz, $CDCl_3$, 300 K): δ [ppm] = −64.5.

IR (film) $\tilde{\nu}_{max}/cm^{-1}$ = 3479 (m, NH), 3297 (m, NH), 3034 (w, CH_{arom}), 2958 (w, CH_{aliph}), 2917 (m, CH_{aliph}), 2851 (w, CH_{aliph}), 1694 (s, C=O), 1674 (s, C=C), 1249 (s, C–F), 1144 (s, C–N), 1101 (s, C–O).

2-(Allyloxy)-2-phenylacetamide (*rac*-3h)

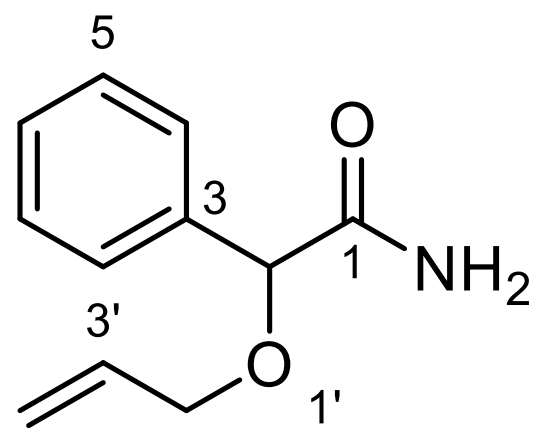

rac-**3h**
$C_{11}H_{13}NO_2$
MW = 191.23 $gmol^{-1}$

Following GP D, the compound *rac*-**3h** was obtained from allyl bromide and methyl 3-hydroxy-2-phenylpropanoate (500 mg, 3.01 mmol, 1.00 equiv.) as a white semi-solid (120 mg, 627 µmol, 21% over two steps).

TLC (50% EtOAc/hexanes): R_f = 0.40 [UV, $KMnO_4$].

M.p.: 84-85 °C

^{1}H-NMR (400 MHz, $CDCl_3$, 300 K): δ [ppm] = 7.44 – 7.31 (m, 5H, H4, H5, H6), 6.72 (bs, 1H, NH_2^a), 5.91 (dddd, ^3J = 17.2 Hz, ^3J = 10.4 Hz, ^3J = 6.0 Hz, ^3J = 5.2 Hz, H3'), 5.75 – 5.66 (m, 1H, NH_2^b), 5.31 – 5.22 (m, 2H, H4'), 4.79 (s, 1H, H2), 4.06 (*virt.* ddt, ^2J = 12.7 Hz, ^3J = 5.3 Hz, $^4J \approx {}^4J$ = 1.5 Hz, 1H, OCH_2^a), 3.97 (*virt.* ddt, ^2J = 12.7 Hz, ^3J = 6.0 Hz, $^4J \approx {}^4J$ = 1.4 Hz, 1H, OCH_2^b).

^{13}C-NMR (101 MHz, $CDCl_3$, 300 K): δ [ppm] = 173.6 (C1), 137.0 (C3), 133.7 (C3'), 128.8 (C5), 128.7 (C6), 127.3 (C4), 118.1 (C4'), 81.3 (C2), 70.3 (C2').

HRMS (ESI) *m/z* $[M+H]^+$: calculated for $[C_{11}H_{14}NO_2]^+$: 192.1019; found: 192.1018.

IR (film) $\tilde{\nu}_{max}/cm^{-1}$ = 3388 (m, NH), 3163 (m, NH), 3088 (w, CH_{arom}), 3034 (w, CH_{arom}), 2978 (w, CH_{aliph}), 2915 (w, CH_{aliph}), 2861 (w, CH_{aliph}), 1657 (s, C=O), 1456 (w, CH_{arom}), 1198 (w, C–N), 1059 (s, C–O).

2-(But-3-yn-1-yloxy)-2-phenylacetamide (*rac*-3i)

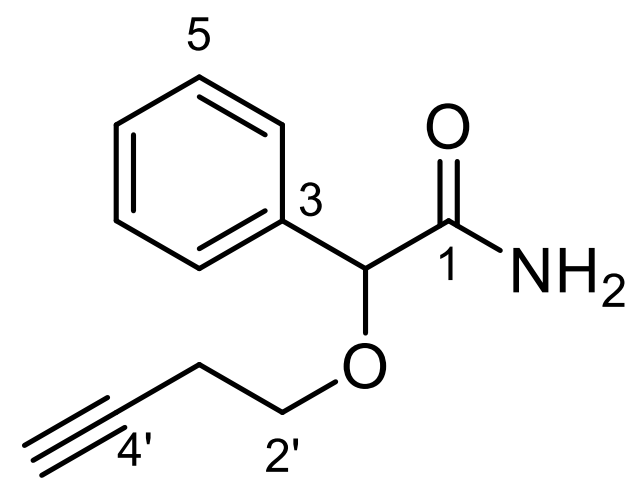

rac-3i
$C_{12}H_{13}NO_2$
MW = 203.24 gmol^{-1}

Following GP B, 2-(but-3-yn-1-yloxy)-2-phenylacetamide (*rac*-**3i**) was obtained from benzaldehyde (180 mg, 173 µL, 1.70 mmol, 1.00 equiv.) and but-3-yn-1-ol (0.76 M) as a white solid (158 mg, 777 µmol, 46%).

TLC (80% EtOAc/hexanes): R_f = 0.41 [UV, $KMnO_4$].

M.p.: 93-94 °C

^{1}H-NMR (400 MHz, $CDCl_3$, 300 K): δ [ppm] = 7.45 – 7.42 (m, 2H, H4), 7.39 – 7.30 (m, 3H, H5, H6), 6.83 (bs, 1H, NH_2^a), 6.04 (bs, 1H, NH_2^b), 4.76 (s, 1H, H2), 3.65 – 3.56 (m, 2H, H2'), 2.58 – 2.44 (m, 2H, H3'), 2.01 (t, ^4J = 2.6 Hz, 1H, H5').

^{13}C-NMR (101 MHz, $CDCl_3$, 300 K): δ [ppm] = 173.5 (C1), 136.8 (C3), 128.7 (C5), 127.2 (C4/C6), 127.2 (C4/C6), 82.4 (C2), 81.2 (C4'), 69.9 (C5'), 67.6 (C2'), 20.1 (C3').

HRMS (ESI) *m/z* $[M+H]^+$: calculated for $[C_{12}H_{14}NO_2]^+$: 204.1019; found: 204.1018.

IR (film) $\tilde{\nu}_{max}/cm^{-1}$ = 3383 (m, NH), 3276 (m, CH_{alkine}), 3155 (m, NH), 3036 (w, CH_{arom}), 2952 (w, CH_{aliph}), 2920 (w, CH_{aliph}), 2876 (w, CH_{aliph}), 1657 (s, C=O), 1457 (w, CH_{arom}), 1079 (s, C–N), 1068 (s, C–O).

2-Phenoxy-2-phenylacetamide (*rac*-3j)

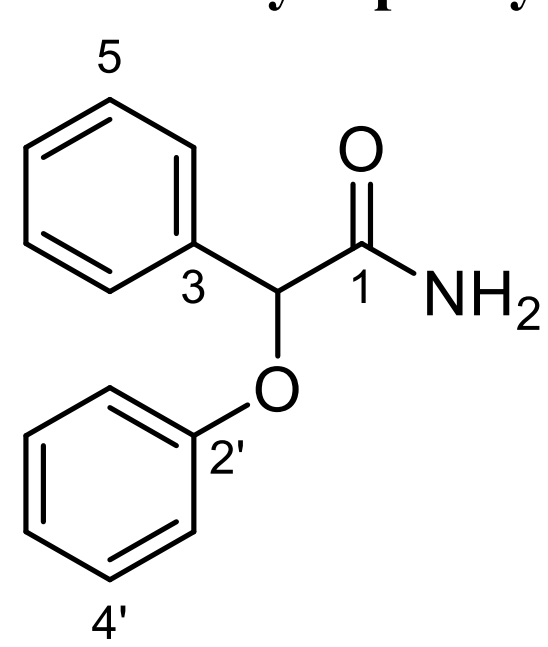

rac-**3j**
$C_{14}H_{13}NO_2$
MW = 227.26 gmol^{-1}

Following GP A, 2-phenoxy-2-phenylacetamide (*rac*-**3j**) was obtained from 2-phenoxy-2-phenylacetic acid (90.0 mg, 394 µmol, 1.00 equiv.) as a white solid (71.0 mg, 312 µmol, 79%).

TLC (50% EtOAc/hexanes): R_f = 0.50 [UV, $KMnO_4$].

M.p.: 169-170 °C

^{1}H-NMR (400 MHz, $CDCl_3$, 300 K): δ [ppm] = 7.57 – 7.54 (m, 2H, H4), 7.41 – 7.26 (m, 5H, H5, H6, H3'), 7.03 – 7.6.95 (m, 3H, H4', H5'), 6.67 (bs, 1H, NH_2^a), 6.05 (bs, 1H, NH_2^b), 5.54 (s, 1H, H2).

^{13}C-NMR (101 MHz, $CDCl_3$, 300 K): δ [ppm] = 172.6 (C1), 156.9 (C2'), 136.3 (C3), 129.9 (C5), 128.8 (C3'), 128.8 (C6), 126.7 (C4), 122.4 (C5'), 115.9 (C4'), 80.4 (C2).

HRMS (ESI) *m/z* $[M+H]^+$: calculated for $[C_{14}H_{14}NO_2]^+$: 228.1019; found: 228.1010.

IR (film) $\tilde{\nu}_{max}/cm^{-1}$ = 3377 (m, NH), 3163 (m, NH), 3042 (w, CH_{arom}), 1658 (s, C=O), 1588 (m, C=C), 1489 (m, CH_{arom}), 1217 (s, C–N), 1039 (s, C–O).

2-Benzyloxy-2-phenylacetamide (*rac*-3k)

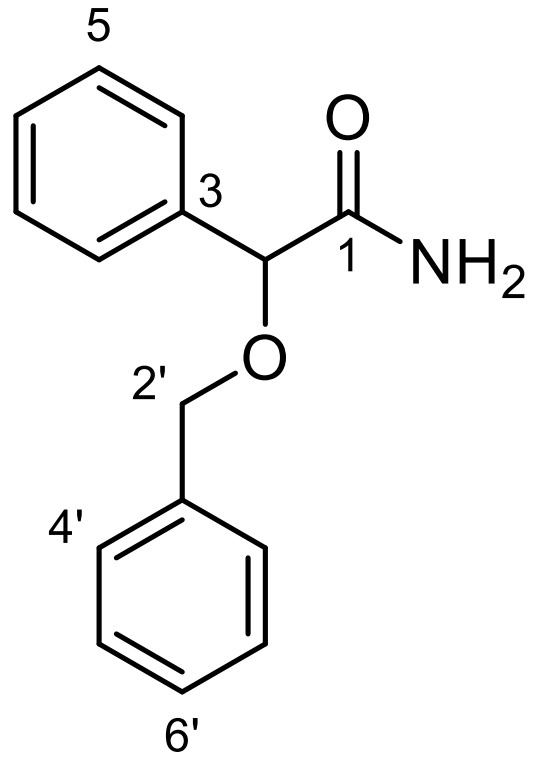

rac-**3k**
$C_{15}H_{15}NO_2$
MW = 241.29 $gmol^{-1}$

Following GP B, 2-benzyloxy-2-phenylacetamide (*rac*-**3k**) was obtained from benzaldehyde (400 mg, 385 µL, 3.77 mmol, 1.00 equiv.) and benzyl alcohol (0.76 M) as a white solid (418 mg, 1.73 mmol, 46% over two steps).

TLC (50% EtOAc/hexanes): $R_f = 0.24$ [UV, $KMnO_4$].

M.p.: 142-143 °C

^{1}H-NMR (400 MHz, $CDCl_3$, 300 K): δ [ppm] = 7.47 – 7.44 (m, 2H, H4), 7.42 – 7.30 (m, 8H, H5, H6, H4', H5', H6'), 6.76 (bs, 1H, NH_2^a), 5.92 (bs, 1H, NH_2^b), 4.83 (s, 1H, H2), 4.59 (d, ^2J = 11.6 Hz, 1H, C2'^{a}), 4.46 (d, ^2J = 11.6 Hz, 1H, C2'^{b}).

^{13}C-NMR (101 MHz, $CDCl_3$, 300 K): δ [ppm] = 173.6 (C1), 137.1 (C3'), 136.8 (C3), 128.8 (C5), 128.8 (C6'), 128.7 (C5'), 128.3 (C6), 128.1 (C4'), 127.4 (C4), 81.3 (C2), 71.3 (C2').

HRMS (ESI) *m/z* $[M+H]^+$: calculated for $[C_{15}H_{16}NO_2]^+$: 242.1176; found: 242.1172.

IR (film) $\tilde{\nu}_{max}/cm^{-1}$ = 3360 (m, NH), 3166 (m, NH), 3088 (w, CH_{arom}), 3062 (w, CH_{arom}), 3028 (w, CH_{arom}), 3008 (w, CH_{arom}), 2885 (w, CH_{aliph}), 2861 (m, CH_{aliph}), 1659 (s, C=O), 1450 (w, CH_{arom}), 1090 (s, C–N), 1070 (m, C–O).

2-Phenyl-2-((triethylsilyl)oxy)acetamide (*rac*-3l)

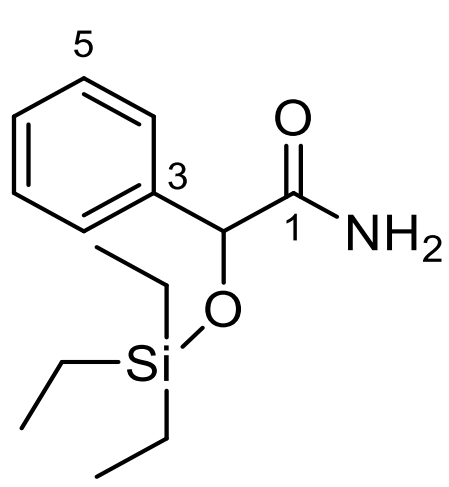

rac-**3l**
$C_{14}H_{23}NO_2Si$
MW = 265.43 gmol-1

A solution of 2-hydroxy-2-phenylacetamide (400 mg, 2.65 mmol, 1.00 equiv.) and imidazole (216 mg, 3.18 mmol, 3.00 equiv.) in dry CH_2Cl_2 (2.5 mL) was cooled to 0 ºC under an argon atmosphere followed by the dropwise addition of triethylchlorosilane (489 µL, 2.11 mmol, 1.10 equiv.) over 15 min. After the completion of the addition, the reaction mixture was stirred at r.t. for 6 h. The reaction mixture was diluted with 15 mL of CH_2Cl_2 and washed with 1 M HCl (3 × 5 mL) and water (3 × 5 mL). extracted with ethyl acetate (3 × 10 mL). The combined organic layer was washed with brine and dried over Na_2SO_4 and concentrated under reduced pressure. The obtained the crude reaction mixture was subjected to FCC (SiO_2, 60 → 100% EtOAc/hexanes) to yield 2-phenyl-2-((triethylsilyl)oxy)acetamide (*rac*-**3l**) as a colorless thick oil (560 mg, 2.11 mmol, 80%).

TLC (50% EtOAc/hexanes): $R_f = 0.39$ [UV, $KMnO_4$].

^{1}H-NMR (400 MHz, $CDCl_3$, 300 K): δ [ppm] = 7.40 – 7.37 (m, 2H, H4), 7.28 – 7.19 (m, 3H, H5, H6), 6.74 (bs, 1H, $NH_2{}^a$), 5.79 – 5.70 (m, 1H, $NH_2{}^b$), 4.99 (s, 1H, H2), 0.83 (t, ^3J = 7.9 Hz, 9H, CH_3), 0.59 – 0.44 (m, 6H, CH_2).

^{13}C-NMR (101 MHz, $CDCl_3$, 300 K): δ [ppm] = 175.4 (C1), 139.9 (C3), 128.5 (C5), 128.3 (C6), 126.4 (C4), 75.7 (C2), 6.74 (CH_3), 4.74 (CH_2).

HRMS (ESI) *m/z* $[M+H]^+$: calculated for $[C_{14}H_{24}NO_2Si]^+$: 266.1571; found: 266.1560.

IR (film) $\tilde{\nu}_{max}/cm^{-1}$ = 3476 (m, NH), 3168 (w, NH), 3090 (w, CH_{arom}), 3064 (w, CH_{arom}), 3032 (w, CH_{arom}), 2954 (m, CH_{aliph}), 2911 (w, CH_{aliph}), 2876 (m, CH_{aliph}), 1687 (s, C=O), 1191 (w, C–N), 1094 (s, C–O), 1068 (s, C–Si).

2-Amino-2-oxo-1-phenylethyl 4-methylbenzenesulfonate (*rac*-3m)

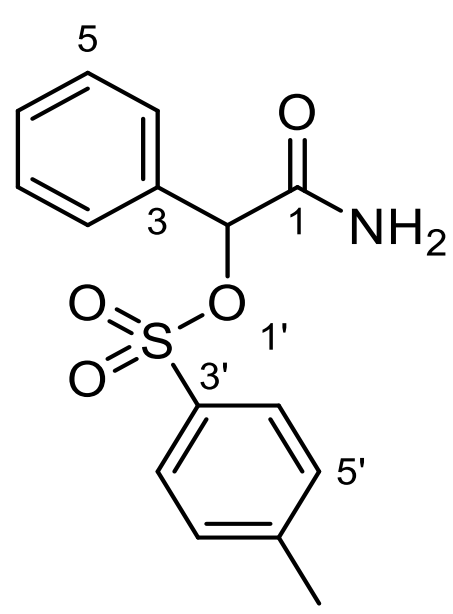

rac-**3m**
$C_{15}H_{15}NO_4S$
MW = 305.35 gmol-1

A solution of 2-hydroxy-2-phenylacetamide (400 mg, 2.65 mmol, 1.00 equiv.), *N*,*N*-diisopropylethylamine (DIPEA) (1.35 mL, 7.94 mmol, 3.00 equiv.), 4-dimethylaminopyridine (DMAP) (48.5 mg, 397 µmol, 0.15 equiv.) and *p*-toluenesulfonyl chloride (TsCl) (757 mg, 3.97 mmol, 1.50 equiv.) in CH_3CN (4 mL) was stirred for 6 h at r.t. Then, the solvent was removed under reduced pressure. The mixture was diluted with 15 mL of H_2O and was extracted with ethyl acetate (10 mL × 3). The combined organic layer was washed with brine and dried over Na_2SO_4 and concentrated under reduced pressure. Subsequently, the crude reaction mixture was subjected to FCC (SiO_2, 60 → 100% EtOAc/hexanes) to yield 2-amino-2-oxo-1-phenylethyl 4-methylbenzenesulfonate (*rac*-**3m**) as a white solid (523 mg, 1.71 mmol, 65%).

TLC (70% EtOAc/hexanes): R_f = 0.2 [UV, $KMnO_4$].

M.p.: 148-149 °C

^{1}H-NMR (400 MHz, DMSO-d$_6$, 300 K): δ [ppm] = 7.77 – 7.75 (m, 3H, H4', $NH_2{}^a$), 7.43 – 7.40 (m, 3H, H5', $NH_2{}^b$), 7.35 – 7.31 (m, 5H, H4, H5, H6), 5.68 (s, 1H, H2), 2.39 (s, 3H, CH_3).

^{13}C-NMR (101 MHz, , DMSO-d$_6$, 300 K): δ [ppm] = 167.9 (C1), 145.1 (C6'), 134.6 (C3), 132.8 (C3'), 130.0 (C5') 128.5 (C4'), 127.7 (C5), 127.1 (C4), 79.7 (C2), 21.1 (CH_3).

HRMS (ESI) *m/z* $[M +H]^+$: calculated for $[C_{15}H_{16}NO_4S]^+$: 306.0795; found: 306.0782.

IR (film) $\tilde{\nu}_{max}/cm^{-1}$ = 3414 (w, NH), 3169 (w, NH), 1668 (s, C=O), 1457 (s, CH_{arom}), 1177 (s, C–N), 1094 (m, C–O), 1034 (m, C–Cl), 962 (s, S=O).

2-((2-Methoxyethoxy)methoxy)-2-phenylacetamide (*rac*-3o)

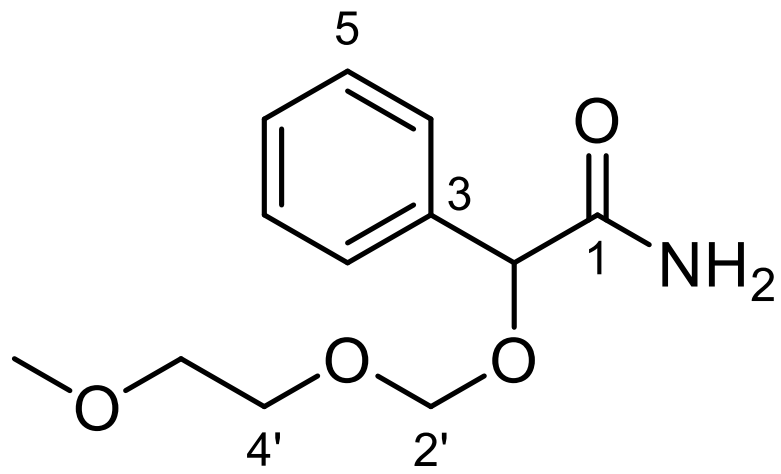

rac-**3o**
$C_{12}H_{17}NO_4$
MW = 239.27 $gmol^{-1}$

2-Methoxyethoxymethyl chloride (1.12 g, 1.03 mL, 9.03 mmol, 1.50 equiv.) was added to a solution of methyl-2-hydroxy-2-phenylacetate (1.00 g, 6.02 mmol, 1.00 equiv.) and NEt^iPr_2 (1.17 g, 1.58 mL, 9.03 mmol, 1.50 equiv.). in CH_2Cl_2 (15 mL) at 0 °C and the resulting solution was allowed to warm to r.t. and stirred for 14 h. Subsequently, aqueous ammonia (1.54 g, 6.45 mL, 90.3 mmol, 60.0 equiv.) was added and the resulting reaction mixture was stirred at r.t. for 30 h, before water (10 mL) and CH_2Cl_2 (10 mL) were added, the layers were separated and the aqueous layer was extracted thrice with CH_2Cl_2 (3 × 10 mL). The combined organic layers were washed with brine and dried over Na_2SO_4. The solvents were removed under reduced pressure, and the crude product was subjected to flash column chromatography (FCC) (SiO_2, EtOAc/hexanes 50 → 100%) to yield 2-((2-methoxyethoxy)methoxy)-2-phenylacetamide (*rac*-**3o**) as a white solid (110 mg, 434 µmol, 29% over two steps).

TLC (70% EtOAc/hexanes): R_f = 0.40 [UV, $KMnO_4$].

M.p.: 60-61 °C

^{1}H-NMR (400 MHz, $CDCl_3$, 300 K): δ [ppm] = 7.43 – 7.40 (m, 2H, H4), 7.37 – 7.31 (m, 3H, H5, H6), 6.72 (bs, 1H, $NH_2{}^a$), 6.17 (bs, 1H, $NH_2{}^b$), 5.09 (s, 1H, H2), 4.78 (d, ^2J = 6.9 Hz, 1H, C2’^{a}), 4.70 (d, ^2J = 6.9 Hz, 1H, C2’^{b}), 3.76 (ddd, ^2J = 10.9 Hz, ^3J = 5.9 Hz, ^3J = 3.3 Hz, 1H, H4’^{a}), 3.62 (ddd, ^2J = 10.9 Hz, ^3J = 5.9 Hz, ^3J = 3.4 Hz, 1H, H4’^{b}), 3.53 – 3.44 (m, 2H, H5’), 3.36 (s, 3H, CH_3).

^{13}C-NMR (101 MHz, $CDCl_3$, 300 K): δ [ppm] = 173.6 (C1), 136.8 (C3), 128.7 (C6), 128.7 (C5), 127.7 (C4), 93.7 (C2’), 78.1 (C2), 71.7 (C5’), 67.8 (C4’), 59.1 (CH_3).

HRMS (ESI) *m/z* $[M+H]^+$: calculated for $[C_{12}H_{18}NO_4]^+$: 240.1230; found: 240.1225.

IR (film) $\tilde{\nu}_{max}/cm^{-1}$ = 3371 (m, NH), 3174 (m, NH), 3032 (w, CH_{arom}), 2917 (w, CH_{aliph}), 2891 (w, CH_{aliph}), 2835 (w, CH_{aliph}), 1659 (s, C=O), 1454 (w, CH_{arom}), 1098 (s, C–N), 1016 (s, C–O), 982 (s, C–O).

(*R*)-2-((*S*)-2-Methylbutoxy)-2-phenylacetamide (2(*R*)-3p) and (*S*)-2-((*S*)-2-Methylbutoxy)-2-phenylacetamide (2(*S*)-3p)

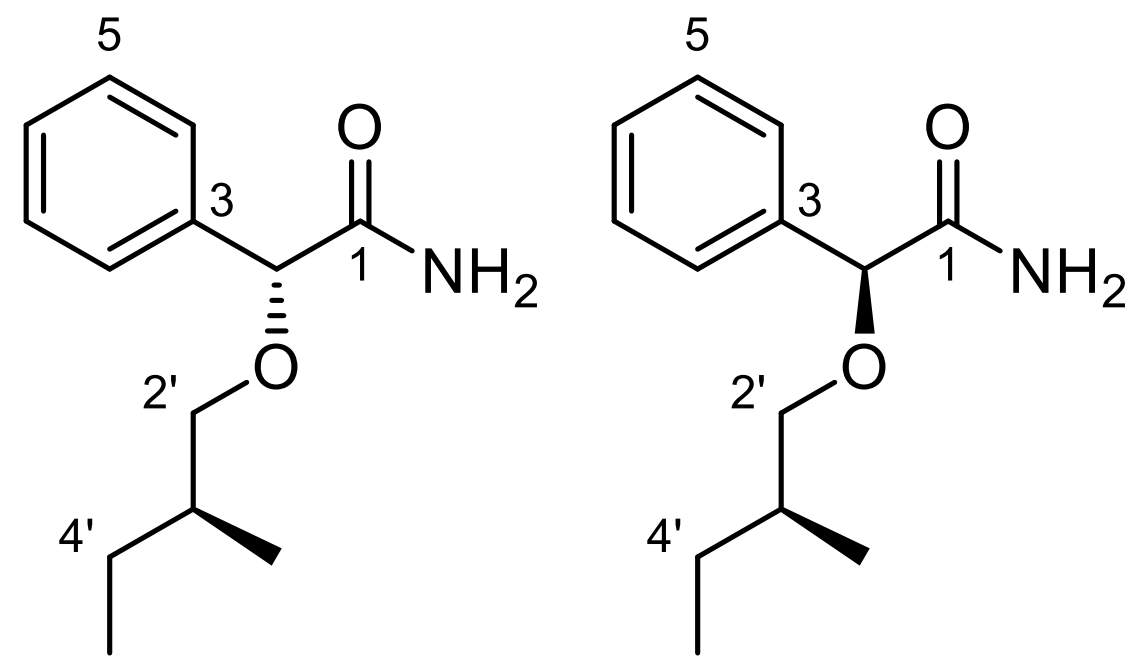

2(*R*)-**3p** 2(*S*)-**3p**

$C_{13}H_{19}NO_2$

MW = 221.30 gmol^{-1}

Following GP B, a diastereomeric mixture of (*R*)-2-((*S*)-2-methylbutoxy)-2-phenylacetamide (2(*R*)-**3p**) and (*S*)-2-((*S*)-2-methylbutoxy)-2-phenylacetamide (2(*S*)-**3p**) (d.r. = 1:1) was obtained from benzaldehyde (240 mg, 231 µL, 2.26 mmol, 1.00 equiv.) and (*S*)-2-methylbutan-1-ol (0.76 M) as a white solid (111 mg, 502 µmol, 22% over two steps).

TLC (50% EtOAc/hexanes): R_f = 0.40 [UV, $KMnO_4$].

M.p.: 71-72 °C

^{1}H-NMR (400 MHz, $CDCl_3$, 300 K): δ [ppm] = 7.43 – 7.40 (m, 2H, H4), 7.38 – 7.29 (m, 3H, H5, H6), 6.69 (bs, 1H, NH_2^a), 5.96 (bs, 1H, NH_2^b), 4.68 (s, 1H, H2), 3.35 – 3.31 (m, 1H, H1'^{a}), 3.28 – 3.24 (m, 1H, H2'^{b}), 1.74 – 1.65 (m, 1H, H3'), 1.52 – 1.39 (m, 1H, H4'^{a}), 1.24 – 1.12 (m, 1H, H4'^{a}), 0.94 – 0.92 (m, 3H, C2'CH_3), 0.90 – 0.86 (m, 3H, H5').

^{13}C-NMR (101 MHz, $CDCl_3$, 300 K): δ [ppm] = 174.0 (C1), 174.0 (C1), 137.4 (C3), 137.4 (C3), 128.7 (C5), 128.7 (C5), 128.5 (C6), 128.5 (C6), 127.1 (C4), 127.1 (C4), 82.5 (C2), 82.4 (C2), 74.9 (C2'), 74.9 (C2'), 35.2 (C3') 35.1 (C3'), 26.3 (C4'), 26.3 (C4'), 16.8 (C3'*C*H_3), 16.7 (C3'*C*H_3), 11.5 (C5'), 11.4 (C5').

HRMS (ESI) *m/z* $[M+H]^+$: calculated for $[C_{13}H_{20}NO_2]^+$: 222.1489; found: 222.1485.

IR (film) $\tilde{\nu}_{max}$/cm^{-1} = 3394 (m, NH), 3185 (m, NH), 3062 (w, CH_{arom}), 3032 (w, CH_{arom}), 2961 (w, CH_{aliph}), 2909 (w, CH_{aliph}), 2876 (w, CH_{aliph}), 1657 (s, C=O), 1452 (w, CH_{arom}), 1090 (s, C–N), 1073 (s, C–O).

(2*R*)-2-(2-((1*R*)-6,6-dimethylbicyclo[3.1.1]hept-2-en-2-yl)ethoxy)-2-phenylacetamide and (2*S*)-2-(2-((1*R*)-6,6-dimethylbicyclo[3.1.1]hept-2-en-2-yl)ethoxy)-2-phenylacetamide (3q)

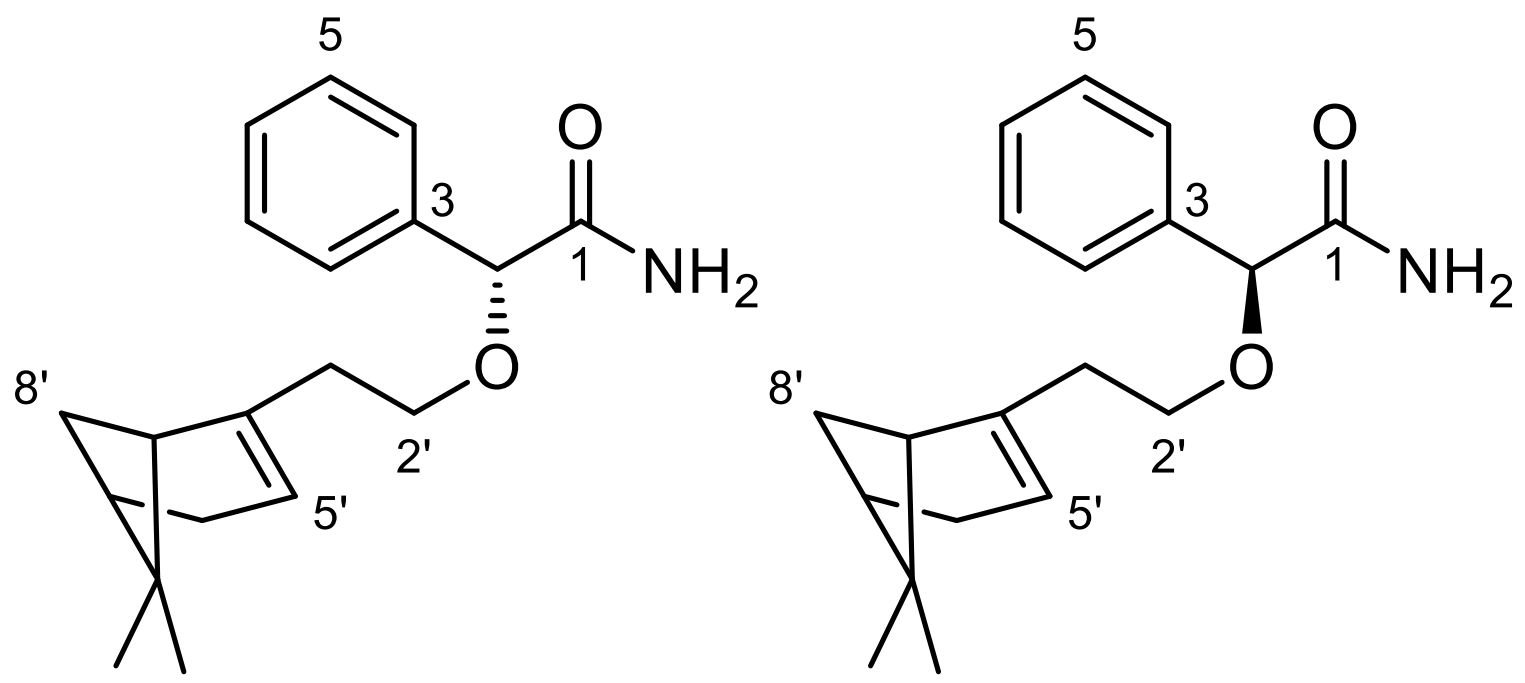

(2*R*)-**3q** (2*S*)-**3q**

$C_{19}H_{25}NO_2$

MW = 299.41 $gmol^{-1}$

Following GP B, a diastereomeric mixture of (2*R*)-2-(2-((1*R*)-6,6-dimethylbicyclo[3.1.1]hept-2-en-2-yl)ethoxy)-2-phenylacetic acid ((2*R*)-**3q**) and (2*S*)-2-(2-((1*R*)-6,6-dimethylbicyclo[3.1.1]hept-2-en-2-yl)ethoxy)-2-phenylacetic acid ((2*S*)-**3q**) (d.r. = 1:1) was obtained from benzaldehyde (700 mg, 673 µL, 6.60 mmol, 1.00 equiv.) and (1*R*)-(−)-nopol (0.76 M) as as a white solid (140 mg, 468 µmol, 7% over two steps).

TLC (40% EtOAc/hexanes): R_f = 0.30 [UV, $KMnO_4$].

M.p.: 107-108 °C

^{1}H-NMR (400 MHz, $CDCl_3$, 300 K): δ [ppm] = 7.42 – 7.29 (m, 5H, H4, H5, H6), 6.73 (bs, 1H, $NH_2{}^a$), 6.06 (bs, 1H, $NH_2{}^b$), 5.27 – 5.26 (m, 1H, H5’), 4.69 – 4.68 (m, 1H, H2), 3.53 – 3.41 (m, 2H, Hx), 2.38 – 2.16 (m, 5H, H3’, H6’/H8’, H7’), 2.10 – 1.98 (m, 2H, H6’/H8’), 1.26 – 1.25 (m, 3H, $CH_3{}^a$), 1.15 – 1.11 (m, 1H, H9’), 0.81 – 0.80 (m, 3H, $CH_3{}^a$).

^{13}C-NMR (101 MHz, $CDCl_3$, 300 K): δ [ppm] = 174.0 (C1, d1), 174.0 (C1, d2), 144.8 (C4’, d1), 144.8 (C4’, d2), 137.3 (C3, d1), 137.3 (C3, d1), 128.6 (C5, d1), 128.6 (C5, d2), 128.5 (C6, d1), 128.5 (C6, d2), 127.2 (C4, d1), 127.2 (C4, d2), 118.5 (C5’, d1), 118.5 (C5’, d2), 82.2 (C2, d1), 82.2 (C2, d2), 68.1 (C2’, d1), 67.9 (C2’, d2), 45.9 (C6’/C8’, d1), 45.9 (C6’/C8’, d2), 40.8 (C6’/C8’), 38.1 (*C*(CH_3)$_2$, d1), 38.1 (*C*(CH_3)$_2$, d2), 37.2 (C3’), 31.8 (C9’), 31.5 (C7’), 26.4 ($CH_3{}^a$, d1), 26.4 ($CH_3{}^a$, d2), 21.3 ($CH_3{}^b$, d1), 21.2 ($CH_3{}^b$, d2).

HRMS (ESI) *m/z* $[M+H]^+$: calculated for $[C_{19}H_{26}NO_2]^+$: 300.1958; found: 300.1952.

IR (film) $\tilde{\nu}_{max}/cm^{-1}$ = 3381 (m, NH), 3166 (m, NH), 3032 (w, CH_{arom}), 2982 (w, CH_{aliph}), 2912 (m, CH_{aliph}), 2878 (w, CH_{aliph}), 2831 (w, CH_{aliph}), 1657 (s, C=O), 1454 (w, CH_{arom}), 1077 (s, C–N), 1062 (s, C–O).

(*R*)-2-((*S*)-sec-butoxy)-2-phenylacetamide (2(*R*)-3r) and (*S*)-2-((*S*)-sec-butoxy)-2-phenylacetamide (2(*S*)-3r)

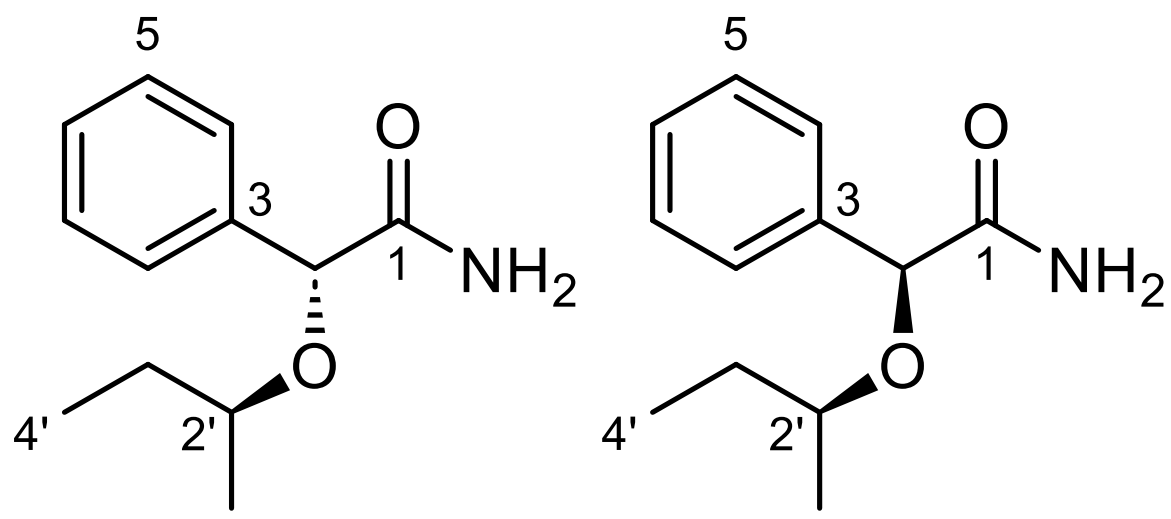

(2*R*)-**3r** (2*S*)-**3r**

$C_{12}H_{17}NO_2$

MW = 207.27 gmol^{-1}

Following GP B, a diastereomeric mixture of (*R*)-2-((*S*)-sec-butoxy)-2-phenylacetamide (2(*R*)-**3r**) and (*S*)-2-((*S*)-sec-butoxy)-2-phenylacetamide (2(*S*)-**3r**) (d.r. = 1:1) was obtained from benzaldehyde (200 mg, 192 µL, 1.88 mmol, 1.00 equiv.) and (*S*)-butan-2-ol (0.76 M) as a white solid (132 mg, 637 µmol, 34% over two steps).

TLC (50% EtOAc/hexanes): R_f = 0.24 [UV, $KMnO_4$].

M.p.: 50-51 °C

^{1}H-NMR (400 MHz, $CDCl_3$, 300 K): δ [ppm] = 7.46 – 7.42 (m, 2H, H4), 7.37 – 7.28 (m, 3H, H5, H6), 6.76 (bs, 1H, $NH_2{}^a$), 6.22 (bs, 1H, $NH_2{}^b$), 4.82 – 4.82 (m, 1H, H2), 3.52 (*virt.* sext, $^3J \approx {}^3J$ = 6.3 Hz, 1H, H2', d1), 3.52 (*virt.* sext, $^3J \approx {}^3J$ = 6.1 Hz, 1H, H2', d2), 1.69 – 1.40 (m, 2H, H3'), 1.18 (d, ^3J = 6.1 Hz, 3H, C2'CH_3, d2), 1.10 (d, ^3J = 6.3 Hz, 3H, C2'CH_3, d1), 0.93 (t, ^3J = 7.5 Hz, 3H, C4', d1/d2), 0.85 (t, ^3J = 7.5 Hz, 3H, C4', d1/d2).

^{13}C-NMR (101 MHz, $CDCl_3$, 300 K): δ [ppm] = 174.8 (C1, d1), 174.7 (C1, d2), 138.4 (C3, dx), 137.9 (C3, dx), 128.6 (C5, dx), 128.5 (C5, dx), 128.4 (C6, dx), 128.3 (C6, dx), 127.3 (C4, dx), 127.0 (C4, dx), 79.9 (C2, dx), 79.4 (C2, dx), 76.3 (C2', d1), 75.4 (C2', d2), 29.8 (Cx, dx), 28.7 (Cx, dx), 19.9 (C2'*C*H_3, d1), 18.8 (C2'*C*H_3, d2), 9.90 (C4', dx), 9.60 (C4', dx).

HRMS (ESI) *m/z* $[M+H]^+$: calculated for $[C_{12}H_{18}NO_2]^+$: 208.1332; found: 208.1330.

IR (film) $\tilde{\nu}_{max}$/cm^{-1} = 3461 (m, NH), 3157 (m, NH), 3064 (w, CH_{arom}), 3030 (w, CH_{arom}), 2969 (m, CH_{aliph}), 2930 (w, CH_{aliph}), 2879 (w, CH_{aliph}), 1675 (s, C=O), 1590 (m, C=C), 1452 (w, CH_{arom}), 1087 (m, C–N), 1064 (s, C–O).

5-Methylisochromane-1-carboxamide (*rac*-4b)

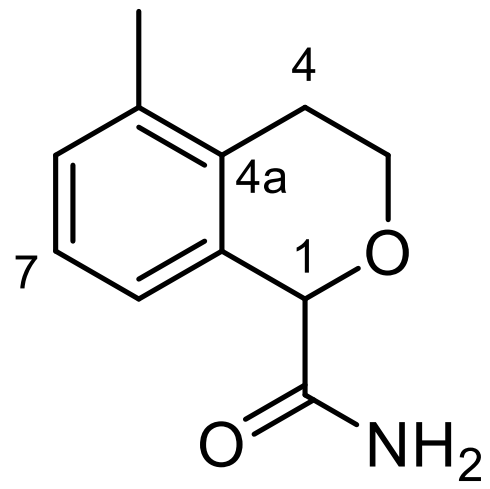

rac-**4b**
$C_{11}H_{13}NO_2$
MW = 191.23 $gmol^{-1}$

A solution of 2-(o-tolyl)ethan-1-ol (700 mg, 5.14 mmol, 1.00 equiv.) and glyoxylic acid monohydrate (520 mg, 388 µL, 5.65 mmol, 1.10 equiv.) in trifluoroacetic acid (2.6 mL) was added to a pressure tube (max. 150 psig at 120 °C) under an argon atmosphere. The reaction mixture was heated to 75 °C and stirred at this temperature for 16 h. Subsequently, the solvent was removed under reduced pressure. Water (10 mL) was added to the residue, and the mixture was basified with aq. ammonia to pH > 7. The aqueous layer was washed thrice with diethyl ether (3 × 10 mL), then acidified with $NaHSO_4$ to pH ~ 3, and extracted thrice with EtOAc (3 × 10 mL). The combined organic layers were concentrated under reduced pressure to obtain the crude intermediary 5-methylisochromane-1-carboxylic as a yellow solid, which was converted to 5-methylisochromane-1-carboxamide (*rac*-**4b**) following GP A.

TLC (60% EtOAc/hexanes): R_f = 0.31 [UV, $KMnO_4$].

M.p.: 149-150 °C

^{1}H-NMR (400 MHz, DMSO-d_6, 300 K): δ [ppm] = 7.34 (bs, 1H, NH_2^a), 7.25 – 7.20 (m, 2H, H8, NH_2^b), 7.08 – 7.04 (m, 2H, H6, H7), 5.02 (s, 1H, H1), 4.17 (*virt.* dt, ^2J = 11.4 Hz, $^3J \approx {}^3J$ = 5.1 Hz, 1H, H3^a), 3.80 (ddd, ^2J = 11.4 Hz, ^3J = 8.4 Hz, ^3J = 4.5 Hz, 1H, H3^b), 2.74 (ddd, ^2J = 16.8 Hz, ^3J = 8.4 Hz, ^3J = 5.5 Hz, 1H, H4^b), 2.60 (*virt.* dt, ^2J = 16.8 Hz, $^3J \approx {}^3J$ = 4.4 Hz, 1H, H4^a), 2.20 (s, 3H, CH_3).

^{13}C-NMR (101 MHz, DMSO-d_6, 300 K): δ [ppm] = 172.5 ($CONH_2$), 135.6 (C5), 132.6 (C4a), 131.8 (C8a), 127.9 (C6), 125.1 (C7), 123.0 (C8), 76.1 (C1), 62.6 (C3), 52.2 (C4), 18.5 (CH_3).

IR (film) $\tilde{\nu}_{max}/cm^{-1}$ = 3397 (m, NH), 3175 (m, NH) 2925 (m, CH_{aliph}), 2861 (w, CH_{aliph}), 1658 (s, C=O), 1613 (m, C=C), 1462 (m, CH_{arom}), 1113 (s, C–N), 1101 (s, C–O).

***tert*-Butyl (2-amino-2-oxo-1-phenylethyl)carbamate (*rac*-5a)**

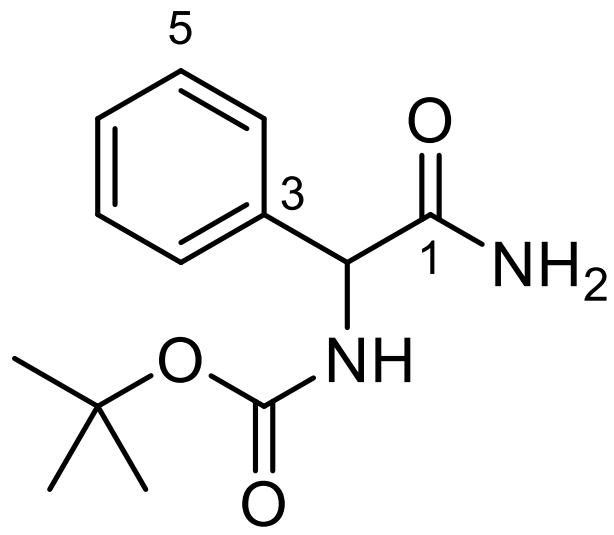

rac-**5a**
$C_{13}H_{18}N_2O_3$
MW = 250.30 gmol^{-1}

Follwoing GP A, *tert*-butyl (2-amino-2-oxo-1-phenylethyl)carbamate (*rac*-**5a**) was obtained from 2-((*tert*-butoxycarbonyl)amino)-2-phenylacetic acid (200 mg, 796 µmol, 1.00 equiv.) as a white solid (142 mg, 567 µmol, 71%).

TLC (50% EtOAc/hexanes): R_f = 0.18 [UV, $KMnO_4$].

M.p.: 125-126 °C

^{1}H-NMR (400 MHz, $CDCl_3$, 300 K): *δ* [ppm] = 7.39 – 7.29 (m, 5H, H3, H4, H5), 5.88 – 5.75 (m, 3H, NH_2, NH), 5.20 (s, 1H, H2), 1.40 (s, 9H, (CH_3).

^{13}C-NMR (101 MHz, $CDCl_3$, 300 K): *δ* [ppm] = 172.6 (C1), 155.2 (CONHO), 138.3 (C3), 129.2 (C5), 128.6 (C6), 127.4 (C4), 80.3 (O*C*(CH_3)$_3$), 58.4 (C2), 28.4 (CH_3).

***tert*-Butyl (2-amino-2-oxo-1-phenylethyl)(methyl)carbamate (*rac*-5b)**

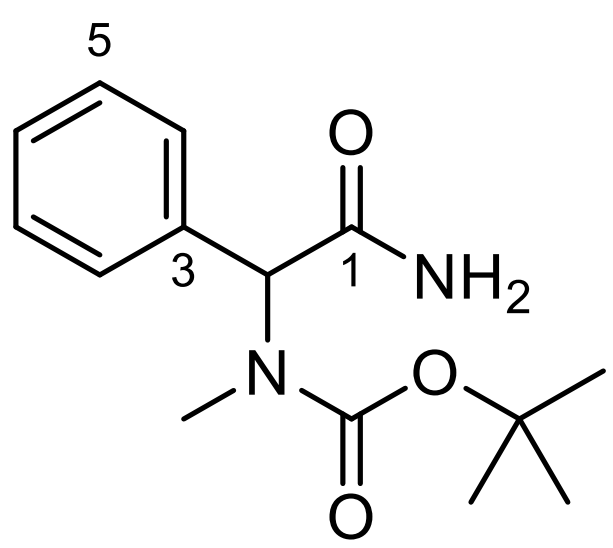

rac-**5b**
$C_{14}H_{20}N_2O_3$
WM = 264.33 gmol^{-1}

Following GP A, *tert*-butyl (2-amino-2-oxo-1-phenylethyl)(methyl)carbamate (*rac*-**5b**) was obtained from 2-((*tert*-butoxycarbonyl)(methyl)amino)-2-phenylacetic acid (80.0 mg, 301 µmol, 1.00 equiv.) as a white solid (61.4 mg, 232 µmol, 77%).

TLC (50% EtOAc/hexanes): R_f = 0.33 [UV, $KMnO_4$].

M.p.: 111-112 °C

^{1}H-NMR (400 MHz, $CDCl_3$, 300 K): *δ* [ppm] = 7.40 – 7.31 (m, 5H, H4, H5, H6), 5.82 – 5.70 (m, 2H, NH_2), 2.74 (3H, H2, NCH_3), 1.49 (s, 9H, (CH_3)$_3$).

^{13}C-NMR (101 MHz, $CDCl_3$, 300 K): *δ* [ppm] = 172.3 (C1), 135.3 (C3), 129.3 (C5), 128.9 (C4), 128.5 (C6), 80.9 (*C*(CH_3)$_3$), 31.9 (NCH_3), 28.5 ((CH_3)$_3$).

HRMS (ESI) *m/z* $[M{-}C_5H_{10}O_2{+}H]^+$: calculated for $[C_9H_{13}N_2O]^+$: 165.1022; found: 165.1016.

IR (film) $\tilde{\nu}_{max}$/cm^{-1} = 3360 (m, NH), 3171 (w, NH), 3002 (w, CH_{arom}), 2980 (m, CH_{aliph}), 2945 (w, CH_{aliph}), 1693 (s, C=O), 1585 (m, C=C), 1458 (s, CH_{arom}), 1145 (s, C–N), 1078 (m, C–O).

The signal for C2 could not be found in the ^{13}C-NMR spectrum.

Benzyl (2-amino-2-oxo-1-phenylethyl)carbamate (*rac*-5c)

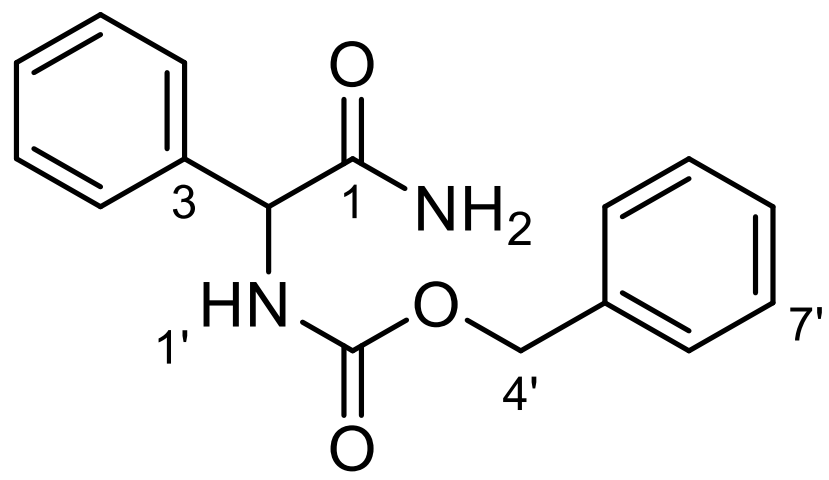

rac-**5c**
$C_{16}H_{16}N_2O_3$
MW = 284.32 gmol^{-1}

Following GP A, benzyl (2-amino-2-oxo-1-phenylethyl)carbamate (*rac*-**5c**) was obtained from 2-(((benzyloxy)carbonyl)amino)-2-phenylacetic acid (330 mg, 1.16 mmol, 1.00 equiv.) as a white solid (221 mg, 777 μmol, 67%).

TLC (70% EtOAc/hexanes): R_f = 0.25 [UV, $KMnO_4$].

M.p.: 170-172 °C

^{1}H-NMR (400 MHz, DMSO-d$_6$, 300 K): δ [ppm] = 7.77 (d, ^3J = 8.5 Hz, 1H, C2NH), 7.59 (bs, 1H, NH_2[a]), 7.45 – 7.25 (m, 11H, H4, H5, H6, H6', H7', H8', NH_2[b]), 5.17 (d, ^3J = 8.5 Hz, 1H, H2), 5.07 – 5.00 (m, 2H, H4').

^{13}C-NMR (101 MHz, DMSO-d$_6$, 300 K): δ [ppm] = 171.6 (C1), 155.5 (NHCOO), 138.8 (C3), 137.0 (C5'), 128.3 (C_{aryl}), 128.2 (C_{aryl}), 127.7 (C_{aryl}), 127.6 (C_{aryl}), 127.5 (C_{aryl}), 127.1 (C_{aryl}), 65.5 (C4'), 58.1 (C2).

HRMS (ESI) *m/z* [M +H]$^+$: calculated for $[C_{16}H_{17}N_2O_3]^+$: 285.1234; found: 285.1222.

IR (film) $\tilde{\nu}_{max}$/cm^{-1} = 3379 (w, NH), 3260 (w, NH), 3198 (w, NH), 3066 (w, CH_{arom}), 3032 (w, CH_{arom}), 2948 (m, CH_{aliph}), 2887 (m, CH_{aliph}), 2801 (w, CH_{aliph}), 1661 (s, C=O), 1552 (m, C=C), 1264 (s, CH_{arom}), 1157 (m, C–N), 1051 (m, C–O).

***tert*-Butyl (2-amino-1-(4-fluorophenyl)-2-oxoethyl)carbamate (*rac*-5d)**

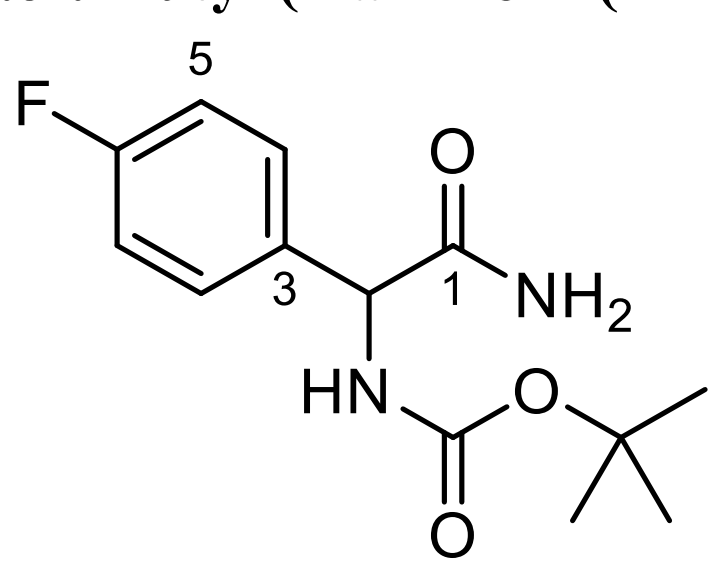

rac-**5d**
$C_{13}H_{17}FN_2O_3$
MW = 268.29 gmol^{-1}

Follwoing GP A, *tert*-butyl (2-amino-1-(4-fluorophenyl)-2-oxoethyl)carbamate (*rac*-**5d**) was obtained from 2-((*tert*-butoxycarbonyl)amino)-2-(4-fluorophenyl)acetic acid (140 mg, 520 μmol, 1.00 equiv.) as a white solid (125 mg, 466 μmol, 90%).

TLC (50% EtOAc/hexanes): R_f = 0.2 [UV, $KMnO_4$].

^{1}H-NMR (400 MHz, $CDCl_3$, 300 K): δ [ppm] = 7.38 – 7.35 (m, 2H, Hx), 7.07 – 7.01 (m, 2H, Hx), 5.93 – 5.74 (m, 3H, NH_2, C2NH), 5.21 (s, 1H, H2), 1.40 (s, 9H, $(CH_3)_3$).

^{13}C-NMR (101 MHz, $CDCl_3$, 300 K): δ [ppm] = 172.3 (C1), 162.9 (d, $^1J_{CF}$ = 247 Hz, C6), 155.3 ($HNCO_2$), 134.2 (d, $^4J_{CF}$ = 3.4 Hz, C3), 129.2 (d, $^3J_{CF}$ = 8.2 Hz, C3), 116.1 (d, $^2J_{CF}$ = 21.7 Hz, C5), 80.5 ($C(CH_3)_3$), 57.6 (C2), 28.4 ($((CH_3)_3)$).

^{19}F-NMR (376 MHz, $CDCl_3$, 300 K): δ [ppm] = −113.3.

HRMS (ESI) *m/z* $[M{-}C_5H_{10}O_2{+}H]^+$: calculated for $[C_8H_{10}FN_2O]^+$: 169.0772; found: 169.0771.

IR (film) $\tilde{\nu}_{max}/cm^{-1}$ = 3401 (w, NH), 3285 (m, NH), 3072 (w, CH_{arom}), 2978 (w, CH_{aliph}), 2950 (m, CH_{aliph}), 2913 (m, CH_{aliph}), 2853 (w, CH_{aliph}), 2831 (w, CH_{aliph}), 1661 (s, C=O), 1550 (m, C=C), 1393 (m, CH_{arom}), 1236 (m, C–F) 1171 (m, C–N), 1054 (s, C–O).

***tert*-Butyl (2-amino-1-(4-chlorophenyl)-2-oxoethyl)carbamate (*rac*-5e)**

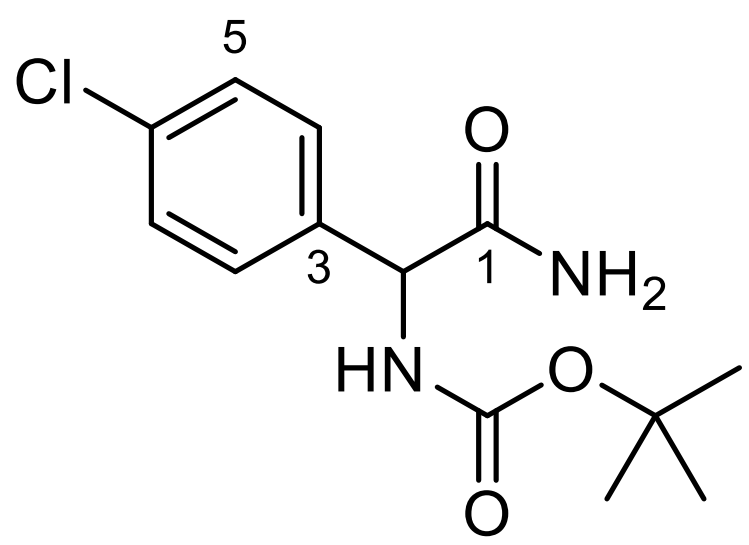

rac-**5e**
$C_{13}H_{17}ClN_2O_3$
MW = 284.74 $gmol^{-1}$

Follwoing GP A, *tert*-butyl (2-amino-1-(4-chlorophenyl)-2-oxoethyl)carbamate (*rac*-**5e**) was obtained from 2-((*tert*-butoxycarbonyl)amino)-2-(4-chlorophenyl)acetic acid (150 mg, 525 μmol, 1.00 equiv.) as a white solid (128 mg, 451 μmol, 86%).

TLC (50% EtOAc/hexanes): R_f = 0.19 [UV, $KMnO_4$].

^{1}H-NMR (400 MHz, $CDCl_3$, 300 K): *δ* [ppm] = 7.33 (m, 4H, H4, H5, H6), 5.91 – 5.71 (m, 3H, NH_2, C2NH), 5.20 (s, 1H, H2), 1.40 (s, 9H, $(CH_3)_3$).

^{13}C-NMR (101 MHz, $CDCl_3$, 300 K): *δ* [ppm] = 172.0 (C1), 136.9 (C6), 134.6 (C3), 129.4 (C5), 128.8 (C4), 80.6 (*C*$(CH_3)_3$), 57.7 (C2) 28.4 ($(CH_3)_3$)

HRMS (ESI) *m/z* $[M{-}C_5H_{10}O_2{+}H]^+$: calculated for $[C_8H_{10}{}^{35}ClN_2O]^+$: 185.0476; found: 185.0476.

IR (film) $\tilde{\nu}_{max}/cm^{-1}$ = 3450 (w, NH), 3295 (m, NH), 3201 (w, CH_{arom}), 2985 (m, CH_{aliph}), 1663 (s, C=O), 1581 (m, C=C), 1495 (m, CH_{arom}), 1168 (m, C–N), 1053 (m, C–O), 1029 (m, C–Cl), 1018 (m, C–Cl).

***tert*-Butyl (1-amino-1-oxo-3-phenylpropan-2-yl)carbamate (*rac*-5f)**

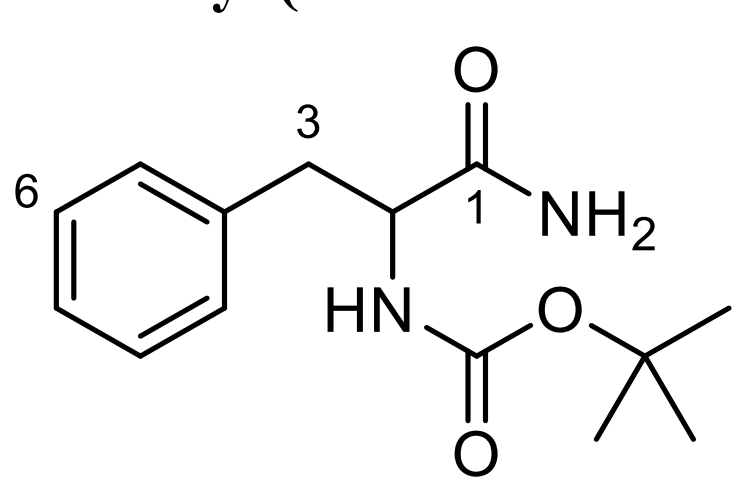

rac-**5f**
$C_{14}H_{20}N_2O_3$
MW = 264.33 $gmol^{-1}$

Follwoing GP A, *tert*-butyl (1-amino-1-oxo-3-phenylpropan-2-yl)carbamate (*rac*-**5f**) was obtained from (*tert*-butoxycarbonyl)phenylalanine (250 mg, 942 μmol, 1.00 equiv.) as a white solid (152 mg, 576 μmol, 61%).

TLC (50% EtOAc/hexanes): R_f = 0.33 [UV, $KMnO_4$].

M.p.: 132-134 °C

^{1}H-NMR (400 MHz, $CDCl_3$, 300 K): *δ* [ppm] = 7.25 – 7.15 (m, 5H, H5, H6, H7), 5.85 (m, 1H, $NH_2{}^a$), 5.52 (bs, 1H, $NH_2{}^b$), 5.05 (bs, 1H, C2NH), 4.32 (s, 1H, H2), 2.13 (m, 2H, H3), 1.34 (s, 9H, $(CH_3)_3$).

^{13}C-NMR (101 MHz, $CDCl_3$, 300 K): δ [ppm] = 172.3 (C1), 155.6 ($HNCO_2$),136.8 (C4), 129.5 (C5), 128.8 (C6), 127.1 (C7), 80.4 (*C*(CH_3)$_3$), 55.6 (C2), 38.6 (C3), 28.4 ((CH_3)$_3$).
HRMS (ESI) *m/z* $[M-C_5H_{10}O_2+H]^+$: calculated for $[C_9H_{13}N_2O]^+$: 165.1022; found: 165.1016.
IR (film) $\tilde{\nu}_{max}$/cm^{-1} = 3394 (m, NH), 3344 (m, NH), 3199 (w, CH_{arom}), 2975 (w, CH_{aliph}), 1657 (s, C=O), 1519 (s, C=C), 1496 (m, CH_{arom}), 1165 (m, C–N), 1049 (m, C–O).

2-Phenylpropanamide (*rac*-5g)

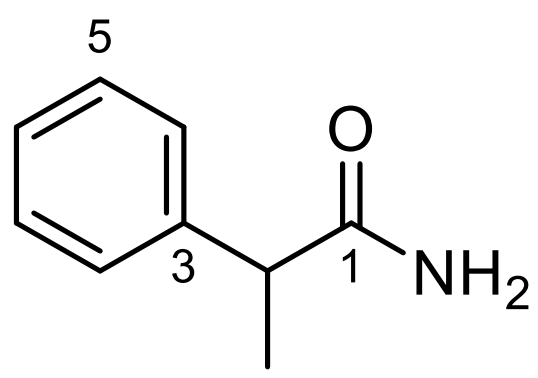

rac-**5g**
$C_9H_{11}NO$
MW = 149.19 gmol^{-1}

Following GP A, 2-phenylpropanamide (*rac*-**5g**) was obtained from 2-phenylpropionic acid (500 mg, 3.33 mmol, 1.00 equiv.) as a white solid (412 mg, 2.76 mmol, 83%).
TLC (60% EtOAc/hexanes): R_f = 0.23 [UV, $KMnO_4$].
^{1}H-NMR (400 MHz, $CDCl_3$, 300 K): δ [ppm] = 7.36 – 7.24 (m, 5H, H4, H5, H6), 5.78 (bs, 1H, NH_2^{a}), 5.37 (bs, 1H, NH_2^{b}), 3.58 (q, ^{3}J = 7.2 Hz, 1H, H2), 1.51 (t, ^{3}J = 7.2 Hz, 3H, CH_3).
^{13}C-NMR (101 MHz, $CDCl_3$, 300 K): δ [ppm] = 176.9 (C1), 141.4 (C3), 129.1 (C5), 127.7 (C4), 127.5 (C6), 46.8 (C2), 18.4 (CH_3).
The spectroscopic data matches the one reported in the literature.[8]

2-Fluoro-2-phenylacetamide (*rac*-5h)

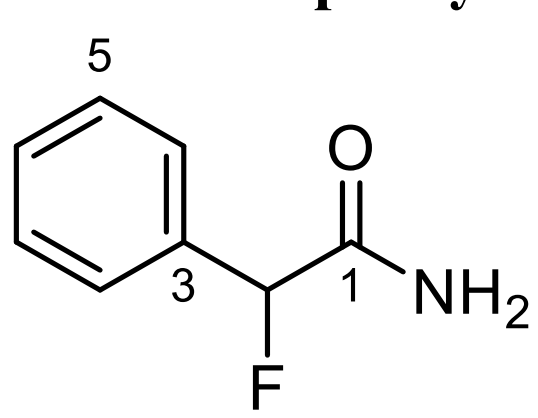

rac-**5h**
C_8H_8FNO
MW = 153.16 gmol^{-1}

Following GP A, 2-fluoro-2-phenylacetamide (*rac*-**5h**) was obtained from 2-fluoro-2-phenylacetic acid (250 mg, 1.62 mmol, 1.00 equiv.) as a white solid (210 mg, 1.37 mmol, 85%).
TLC (50% EtOAc/hexanes): R_f = 0.30 [UV, $KMnO_4$].
M.p.: 140 °C
^{1}H-NMR (400 MHz, $CDCl_3$, 300 K): δ [ppm] = 7.49 – 7.39 (m, 5H, H5, H6, H7), 6.47 – 6.26 (m, 2H, NH_2), 5.76 (d, $^{3}J_{HF}$ = 48.3 Hz, 1H, H2).
^{13}C-NMR (101 MHz, $CDCl_3$, 300 K): δ [ppm] = 171.5 (d, $^{2}J_{CF}$ = 23.4 Hz, C1), 134.6 (d, $^{2}J_{CF}$ = 19.1 Hz, C3), 171.5 (d, $^{2}J_{CF}$ = 23.4 Hz, C1), 129.7 (d, $^{4}J_{CF}$ = 2.6 Hz, C5), 128.9 (C6), 126.8 (d, $^{3}J_{CF}$ = 6.5 Hz, C4), 91.8 (d, $^{1}J_{CF}$ = 188 Hz, C2).
^{19}F-NMR (376 MHz, $CDCl_3$, 300 K): δ [ppm] = −174.7 (d, $^{3}J_{HF}$ = 48.3 Hz).

2-Bromo-2-phenylacetamide (*rac*-5i)

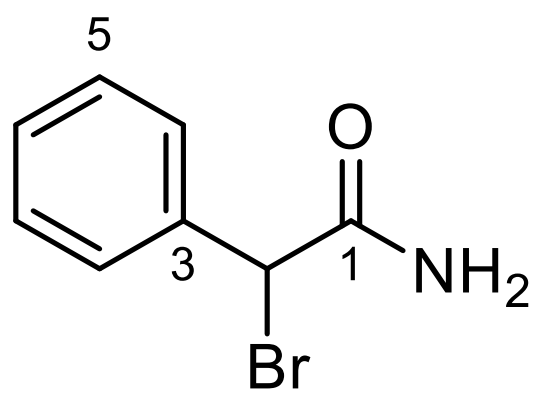

rac-**5i**
C_8H_8BrNO
MW = 153.16 gmol^{-1}

BBr_3 (1.00 M in CH_2Cl_2, 561 mg, 2.24 mL, 2.24 mmol, 3.70 equiv.) was added dropwise to a solution of 2-methoxy-2-phenylacetamide (*rac*-**2a**) (100 mg, 605 µmol, 1.00 equiv.) in CH_2Cl_2 (20 mL) at −78 C. The resulting solution was stirred allowed to warm to r.t. for 14 h. Subsequentyl, water (20 mL) was added and the layers were separated and the aqueous layer was extracted thrice with CH_2Cl_2 (3 × 10 mL). The combined organic layers were washed with brine and dried over Na_2SO_4. The solvents were removed under reduced pressure and the crude product was subjected to FCC (SiO_2, 50 → 100% EtOAc/hexanes) to yield 2-bromo-2-phenylacetamide (*rac*-**5i**) as a white solid (104 mg, 486 µmol, 80%).

TLC (80% EtOAc/hexanes): R_f = 0.50 [UV, $KMnO_4$].

M.p.: 145-146 °C

^{1}H-NMR (400 MHz, $CDCl_3$, 300 K): δ [ppm] = 7.64 – 7.32 (m, 5H, H5, H6, H7), 6.53 (bs, 1H, NH_2^{a}), 5.97 (bs, 1H, NH_2^{b}), 5.42 (s, 1H, H2).

^{13}C-NMR (101 MHz, $CDCl_3$, 300 K): δ [ppm] = 169.7 (C1), 137.2 (C3), 129.4 (C6), 129.2 (C5), 128.5 (C4), 50.6 (C2).

The spectroscopic data matches the one reported in the literature.[9]

3-Methoxy-2-phenylpropanamide (*rac*-5j)

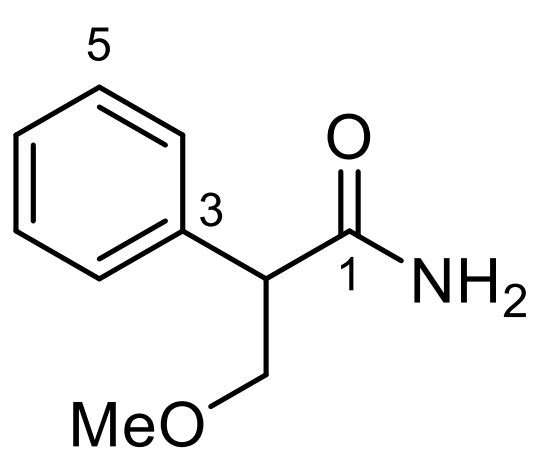

rac-**5j**
$C_{10}H_{13}NO_2$
MW = 179.22 gmol^{-1}

Following GP D, 3-methoxy-2-phenylpropanamide (*rac*-**5j**) was obtained from MeI and methyl-3-hydroxy-2-phenylpropanoate (500 mg, 2.78 mmol, 1.00 equiv.) as a white solid (210 mg, 1.17 mmol, 42% over two steps).

TLC (50% EtOAc/hexanes): R_f = 0.20 [UV, $KMnO_4$].

M.p.: 78-80 °C

^{1}H-NMR (400 MHz, $CDCl_3$, 300 K): δ [ppm] = 7.36 – 7.27 (m, 5H, H4, H5, H6), 5.88 (bs, 1H, NH_2^{a}), 5.55 (bs, 1H, NH_2^{b}), 3.99 – 3.96 (m, 1H, $CH_3OCH_2^{a}$), 3.73 – 3.68 (m, 1H, H2, $CH_3OCH_2^{b}$), 3.38 (s, 3H, OCH_3).

^{13}C-NMR (101 MHz, $CDCl_3$, 300 K): δ [ppm] = 174.3 (C1), 137.1 (C3), 129.0 (C5), 128.4 (C4), 127.8 (C6), 74.1 (CH_3OCH_2), 59.2 (OCH_3), 52.9 (C2).

HRMS (ESI) *m/z* $[M+H]^+$: calculated for $[C_{10}H_{14}NO_2]^+$: 180.1019; found: 180.1018.

IR (film) $\tilde{\nu}_{max}$/cm^{-1} = 3364 (m, NH), 3167 (m, NH), 2931 (m, CH_{aliph}), 2904 (w, CH_{aliph}), 1667 (s, C=O), 1598 (m, C=C), 1455 (m, CH_{arom}), 1400 (s, CH_{aliph}), 1198 (s, C–N), 1116 (s, C–O), 1085 (s, C–O).

2-(Methylthio)-2-phenylacetamide (*rac*-5k)

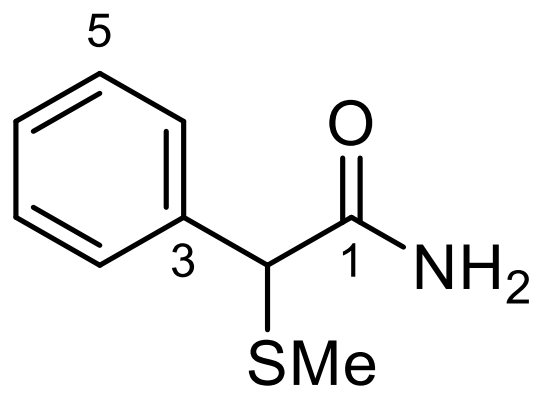

rac-**5k**
$C_9H_{11}NOS$
MW = 181.25 gmol^{-1}

NaSMe (98.2 mg, 1.40 mmol, 3.00 equiv.) was added to a suspension of 2-bromo-2-phenylacetamide (*rac*-**5i**) in MeCN (4.7 mL) and the resulting reaction mixture was stirred at 50 °C for 17 h. Subsequently, the mixture was allowed to cool to r.t. and the solvent was removed under reduced pressure. Water (10 mL) and CH_2Cl_2 (10 mL) were added, the layers were separated, and the aqueous layer was extracted thrice with CH_2Cl_2 (3 × 10 mL). The combined organic layers were washed with brine and dried over Na_2SO_4. The solvents were removed under reduced pressure and the crude product was subjected to FCC (SiO_2, 20 → 100% EtOAc/hexanes) to yield 2-(methylthio)-2-phenylacetamide (*rac*-**5k**) as an off-white solid (41.5 mg, 229 µmol, 49%).

TLC (80% EtOAc/hexanes): R_f = 0.28 [UV, $KMnO_4$].

M.p.: 82-83 °C

^{1}H-NMR (400 MHz, $CDCl_3$, 300 K): δ [ppm] = 7.43 – 7.40 (m, 2H, H4), 7.38 – 7.29 (m, 3H, H5, H6), 6.58 (bs, 1H, NH_2^a), 5.75 (bs, 1H, NH_2^b), 4.48 (s, 1H, H2), 3.10 (s, 3H, CH_3).

^{13}C-NMR (101 MHz, $CDCl_3$, 300 K): δ [ppm] = 172.3 (C1), 136.6 (C3), 129.1 (C5), 128.4 (C6), 128.2 (C4), 56.4 (C2), 16.0 (CH_3).

HRMS (ESI) *m/z* $[M+H]^+$: calculated for $[C_9H_{12}NOS]^+$: 182.0634; found: 182.0628.

IR (film) $\tilde{\nu}_{max}$/cm^{-1} = 3388 (m, NH), 3167 (m, NH), 2914 (m, CH_{aliph}), 1651 (s, C=O), 1583 (m, C=C), 1454 (m, CH_{arom}), 1420 (m, CH_{aliph}), 1199 (m, C–N), 716 (m, C–S).

2-Phenyl-2-(phenylthio)acetamide (*rac*-5l) (MI-VS-24)

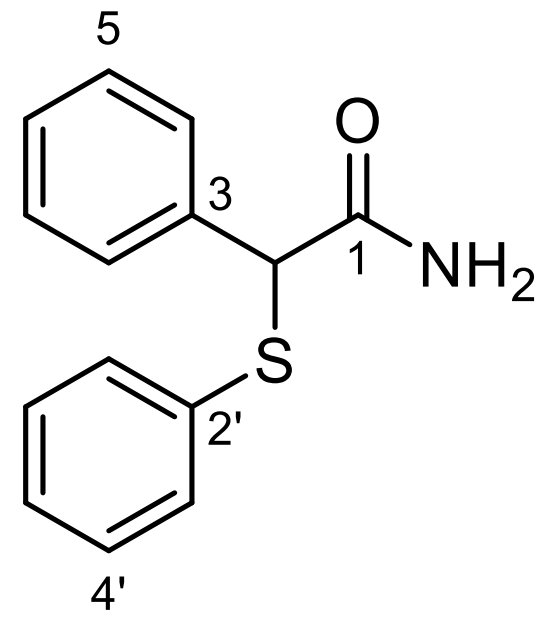

rac-**5l**
$C_{14}H_{13}NOS$
MW = 243.32 gmol^{-1}

Following GP A, 2-phenyl-2-(phenylthio)acetamide (*rac*-**5l**) was obtained from 2-phenyl-2-(phenylthio)acetic acid (200 mg, 819 μmol, 1.00 equiv.) as a white solid (151 mg, 620 μmol, 76%).

TLC (50% EtOAc/hexanes): R_f = 0.50 [UV, $KMnO_4$].

M.p.: 171-172 °C

^{1}H-NMR (400 MHz, DMSO-d_6, 300 K): δ [ppm] = 7.73 (bs, 1H, NH_2[a]), 7.50 – 7.48 (m, 2H, H_{aryl}), 7.35 – 7.25 (m, 7H, H_{aryl}), 7.24 – 7.18 (m, 2H, H H_{aryl}, NH_2[b]), 5.09 (s, 1H, H2).

^{13}C-NMR (101 MHz, DMSO-d_6, 300 K): δ [ppm] = 170.6 (C1), 137.6 (C_{aryl}), 135.1 (C_{aryl}), 129.7 (C_{aryl}), 129.0 (C_{aryl}), 128.3 (C_{aryl}), 128.1 (C_{aryl}), 127.7 (C_{aryl}), 126.7 (C_{aryl}), 55.4 (C2).

HRMS (ESI) *m/z* $[M+H]^+$: calculated for $[C_{14}H_{14}NOS]^+$: 244.0791; found: 244.0786.

IR (film) $\tilde{\nu}_{max}/cm^{-1}$ = 3399 (m, NH), 3166 (m, NH), 3055 (w, CH_{arom}), 3034 (w, CH_{arom}), 2958 (w, CH_{aliph}), 2924 (m, CH_{aliph}), 1651 (s, $C{=}O_{amide}$), 1571 (m, CH_{arom}), 1195 (m, C–N), 718 (m, C–S).

S10. Photochemical Deracemization Reactions

(*R*)-2-Methoxy-2-phenylacetamide (2a)

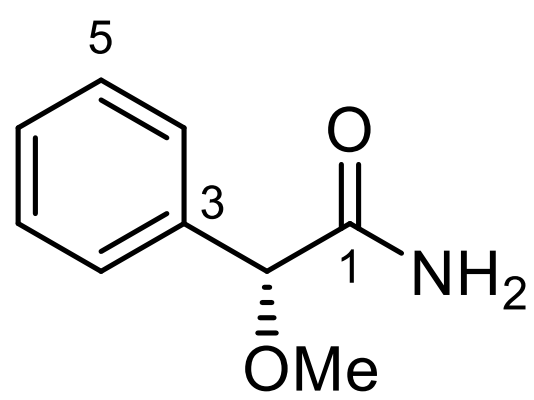

2a
$C_9H_{11}NO_2$
MW = 165.19 gmol^{-1}

According to GP E, a solution of 2-methoxy-2-phenylacetamide (*rac*-**2a**) (4.13 mg, 25.0 µmol, 1.00 equiv.) and enantiomerically pure (+)-benzophenone **1** (755 µg, 1.87 µmol, 7.50 mol%) in 10 mL dry $PhCF_3$ was degassed by being sparged with argon under ultrasonication for 15 minutes. The solution was irradiated at λ = 366 nm at 30 °C for 18 h. Another portion of **1** (755 µg, 1.87 µmol, 7.50 mol%) was added and the solution was again degassed for 15 min and then irradiated at λ = 366 nm for 7 h. After irradiation, the volatile compounds were removed under reduced pressure and the crude reaction mixture was subjected to FCC (SiO_2, 60 → 80% EtOAc/hexanes) to yield (*R*)-2-methoxy-2-phenylacetamide (**2a**) as a white solid (4.0 mg, 24.2 µmol, 97%, 96.5:3.5 e.r.).

Specific Rotation: $[\alpha]_D^{29}$: −110 (*c* = 1.0, $CHCl_3$) [96.5:3.5 e.r.].

Chiral HPLC: 96.5:3.5 e.r. (IC 250 × 4.6 mm, *n*-heptane/^{i}PrOH = 70/30, 1 ml/min, λ = 210 nm); t_R = 17.79 min (major, **2a**), 22.72 min (minor, *ent*-**2a**).

1.00 mmol Scale:

According to GP E, a solution of 2-methoxy-2-phenylacetamide (*rac*-**2a**) (165.2 mg, 1.00 mmol, 1.00 equiv.) and enantiomerically pure (+)-benzophenone **1** (30.2 mg, 75.0 µmol, 7.50 mol%) in 400 mL dry $PhCF_3$ was degassed by being sparged with argon under ultrasonication for 30 minutes. The solution was irradiated at λ = 366 nm at 30 °C for 20 h. Another portion of **1** (30.2 mg, 75.0 µmol, 7.50 mol%) was added and the solution was again degassed for 30 min and then irradiated at λ = 366 nm for 10 h. After irradiation, the volatile compounds were removed under reduced pressure and the crude reaction mixture was subjected to FCC (SiO_2, 60 → 80% EtOAc/hexanes) to yield (*R*)-2-methoxy-2-phenylacetamide (**2a**) as a white solid (156 mg, 944 µmol, 94%, 95.5:4.5 e.r.).

(*R*)-2-Methoxy-2-(*p*-tolyl)acetamide (2b)

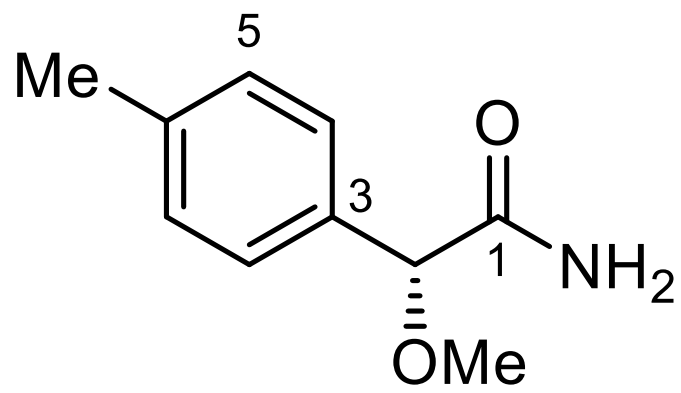

2b

$C_{10}H_{13}NO_2$

MW = 179.22 gmol^{-1}

According to GP E, a solution of 2-methoxy-2-(*p*-tolyl)acetamide (*rac*-**2b**) (4.48 mg, 25.0 µmol, 1.00 equiv.) and enantiomerically pure (+)-benzophenone **1** (755 µg, 1.87 µmol, 7.50 mol%) in 10 mL dry $PhCF_3$ was degassed by being sparged with argon under ultrasonication for 15 minutes. The solution was irradiated at λ = 366 nm at 30 °C for 18 h. Another portion of **1** (755 µg, 1.87 µmol, 7.50 mol%) was added and the solution was again degassed for 15 min and then irradiated at λ = 366 nm for 7 h. After irradiation, the volatile compounds were removed under reduced pressure and the crude reaction mixture was subjected to FCC (SiO_2, 60 → 80% EtOAc/hexanes) to yield (*R*)-2-methoxy-2-(*p*-tolyl)acetamide (**2b**) as a white solid (4.0 mg, 22.3 µmol, 89%, 99.5:0.5 e.r.).

Specific Rotation: $[a]_D^{25}$: −112 (*c* = 1.0, $CHCl_3$) [99.5:0.5 e.r.].

Chiral HPLC: 99.5:0.5 e.r. (AD-H 250 × 4.6 mm, *n*-heptane/^{i}PrOH = 90/10, 1 ml/min, λ = 210 nm); t_R = 12.25 min (major, **2b**), 13.22 min (minor, *ent*-**2b**).

(*R*)-2-(4-Fluorophenyl)-2-methoxyacetamide (2c)

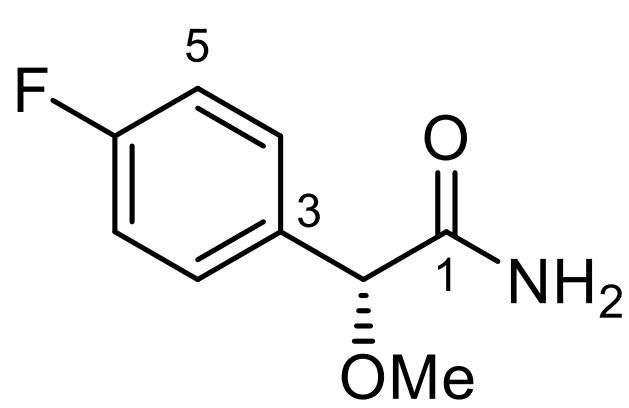

2c

$C_9H_{10}FNO_2$

MW = 183.18 gmol^{-1}

According to GP E, a solution of 2-(4-fluorophenyl)-2-methoxyacetamide (*rac*-**2c**) (4.58 mg, 25.0 µmol, 1.00 equiv.) and enantiomerically pure (+)-benzophenone **1** (755 µg, 1.87 µmol, 7.50 mol%) in 10 mL dry $PhCF_3$ was degassed by being sparged with argon under ultrasonication for 15 minutes. The solution was irradiated at λ = 366 nm at 30 °C for 18 h. Another portion of **1** (755 µg, 1.87 µmol, 7.50 mol%) was added and the solution was again degassed for 15 min and then irradiated at λ = 366 nm for 7 h. After irradiation, the volatile compounds were removed under reduced pressure and the crude reaction mixture was subjected to FCC (SiO_2, 60 → 80% EtOAc/hexanes) to yield (*R*)-2-(4-fluorophenyl)-2-methoxyacetamide (**2c**) as a white solid (4.35 mg, 23.8 µmol, 95%, 93.5:6.5 e.r.).

Specific Rotation: $[a]_D^{25}$: −60 (*c* = 0.5, $CHCl_3$) [93.5:6.5 e.r.].

Chiral HPLC: 93.5:6.5 e.r. (IA 250 × 4.6 mm, *n*-heptane/^{i}PrOH = 90/10, 1 ml/min, λ = 210 nm); t_R = 10.22 min (major, **2c**), 11.68 min (minor, *ent*-**2c**).

(*R*)-2-(4-Chlorophenyl)-2-methoxyacetamide (2d)

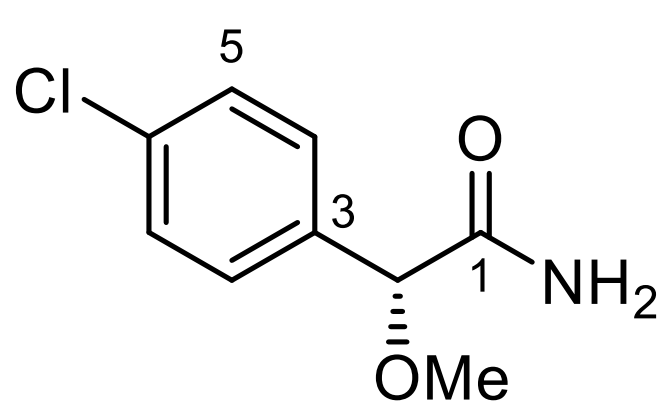

2d
$C_9H_{10}ClNO_2$
MW = 199.63 gmol^{-1}

According to GP E, a solution of 2-(4-chlorophenyl)-2-methoxyacetamide (*rac*-**2d**) (4.99 mg, 25.0 µmol, 1.00 equiv.) and enantiomerically pure (+)-benzophenone **1** (755 µg, 1.87 µmol, 7.50 mol%) in 10 mL dry $PhCF_3$ was degassed by being sparged with argon under ultrasonication for 15 minutes. The solution was irradiated at λ = 366 nm at 30 °C for 18 h. Another portion of **1** (755 µg, 1.87 µmol, 7.50 mol%) was added and the solution was again degassed for 15 min and then irradiated at λ = 366 nm for 7 h. After irradiation, the volatile compounds were removed under reduced pressure and the crude reaction mixture was subjected to FCC (SiO_2, 60 → 80% EtOAc/hexanes) to yield (*R*)-2-(4-chlorophenyl)-2-methoxyacetamide (**2d**) as a white solid (4.8 mg, 24.0 µmol, 96%, 95:5 e.r.).

Specific Rotation: $[\alpha]_D^{29}$: −86 (*c* = 1.0, $CHCl_3$) [95:5 e.r.].

Chiral HPLC: 95:5 e.r. (AD-H 250 × 4.6 mm, *n*-heptane/^{i}PrOH = 90/10, 1 ml/min, λ = 210 nm); t_R = 11.95 min (major, **2d**), 15.55 min (minor, *ent*-**2d**).

(*R*)-2-(4-Bromophenyl)-2-methoxyacetamide (2e)

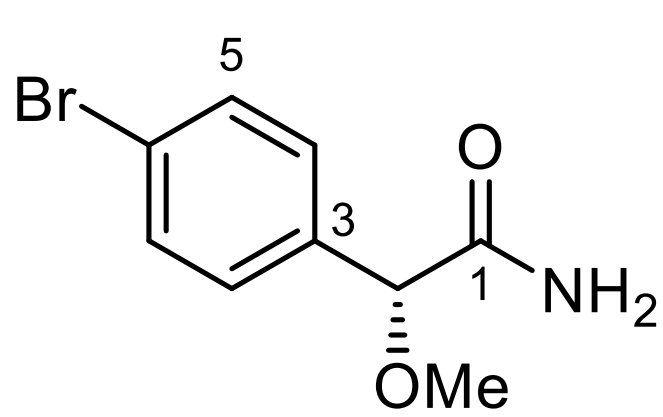

2e
$C_9H_{10}BrNO_2$
MW = 244.09 gmol^{-1}

According to GP E, a solution of 2-(4-bromophenyl)-2-methoxyacetamide (*rac*-**2e**) (6.10 mg, 25.0 µmol, 1.00 equiv.) and enantiomerically pure (+)-benzophenone **1** (755 µg, 1.87 µmol, 7.50 mol%) in 10 mL dry $PhCF_3$ was degassed by being sparged with argon under ultrasonication for 15 minutes. The solution was irradiated at λ = 366 nm at 30 °C for 18 h. Another portion of **1** (755 µg, 1.87 µmol, 7.50 mol%) was added and the solution was again degassed for 15 min and then irradiated at λ = 366 nm for 7 h. After irradiation, the volatile compounds were removed under reduced pressure and the crude reaction mixture was subjected

to FCC (SiO_2, 60 → 80% EtOAc/hexanes) to yield (*R*)-2-(4-bromophenyl)-2-methoxyacetamide (**2e**) as a white solid (5.50 mg, 22.5 μmol, 90%, 94.5:5.5 e.r.).

Specific Rotation: $[\alpha]_D^{29}$: −40 (*c* = 1.0, $CHCl_3$) [94.5:5.5 e.r.].

Chiral HPLC: 94.5:5.5 e.r. (IA 250 × 4.6 mm, *n*-heptane/^{i}PrOH = 90/10, 1 ml/min, λ = 210 nm); t_R = 11.32 min (major, **2e**), 14.33 min (minor, *ent*-**2e**).

(*R*)-2-Methoxy-2-(4-methoxyphenyl)acetamide (**2f**) (BGH-R547-02)

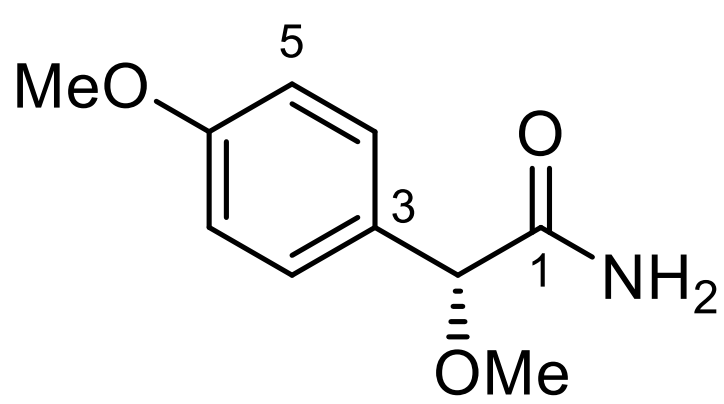

2f

$C_{10}H_{13}NO_3$

MW = 195.22 gmol^{-1}

According to GP E, a of solution 2-methoxy-2-(4-methoxyphenyl)acetamide (*rac*-**2f**) (4.88 mg, 25.0 μmol, 1.00 equiv.) and enantiomerically pure (+)-benzophenone **1** (755 μg, 1.87 μmol, 7.50 mol%) in 10 mL dry $PhCF_3$ was degassed by being sparged with argon under ultrasonication for 15 minutes. The solution was irradiated at λ = 366 nm at 30 °C for 18 h. Another portion of **1** (755 μg, 1.87 μmol, 7.50 mol%) was added and the solution was again degassed for 15 min and then irradiated at λ = 366 nm for 7 h. After irradiation, the volatile compounds were removed under reduced pressure and the crude reaction mixture was subjected to FCC (SiO_2, 60 → 100% EtOAc/hexanes) to yield (*R*)-2-methoxy-2-(4-methoxyphenyl)acetamide (**2f**) as a white solid (4.30 mg, 22.0 μmol, 88%, 88.5:11.5 e.r.).

Specific Rotation: $[\alpha]_D^{29}$: −40 (*c* = 1.0, $CHCl_3$) [88.5:11.5 e.r.].

Chiral HPLC: 88.5:11.5 e.r. (AD-H 250 × 4.6 mm, *n*-heptane/^{i}PrOH = 90/10, 1 ml/min, λ = 210 nm); t_R = 17.76 min (major, **2f**), 21.53 min (minor, *ent*-**2f**).

(*R*)-(2-Methoxy-2-(4-(trifluoromethyl)phenyl)acetamide (2g)

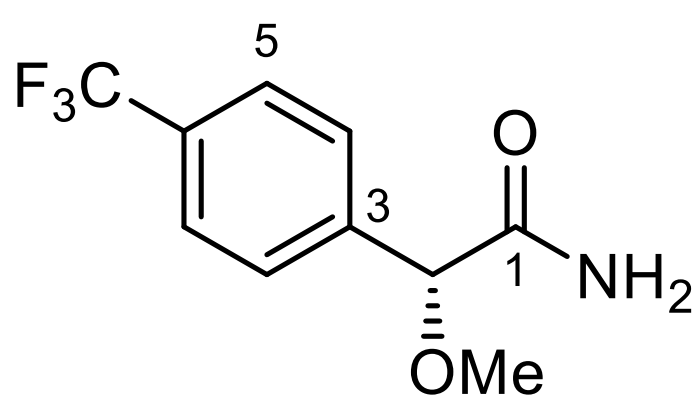

2g
$C_{10}H_{10}F_3NO_3$
MW = 233.19 gmol^{-1}

According to GP E, a solution of 2-methoxy-2-(4-(trifluoromethyl)phenyl)acetamide (*rac*-**2g**) (5.83 mg, 25.0 µmol, 1.00 equiv.) and enantiomerically pure (+)-benzophenone **1** (755 µg, 1.87 µmol, 7.50 mol%) in 10 mL dry $PhCF_3$ was degassed by being sparged with argon under ultrasonication for 15 minutes. The solution was irradiated at λ = 366 nm at 30 °C for 18 h. Another portion of **1** (755 µg, 1.87 µmol, 7.50 mol%) was added and the solution was again degassed for 15 min and then irradiated at λ = 366 nm for 7 h. After irradiation, the volatile compounds were removed under reduced pressure and the crude reaction mixture was subjected to FCC (SiO_2, 40 → 80% EtOAc/hexanes) to yield (*R*)-2-methoxy-2-(4-(trifluoromethyl)phenyl)acetamide (**2g**) as a white solid (4.90 mg, 21.0 µmol, 84%, 92.5:7.5 e.r.).

Specific Rotation: $[\alpha]_D^{27}$: −42 (*c* = 0.5, $CHCl_3$) [92.5:7.5 e.r.].

Chiral HPLC: 92.5:7.5 e.r. (IA 250 × 4.6 mm, *n*-heptane/^{i}PrOH = 90/10, 1 ml/min, λ = 210 nm); t_R = 8.35 min (major, **2g**), 11.99 min (minor, *ent*-**2g**).

(*R*)-2-(2-Fluorophenyl)-2-methoxyacetamide (2h)

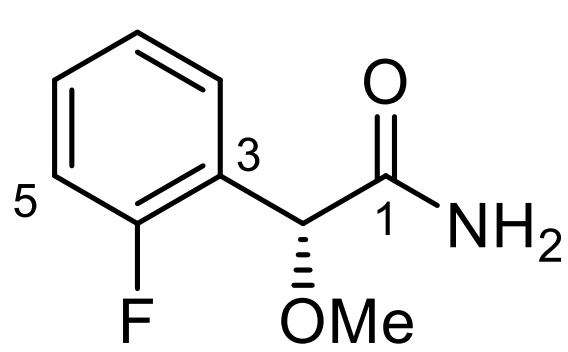

2h
$C_9H_{10}FNO_2$
MW = 183.18 gmol^{-1}

According to GP E, a solution of 2-(2-fluorophenyl)-2-methoxyacetamide (*rac*-**2h**) (4.58 mg, 25.0 µmol, 1.00 equiv.) and enantiomerically pure (+)-benzophenone **1** (755 µg, 1.87 µmol, 7.50 mol%) in 10 mL dry $PhCF_3$ was degassed by being sparged with argon under ultrasonication for 15 minutes. The solution was irradiated at λ = 366 nm at 30 °C for 18 h. Another portion of **1** (755 µg, 1.87 µmol, 7.50 mol%) was added and the solution was again degassed for 15 min and then irradiated at λ = 366 nm for 7 h. After irradiation, the volatile compounds were removed under reduced pressure and the crude reaction mixture was subjected to FCC (SiO_2, 60 → 80% EtOAc/hexanes) to yield (*R*)-2-(2-fluorophenyl)-2-methoxyacetamide (**2h**) as a white solid (4.50 mg, 24.6 µmol, 98%, 81.5:18.5 e.r.).

Specific Rotation: $[\alpha]_D^{29}$: −18 (*c* = 1.0, $CHCl_3$) [81.5:18.5 e.r.].

Chiral HPLC: 81.5:18.5 e.r. (AD-H 250 × 4.6 mm, *n*-heptane/^{i}PrOH = 90/10, 1 ml/min, λ = 210 nm); t_R = 11.39 min (major, **2h**), 12.56 min (minor, *ent*-**2h**).

(*R*)-2-(2-Chlorophenyl)-2-methoxyacetamide (2i)

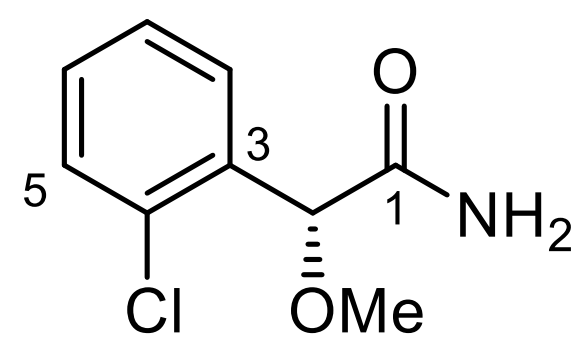

2i
$C_9H_{10}ClNO_2$
MW = 199.63 gmol^{-1}

According to GP E, a solution of 2-(2-chlorophenyl)-2-methoxyacetamide (*rac*-**2i**) (4.99 mg, 25.0 µmol, 1.00 equiv.) and enantiomerically pure (+)-benzophenone **1** (755 µg, 1.87 µmol, 7.50 mol%) in 10 mL dry $PhCF_3$ was degassed by being sparged with argon under ultrasonication for 15 minutes. The solution was irradiated at λ = 366 nm at 30 °C for 18 h. Another portion of **1** (755 µg, 1.87 µmol, 7.50 mol%) was added and the solution was again degassed for 15 min and then irradiated at λ = 366 nm for 7 h. After irradiation, the volatile compounds were removed under reduced pressure and the crude reaction mixture was subjected to FCC (SiO_2, 60 → 100% EtOAc/hexanes) to yield (*R*)-2-(2-chlorophenyl)-2-methoxyacetamide (**2i**) as a white solid (4.50 mg, 22.5 µmol, 90%, 75.5:24.5 e.r.).

Specific Rotation: $[\alpha]_D^{29}$: −16 (*c* = 1.0, $CHCl_3$) [75.5:24.5 e.r.].

Chiral HPLC: 75.5:24.5 e.r. (IC 250 × 4.6 mm, *n*-heptane/^{i}PrOH = 70/30, 1 ml/min, λ = 210 nm); t_R = 16.88 min (major, **2i**), 18.28 min (minor, *ent*-**2i**).

(*R*)-2-(3-Chlorophenyl)-2-methoxyacetamide (2j)

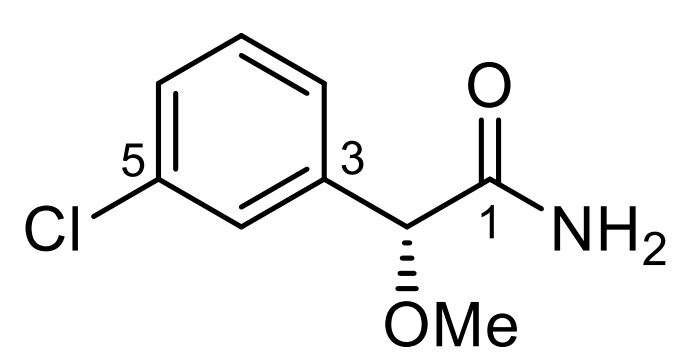

2j
$C_9H_{10}ClNO_2$
MW = 199.63 gmol^{-1}

According to GP E, a solution of 2-(3-chlorophenyl)-2-methoxyacetamide (*rac*-**2j**) (4.99 mg, 25.0 µmol, 1.00 equiv.) and enantiomerically pure (+)-benzophenone **1** (755 µg, 1.87 µmol, 7.50 mol%) in 10 mL dry $PhCF_3$ was degassed by being sparged with argon under ultrasonication for 15 minutes. The solution was irradiated at λ = 366 nm at 30 °C for 18 h. Another portion of **1** (755 µg, 1.87 µmol, 7.50 mol%) was added and the solution was again degassed for 15 min and then irradiated at λ = 366 nm for 7 h. After irradiation, the volatile compounds were removed under reduced pressure and the crude reaction mixture was subjected to FCC (SiO_2, 60 → 100%

EtOAc/hexanes) to yield (*R*)-2-(3-chlorophenyl)-2-methoxyacetamide (**2j**) as a white solid (4.75 mg, 23.8 µmol, 95%, 85.5:14.5 e.r.).

Specific Rotation: $[\alpha]_D^{29}$: −22 (c = 1.0, $CHCl_3$) [85.5:14.5 e.r.].

Chiral HPLC: 85.5:14.5 e.r. (Daicel Chiralpak IC 250 × 4.6 mm, *n*-heptane/^{i}PrOH = 70/30, 1 ml/min, λ = 210 nm); t_R = 10.55 min (major, **2j**), 12.84 min (minor, *ent*-**2j**).

(*R*)-2-(3,4-Dichlorophenyl)-2-methoxyacetamide (2k)

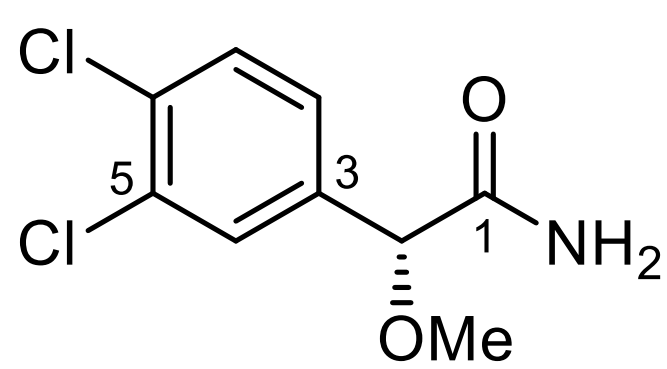

2k
$C_9H_9Cl_2NO_2$
MW = 234.08 gmol^{-1}

According to GP E, a solution of 2-(3,4-dichlorophenyl)-2-methoxyacetamide (*rac*-**2k**) (5.85 mg, 25.0 µmol, 1.00 equiv.) and enantiomerically pure (+)-benzophenone **1** (755 µg, 1.87 µmol, 7.50 mol%) in 10 mL dry $PhCF_3$ was degassed by being sparged with argon under ultrasonication for 15 minutes. The solution was irradiated at λ = 366 nm at 30 °C for 18 h. Another portion of **1** (755 µg, 1.87 µmol, 7.50 mol%) was added and the solution was again degassed for 15 min and then irradiated at λ = 366 nm for 7 h. After irradiation, the volatile compounds were removed under reduced pressure and the crude reaction mixture was subjected to FCC (SiO_2, 60 → 90% EtOAc/hexanes) to yield (*R*)-2-(3,4-dichlorophenyl)-2-methoxyacetamide (**2k**) as a white solid (4.85 mg, 20.7 µmol, 83%, 91.5:8.5 e.r.).

Specific Rotation: $[\alpha]_D^{27}$: −38 (c = 0.5, $CHCl_3$) [91.5:8.5 e.r.].

Chiral HPLC: 91.5:8.5 e.r. (ChiralPak IA 250 × 4.6 mm, *n*-heptane/^{i}PrOH = 90/10, 1 ml/min, λ = 210 nm); t_R = 9.50 min (major, **2k**), 11.87 min (minor, *ent*-**2k**).

(*R*)-2-(3,4-Dimethoxyphenyl)-2-methoxyacetamide (2l)

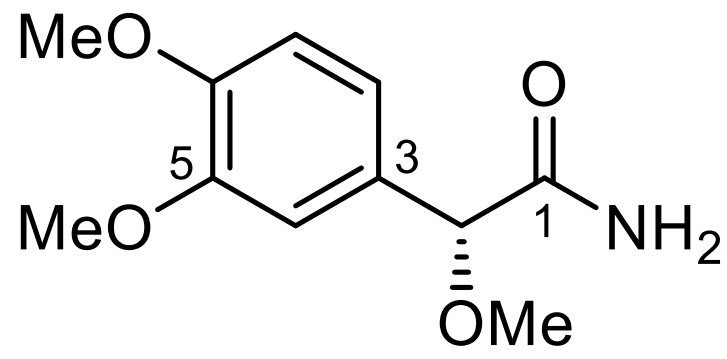

2l
$C_{11}H_{15}NO_4$
MW = 225.24 gmol^{-1}

According to GP E, a solution of 2-(3,4-dimethoxyphenyl)-2-methoxyacetamide (*rac*-**2l**) (5.63 mg, 25.0 µmol, 1.00 equiv.) and enantiomerically pure (+)-benzophenone **1** (755 µg, 1.87 µmol, 7.50 mol%) in 10 mL dry $PhCF_3$ was degassed by being sparged with argon under ultrasonication for 15 minutes. The solution was irradiated at λ = 366 nm at 30 °C for 18 h. Another portion of **1** (755 µg, 1.87 µmol, 7.50 mol%) was added and the solution was again degassed for 15 min and then irradiated at λ = 366 nm for 7 h. After irradiation, the volatile compounds were removed under reduced pressure and the crude reaction mixture was subjected to FCC (SiO_2, 60 → 90% EtOAc/hexanes) to yield (*R*)-2-(3,4-dimethoxyphenyl)-2-methoxyacetamide (**2l**) as a white solid (4.70 mg, 20.9 µmol, 83%, 92.5:7.5 e.r.).

Specific Rotation: $[\alpha]_D^{27}$: −43 (*c* = 0.5, $CHCl_3$) [92.5:7.5 e.r.].

Chiral HPLC: 92.5:7.5 e.r. (ChiralPak AS-H 250 × 4.6 mm, *n*-heptane/^{i}PrOH = 50/50, 1 ml/min, λ = 210 nm); t_R = 9.86 min (minor, *ent*-**2l**), 22.88 min (major, **2l**).

(*R*)-2-Methoxy-2-(thiophen-3-yl)acetamide (2m)

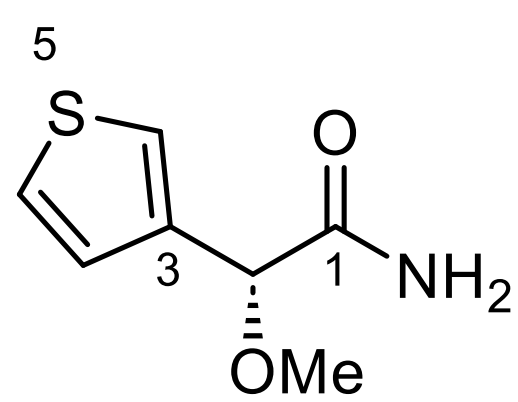

2m
$C_7H_9NO_2S$
MW = 171.21 gmol^{-1}

According to GP E, a solution of 2-methoxy-2-(thiophen-3-yl)acetamide (*rac*-**2m**) (4.28 mg, 25.0 µmol, 1.00 equiv.) and enantiomerically pure (+)-benzophenone **1** (755 µg, 1.87 µmol, 7.50 mol%) in 10 mL dry $PhCF_3$ was degassed by being sparged with argon under ultrasonication for 15 minutes. The solution was irradiated at λ = 366 nm at 30 °C for 18 h. Another portion of **1** (755 µg, 1.87 µmol, 7.50 mol%) was added and the solution was again degassed for 15 min and then irradiated at λ = 366 nm for 7 h. After irradiation, the volatile compounds were removed under reduced pressure and the crude reaction mixture was subjected to FCC (SiO_2, 50 → 100% EtOAc/hexanes) to yield (*R*)-2-methoxy-2-(thiophen-3-yl)acetamide (**2m**) as a white solid (3.49 mg, 20.4 µmol, 82%, 95:5 e.r.).

Specific Rotation: $[\alpha]_D^{27}$: −18 (*c* = 0.5, $CHCl_3$) [95:5 e.r.].

Chiral HPLC: 95:5 e.r. (ChiralPak AD-H 250 × 4.6 mm, *n*-heptane/^{i}PrOH = 90/10, 1 ml/min, λ = 210 nm); t_R = 11.88 min (major, **2m**), 14.78 min (minor, *ent*-**2m**).

(*R*)-2-Methoxy-2-(thiophen-2-yl)acetamide (2n)

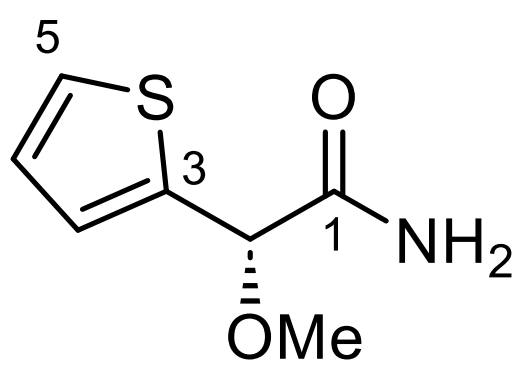

2n

$C_7H_9NO_2S$

MW = 171.21 gmol^{-1}

According to GP E, a solution of 2-methoxy-2-(thiophen-2-yl)acetamide (*rac*-**2n**) (4.28 mg, 25.0 μmol, 1.00 equiv.) and enantiomerically pure (+)-benzophenone **1** (755 μg, 1.87 μmol, 7.50 mol%) in 10 mL dry $PhCF_3$ was degassed by being sparged with argon under ultrasonication for 15 minutes. The solution was irradiated at λ = 366 nm at 30 °C for 18 h. Another portion of **1** (755 μg, 1.87 μmol, 7.50 mol%) was added and the solution was again degassed for 15 min and then irradiated at λ = 366 nm for 7 h. After irradiation, the volatile compounds were removed under reduced pressure and the crude reaction mixture was subjected to FCC (SiO_2, 50 → 100% EtOAc/hexanes) to yield (*R*)-2-methoxy-2-(thiophen-2-yl)acetamide (**2n**) as a white solid (2.85 mg, 16.7 μmol, 67%, 52.5:47.5 e.r.).

Chiral HPLC: 52.5:47.5 e.r. (ChiralPak IC 250 × 4.6 mm, *n*-heptane/^{i}PrOH = 70/30, 1 ml/min, λ = 210 nm); t_R = 19.89 min (major, **2n**), 24.79 min (minor, *ent*-**2n**).

(*R*)-2-(Furan-3-yl)-2-methoxyacetamide (2o)

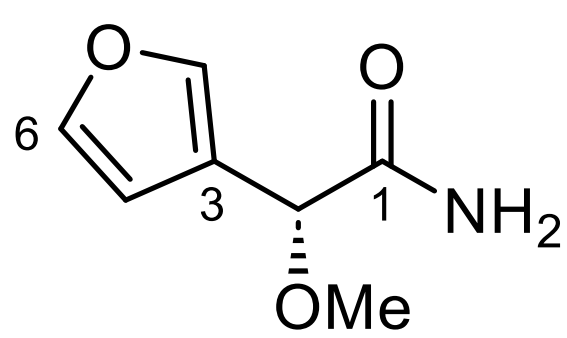

2o

$C_7H_9NO_3$

MW = 155.15 gmol^{-1}

According to GP E, a solution of 2-(furan-3-yl)-2-methoxyacetamide (*rac*-**2o**) (3.88 mg, 25.0 μmol, 1.00 equiv.) and enantiomerically pure (+)-benzophenone **1** (755 μg, 1.87 μmol, 7.50 mol%) in 10 mL dry $PhCF_3$ was degassed by being sparged with argon under ultrasonication for 15 minutes. The solution was irradiated at λ = 366 nm at 30 °C for 18 h. Another portion of **1** (755 μg, 1.87 μmol, 7.50 mol%) was added and the solution was again degassed for 15 min and then irradiated at λ = 366 nm for 7 h. After irradiation, the volatile compounds were removed under reduced pressure and the crude reaction mixture was subjected to FCC (SiO_2, 50 → 100% EtOAc/hexanes) to yield (*R*)- 2-(furan-3-yl)-2-methoxyacetamide (**2o**) as a white solid (3.80 mg, 24.5 μmol, 98%, 55.5:44.5 e.r.).

Chiral HPLC: 55.5:44.5 e.r. (ChiralPak AS-H 250 × 4.6 mm, *n*-heptane/^{i}PrOH = 70/30, 1 ml/min, λ = 210 nm); t_R = 24.36 min (minor, *ent*-**2o**), 27.95 min (major, **2o**).

(*R*)-2-(Furan-2-yl)-2-methoxyacetamide (2p)

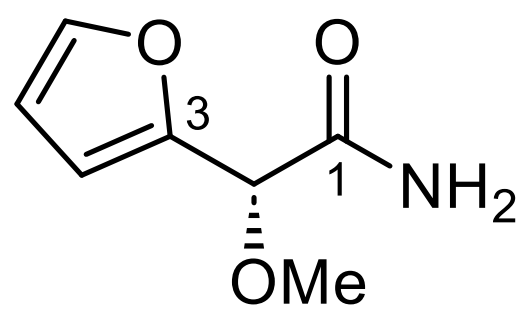

2p
$C_7H_9NO_3$
MW = 155.15 gmol^{-1}

According to GP E, a solution of 2-(furan-2-yl)-2-methoxyacetamide (*rac*-**2p**) (3.88 mg, 25.0 μmol, 1.00 equiv.) and enantiomerically pure (+)-benzophenone **1** (755 μg, 1.87 μmol, 7.50 mol%) in 10 mL dry $PhCF_3$ was degassed by being sparged with argon under ultrasonication for 15 minutes. The solution was irradiated at λ = 366 nm at 30 °C for 18 h. Another portion of **1** (755 μg, 1.87 μmol, 7.50 mol%) was added and the solution was again degassed for 15 min and then irradiated at λ = 366 nm for 7 h. After irradiation, the volatile compounds were removed under reduced pressure and the crude reaction mixture was subjected to FCC (SiO_2, 50 → 100% EtOAc/hexanes) to yield (*R*)- 2-(furan-2-yl)-2-methoxyacetamide (**2p**) as a white solid (3.52 mg, 22.7 μmol, 91%, 51:49 e.r.).

Chiral HPLC: 51:49 e.r. (ChiralPak AD-H 250 × 4.6 mm, *n*-heptane/^{i}PrOH = 90/10, 1 ml/min, λ = 210 nm); t_R = 11.70 min (minor, *ent*-**2p**), 13.14 min (major, **2p**).

(*R*)-2-Methoxy-3-phenylpropanamide (2q)

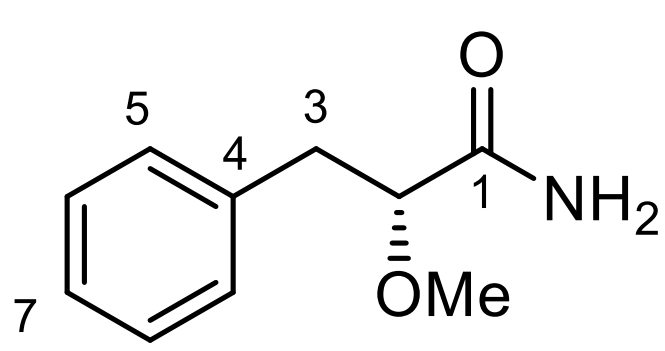

2q
$C_{10}H_{13}NO_2$
MW = 179.22 gmol^{-1}

According to GP E, a solution of 2-methoxy-3-phenylpropanamide (*rac*-**2q**) (4.48 mg, 25.0 μmol, 1.00 equiv.) and enantiomerically pure (+)-benzophenone **1** (755 μg, 1.87 μmol, 7.50 mol%) in 10 mL dry $PhCF_3$ was degassed by being sparged with argon under ultrasonication for 15 minutes. The solution was irradiated at λ = 366 nm at 30 °C for 18 h. Another portion of **1** (755 μg, 1.87 μmol, 7.50 mol%) was added and the solution was again degassed for 15 min and then irradiated at λ = 366 nm for 7 h. After irradiation, the volatile compounds were removed under reduced pressure and the crude reaction mixture was subjected to FCC (SiO_2, 60 → 80% EtOAc/hexanes) to yield (*R*)-2-methoxy-3-phenylpropanamide (**2q**) as a white solid (3.68 mg, 20.5 μmol, 82%, 80:20 e.r.).

Specific Rotation: $[\alpha]_D^{24}$: −28 (c = 0.5, $CHCl_3$) [80:20 e.r.].

Chiral HPLC: 80:20 e.r. (Chiralcel OD-H 250 × 4.6 mm, *n*-heptane/^{i}PrOH = 90/10, 1 ml/min, λ = 210 nm); t_R = 10.23 min (minor, *ent*-**2q**), 13.28 min (major, **2q**).

(*R*)-2-Cyclopropyl-2-methoxyacetamide (2r)

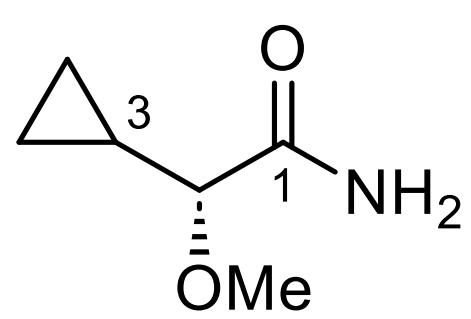

2r
$C_6H_{11}NO_2$
MW = 129.16 gmol^{-1}

According to GP E, a solution of 2-cyclopropyl-2-methoxyacetamide (*rac*-**2r**) (3.23 mg, 25.0 µmol, 1.00 equiv.) and enantiomerically pure (+)-benzophenone **1** (755 µg, 1.87 µmol, 7.50 mol%) in 10 mL dry $PhCF_3$ was degassed by being sparged with argon under ultrasonication for 15 minutes. The solution was irradiated at λ = 366 nm at 30 °C for 18 h. Another portion of **1** (755 µg, 1.87 µmol, 7.50 mol%) was added and the solution was again degassed for 15 min and then irradiated at λ = 366 nm for 7 h. After irradiation, the volatile compounds were removed under reduced pressure and the crude reaction mixture was subjected to FCC (SiO_2, 50 → 100% EtOAc/hexanes) to yield (*R*)-2-cyclopropyl-2-methoxyacetamide (**2r**) as a white solid (2.28 mg, 17.7 µmol, 71%, <51:49 e.r.).

Chiral HPLC: <51:49 e.r. (Chiralcel IC 250 × 4.6 mm, *n*-heptane/^{i}PrOH = 50/50, 1 ml/min, λ = 210 nm); t_R = 7.19 min (major, **2r**), 7.62 min (minor, *ent*-**2r**).

(*R*)-2-Ethoxy-2-phenylacetamide (3a)

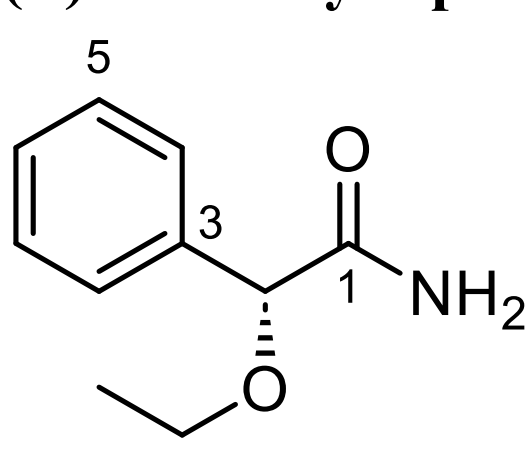

3a
$C_{10}H_{13}NO_2$
MW = 179.22 gmol^{-1}

According to GP E, a solution of 2-ethoxy-3-phenylacetamide (*rac*-**3a**) (4.48 mg, 25.0 µmol, 1.00 equiv.) and enantiomerically pure (+)-benzophenone **1** (755 µg, 1.87 µmol, 7.50 mol%) in 10 mL dry $PhCF_3$ was degassed by being sparged with argon under ultrasonication for 15 minutes. The solution was irradiated at λ = 366 nm at 30 °C for 18 h. Another portion of **1** (755 µg, 1.87 µmol, 7.50 mol%) was added and the solution was again degassed for 15 min and then irradiated at λ = 366 nm for 7 h. After irradiation, the volatile compounds were removed under reduced pressure and the crude reaction mixture was subjected

to FCC (SiO_2, 60 → 80% EtOAc/hexanes) to yield (*R*)-2-ethoxy-3-phenylacetamide (**3a**) as a white solid (3.75 mg, 20.9 µmol, 84%, 98.5:1.5 e.r.).

Specific Rotation: $[\alpha]_D^{27}$: −80 (*c* = 1.0, $CHCl_3$) [98.5:1.5 e.r.].

Chiral HPLC: 98.5:1.5 e.r. (Chiralcel AS-H 250 × 4.6 mm, *n*-heptane/^{i}PrOH = 70/30, 1 ml/min, λ = 210 nm); t_R = 12.86 min (minor, *ent*-**3a**), 23.22 min (major, **3a**).

(*R*)-2-Phenyl-2-propoxyacetamide (3b)

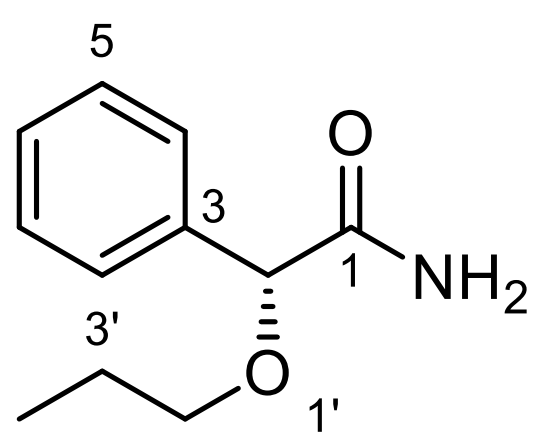

3b
$C_{11}H_{15}NO_2$
MW = 193.25 gmol^{-1}

According to GP E, a solution of 2-phenyl-2-propoxyacetamide (*rac*-**3b**) (4.83 mg, 25.0 µmol, 1.00 equiv.) and enantiomerically pure (+)-benzophenone **1** (755 µg, 1.87 µmol, 7.50 mol%) in 10 mL dry $PhCF_3$ was degassed by being sparged with argon under ultrasonication for 15 minutes. The solution was irradiated at λ = 366 nm at 30 °C for 18 h. Another portion of **1** (755 µg, 1.87 µmol, 7.50 mol%) was added and the solution was again degassed for 15 min and then irradiated at λ = 366 nm for 7 h. After irradiation, the volatile compounds were removed under reduced pressure and the crude reaction mixture was subjected to FCC (SiO_2, 60 → 80% EtOAc/hexanes) to yield (*R*)-2-phenyl-2-propoxyacetamide (**3b**) as a white solid (4.40 mg, 22.8 µmol, 91%, 98:2 e.r.).

Specific Rotation: $[\alpha]_D^{29}$: −76 (*c* = 1.0, $CHCl_3$) [98:2 e.r.].

Chiral HPLC: 98:2 e.r. (Chiralcel OJ-H 250 × 4.6 mm, *n*-heptane/^{i}PrOH = 90/10, 1 ml/min, λ = 210 nm); t_R = 12.21 min (minor, *ent*-**3b**), 16.17 min (major, **3b**).

(*R*)-2-Butoxy-2-phenylacetamide (3c)

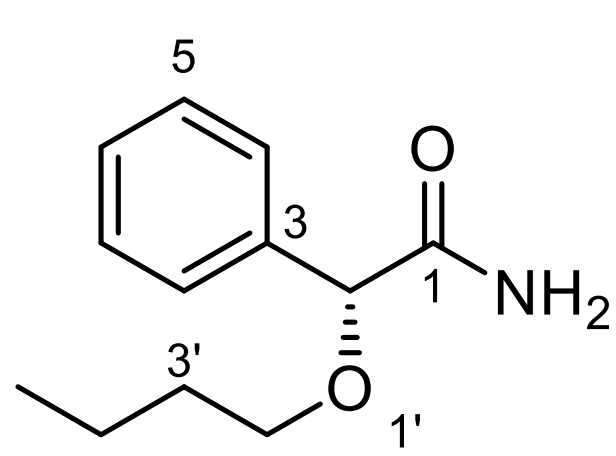

3c
$C_{12}H_{17}NO_2$
MW = 207.27 gmol^{-1}

According to GP E, a solution 2-butoxy-2-phenylacetamide (*rac*-**3c**) (5.18 mg, 25.0 µmol, 1.00 equiv.) and enantiomerically pure (+)-benzophenone **1** (755 µg, 1.87 µmol, 7.50 mol%) in 10 mL dry $PhCF_3$ was degassed by being sparged with argon under ultrasonication for 15 minutes. The solution was irradiated at λ = 366 nm at 30 °C for 18 h. Another portion of **1** (755 µg, 1.87 µmol, 7.50 mol%) was added and the solution was again degassed for 15 min and then irradiated at λ = 366

nm for 7 h. After irradiation, the volatile compounds were removed under reduced pressure and the crude reaction mixture was subjected to FCC (SiO_2, 60 → 80% EtOAc/hexanes) to yield (*R*)-2-butoxy-2-phenylacetamide (**3c**) as a white solid (4.50 mg, 21.7 μmol, 87%, 98.5:1.5 e.r.).

Specific Rotation: $[\alpha]_D^{29}$: −80 (*c* = 1.0, $CHCl_3$) [98.5:1.5 e.r.].

Chiral HPLC: 98.5:1.5 e.r. (Chiralpak IA 250 × 4.6 mm, *n*-heptane/^{i}PrOH = 90/10, 1 ml/min, λ = 210 nm); t_R = 7.38 min (minor, *ent*-**3c**), 8.22 min (major, **3c**).

(*R*)-2-(But-3-en-1-yloxy)-2-phenylacetamide (3d)

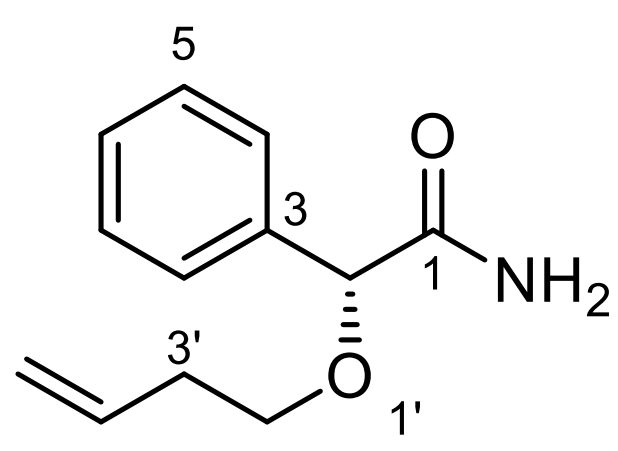

3d
$C_{12}H_{15}NO_2$
MW = 205.26 gmol^{-1}

According to GP E, a solution of 2-(but-3-en-1-yloxy)-2-phenylacetamide (*rac*-**3d**) (5.13 mg, 25.0 μmol, 1.00 equiv.) and enantiomerically pure (+)-benzophenone **1** (755 μg, 1.87 μmol, 7.50 mol%) in 10 mL dry $PhCF_3$ was degassed by being sparged with argon under ultrasonication for 15 minutes. The solution was irradiated at λ = 366 nm at 30 °C for 18 h. Another portion of **1** (755 μg, 1.87 μmol, 7.50 mol%) was added and the solution was again degassed for 15 min and then irradiated at λ = 366 nm for 7 h. After irradiation, the volatile compounds were removed under reduced pressure and the crude reaction mixture was subjected to FCC (SiO_2, 60 → 80% EtOAc/hexanes) to yield (*R*)-2-(but-3-en-1-yloxy)-2-phenylacetamide (**3d**) as a white solid (4.45 mg, 21.7 μmol, 87%, 96:4 e.r.).

Specific Rotation: $[\alpha]_D^{29}$: −98 (*c* = 1.0, $CHCl_3$) [96:4 e.r.].

Chiral HPLC: 96:4 e.r. (Chiralpak AS-H 250 × 4.6 mm, *n*-heptane/^{i}PrOH = 70/30, 1 ml/min, λ = 210 nm); t_R = 15.63 min (minor, *ent*-**3d**), 22.66 min (major, **3d**).

(*R*)-2-Isopropoxy-2-phenylacetamide (3e)

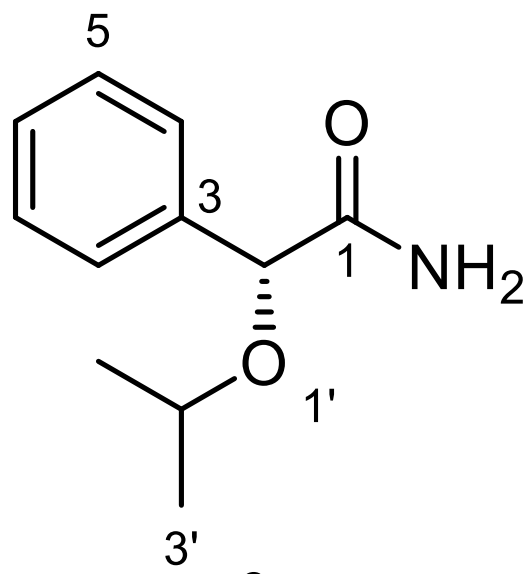

3e

$C_{11}H_{15}NO_2$

MW = 193.25 $gmol^{-1}$

According to GP E, a solution of 2-isopropoxy-2-phenylacetamide (*rac*-**3e**) (4.83 mg, 25.0 µmol, 1.00 equiv.) and enantiomerically pure (+)-benzophenone **1** (755 µg, 1.87 µmol, 7.50 mol%) in 10 mL dry $PhCF_3$ was degassed by being sparged with argon under ultrasonication for 15 minutes. The solution was irradiated at λ = 366 nm at 30 °C for 18 h. Another portion of **1** (755 µg, 1.87 µmol, 7.50 mol%) was added and the solution was again degassed for 15 min and then irradiated at λ = 366 nm for 7 h. After irradiation, the volatile compounds were removed under reduced pressure and the crude reaction mixture was subjected to FCC (SiO_2, 60 → 80% EtOAc/hexanes) to yield (*R*)-2-isopropoxy-2-phenylacetamide (**3e**) as a white solid (4.10 mg, 21.2 µmol, 85%, 98:2 e.r.).

Specific Rotation: $[\alpha]_D^{27}$: −78 (*c* = 1.0, $CHCl_3$) [98:2 e.r.].

Chiral HPLC: 98:2 e.r. (Chiralpak IC 250 × 4.6 mm, *n*-heptane/^{i}PrOH = 70/30, 1 ml/min, λ = 210 nm); t_R = 12.88 min (major, **3e**), 22.98 min (minor, *ent*-**3e**).

(*R*)-2-(2-Fluoroethoxy)-2-phenylacetamide (3f)

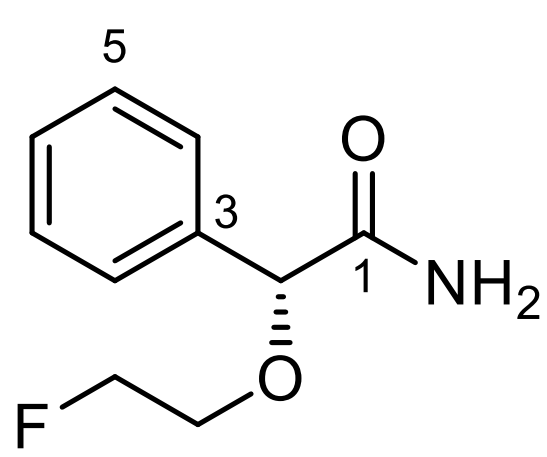

3f

$C_{10}H_{12}FNO_2$

MW = 197.21 $gmol^{-1}$

According to GP E, a solution of 2-(2-fluoroethoxy)-2-phenylacetamide (*rac*-**3f**) (4.93 mg, 25.0 µmol, 1.00 equiv.) and enantiomerically pure (+)-benzophenone **1** (755 µg, 1.87 µmol, 7.50 mol%) in 10 mL dry $PhCF_3$ was degassed by being sparged with argon under ultrasonication for 15 minutes. The solution was irradiated at λ = 366 nm at 30 °C for 18 h. Another portion of **1** (755 µg, 1.87 µmol, 7.50 mol%) was added and the solution was again degassed for 15 min and then irradiated at λ = 366 nm for 7 h. After irradiation, the volatile compounds were removed under reduced pressure and the crude reaction mixture was subjected to FCC (SiO_2, 50 → 70% EtOAc/hexanes) to yield (*R*)-2-(2-fluoroethoxy)-2-phenylacetamide (**3f**) as a white solid (4.20 mg, 21.3 µmol, 85%, 90.5:9.5 e.r.).

Specific Rotation: $[\alpha]_D^{24}$: −61 (*c* = 1.0, $CHCl_3$) [90.5:9.5 e.r.].

Chiral HPLC: 90.5:9.5 e.r. (Chiralpak IC 250 × 4.6 mm, *n*-heptane/^{i}PrOH = 50/50, 1 ml/min, λ = 210 nm); t_R = 14.63 min (minor, *ent*-**3f**), 22.56 min (major, **3f**).

(*R*)-2-Phenyl-2-(3,3,3-trifluoropropoxy)acetamide (3g)

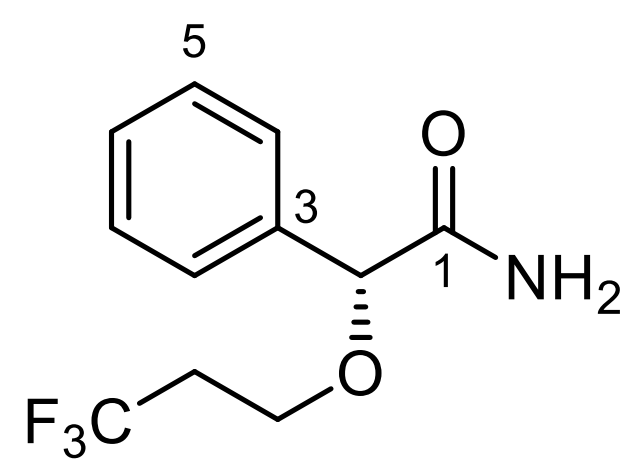

3g

$C_{11}H_{12}F_3NO_2$

MW = 247.22 gmol^{-1}

According to GP E, a solution of 2-phenyl-2-(3,3,3-trifluoropropoxy)acetamide (*rac*-**3g**) (6.18 mg, 25.0 µmol, 1.00 equiv.) and enantiomerically pure (+)-benzophenone **1** (755 µg, 1.87 µmol, 7.50 mol%) in 10 mL dry $PhCF_3$ was degassed by being sparged with argon under ultrasonication for 15 minutes. The solution was irradiated at λ = 366 nm at 30 °C for 18 h. Another portion of **1** (755 µg, 1.87 µmol, 7.50 mol%) was added and the solution was again degassed for 15 min and then irradiated at λ = 366 nm for 7 h. After irradiation, the volatile compounds were removed under reduced pressure and the crude reaction mixture was subjected to FCC (SiO_2, 40 → 70% EtOAc/hexanes) to yield (*R*)-2-phenyl-2-(3,3,3-trifluoropropoxy)acetamide (**3g**) as a white solid (5.40 mg, 21.8 µmol, 87%, 94.5:5.5 e.r.).

Specific Rotation: $[\alpha]_D^{24}$: −55 (*c* = 1.0, $CHCl_3$) [94.5:5.5 e.r.].

Chiral HPLC: 94.5:5.5 e.r. (Chiralpak IC 250 × 4.6 mm, *n*-heptane/^{i}PrOH = 70/30, 1 ml/min, λ = 210 nm); t_R = 14.17 min (minor, *ent*-**3g**), 22.48 min (major, **3g**).

(*R*)-2-(Allyloxy)-2-phenylacetamide (3h)

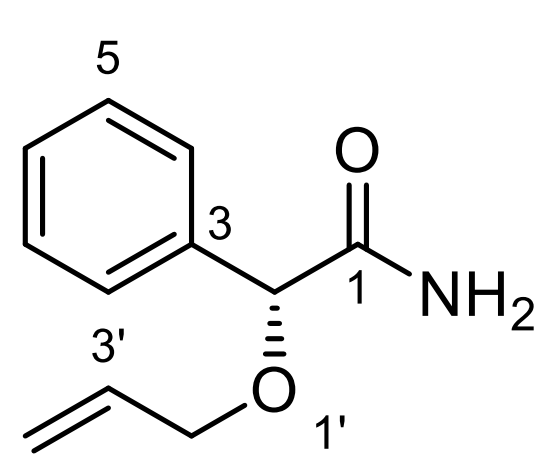

3h

$C_{11}H_{13}NO_2$

MW = 191.23 gmol^{-1}

According to GP E, a solution of 2-(allyloxy)-2-phenylacetamide (*rac*-**3h**) (4.78 mg, 25.0 µmol, 1.00 equiv.) and enantiomerically pure (+)-benzophenone **1** (755 µg, 1.87 µmol, 7.50 mol%) in 10 mL dry $PhCF_3$ was degassed by being sparged with argon under ultrasonication for 15 minutes. The solution was irradiated at λ = 366 nm at 30 °C for 18 h. Another portion of **1** (755 µg, 1.87 µmol, 7.50 mol%) was added and the solution was again degassed for 15 min and then irradiated at λ = 366 nm for 7 h. After irradiation, the volatile compounds were removed under reduced pressure and the crude reaction mixture was subjected to FCC (SiO_2, 50 → 80% EtOAc/hexanes) to yield (*R*)-2-(allyloxy)-2-phenylacetamide (**3h**) as a white solid (4.10 mg, 21.4 µmol, 86%, 89:11 e.r.).

Specific Rotation: $[\alpha]_D^{29}$: −70 (*c* = 1.0, $CHCl_3$) [89:11 e.r.].

Chiral HPLC: 89:11 e.r. (Chiralpak IC 250 × 4.6 mm, *n*-heptane/^{i}PrOH = 70/30, 1 ml/min, λ = 210 nm); t_R = 18.14 min (major, **3h**), 23.17 min (minor, *ent*-**3h**).

(*R*)-2-(But-3-yn-1-yloxy)-2-phenylacetamide (**3i**)

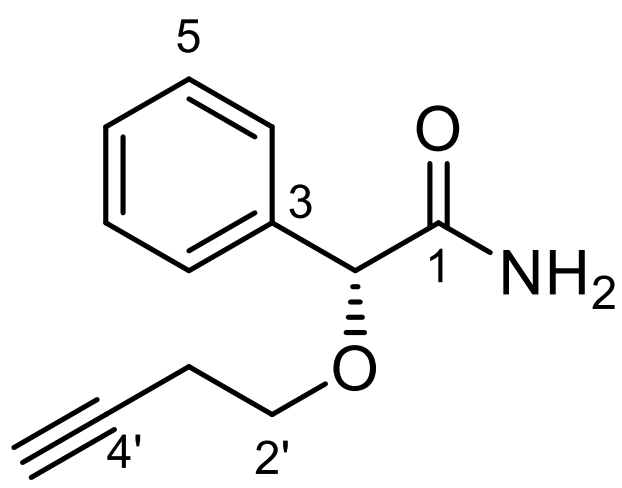

3i
$C_{12}H_{13}NO_2$
MW = 203.24 gmol^{-1}

According to GP E, a solution of 2-(but-3-yn-1-yloxy)-2-phenylacetamide (*rac*-**3i**) (5.08 mg, 25.0 µmol, 1.00 equiv.) and enantiomerically pure (+)-benzophenone **1** (755 µg, 1.87 µmol, 7.50 mol%) in 10 mL dry $PhCF_3$ was degassed by being sparged with argon under ultrasonication for 15 minutes. The solution was irradiated at λ = 366 nm at 30 °C for 18 h. Another portion of **1** (755 µg, 1.87 µmol, 7.50 mol%) was added and the solution was again degassed for 15 min and then irradiated at λ = 366 nm for 7 h. After irradiation, the volatile compounds were removed under reduced pressure and the crude reaction mixture was subjected to FCC (SiO_2, 50 → 80% EtOAc/hexanes) to yield (*R*)-2-(but-3-yn-1-yloxy)-2-phenylacetamide (**3i**) as a white solid (4.10 mg, 20.2 µmol, 81%, 88.5:11.5 e.r.).

Specific Rotation: $[\alpha]_D^{27}$: −40 (*c* = 0.5, $CHCl_3$) [88.5:11.5 e.r.].

Chiral HPLC: 88.5:11.5 e.r. (Chiralpak IC 250 × 4.6 mm, *n*-heptane/^{i}PrOH = 90/10, 1 ml/min, λ = 210 nm); t_R = 22.95 min (major, **3i**), 28.23 min (minor, *ent*-**3i**).

(*R*)-2-Phenoxy-2-phenylacetamide (**3j**)

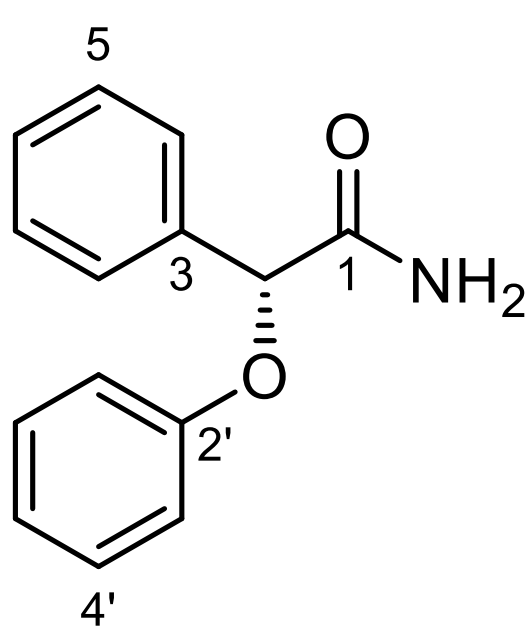

3j
$C_{14}H_{13}NO_2$
MW = 227.26 gmol^{-1}

According to GP E, a solution of 2-phenoxy-2-phenylacetamide (*rac*-**3j**) (5.68 mg, 25.0 µmol, 1.00 equiv.) and enantiomerically pure (+)-benzophenone **1** (755 µg, 1.87 µmol, 7.50 mol%) in 10 mL dry $PhCF_3$ was degassed by being sparged with argon under ultrasonication for 15 minutes. The solution was irradiated at λ = 366 nm at 30 °C for 18 h. Another portion of **1** (755 µg, 1.87 µmol, 7.50 mol%) was added and the solution was again degassed for 15 min and then irradiated at λ = 366 nm for 7 h. After irradiation, the volatile compounds were removed under reduced pressure and the crude reaction mixture was subjected to FCC (SiO_2, 50 → 80% EtOAc/hexanes) to yield (*R*)-2-phenoxy-2-phenylacetamide (**3j**) as a white solid (5.35 mg, 23.5 µmol, 94%, 72:28 e.r.).

Specific Rotation: $[\alpha]_D^{27}$: −17 (*c* = 0.5, $CHCl_3$) [72:28 e.r.].

Chiral HPLC: 72:28 e.r. (Chiralpak IA 250 × 4.6 mm, *n*-heptane/^{i}PrOH = 90/10, 1 ml/min, λ = 210 nm); t_R = 12.44 min (minor, *ent*-**3j**), 14.62 min (major, **3j**).

(*R*)-2-(Benzyloxy)-2-phenylacetamide (3k)

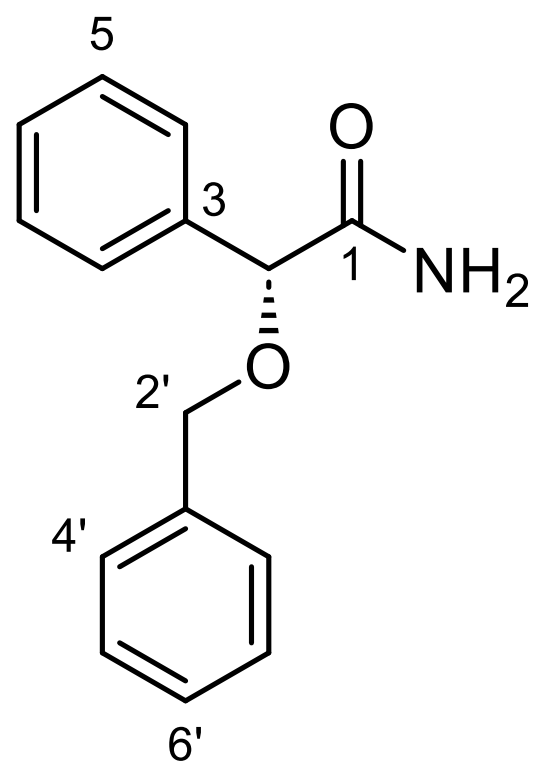

3k
$C_{15}H_{15}NO_2$
MW = 241.29 gmol^{-1}

According to GP E, a solution of 2-(benzyloxy)-2-phenylacetamide (*rac*-**3k**) (6.03 mg, 25.0 µmol, 1.00 equiv.) and enantiomerically pure (+)-benzophenone **1** (755 µg, 1.87 µmol, 7.50 mol%) in 10 mL dry $PhCF_3$ was degassed by being sparged with argon under ultrasonication for 15 minutes. The solution was irradiated at λ = 366 nm at 30 °C for 18 h. Another portion of **1** (755 µg, 1.87 µmol, 7.50 mol%) was added and the solution was again degassed for 15 min and then irradiated at λ = 366 nm for 7 h. After irradiation, the volatile compounds were removed under reduced pressure and the crude reaction mixture was subjected to FCC (SiO_2, 50 → 100% EtOAc/hexanes) to yield (*R*)-2-(benzyloxy)-2-phenylacetamide (**3k**) as a white solid (4.35 mg, 18.0 µmol, 72%, 90:10 e.r.).

Specific Rotation: $[\alpha]_D^{27}$: −53 (*c* = 0.5, $CHCl_3$) [90:10 e.r.].

Chiral HPLC: 90:10 e.r. (Chiralpak IC 250 × 4.6 mm, *n*-heptane/^{i}PrOH = 50/50, 1 ml/min, λ = 210 nm); t_R = 17.80 min (minor, *ent*-**3k**), 30.09 min (major, **3k**).

(*R*)-2-Phenyl-2-((triethylsilyl)oxy)acetamide (3l)

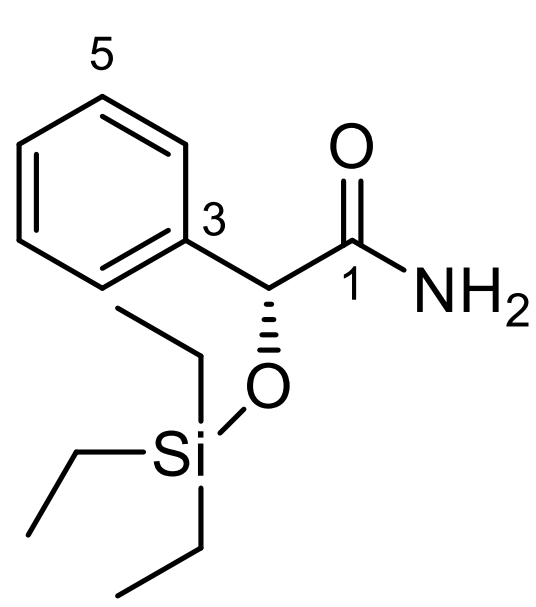

3l
$C_{14}H_{23}NO_2Si$
MW = 265.43 gmol^{-1}

According to GP E, a solution of 2-phenyl-2-((triethylsilyl)oxy)acetamide (*rac*-**3l**) (6.64 mg, 25.0 µmol, 1.00 equiv.) and enantiomerically pure (+)-benzophenone **1** (755 µg, 1.87 µmol, 7.50 mol%) in 10 mL dry $PhCF_3$ was degassed by being sparged with argon under ultrasonication for 15 minutes. The solution was irradiated at λ = 366 nm at 30 °C for 18 h. Another portion of **1** (755 µg, 1.87 µmol, 7.50 mol%) was added and the solution was again degassed for 15 min and then irradiated at λ = 366 nm for 7 h. After irradiation, the volatile compounds were removed under reduced pressure and the crude reaction mixture was subjected to FCC (SiO_2, 50 → 80% EtOAc/hexanes) to yield

(*R*)-2-phenyl-2-((triethylsilyl)oxy)acetamide (**3l**) as a white solid (6.25 mg, 23.6 µmol, 94%, 89.5:10.5 e.r.).

Specific Rotation: $[\alpha]_D^{27}$: −60 (*c* = 0.5, $CHCl_3$) [89.5:10.5 e.r.].

Chiral HPLC: 89.5:10.5 e.r. (Chiralpak IC 250 × 4.6 mm, *n*-heptane/^{i}PrOH = 70/30, 1 ml/min, λ = 210 nm); t_R = 8.97 min (major, **3l**), 23.77 min (minor, *ent*-**3l**).

2-Amino-2-oxo-1-phenylethyl 4-methylbenzenesulfonate (3m)

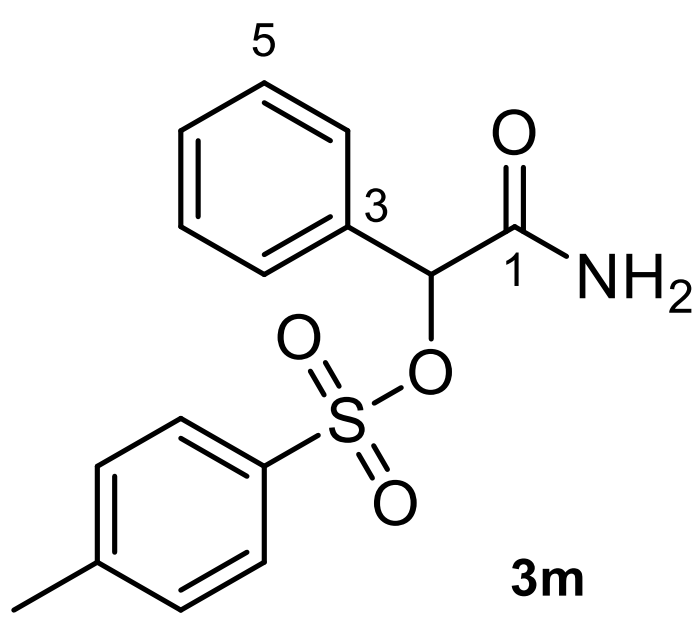

3m
$C_{15}H_{15}NO_4S$
MW = 305.35 gmol^{-1}

According to GP E, a solution of 2-amino-2-oxo-1-phenylethyl 4-methylbenzenesulfonate (*rac*-**3m**) (7.63 mg, 25.0 µmol, 1.00 equiv.) and enantiomerically pure (+)-benzophenone **1** (755 µg, 1.87 µmol, 7.50 mol%) in 10 mL dry $PhCF_3$ was degassed by being sparged with argon under ultrasonication for 15 minutes. The solution was irradiated at λ = 366 nm at 30 °C for 18 h. Another portion of **1** (755 µg, 1.87 µmol, 7.50 mol%) was added and the solution was again degassed for 15 min and then irradiated at λ = 366 nm for 7 h. After irradiation, the volatile compounds were removed under reduced pressure and the crude reaction mixture was subjected to FCC (SiO_2, 50 → 100% EtOAc/hexanes) to yield 2-amino-2-oxo-1-phenylethyl 4-methylbenzenesulfonate (**3m**) as a white solid (7.25 mg, 23.7 µmol, 95%, 52:48 e.r.).

Chiral HPLC: 52:48 e.r. (Chiralpak IC 250 × 4.6 mm, *n*-heptane/^{i}PrOH = 70/30, 1 ml/min, λ = 210 nm); t_R = 8.96 min (major, **3m**), 10.74 min (minor, *ent*-**3m**).

2-Amino-2-oxo-1-phenylethyl acetate (3n)

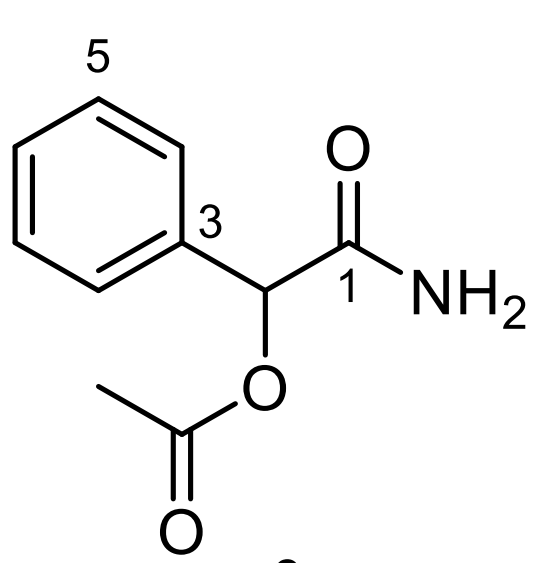

3n
$C_{10}H_{11}NO_3$
MW = 193.20 gmol^{-1}

According to GP E, a solution of 2-amino-2-oxo-1-phenylethyl acetate (*rac*-**3n**) (4.83 mg, 25.0 µmol, 1.00 equiv.) and enantiomerically pure (+)-benzophenone **1** (755 µg, 1.87 µmol, 7.50 mol%) in 10 mL dry $PhCF_3$ was degassed by being sparged with argon under ultrasonication for 15 minutes. The solution was irradiated at λ = 366 nm at 30 °C for 18 h. Another portion of **1** (755 µg, 1.87 µmol, 7.50 mol%) was added and the solution was again degassed for 15 min and then irradiated at λ = 366 nm

for 7 h. After irradiation, the volatile compounds were removed under reduced pressure and the crude reaction mixture was subjected to FCC (SiO_2, 50 → 100% EtOAc/hexanes) to yield 2-amino-2-oxo-1-phenylethyl acetate (**3n**) as a white solid (4.00 mg, 20.7 µmol, 83%, <51:49 e.r.).

Chiral HPLC: <51:49 e.r. (Chiralpak AD-H 250 × 4.6 mm, *n*-heptane/^{i}PrOH = 90/10, 1 ml/min, λ = 210 nm); t_R = 13.42 min and 15.08 min.

(*R*)-2-((2-Methoxyethoxy)methoxy)-2-phenylacetamide (3o)

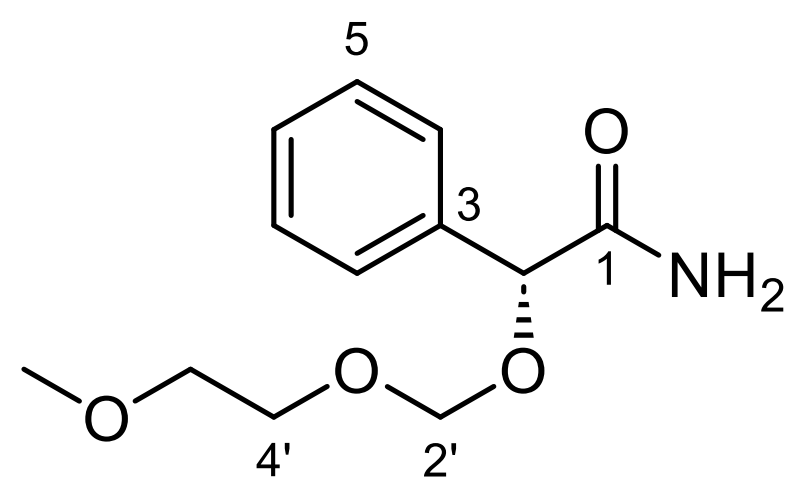

3o
$C_{12}H_{17}NO_4$
MW = 239.27 gmol^{-1}

According to GP E, a solution of 2-((2-methoxyethoxy)methoxy)-2-phenylacetamide (*rac*-**3o**) (5.98 mg, 25.0 µmol, 1.00 equiv.) and enantiomerically pure (+)-benzophenone **1** (755 µg, 1.87 µmol, 7.50 mol%) in 10 mL dry $PhCF_3$ was degassed by being sparged with argon under ultrasonication for 15 minutes. The solution was irradiated at λ = 366 nm at 30 °C for 18 h. Another portion of **1** (755 µg, 1.87 µmol, 7.50 mol%) was added and the solution was again degassed for 15 min and then irradiated at λ = 366 nm for 7 h. After irradiation, the volatile compounds were removed under reduced pressure and the crude reaction mixture was subjected to FCC (SiO_2, 50 → 100% EtOAc/hexanes) to yield (*R*)-2-((2-methoxyethoxy)methoxy)-2-phenylacetamide (**3o**) as a white solid (5.10 mg, 21.3 µmol, 85%, 78:22 e.r.).

Specific Rotation: $[\alpha]_D^{27}$: −68 (*c* = 0.5, $CHCl_3$) [78:22 e.r.].

Chiral HPLC: 78:22 e.r. (Chiralpak IA 250 × 4.6 mm, *n*-heptane/^{i}PrOH = 90/10, 1 ml/min, λ = 210 nm); t_R = 17.69 min (major, **3o**), 20.29 min (minor, *ent*-**3o**).

(*R*)-Isochromane-1-carboxamide (4a)

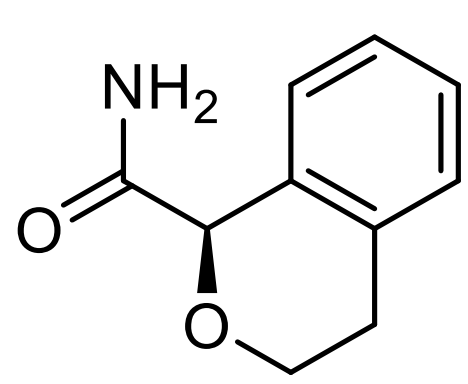

4a
$C_{10}H_{11}NO_2$
MW = 177.20 gmol^{-1}

A solution of isochromane-1-carboxamide (*rac*-**4a**) (4.43 mg, 25.0 µmol, 1.00 equiv.) and enantiomerically pure (+)-benzophenone **1** (1.01 mg, 2.50 µmol, 10.0 mol%) in 10 mL dry $PhCF_3$ was degassed by being sparged with argon under ultrasonication for 15 minutes. The solution was irradiated at λ = 366 nm at 30 °C for 18 h. After irradiation, the volatile compounds were removed under reduced pressure and the crude reaction mixture was

subjected to FCC (SiO_2, 50 → 100% EtOAc/hexanes) to yield (*R*)-isochromane-1-carboxamide (**4a**) as a white solid (3.75 mg, 21.2 μmol, 85%, 97.5:2.5 e.r.).

TLC (50% EtOAc/hexanes): R_f = 0.35 [UV, $KMnO_4$].

Specific Rotation: $[\alpha]_D^{27}$: −53 (*c* = 0.5, $CHCl_3$) [97.5:2.5 e.r.].

Chiral HPLC: 97.5:2.5 e.r. (Daicel Chiralpak, AS-H 250 × 4.6 mm, *n*-heptane/^{i}PrOH = 70/30, 1 ml/min, λ = 210 nm); t_R = 15.00 min (minor, *ent*-**4a**), 17.53 min (major, **4a**).

(*R*)-5-Methylisochromane-1-carboxamide (4b)

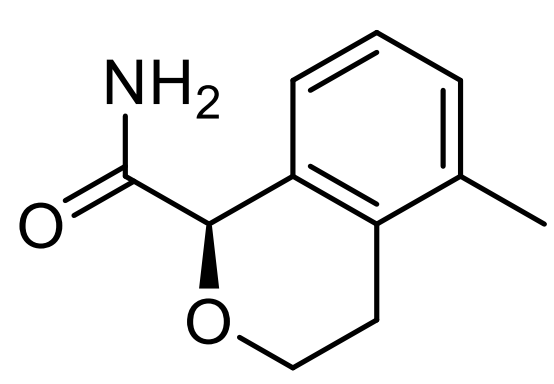

4b
$C_{11}H_{13}NO_2$
MW = 191.23 gmol^{-1}

A solution of 5-methylisochromane-1-carboxamide (*rac*-**4a**) (4.78 mg, 25.0 μmol, 1.00 equiv.) and enantiomerically pure (+)-benzophenone **1** (1.01 mg, 2.50 μmol, 10.0 mol%) in 10 mL dry $PhCF_3$ was degassed by being sparged with argon under ultrasonication for 15 minutes. The solution was irradiated at λ = 366 nm at 30 °C for 18 h. After irradiation, the volatile compounds were removed under reduced pressure and the crude reaction mixture was subjected to FCC (SiO_2, 50 → 100% EtOAc/hexanes) to yield (*R*)-5-methylisochromane-1-carboxamide (**4b**) as a white solid (4.15 mg, 21.7 μmol, 87%, 97.5:2.5 e.r.).

TLC (50% EtOAc/hexanes): R_f = 0.35 [UV, $KMnO_4$].

Specific Rotation: $[\alpha]_D^{27}$: −67 (*c* = 1.0, $CHCl_3$) [97.5:2.5 e.r.].

Chiral HPLC: 97.5:2.5 e.r. (Daicel Chiralpak, AS-H 250 × 4.6 mm, *n*-heptane/^{i}PrOH = 70/30, 1 ml/min, λ = 210 nm); t_R = 15.05 min (minor, *ent*-**4a**), 17.61 min (major, **4a**).

tert-butyl (*S*)-(2-Amino-2-oxo-1-phenylethyl)carbamate (5a)

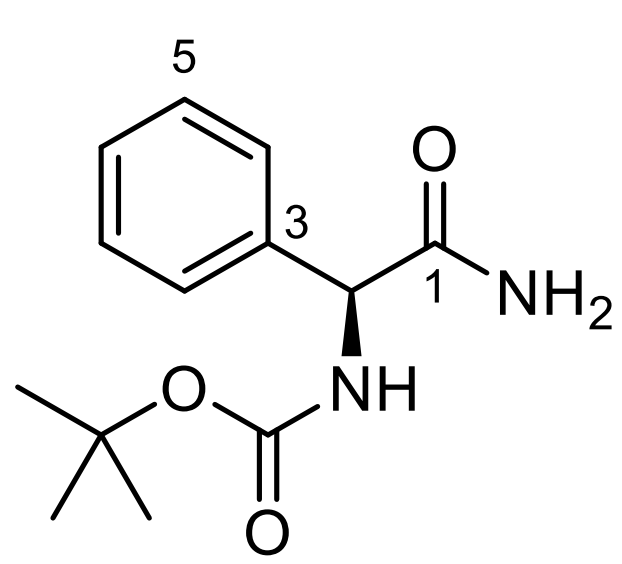

5a
$C_{13}H_{18}N_2O_3$
MW = 250.30 gmol^{-1}

According to GP F, a solution of *tert*-butyl (2-amino-2-oxo-1-phenylethyl)carbamate (*rac*-**5a**) (6.26 mg, 25.0 μmol, 1.00 equiv.) and enantiomerically pure (+)-benzophenone **1** (755 μg, 1.87 μmol, 7.50 mol%) in 10 mL dry $PhCF_3$ was degassed by being sparged with argon under ultrasonication for 15 minutes. The solution was irradiated at λ = 366 nm at 30 °C for 18 h. Another portion of **1** (755 μg, 1.87 μmol, 7.50 mol%) was added and the solution was again degassed for 15 min and then irradiated at λ = 366 nm for 7 h. After irradiation, the volatile

compounds were removed under reduced pressure and the crude reaction mixture was subjected to FCC (SiO_2, 50 → 100% EtOAc/hexanes) to yield *tert*-butyl (*S*)-(2-amino-2-oxo-1-phenylethyl)carbamate (**5a**) as a white solid (5.40 mg, 21.6 μmol, 86%, 92:8 e.r.).

Specific Rotation: $[\alpha]_D^{27}$: +118 (*c* = 1.0, $CHCl_3$) [92:8 e.r.].

Chiral HPLC: 92:8 e.r. (Chiralpak AD-H 250 × 4.6 mm, *n*-heptane/^{i}PrOH = 90/10, 1 ml/min, λ = 210 nm); t_R = 13.25 min (major, **5a**), 17.02 min (minor, *ent*-**5a**).

[**N.B.**: solubility of **5a** in TFT is poor]

tert-butyl (2-Amino-2-oxo-1-phenylethyl)(methyl)carbamate (5b)

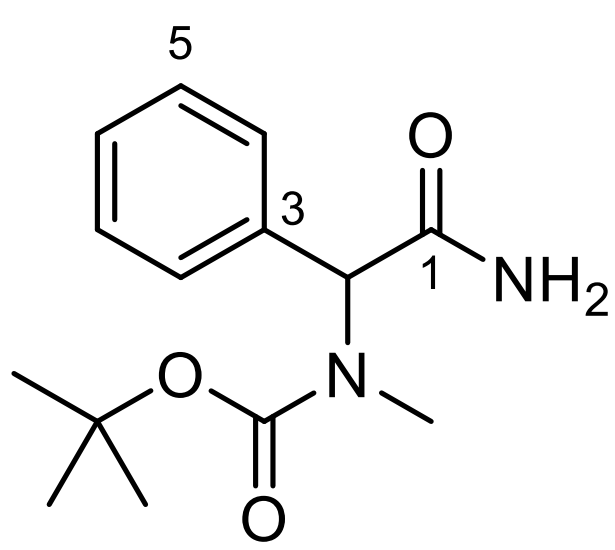

5b
$C_{14}H_{20}N_2O_3$
MW = 264.33 gmol^{-1}

According to GP F, a solution of *tert*-butyl (2-amino-2-oxo-1-phenylethyl)(methyl)carbamate (*rac*-**5b**) (6.61 mg, 25.0 μmol, 1.00 equiv.) and enantiomerically pure (+)-benzophenone **1** (755 μg, 1.87 μmol, 7.50 mol%) in 10 mL dry $PhCF_3$ was degassed by being sparged with argon under ultrasonication for 15 minutes. The solution was irradiated at λ = 366 nm at 30 °C for 18 h. Another portion of **1** (755 μg, 1.87 μmol, 7.50 mol%) was added and the solution was again degassed for 15 min and then irradiated at λ = 366 nm for 7 h. After irradiation, the volatile compounds were removed under reduced pressure and the crude reaction mixture was subjected to FCC (SiO_2, 50 → 100% EtOAc/hexanes) to yield *tert*-butyl-(2-amino-2-oxo-1-phenylethyl)carbamate (**5b**) as a white solid (6.20 mg, 23.5 μmol, 94%, 52:48 e.r.).

Chiral HPLC: 52:48 e.r. (Chiralpak AD-H 250 × 4.6 mm, *n*-heptane/^{i}PrOH = 90/10, 1 ml/min, λ = 210 nm); t_R = 9.34 min (major, **5b**), 11.27 min (minor, *ent*-**5b**).

Benzyl (*S*)-(2-amino-2-oxo-1-phenylethyl)carbamate (5c)

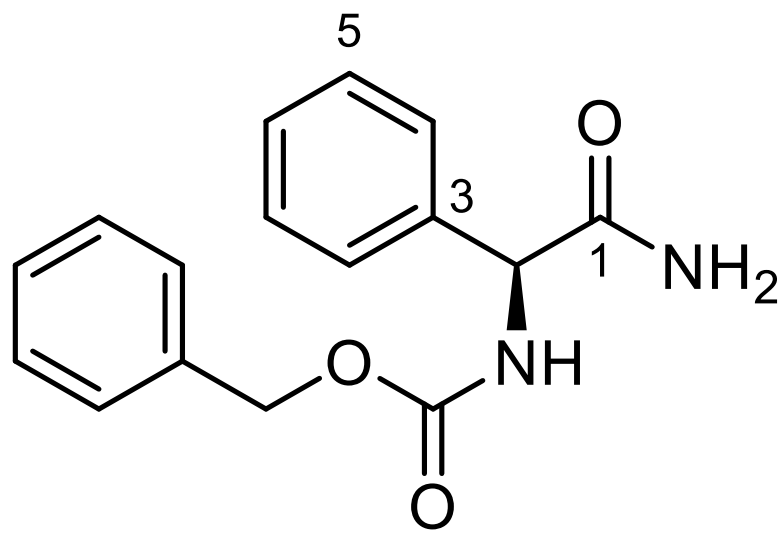

5c

$C_{16}H_{16}N_2O_3$

MW = 284.32 $gmol^{-1}$

According to GP F, a of solution benzyl (2-amino-2-oxo-1-phenylethyl)carbamate (*rac*-**5c** 45:55 e.r.) (7.11 mg, 25.0 μmol, 1.00 equiv.) and enantiomerically pure (+)-benzophenone **1** (755 μg, 1.87 μmol, 7.50 mol%) in 10 mL dry $PhCF_3$ was degassed by being sparged with argon under ultrasonication for 15 minutes. The solution was irradiated at λ = 366 nm at 30 °C for 18 h. Another portion of **1** (755 μg, 1.87 μmol, 7.50 mol%) was added and the solution was again degassed for 15 min and then irradiated at λ = 366 nm for 7 h. After irradiation, the volatile compounds were removed under reduced pressure and the crude reaction mixture was subjected to FCC (SiO_2, 50 → 100% EtOAc/hexanes) to yield benzyl (*S*)-(2-amino-2-oxo-1-phenylethyl)carbamate (**5c**) as a white solid (6.30 mg, 22.2 μmol, 89%, 96:4 e.r.).

Specific Rotation: $[\alpha]_D^{28}$: +82 (*c* = 1.0, DMSO) [96:4 e.r.].

Chiral HPLC: 96:4 e.r. (Chiralpak AD-H 250 × 4.6 mm, *n*-heptane/^{i}PrOH = 70/30, 1 ml/min, λ = 210 nm); t_R = 9.52 min (major, **5c**), 13.81 min (minor, *ent*-**5c**).

[**N.B.**: solubility of **5c** in TFT is very poor, the reaction mixture was heated before starting the irradiation to obtain a cloudy suspension]

***tert*-Butyl (*S*)-(2-amino-1-(4-fluorophenyl)-2-oxoethyl)carbamate (5d)**

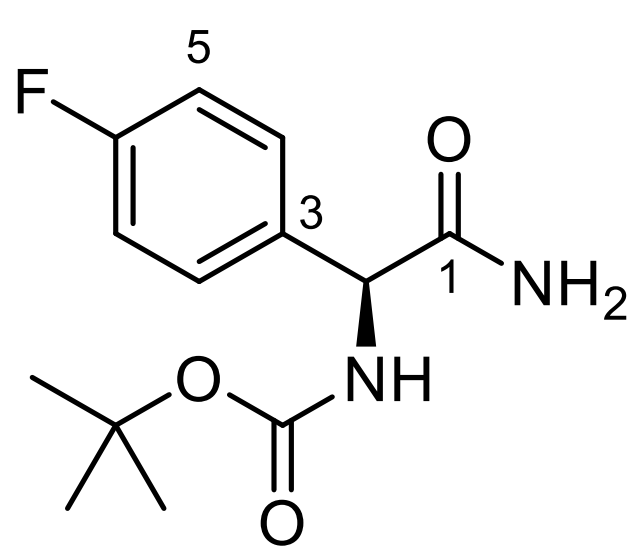

5d

$C_{13}H_{17}FN_2O_3$

MW = 268.29 $gmol^{-1}$

According to GP F, a solution of *tert*-butyl (2-amino-1-(4-fluorophenyl)-2-oxoethyl)carbamate (*rac*-**5d**) (6.71 mg, 25.0 μmol, 1.00 equiv.) and enantiomerically pure (+)-benzophenone **1** (755 μg, 1.87 μmol, 7.50 mol%) in 10 mL dry $PhCF_3$ was degassed by being sparged with argon under ultrasonication for 15 minutes. The solution was irradiated at λ = 366 nm at 30 °C for 18 h. Another portion of **1** (755 μg, 1.87 μmol, 7.50 mol%) was added and the solution was again degassed for 15 min and then irradiated at λ = 366 nm for 7 h. After irradiation, the volatile compounds were removed under reduced pressure and the crude reaction mixture was subjected to FCC (SiO_2, 50 → 100% EtOAc/hexanes) to yield tert-butyl (*S*)-(2-

amino-1-(4-fluorophenyl)-2-oxoethyl)carbamate (**5d**) as a white solid (4.93 mg, 18.4 μmol, 73%, 81:19 e.r.).

Specific Rotation: $[\alpha]_D^{27}$: +82 (c = 1.0, $CHCl_3$) [81:19 e.r.].

Chiral HPLC: 81:19 e.r. (Chiralpak AD-H 250 × 4.6 mm, *n*-heptane/^{i}PrOH = 90/10, 1 ml/min, λ = 210 nm); t_R = 12.32 min (major, **5d**), 18.38 min (minor, *ent*-**5d**).

[**N.B.**: solubility of **5d** in TFT is poor]

***tert*-Butyl (*S*)-(2-amino-1-(4-chlorophenyl)-2-oxoethyl)carbamate (5e)**

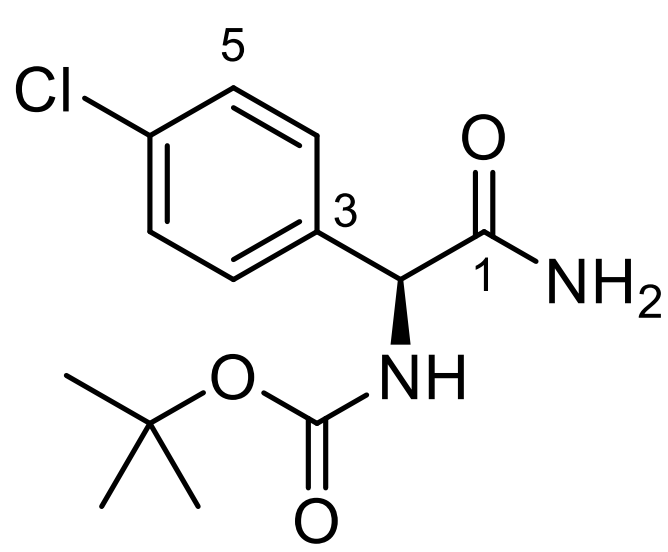

5e
$C_{13}H_{17}ClN_2O_3$
MW = 284.74 gmol^{-1}

According to GP F, a solution of *tert*-butyl (2-amino-1-(4-chlorophenyl)-2-oxoethyl)carbamate (*rac*-**5e**) (7.12 mg, 25.0 μmol, 1.00 equiv.) and enantiomerically pure (+)-benzophenone **1** (755 μg, 1.87 μmol, 7.50 mol%) in 10 mL dry $PhCF_3$ was degassed by being sparged with argon under ultrasonication for 15 minutes. The solution was irradiated at λ = 366 nm at 30 °C for 18 h. Another portion of **1** (755 μg, 1.87 μmol, 7.50 mol%) was added and the solution was again degassed for 15 min and then irradiated at λ = 366 nm for 7 h. After irradiation, the volatile compounds were removed under reduced pressure and the crude reaction mixture was subjected to FCC (SiO_2, 50 → 100% EtOAc/hexanes) to yield tert-butyl (*S*)-(2-amino-1-(4-chlorophenyl)-2-oxoethyl)carbamate (**5e**) as a white solid (5.34 mg, 18.8 μmol, 75%, 80.5:19.5 e.r.).

Specific Rotation: $[\alpha]_D^{27}$: +75 (c = 1.0, $CHCl_3$) [80.5:19.5 e.r.].

Chiral HPLC: 80.5:19.5 e.r. (Chiralpak AD-H 250 × 4.6 mm, *n*-heptane/^{i}PrOH = 90/10, 1 ml/min, λ = 210 nm); t_R = 13.11 min (major, **5e**), 21.61 min (minor, *ent*-**5e**).

tert-butyl (*S*)-(1-Amino-1-oxo-3-phenylpropan-2-yl)carbamate (5f)

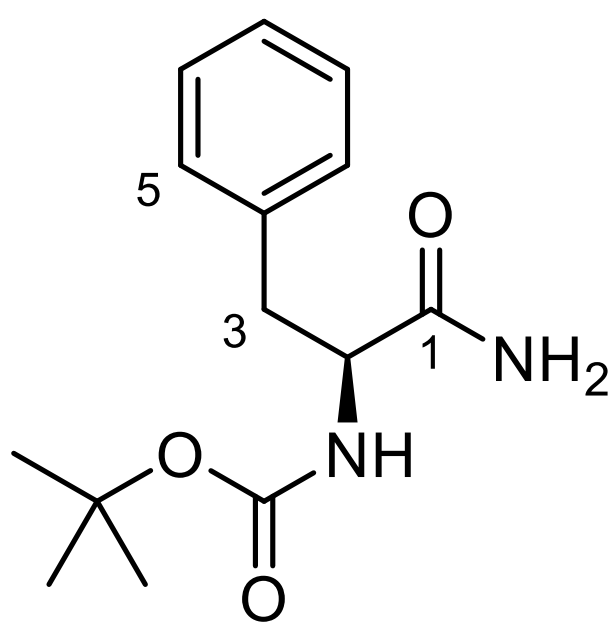

5f
$C_{14}H_{20}N_2O_3$
MW = 264.33 gmol^{-1}

According to GP F, a of solution *tert*-butyl (1-amino-1-oxo-3-phenylpropan-2-yl)carbamate (*rac*-**5f**) (6.61 mg, 25.0 µmol, 1.00 equiv.) and enantiomerically pure (+)-benzophenone **1** (755 µg, 1.87 µmol, 7.50 mol%) in 10 mL dry $PhCF_3$ was degassed by being sparged with argon under ultrasonication for 15 minutes. The solution was irradiated at λ = 366 nm at 30 °C for 18 h. Another portion of **1** (755 µg, 1.87 µmol, 7.50 mol%) was added and the solution was again degassed for 15 min and then irradiated at λ = 366 nm for 7 h. After irradiation, the volatile compounds were removed under reduced pressure and the crude reaction mixture was subjected to FCC (SiO_2, 50 → 100% EtOAc/hexanes) to yield *tert*-butyl (*S*)-(1-amino-1-oxo-3-phenylpropan-2-yl)carbamate (**5f**) as a white solid (5.15 mg, 19.5 µmol, 78%, 83.5:16.5 e.r.).

Specific Rotation: $[\alpha]_D^{27}$: +42 (*c* = 0.5, $CHCl_3$) [83.5:16.5].

Chiral HPLC: 83.5:16.5 (Chiralpak AS-H 250 × 4.6 mm, *n*-heptane/^{i}PrOH = 70/30, 1 ml/min, λ = 210 nm); t_R = 10.81 min (major, **5f**), 24.06 min (minor, *ent*-**5f**).

[**N.B.**: solubility of 5f in TFT is poor]

(*S*)-2-Phenylpropanamide (5g)

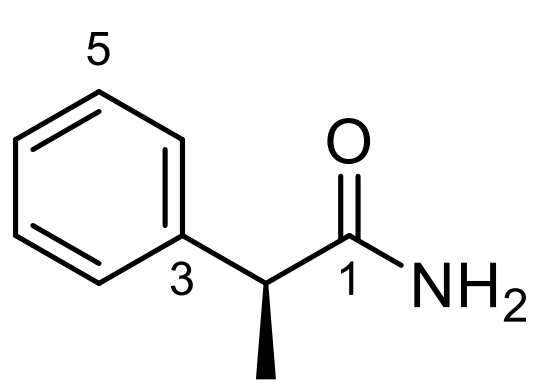

5g
$C_9H_{11}NO$
MW = 149.19 gmol^{-1}

According to GP F, a of solution 2-phenylpropanamide (*rac*-**5g**) (3.73 mg, 25.0 µmol, 1.00 equiv.) and enantiomerically pure (+)-benzophenone **1** (755 µg, 1.87 µmol, 7.50 mol%) in 10 mL dry $PhCF_3$ was degassed by being sparged with argon under ultrasonication for 15 minutes. The solution was irradiated at λ = 366 nm at 30 °C for 18 h. Another portion of **1** (755 µg, 1.87 µmol, 7.50 mol%) was added and the solution was again degassed for 15 min and then irradiated at λ = 366 nm for 7 h. After irradiation, the volatile compounds were removed under reduced pressure and the crude reaction mixture was subjected to FCC (SiO_2, 50 → 100% EtOAc/hexanes) to yield (*S*)-2-phenylpropanamide (**5g**) as a white solid (3.6 mg, 24.1 µmol, 97%, 66:34 e.r.).

Chiral HPLC: 66:34 e.r. (Chiralpak IC 250 × 4.6 mm, *n*-heptane/^{i}PrOH = 70/30, 1 ml/min, λ = 210 nm); t_R = 8.77 min (major, **5g**), 10.29 min (minor, *ent*-**5g**).

2-Fluoro-2-phenylacetamide (5h)

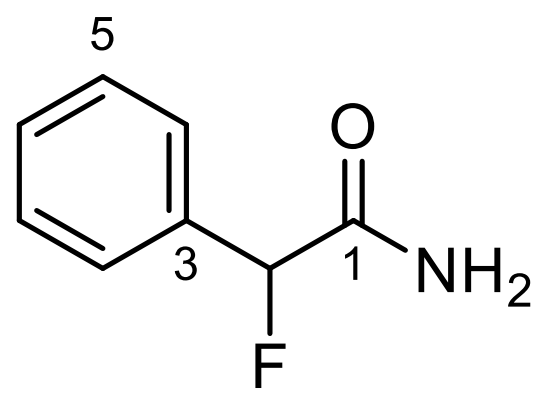

5h
C_8H_8FNO
MW = 153.16 gmol^{-1}

According to GP F, a of solution 2-fluoro-2-phenylacetamide (*rac*-**5h**) (3.73 mg, 25.0 µmol, 1.00 equiv.) and enantiomerically pure (+)-benzophenone **1** (755 µg, 1.87 µmol, 7.50 mol%) in 10 mL dry $PhCF_3$ was degassed by being sparged with argon under ultrasonication for 15 minutes. The solution was irradiated at λ = 366 nm at 30 °C for 18 h. Another portion of **1** (755 µg, 1.87 µmol, 7.50 mol%) was added and the solution was again degassed for 15 min and then irradiated at λ = 366 nm for 7 h. After irradiation, the volatile compounds were removed under reduced pressure and the crude reaction mixture was subjected to FCC (SiO_2, 50 → 100% EtOAc/hexanes) to yield 2-fluoro-2-phenylacetamide (**5h**) as a white solid (3.6 mg, 23.5 µmol, 94%, 66:34 e.r.).

Chiral HPLC: 66:34 e.r. (Chiralpak IA 250 × 4.6 mm, *n*-heptane/^{i}PrOH = 90/10, 1 ml/min, λ = 210 nm); t_R = 8.63 min (major, **5h**), 9.87 min (minor, *ent*-**5h**).

(*S*)-2-bromo-2-phenylacetamide (5i)

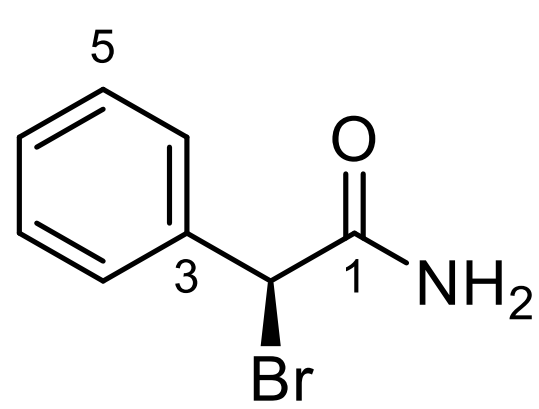

5i
C_8H_8BrNO
MW = 214.06 gmol^{-1}

According to GP F, a solution of 2-bromo-2-phenylacetamide (*rac*-**5i**) (5.35 mg, 25.0 µmol, 1.00 equiv.) and enantiomerically pure (+)-benzophenone **1** (755 µg, 1.87 µmol, 7.50 mol%) in 10 mL dry $PhCF_3$ was degassed by being sparged with argon under ultrasonication for 15 minutes. The solution was irradiated at λ = 366 nm at 30 °C for 18 h. Another portion of **1** (755 µg, 1.87 µmol, 7.50 mol%) was added and the solution was again degassed for 15 min and then irradiated at λ = 366 nm for 7 h. After irradiation, the volatile compounds were removed under reduced pressure and the crude reaction mixture was subjected to FCC (SiO_2, 50 → 100% EtOAc/hexanes) to yield (*S*)-2-bromo-2-phenylacetamide (**5i**) as a white solid (5.0 mg, 23.4 µmol, 93%, 53:47 e.r.).

Chiral HPLC: 53:47 e.r. (Chiralpak AD-H 250 × 4.6 mm, *n*-heptane/^{i}PrOH = 95/5, 1 ml/min, λ = 210 nm); t_R = 26.49 min (major, **5i**), 28.88 min (minor, *ent*-**5i**).

3-Methoxy-2-phenylpropanamide (5j)

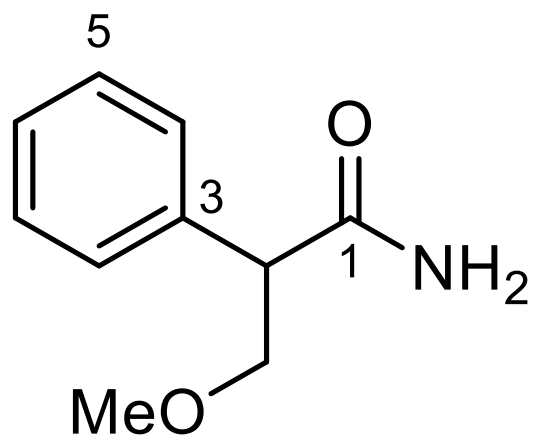

5j
$C_{10}H_{13}NO_2$
MW = 179.22 gmol^{-1}

According to GP F, a of solution 3-methoxy-2-phenylpropanamide (*rac*-**5j**) (4.48 mg, 25.0 µmol, 1.00 equiv.) and enantiomerically pure (+)-benzophenone **1** (755 µg, 1.87 µmol, 7.50 mol%) in 10 mL dry $PhCF_3$ was degassed by being sparged with argon under ultrasonication for 15 minutes. The solution was irradiated at λ = 366 nm at 30 °C for 18 h. Another portion of **1** (755 µg, 1.87 µmol, 7.50 mol%) was added and the solution was again degassed for 15 min and then irradiated at λ = 366 nm for 7 h. After irradiation, the volatile compounds were removed under reduced pressure and the crude reaction mixture was subjected to FCC (SiO_2, 50 → 100% EtOAc/hexanes) to yield 3-methoxy-2-phenylpropanamide (**5j**) as a white solid (3.9 mg, 21.8 µmol, 87%, <51:49 e.r.).

Chiral HPLC: <51:49 e.r. (Chiralpak IC 250 × 4.6 mm, *n*-heptane/^{i}PrOH = 70/30, 1 ml/min, λ = 210 nm); t_R = 11.67 min, 16.55 min.

2-(Methylthio)-2-phenylacetamide (5k)

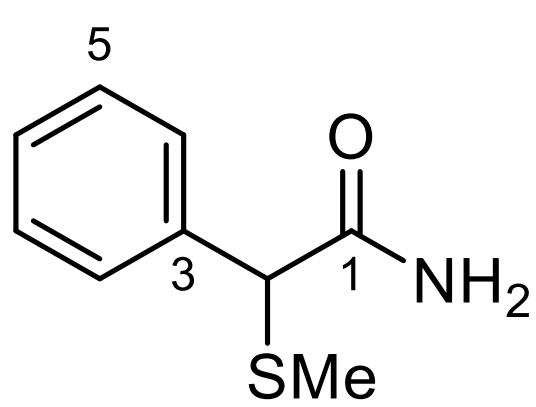

rac-**5k**
$C_9H_{11}NOS$
MW = 181.25 gmol^{-1}

According to GP F, a solution of 2-(methylthio)-2-phenylacetamide (*rac*-**5k**) (4.53 mg, 25.0 µmol, 1.00 equiv.) and enantiomerically pure (+)-benzophenone **1** (755 µg, 1.87 µmol, 7.50 mol%) in 10 mL dry $PhCF_3$ was degassed by being sparged with argon under ultrasonication for 15 minutes. The solution was irradiated at λ = 366 nm at 30 °C for 18 h. Another portion of **1** (755 µg, 1.87 µmol, 7.50 mol%) was added and the solution was again degassed for 15 min and then irradiated at λ = 366 nm for 7 h. After irradiation, the volatile compounds were removed under reduced pressure and the crude reaction mixture was subjected to FCC (SiO_2, 50 → 100% EtOAc/hexanes) to yield (methylthio)-2-phenylacetamide (**5k**) as a white solid (2.09 mg, 11.5 µmol, 46%, <51:49 e.r.).

Chiral HPLC: <51:49 e.r. (Chiralpak IC 250 × 4.6 mm, *n*-heptane/^{i}PrOH = 70/30, 1 ml/min, λ = 210 nm) t_R = 10.55 min (minor, *ent*-**5k**), 12.84 min (major, **5k**).

(*R*)-2-Phenyl-2-(phenylthio)acetamide (5l)

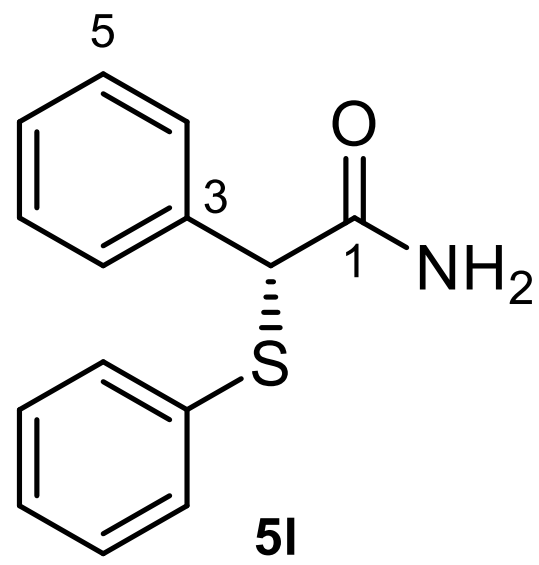

5l

$C_{14}H_{13}NOS$

MW = 243.32 gmol^{-1}

According to GP F, a solution of 2-phenyl-2-(phenylthio)acetamide (*rac*-**5l**) (5.74 mg, 25.0 μmol, 1.00 equiv.) and enantiomerically pure (+)-benzophenone **1** (755 μg, 1.87 μmol, 7.50 mol%) in 10 mL dry $PhCF_3$ was degassed by being sparged with argon under ultrasonication for 15 minutes. The solution was irradiated at λ = 366 nm at 30 °C for 18 h. Another portion of **1** (755 μg, 1.87 μmol, 7.50 mol%) was added and the solution was again degassed for 15 min and then irradiated at λ = 366 nm for 7 h. After irradiation, the volatile compounds were removed under reduced pressure and the crude reaction mixture was subjected to FCC (SiO_2, 50 → 100% EtOAc/hexanes) to yield (*R*)-2-phenyl-2-(phenylthio)acetamide (**5l**) as a white solid (5.35 mg, 22.0 μmol, 88%, 87.5:12.5 e.r.).

Specific Rotation: $[\alpha]_D^{27}$: −34 (*c* = 1.0, $CHCl_3$) [87.5:12.5 e.r.].

Chiral HPLC: 87.5:12.5 e.r. (Chiralpak IC 250 × 4.6 mm, *n*-heptane/^{i}PrOH = 70/30, 1 ml/min, λ = 210 n t_R = 10.88 min (minor, *ent*-**5l**), 12.62 min (major, **5l**).

S11. Stereochemical Editing

The influence of an additional stereocenter on the alkoxy substituents of *rac*-**2** on deracemization efficiency was investigated under the established deracemization conditions.

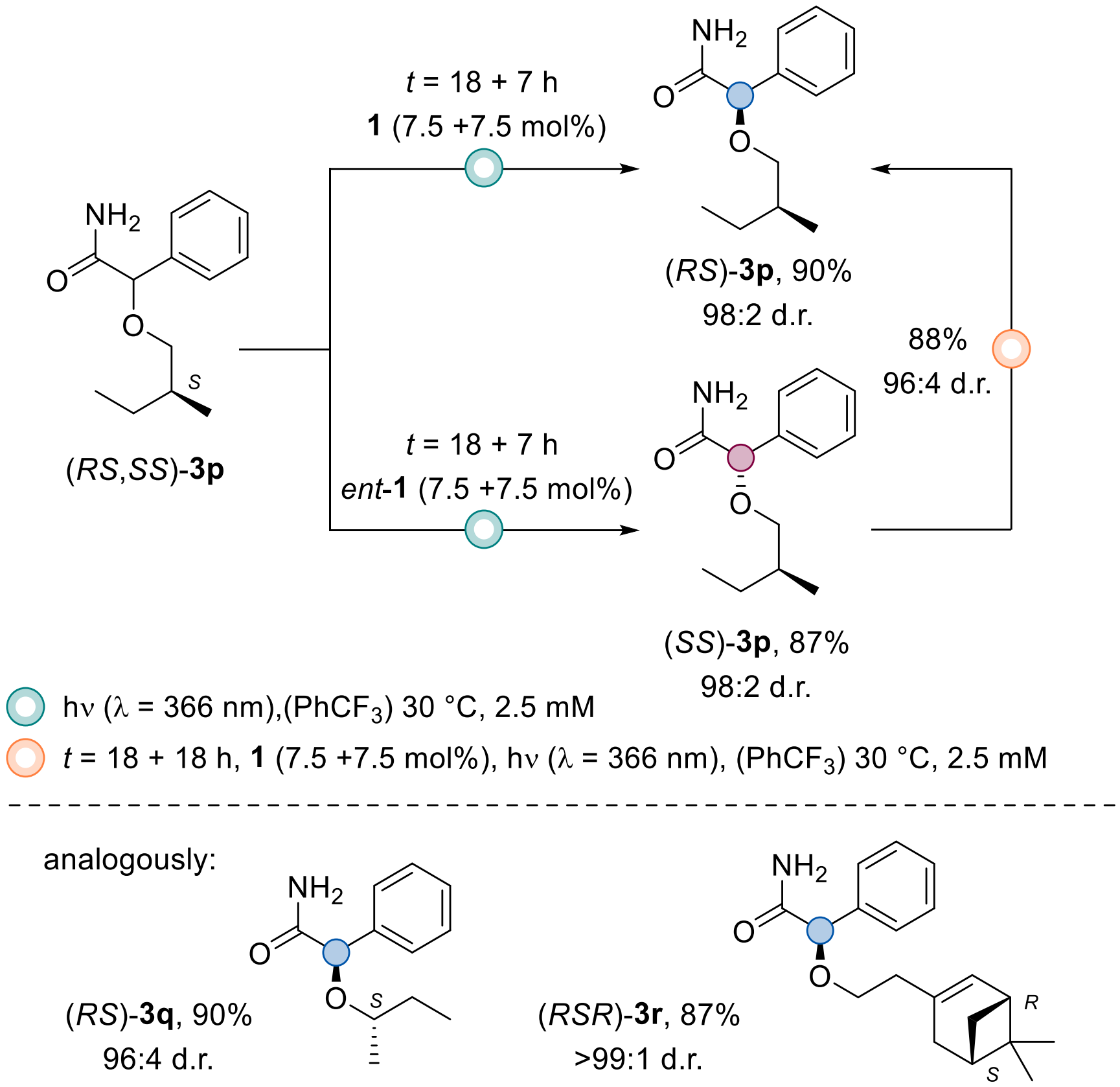

(*R*)-2-((*S*)-2-Methylbutoxy)-2-phenylacetamide (*RS*-**3p**)

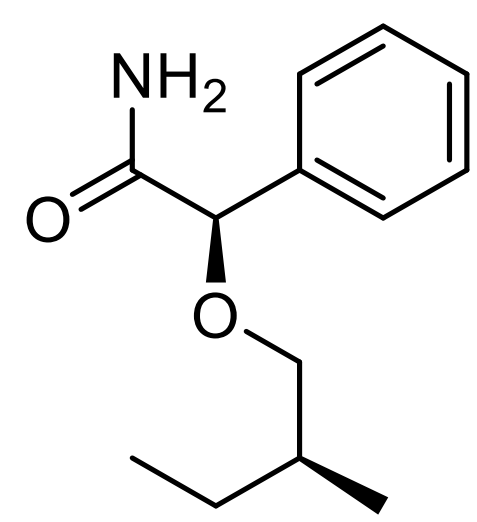

(*RS*)-**3p**
$C_{13}H_{19}NO_2$
MW = 221.30 gmol^{-1}

According to GP F, a solution of 2-((*S*)-2-methylbutoxy)-2-phenylacetamide (*RS,SS*-**3p**) (11.06 mg, 50.0 µmol, 1.00 equiv.) and enantiomerically pure (+)-benzophenone **1** (1.51 mg, 3.75 µmol, 7.50 mol%) in 20 mL dry $PhCF_3$ was degassed by being sparged with argon under ultrasonication for 15 minutes. The solution was irradiated at λ = 366 nm at 30 °C for 18 h. Another portion of **1** (1.51 mg, 3.75 µmol, 7.50 mol%) was added and the solution was again degassed for 15 min and then irradiated at λ = 366 nm for 7 h. After irradiation, the volatile compounds were removed under reduced pressure and the crude reaction mixture was subjected to FCC (SiO_2, 50 → 100% EtOAc/hexanes) to yield (*R*)-2-((*S*)-2-methylbutoxy)-2-phenylacetamide (*RS*-**3p**) as a white solid

(10.0 mg, 45.2 µmol, 90%, 98:2 d.r.). N.B.: Diastereomeric ratio (d.r.) was determined by HPLC analysis as it was not possible to determine by ^{1}H-NMR of crude reaction mixture.

Specific Rotation: $[\alpha]_D^{23}$: −73 (*c* = 1.0, $CHCl_3$) [98:2 d.r.].

Chiral HPLC: 98:2 d.r. (Daicel Chiralpak, AS-H 250 × 4.6 mm, *n*-heptane/^{i}PrOH = 70/30, 1 ml/min, λ = 210 nm); t_R = 11.04 min (minor, *SS*-**3p**), 16.32 min (major, *RS*-**3p**).

(*S*)-2-((*S*)-2-Methylbutoxy)-2-phenylacetamide (*SS*-**3p**)

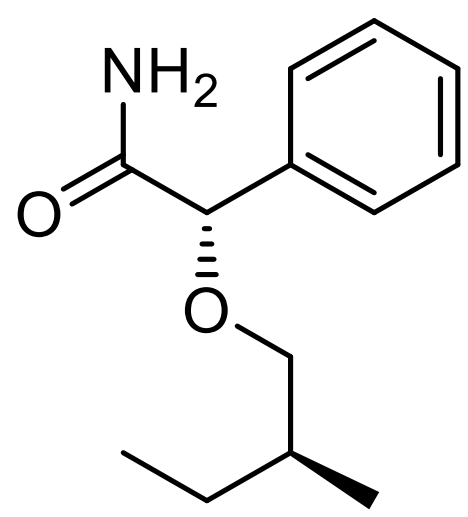

(*SS*)-**3p**
$C_{13}H_{19}NO_2$
MW = 221.30 gmol^{-1}

According to GP F, a of solution 2-((*S*)-2-methylbutoxy)-2-phenylacetamide (*RS*,*SS*-**3p**) (11.06 mg, 50.0 µmol, 1.00 equiv.) and enantiomerically pure (−)-benzophenone **1** (1.51 mg, 3.75 µmol, 7.50 mol%) in 20 mL dry $PhCF_3$ was degassed by being sparged with argon under ultrasonication for 15 minutes. The solution was irradiated at λ = 366 nm at 30 °C for 18 h. Another portion of **1** (1.51 mg, 3.75 µmol, 7.50 mol%) was added and the solution was again degassed for 15 min and then irradiated at λ = 366 nm for 7 h. After irradiation, the volatile compounds were removed under reduced pressure and the crude reaction mixture was subjected to FCC (SiO_2, 50 → 100% EtOAc/hexanes) to yield (*S*)-2-((*S*)-2-methylbutoxy)-2-phenylacetamide (*SS*-**3p**) as a white solid (9.6 mg, 43.4 µmol, 87%, 98:2 d.r.). N.B.: Diastereomeric ratio (d.r.) was determined by HPLC analysis as it was not possible to determine by 1H-NMR of crude reaction mixture.

Specific Rotation: $[\alpha]_D^{23}$: +79 (*c* = 1.0, $CHCl_3$) [98:2 d.r.].

Chiral HPLC: 98:2 d.r. (Daicel Chiralpak, AS-H 250 × 4.6 mm, *n*-heptane/^{i}PrOH = 70/30, 1 ml/min, λ = 210 nm); t_R = 10.90 min (major, *SS*-**3p**), 16.63 min (minor, *RS*-**3p**).

(*R*)-2-((*S*)-2-Methylbutoxy)-2-phenylacetamide (*RS*-3p)

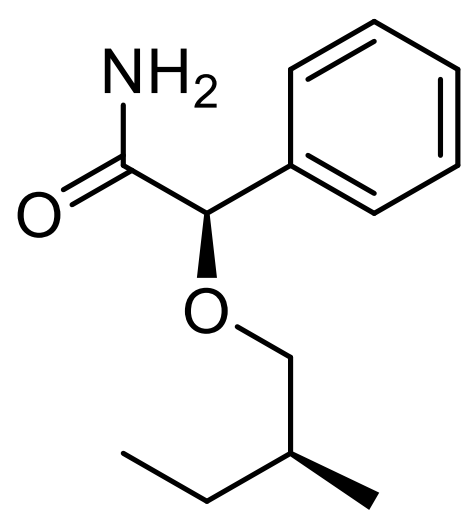

(*RS*)-**3p**
$C_{13}H_{19}NO_2$
MW = 221.30 gmol^{-1}

According to GP F, a solution of (*S*)-2-((*S*)-2-methylbutoxy)-2-phenylacetamide3-methoxy-2-phenylpropanamide (*SS*-**3p**) (5.53 mg, 25.0 µmol, 1.00 equiv.) and enantiomerically pure (+)-benzophenone **1** (755 µg, 1.87 µmol, 7.50 mol%) in 10 mL dry $PhCF_3$ was degassed by being sparged with argon under ultrasonication for 15 minutes. The solution was irradiated at λ = 366 nm at 30 °C for 18 h. Another portion of **1** (755 µg, 1.87 µmol, 7.50 mol%) was added and the solution was again degassed for 15 min and then irradiated at λ = 366 nm for 18 h. After irradiation, the volatile compounds were removed under reduced pressure and the crude reaction mixture was subjected to FCC (SiO_2, 50 → 100% EtOAc/hexanes) to yield (*R*)-2-((*S*)-2-methylbutoxy)-2-phenylacetamide (*RS*-**3p**) as a white solid (4.85 mg, 21.9 µmol, 88%, 96:4 d.r.). N.B.: Diastereomeric ratio (d.r.) was determined by HPLC analysis as it was not possible to determine by 1H-NMR of crude reaction mixture.

(*R*)-2-((*S*)-Sec-butoxy)-2-phenylacetamide (*RS*-3q)

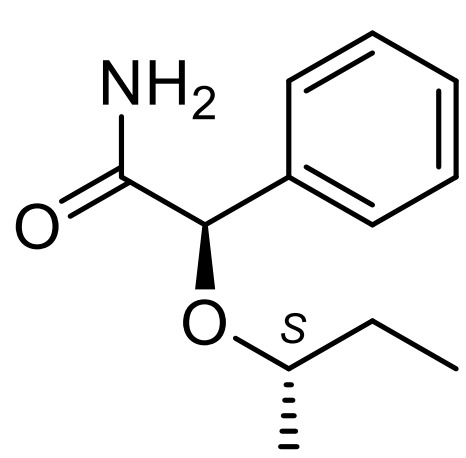

(*RS*)-**3q**
$C_{12}H_{17}NO_2$
MW = 207.27 gmol^{-1}

According to GP F, a solution of 2-((*S*)-sec-butoxy)-2-phenylacetamide (*RS*,*SS*-**3q**) (20.73 mg, 75.0 µmol, 1.00 equiv.) and enantiomerically pure (+)-benzophenone **1** (2.26 mg, 5.63 µmol, 7.50 mol%) in 30 mL dry $PhCF_3$ was degassed by being sparged with argon under ultrasonication for 15 minutes. The solution was irradiated at λ = 366 nm at 30 °C for 18 h. Another portion of **1** (2.26 mg, 5.63 µmol, 7.50 mol%) was added and the solution was again degassed for 15 min and then irradiated at λ = 366 nm for 7 h. After irradiation, the volatile compounds were removed under reduced pressure and the crude reaction mixture was subjected to FCC (SiO_2, 50 → 100% EtOAc/hexanes) to yield (*R*)-2-((*S*)-Sec-butoxy)-2-phenylacetamide (*RS*-**3q**) as a white solid (14.0 mg, 67.6 µmol, 90%, 96:4 d.r.).

Specific Rotation: $[a]_D^{23}$: −51 (*c* = 1.0, $CHCl_3$) [96:4 d.r.].

Chiral HPLC: 96:4 d.r. (Chiralpak IC 250 × 4.6 mm, *n*-heptane/^{i}PrOH = 70/30, 1 ml/min, λ = 210 nm); t_R = 11.78 min (major, *RS*-**3q**), 22.99 min (minor, *SS*-**3q**).

(*R*)-2-(2-((1*R*,5*S*)-6,6-Dimethylbicyclo[3.1.1]hept-2-en-3-yl)ethoxy)-2-phenylacetamide (*RSR*-**3r**)

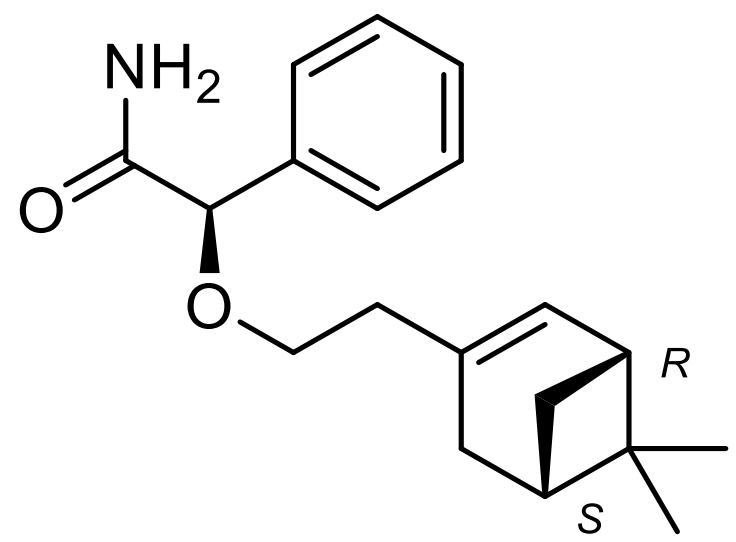

(*RSR*)-**3r**
$C_{19}H_{25}NO_2$
MW = 299.41 gmol^{-1}

According to GP F, a solution of 2-(2-((1*R*,5*S*)-6,6-dimethylbicyclo[3.1.1]hept-2-en-3-yl)ethoxy)-2-phenylacetamide (*RSR*,*SSR*-**3r**) (14.97 mg, 50.0 µmol, 1.00 equiv.) and enantiomerically pure (+)-benzophenone **1** (1.51 mg, 3.75 µmol, 7.50 mol%) in 20 mL dry $PhCF_3$ was degassed by being sparged with argon under ultrasonication for 15 minutes. The solution was irradiated at λ = 366 nm at 30 °C for 18 h. Another portion of **1** (1.51 mg, 3.75 µmol, 7.50 mol%) was added and the solution was again degassed for 15 min and then irradiated at λ = 366 nm for 7 h. After irradiation, the volatile compounds were removed under reduced pressure and the crude reaction mixture was subjected to FCC (SiO_2, 50 → 100% EtOAc/hexanes) to yield (*R*)-2-(2-((1*R*,5*S*)-6,6-dimethylbicyclo[3.1.1]hept-2-en-3-yl)ethoxy)-2-phenylacetamide (*RSR*-**3r**) as a white solid (13.0 mg, 43.4 µmol, 87%, >99:1 d.r.).

Specific Rotation: $[\alpha]_D^{23}$: −89 (*c* = 1.0, $CHCl_3$) [>99:1 d.r.].

Chiral HPLC: >99:1 d.r. (Daicel Chiralpak, AS-H 250 × 4.6 mm, *n*-heptane/^{i}PrOH = 70/30, 1 ml/min, λ = 210 nm); t_R = 11.70 min (major, *RSR*-**3r**), 19.33 min (minor, *SSR*-**3r**).

S12. Conversion of 2a to *ent*-5i

(*R*)-2-Bromo-2-phenylacetamide (*ent*-**5i**)

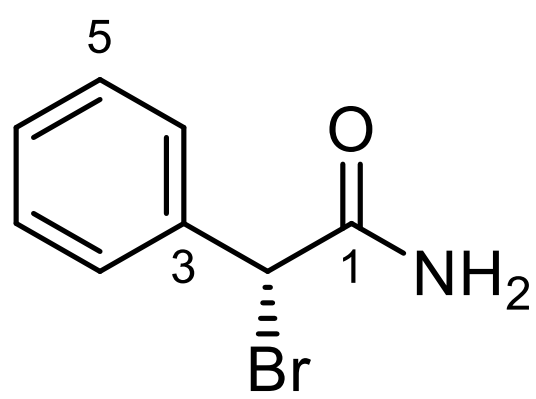

ent-**5i**
C_8H_8BrNO
MW = 153.16 gmol^{-1}

BBr_3 (1.00 M in CH_2Cl_2, 84.2 mg, 336 µL, 336 µmol, 3.70 equiv.) was added dropwise to a solution of 2-methoxy-2-phenylacetamide (*rac*-**2a**) (15.0 mg, 90.8 µmol, 1.00 equiv.) in CH_2Cl_2 (3.0 mL) at −78 C. The resulting solution was stirred allowed to warm to r.t. for 14 h. Subsequently, water (20 mL) was added, and the layers were separated and the aqueous layer was extracted thrice with CH_2Cl_2 (3 × 5 mL). The combined organic layers were washed with brine and dried over Na_2SO_4. The solvents were removed under reduced pressure and the crude product was subjected to FCC (SiO_2, 50 → 100% EtOAc/hexanes) to yield (*R*)-2-bromo-2-phenylacetamide (*ent*-**5i**) as a white solid (19.1 mg, 84.4 µmol, 93%, 81:19 e.r.).

Specific Rotation: $[\alpha]_D^{25}$: +34 (*c* = 1.0, $CHCl_3$) [81:19 e.r.].[9]

Chiral HPLC: 81:19 e.r. (Chiralpak AD-H 250 × 4.6 mm, *n*-heptane/^{i}PrOH = 95/5, 1 ml/min, λ = 210 nm); t_R = 28.28 min (minor, **5i**), 30.92 min (major, *ent*-**5i**).

S13. Detection and Attempted Isolation of Catalyst Coupling Products 9 and 10

(7*R*)-7-(6-(1,2-Dihydroxy-1,2-diphenyl-2-(2-((7*R*)-1,5,7-trimethyl-2-oxo-3-azabicyclo[3.3.1]nonan-7-yl)benzo[d]oxazol-5-yl)ethyl)benzo[d]oxazol-2-yl)-1,5,7-trimethyl-3-azabicyclo[3.3.1]nonan-2-one (9)

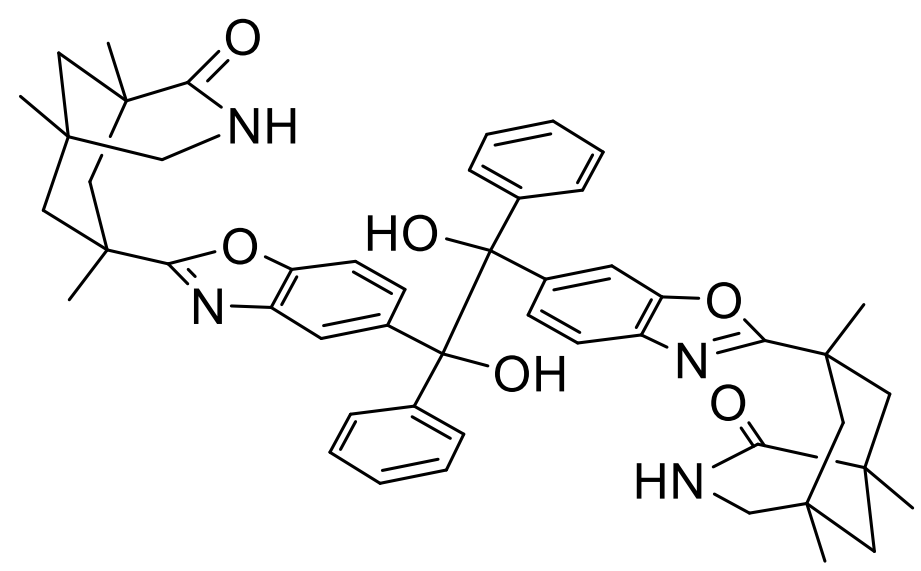

9
$C_{50}H_{54}N_4O_6$
MW = 807.00 gmol^{-1}

According to a modified GP F, a solution of 2-methoxy-2-phenylacetamide (*rac*-**2a**) (33.0 mg, 200 µmol, 1.00 equiv.) and enantiomerically pure (+)-benzophenone **1** (20.1 mg, 50.0 µmol, 25.0 mol%) in 80 mL dry $PhCF_3$ was degassed by being sparged with argon under ultrasonication for 15 minutes. The solution was irradiated at λ = 366 nm at 30 °C for 3 h. The volatile compounds were removed under reduced pressure, and the crude product was analyzed by ESI-HRMS. Separation of the components by FCC (SiO_2, 50 → 100% EtOAc/hexanes) followed by preparative TLC (SiO_2, 100% EtOAc) yielded (7*R*)-7-(6-(1,2-dihydroxy-1,2-diphenyl-2-(2-((7*R*)-1,5,7-trimethyl-2-oxo-3-azabicyclo[3.3.1]nonan-7-yl)benzo[d]oxazol-5-yl)ethyl)benzo[d]oxazol-2-yl)-1,5,7-trimethyl-3-azabicyclo[3.3.1]nonan-2-one (**9**) as an off white solid (7.90 mg) which was assigned by its exact mass. Benzophenone **1** remained as an unquantifiable impurity.

HRMS (ESI) *m/z* $[M+H]^+$: calculated for $[C_{50}H_{55}N_4O_6]^+$: 807.4116; found: 807.4126.

Due to the presence of multiple stereoisomers as well as an impurity of **1**, characterization by NMR was not applicable. Comparison of the chiral HPLC traces of isolated compound **9** with authentic **1** shows the presence of **1** next to three other UV-active compounds which show the same UV-Vis spectra and are assumed to be stereoisomers. While **1** absorbs light in the 366 nm range, pinacol adduct **9** does not (Figure S4, Figure S5).

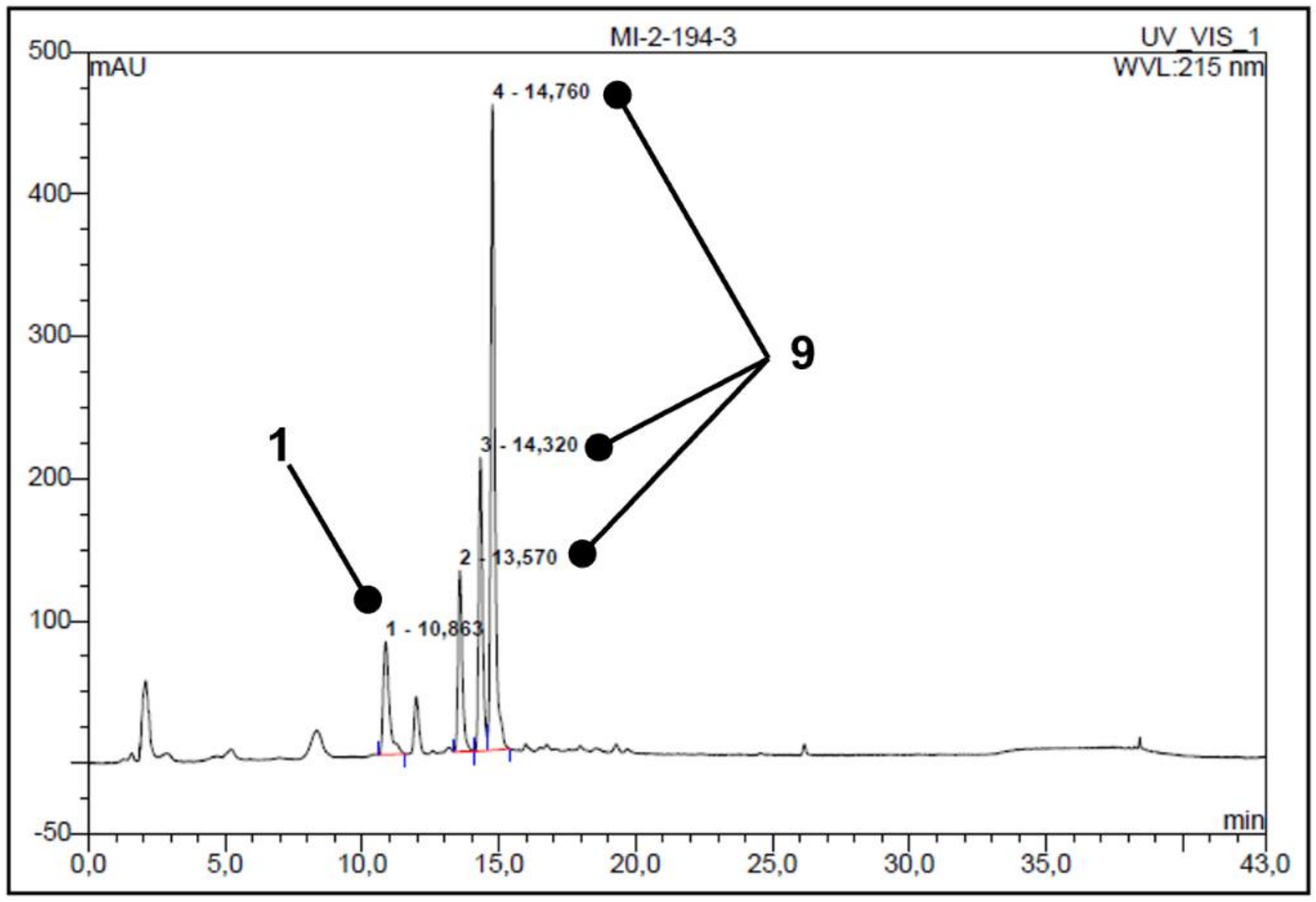

No.	Ret.Time min	Peak Name	Height mAU	Area mAU*min	Rel.Area %	Amount	Type
1	10,86	n.a.	79,279	20,572	13,00	n.a.	BMB
2	13,57	n.a.	127,080	22,819	14,42	n.a.	BMB
3	14,32	n.a.	206,192	34,639	21,89	n.a.	BM
4	14,76	n.a.	454,016	80,217	50,69	n.a.	MB
Total:			866,568	158,246	100,00	0,000	

Figure S4: HPLC trace of pinacol product **9** with **1** as an impurity.

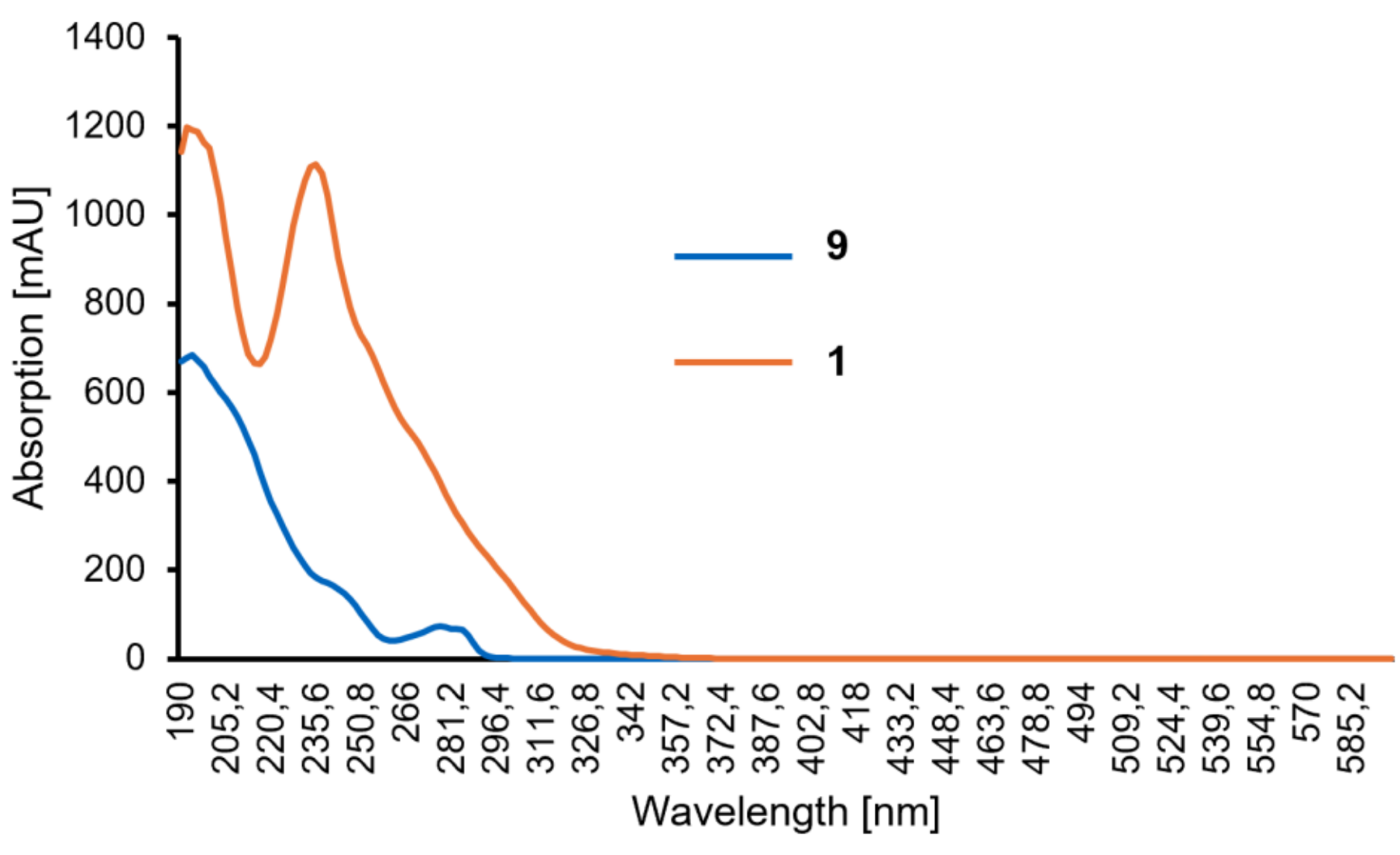

Figure S5: UV-Vis spectra of (+)-benzophenone catalyst **1** and its pinacol adduct **9**.

3-Hydroxy-2-methoxy-2,3-diphenyl-3-(2-((7*R*)-1,5,7-trimethyl-2-oxo-3-azabicyclo[3.3.1]nonan-7-yl)benzo[d]oxazol-5-yl)propanamide (10)

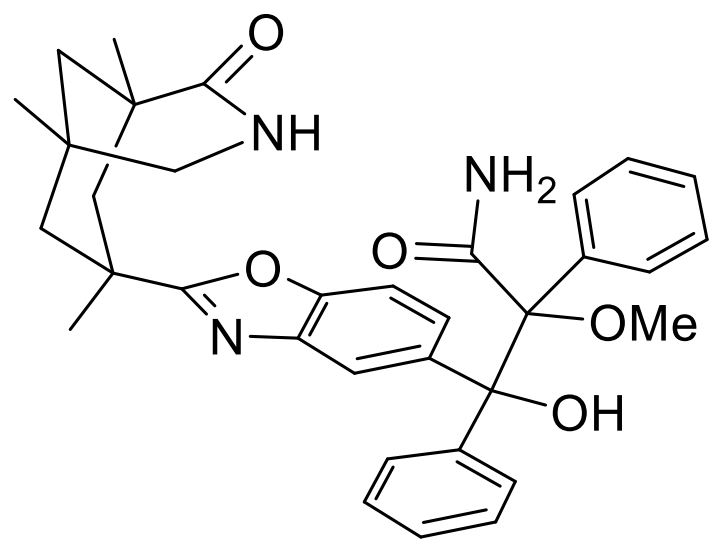

10
$C_{34}H_{37}N_3O_5$
MW = 567.69 gmol^{-1}

According to a modified GP F, a solution of 2-methoxy-2-phenylacetamide (*rac*-**2a**) (33.0 mg, 200 μmol, 1.00 equiv.) and enantiomerically pure (+)-benzophenone **1** (20.1 mg, 50.0 μmol, 25.0 mol%) in 80 mL dry $PhCF_3$ was degassed by being sparged with argon under ultrasonication for 15 minutes. The solution was irradiated at λ = 366 nm at 30 °C for 3 h. The volatile compounds were removed under reduced pressure, and the crude product was analyzed by ESI-HRMS. An attempted isolation of 3-hydroxy-2-methoxy-2,3-diphenyl-3-(2-((7*R*)-1,5,7-trimethyl-2-oxo-3-azabicyclo[3.3.1]nonan-7-yl)benzo[d]oxazol-5-yl)propanamide (**10**) by FCC (SiO_2, 50 → 100% EtOAc/hexanes) was unsuccessful. The presence of 1:1 adduct **10** in the crude product was however confirmed by HRMS.

HRMS (ESI) *m/z* $[M+H]^+$: calculated for $[C_{34}H_{38}N_3O_5]^+$: 568.2806; found: 568.2804.

S14. Deracemization of *rac*-2a with 9

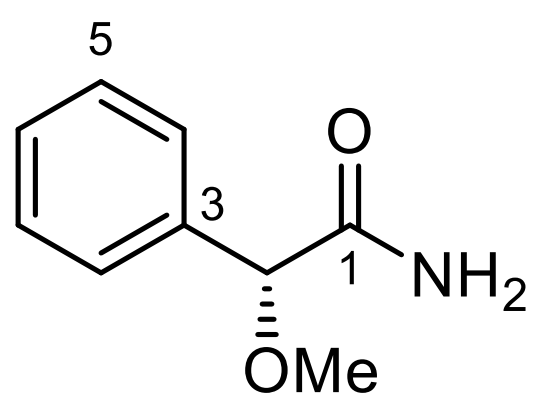

2a

$C_9H_{11}NO_2$

MW = 165.19 gmol^{-1}

A solution of 2-methoxy-2-phenylacetamide (*rac*-**2a**) (4.13 mg, 25.0 µmol, 1.00 equiv.) and (7*R*)-7-(6-(1,2-dihydroxy-1,2-diphenyl-2-(2-((7*R*)-1,5,7-trimethyl-2-oxo-3-azabicyclo[3.3.1]nonan-7-yl)benzo[d]oxazol-5-yl)ethyl)benzo[d]oxazol-2-yl)-1,5,7-trimethyl-3-azabicyclo[3.3.1]nonan-2-one (**9**) in 10 mL dry $PhCF_3$ was degassed by being sparged with argon under ultrasonication for 15 minutes. The solution was irradiated at λ = 366 nm at 30 °C for 13 h. After irradiation, the volatile compounds were removed and an e.r. of 59:41 of the crude product was determined by chiral HPLC.

Chiral HPLC: 59:41 e.r. (AS-H 250 × 4.6 mm, *n*-heptane/^{i}PrOH = 70/30, 1 ml/min, λ = 210 nm); t_R = 18.83 min (minor, *ent*-**2a**), 23.40 min (major, **2a**).

S15. Racemization Studies

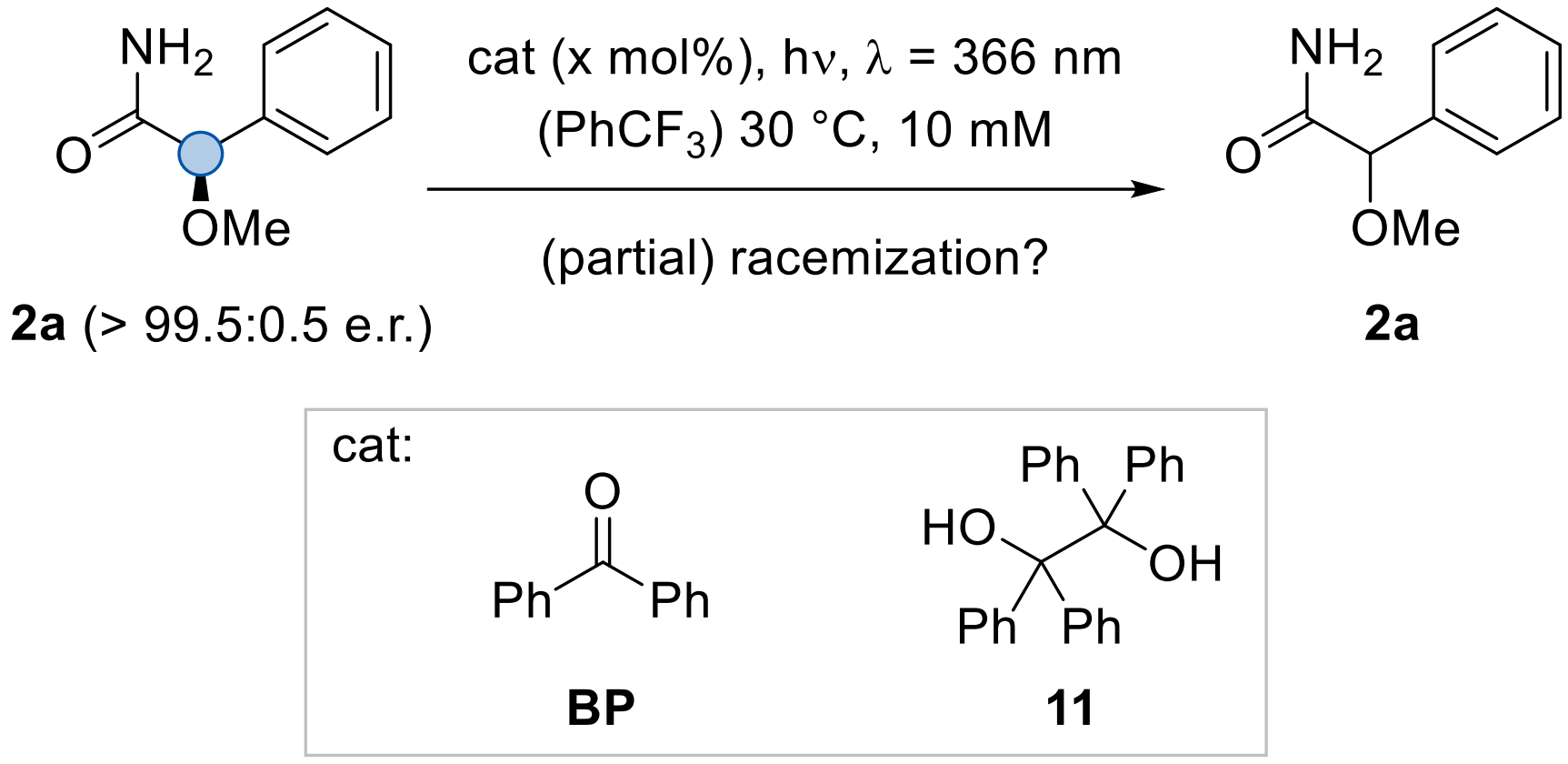

Table S3: Racemization conditions for the racemization of (*R*)-**2a**. All reactions were performed on a 50.0 μmol scale.

Entry	cat	x	λ [nm]	t [h]	yield (%)	e.r.
1	**-**	-	366	4	quant.	>99.5:0.5
2	**BP**	10.0	366	4	98	94.5:5.5
3	**BP**	20.0	366	4	98	91:9
4	**BP**	20.0	366	15	97	89:11
5	**11**	20.0	366	4	99	>99.5:0.5
6	**11**	20.0	366	15	99	>99.5:0.5
7	**11**	20.0	350	15	99	>99.5:0.5

A solution of (*R*)-**2a** (8.26 mg, 50.0 μmol, c = 10 mM, 1.00 equiv.) and benzophenone (1.82 mg, 10.0 μmol 0.2 equiv.) in 5 mL dry $PhCF_3$ was degassed by being sparged with argon under ultrasonication for 15 minutes. The solution was irradiated at λ = 366 nm at 30 °C for 4 h and 15 h. After irradiation, all the volatile compounds were removed under reduced pressure and the crude products were subjected to FCC (SiO_2, 60 → 80% EtOAc/hexanes) to afford **2a**.

Similarly, a solution of (*R*)-**2a** (8.26 mg, 50.0 μmol, c = 10 mM, 1.00 equiv.) and **11** (3.67 mg, 10.0 μmol 0.2 equiv.) in 5 mL dry $PhCF_3$ was degassed by being sparged with argon under ultrasonication for 15 minutes. The solution was irradiated at above mentioned wavelength (λ = 366 nm or 350 nm) at 30 °C for 4 h and 15 h. After irradiation, all the volatile compounds were

removed under reduced pressure and the crude products were subjected to FCC (SiO_2, 60 → 80% EtOAc/hexanes) to afford **2a.**

S16. NMR Spectra

^{1}H-NMR, $CDCl_3$, 400 MHz

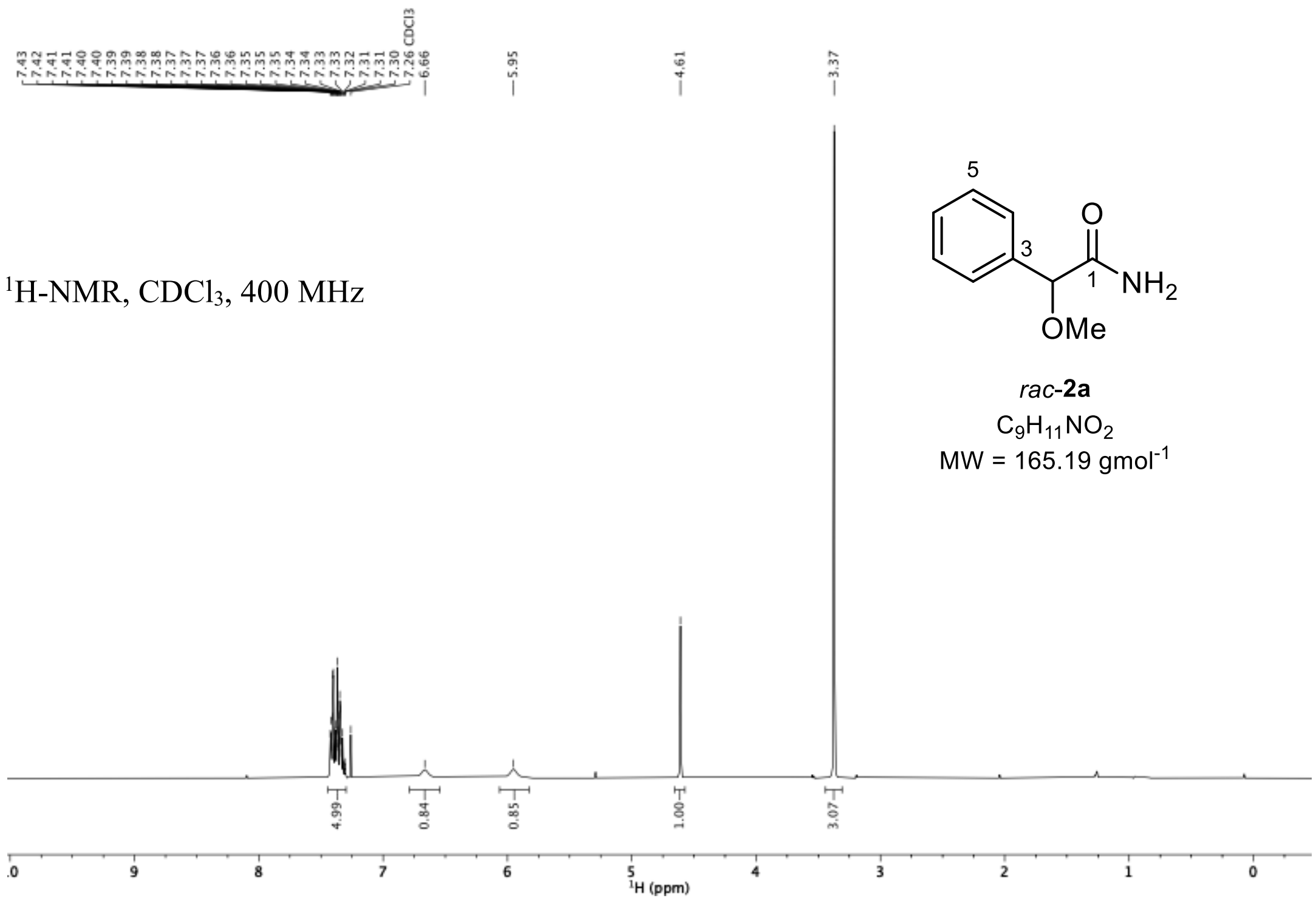

^{13}C-NMR, $CDCl_3$, 101 MHz

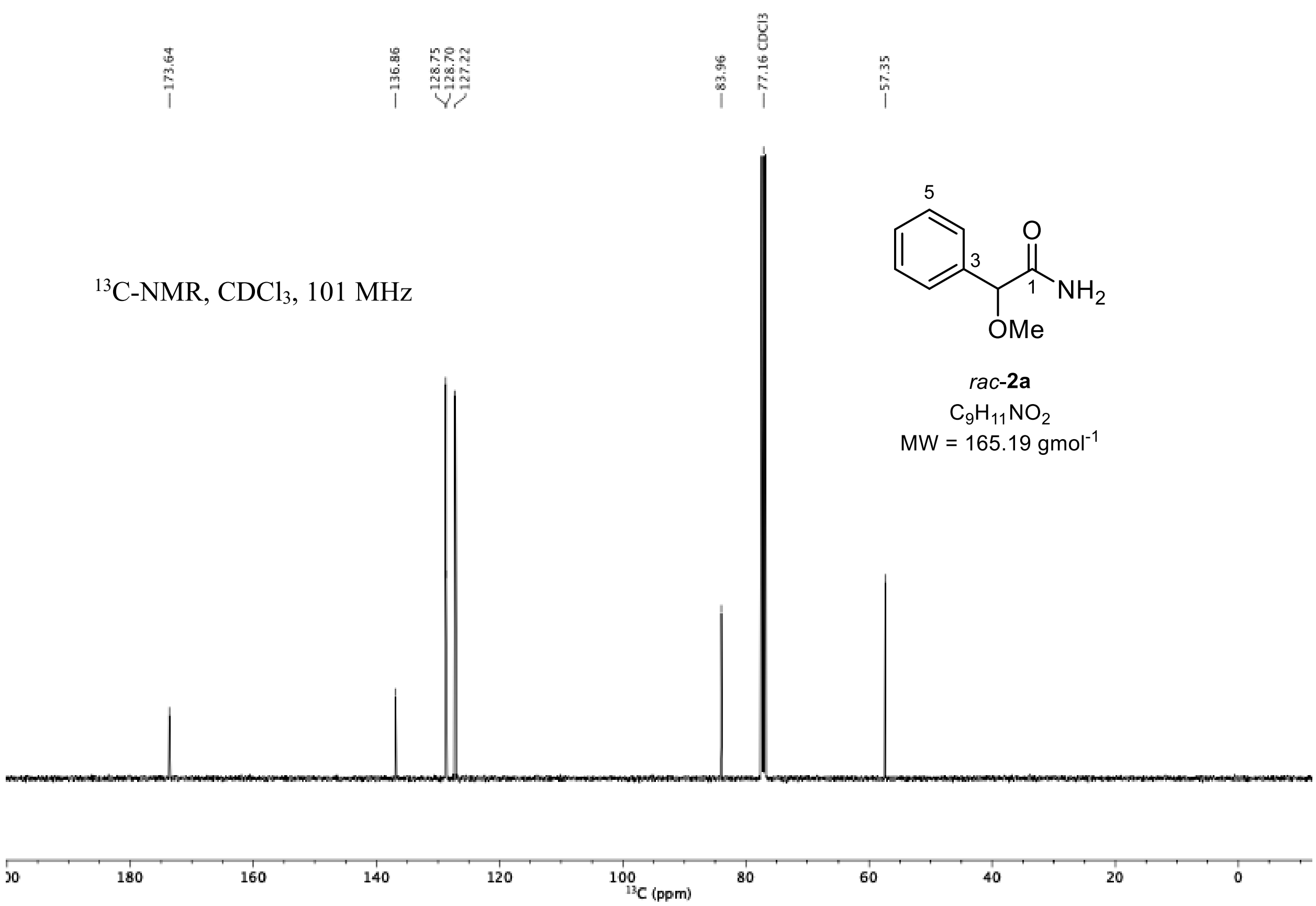

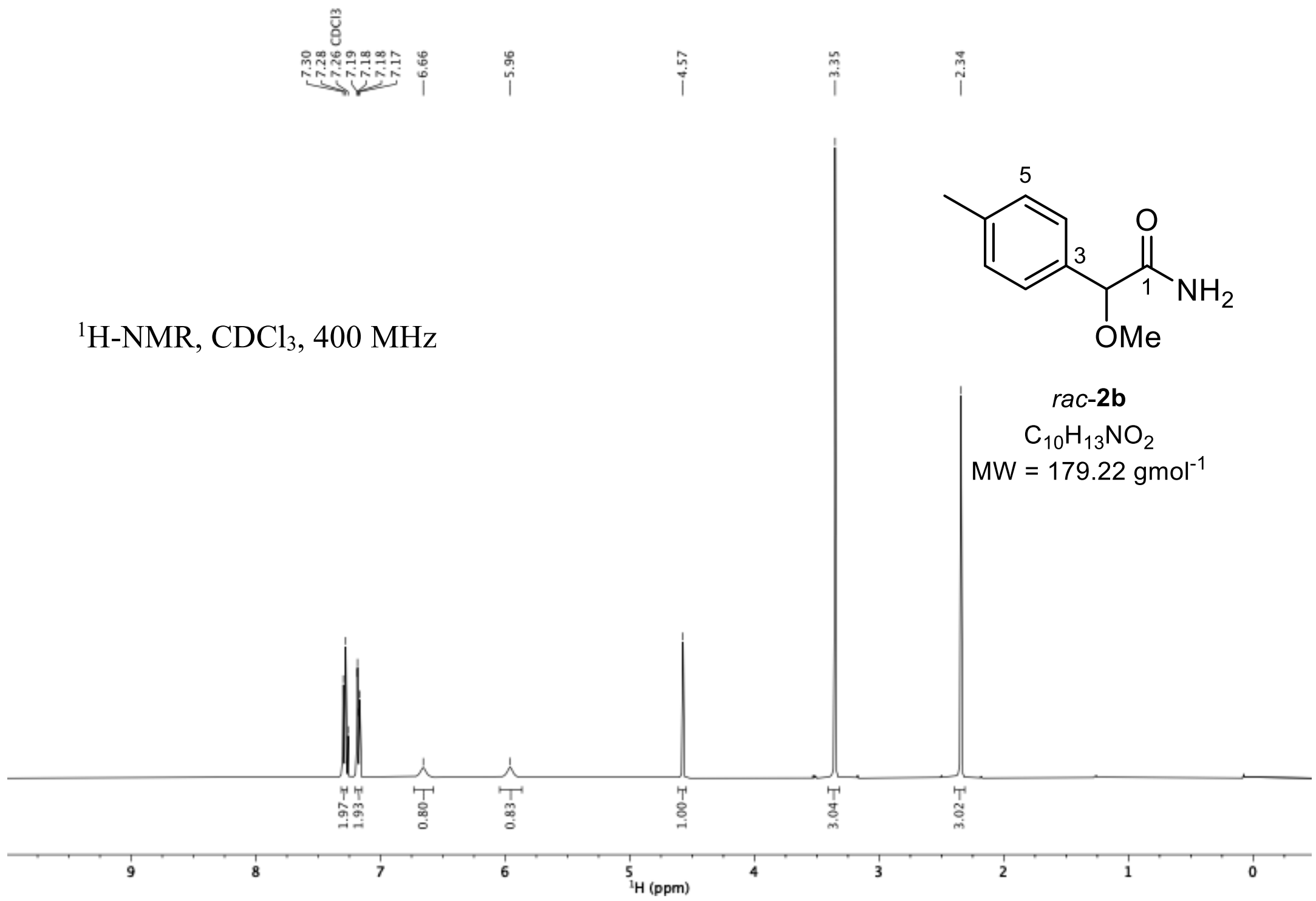
7.30
7.28
7.26 CDCl3
7.19
7.18
7.18
7.17
6.66
5.96
4.57
3.35
2.34
1H-NMR, CDCl3, 400 MHz
5
3
1
O
NH2
OMe
rac-2b
C10H13NO2
MW = 179.22 gmol-1
1.97
1.93
0.80
0.83
1.00
3.04
3.02
9
8
7
6
5
4
3
2
1
0
1H (ppm)

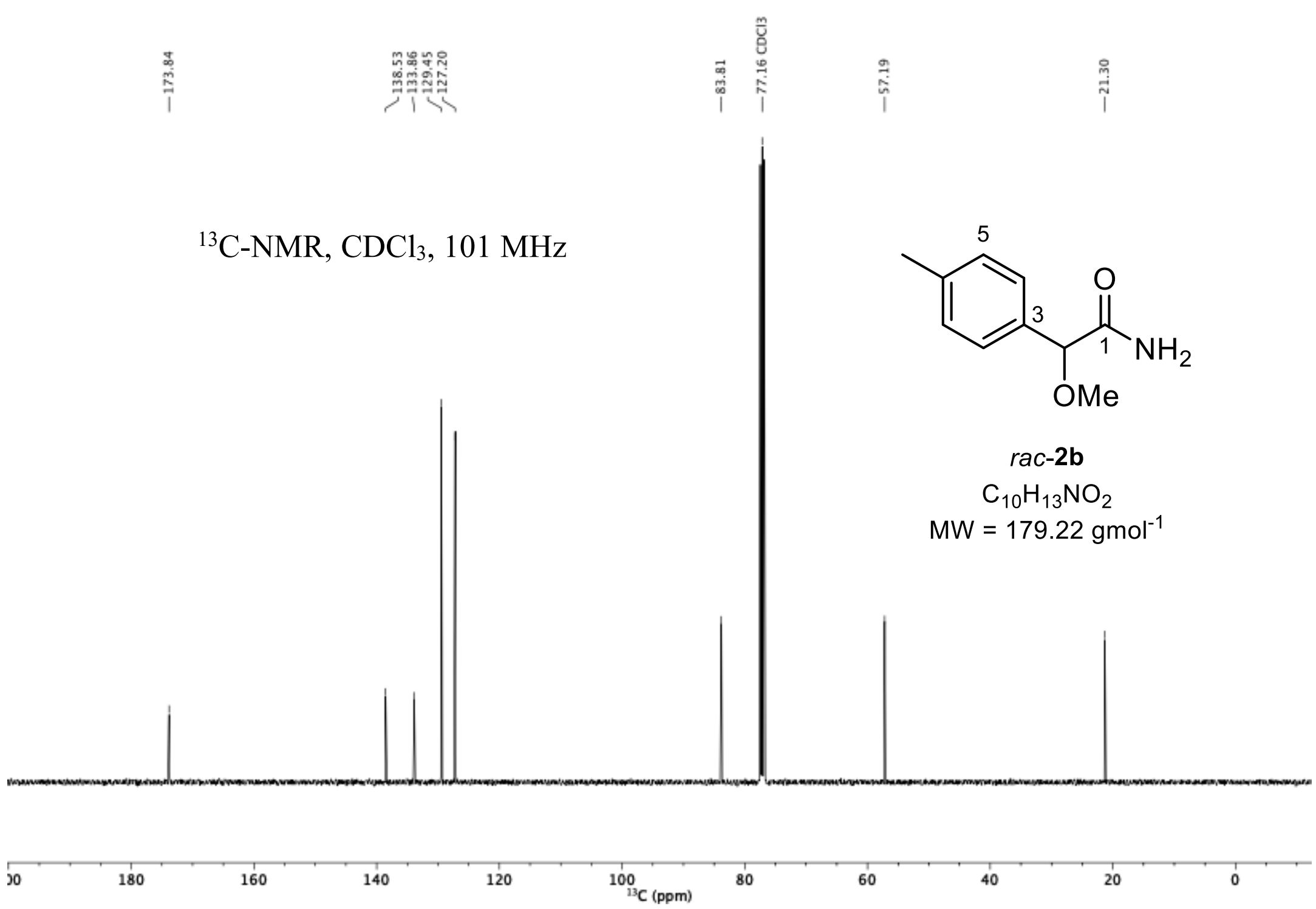
173.84
138.53
133.86
129.45
127.20
83.81
77.16 CDCl3
57.19
21.30
13C-NMR, CDCl3, 101 MHz
5
3
1
O
NH2
OMe
rac-2b
C10H13NO2
MW = 179.22 gmol-1
180
160
140
120
100
80
60
40
20
0
13C (ppm)

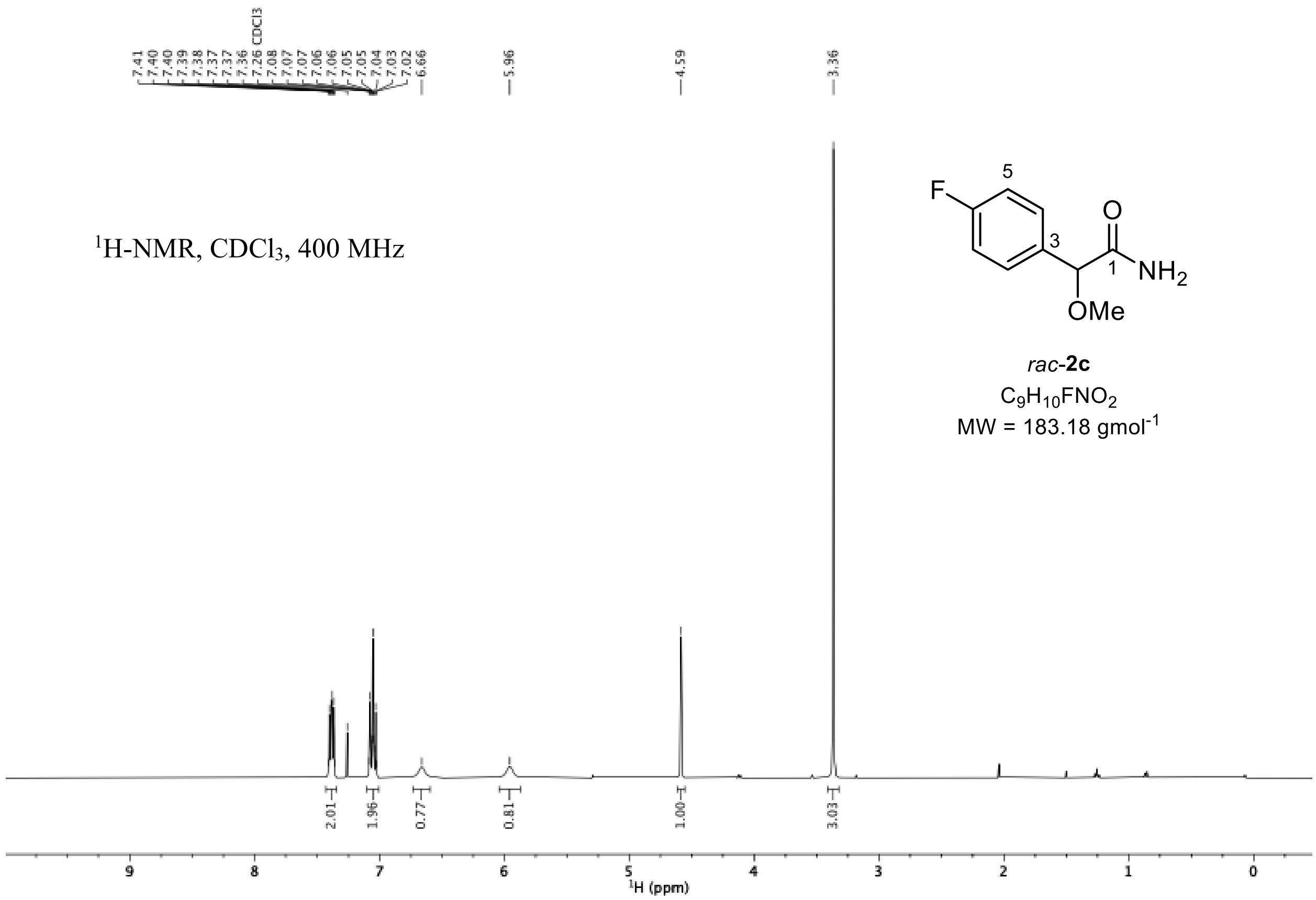

rac-**2c**

$C_9H_{10}FNO_2$

MW = 183.18 $gmol^{-1}$

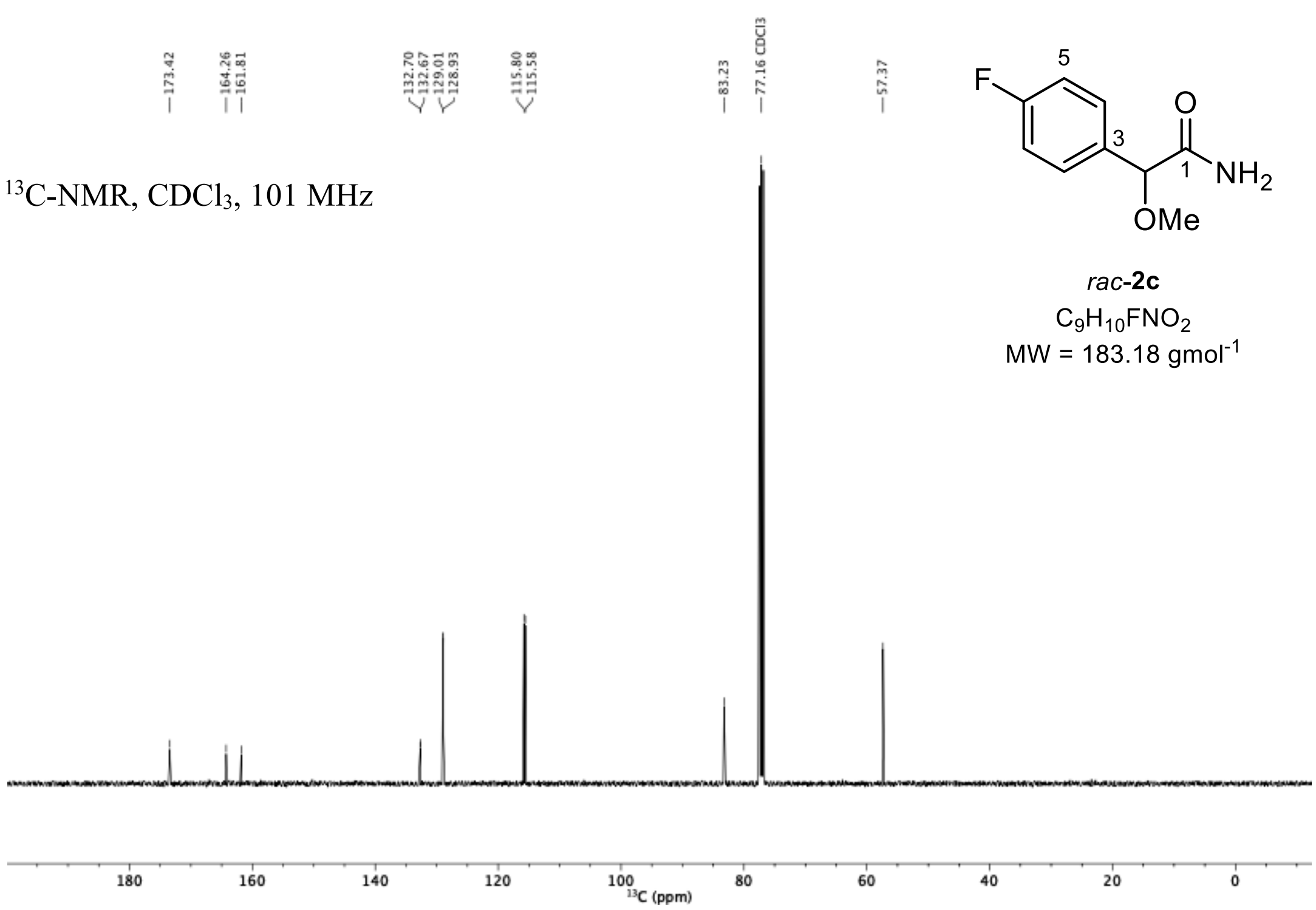

rac-**2c**

$C_9H_{10}FNO_2$

MW = 183.18 $gmol^{-1}$

^{19}F-NMR, $CDCl_3$, 376 MHz

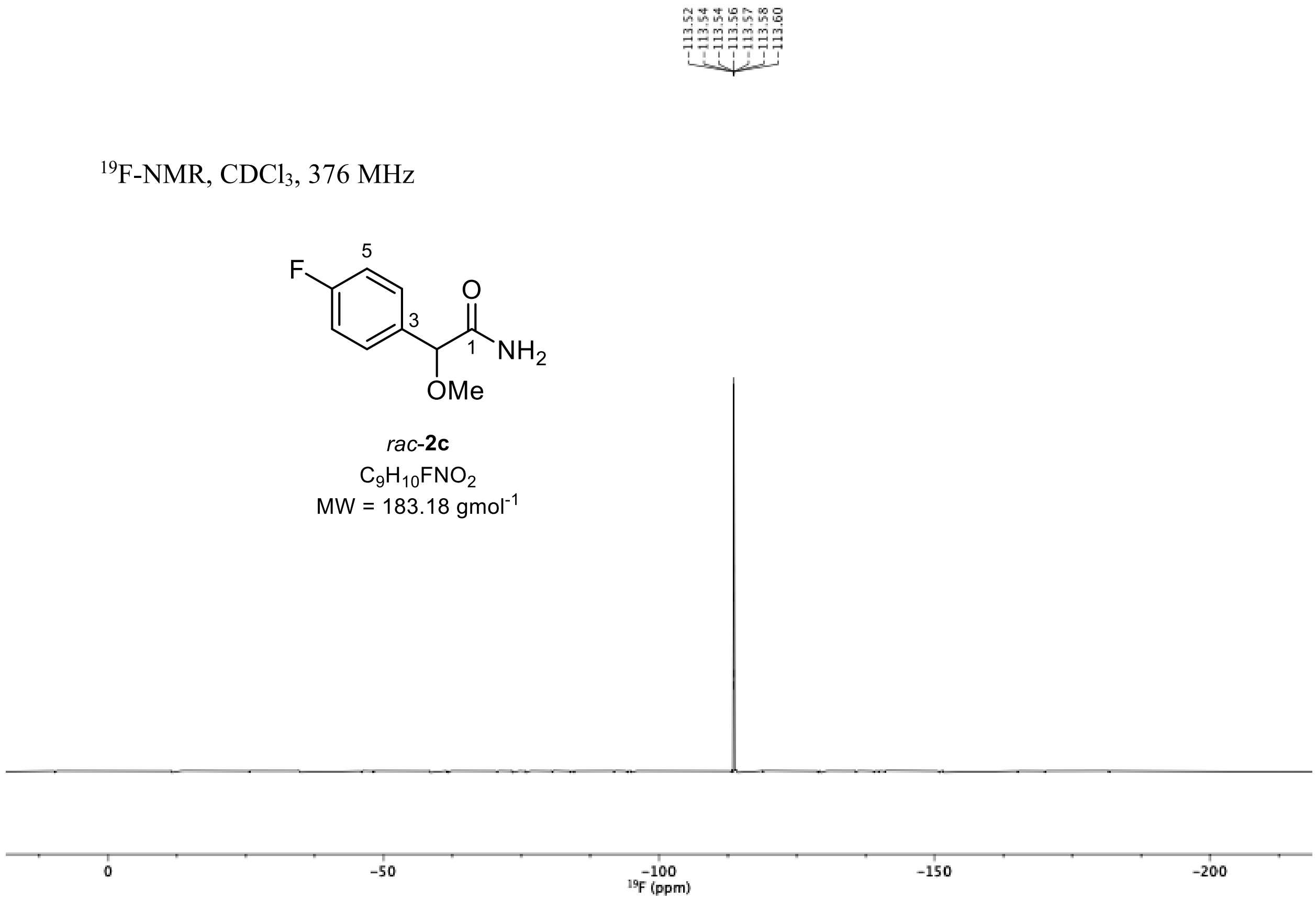

rac-**2c**

$C_9H_{10}FNO_2$

MW = 183.18 $gmol^{-1}$

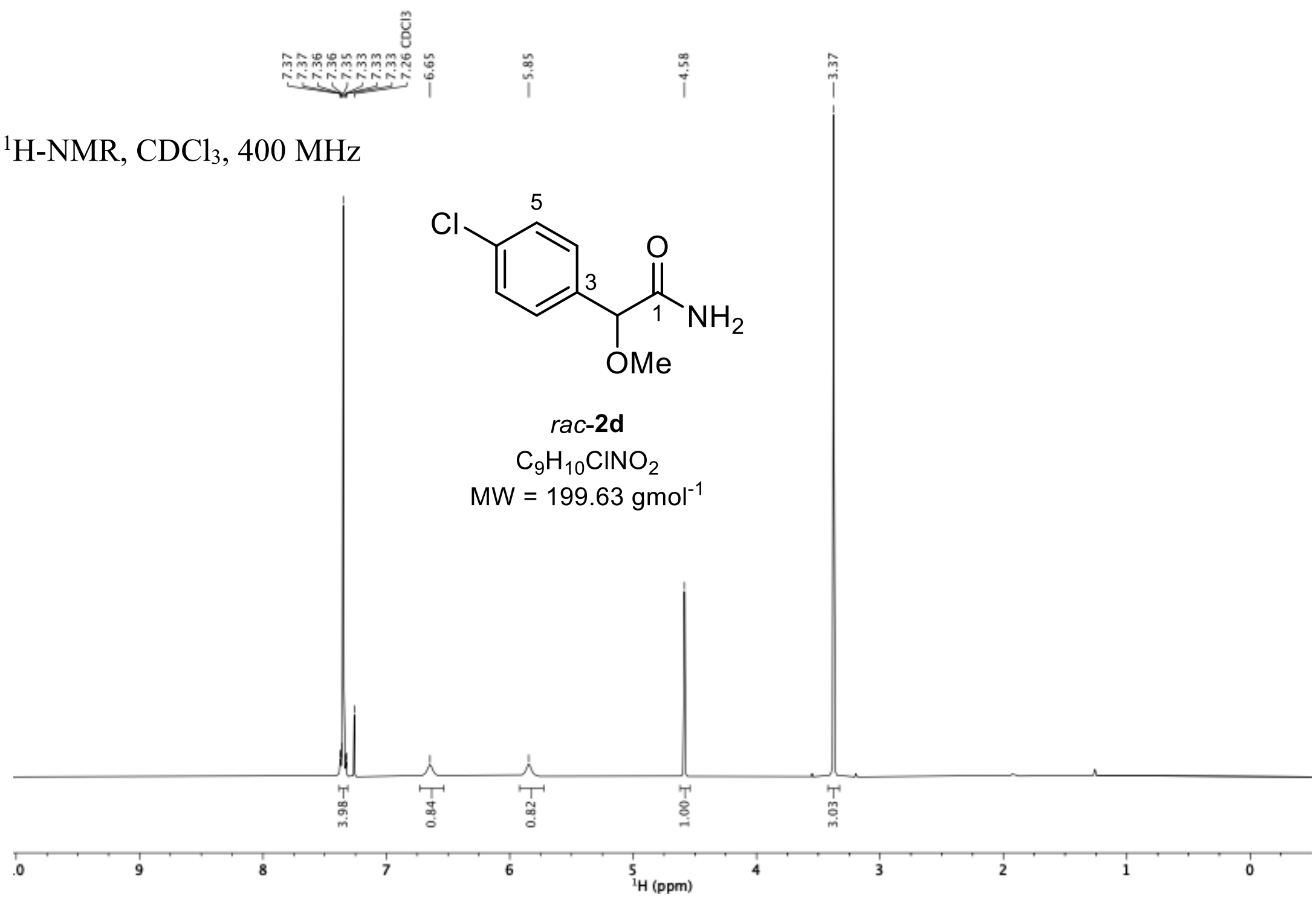

7.37
7.37
7.36
7.35
7.33
7.33
7.33
7.26 CDCl3
6.65
5.85
4.58
3.37
1H-NMR, CDCl3, 400 MHz
Cl
5
3
O
1
NH2
OMe
rac-2d
C9H10ClNO2
MW = 199.63 gmol-1
3.98
0.84
0.82
1.00
3.03
1H (ppm)

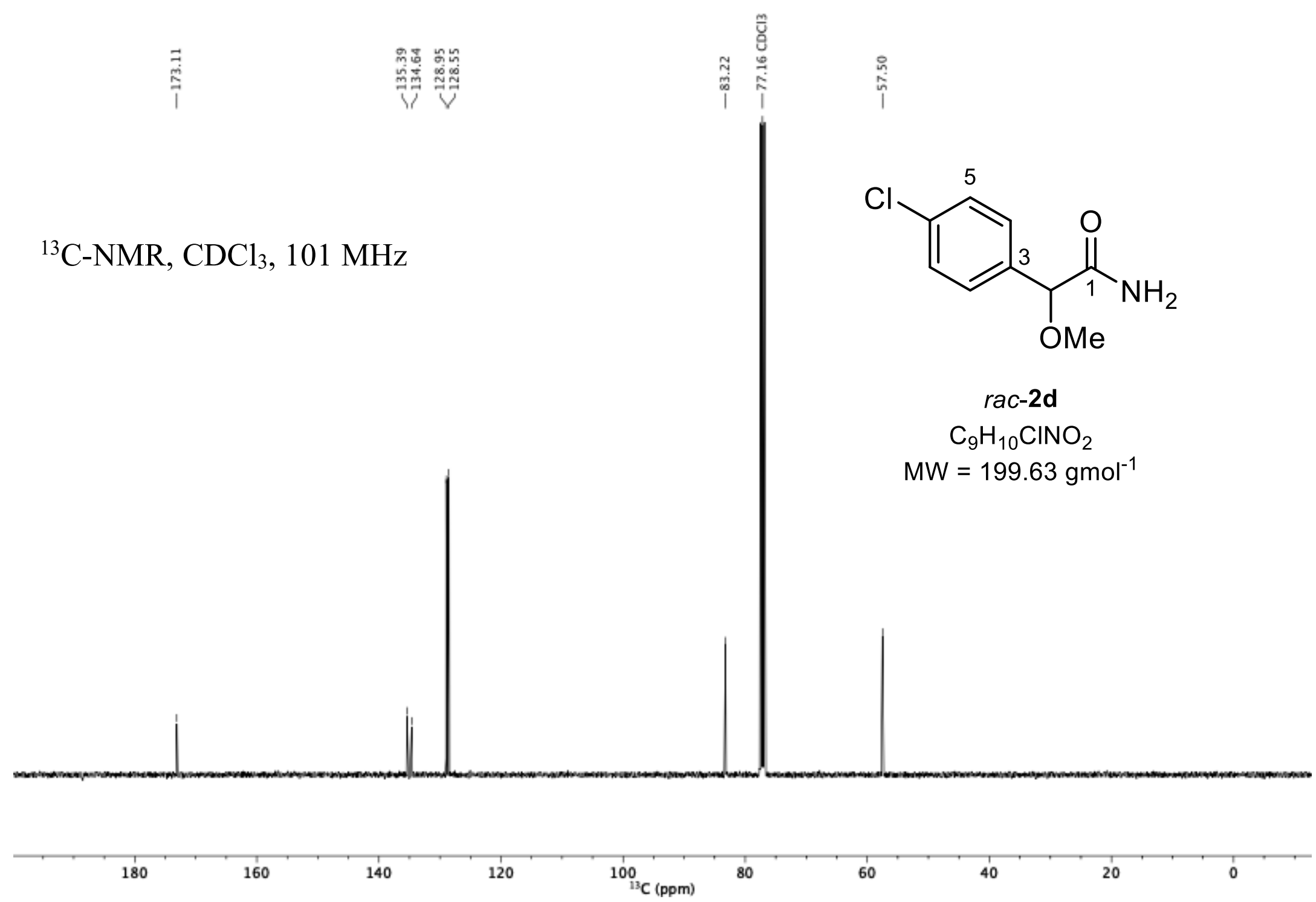

173.11
135.39
134.64
128.95
128.55
83.22
77.16 CDCl3
57.50
13C-NMR, CDCl3, 101 MHz
Cl
5
3
O
1
NH2
OMe
rac-2d
C9H10ClNO2
MW = 199.63 gmol-1
13C (ppm)

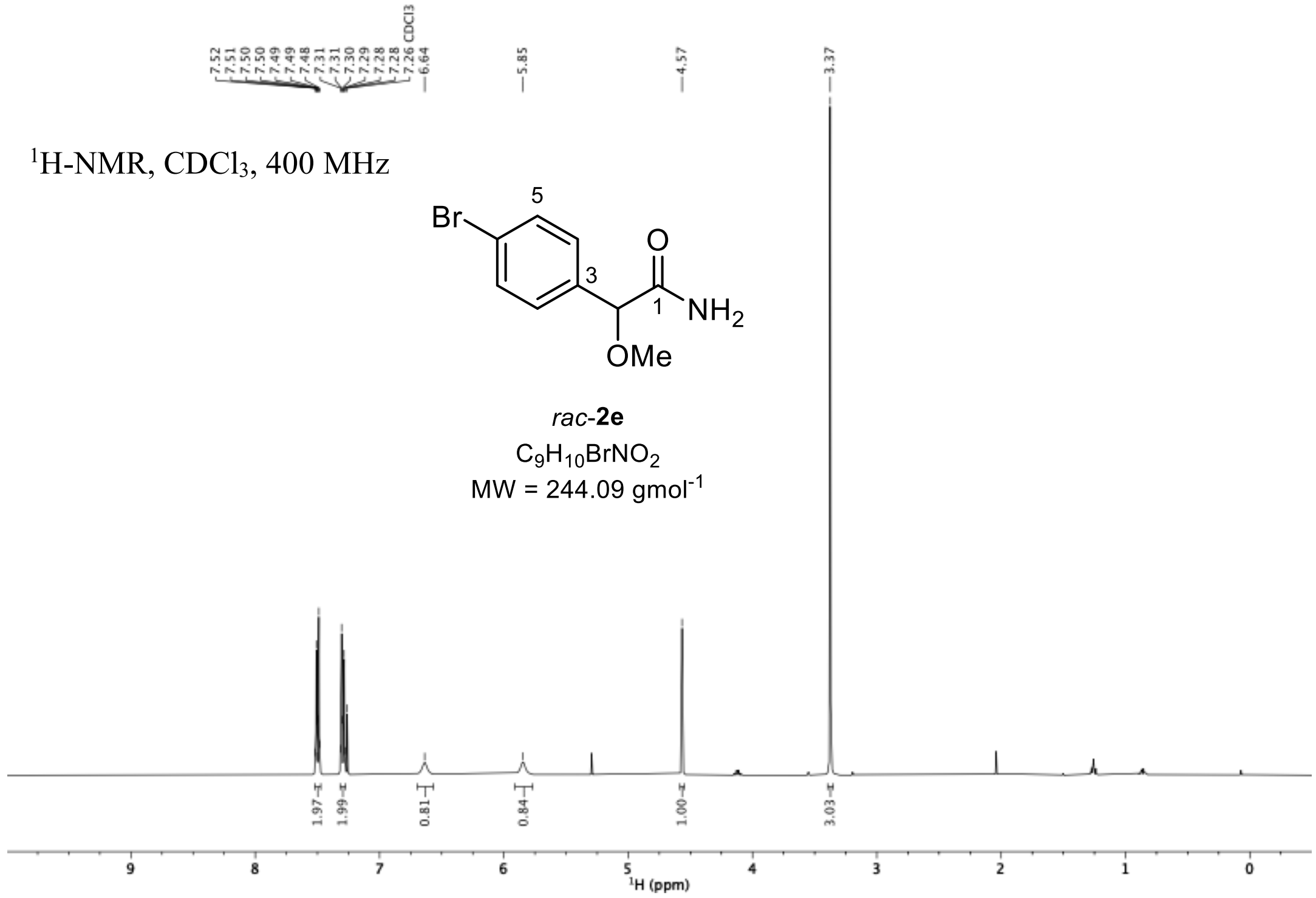
7.52 7.51 7.50 7.50 7.49 7.49 7.48 7.31 7.31 7.30 7.29 7.28 7.28 7.26 CDCl3
6.64
5.85
4.57
3.37
1H-NMR, CDCl3, 400 MHz
Br
5
3
O
1
NH2
OMe
rac-2e
C9H10BrNO2
MW = 244.09 gmol-1
1.97
1.99
0.81
0.84
1.00
3.03
1H (ppm)

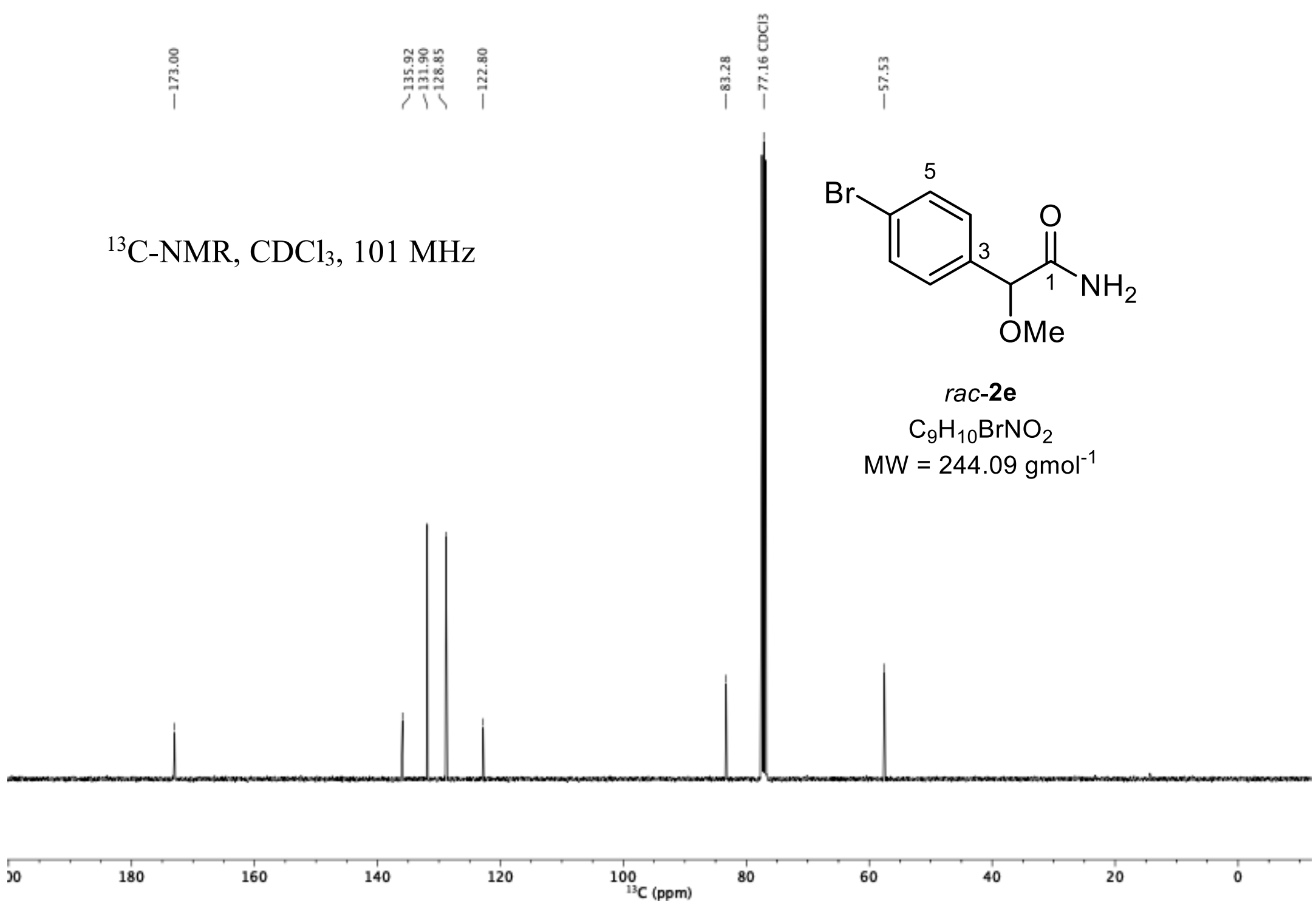
173.00
135.92
131.90
128.85
122.80
83.28
77.16 CDCl3
57.53
13C-NMR, CDCl3, 101 MHz
Br
5
3
O
1
NH2
OMe
rac-2e
C9H10BrNO2
MW = 244.09 gmol-1
13C (ppm)

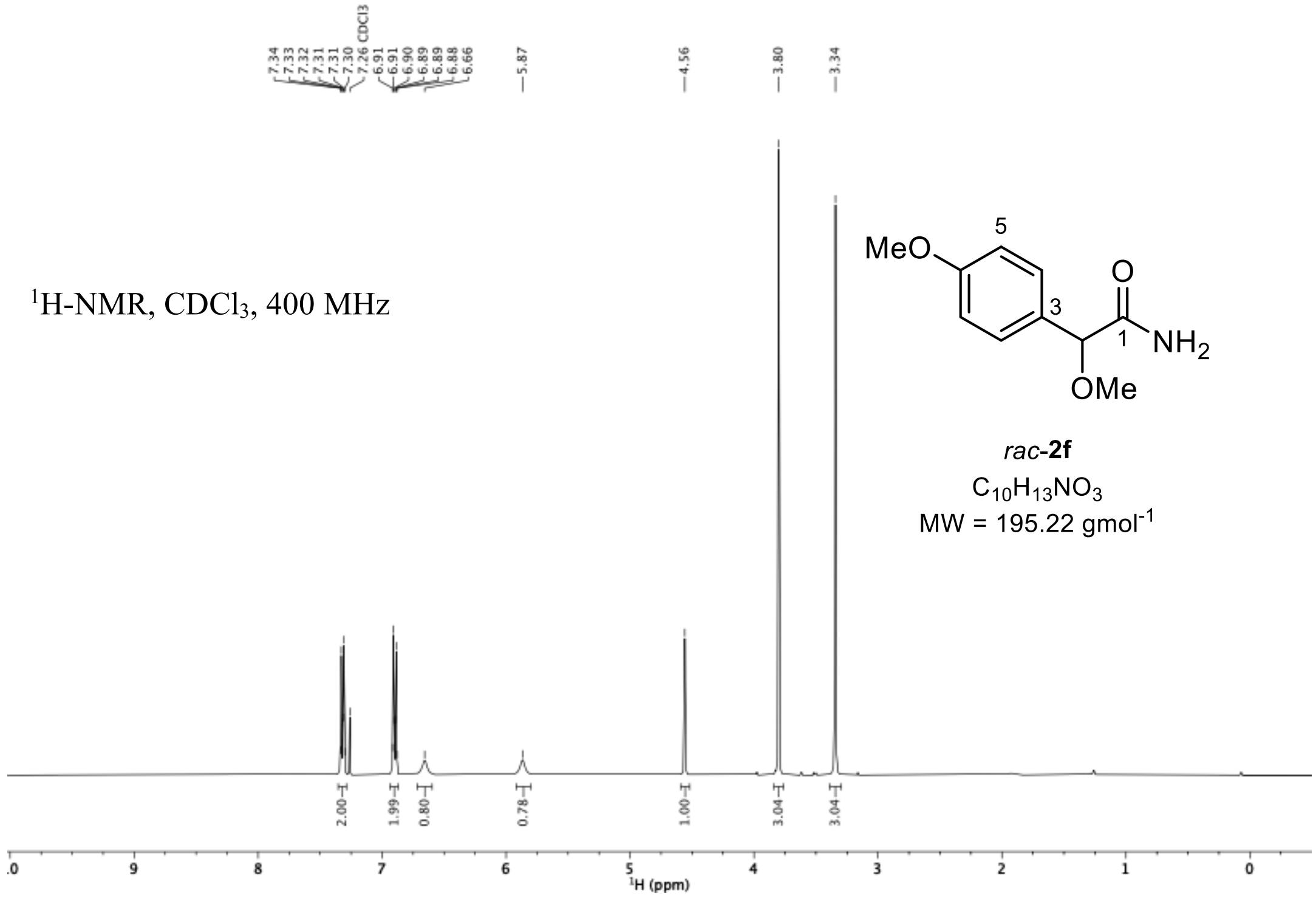
7.34
7.33
7.32
7.31
7.31
7.30
7.26 CDCl3
6.91
6.91
6.90
6.89
6.89
6.88
6.66
5.87
4.56
3.80
3.34
1H-NMR, CDCl3, 400 MHz
MeO
5
3
1
O
NH2
OMe
rac-2f
C10H13NO3
MW = 195.22 gmol-1
2.00
1.99
0.80
0.78
1.00
3.04
3.04
1H (ppm)

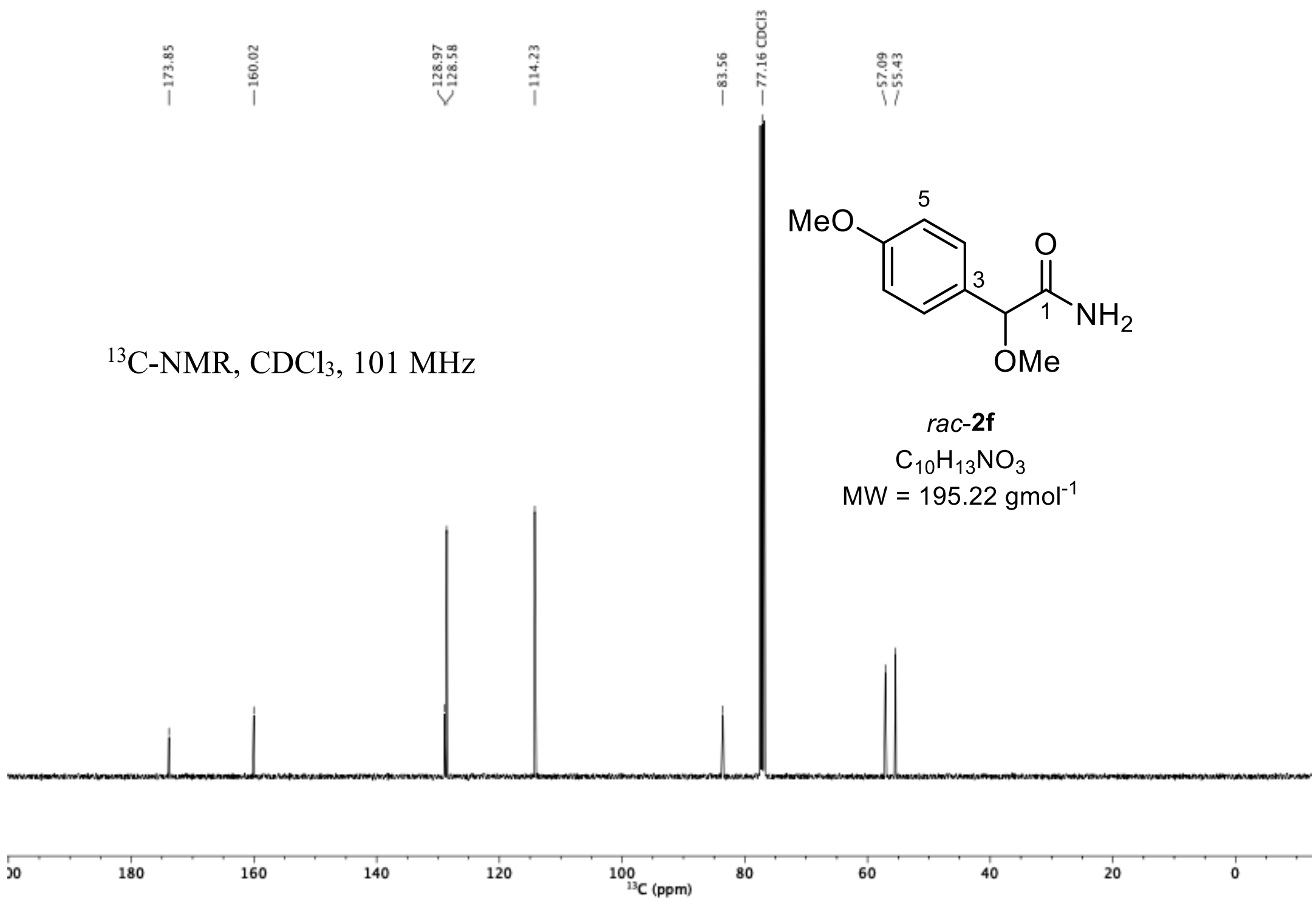
173.85
160.02
128.97
128.58
114.23
83.56
77.16 CDCl3
57.09
55.43
13C-NMR, CDCl3, 101 MHz
MeO
5
3
1
O
NH2
OMe
rac-2f
C10H13NO3
MW = 195.22 gmol-1
13C (ppm)

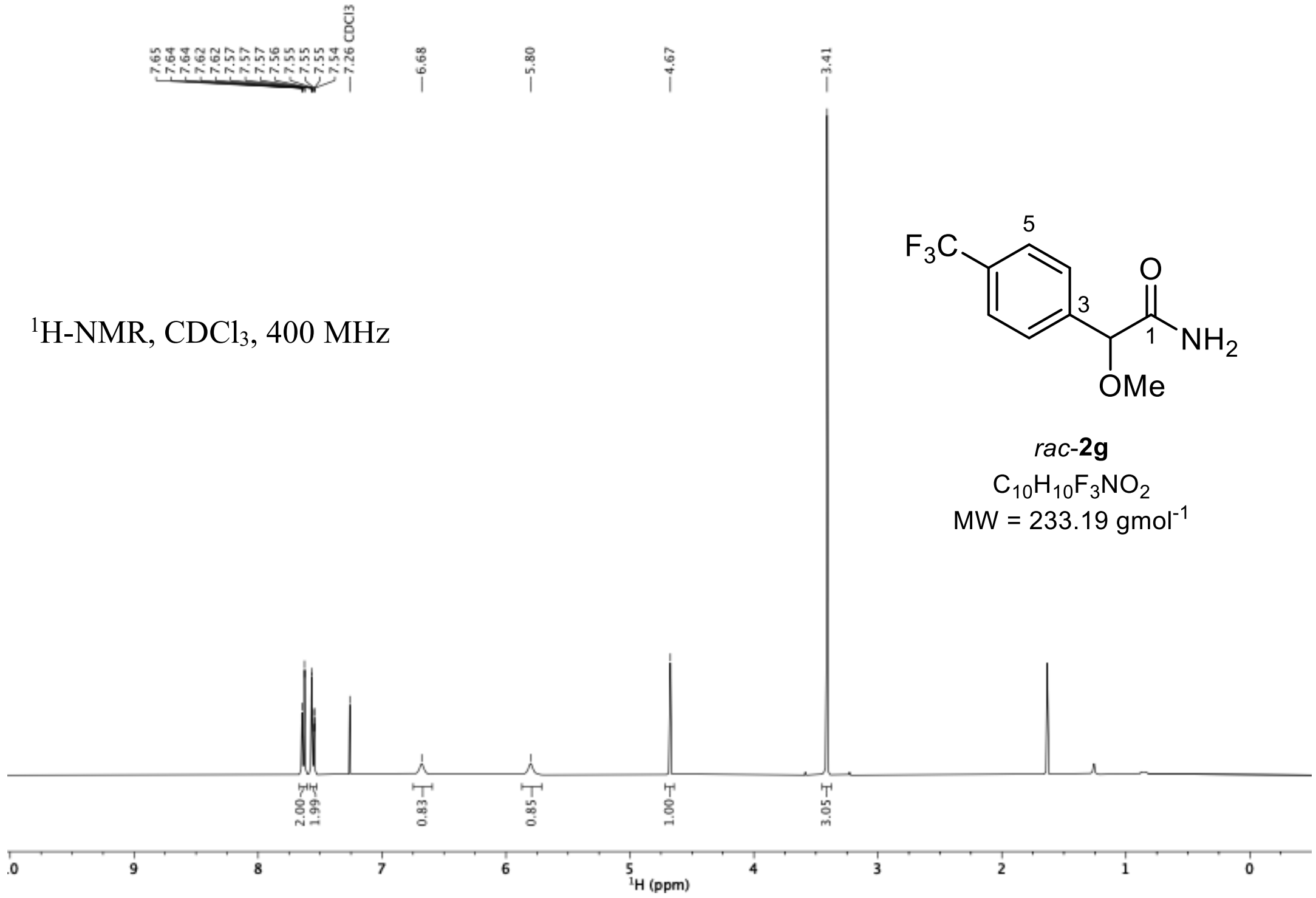

7.65
7.64
7.64
7.62
7.62
7.57
7.57
7.57
7.56
7.55
7.55
7.55
7.54
7.26 CDCl3
6.68
5.80
4.67
3.41
1H-NMR, CDCl3, 400 MHz
F3C
5
3
1
O
NH2
OMe
rac-2g
C10H10F3NO2
MW = 233.19 gmol-1
2.00
1.99
0.83
0.85
1.00
3.05
1H (ppm)

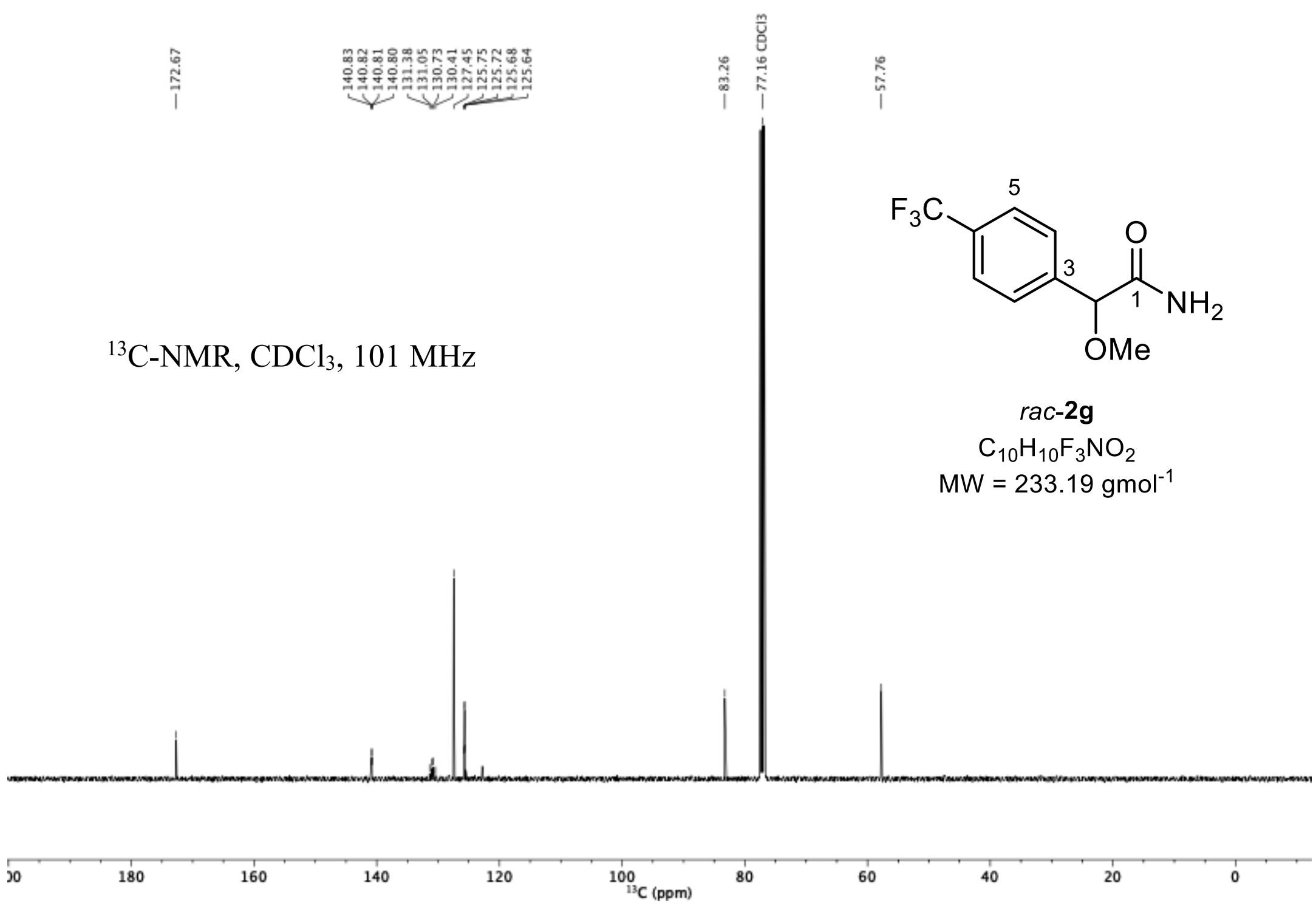

172.67
140.83
140.82
140.81
140.80
131.38
131.05
130.73
130.41
127.45
125.75
125.72
125.68
125.64
83.26
77.16 CDCl3
57.76
13C-NMR, CDCl3, 101 MHz
F3C
5
3
1
O
NH2
OMe
rac-2g
C10H10F3NO2
MW = 233.19 gmol-1
13C (ppm)

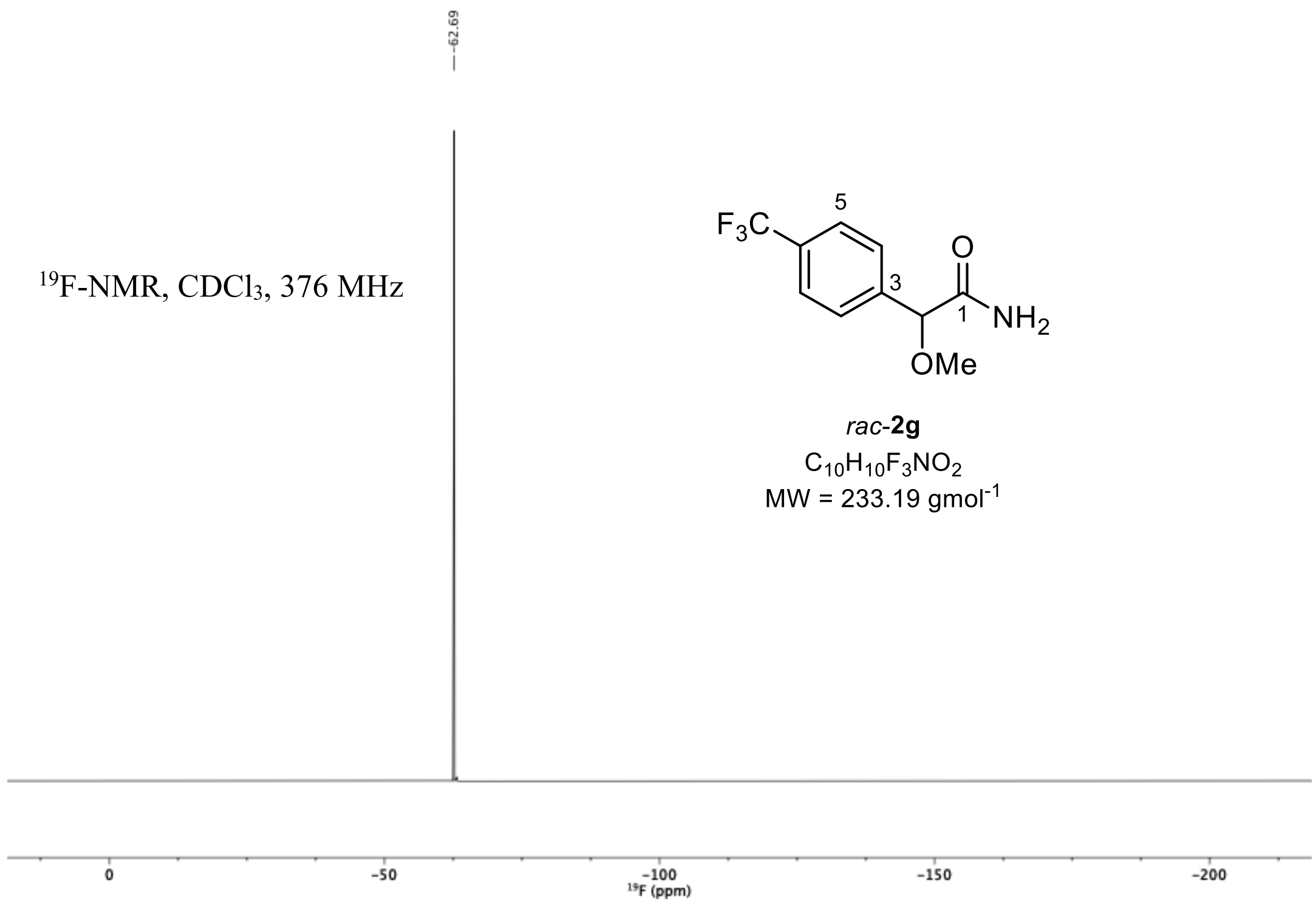

-62.69
19F-NMR, CDCl3, 376 MHz
F3C
5
3
O
1
NH2
OMe
rac-2g
C10H10F3NO2
MW = 233.19 gmol-1
0
-50
-100
-150
-200
19F (ppm)

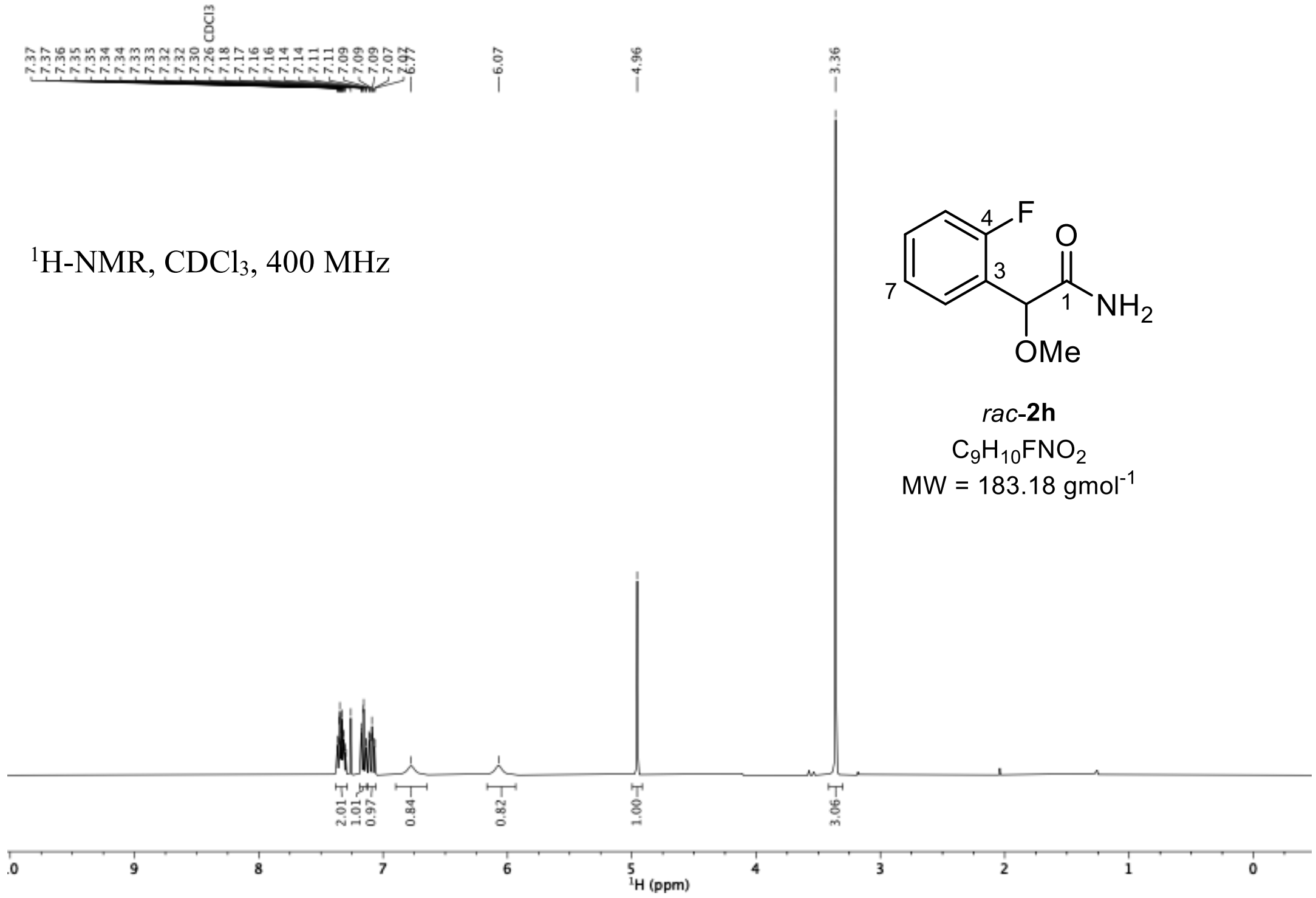

1H-NMR, CDCl3, 400 MHz
rac-2h
C9H10FNO2
MW = 183.18 gmol-1
7.37 7.37 7.36 7.35 7.35 7.34 7.34 7.33 7.33 7.32 7.32 7.30 7.26 CDCl3 7.18 7.17 7.16 7.16 7.14 7.14 7.11 7.11 7.09 7.09 7.09 7.07 7.07 6.77
6.07
4.96
3.36
2.01 1.01 0.97 0.84 0.82 1.00 3.06
1H (ppm)

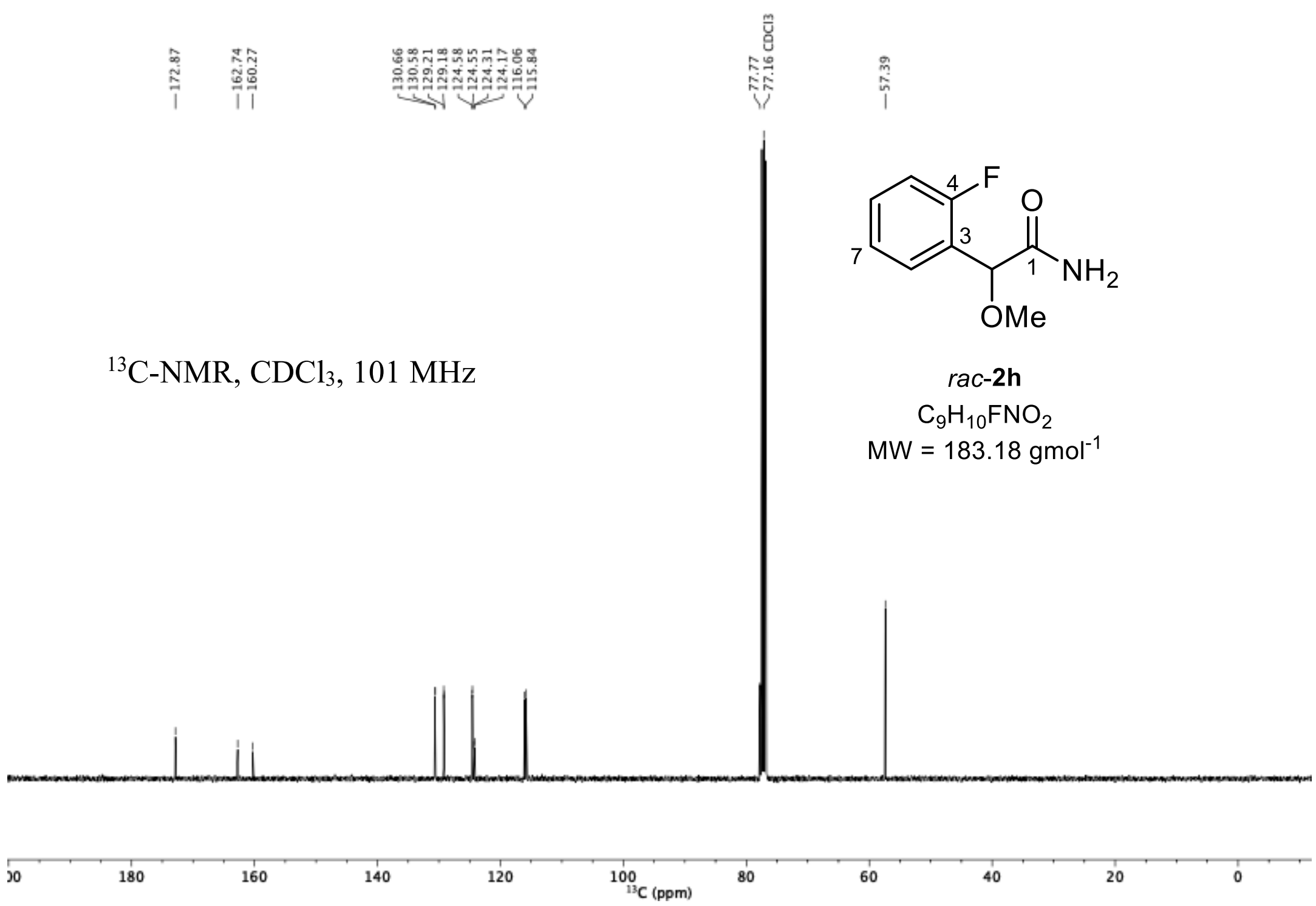

13C-NMR, CDCl3, 101 MHz
rac-2h
C9H10FNO2
MW = 183.18 gmol-1
172.87
162.74 160.27
130.66 130.58 129.21 129.18 124.58 124.55 124.31 124.17 116.06 115.84
77.77 77.16 CDCl3
57.39
13C (ppm)

^{19}F-NMR, CDCl$_3$, 376 MHz

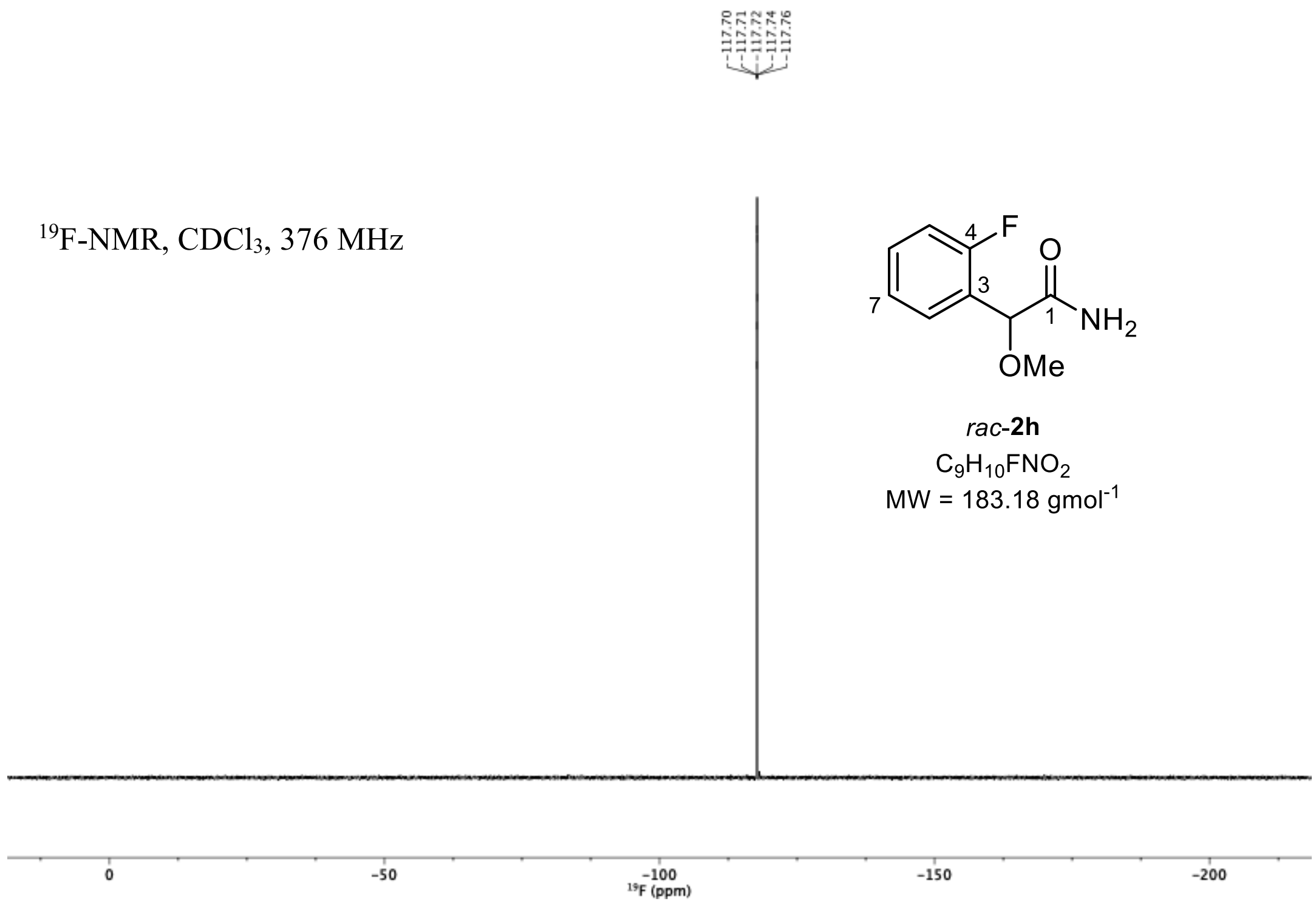

rac-**2h**

$C_9H_{10}FNO_2$

MW = 183.18 gmol^{-1}

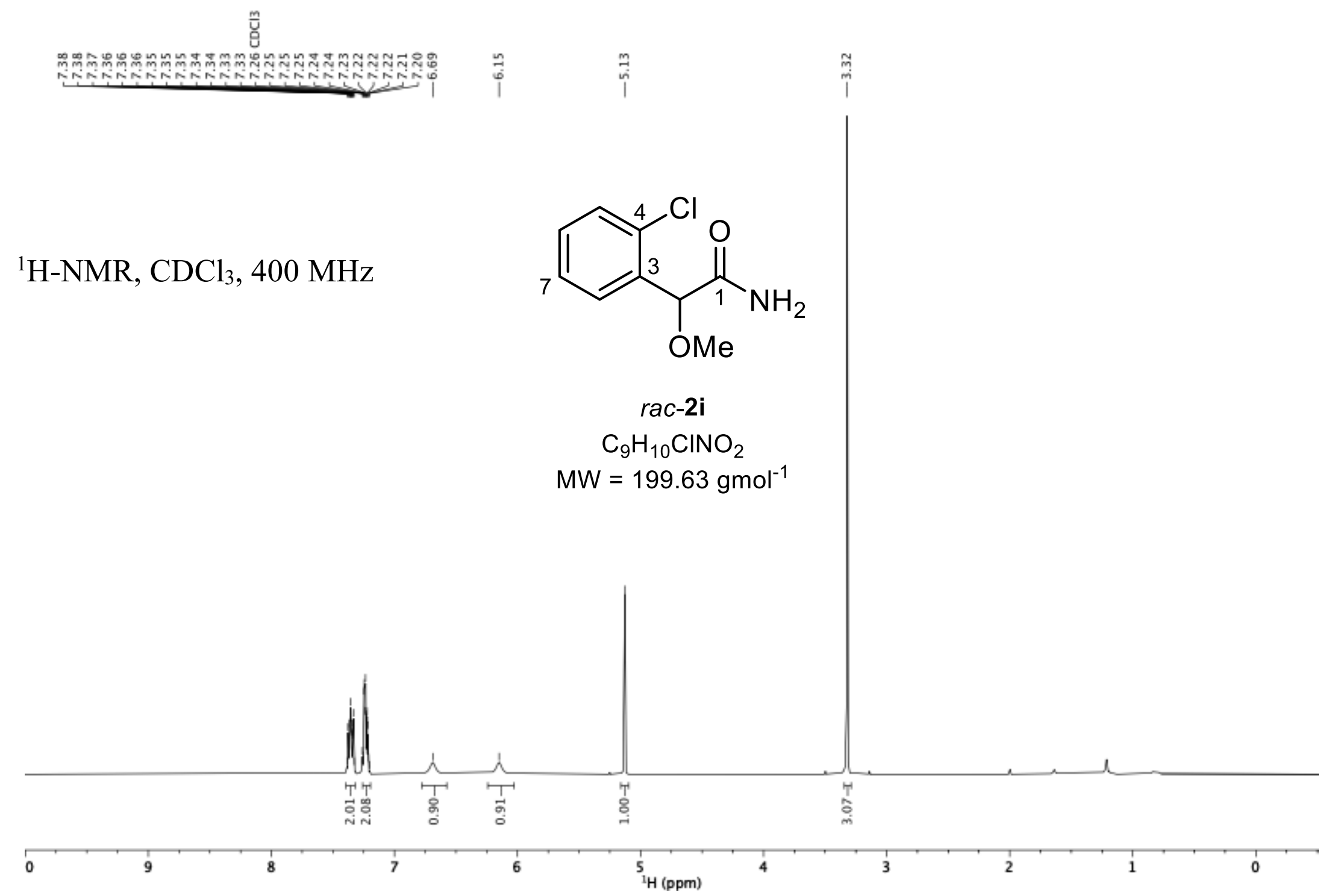

7.38
7.38
7.37
7.36
7.36
7.35
7.35
7.34
7.34
7.33
7.33
7.26 CDCl3
7.25
7.25
7.25
7.24
7.24
7.23
7.22
7.22
7.22
7.21
7.20
6.69
6.15
5.13
3.32
¹H-NMR, CDCl₃, 400 MHz
Cl
O
NH₂
OMe
4
3
1
7
rac-2i
C₉H₁₀ClNO₂
MW = 199.63 gmol⁻¹
2.01
2.08
0.90
0.91
1.00
3.07
¹H (ppm)

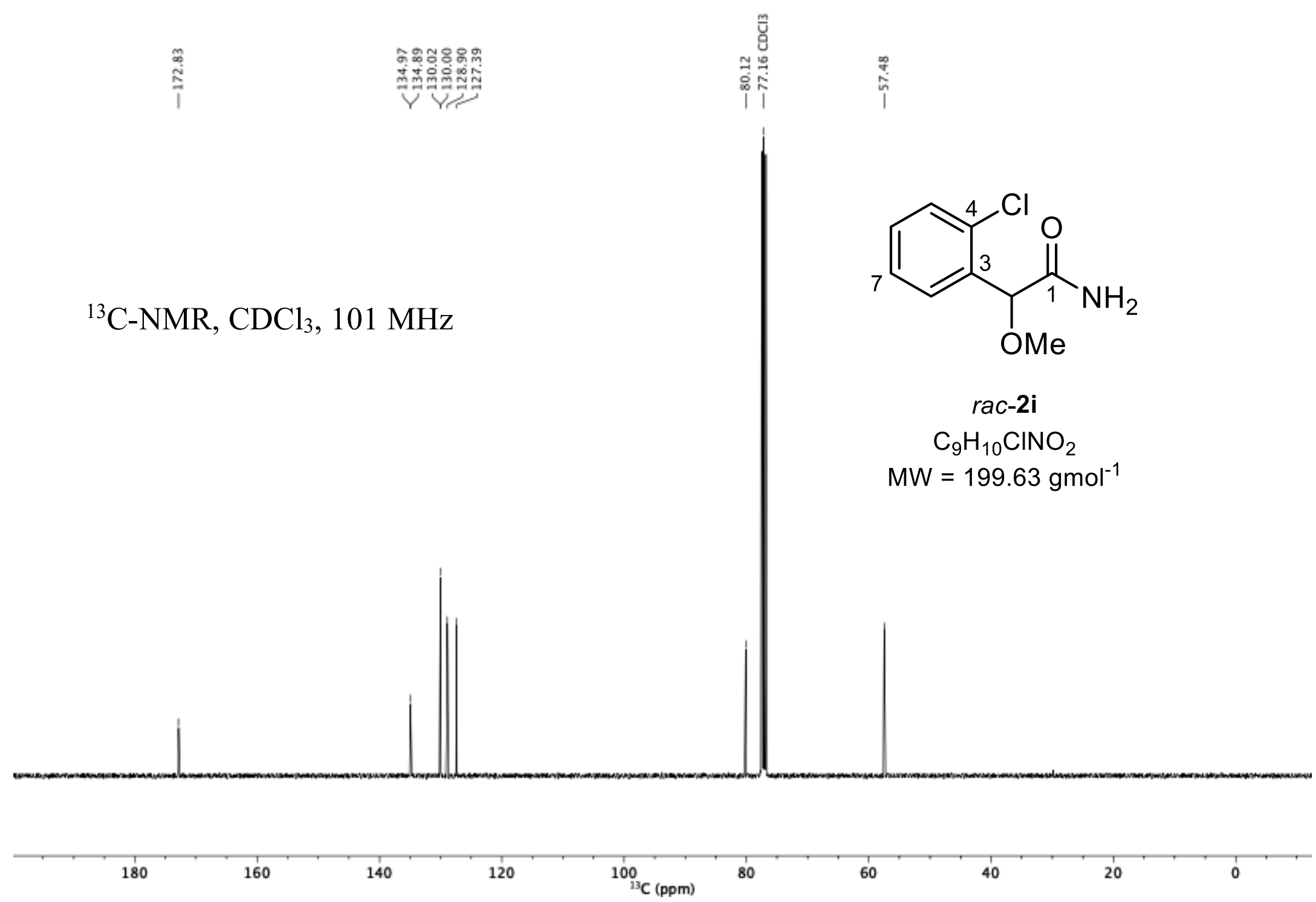

172.83
134.97
134.89
130.02
130.00
128.90
127.39
80.12
77.16 CDCl3
57.48
¹³C-NMR, CDCl₃, 101 MHz
Cl
O
NH₂
OMe
4
3
1
7
rac-2i
C₉H₁₀ClNO₂
MW = 199.63 gmol⁻¹
¹³C (ppm)

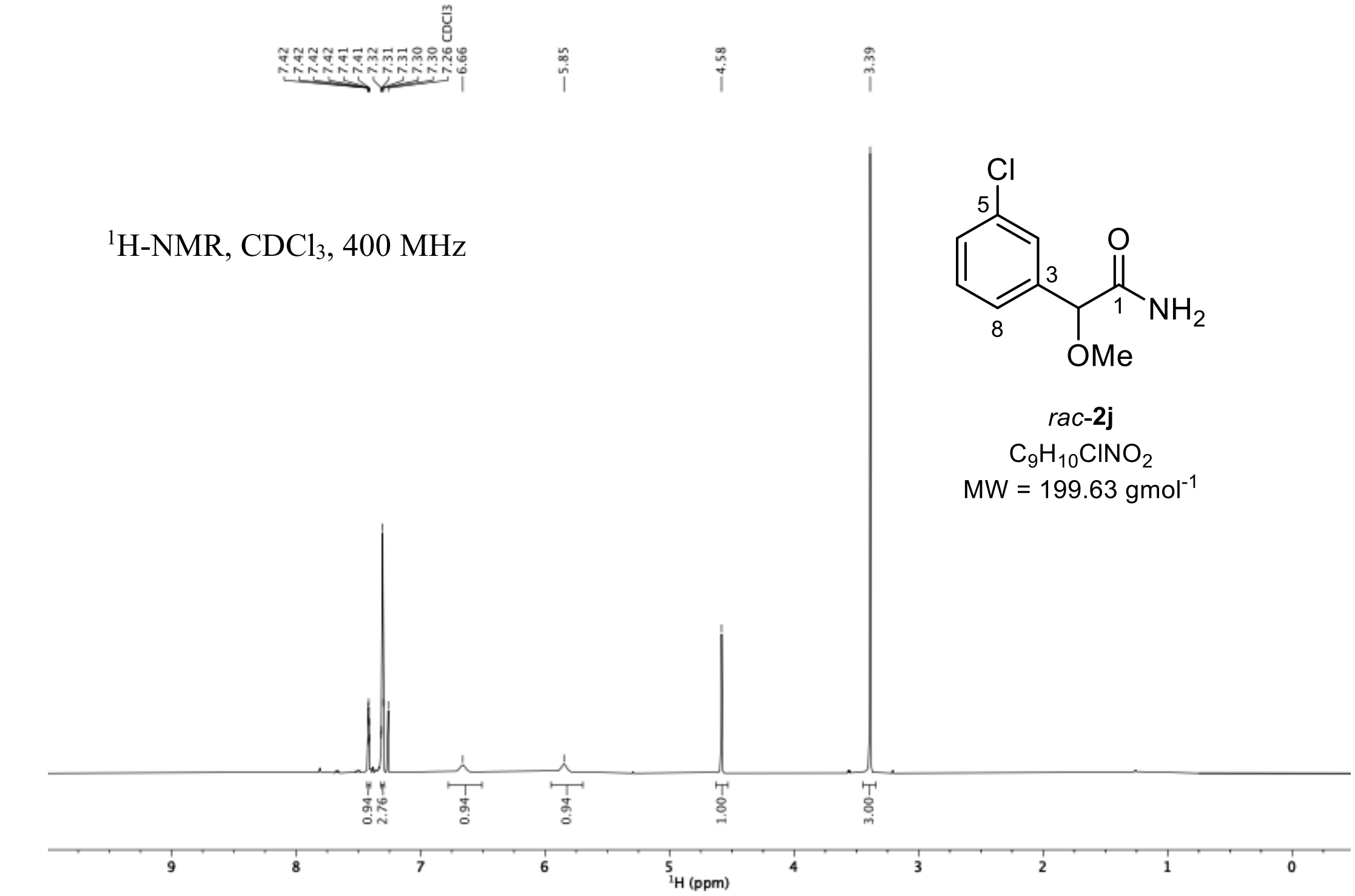

7.42
7.42
7.42
7.42
7.41
7.41
7.32
7.31
7.31
7.30
7.30
7.26 CDCl3
6.66
5.85
4.58
3.39
¹H-NMR, CDCl₃, 400 MHz
Cl
5
3
1
O
NH₂
8
OMe
rac-2j
C₉H₁₀ClNO₂
MW = 199.63 gmol⁻¹
0.94
2.76
0.94
0.94
1.00
3.00
9
8
7
6
5
4
3
2
1
0
¹H (ppm)

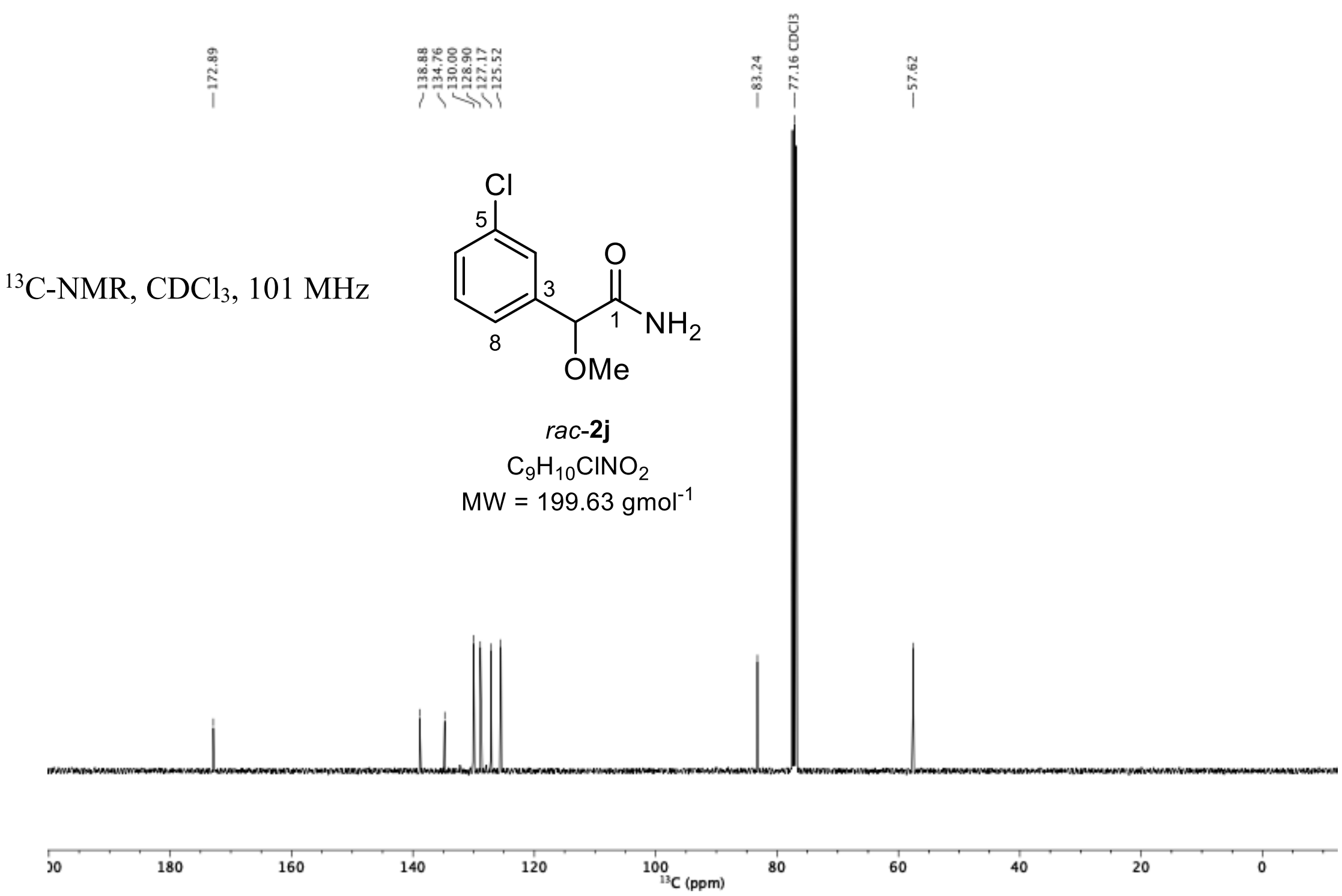

172.89
138.88
134.76
130.00
128.90
127.17
125.52
83.24
77.16 CDCl3
57.62
¹³C-NMR, CDCl₃, 101 MHz
Cl
5
3
1
O
NH₂
8
OMe
rac-2j
C₉H₁₀ClNO₂
MW = 199.63 gmol⁻¹
180
160
140
120
100
80
60
40
20
0
¹³C (ppm)

^{1}H-NMR, $CDCl_3$, 500 MHz

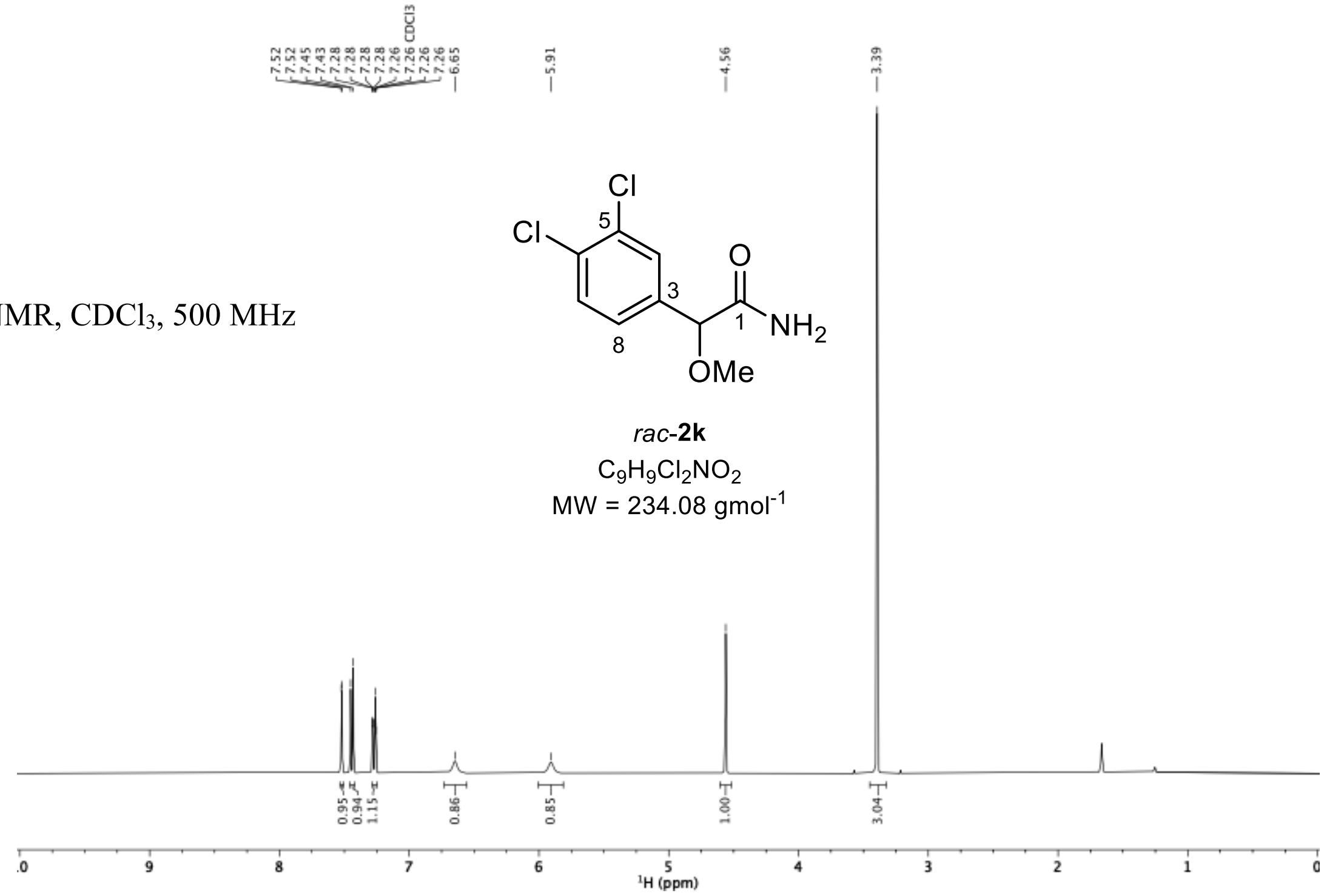

^{13}C-NMR, $CDCl_3$, 101 MHz

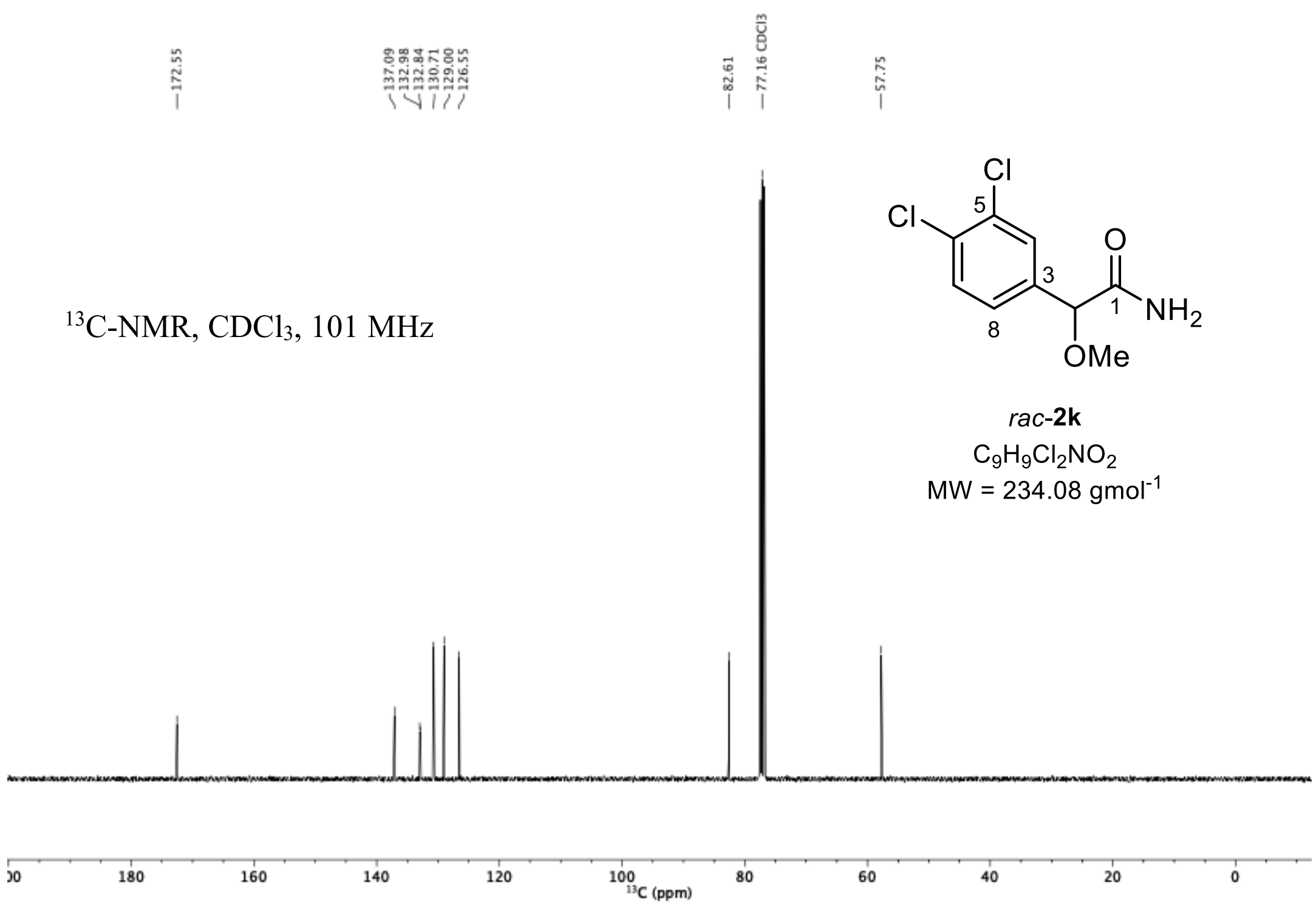

^{1}H-NMR, $CDCl_3$, 400 MHz

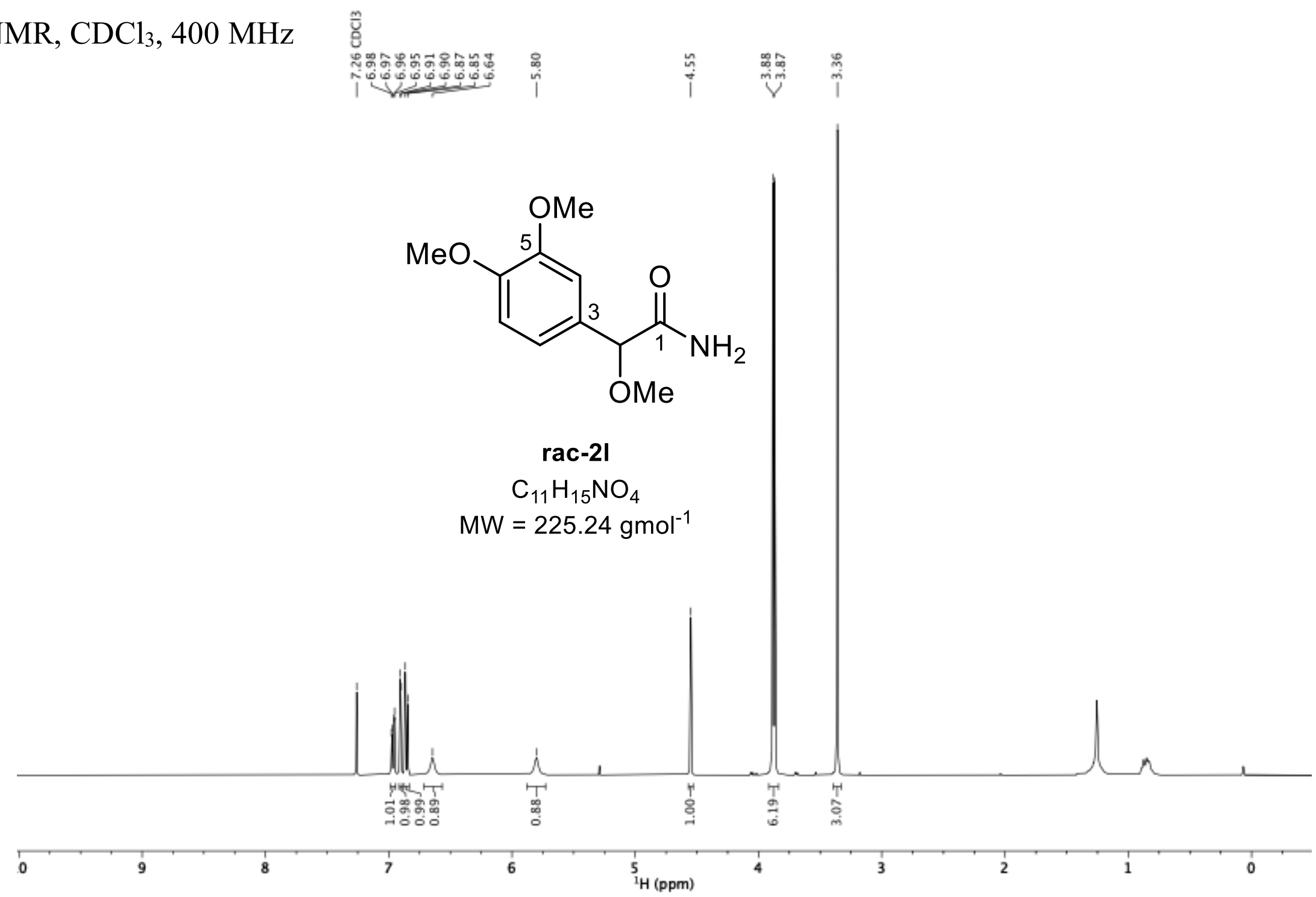

^{13}C-NMR, $CDCl_3$, 101 MHz

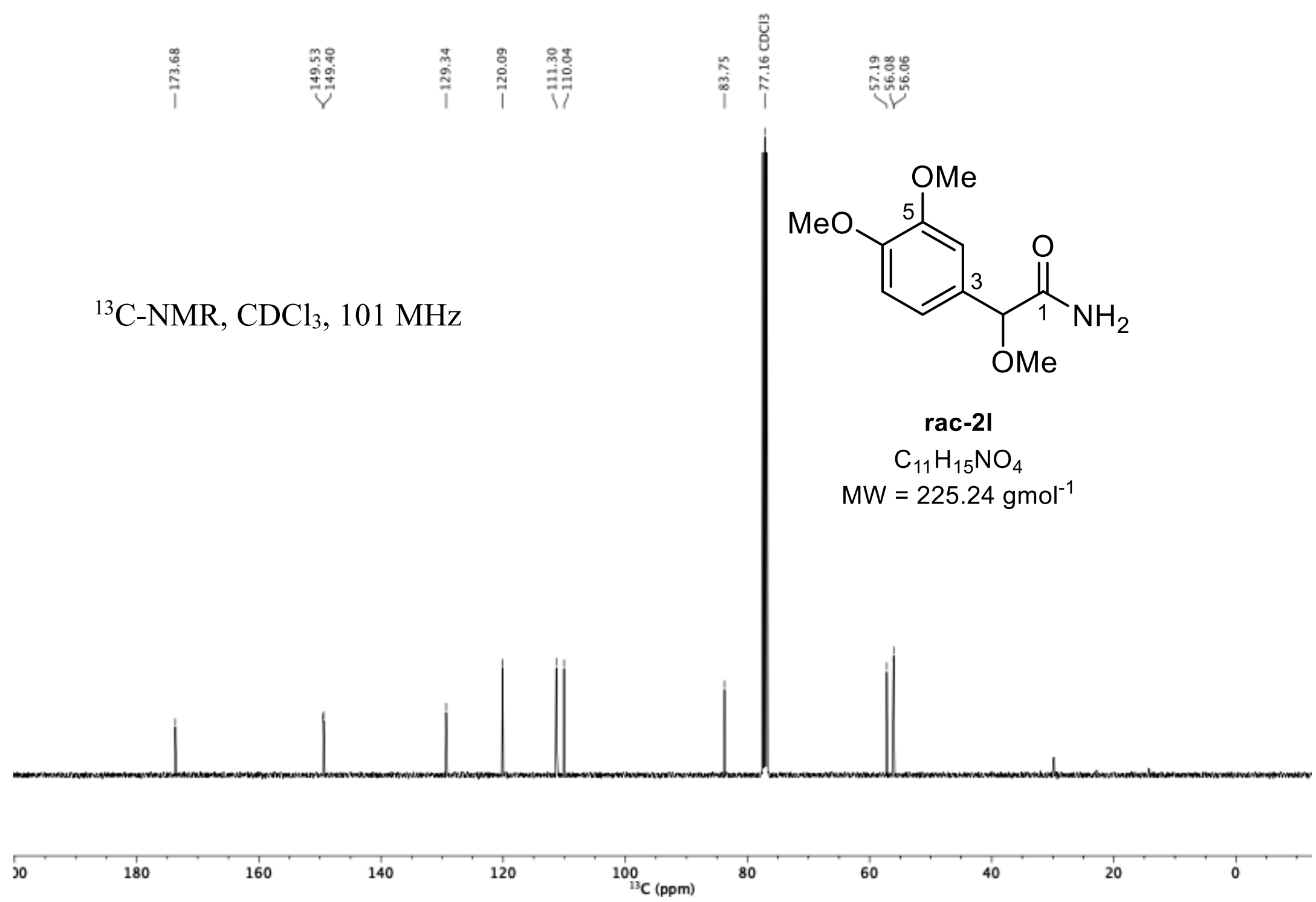

^{1}H-NMR, $CDCl_3$, 400 MHz

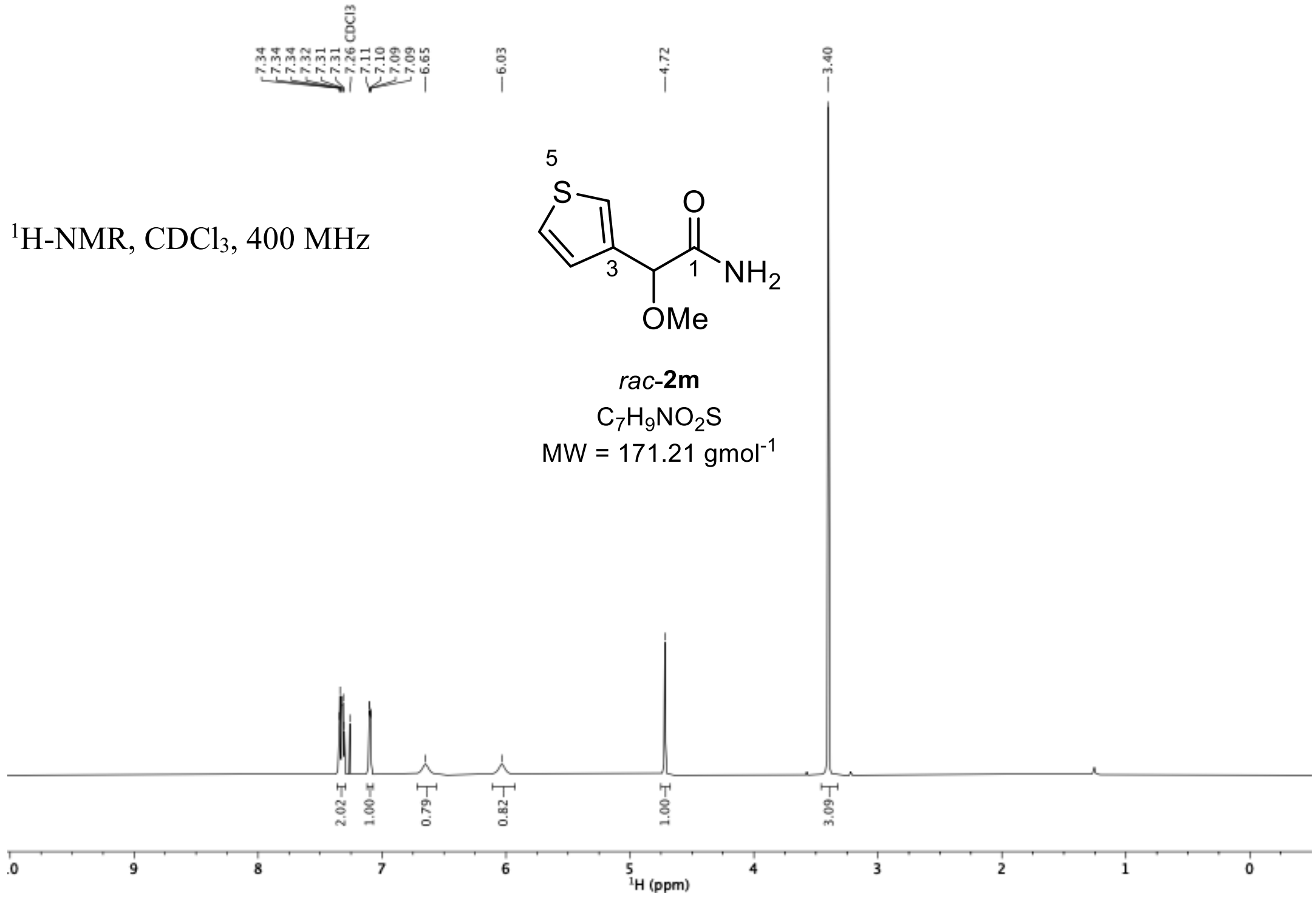

^{13}C-NMR, $CDCl_3$, 101 MHz

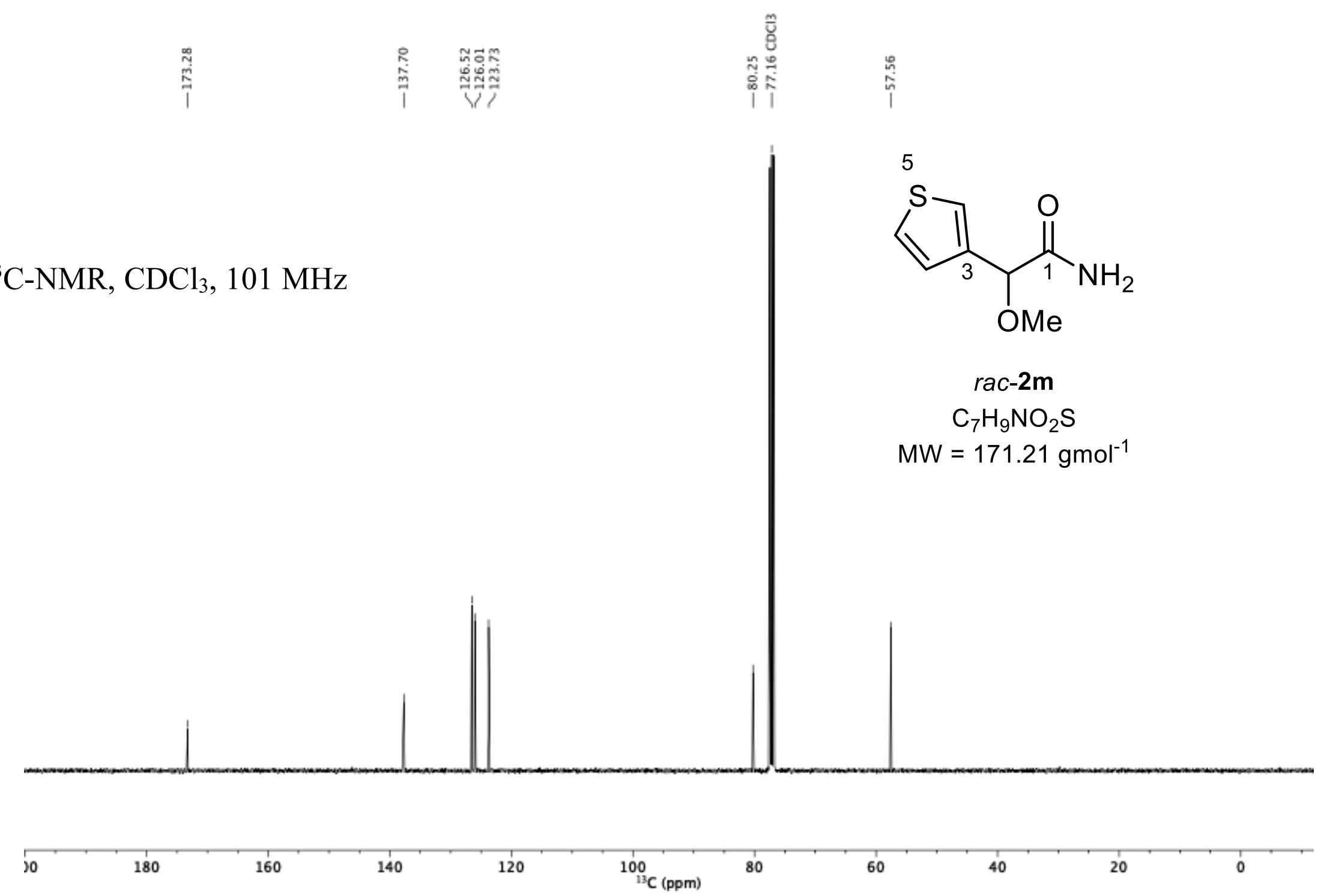

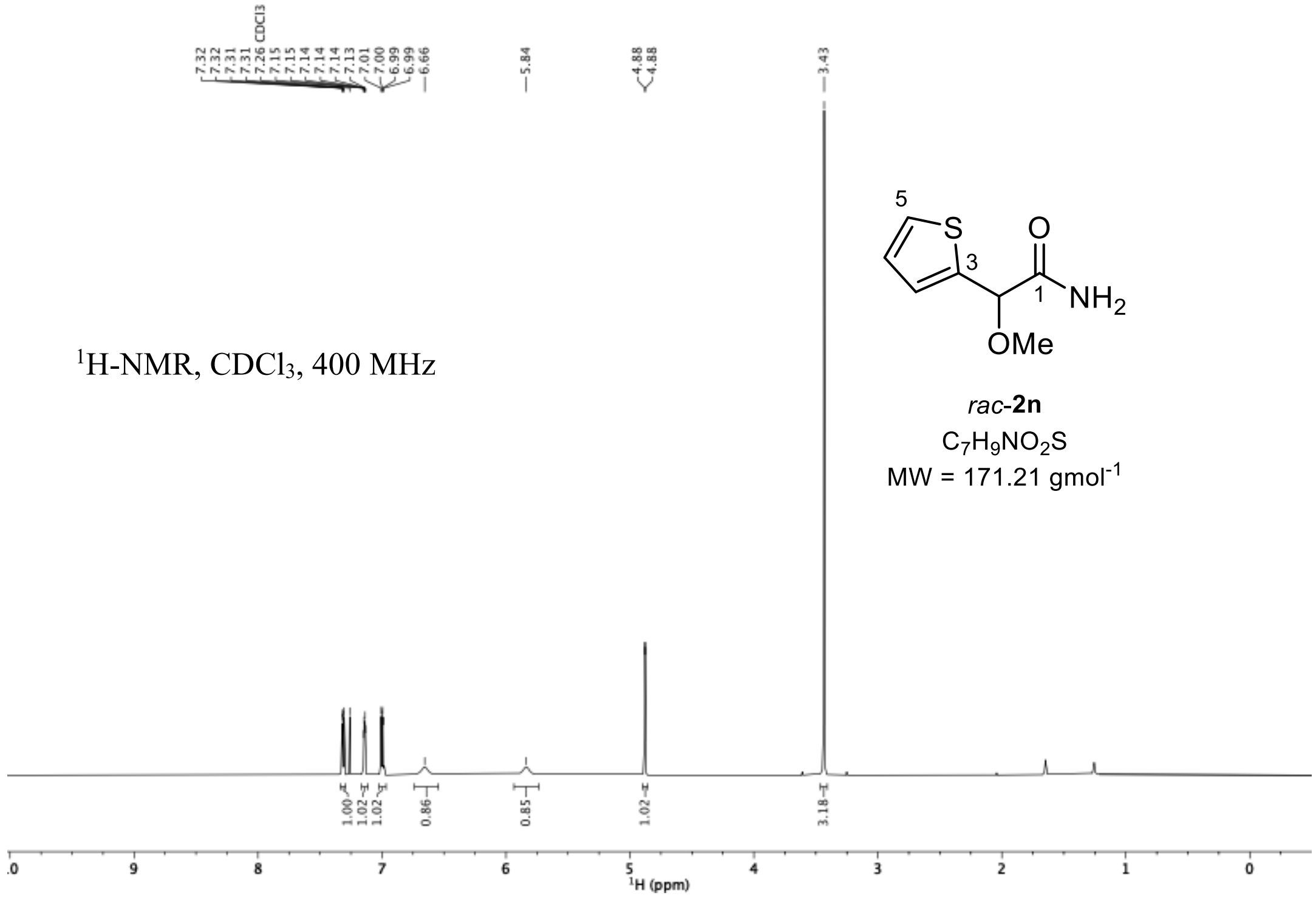
1H-NMR, CDCl3, 400 MHz
rac-2n
C7H9NO2S
MW = 171.21 gmol-1
NH2
OMe
1H (ppm)

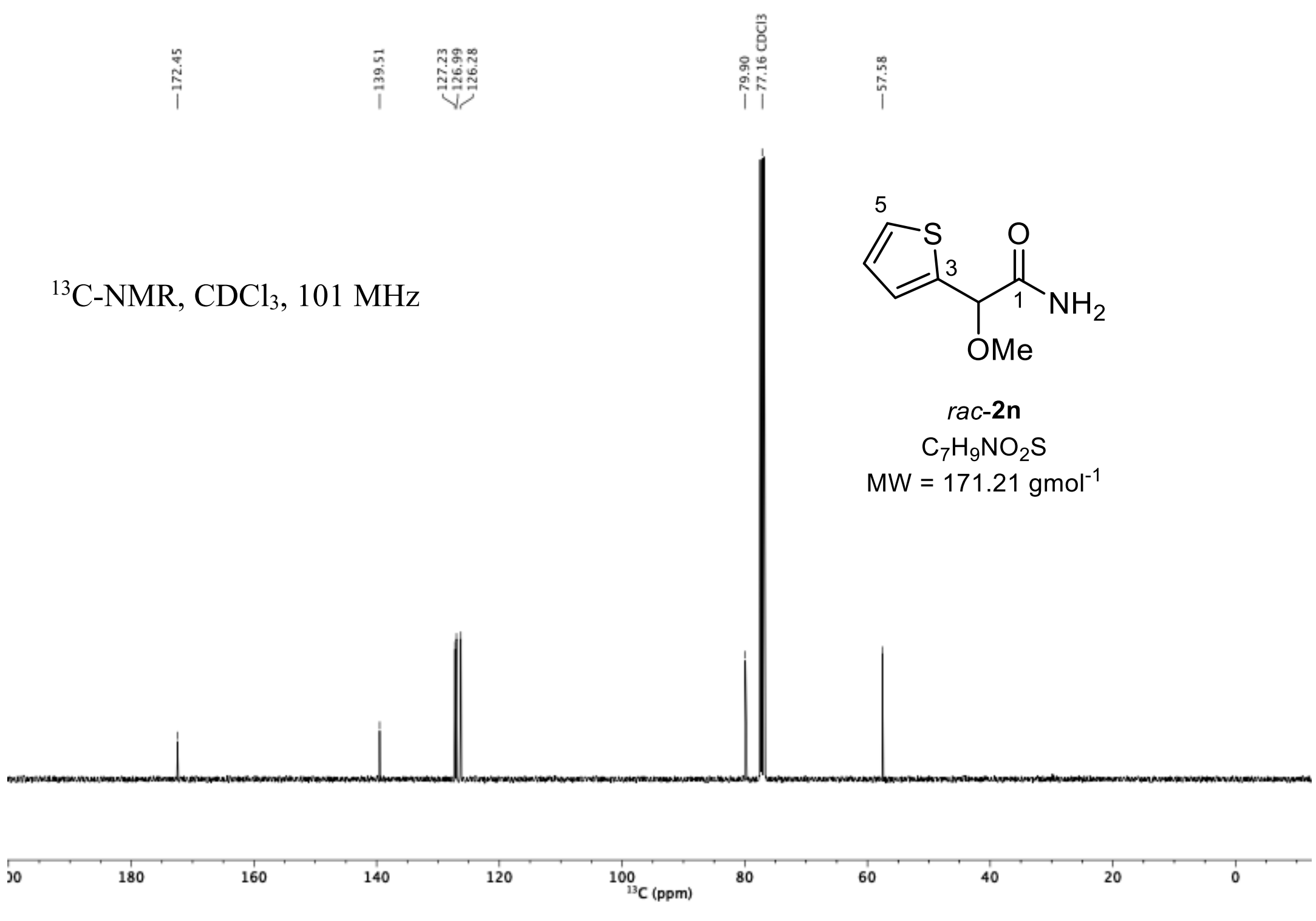
13C-NMR, CDCl3, 101 MHz
rac-2n
C7H9NO2S
MW = 171.21 gmol-1
NH2
OMe
172.45
139.51
127.23
126.99
126.28
79.90
77.16 CDCl3
57.58
13C (ppm)

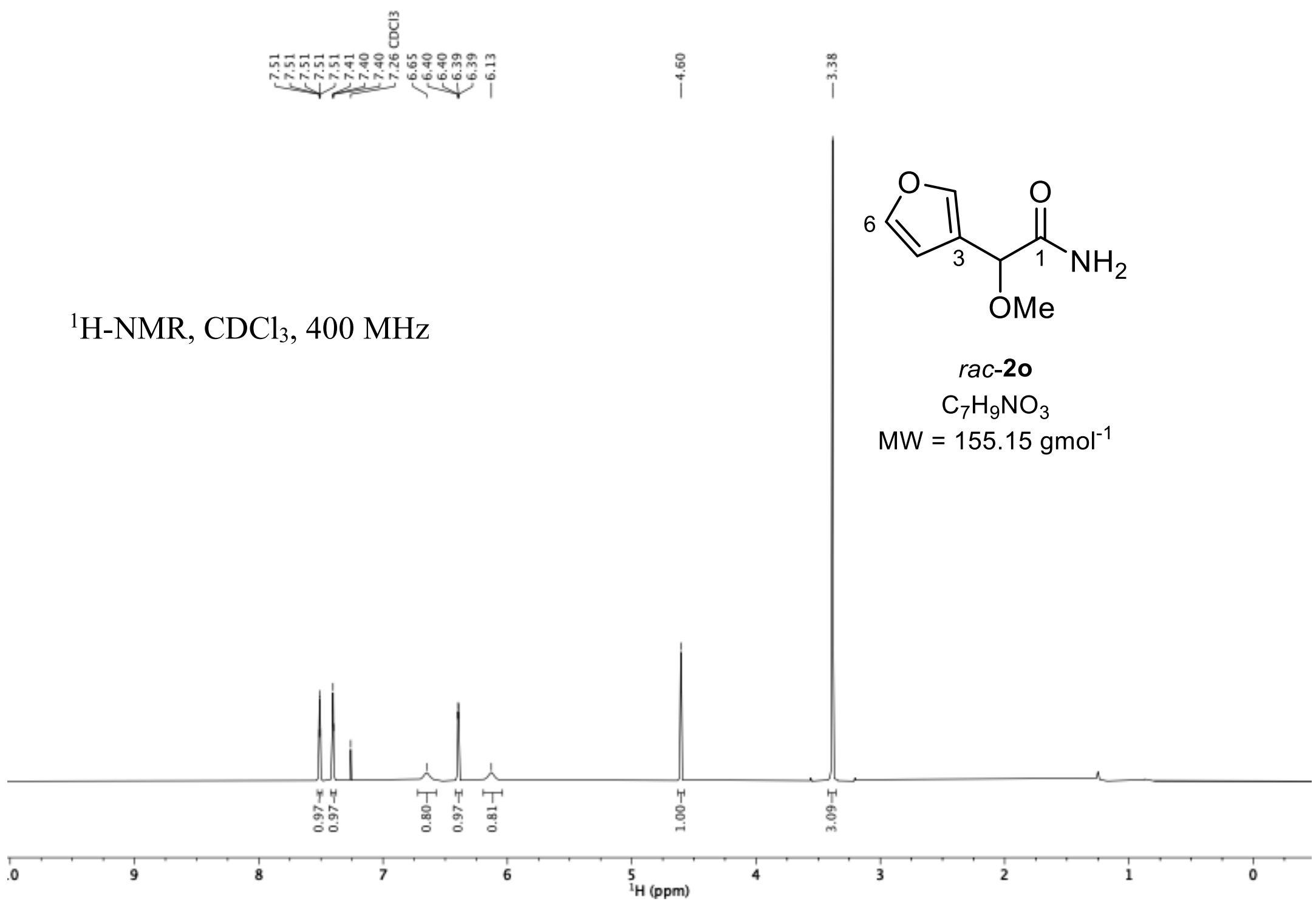

7.51
7.51
7.51
7.51
7.51
7.41
7.40
7.40
7.26 CDCl3
6.65
6.40
6.40
6.39
6.39
6.13
4.60
3.38
0.97
0.97
0.80
0.97
0.81
1.00
3.09
6
3
1
O
NH2
OMe
¹H-NMR, CDCl3, 400 MHz
rac-2o
C7H9NO3
MW = 155.15 gmol-1
¹H (ppm)

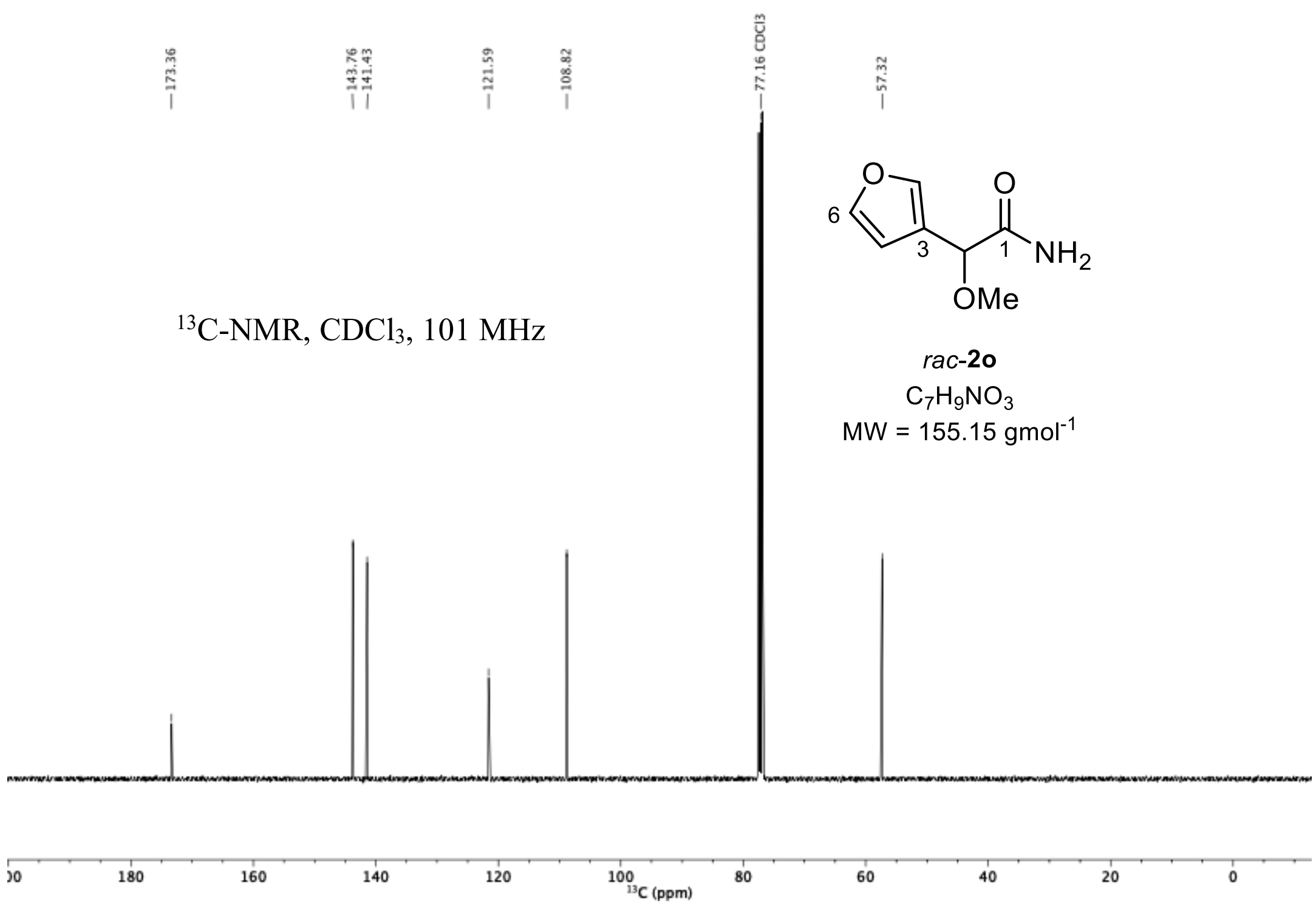

173.36
143.76
141.43
121.59
108.82
77.16 CDCl3
57.32
6
3
1
O
NH2
OMe
¹³C-NMR, CDCl3, 101 MHz
rac-2o
C7H9NO3
MW = 155.15 gmol-1
¹³C (ppm)

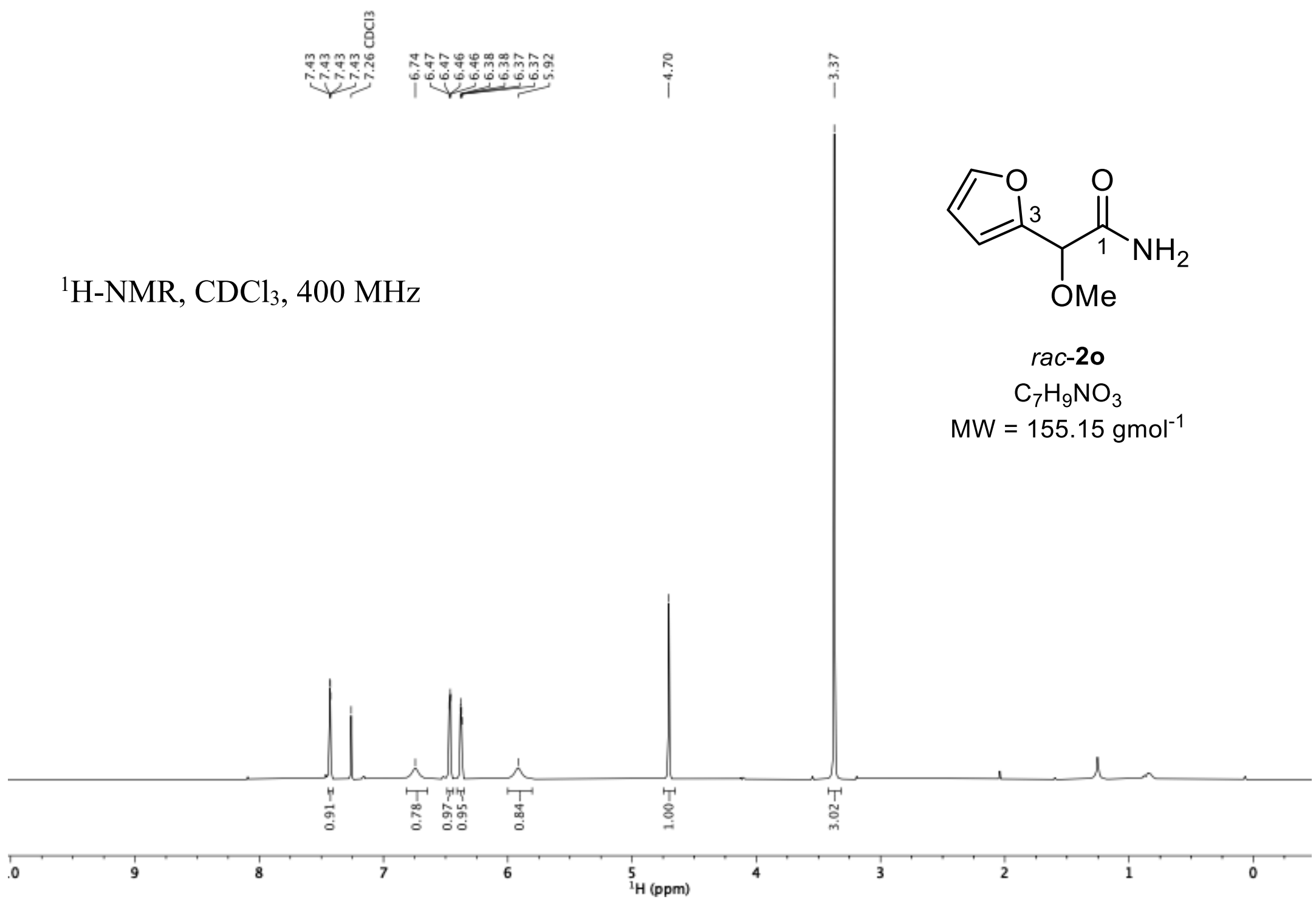

^{1}H-NMR, CDCl$_3$, 400 MHz

rac-**2o**
$C_7H_9NO_3$
MW = 155.15 gmol^{-1}

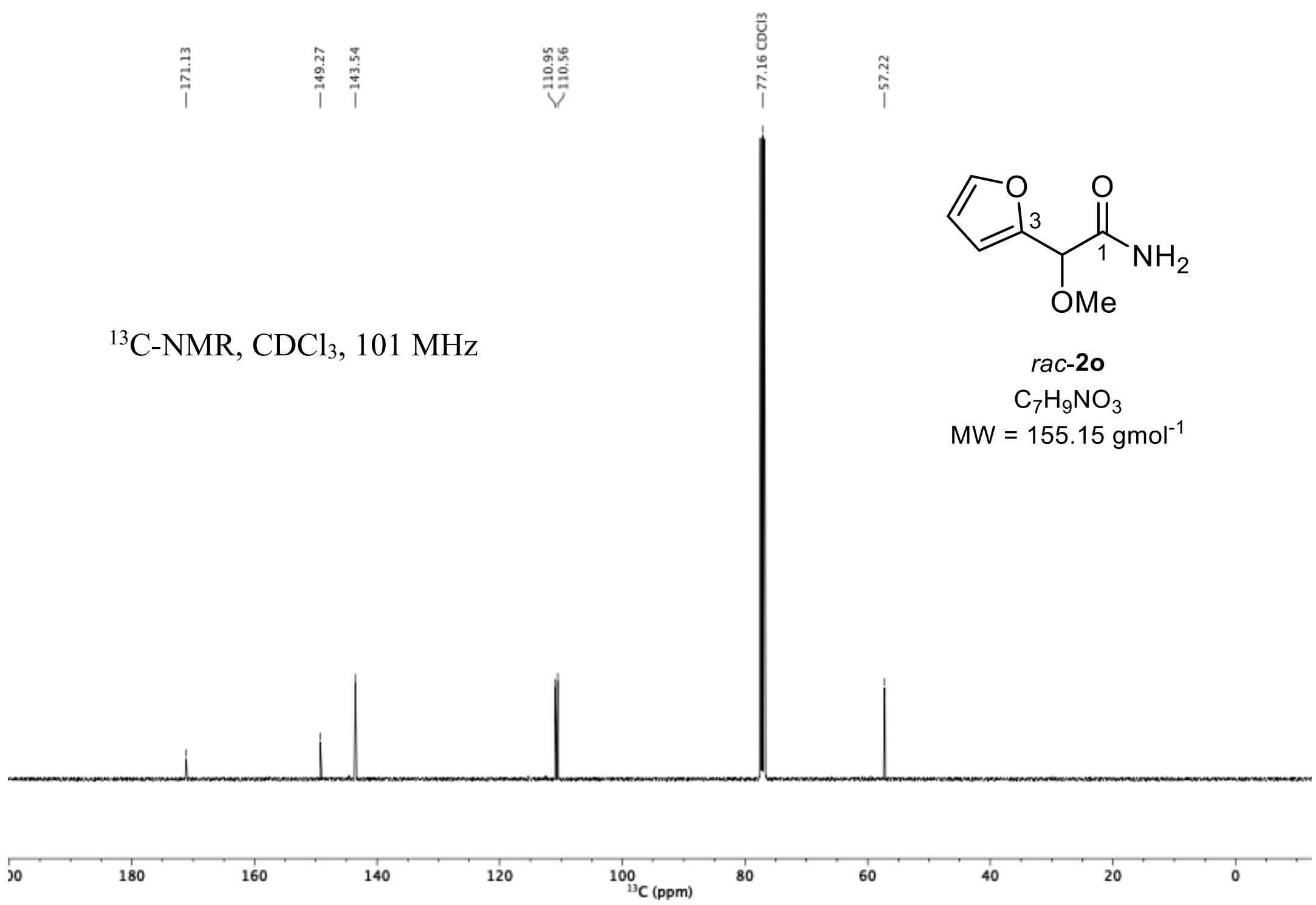

^{13}C-NMR, CDCl$_3$, 101 MHz

rac-**2o**
$C_7H_9NO_3$
MW = 155.15 gmol^{-1}

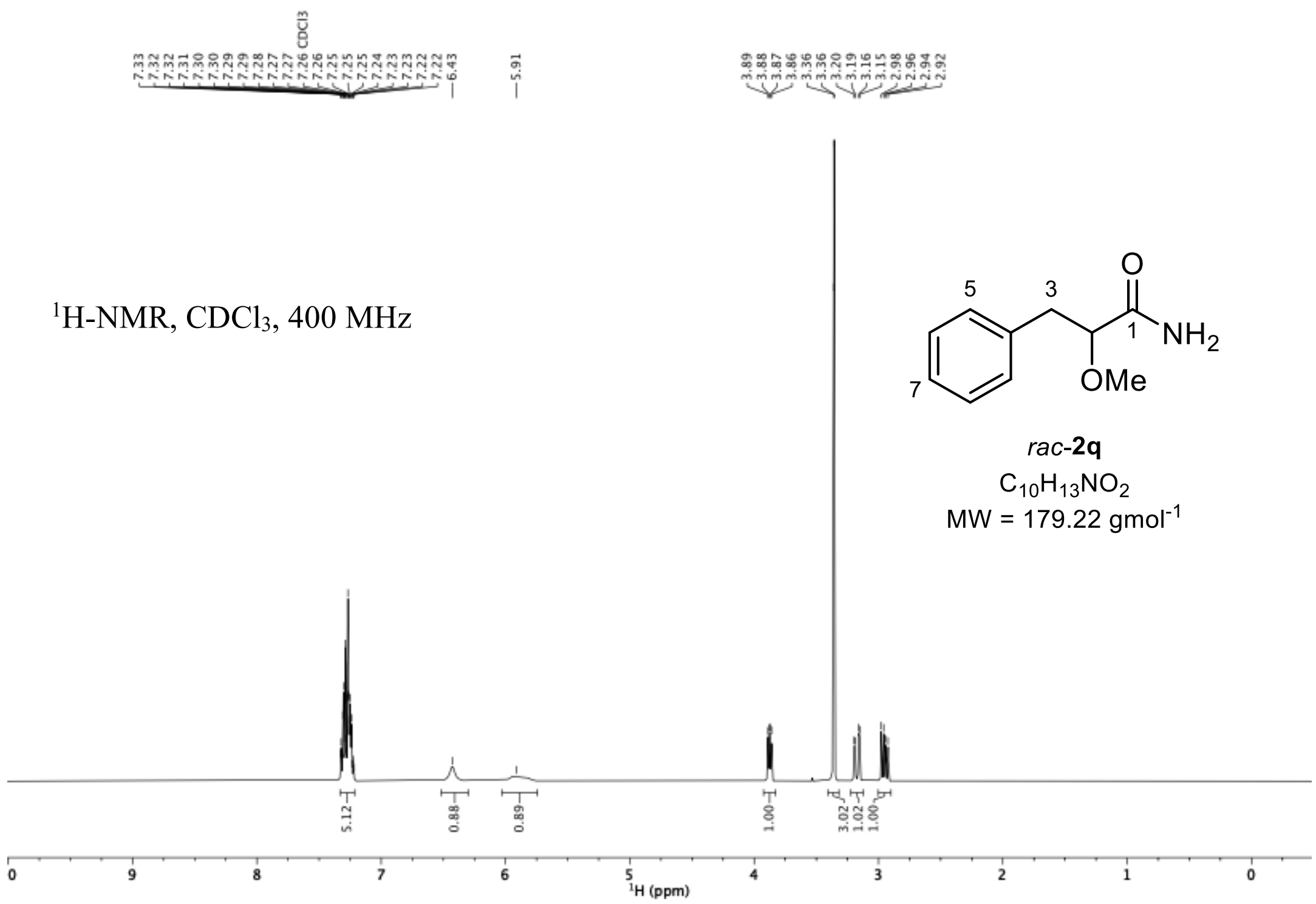

1H-NMR, CDCl3, 400 MHz
5
3
O
1
NH2
7
OMe
rac-2q
C10H13NO2
MW = 179.22 gmol-1
5.12
0.88
0.89
1.00
3.02
1.02
1.00
1H (ppm)

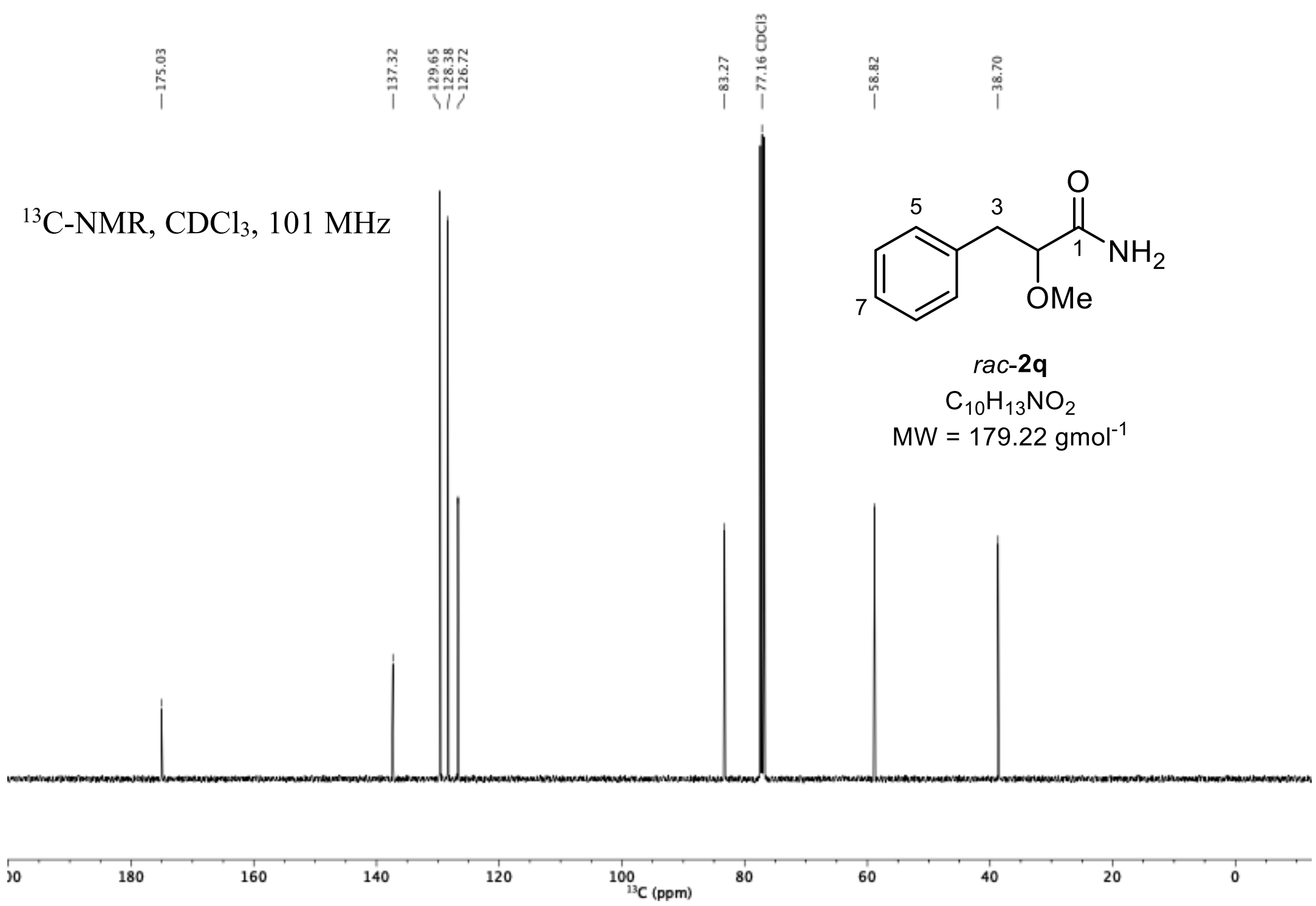

13C-NMR, CDCl3, 101 MHz
175.03
137.32
129.65
128.38
126.72
83.27
77.16 CDCl3
58.82
38.70
5
3
O
1
NH2
7
OMe
rac-2q
C10H13NO2
MW = 179.22 gmol-1
13C (ppm)

^{1}H-NMR, $CDCl_3$, 400 MHz

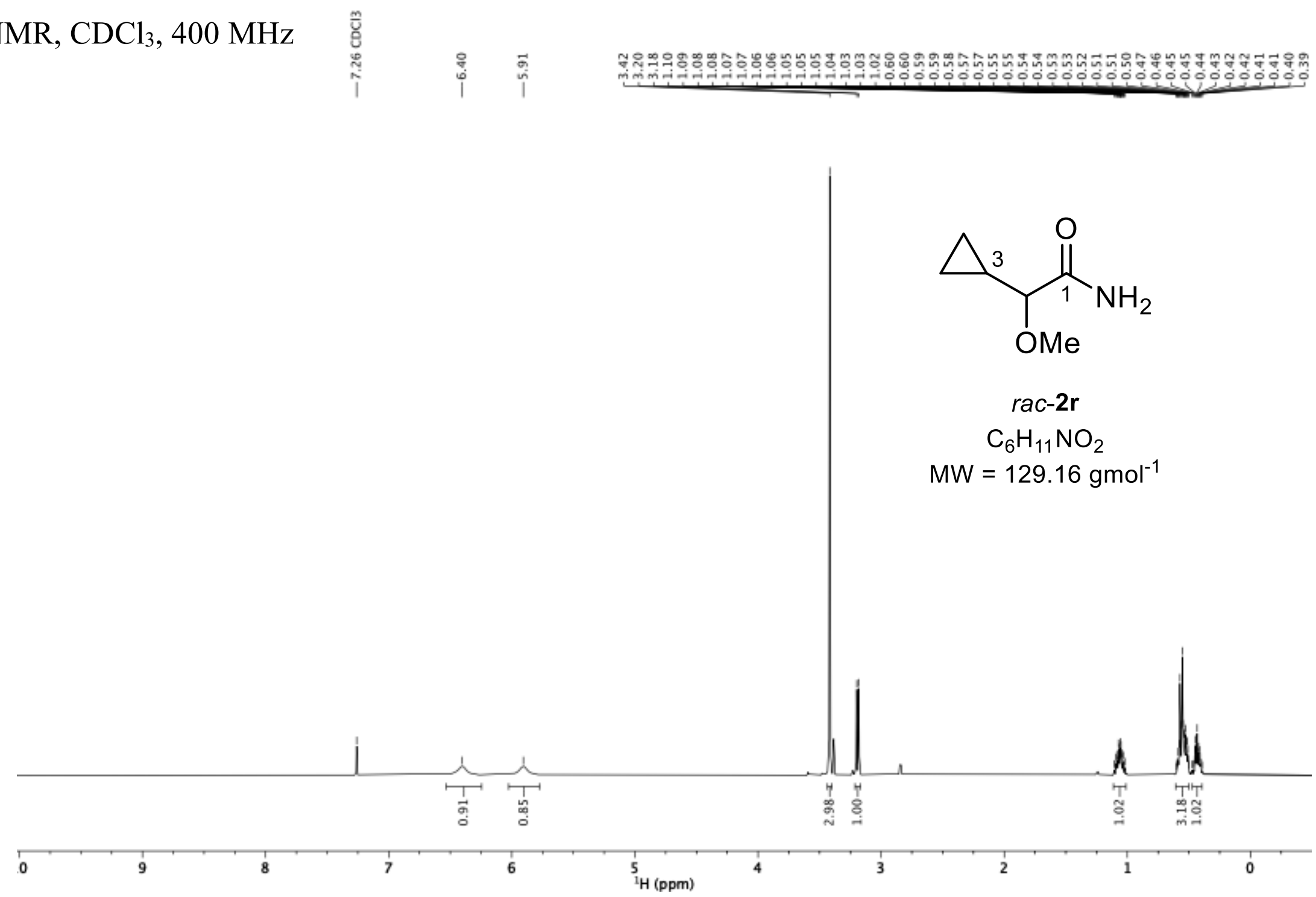

^{13}C-NMR, $CDCl_3$, 101 MHz

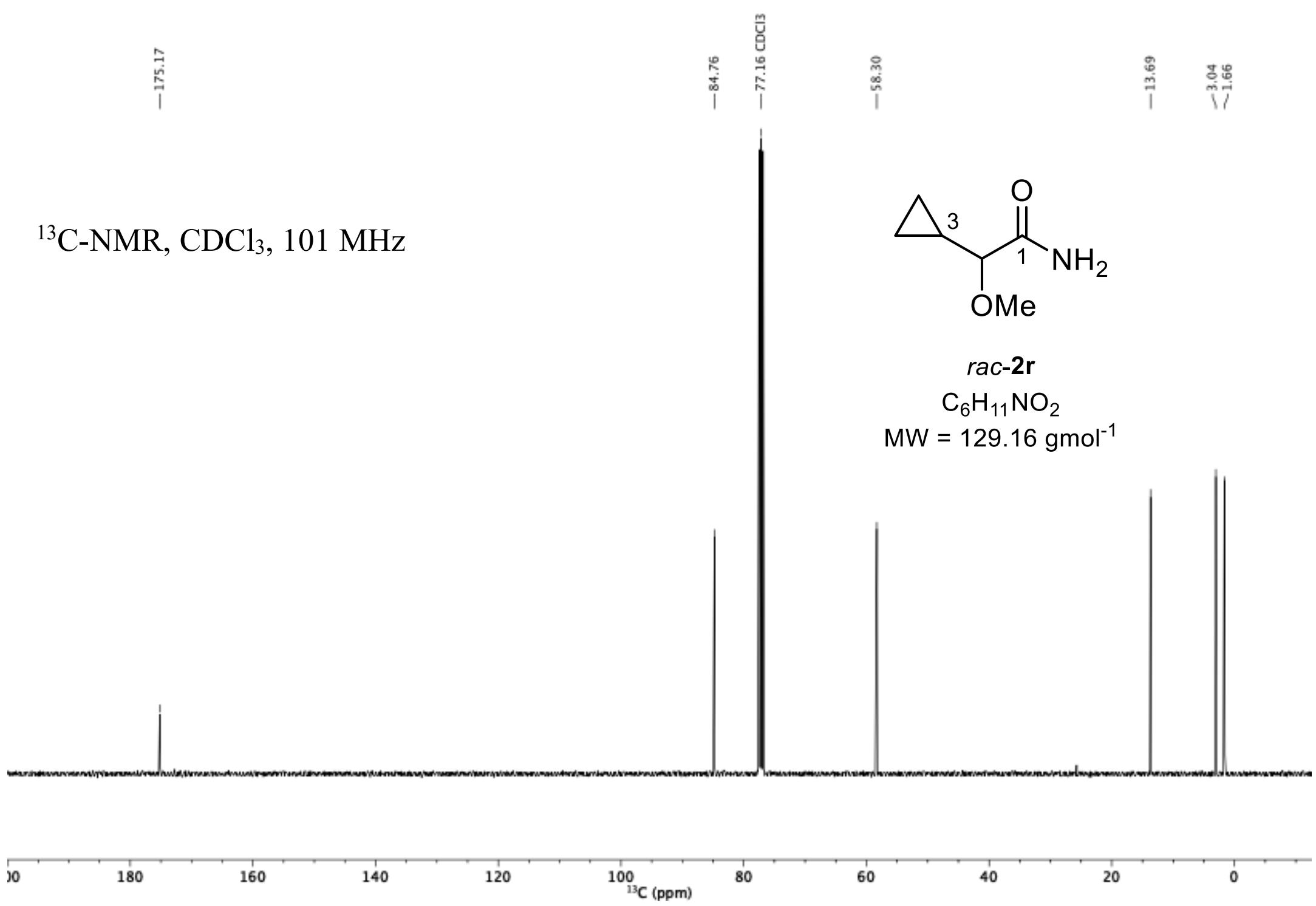

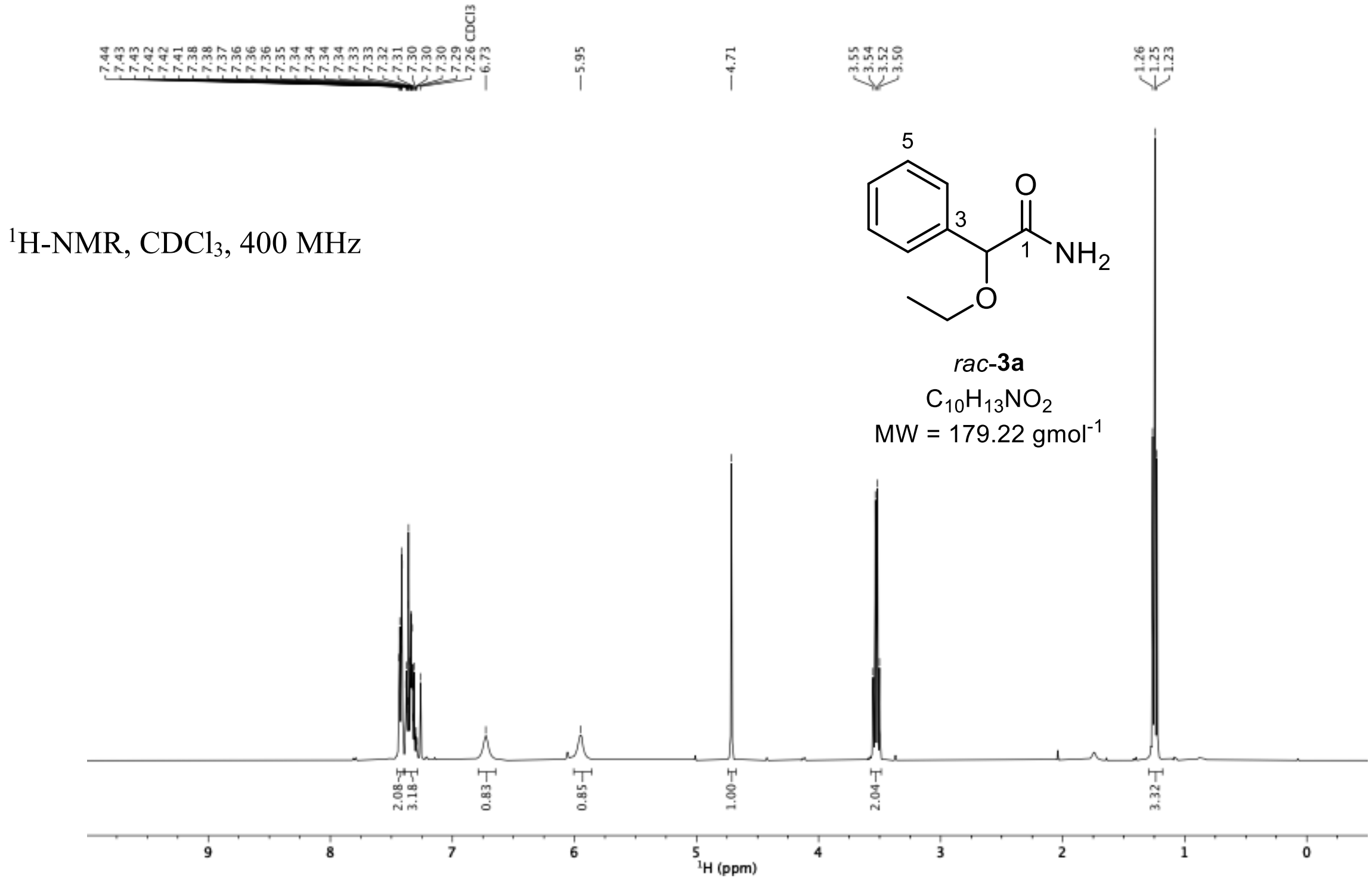
7.44
7.43
7.43
7.42
7.42
7.41
7.38
7.38
7.37
7.36
7.36
7.36
7.35
7.34
7.34
7.34
7.34
7.34
7.33
7.33
7.32
7.32
7.31
7.30
7.30
7.30
7.29
7.26 CDCl3
6.73
5.95
4.71
3.55
3.54
3.52
3.50
1.26
1.25
1.23
1H-NMR, CDCl3, 400 MHz
5
3
1
O
NH2
O
rac-3a
C10H13NO2
MW = 179.22 gmol-1
2.08
3.18
0.83
0.85
1.00
2.04
3.32
9
8
7
6
5
4
3
2
1
0
1H (ppm)

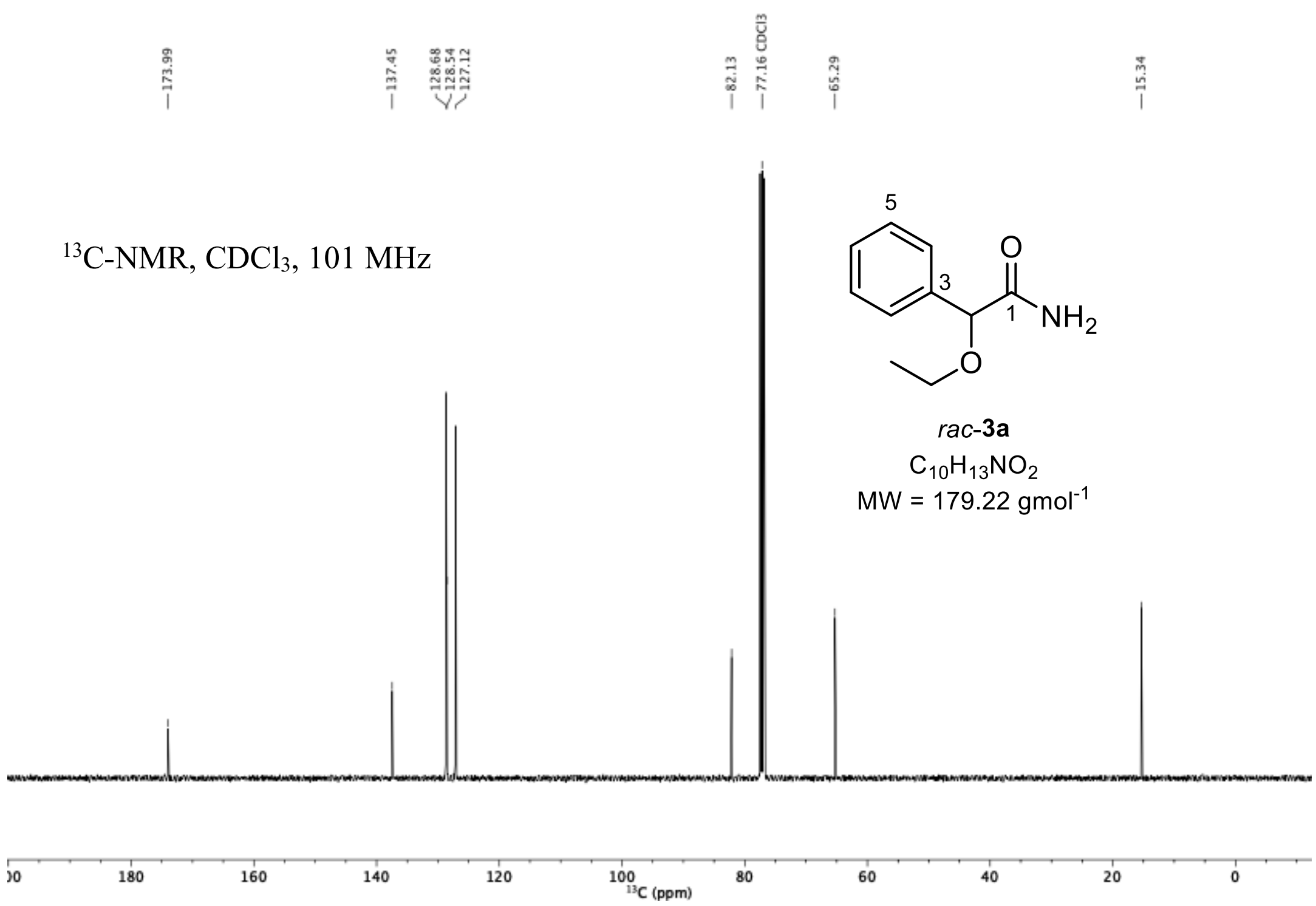
173.99
137.45
128.68
128.54
127.12
82.13
77.16 CDCl3
65.29
15.34
13C-NMR, CDCl3, 101 MHz
5
3
1
O
NH2
O
rac-3a
C10H13NO2
MW = 179.22 gmol-1
180
160
140
120
100
80
60
40
20
0
13C (ppm)

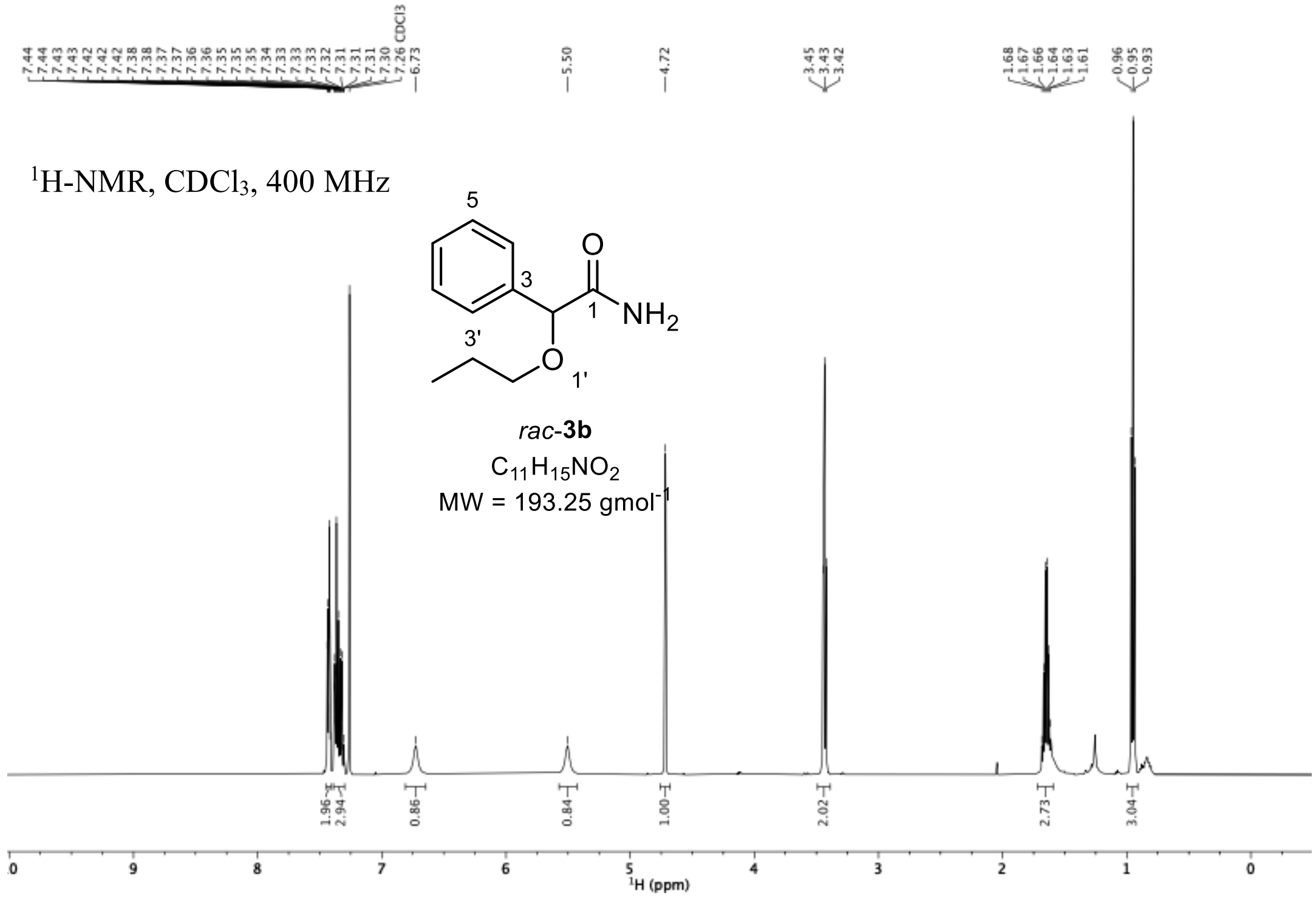
1H-NMR, CDCl3, 400 MHz
rac-3b
C11H15NO2
MW = 193.25 gmol-1
1H (ppm)

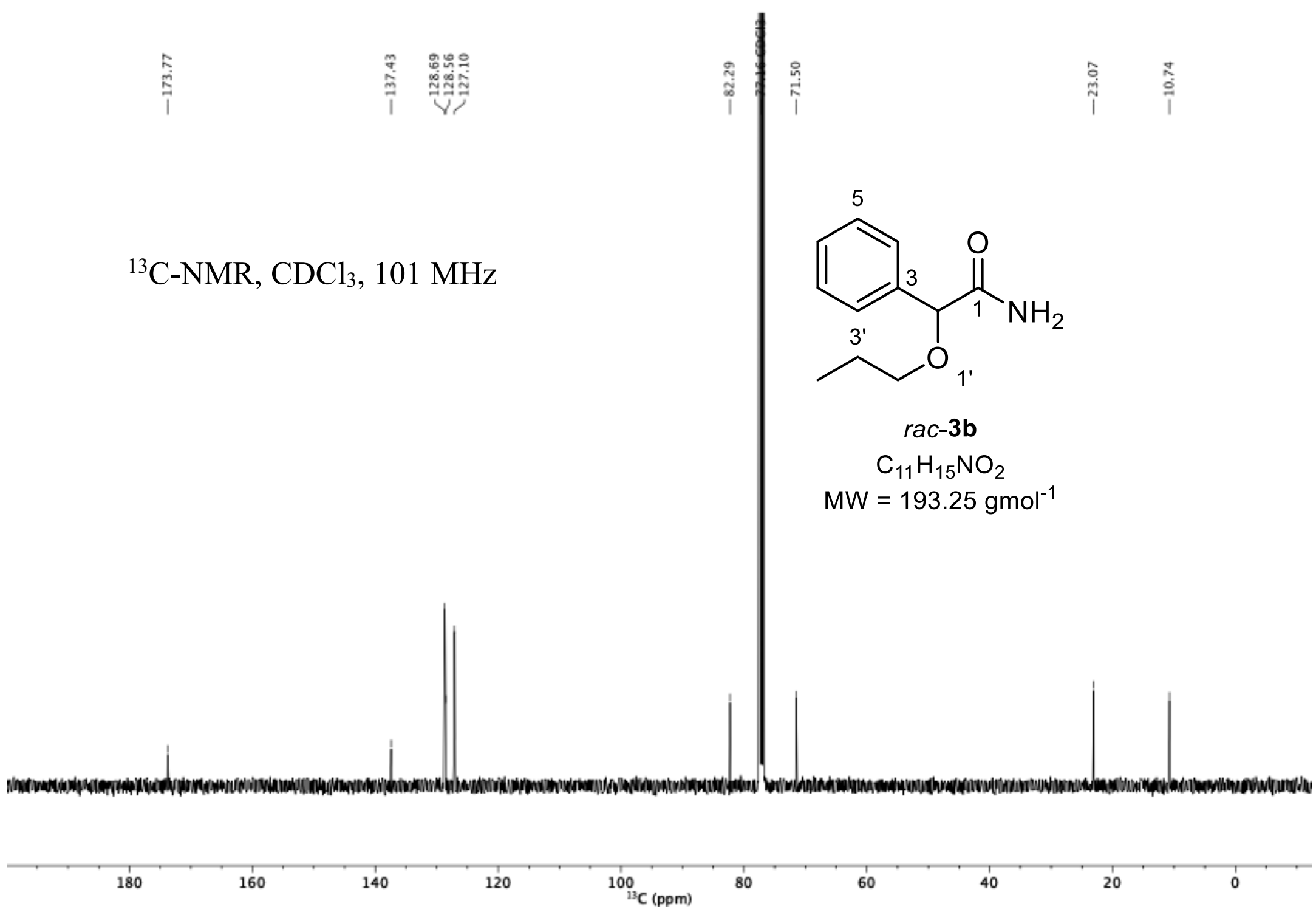
13C-NMR, CDCl3, 101 MHz
rac-3b
C11H15NO2
MW = 193.25 gmol-1
13C (ppm)

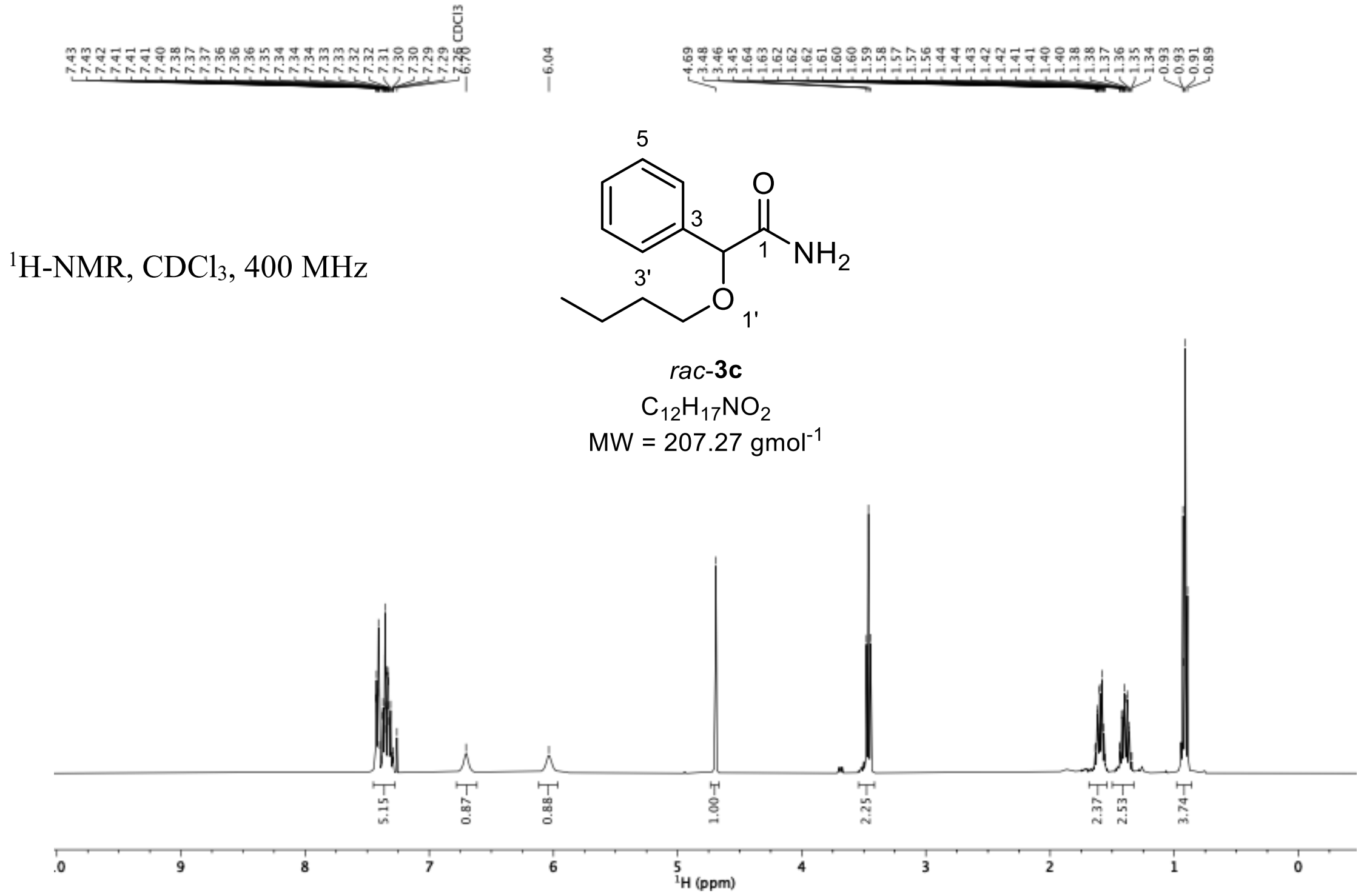

^{1}H-NMR, CDCl$_3$, 400 MHz
5
3
3'
1
1'
O
NH$_2$
rac-**3c**
$C_{12}H_{17}NO_2$
MW = 207.27 gmol^{-1}
7.43 7.43 7.42 7.41 7.41 7.40 7.38 7.37 7.37 7.36 7.36 7.35 7.34 7.34 7.34 7.33 7.33 7.32 7.31 7.30 7.30 7.29 7.29 7.26 CDCl3 6.70
6.04
4.69 3.48 3.46 3.45 1.64 1.63 1.62 1.62 1.61 1.60 1.60 1.59 1.58 1.57 1.56 1.44 1.44 1.43 1.42 1.41 1.41 1.40 1.38 1.38 1.37 1.36 1.35 1.34 0.93 0.93 0.91 0.89
5.15 0.87 0.88 1.00 2.25 2.37 2.53 3.74
^{1}H (ppm)

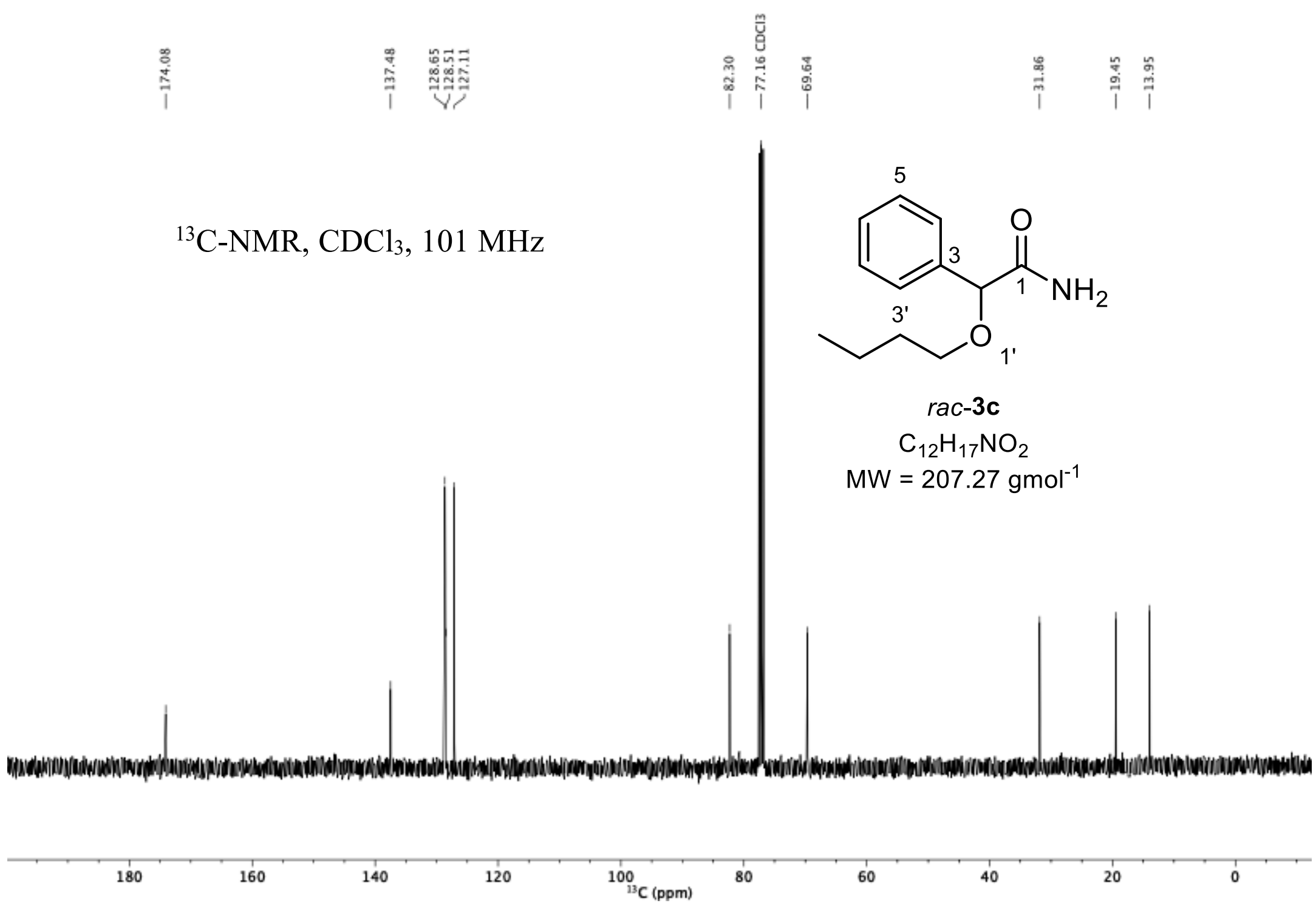

^{13}C-NMR, CDCl$_3$, 101 MHz
5
3
3'
1
1'
O
NH$_2$
rac-**3c**
$C_{12}H_{17}NO_2$
MW = 207.27 gmol^{-1}
174.08
137.48
128.65 128.51 127.11
82.30
77.16 CDCl3
69.64
31.86
19.45
13.95
^{13}C (ppm)

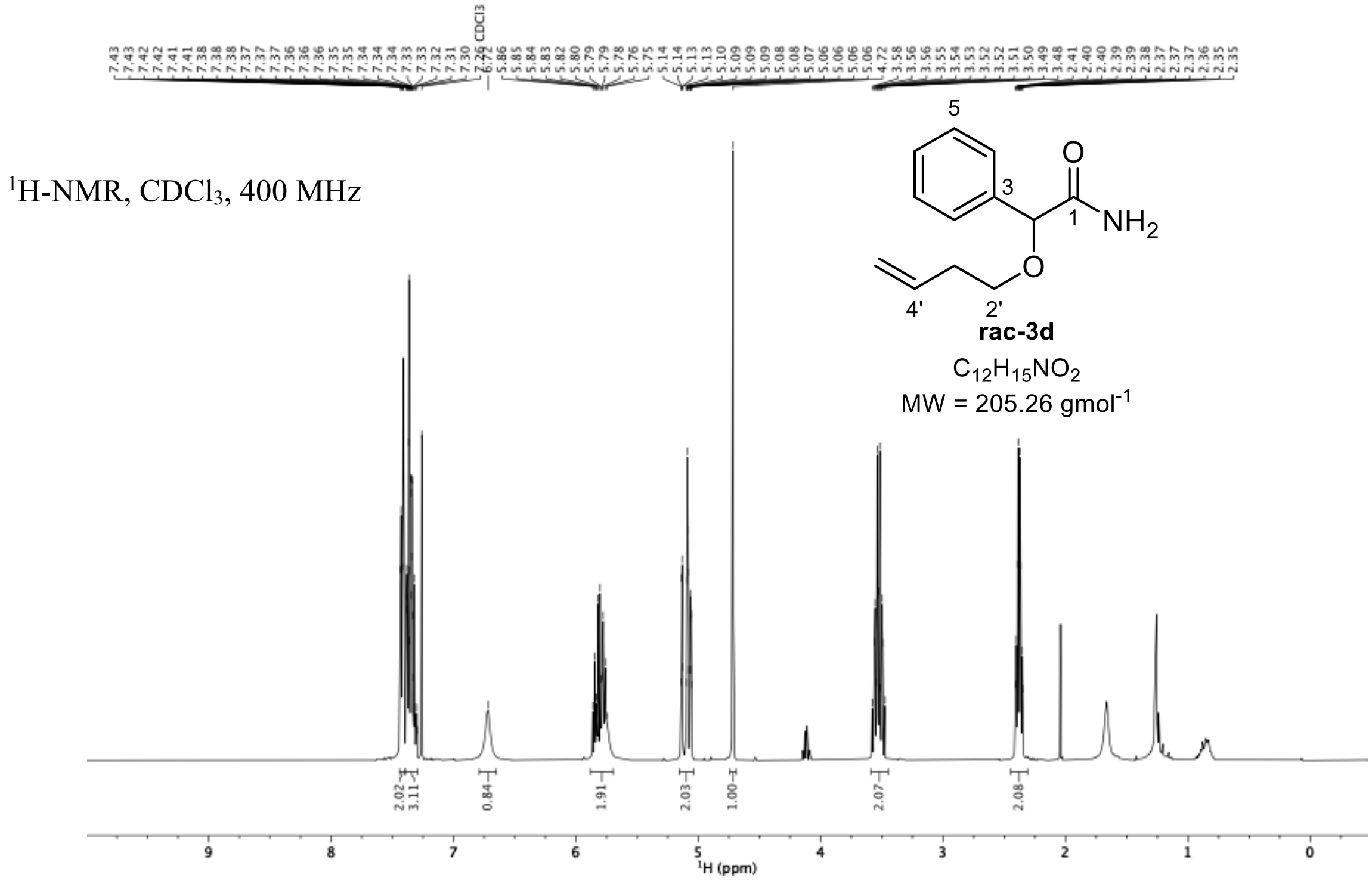
^{1}H-NMR, CDCl$_3$, 400 MHz
rac-3d
$C_{12}H_{15}NO_2$
MW = 205.26 gmol^{-1}
^{1}H (ppm)

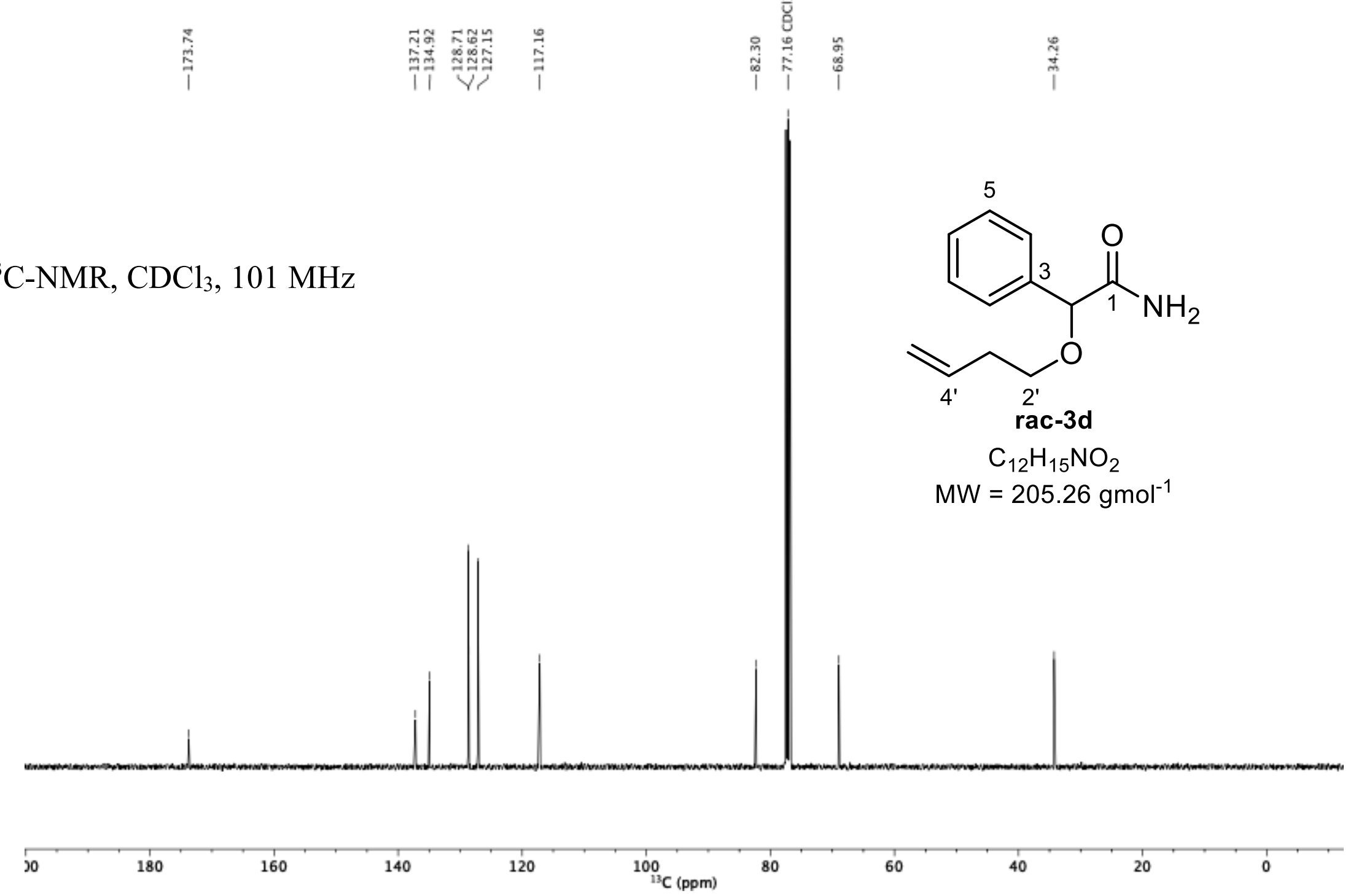
^{13}C-NMR, CDCl$_3$, 101 MHz
173.74
137.21
134.92
128.71
128.62
127.15
117.16
82.30
77.16 CDCl3
68.95
34.26
rac-3d
$C_{12}H_{15}NO_2$
MW = 205.26 gmol^{-1}
^{13}C (ppm)

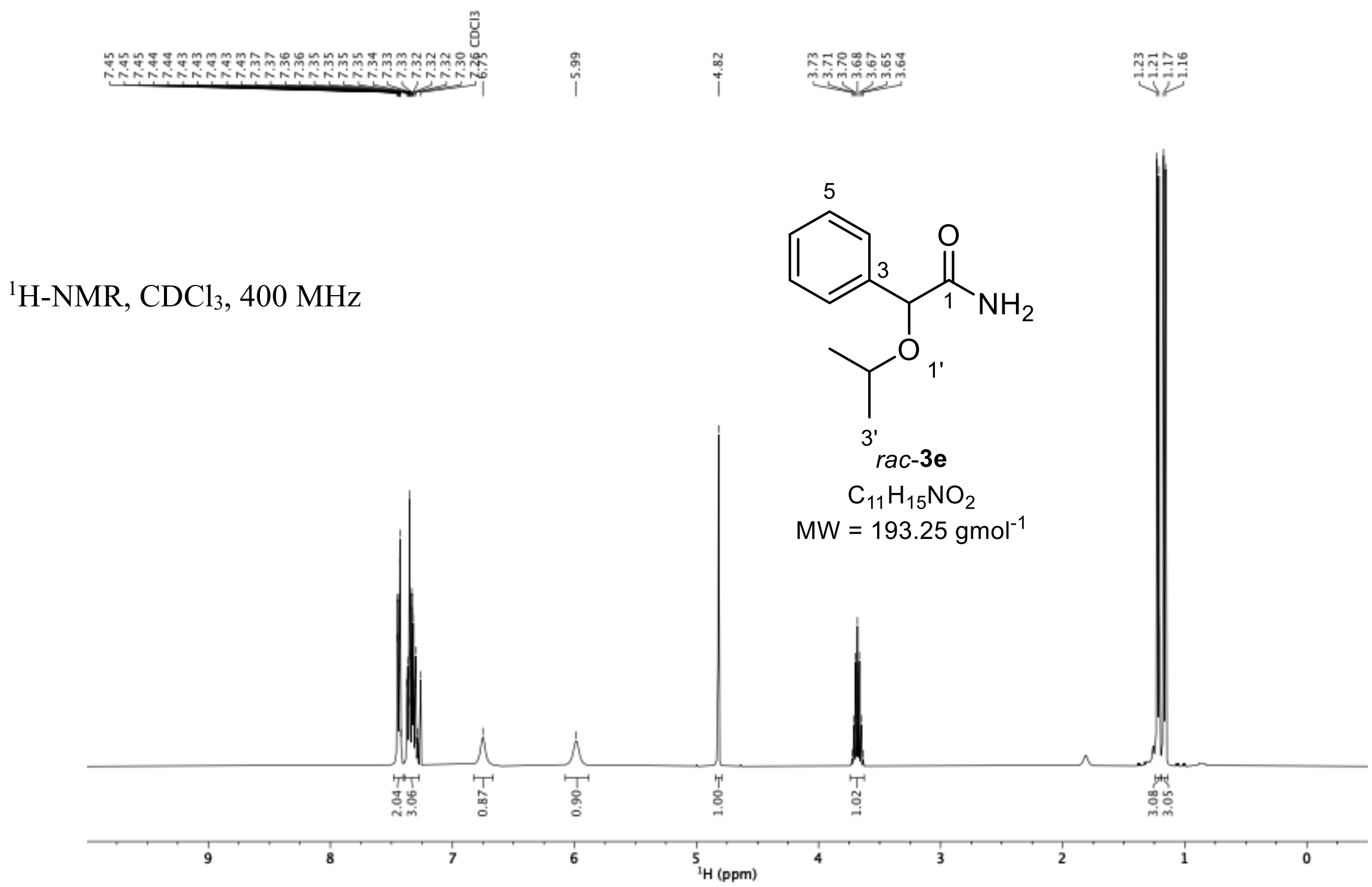
^{1}H-NMR, $CDCl_3$, 400 MHz
rac-3e
$C_{11}H_{15}NO_2$
MW = 193.25 gmol^{-1}
^{1}H (ppm)

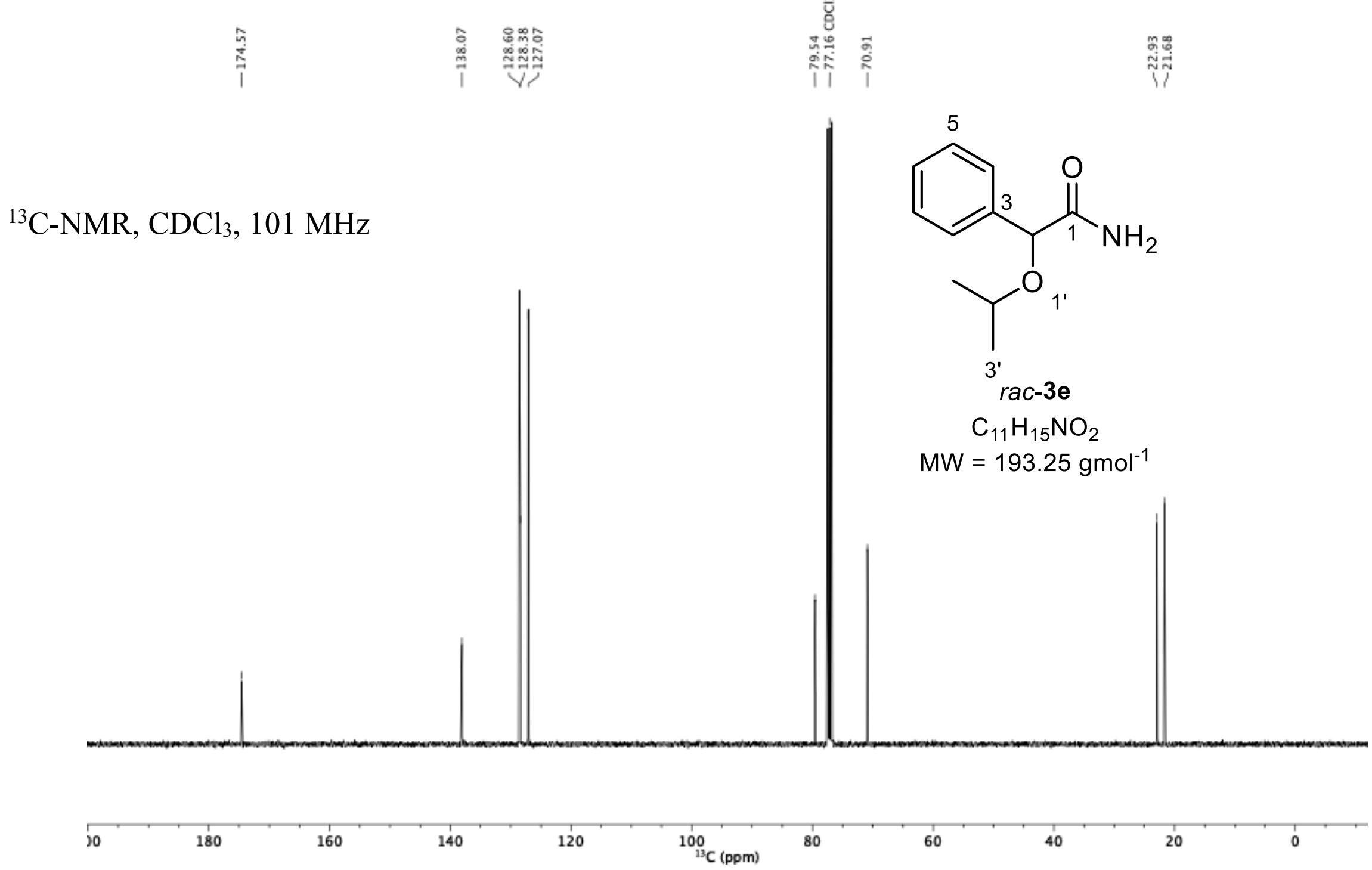
^{13}C-NMR, $CDCl_3$, 101 MHz
rac-3e
$C_{11}H_{15}NO_2$
MW = 193.25 gmol^{-1}
^{13}C (ppm)

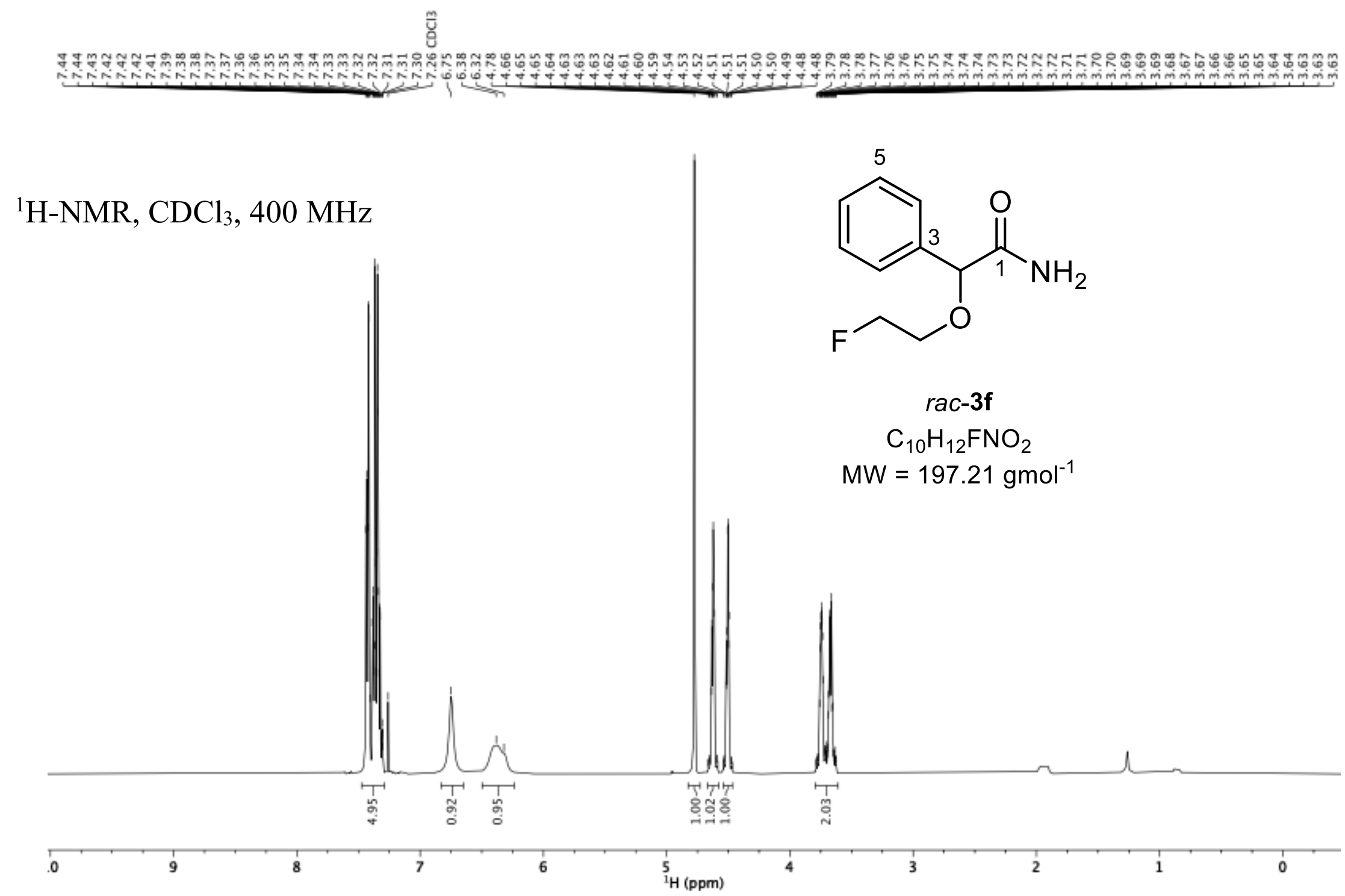

1H-NMR, CDCl3, 400 MHz
rac-3f
C10H12FNO2
MW = 197.21 gmol-1
1H (ppm)

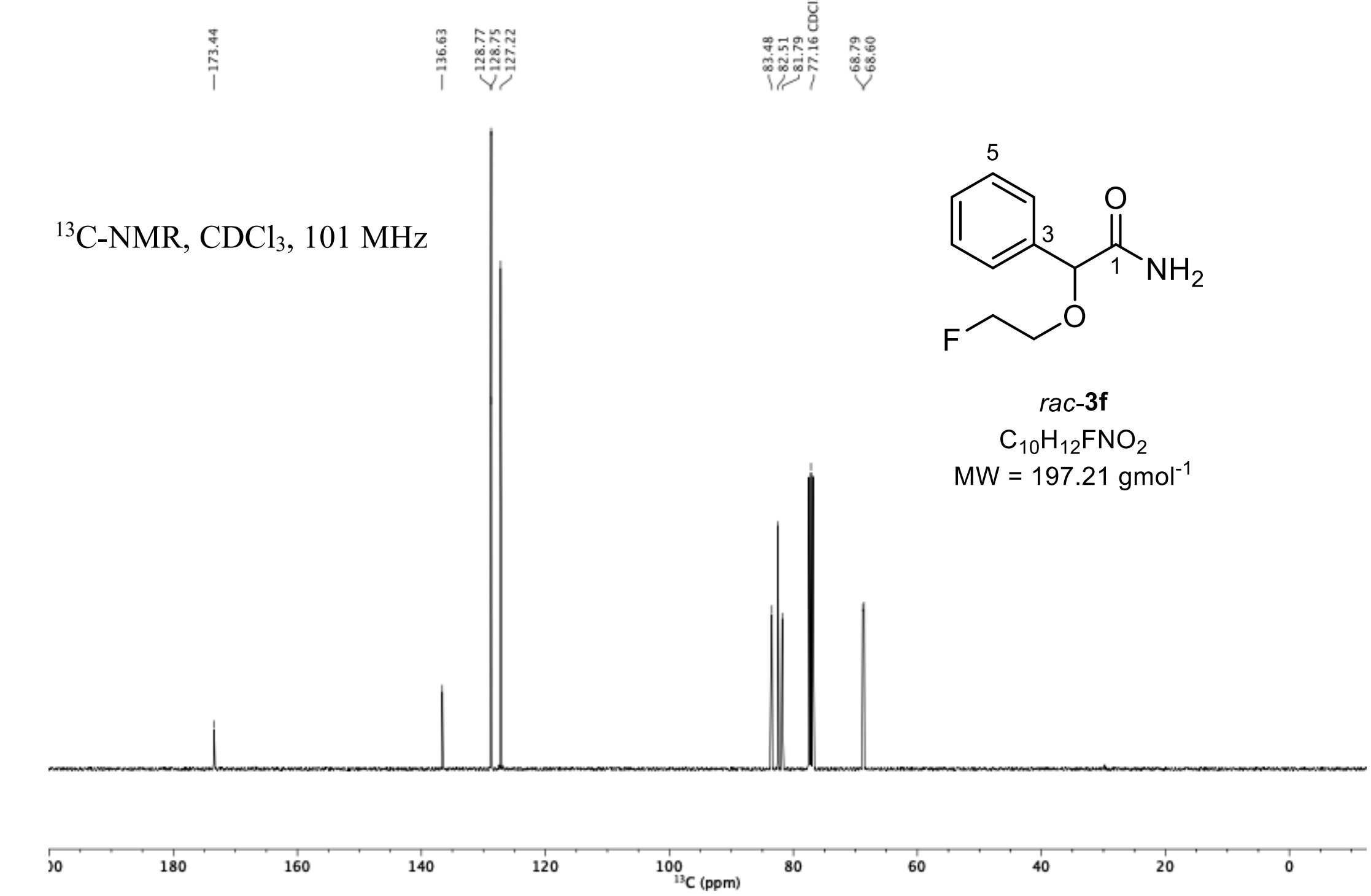

13C-NMR, CDCl3, 101 MHz
rac-3f
C10H12FNO2
MW = 197.21 gmol-1
13C (ppm)

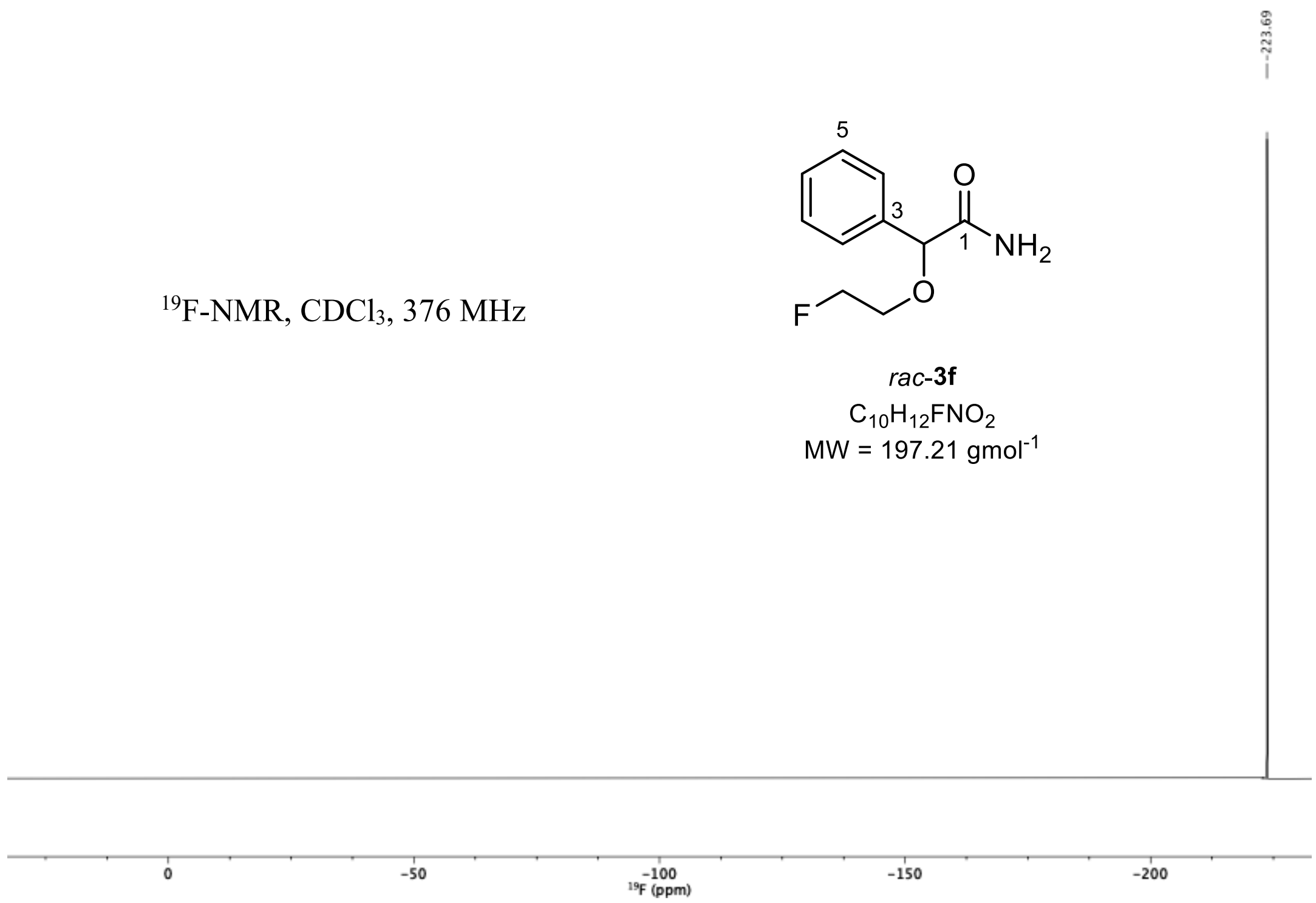
^{19}F-NMR, $CDCl_3$, 376 MHz
5
3
O
1
NH_2
F
O
rac-**3f**
$C_{10}H_{12}FNO_2$
MW = 197.21 gmol^{-1}
−223.69
0
−50
−100
−150
−200
^{19}F (ppm)

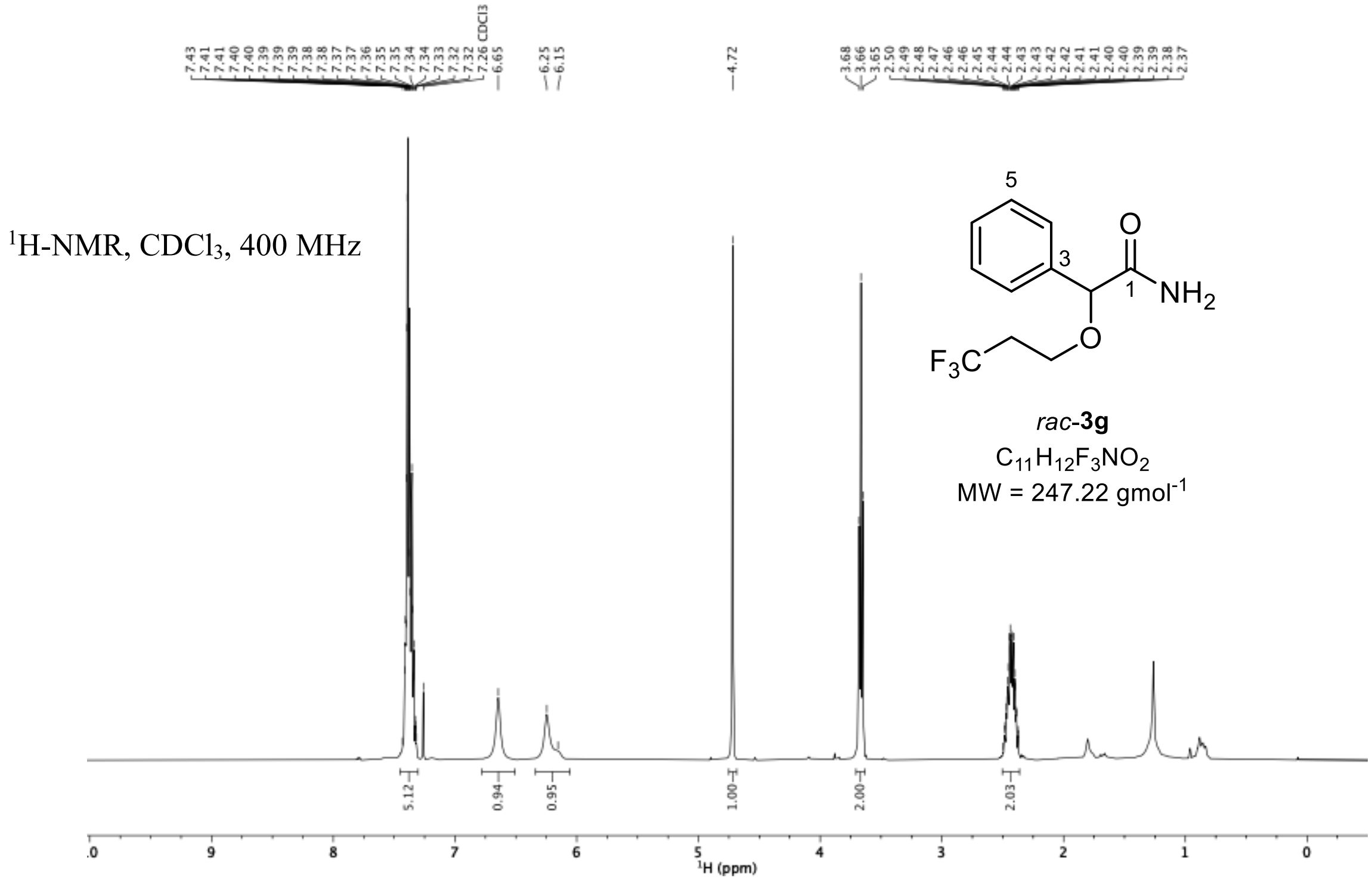

1H-NMR, CDCl3, 400 MHz
rac-3g
C11H12F3NO2
MW = 247.22 gmol-1
1H (ppm)

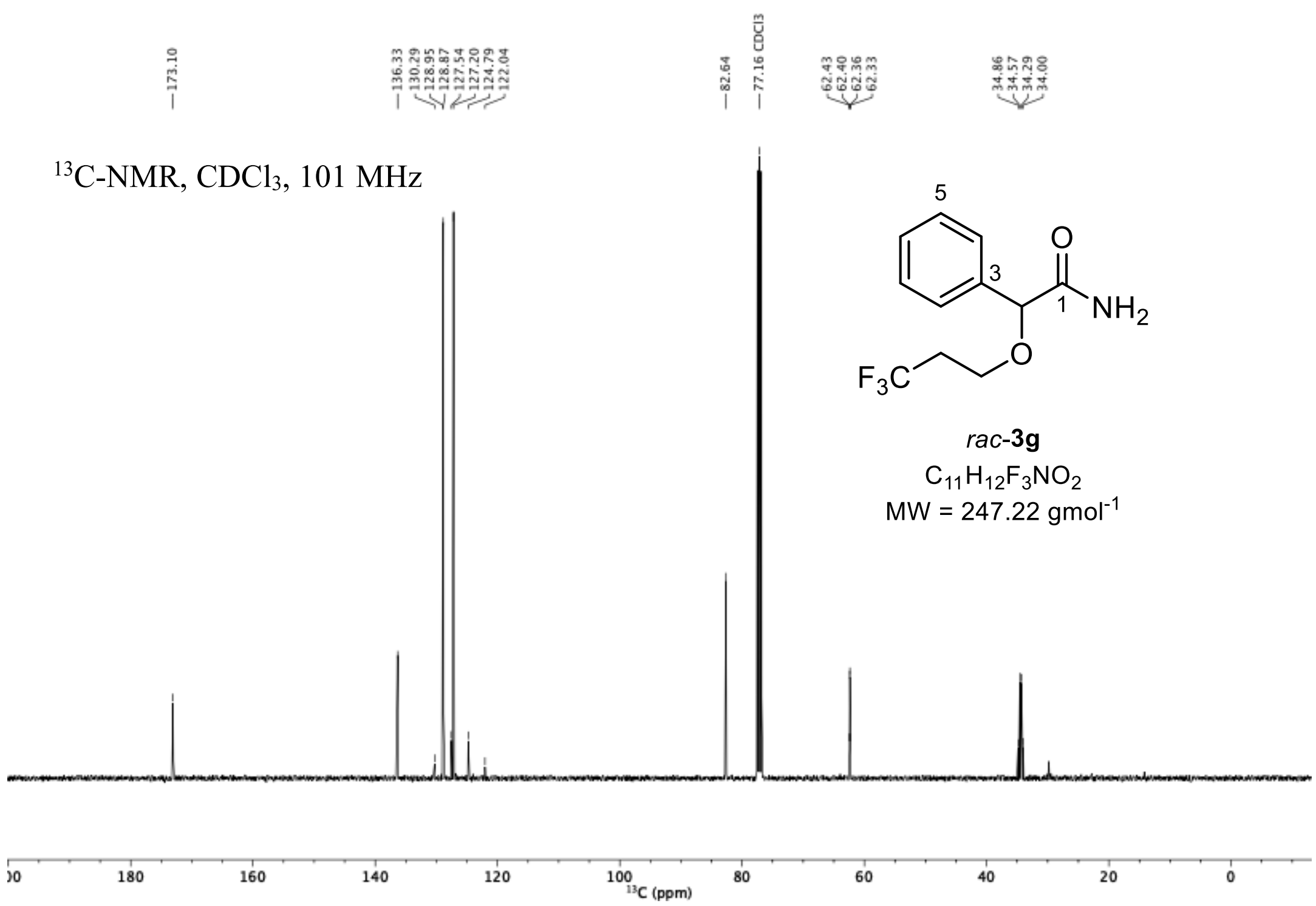

13C-NMR, CDCl3, 101 MHz
rac-3g
C11H12F3NO2
MW = 247.22 gmol-1
13C (ppm)

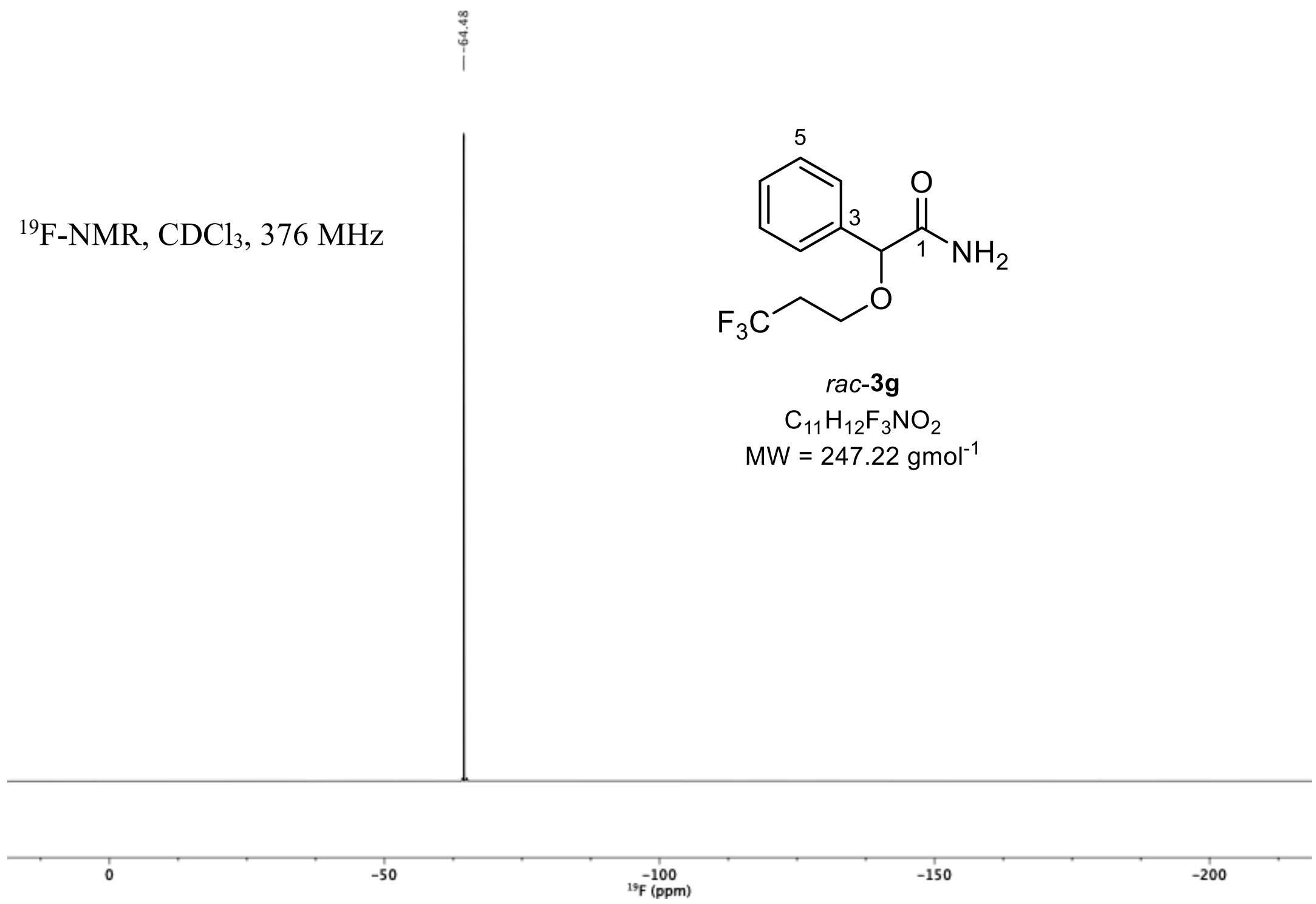

rac-**3g**

$C_{11}H_{12}F_3NO_2$

MW = 247.22 $gmol^{-1}$

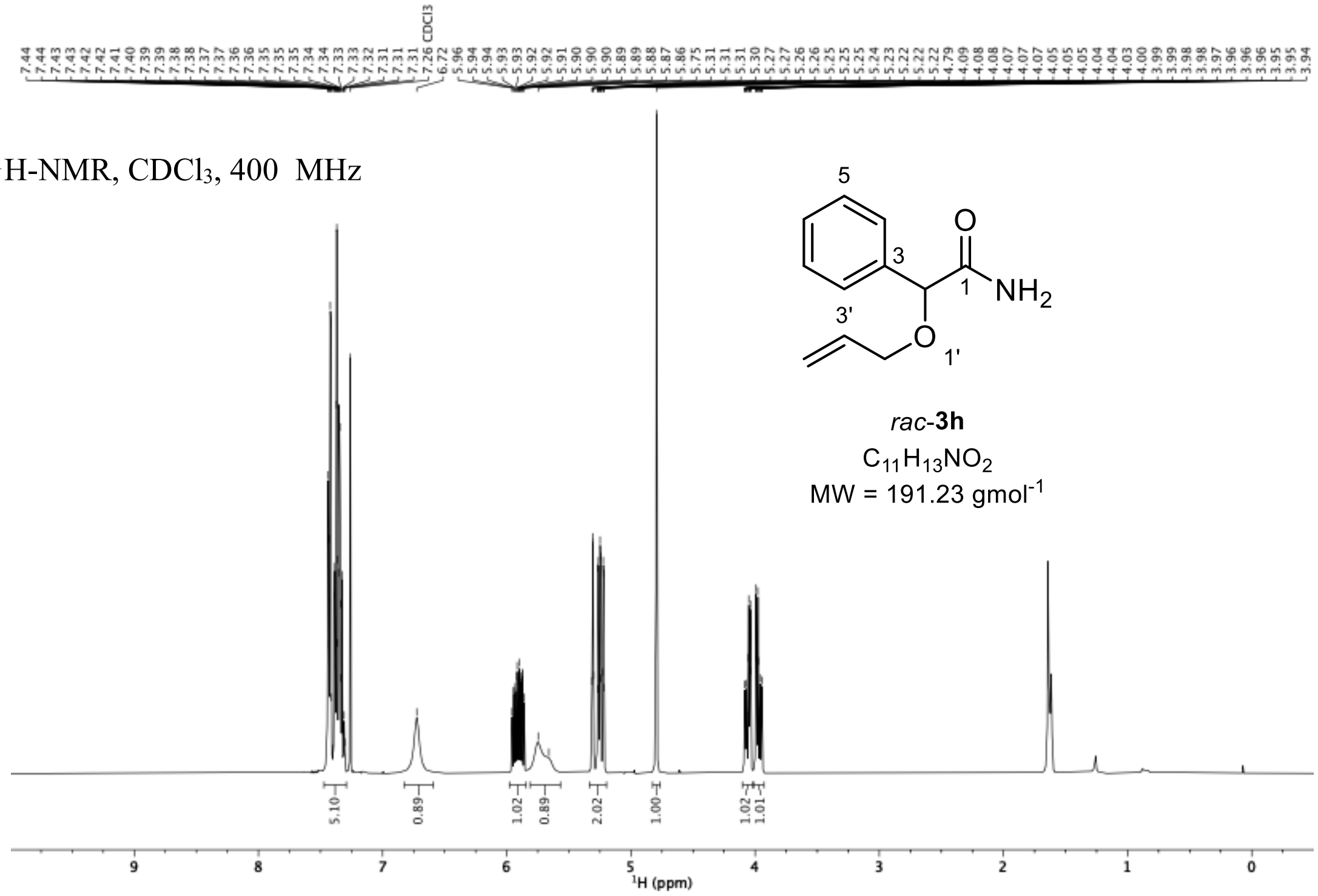
1H-NMR, CDCl3, 400 MHz
rac-3h
C11H13NO2
MW = 191.23 gmol-1
1H (ppm)

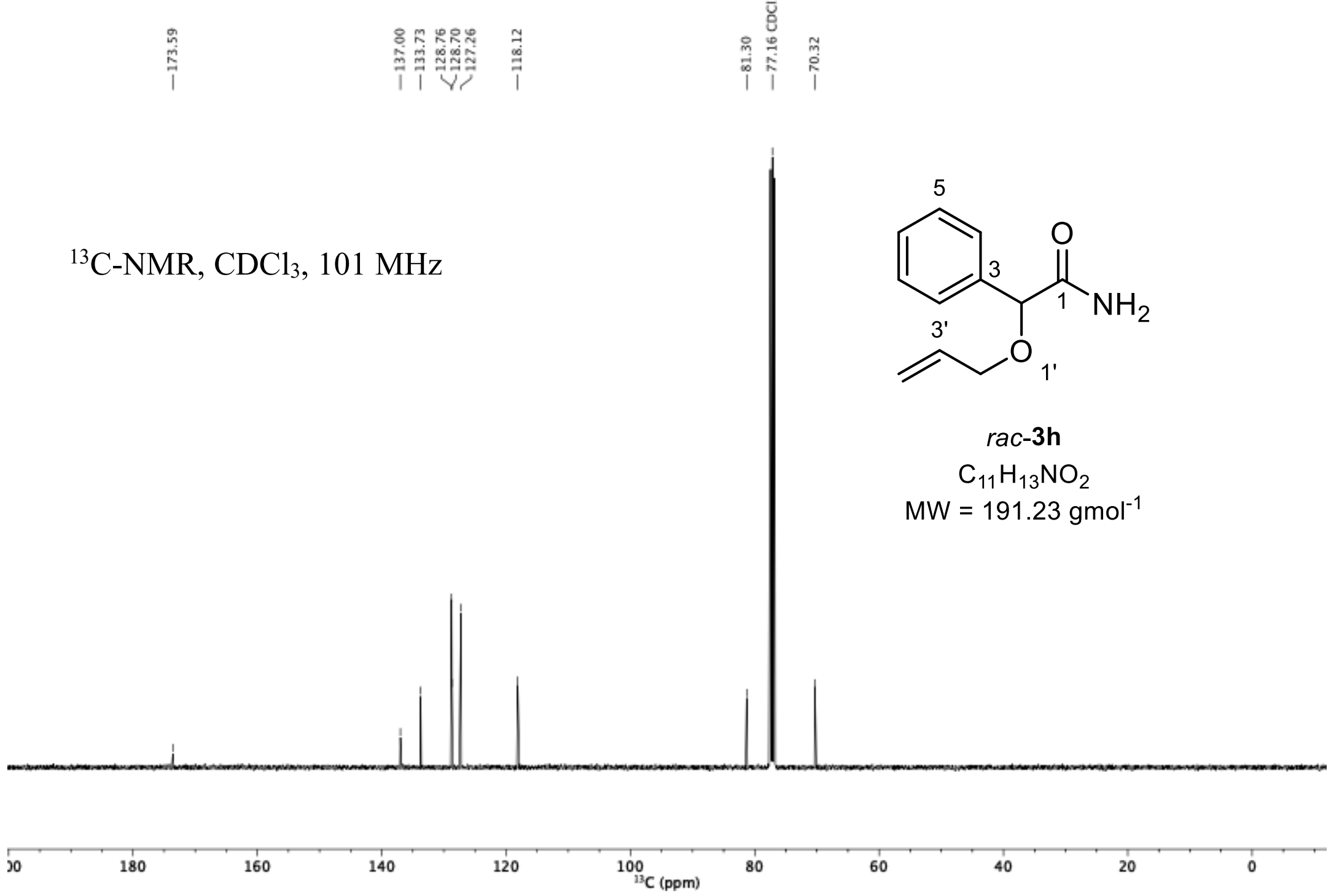
13C-NMR, CDCl3, 101 MHz
173.59
137.00
133.73
128.76
128.70
127.26
118.12
81.30
77.16 CDCl3
70.32
rac-3h
C11H13NO2
MW = 191.23 gmol-1
13C (ppm)

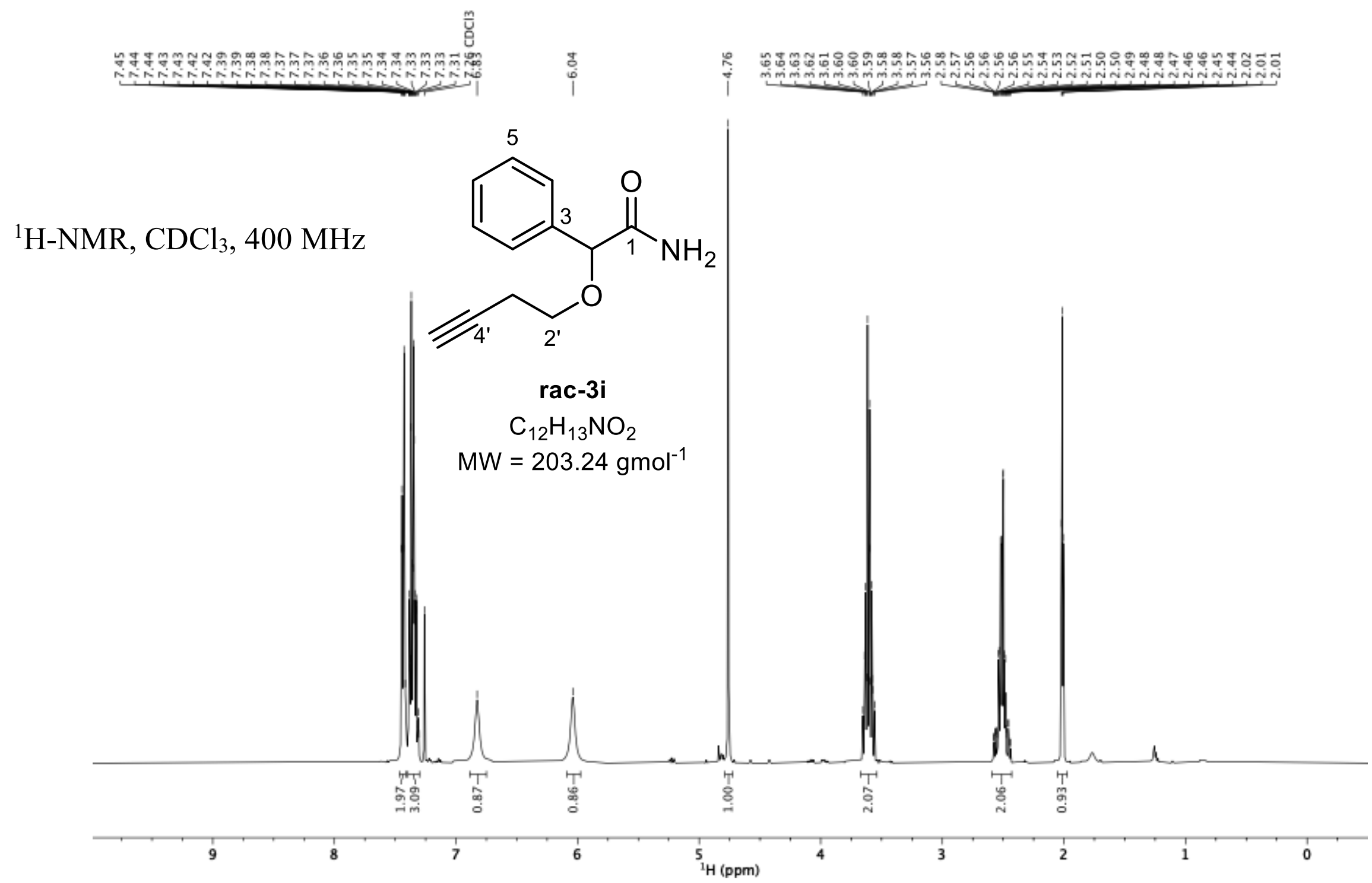

^{1}H-NMR, $CDCl_3$, 400 MHz
5
3
1
NH_2
O
4'
2'
rac-3i
$C_{12}H_{13}NO_2$
MW = 203.24 gmol^{-1}
^{1}H (ppm)

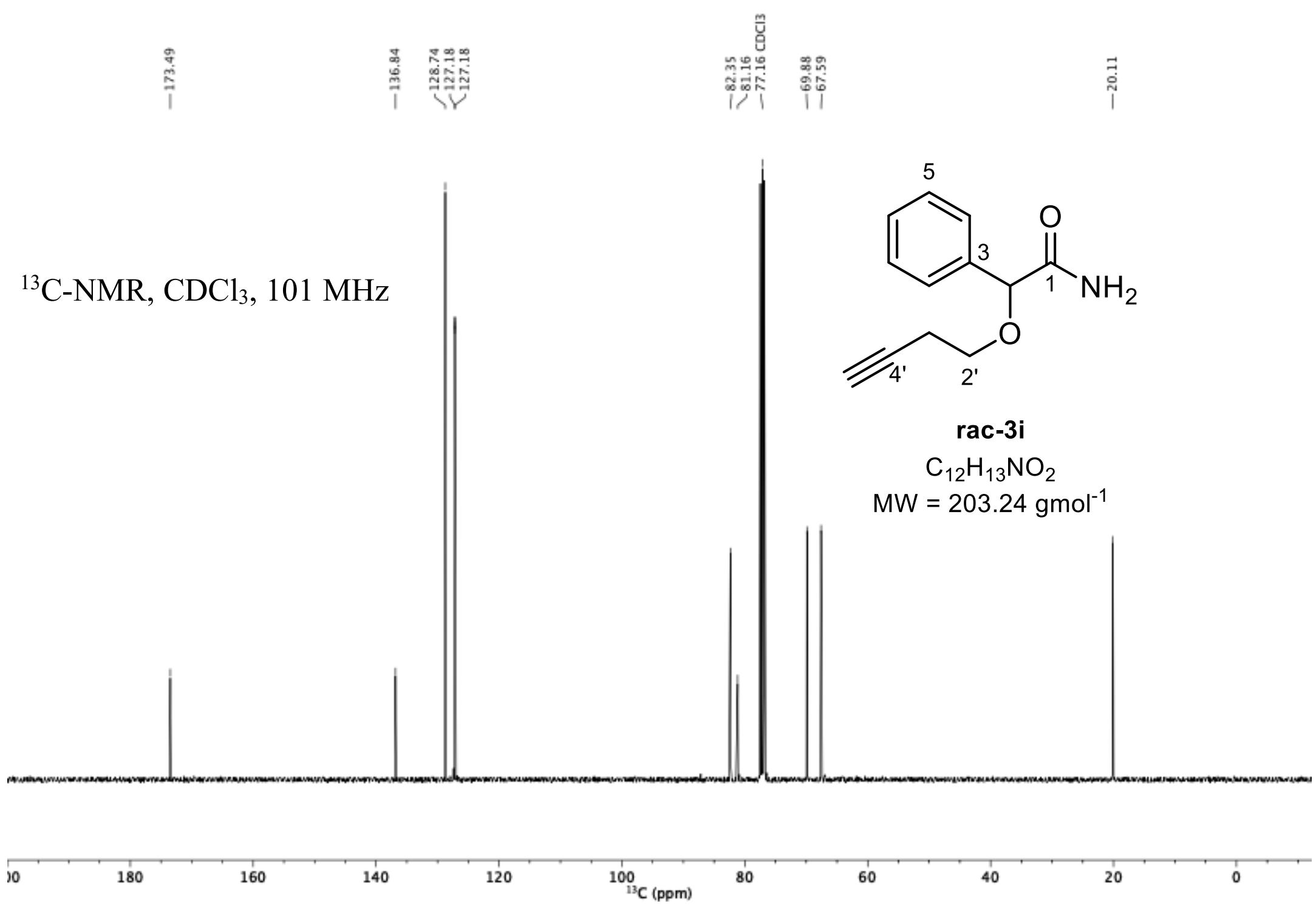

^{13}C-NMR, $CDCl_3$, 101 MHz
173.49
136.84
128.74
127.18
127.18
82.35
81.16
77.16 CDCl3
69.88
67.59
20.11
5
3
1
NH_2
O
4'
2'
rac-3i
$C_{12}H_{13}NO_2$
MW = 203.24 gmol^{-1}
^{13}C (ppm)

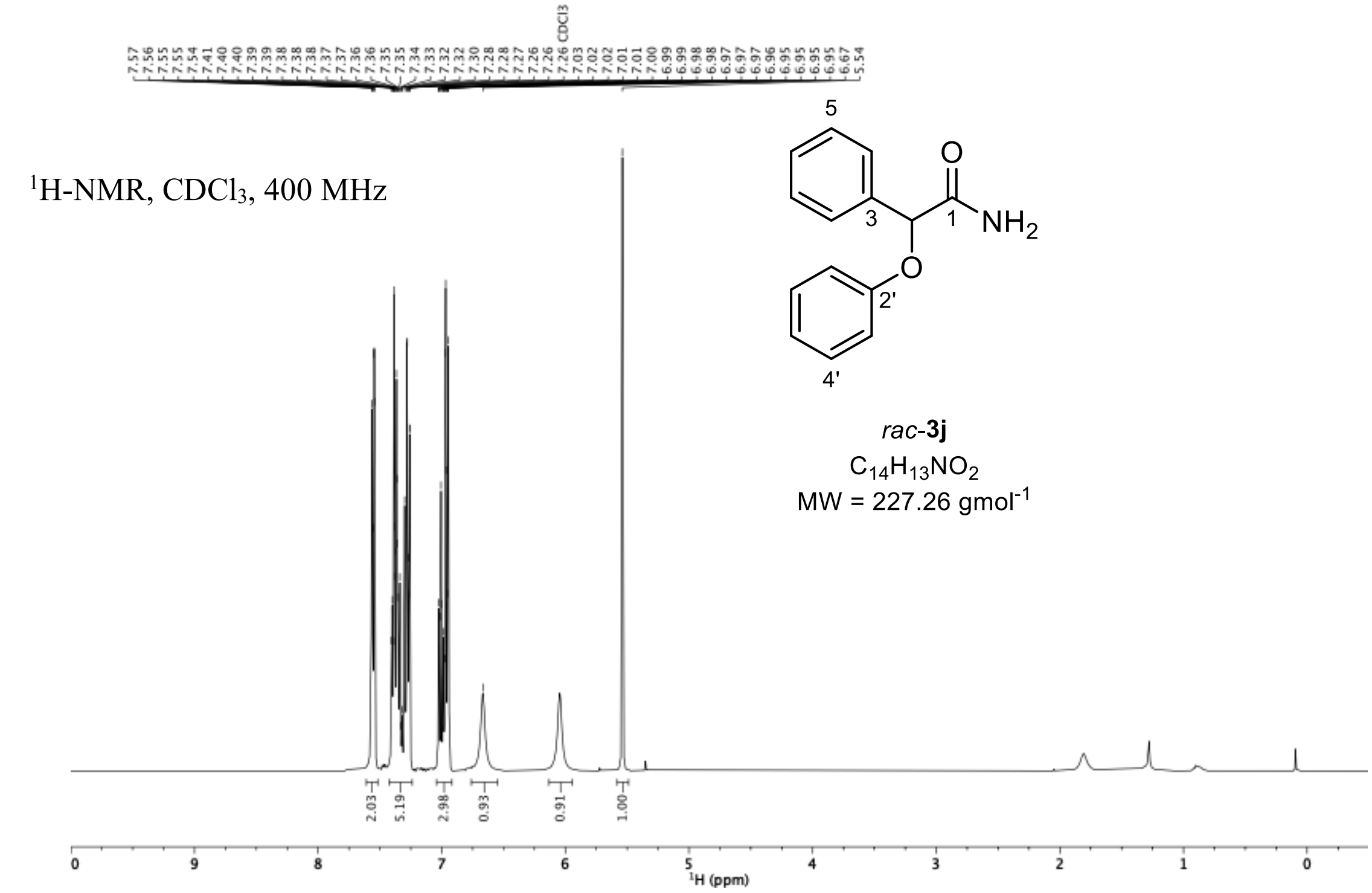
7.57 7.56 7.55 7.54 7.41 7.40 7.39 7.39 7.38 7.38 7.38 7.37 7.37 7.36 7.35 7.35 7.34 7.33 7.32 7.32 7.30 7.28 7.28 7.27 7.26 7.26 CDCl3 7.03 7.02 7.02 7.01 7.01 7.00 6.99 6.99 6.98 6.98 6.97 6.97 6.97 6.96 6.95 6.95 6.95 6.95 6.67 5.54
^{1}H-NMR, $CDCl_3$, 400 MHz
5
O
3
1
NH_2
O
2'
4'
rac-**3j**
$C_{14}H_{13}NO_2$
MW = 227.26 $gmol^{-1}$
2.03
5.19
2.98
0.93
0.91
1.00
^{1}H (ppm)

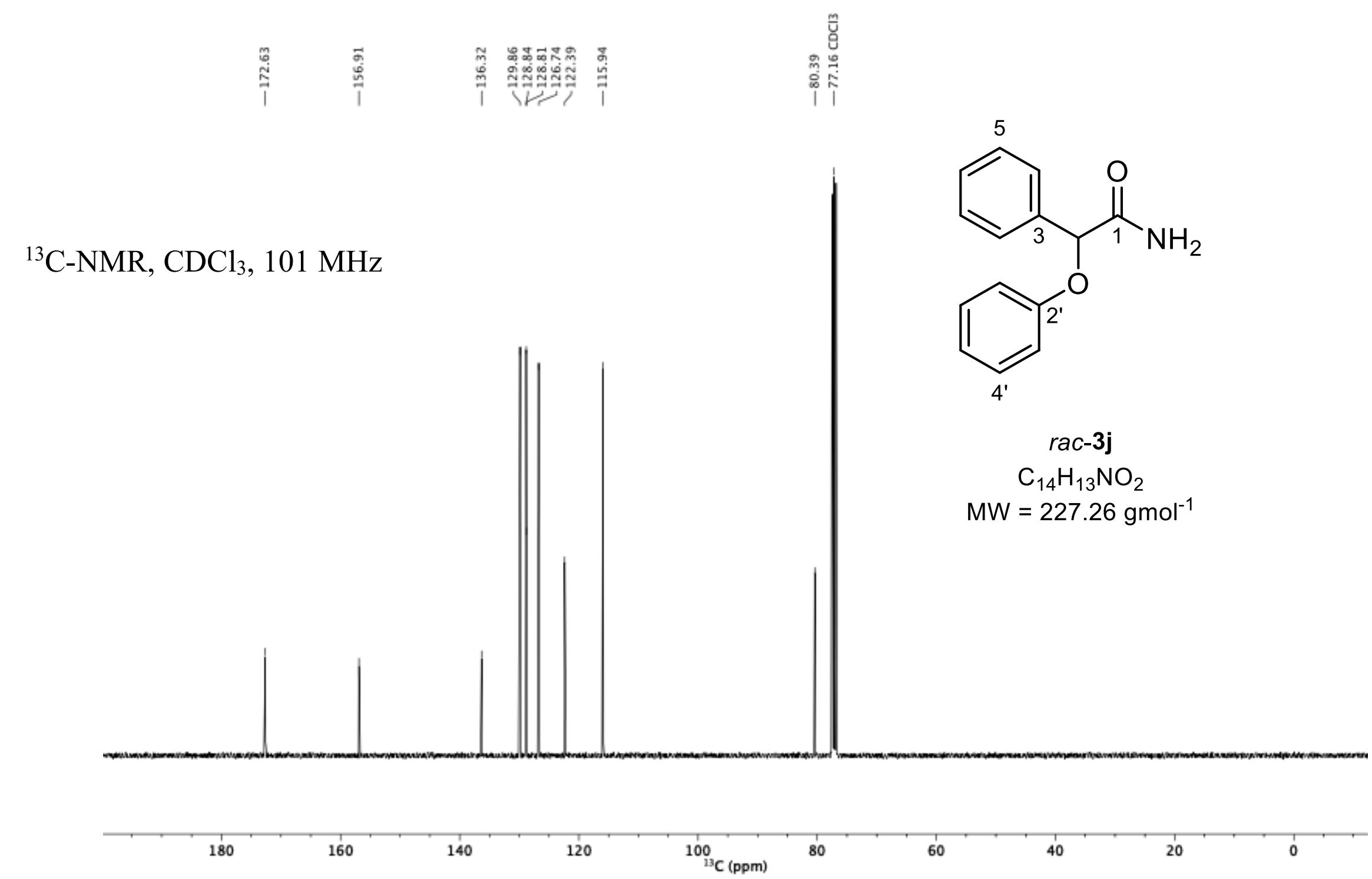
172.63
156.91
136.32
129.86 128.84 128.81 126.74 122.39
115.94
80.39
77.16 CDCl3
^{13}C-NMR, $CDCl_3$, 101 MHz
5
O
3
1
NH_2
O
2'
4'
rac-**3j**
$C_{14}H_{13}NO_2$
MW = 227.26 $gmol^{-1}$
^{13}C (ppm)

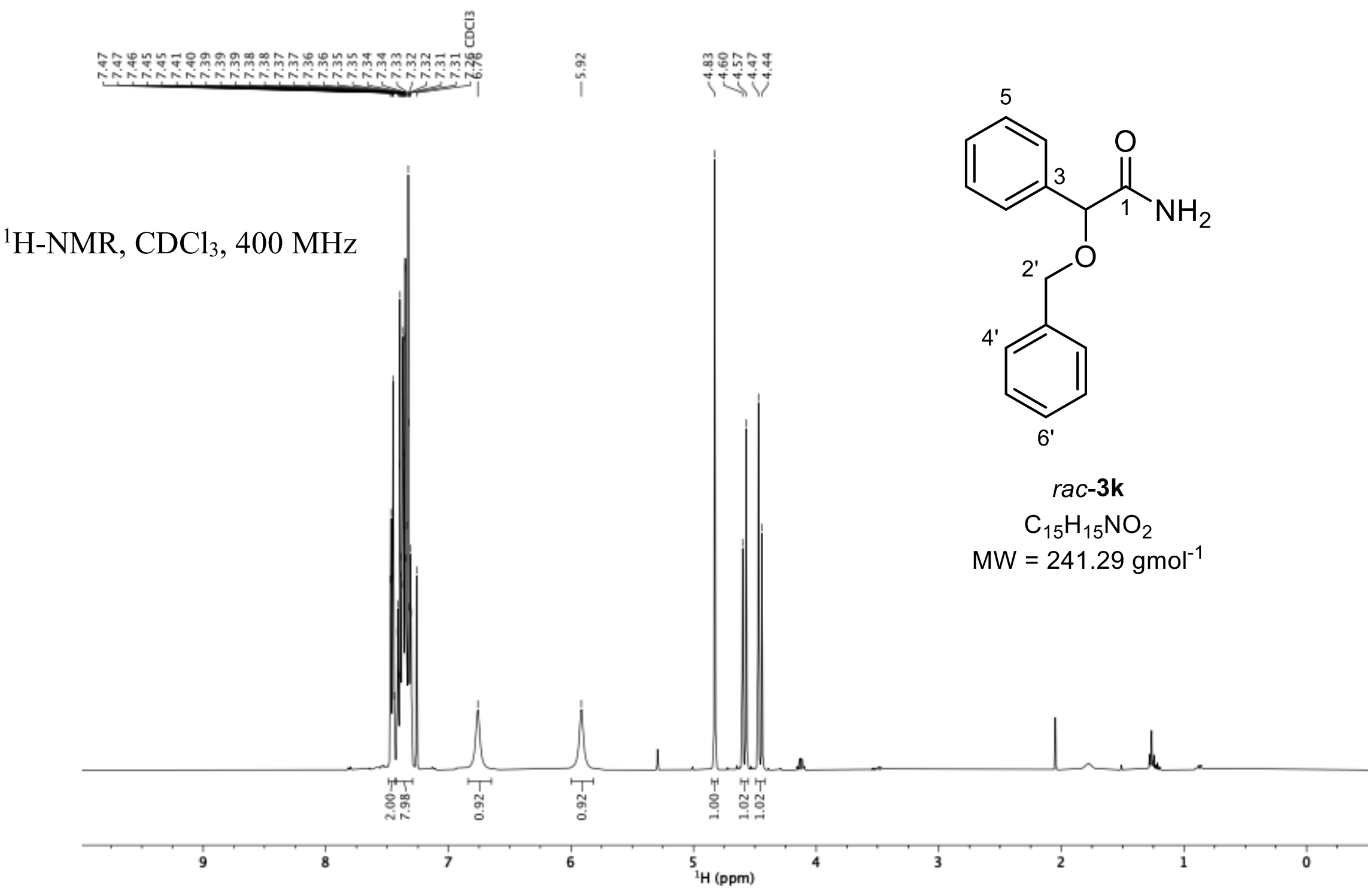
^{1}H-NMR, $CDCl_3$, 400 MHz
rac-**3k**
$C_{15}H_{15}NO_2$
MW = 241.29 gmol^{-1}
^{1}H (ppm)

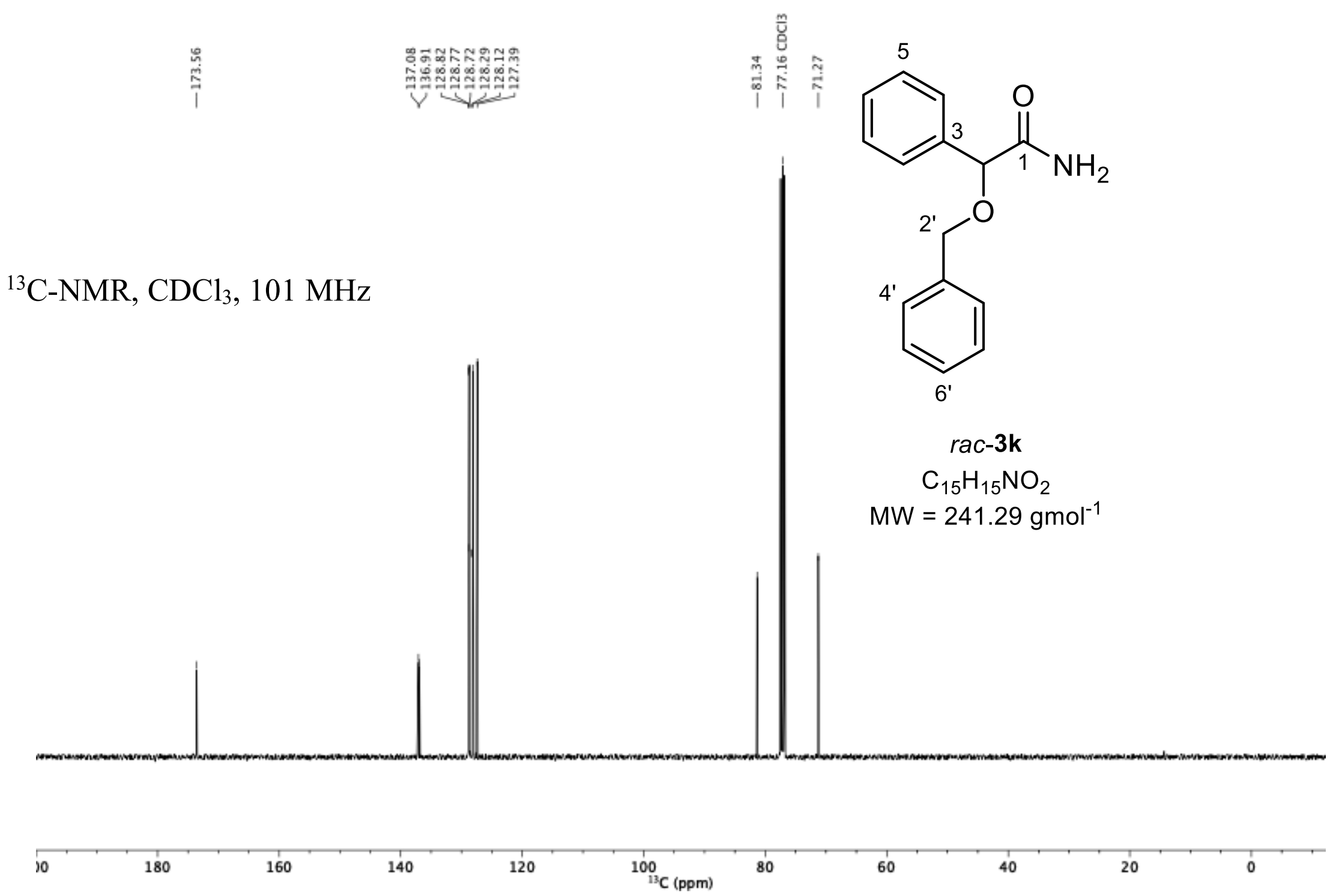
^{13}C-NMR, $CDCl_3$, 101 MHz
rac-**3k**
$C_{15}H_{15}NO_2$
MW = 241.29 gmol^{-1}
^{13}C (ppm)

¹H-NMR, CDCl$_3$, 400 MHz

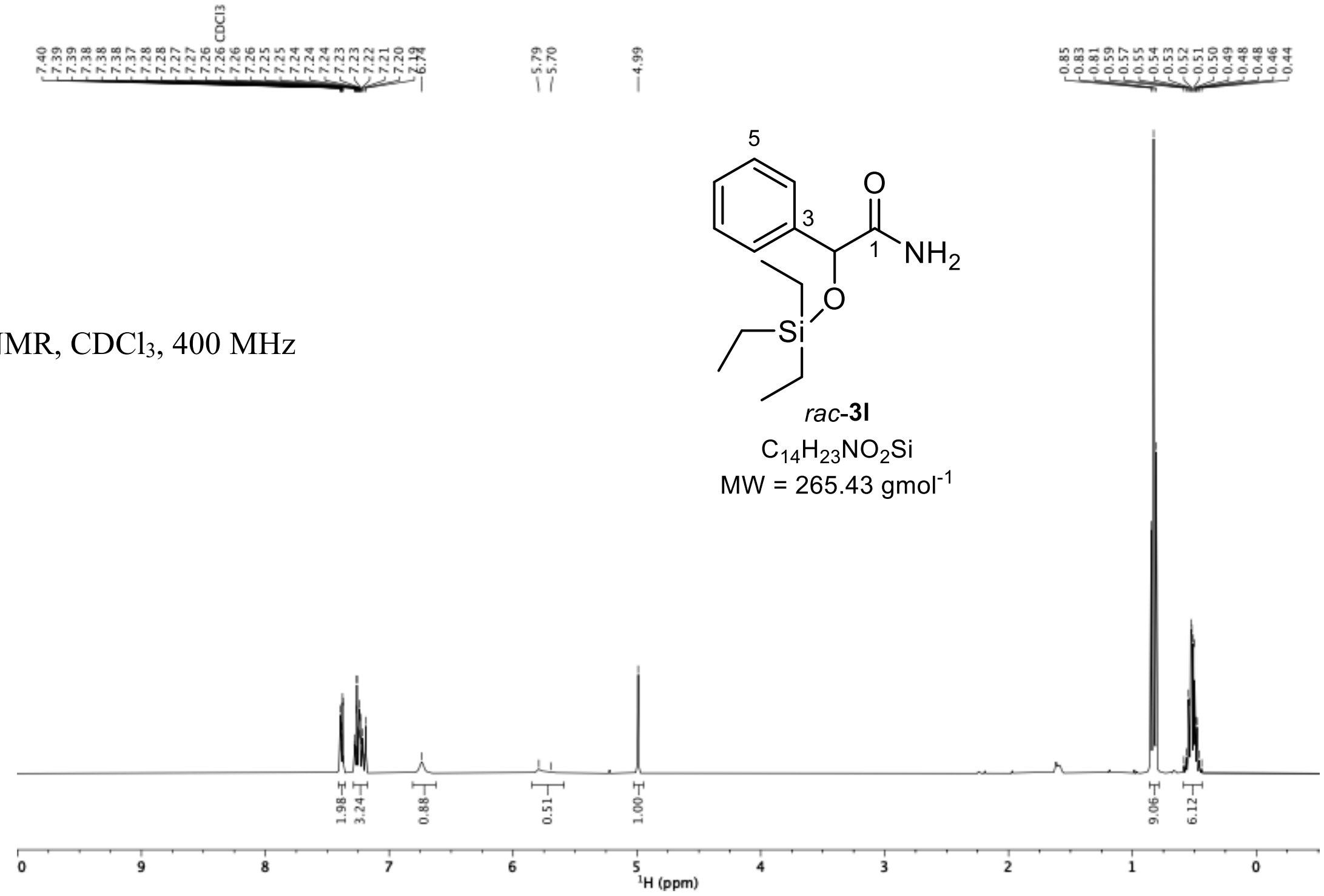

^{13}C-NMR, CDCl$_3$, 101 MHz

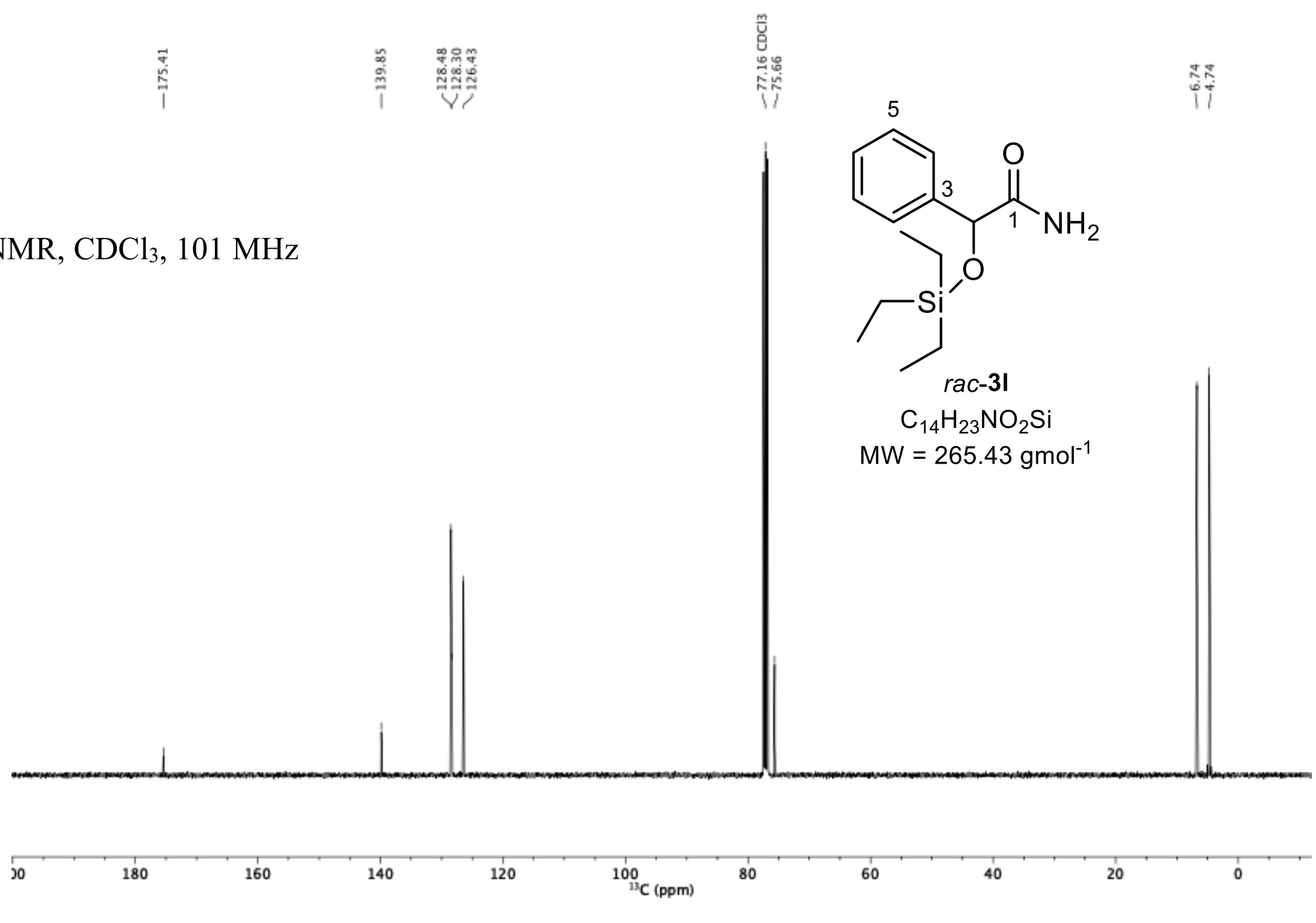

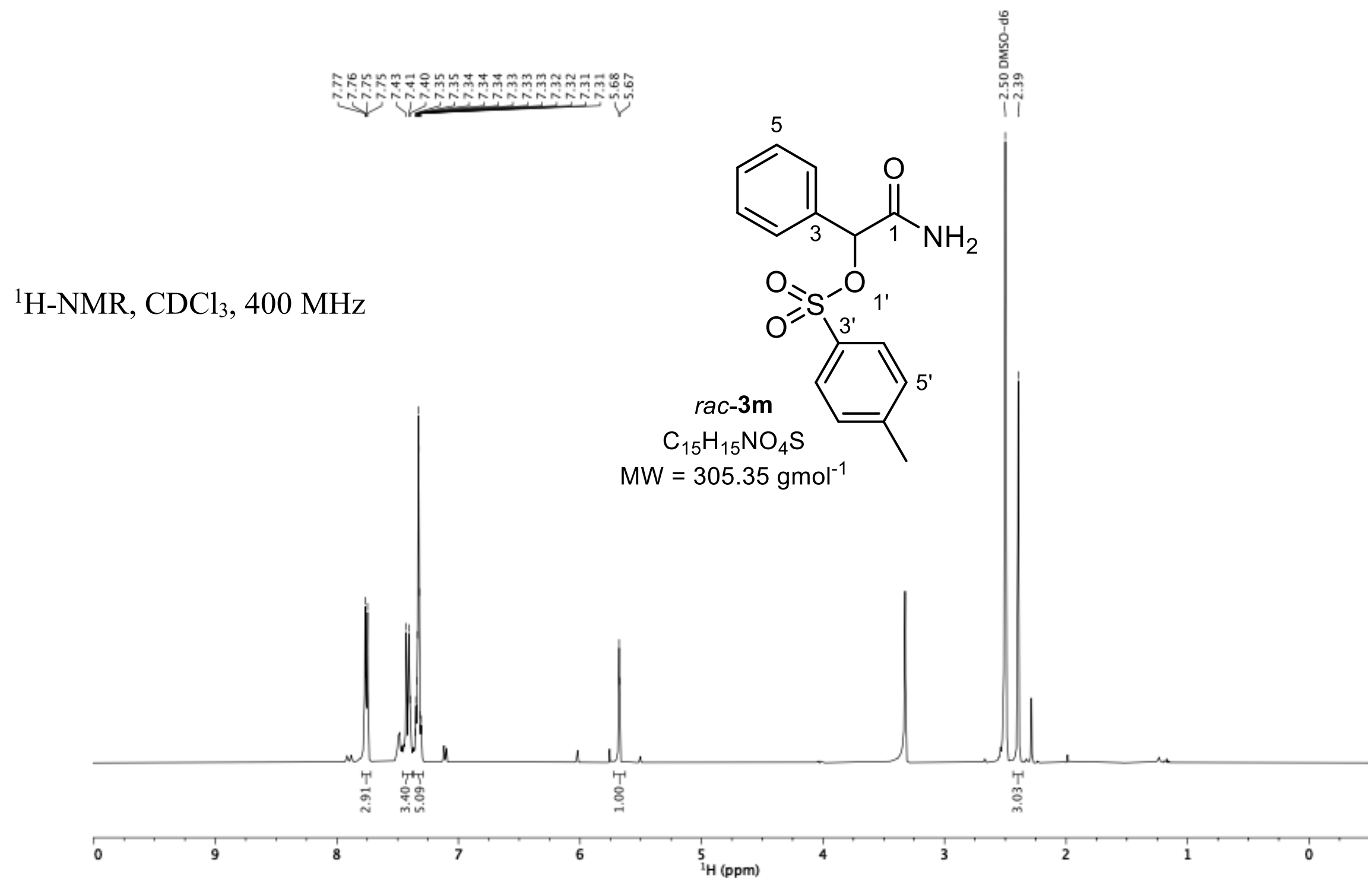

¹H-NMR, CDCl₃, 400 MHz
rac-3m
C15H15NO4S
MW = 305.35 gmol-1
¹H (ppm)

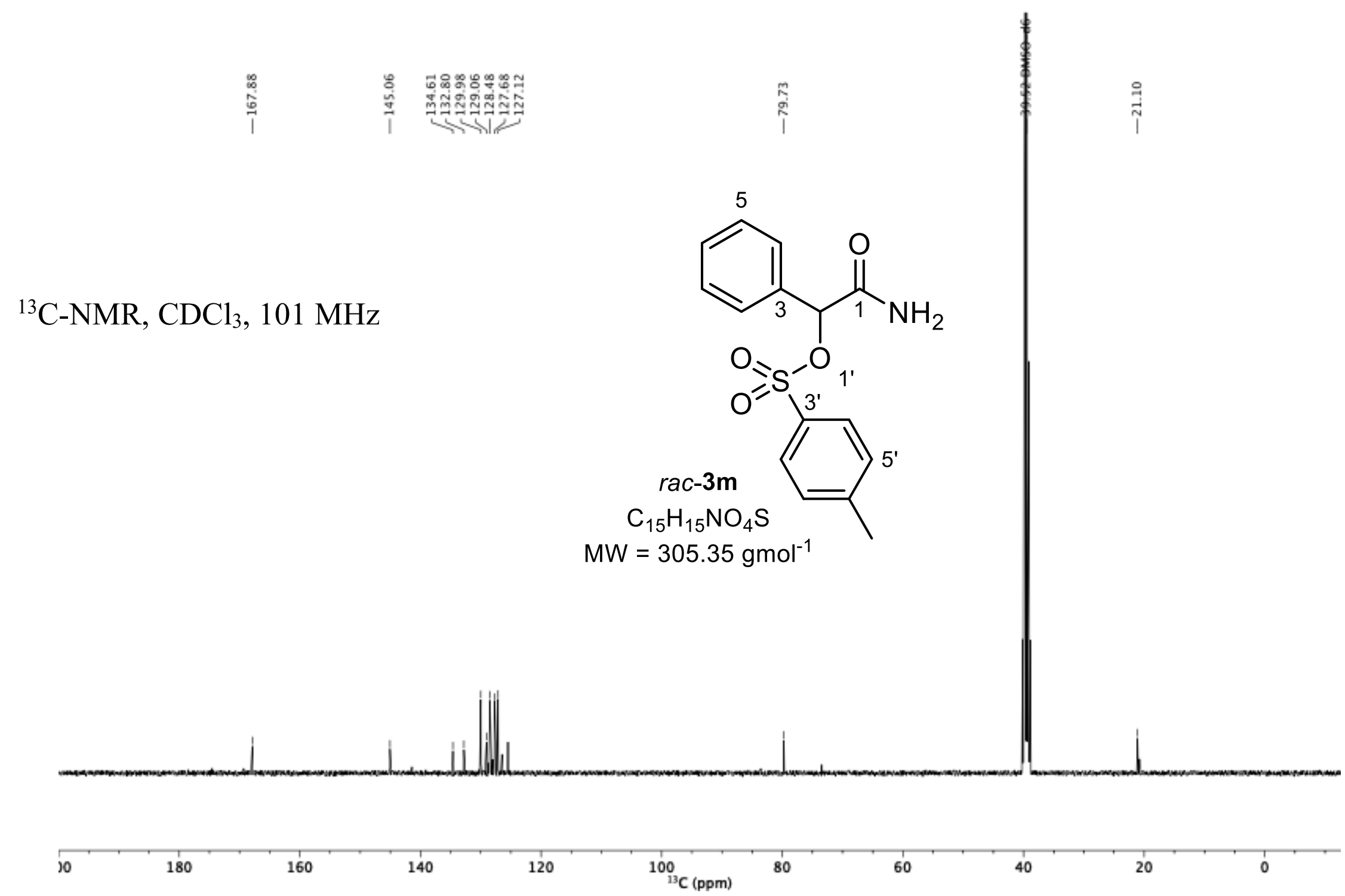

¹³C-NMR, CDCl₃, 101 MHz
rac-3m
C15H15NO4S
MW = 305.35 gmol-1
¹³C (ppm)

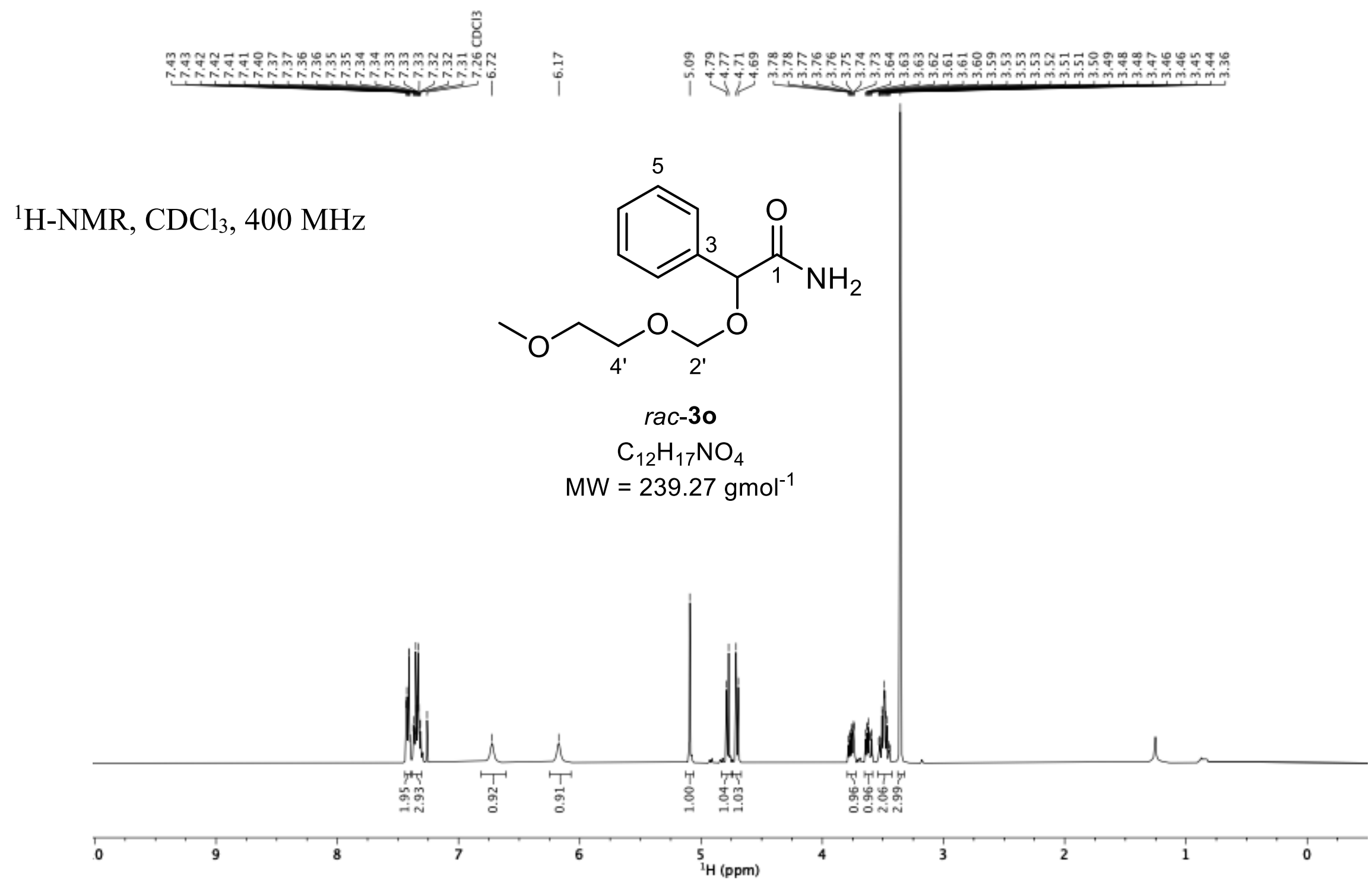
1H-NMR, CDCl3, 400 MHz
rac-3o
C12H17NO4
MW = 239.27 gmol-1
1H (ppm)

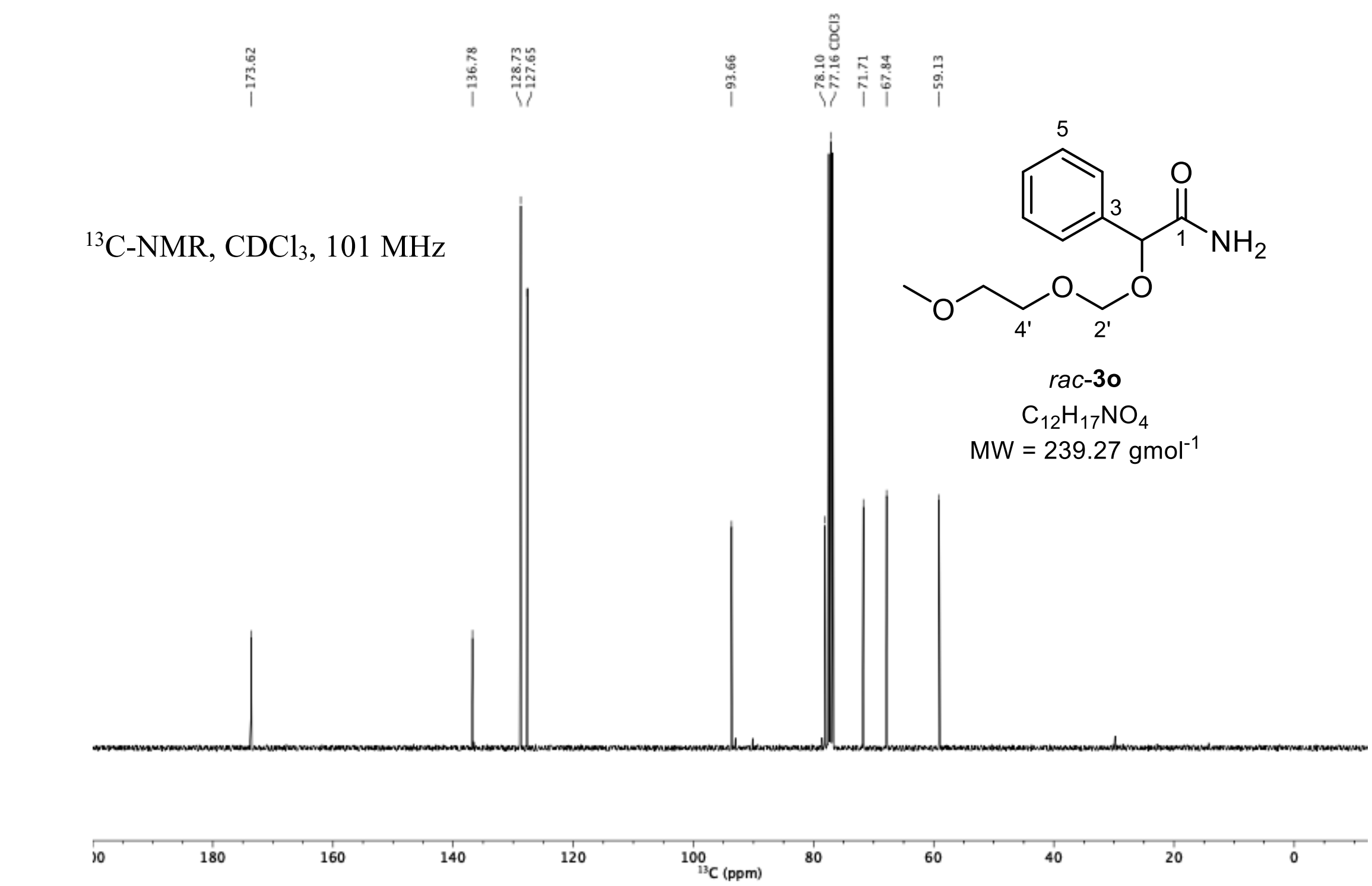
13C-NMR, CDCl3, 101 MHz
173.62
136.78
128.73
127.65
93.66
78.10
77.16 CDCl3
71.71
67.84
59.13
rac-3o
C12H17NO4
MW = 239.27 gmol-1
13C (ppm)

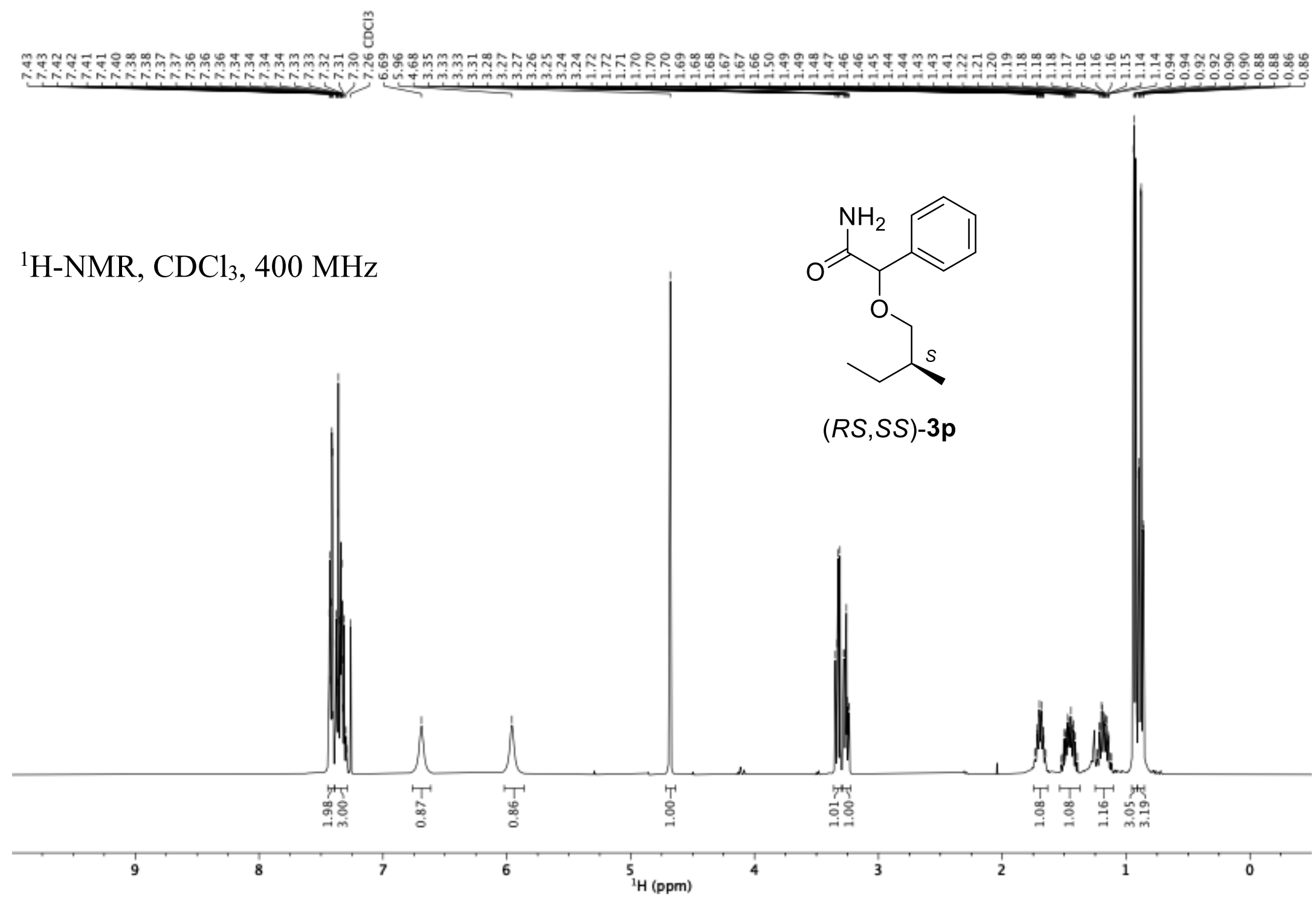

1H-NMR, CDCl3, 400 MHz
NH2
O
O
S
(RS,SS)-3p
1H (ppm)

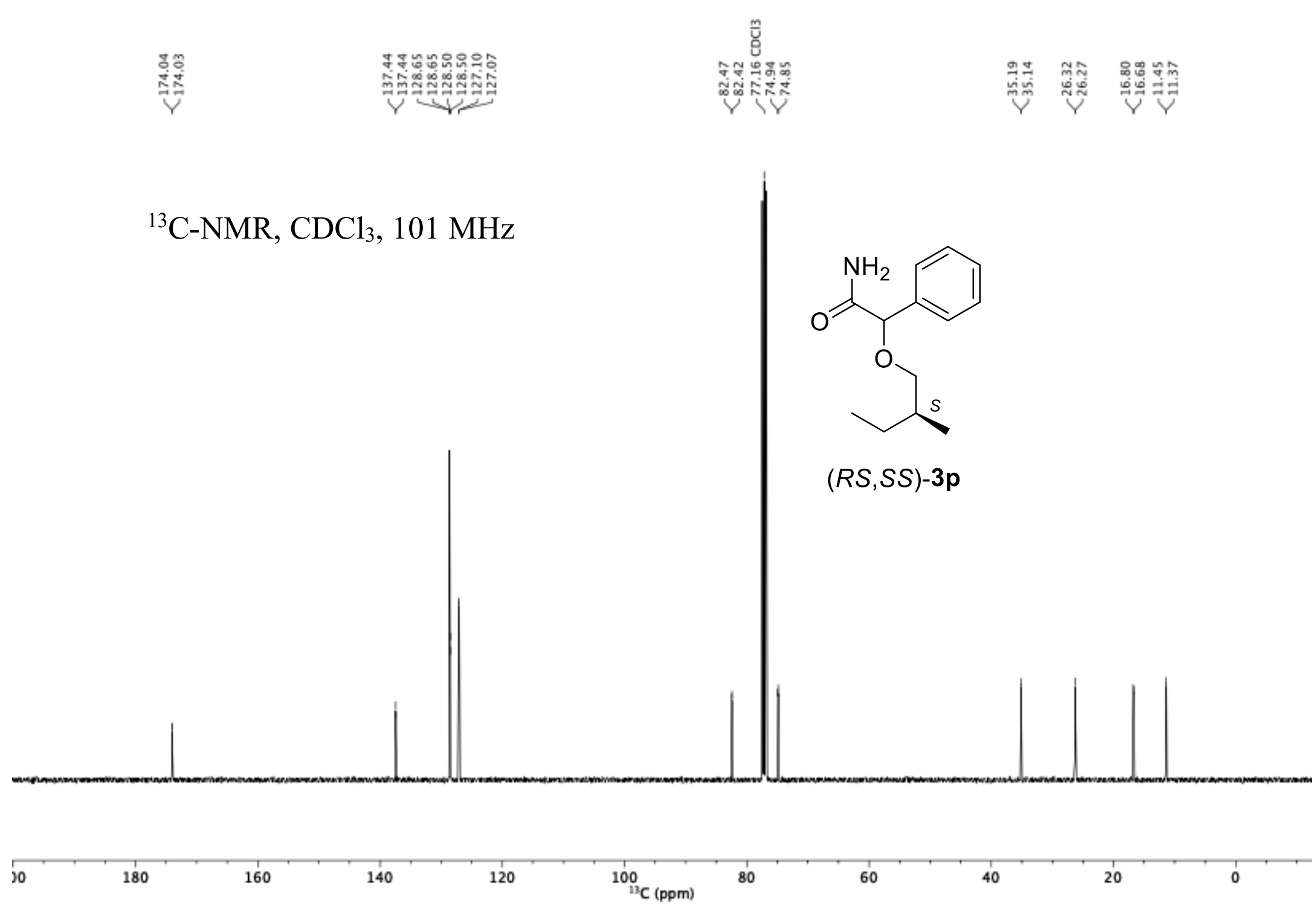

13C-NMR, CDCl3, 101 MHz
NH2
O
O
S
(RS,SS)-3p
13C (ppm)

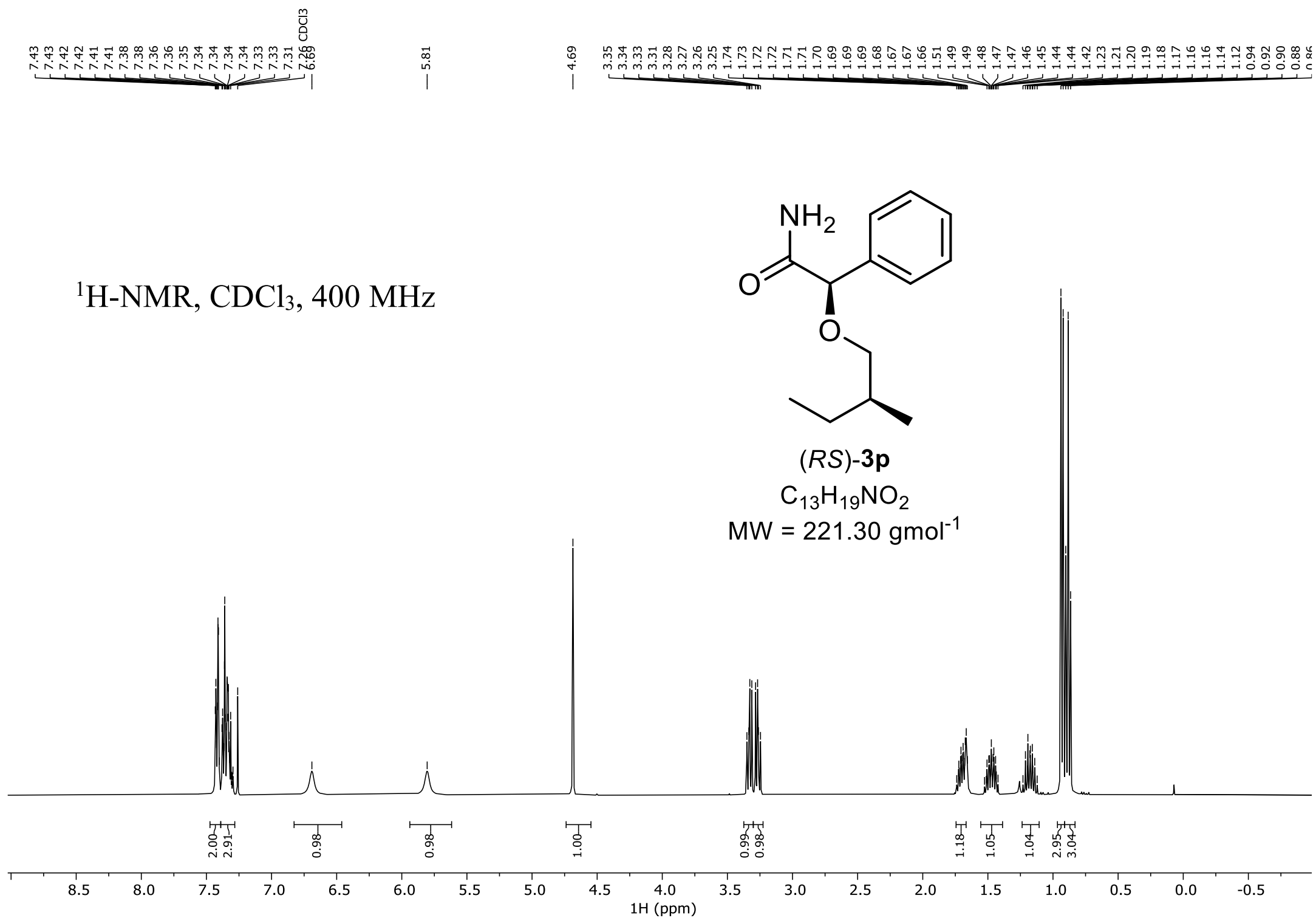
1H-NMR, CDCl3, 400 MHz
NH2
O
O
(RS)-3p
C13H19NO2
MW = 221.30 gmol-1
1H (ppm)

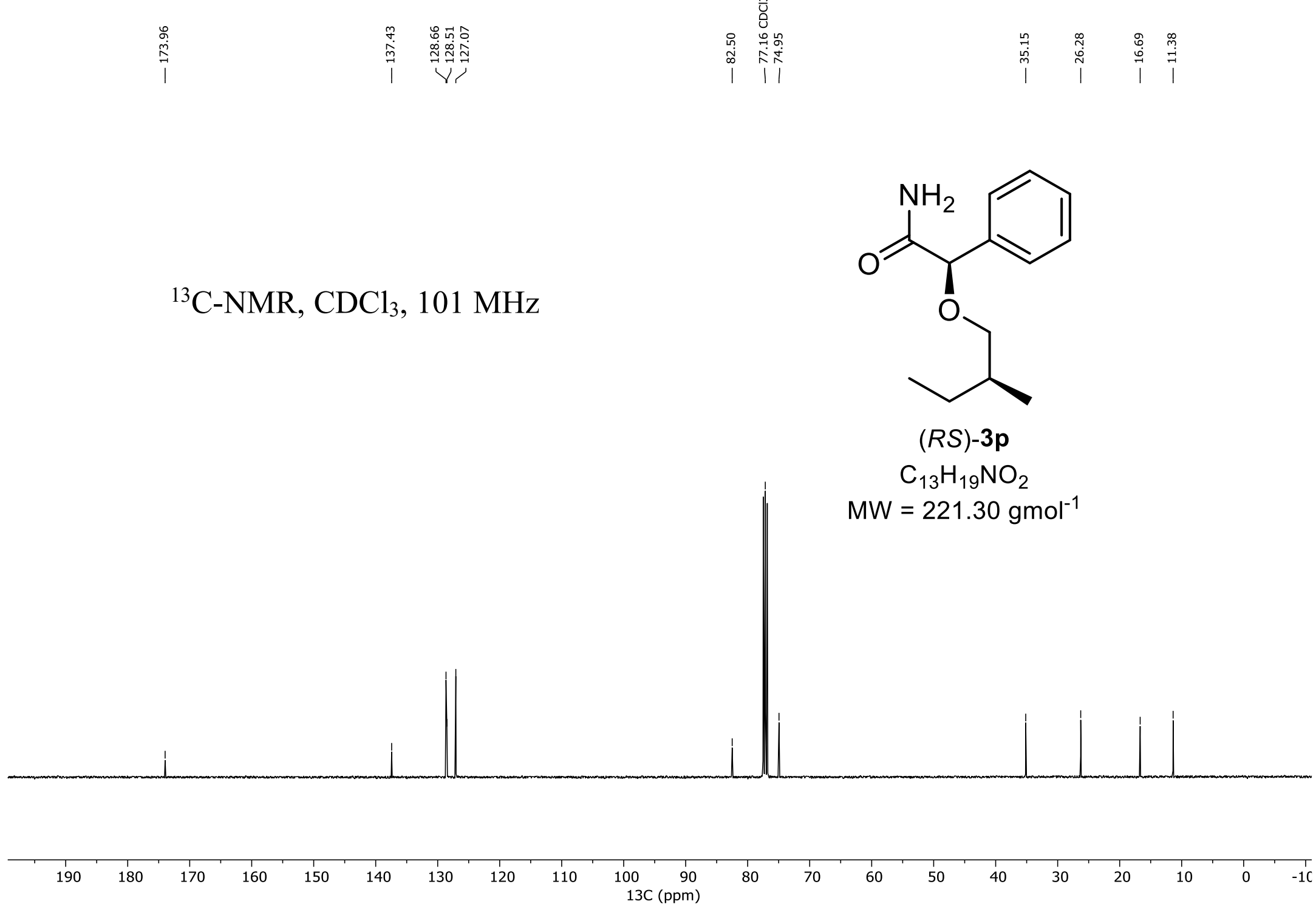
13C-NMR, CDCl3, 101 MHz
173.96
137.43
128.66
128.51
127.07
82.50
77.16 CDCl3
74.95
35.15
26.28
16.69
11.38
NH2
O
O
(RS)-3p
C13H19NO2
MW = 221.30 gmol-1
13C (ppm)

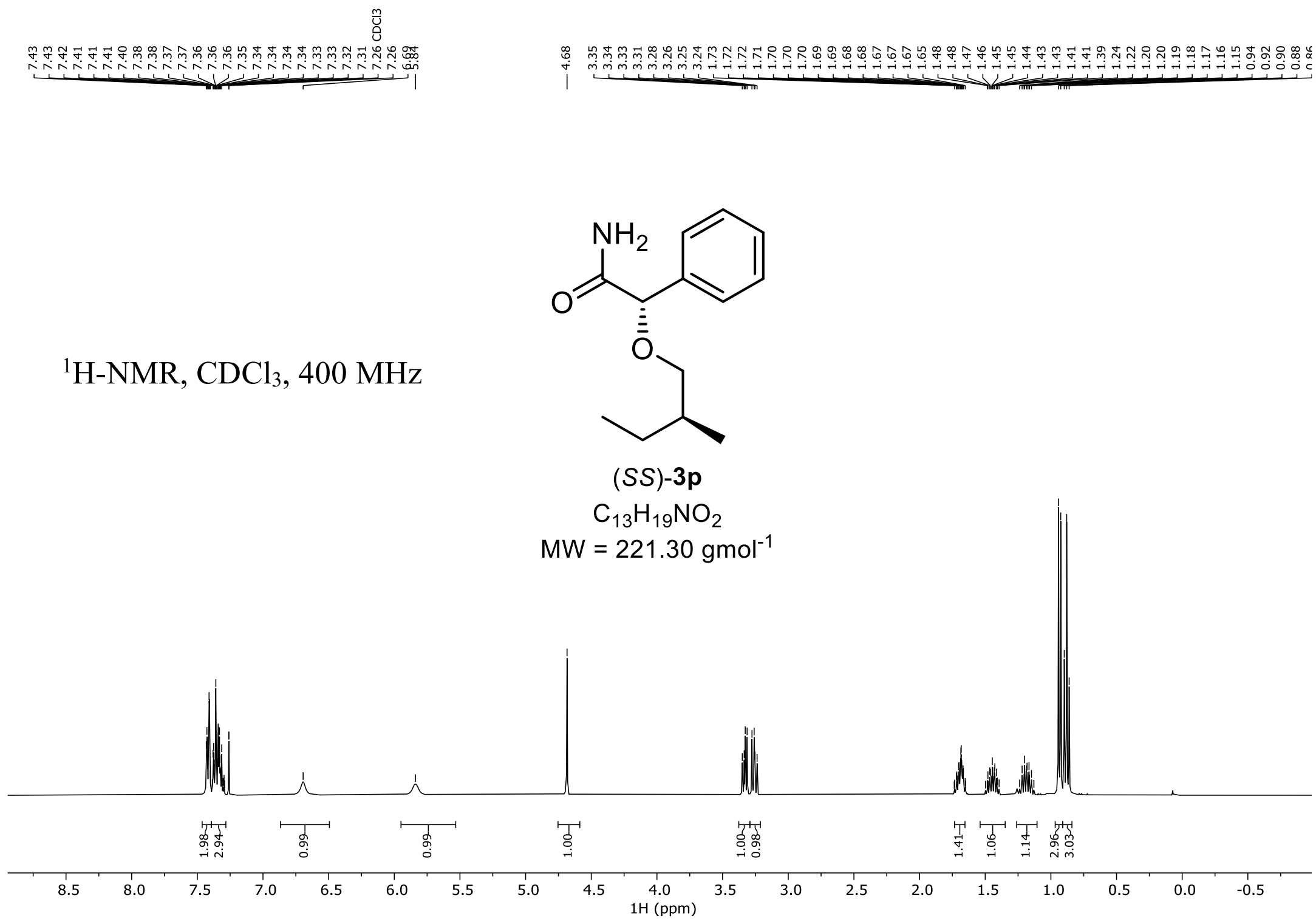

1H-NMR, CDCl3, 400 MHz
NH2
O
(SS)-3p
C13H19NO2
MW = 221.30 gmol-1
1H (ppm)

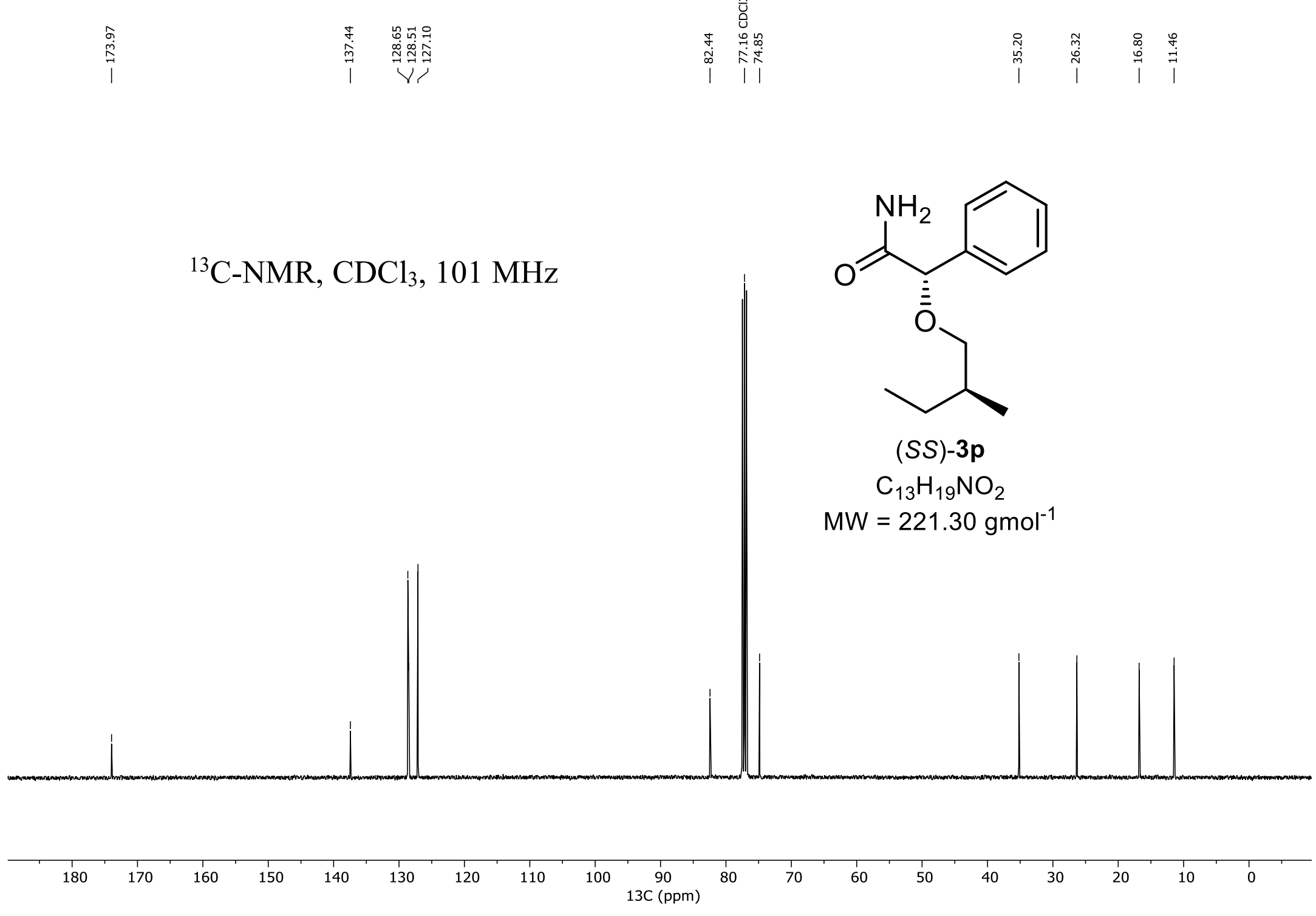

13C-NMR, CDCl3, 101 MHz
173.97
137.44
128.65
128.51
127.10
82.44
77.16 CDCl3
74.85
35.20
26.32
16.80
11.46
NH2
O
(SS)-3p
C13H19NO2
MW = 221.30 gmol-1
13C (ppm)

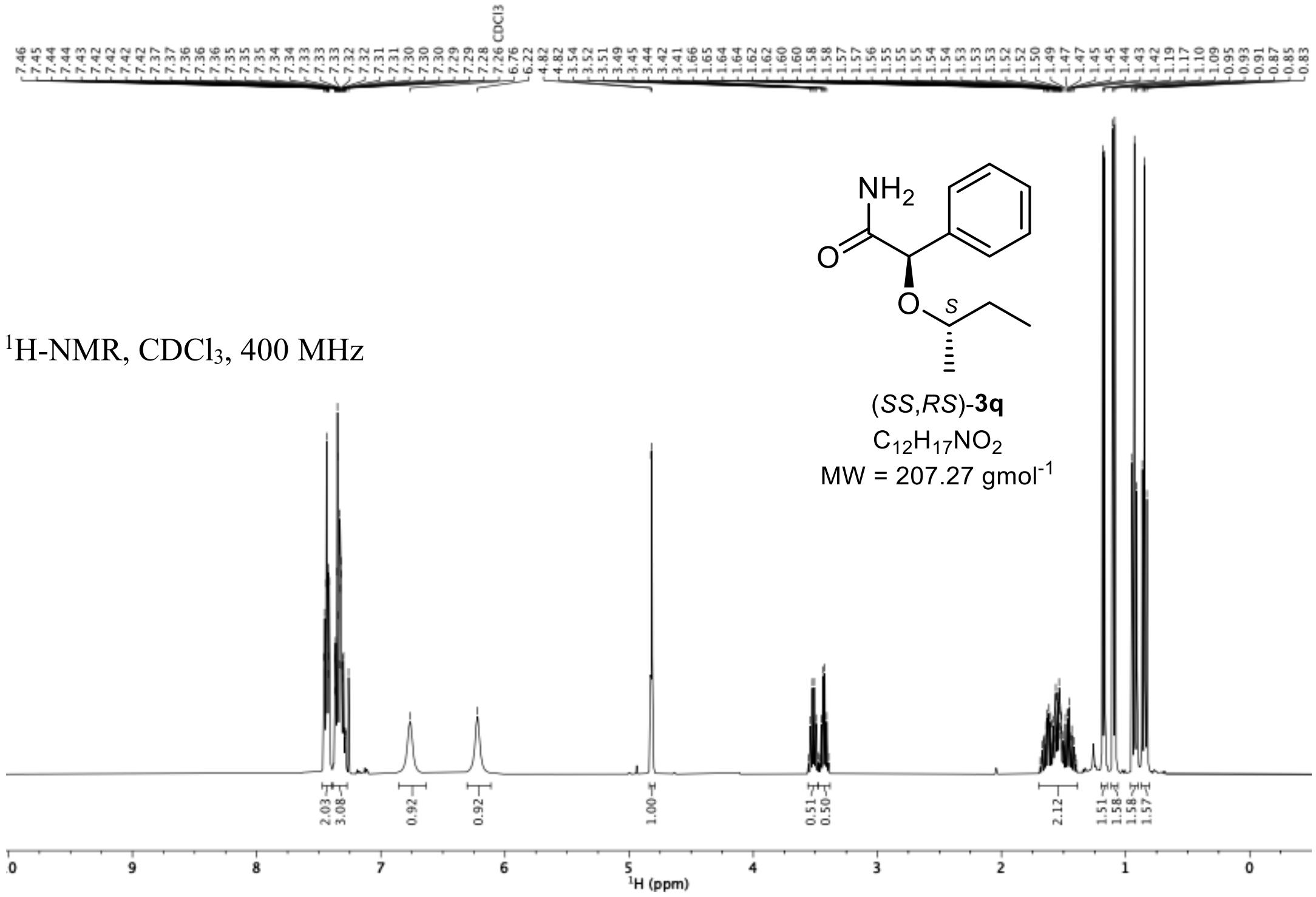
7.46 7.45 7.44 7.43 7.42 7.42 7.42 7.37 7.37 7.36 7.36 7.36 7.35 7.35 7.35 7.34 7.34 7.33 7.33 7.33 7.32 7.32 7.31 7.31 7.30 7.30 7.29 7.29 7.28 7.26 CDCl3 6.76 6.22 4.82 4.82 3.54 3.52 3.51 3.49 3.45 3.44 3.42 3.41 1.66 1.65 1.64 1.64 1.62 1.62 1.60 1.60 1.58 1.58 1.57 1.57 1.56 1.55 1.55 1.55 1.54 1.54 1.53 1.53 1.53 1.52 1.52 1.50 1.49 1.47 1.45 1.45 1.44 1.43 1.42 1.19 1.17 1.10 1.09 0.95 0.93 0.91 0.87 0.85 0.83
NH2
O
O
S
(SS,RS)-3q
C12H17NO2
MW = 207.27 gmol-1
1H-NMR, CDCl3, 400 MHz
2.03 3.08 0.92 0.92 1.00 0.51 0.50 2.12 1.51 1.58 1.58 1.57
1H (ppm)

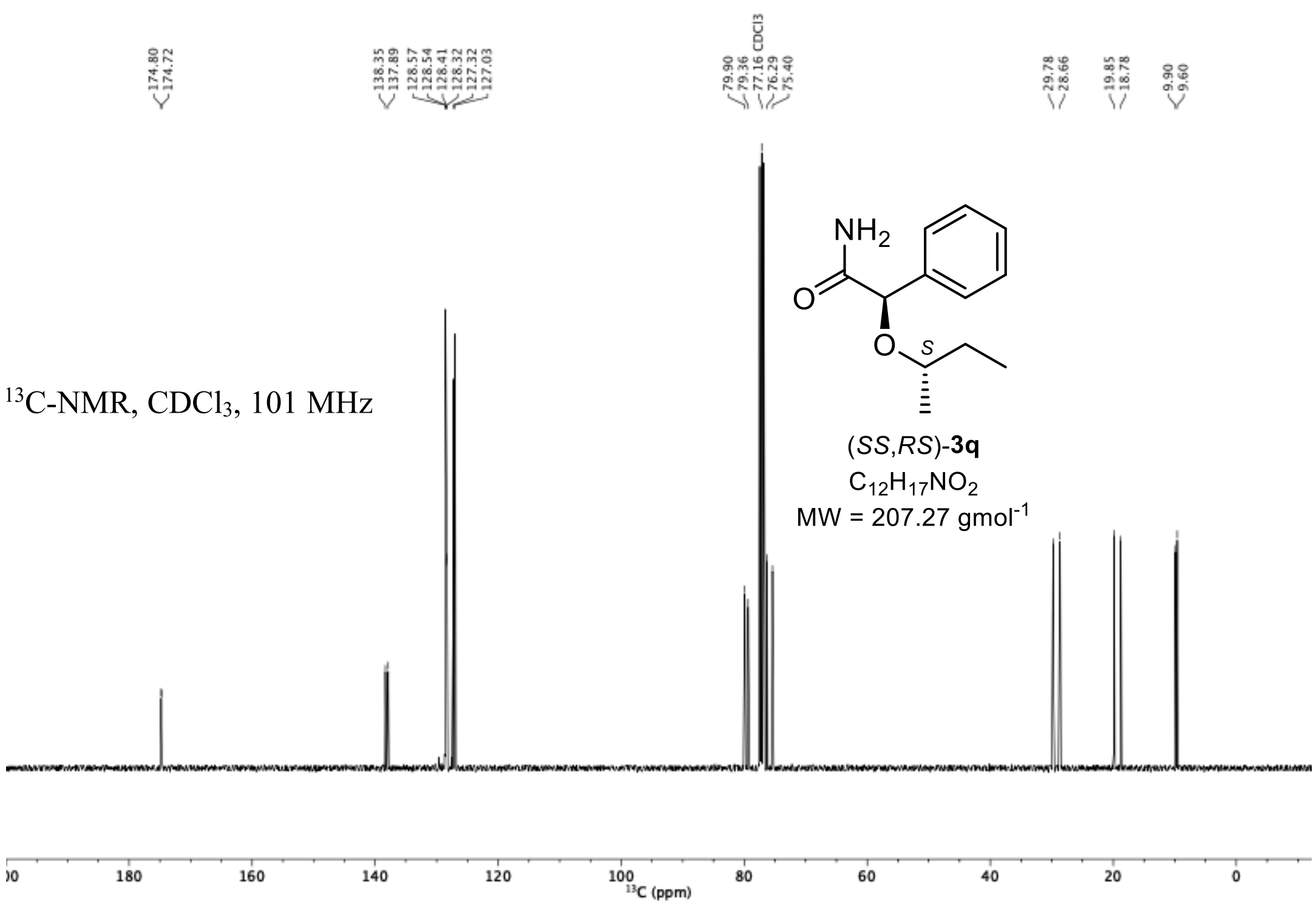
174.80 174.72 138.35 137.89 128.57 128.54 128.41 128.32 127.32 127.03 79.90 79.36 77.16 CDCl3 76.29 75.40 29.78 28.66 19.85 18.78 9.90 9.60
NH2
O
O
S
(SS,RS)-3q
C12H17NO2
MW = 207.27 gmol-1
13C-NMR, CDCl3, 101 MHz
13C (ppm)

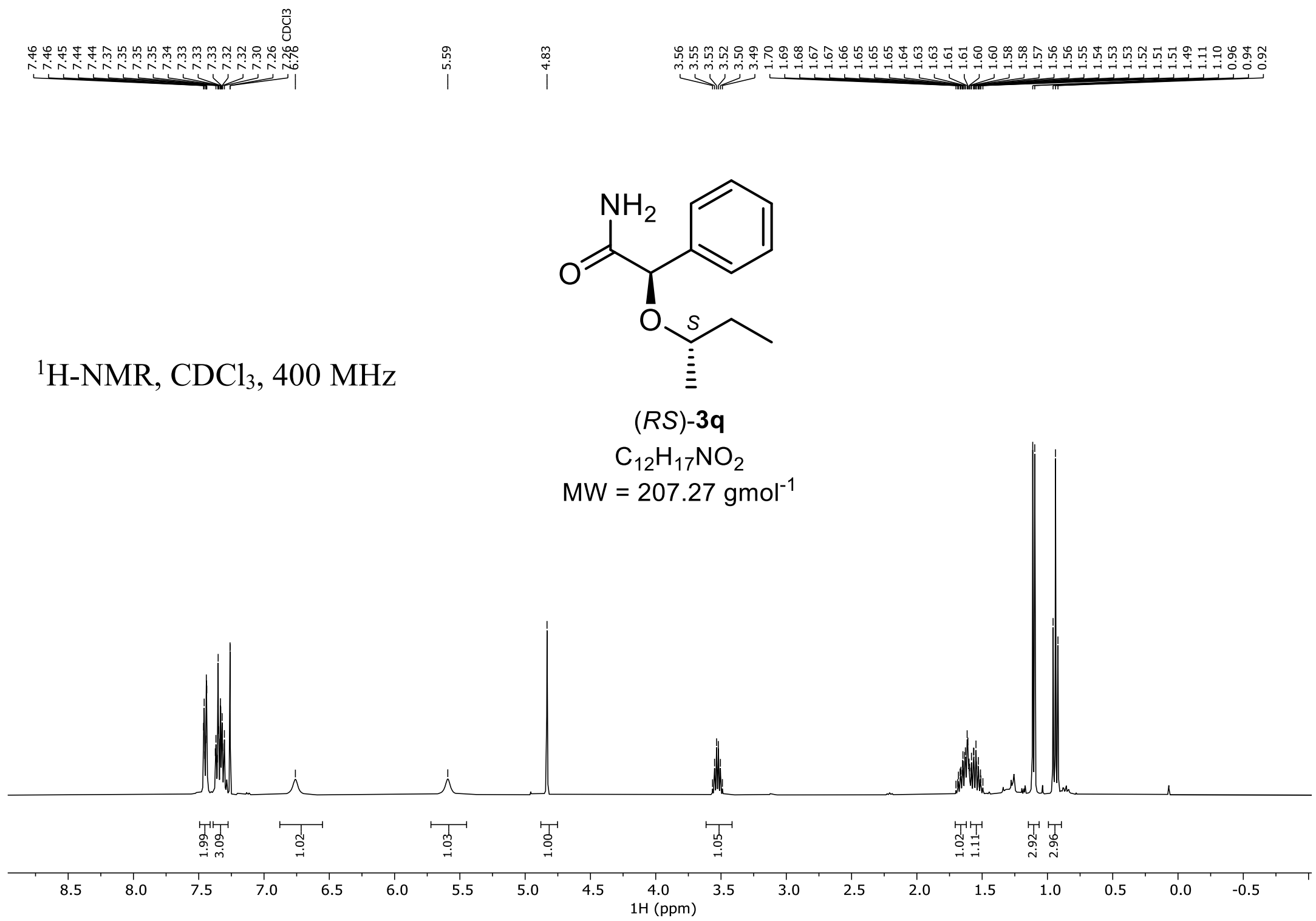
7.46 7.46 7.45 7.44 7.37 7.35 7.35 7.35 7.34 7.33 7.33 7.33 7.32 7.32 7.30 7.26 CDCl3 6.76
5.59
4.83
3.56 3.55 3.53 3.52 3.50 3.49 1.70 1.69 1.68 1.67 1.67 1.66 1.65 1.65 1.65 1.64 1.63 1.63 1.61 1.61 1.60 1.60 1.58 1.58 1.57 1.56 1.56 1.55 1.54 1.53 1.53 1.52 1.51 1.51 1.49 1.11 1.10 0.96 0.94 0.92
NH2
O
O
S
(RS)-3q
$C_{12}H_{17}NO_2$
MW = 207.27 gmol-1
^{1}H-NMR, $CDCl_3$, 400 MHz
1.99 3.09 1.02 1.03 1.00 1.05 1.02 1.11 2.92 2.96
8.5 8.0 7.5 7.0 6.5 6.0 5.5 5.0 4.5 4.0 3.5 3.0 2.5 2.0 1.5 1.0 0.5 0.0 -0.5
1H (ppm)

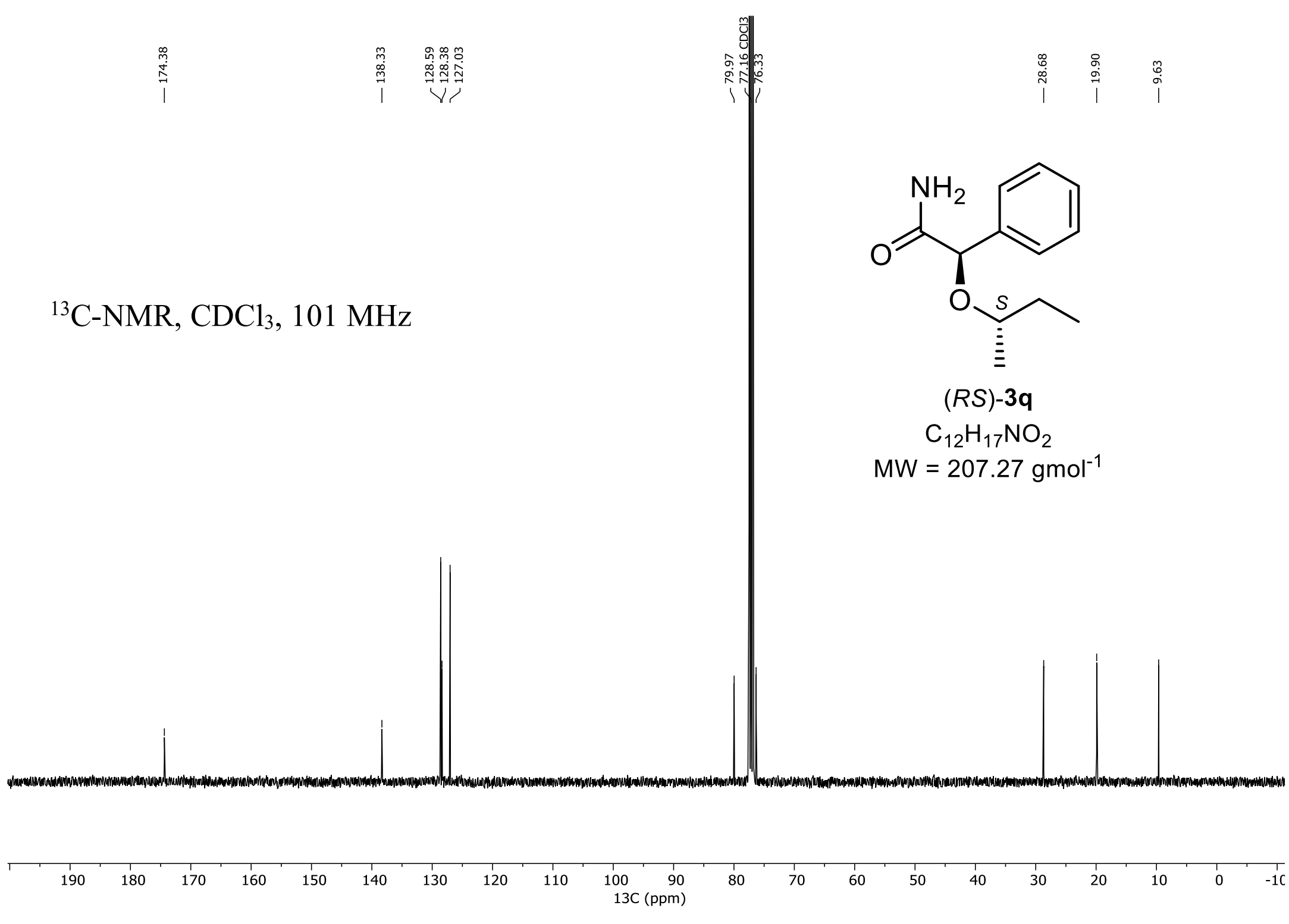
174.38
138.33
128.59 128.38 127.03
79.97 77.16 CDCl3 76.33
28.68
19.90
9.63
NH2
O
O
S
(RS)-3q
$C_{12}H_{17}NO_2$
MW = 207.27 gmol-1
^{13}C-NMR, $CDCl_3$, 101 MHz
190 180 170 160 150 140 130 120 110 100 90 80 70 60 50 40 30 20 10 0 -10
13C (ppm)

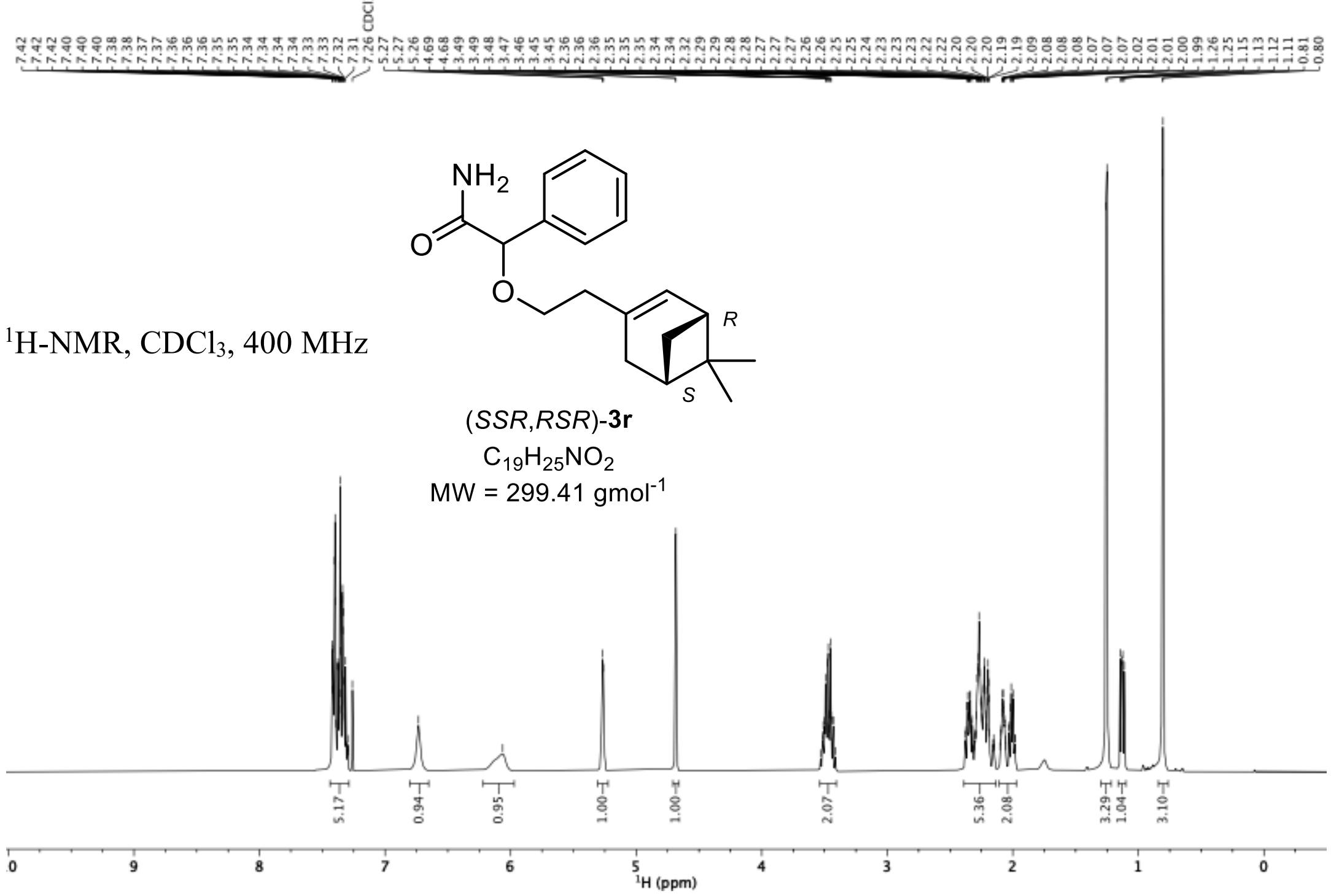

^{1}H-NMR, CDCl$_3$, 400 MHz
NH$_2$
O
O
R
S
(SSR,RSR)-3r
$C_{19}H_{25}NO_2$
MW = 299.41 gmol^{-1}
^{1}H (ppm)

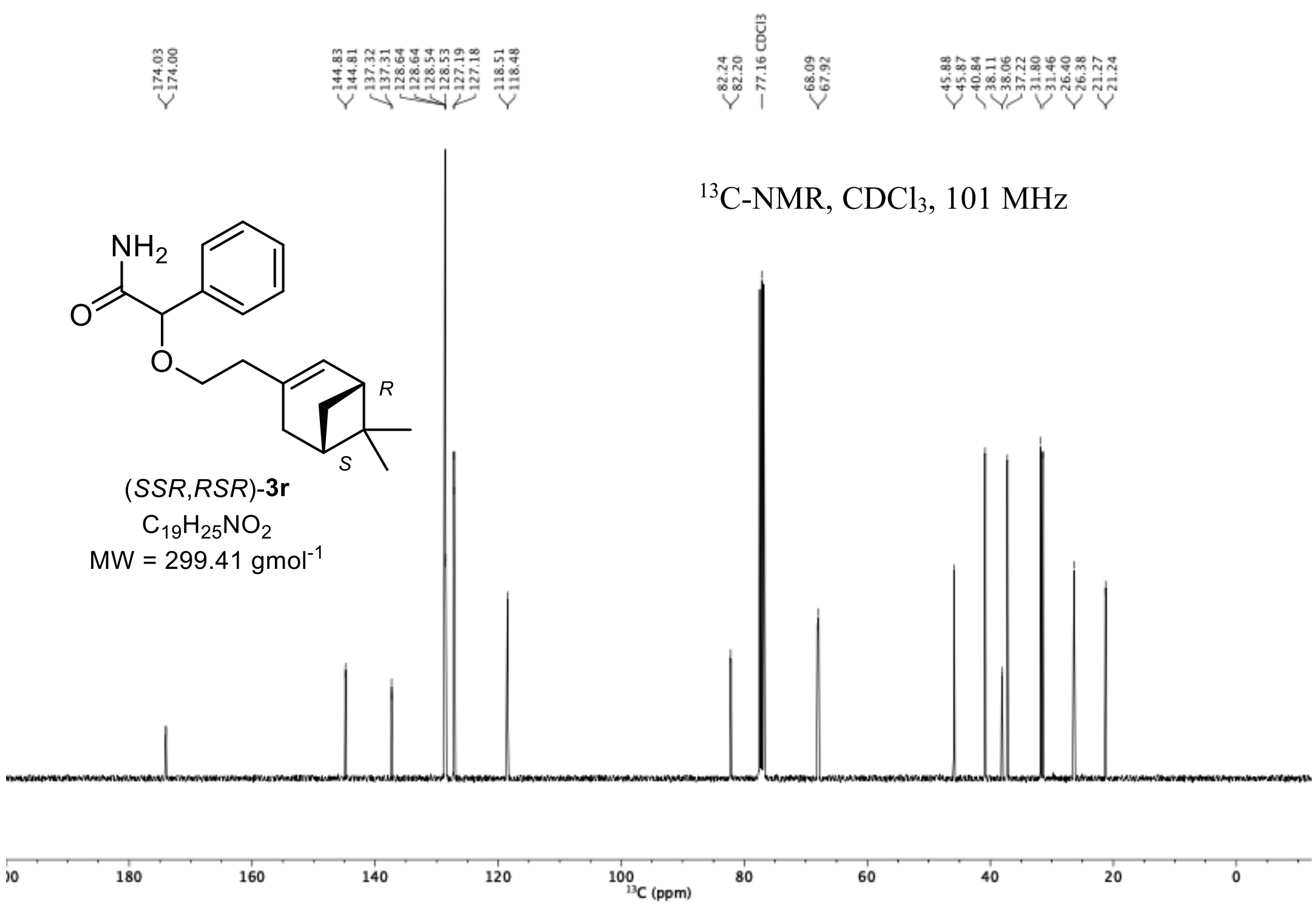

^{13}C-NMR, CDCl$_3$, 101 MHz
NH$_2$
O
O
R
S
(SSR,RSR)-3r
$C_{19}H_{25}NO_2$
MW = 299.41 gmol^{-1}
^{13}C (ppm)

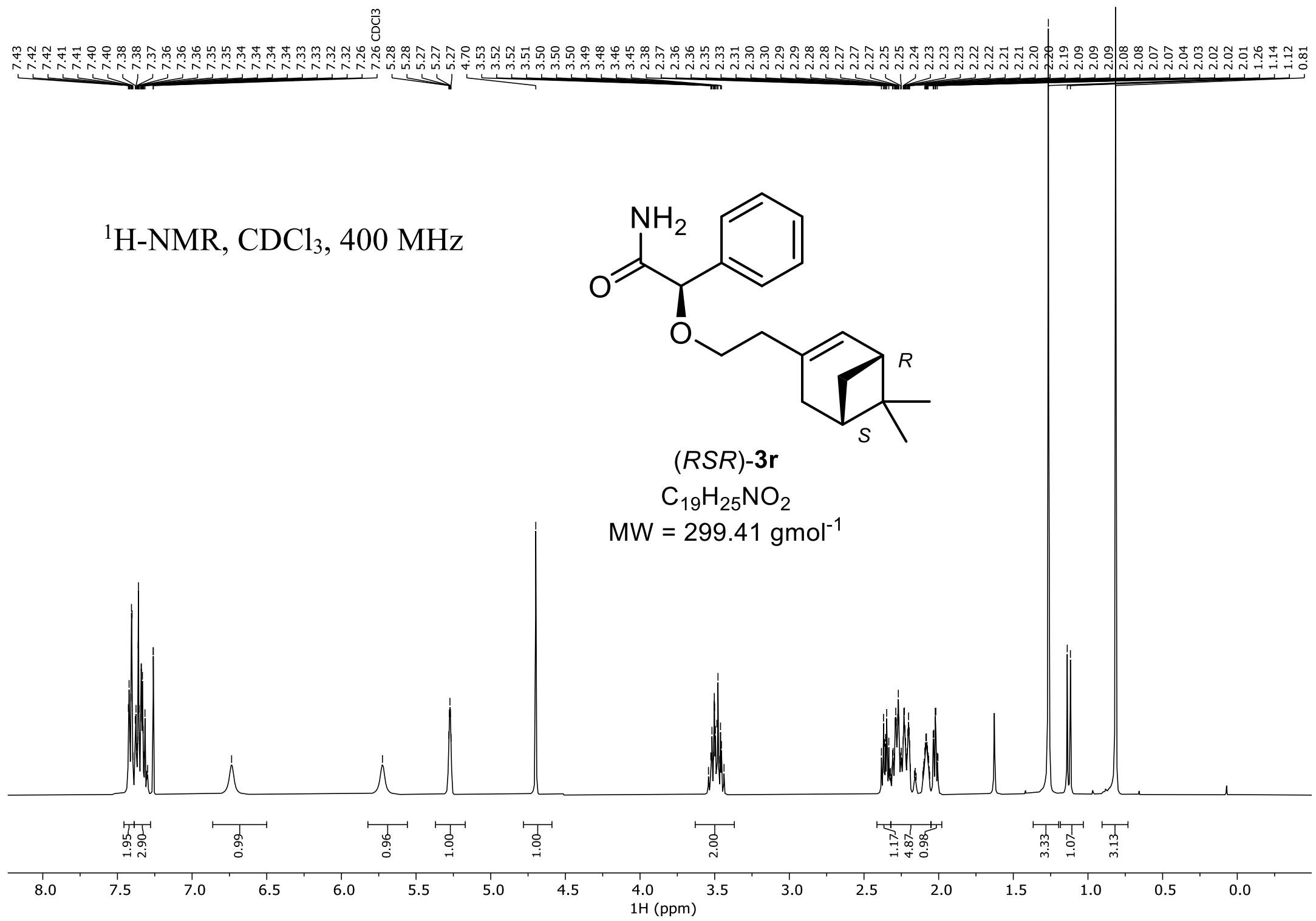

7.43 7.42 7.42 7.41 7.41 7.40 7.38 7.38 7.37 7.36 7.36 7.36 7.35 7.35 7.34 7.34 7.34 7.34 7.33 7.33 7.32 7.32 7.26 CDCl3 5.28 5.28 5.27 5.27 5.27 4.70 3.53 3.52 3.52 3.51 3.50 3.50 3.49 3.48 3.46 3.45 2.38 2.37 2.36 2.36 2.35 2.33 2.31 2.30 2.30 2.29 2.29 2.28 2.28 2.27 2.27 2.27 2.25 2.25 2.24 2.23 2.23 2.23 2.22 2.22 2.22 2.21 2.21 2.20 2.20 2.19 2.09 2.09 2.09 2.09 2.08 2.08 2.07 2.04 2.03 2.02 2.02 2.01 1.26 1.14 1.12 0.81
1H-NMR, CDCl3, 400 MHz
NH2
O
O
R
S
(RSR)-3r
C19H25NO2
MW = 299.41 gmol-1
1.95 2.90 0.99 0.96 1.00 1.00 2.00 1.17 4.87 0.98 3.33 1.07 3.13
1H (ppm)

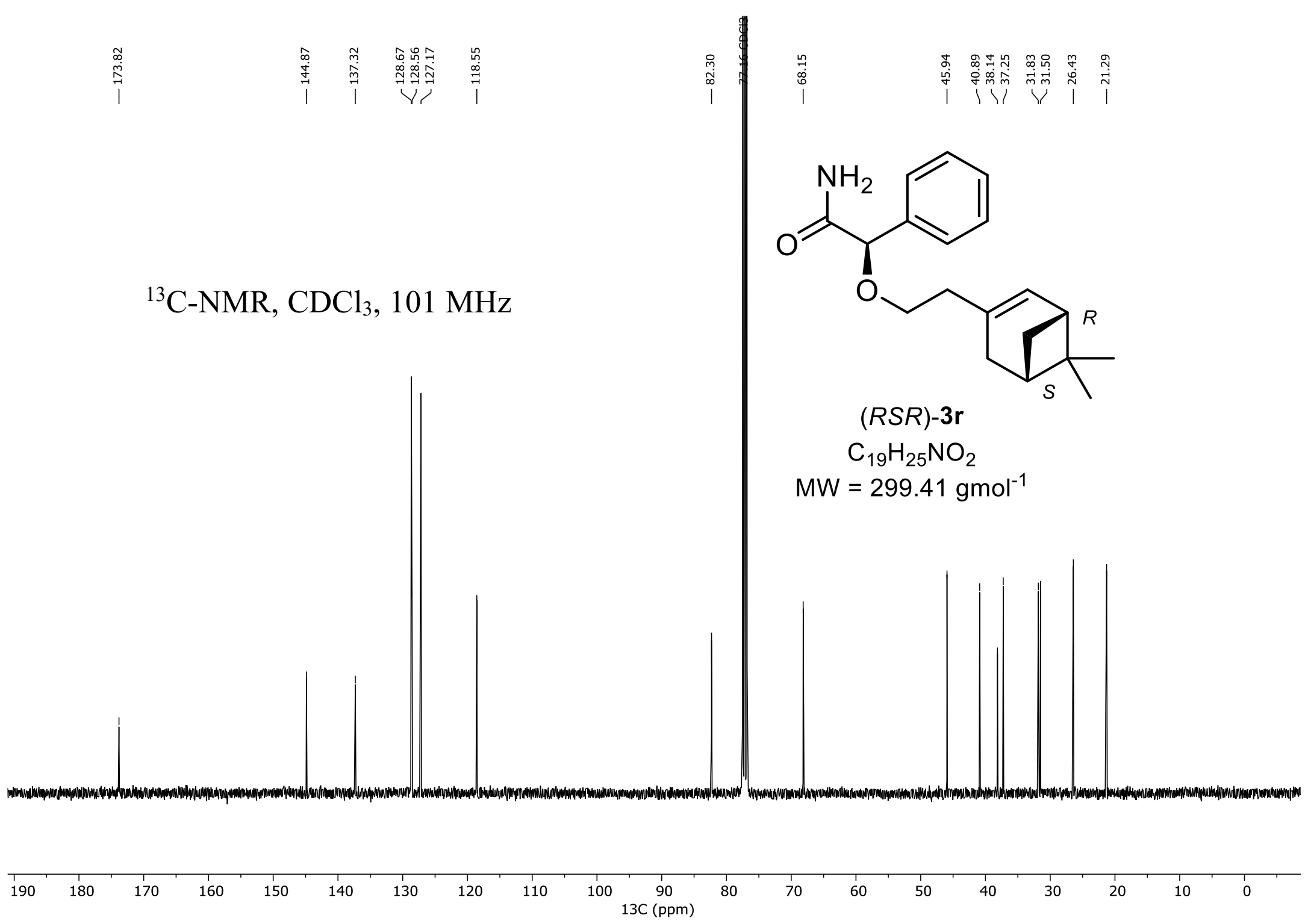

173.82 144.87 137.32 128.67 128.56 127.17 118.55 82.30 77.16 CDCl3 68.15 45.94 40.89 38.14 37.25 31.83 31.50 26.43 21.29
13C-NMR, CDCl3, 101 MHz
NH2
O
O
R
S
(RSR)-3r
C19H25NO2
MW = 299.41 gmol-1
13C (ppm)

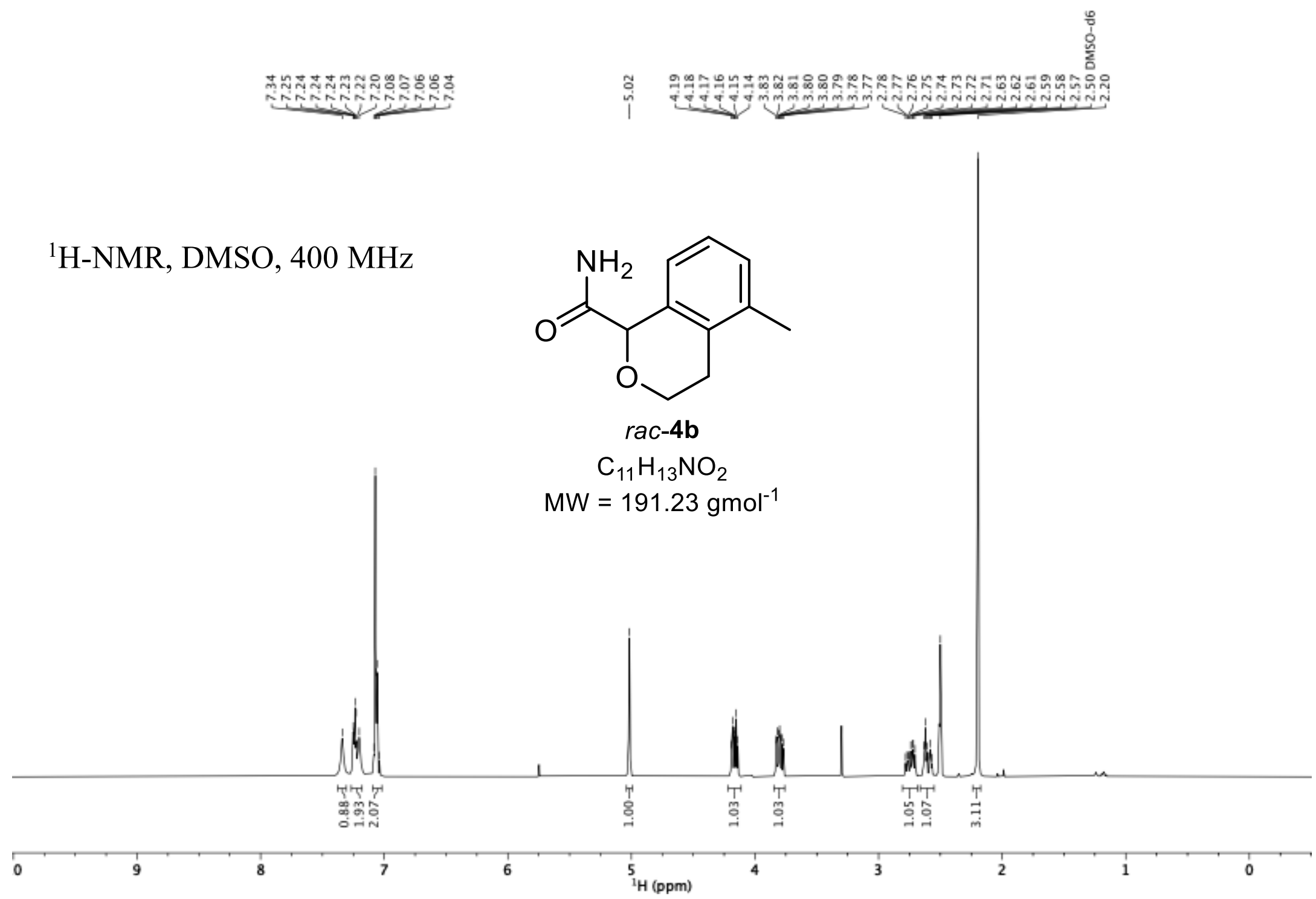

7.34
7.25
7.24
7.24
7.24
7.23
7.22
7.20
7.08
7.07
7.06
7.06
7.04
5.02
4.19
4.18
4.17
4.16
4.15
4.14
3.83
3.82
3.81
3.80
3.80
3.79
3.78
3.77
2.78
2.77
2.76
2.75
2.74
2.73
2.72
2.71
2.63
2.62
2.61
2.59
2.58
2.57
2.50 DMSO-d6
2.20
1H-NMR, DMSO, 400 MHz
NH2
O
O
rac-4b
C11H13NO2
MW = 191.23 gmol-1
0.88
1.93
2.07
1.00
1.03
1.03
1.05
1.07
3.11
1H (ppm)

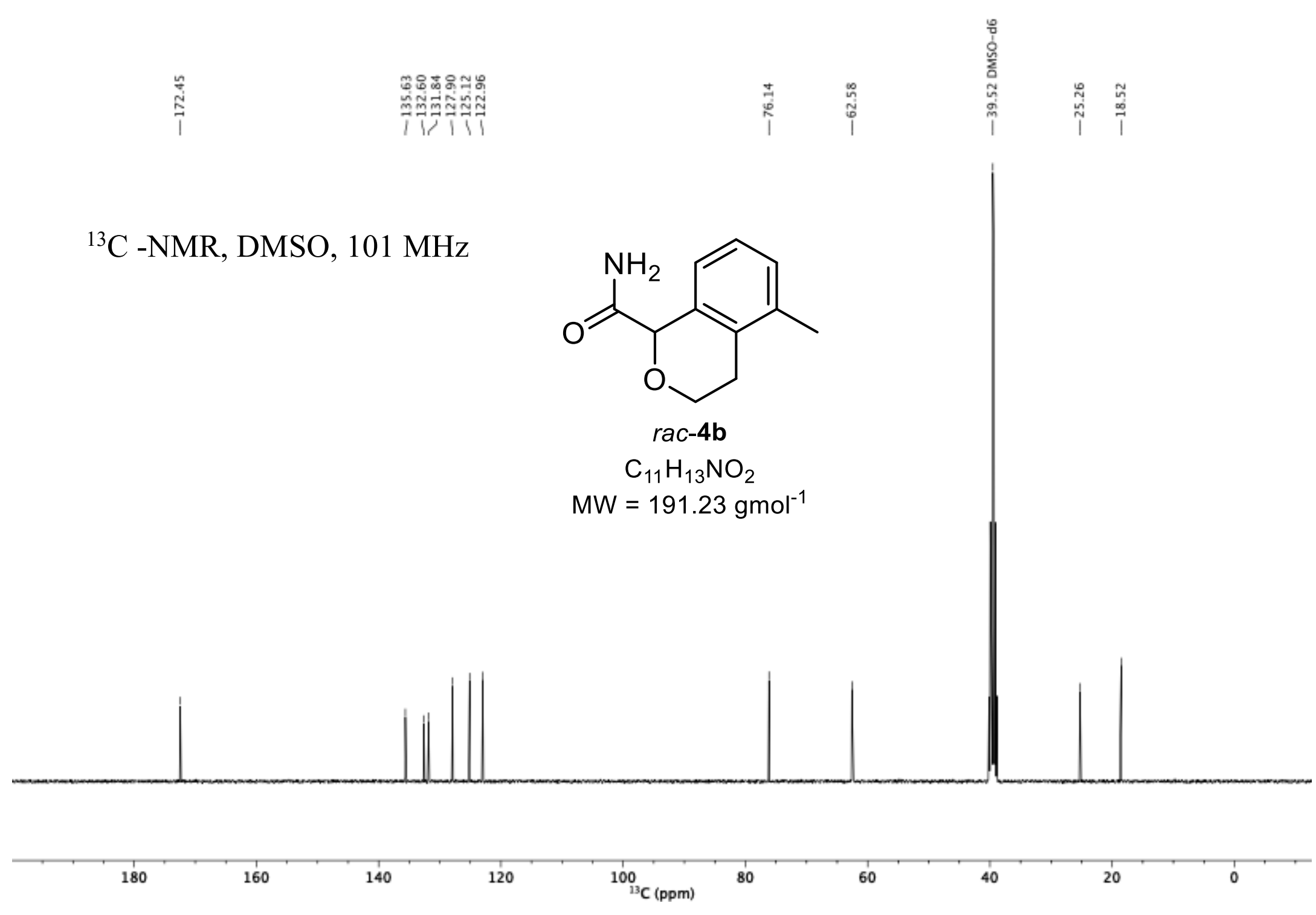

172.45
135.63
132.60
131.84
127.90
125.12
122.96
76.14
62.58
39.52 DMSO-d6
25.26
18.52
13C -NMR, DMSO, 101 MHz
NH2
O
O
rac-4b
C11H13NO2
MW = 191.23 gmol-1
13C (ppm)

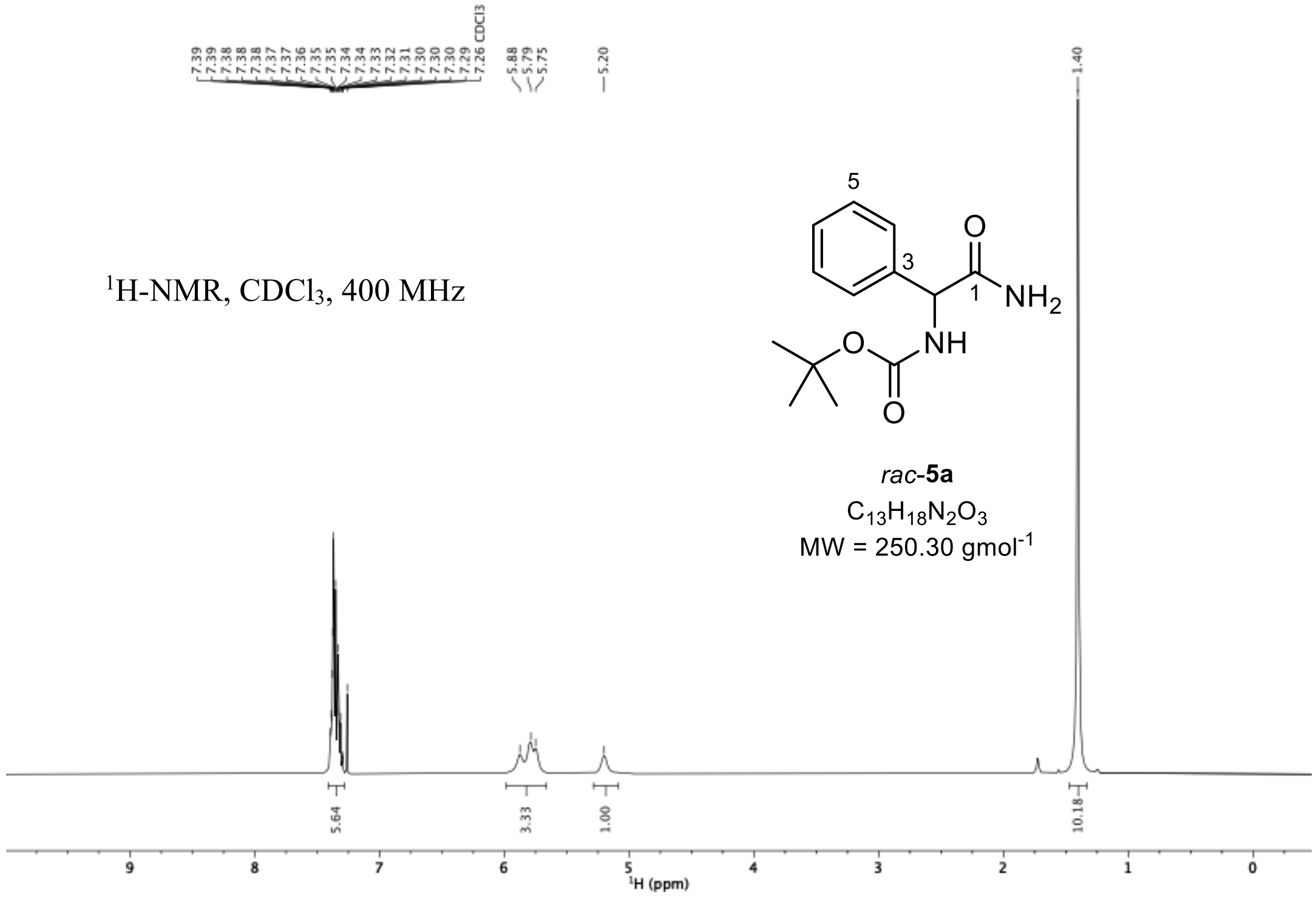

^{1}H-NMR, CDCl$_3$, 400 MHz
rac-5a
$C_{13}H_{18}N_2O_3$
MW = 250.30 gmol^{-1}

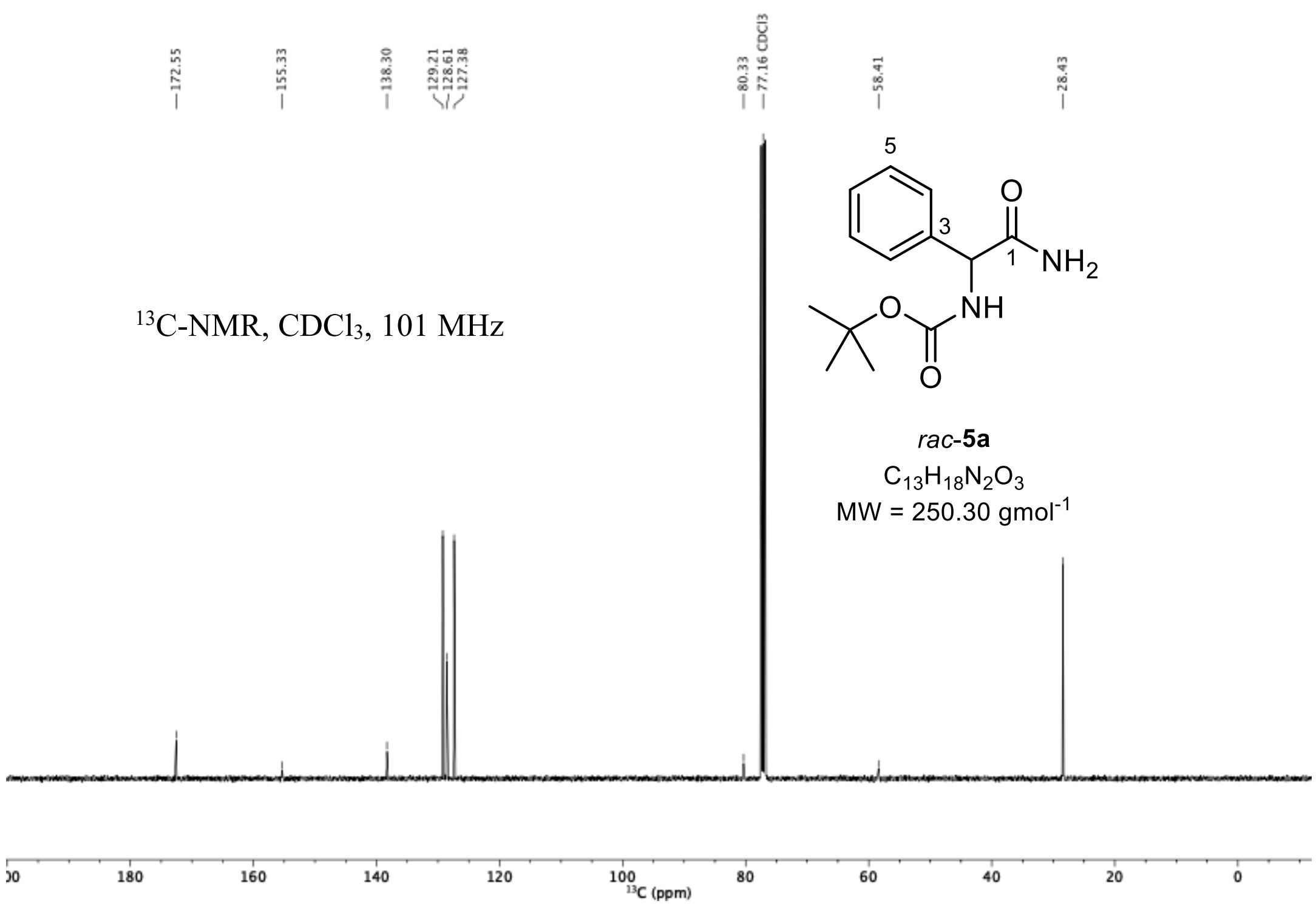

^{13}C-NMR, CDCl$_3$, 101 MHz
rac-5a
$C_{13}H_{18}N_2O_3$
MW = 250.30 gmol^{-1}

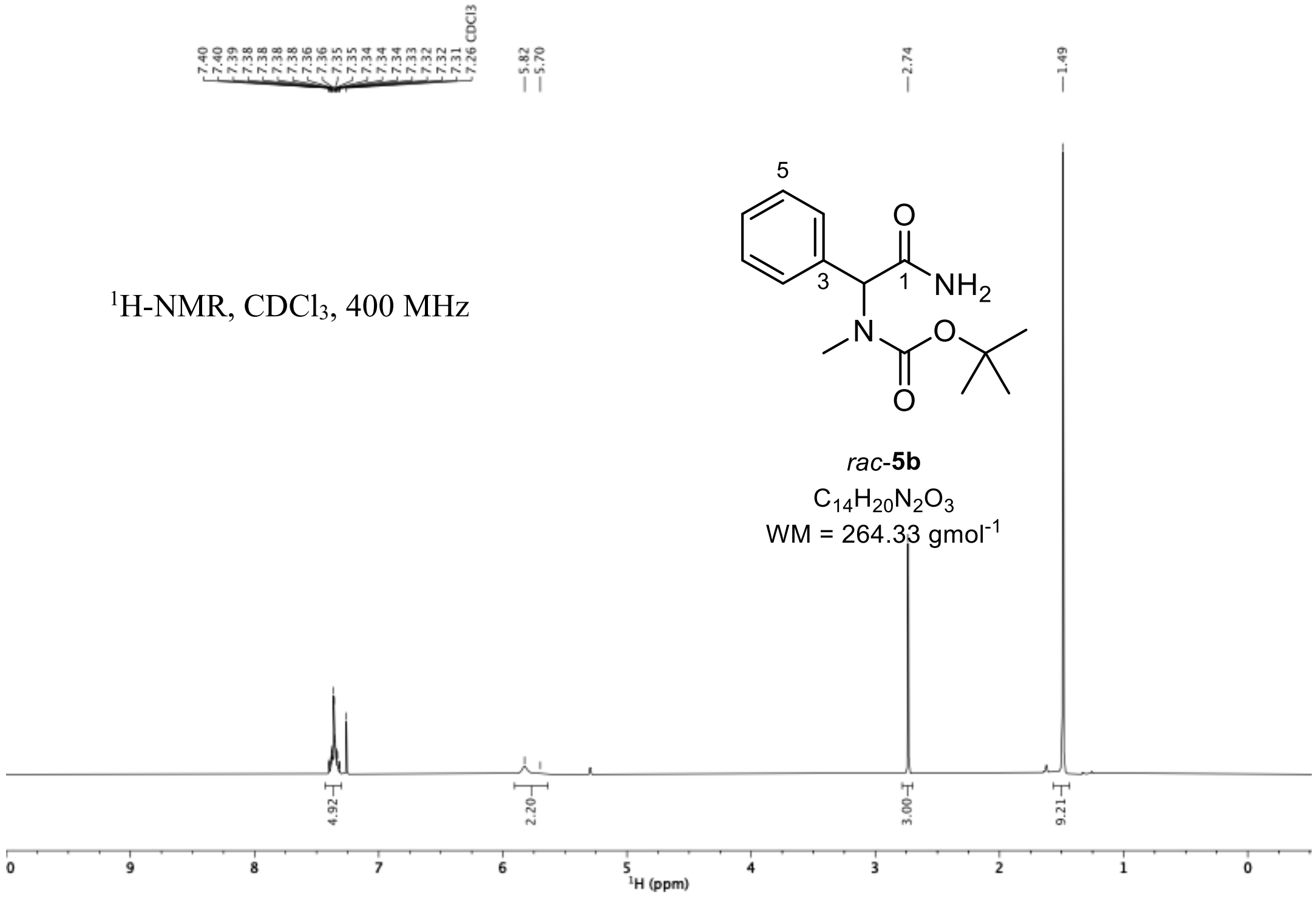

7.40
7.40
7.39
7.38
7.38
7.38
7.38
7.36
7.35
7.35
7.34
7.34
7.34
7.34
7.33
7.33
7.32
7.32
7.31
7.26 CDCl3
5.82
5.70
2.74
1.49
^{1}H-NMR, CDCl$_3$, 400 MHz
5
3
1
O
NH$_2$
N
O
O
rac-**5b**
$C_{14}H_{20}N_2O_3$
WM = 264.33 gmol^{-1}
4.92
2.20
3.00
9.21
^{1}H (ppm)

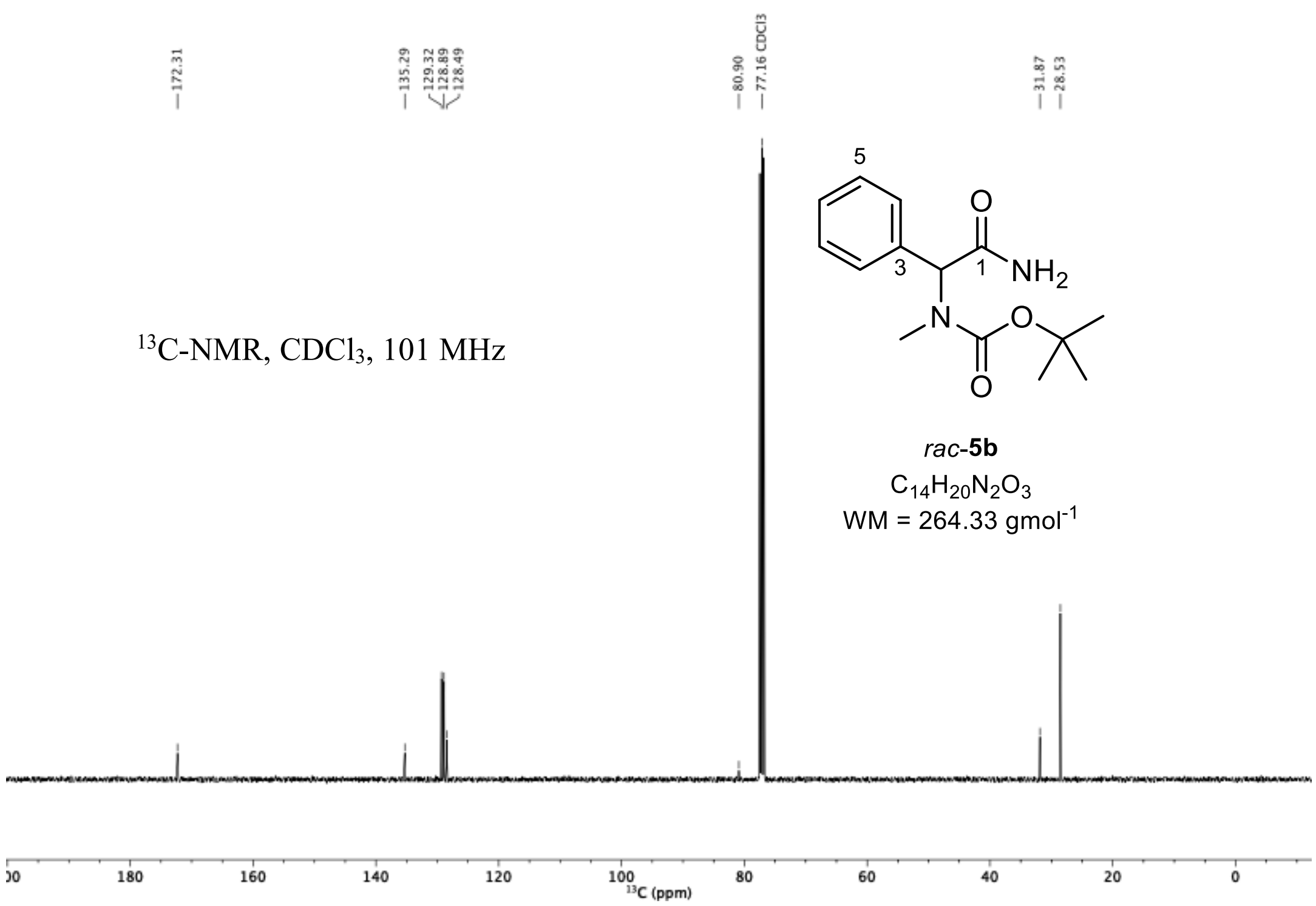

172.31
135.29
129.32
128.89
128.49
80.90
77.16 CDCl3
31.87
28.53
^{13}C-NMR, CDCl$_3$, 101 MHz
5
3
1
O
NH$_2$
N
O
O
rac-**5b**
$C_{14}H_{20}N_2O_3$
WM = 264.33 gmol^{-1}
^{13}C (ppm)

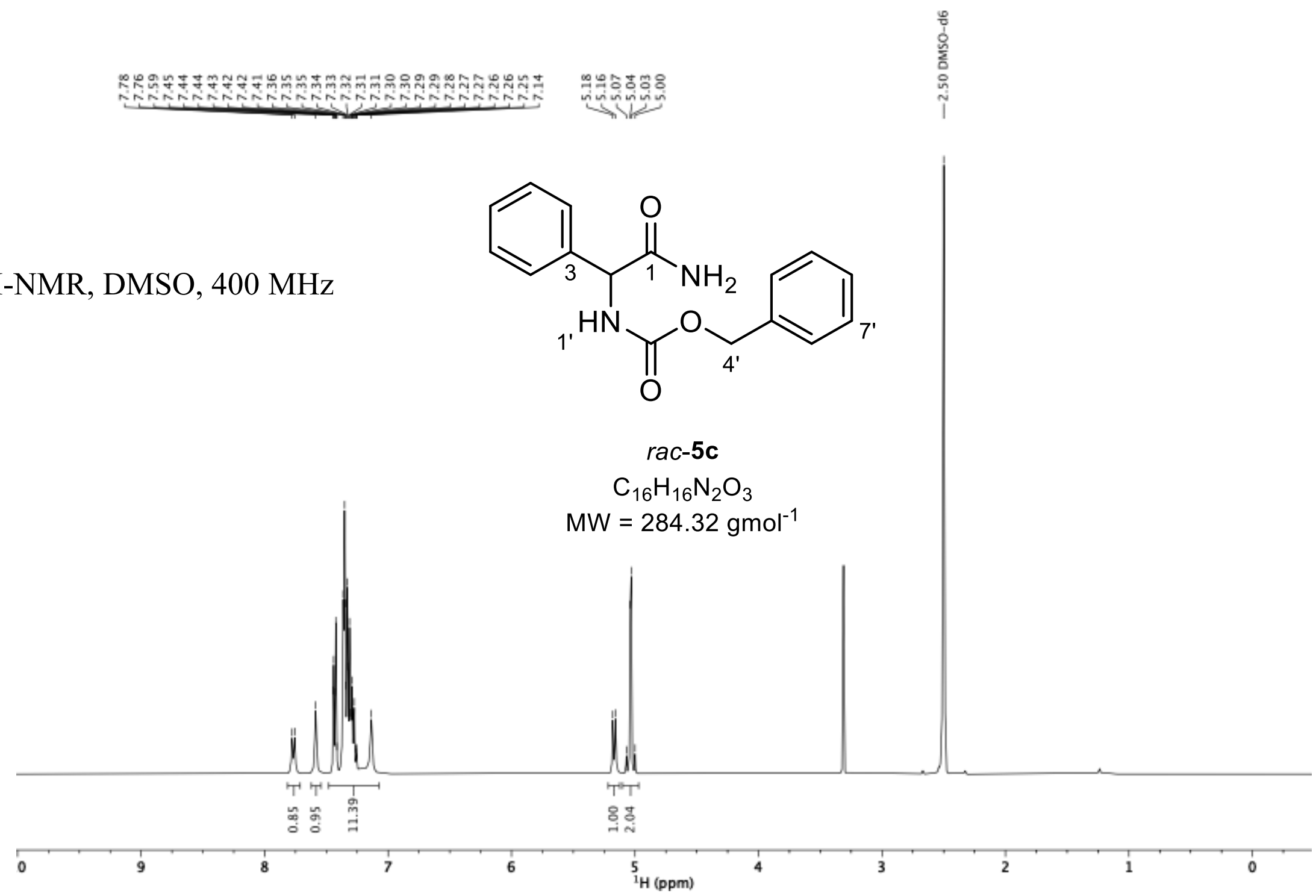
7.78
7.76
7.59
7.45
7.44
7.44
7.43
7.42
7.42
7.41
7.36
7.35
7.35
7.34
7.33
7.32
7.31
7.31
7.30
7.30
7.29
7.29
7.28
7.28
7.27
7.27
7.26
7.26
7.25
7.14
5.18
5.16
5.07
5.04
5.03
5.00
2.50 DMSO-d6
^{1}H-NMR, DMSO, 400 MHz
O
NH$_2$
3
1
HN
1'
O
4'
7'
O
rac-**5c**
$C_{16}H_{16}N_2O_3$
MW = 284.32 gmol^{-1}
0.85
0.95
11.39
1.00
2.04
^{1}H (ppm)

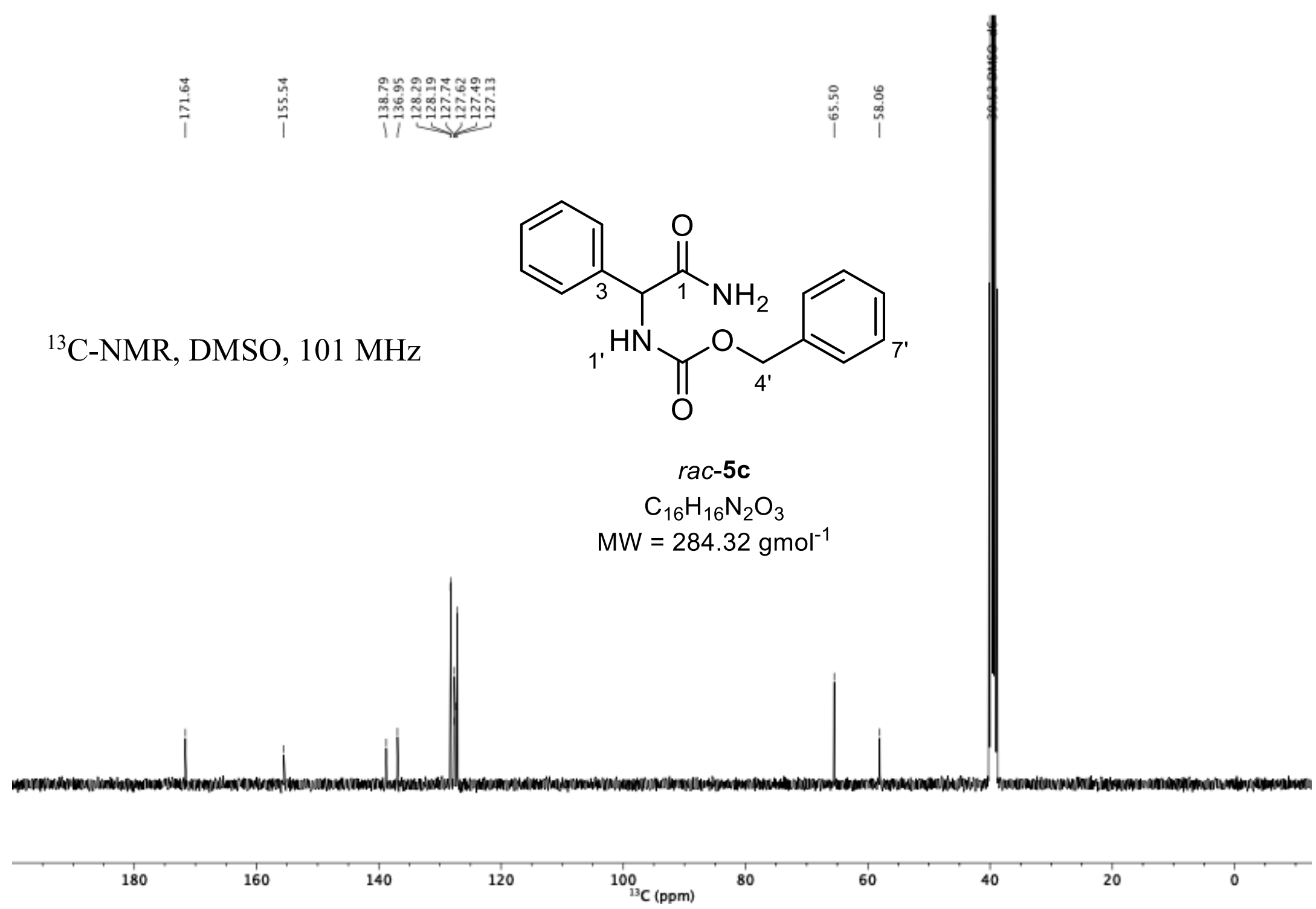
171.64
155.54
138.79
136.95
128.29
128.19
127.74
127.62
127.49
127.13
65.50
58.06
^{13}C-NMR, DMSO, 101 MHz
O
NH$_2$
3
1
HN
1'
O
4'
7'
O
rac-**5c**
$C_{16}H_{16}N_2O_3$
MW = 284.32 gmol^{-1}
^{13}C (ppm)

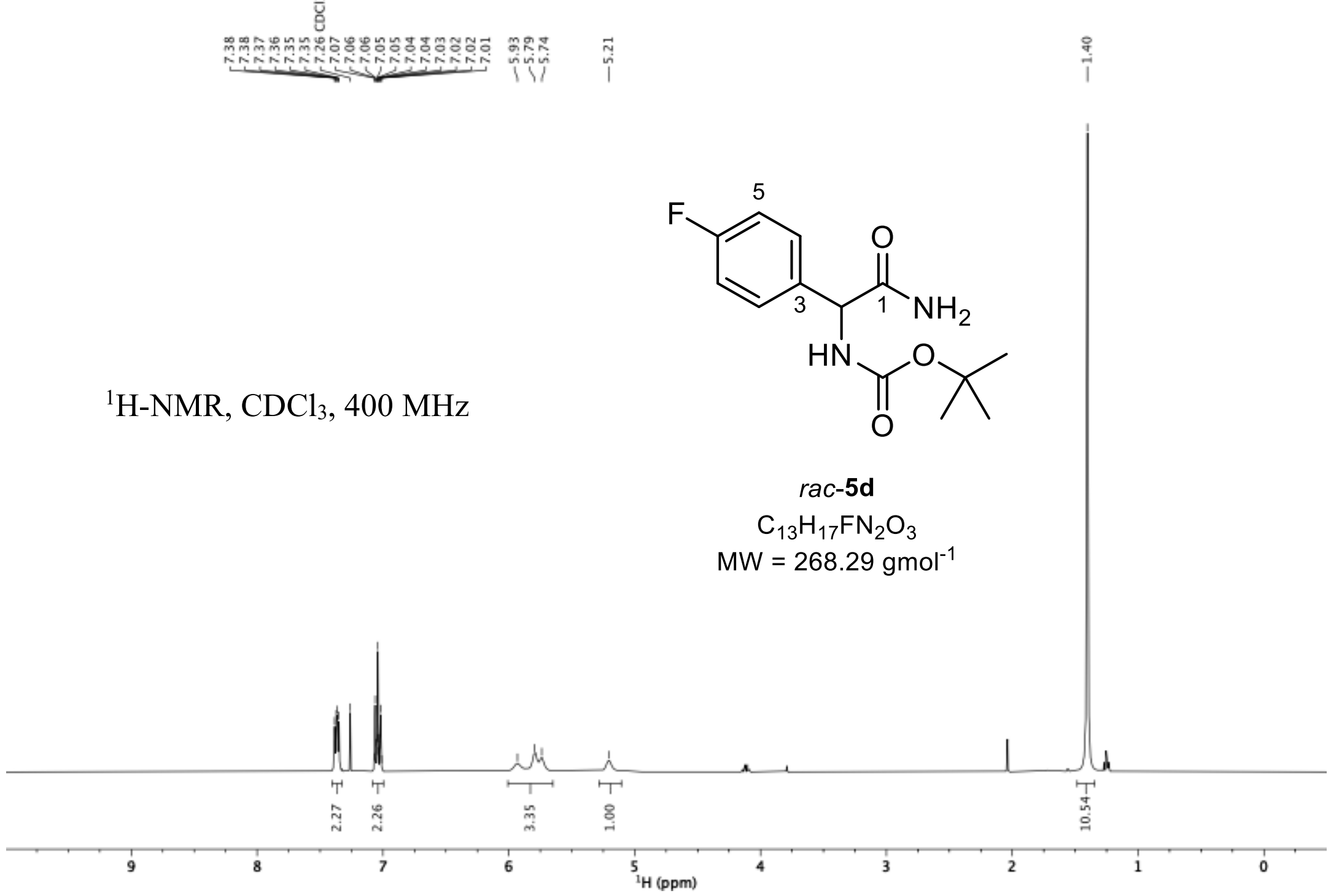

7.38 7.38 7.37 7.36 7.35 7.35 7.26 CDCl3 7.07 7.06 7.06 7.05 7.05 7.04 7.04 7.03 7.02 7.02 7.01
5.93 5.79 5.74
5.21
1.40
F
5
3
1
O
NH2
HN
O
O
1H-NMR, CDCl3, 400 MHz
rac-5d
C13H17FN2O3
MW = 268.29 gmol-1
2.27
2.26
3.35
1.00
10.54
1H (ppm)

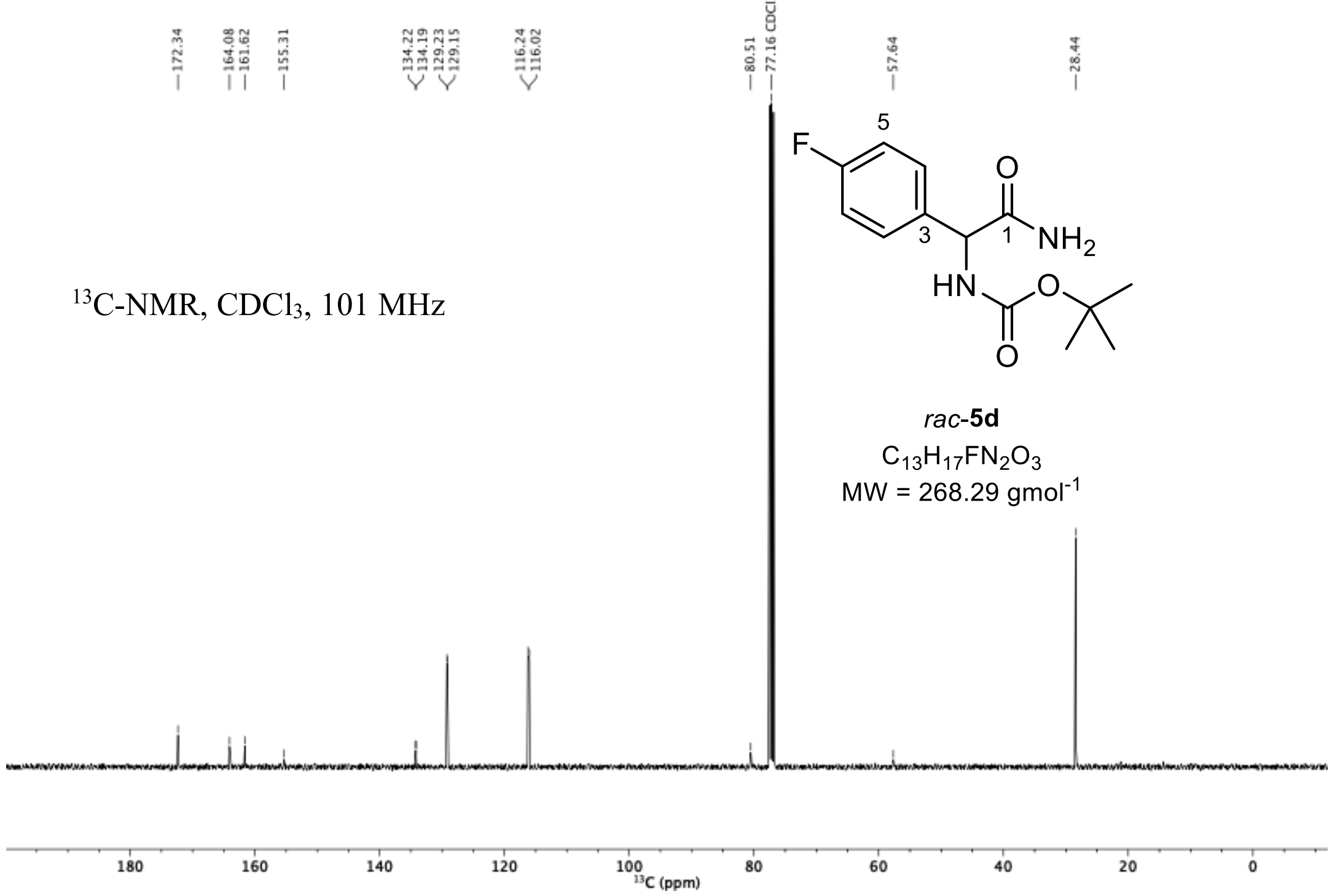

172.34
164.08 161.62
155.31
134.22 134.19 129.23 129.15
116.24 116.02
80.51 77.16 CDCl3
57.64
28.44
F
5
3
1
O
NH2
HN
O
O
13C-NMR, CDCl3, 101 MHz
rac-5d
C13H17FN2O3
MW = 268.29 gmol-1
13C (ppm)

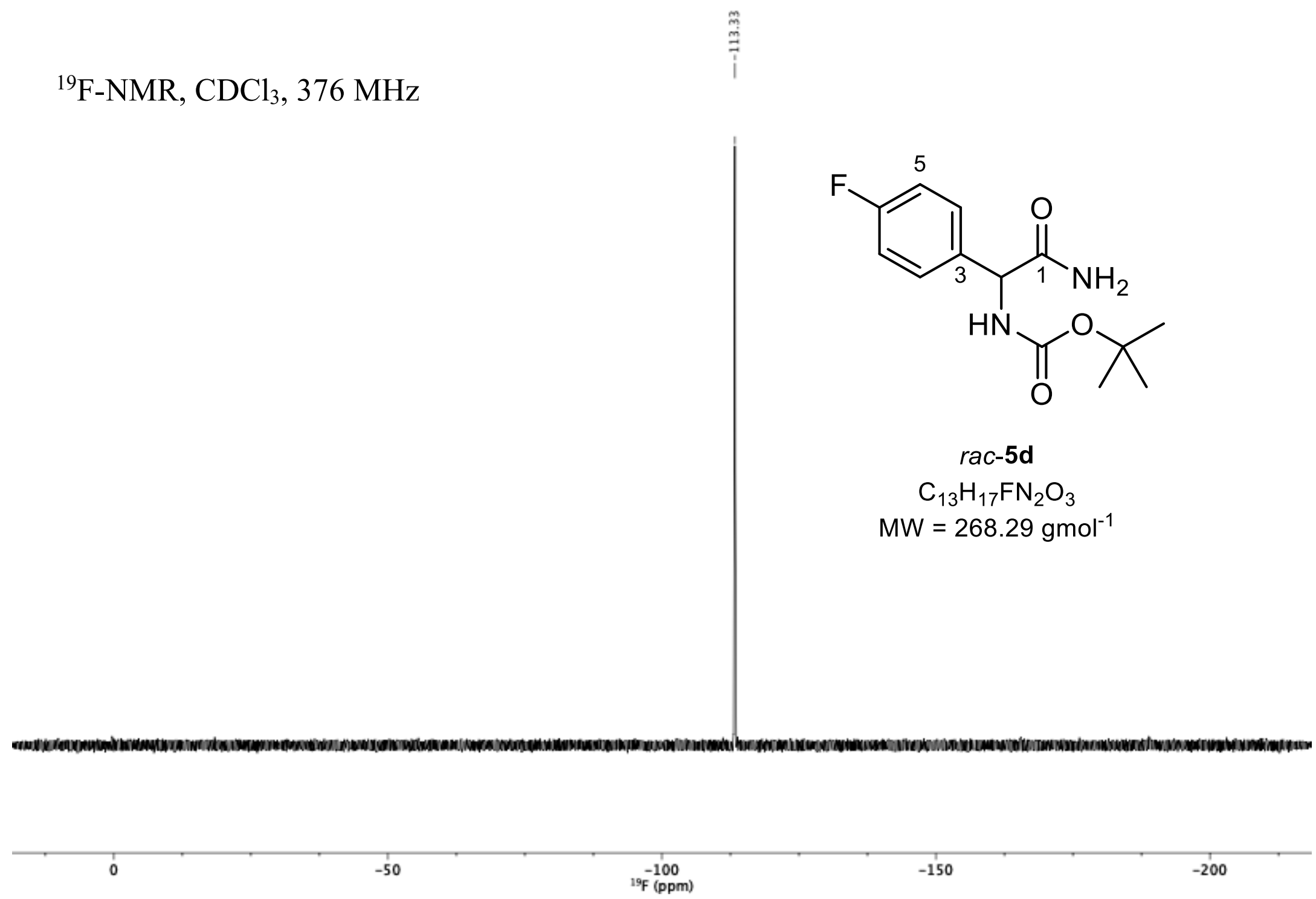

^{19}F-NMR, $CDCl_3$, 376 MHz
-113.33
F
5
3
1
O
NH_2
HN
O
O
rac-**5d**
$C_{13}H_{17}FN_2O_3$
MW = 268.29 $gmol^{-1}$
0
-50
-100
-150
-200
^{19}F (ppm)

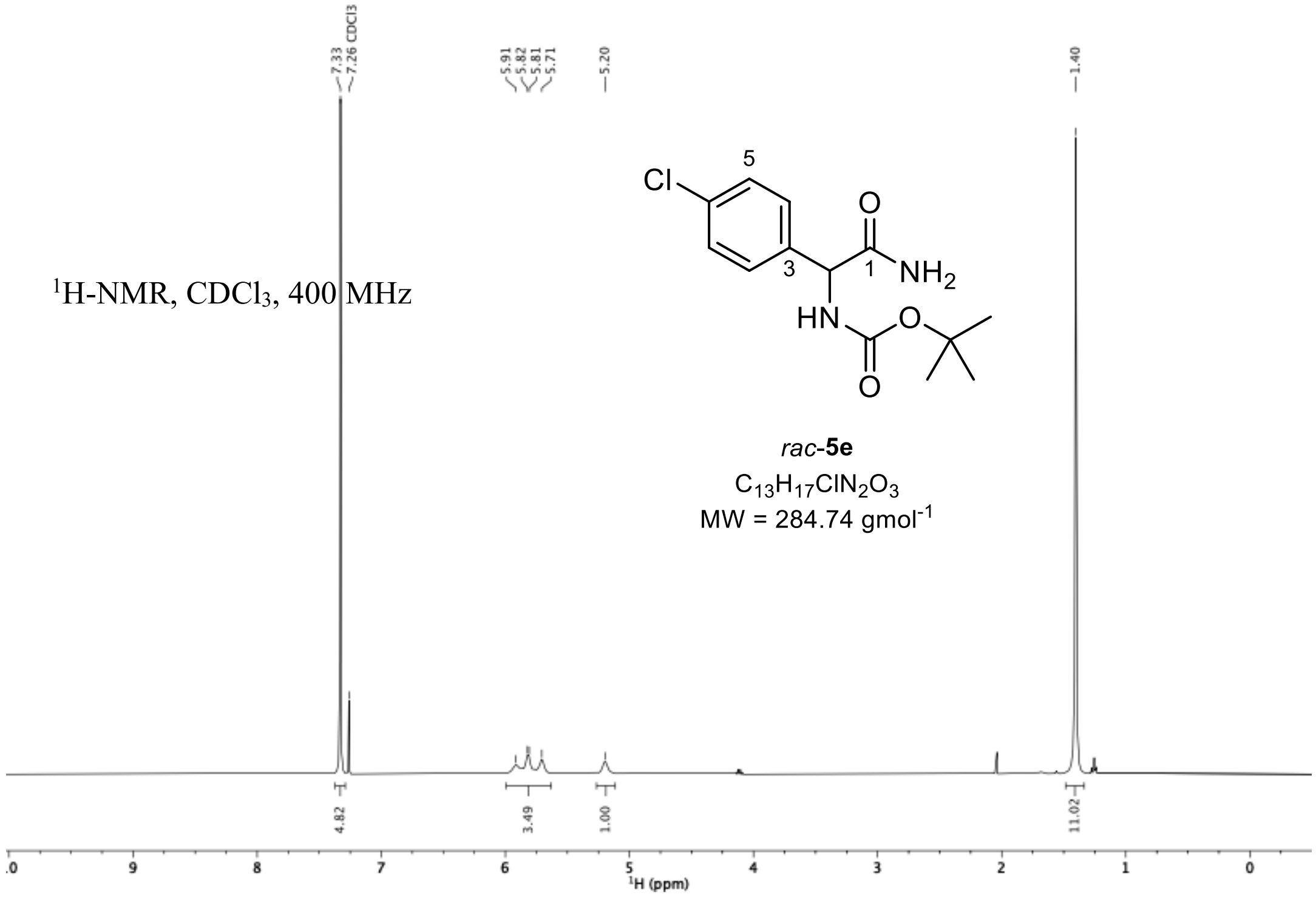

7.33
7.26 CDCl3
5.91
5.82
5.81
5.71
5.20
1.40
^{1}H-NMR, $CDCl_3$, 400 MHz
Cl
5
3
1
O
NH_2
HN
O
O
rac-**5e**
$C_{13}H_{17}ClN_2O_3$
MW = 284.74 $gmol^{-1}$
4.82
3.49
1.00
11.02
^{1}H (ppm)

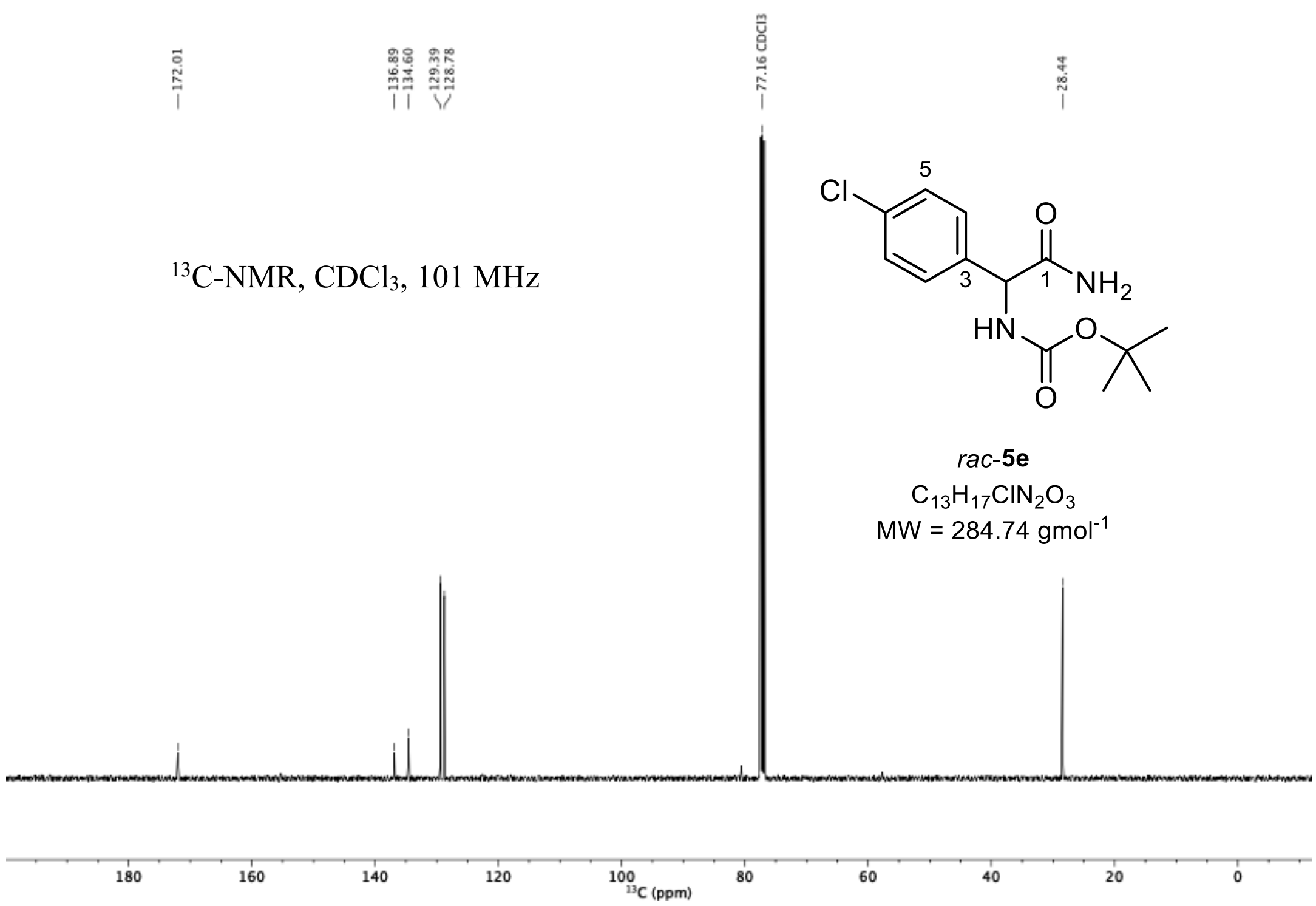

172.01
136.89
134.60
129.39
128.78
77.16 CDCl3
28.44
^{13}C-NMR, $CDCl_3$, 101 MHz
Cl
5
3
1
O
NH_2
HN
O
O
rac-**5e**
$C_{13}H_{17}ClN_2O_3$
MW = 284.74 $gmol^{-1}$
^{13}C (ppm)

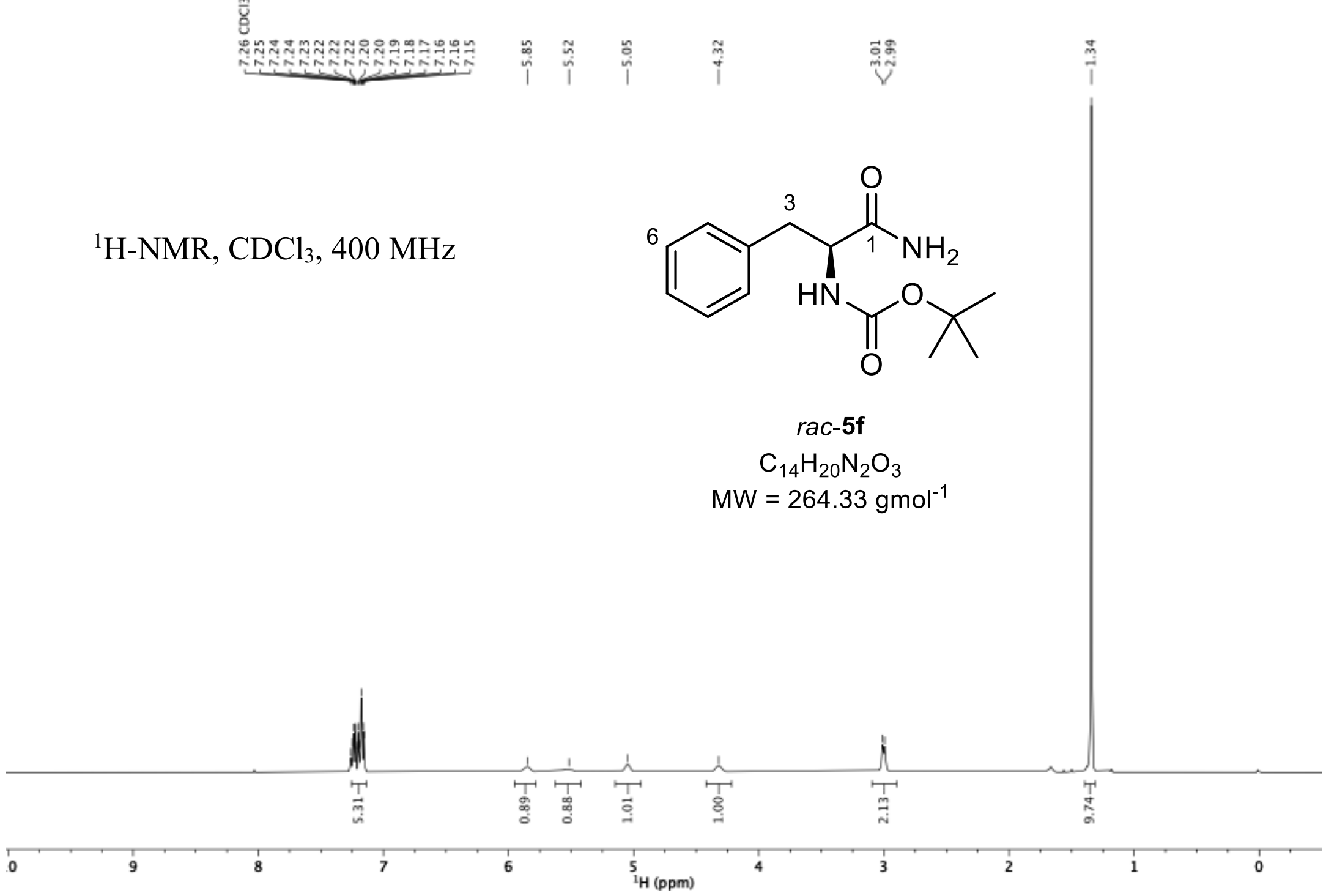
7.26 CDCl3
7.25
7.24
7.24
7.23
7.22
7.22
7.22
7.20
7.20
7.19
7.18
7.17
7.16
7.16
7.15
5.85
5.52
5.05
4.32
3.01
2.99
1.34
1H-NMR, CDCl3, 400 MHz
3
6
1
O
NH2
HN
O
O
rac-5f
C14H20N2O3
MW = 264.33 gmol-1
5.31
0.89
0.88
1.01
1.00
2.13
9.74
1H (ppm)

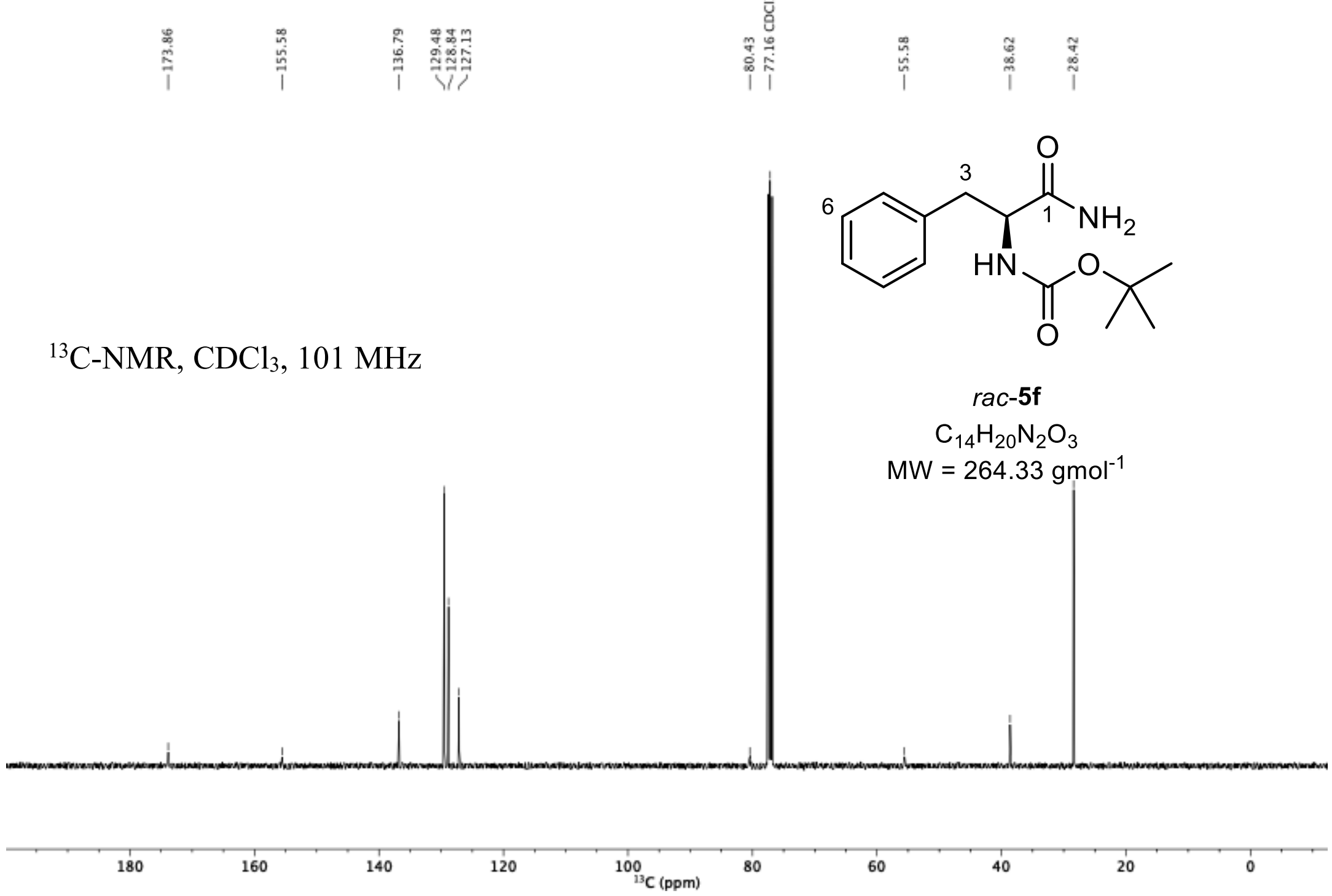
173.86
155.58
136.79
129.48
128.84
127.13
80.43
77.16 CDCl3
55.58
38.62
28.42
13C-NMR, CDCl3, 101 MHz
3
6
1
O
NH2
HN
O
O
rac-5f
C14H20N2O3
MW = 264.33 gmol-1
13C (ppm)

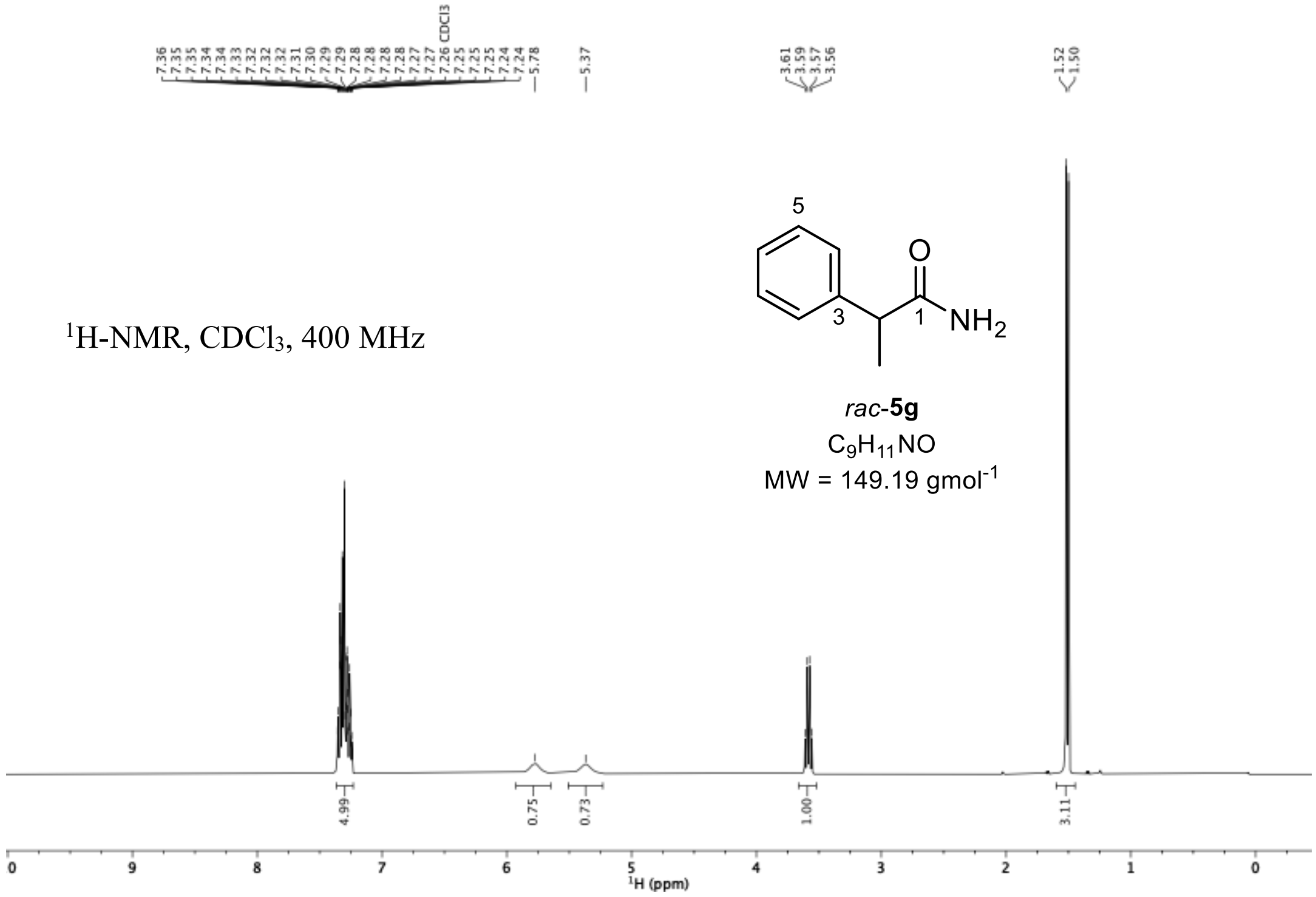

7.36 7.35 7.35 7.34 7.34 7.33 7.32 7.32 7.32 7.31 7.30 7.29 7.29 7.28 7.28 7.28 7.28 7.27 7.27 7.26 CDCl3 7.25 7.25 7.25 7.24 7.24
5.78
5.37
3.61 3.59 3.57 3.56
1.52 1.50
¹H-NMR, CDCl3, 400 MHz
5
3
1
O
NH2
rac-5g
C9H11NO
MW = 149.19 gmol-1
4.99
0.75
0.73
1.00
3.11
1H (ppm)

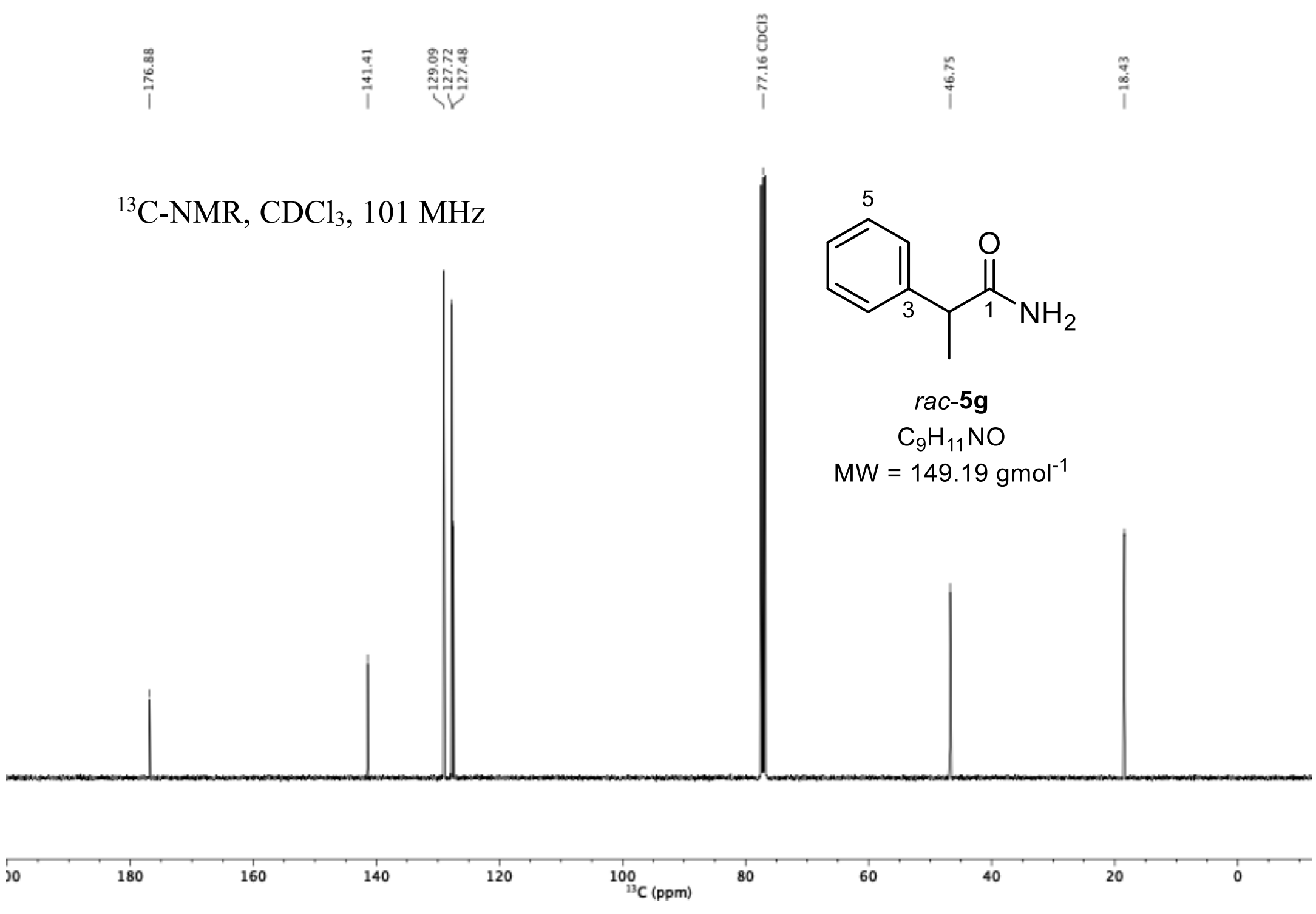

176.88
141.41
129.09 127.72 127.48
77.16 CDCl3
46.75
18.43
¹³C-NMR, CDCl3, 101 MHz
5
3
1
O
NH2
rac-5g
C9H11NO
MW = 149.19 gmol-1
13C (ppm)

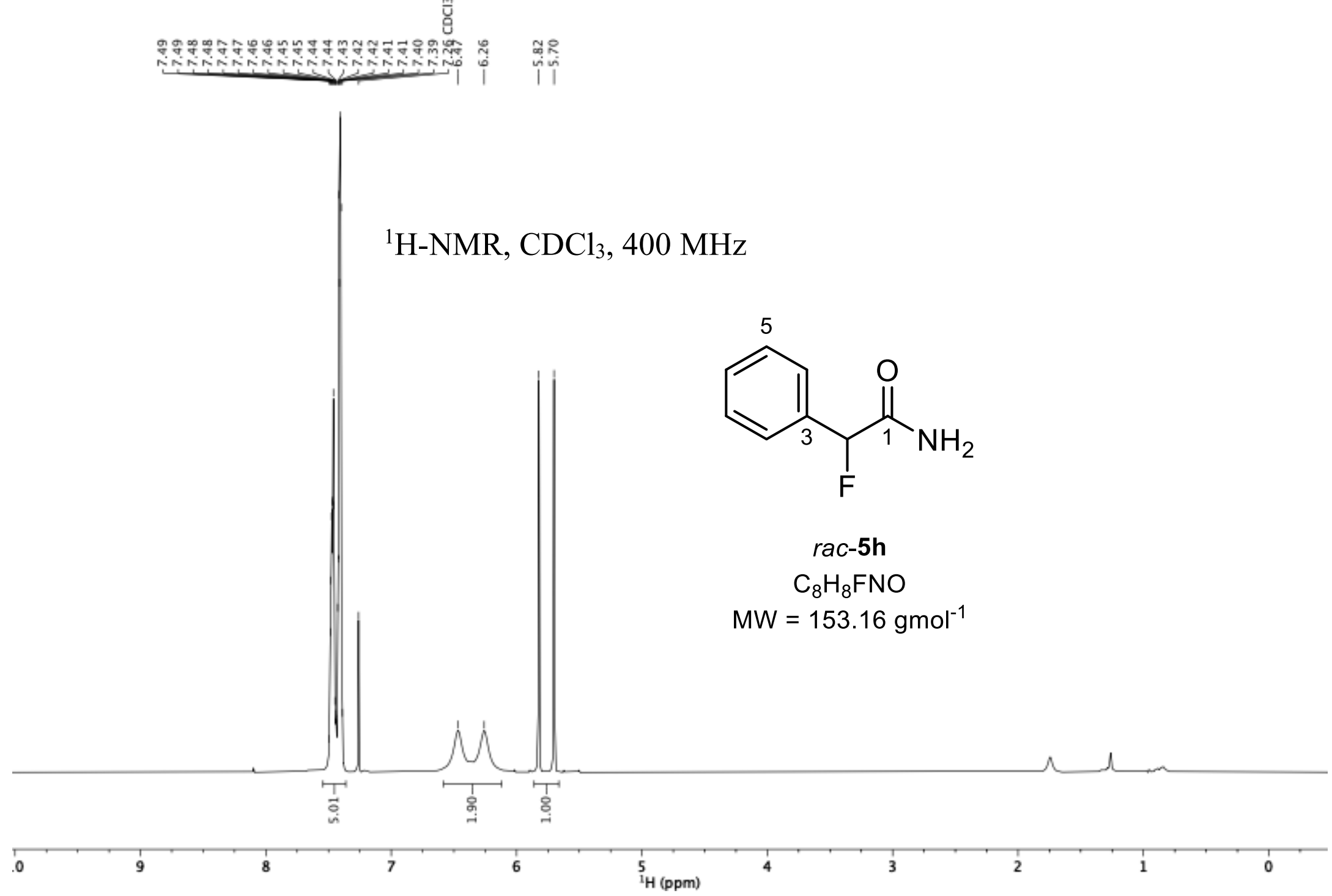

7.49
7.49
7.48
7.47
7.47
7.46
7.46
7.45
7.45
7.44
7.44
7.43
7.42
7.42
7.41
7.41
7.40
7.39
7.26 CDCl3
6.47
6.26
5.82
5.70
1H-NMR, CDCl3, 400 MHz
5
O
3
1
NH2
F
rac-5h
C8H8FNO
MW = 153.16 gmol-1
5.01
1.90
1.00
1H (ppm)

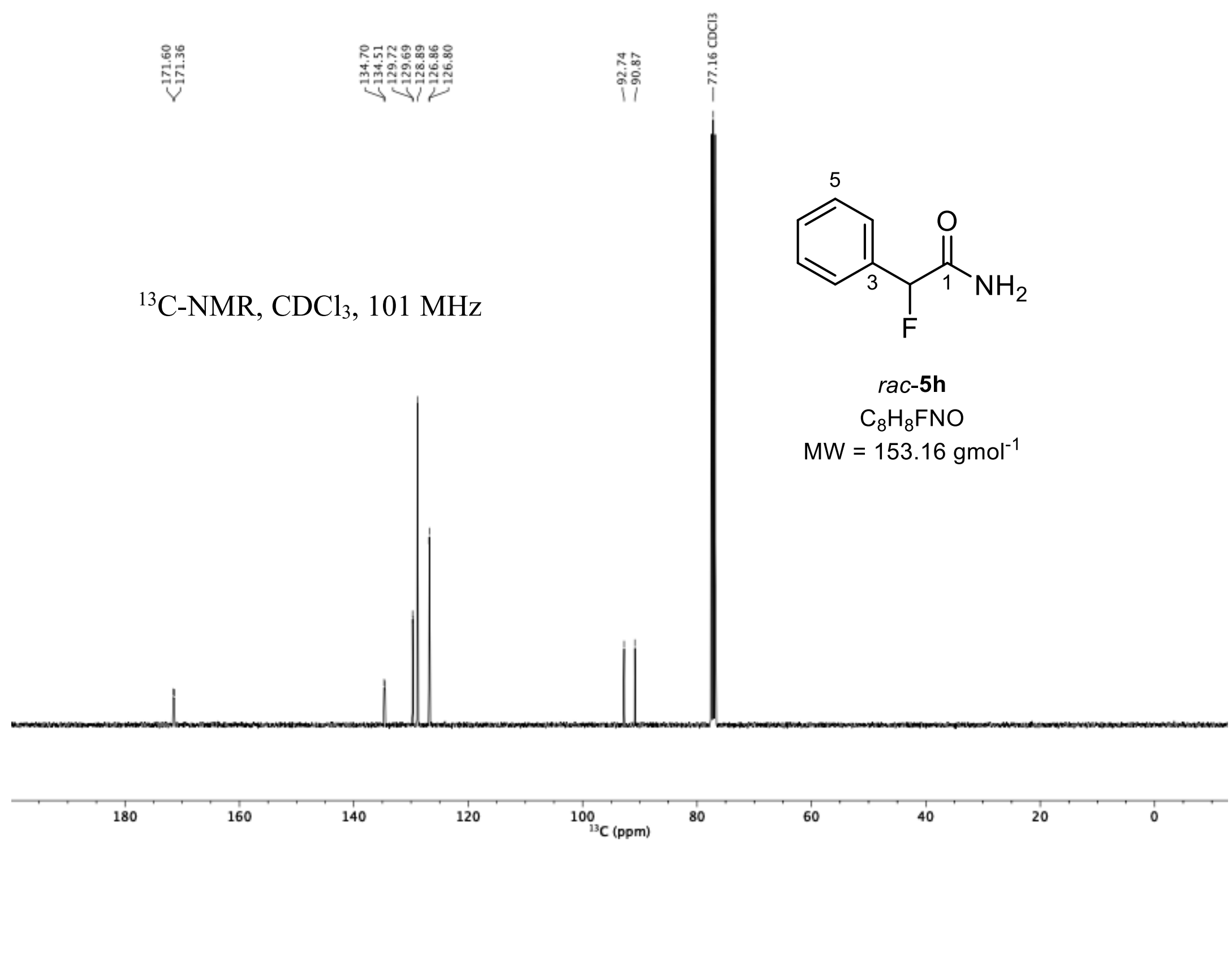

171.60
171.36
134.70
134.51
129.72
129.69
128.89
126.86
126.80
92.74
90.87
77.16 CDCl3
13C-NMR, CDCl3, 101 MHz
5
O
3
1
NH2
F
rac-5h
C8H8FNO
MW = 153.16 gmol-1
13C (ppm)

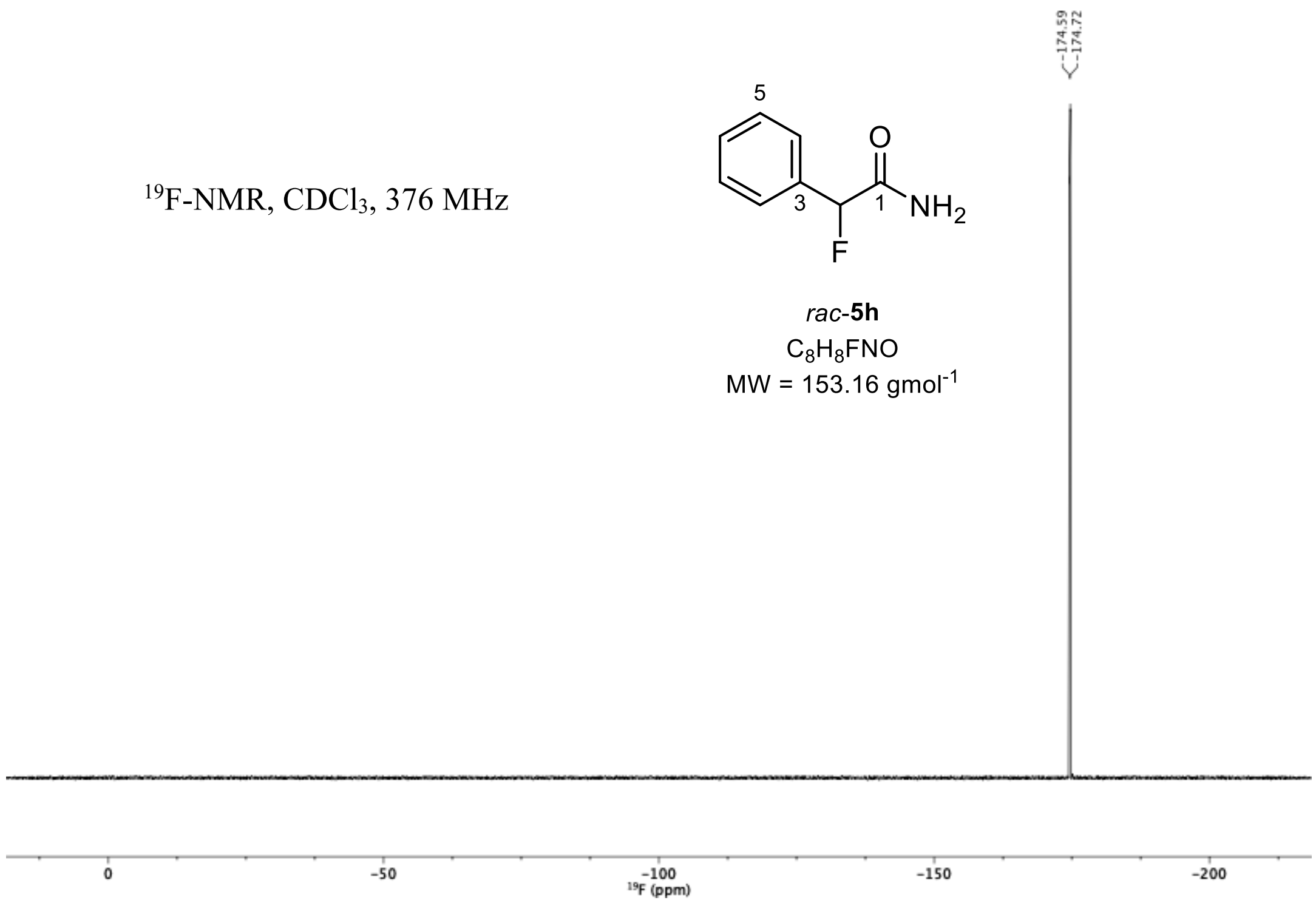

^{19}F-NMR, $CDCl_3$, 376 MHz
rac-**5h**
C_8H_8FNO
MW = 153.16 $gmol^{-1}$
-174.59
-174.72
0
-50
-100
-150
-200
^{19}F (ppm)

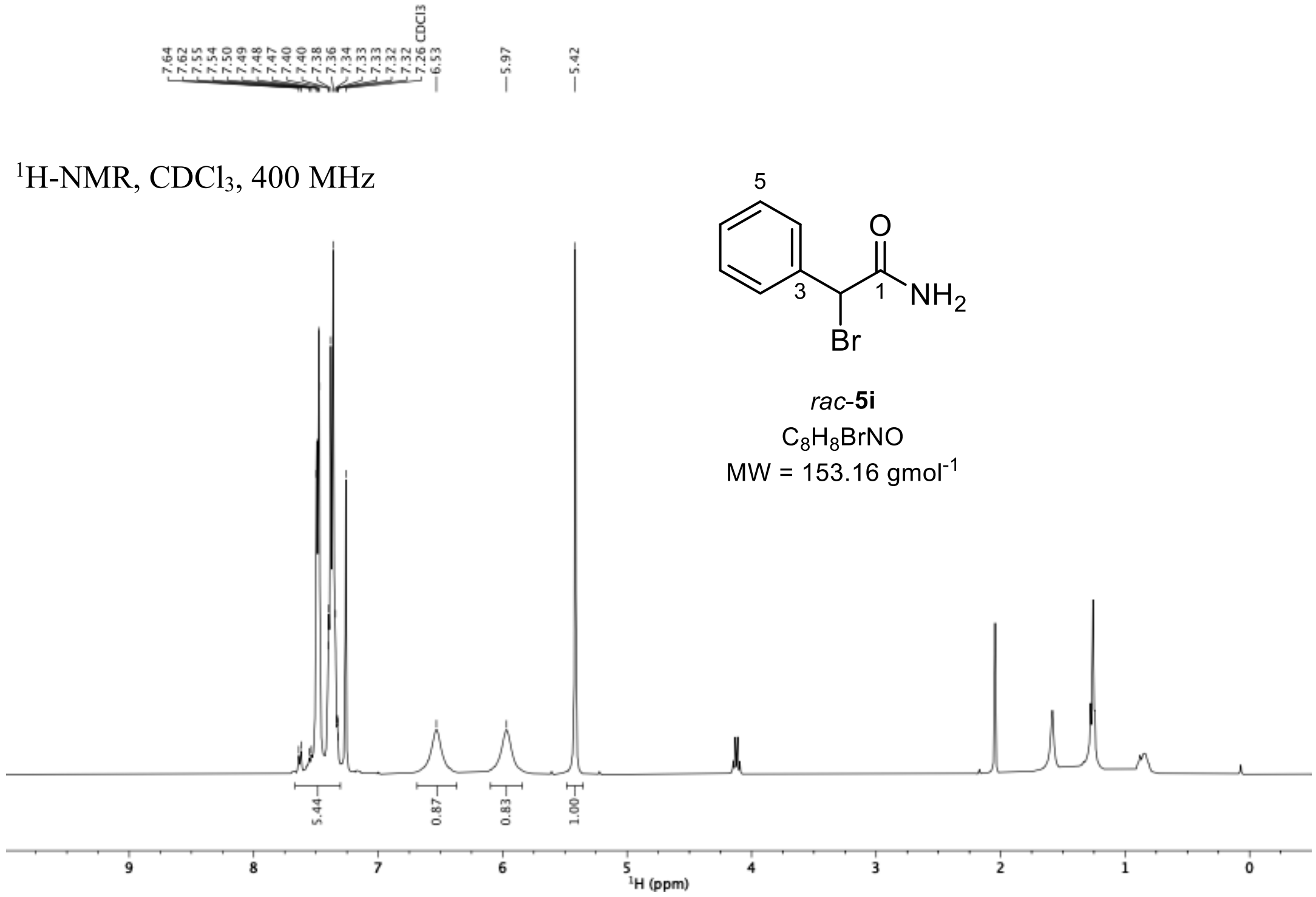
7.64 7.62 7.55 7.54 7.50 7.49 7.48 7.47 7.40 7.40 7.38 7.36 7.34 7.33 7.33 7.33 7.32 7.32 7.26 CDCl3 6.53 5.97 5.42
^{1}H-NMR, $CDCl_3$, 400 MHz
5
3
1
O
NH_2
Br
rac-**5i**
C_8H_8BrNO
MW = 153.16 gmol^{-1}
5.44
0.87
0.83
1.00
^{1}H (ppm)

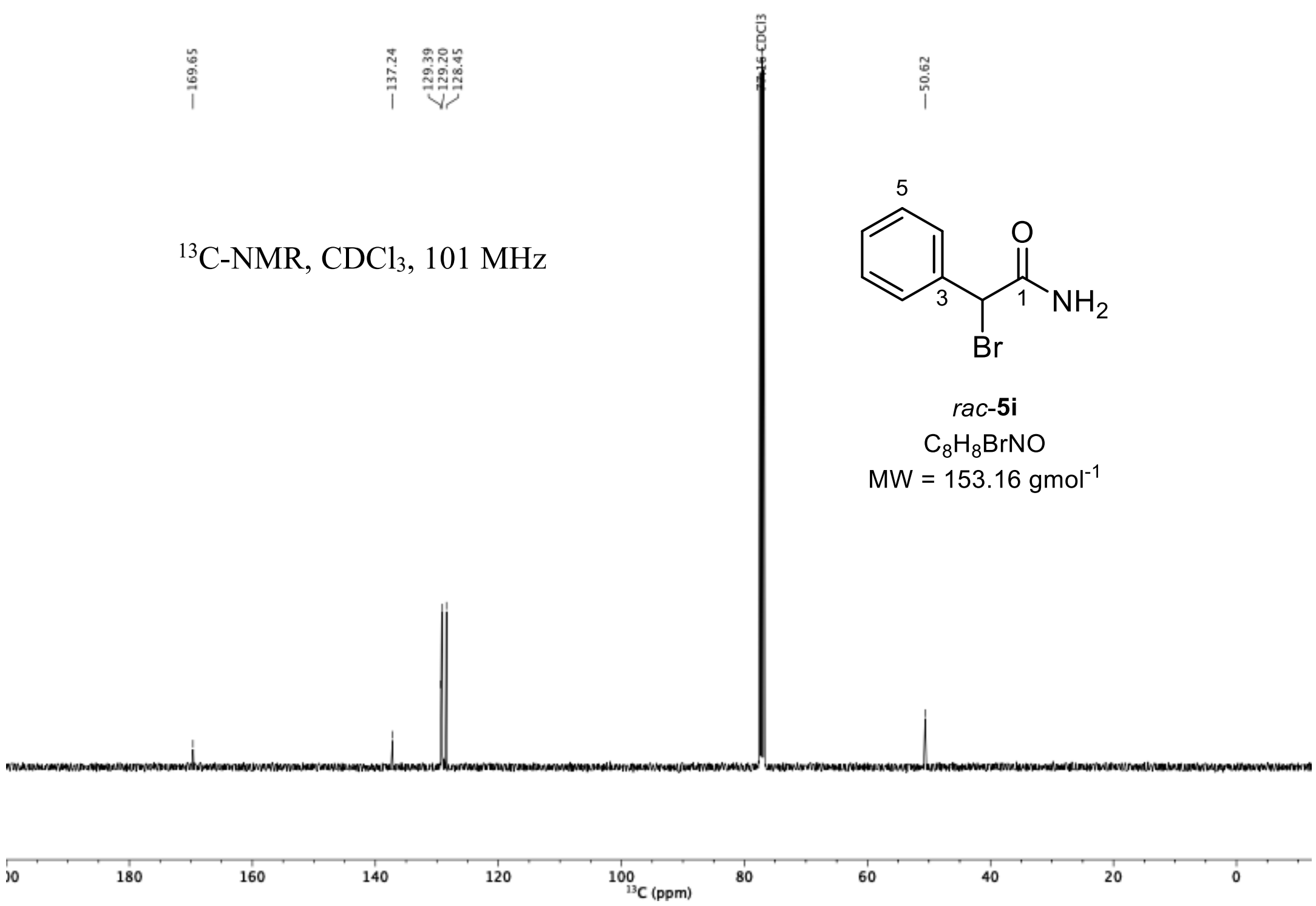
169.65 137.24 129.39 129.20 128.45 77.16 CDCl3 50.62
^{13}C-NMR, $CDCl_3$, 101 MHz
5
3
1
O
NH_2
Br
rac-**5i**
C_8H_8BrNO
MW = 153.16 gmol^{-1}
^{13}C (ppm)

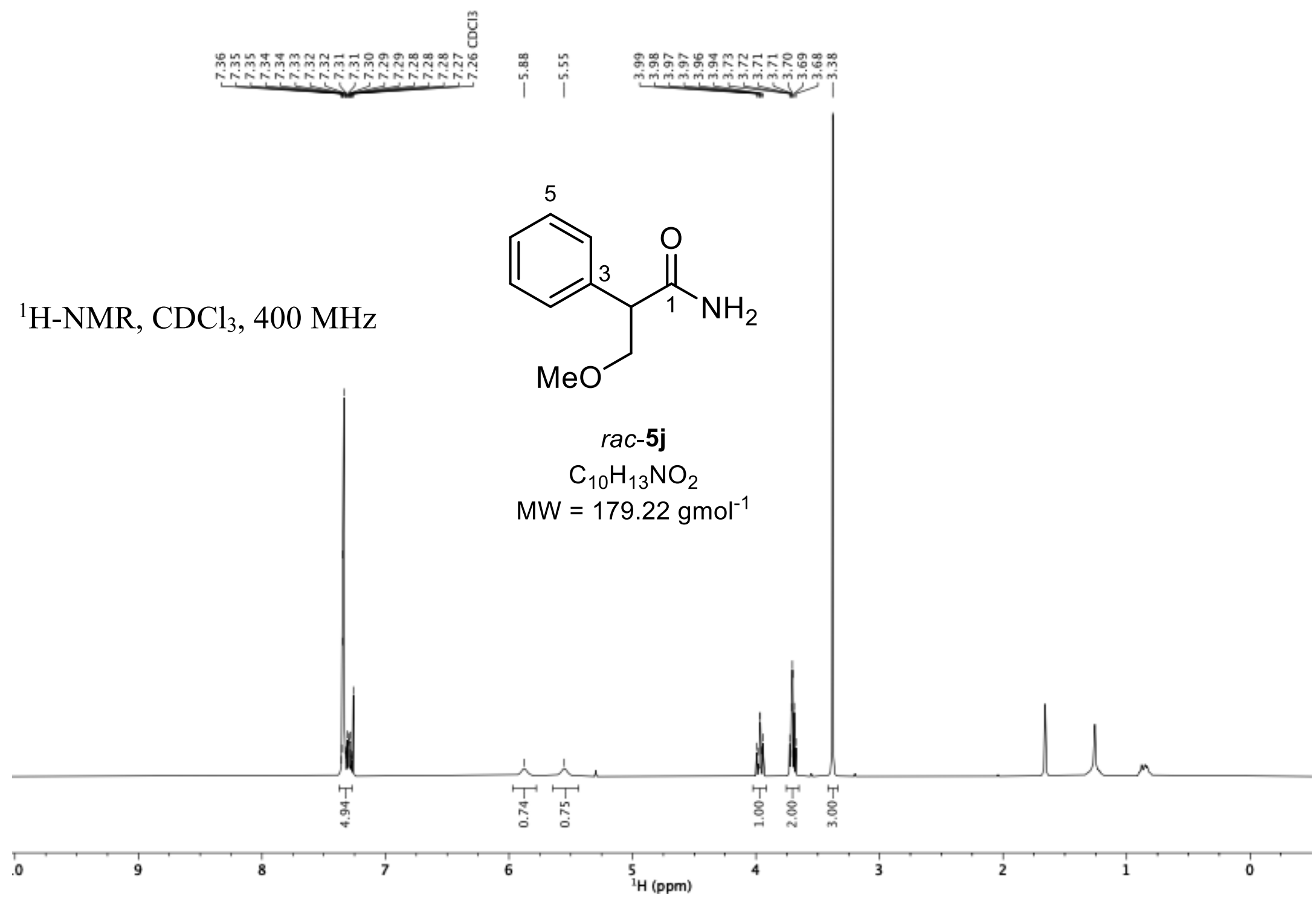

1H-NMR, CDCl3, 400 MHz
rac-5j
C10H13NO2
MW = 179.22 gmol-1
7.36
7.35
7.35
7.34
7.33
7.32
7.32
7.31
7.31
7.30
7.29
7.29
7.28
7.28
7.28
7.27
7.26 CDCl3
5.88
5.55
3.99
3.98
3.97
3.97
3.96
3.94
3.73
3.72
3.71
3.71
3.70
3.69
3.68
3.38
4.94
0.74
0.75
1.00
2.00
3.00
1H (ppm)

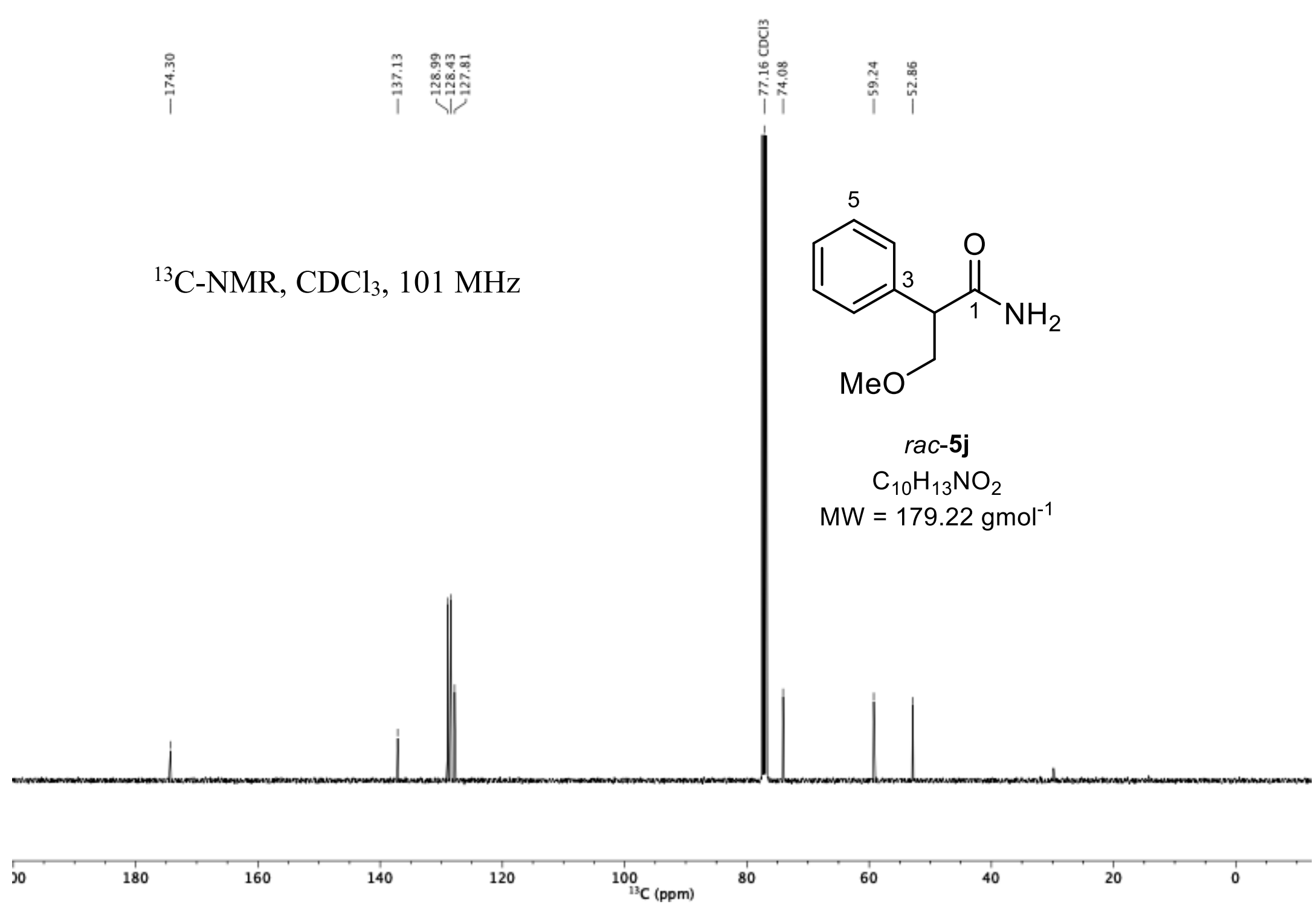

13C-NMR, CDCl3, 101 MHz
rac-5j
C10H13NO2
MW = 179.22 gmol-1
174.30
137.13
128.99
128.43
127.81
77.16 CDCl3
74.08
59.24
52.86
13C (ppm)

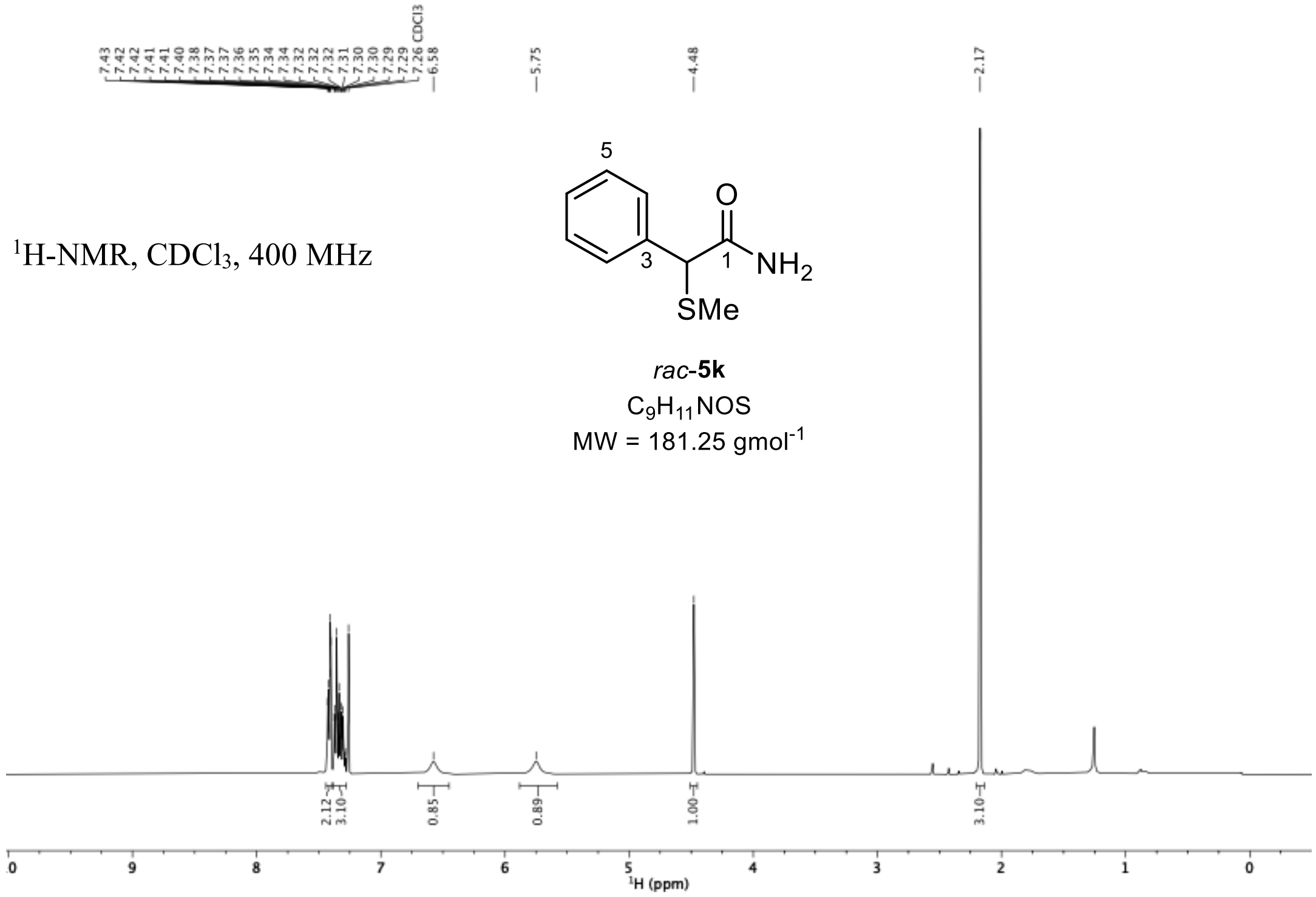

7.43 7.42 7.42 7.41 7.41 7.40 7.38 7.37 7.37 7.36 7.35 7.34 7.34 7.34 7.32 7.32 7.32 7.31 7.31 7.30 7.30 7.29 7.29 7.26 CDCl3
6.58
5.75
4.48
2.17
^{1}H-NMR, $CDCl_3$, 400 MHz
5
3
1
O
NH_2
SMe
rac-**5k**
$C_9H_{11}NOS$
MW = 181.25 gmol^{-1}
2.12
3.10
0.85
0.89
1.00
3.10
^{1}H (ppm)

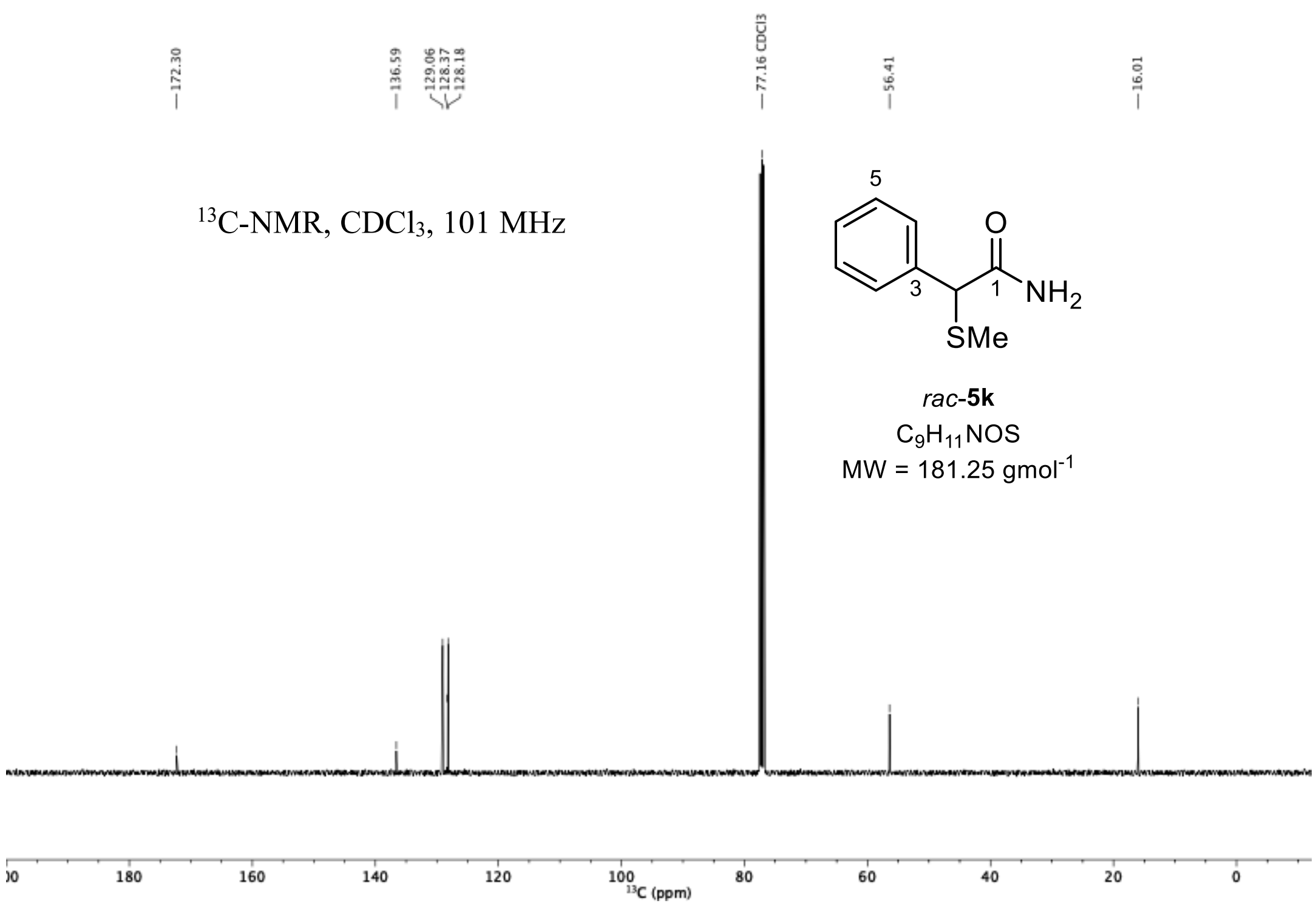

172.30
136.59
129.06
128.37
128.18
77.16 CDCl3
56.41
16.01
^{13}C-NMR, $CDCl_3$, 101 MHz
5
3
1
O
NH_2
SMe
rac-**5k**
$C_9H_{11}NOS$
MW = 181.25 gmol^{-1}
^{13}C (ppm)

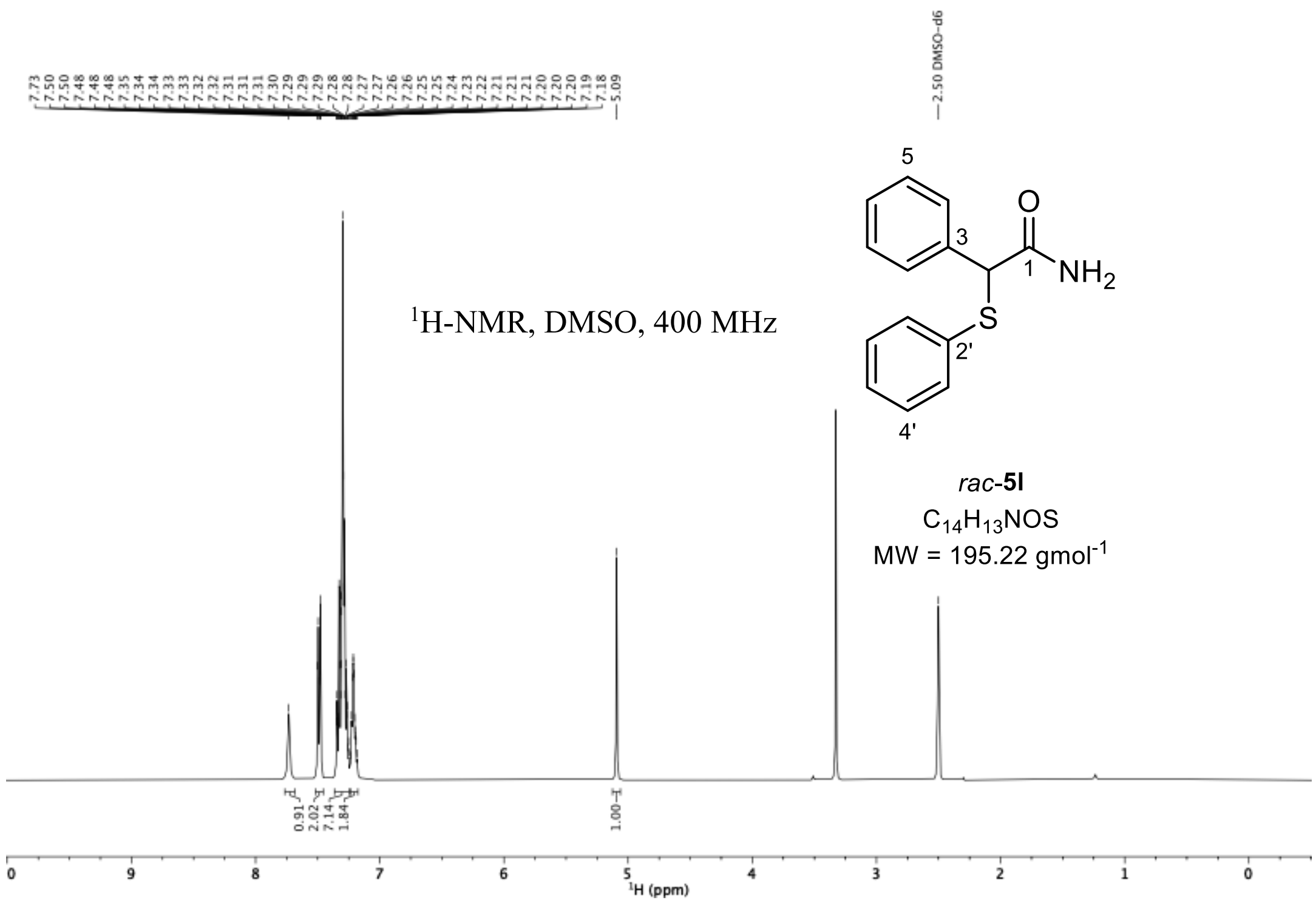
7.73 7.50 7.50 7.48 7.48 7.35 7.34 7.34 7.33 7.33 7.32 7.32 7.31 7.31 7.30 7.29 7.29 7.29 7.28 7.28 7.27 7.27 7.26 7.26 7.25 7.25 7.24 7.23 7.22 7.21 7.21 7.21 7.20 7.20 7.19 7.18 5.09
2.50 DMSO-d6
1H-NMR, DMSO, 400 MHz
5
3
1
O
NH2
S
2'
4'
rac-5l
C14H13NOS
MW = 195.22 gmol-1
0.91
2.02
7.14
1.84
1.00
1H (ppm)

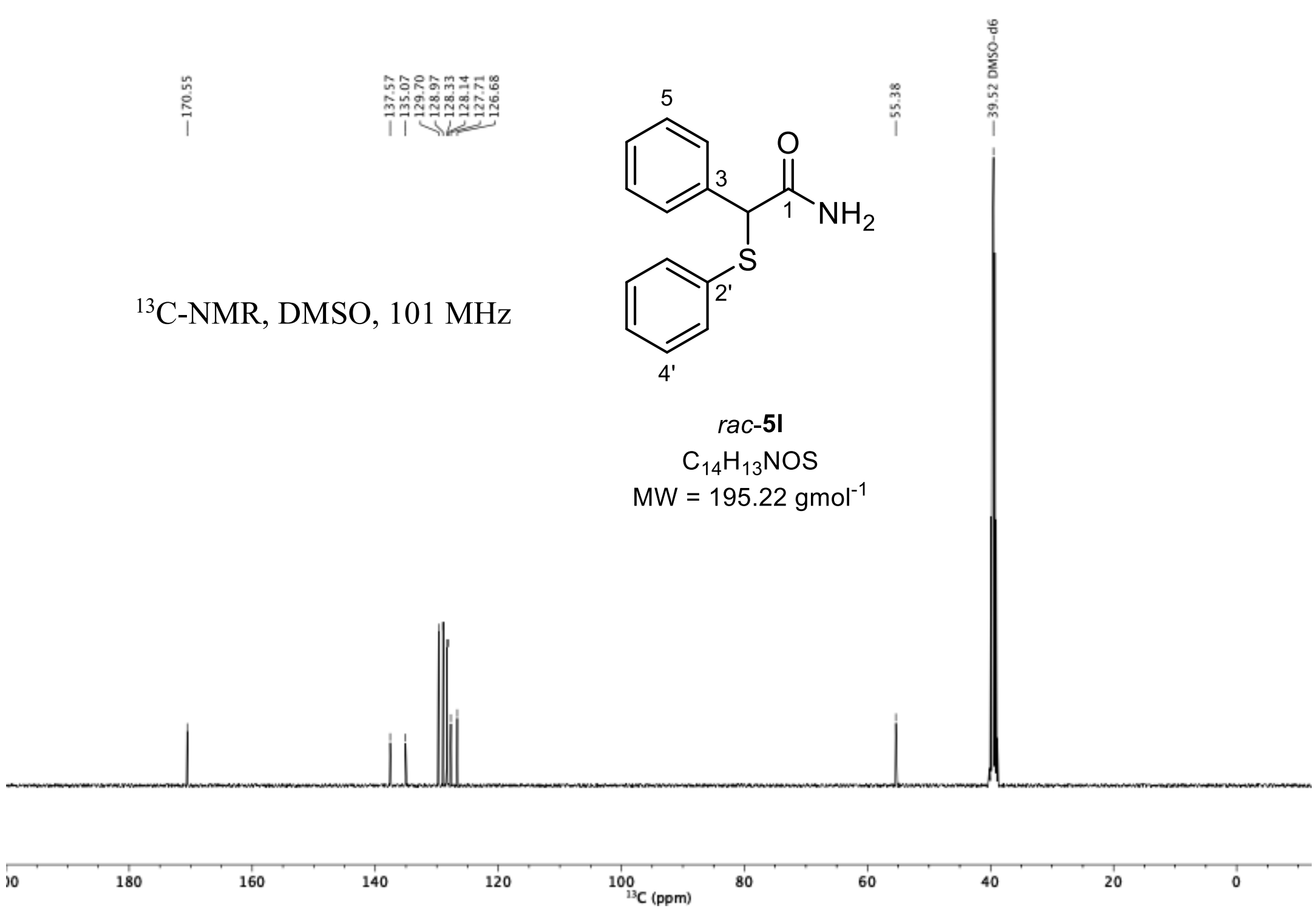
170.55
137.57 135.07 129.70 128.97 128.33 128.14 127.71 126.68
55.38
39.52 DMSO-d6
13C-NMR, DMSO, 101 MHz
5
3
1
O
NH2
S
2'
4'
rac-5l
C14H13NOS
MW = 195.22 gmol-1
13C (ppm)

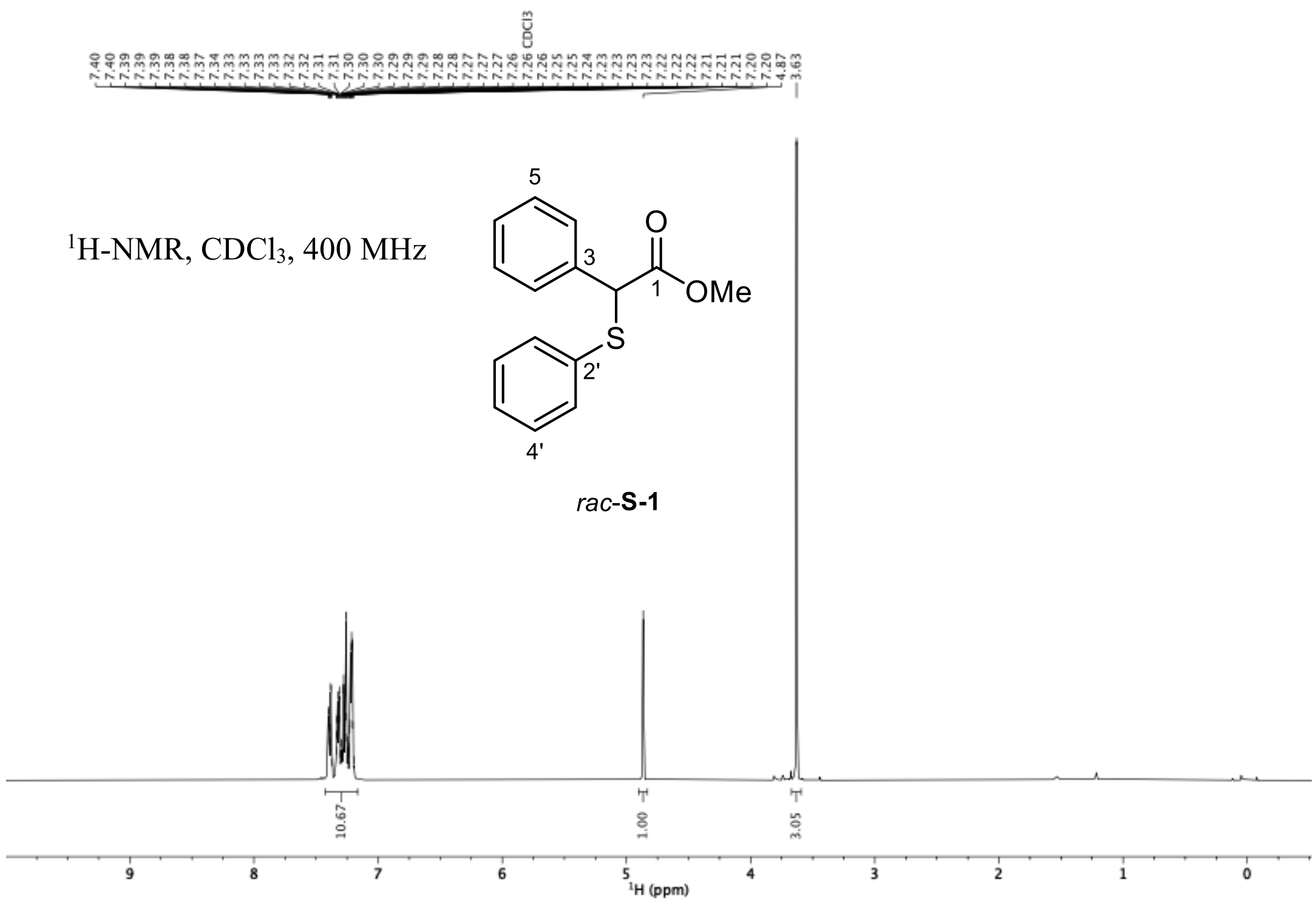

7.40 7.40 7.39 7.39 7.39 7.38 7.38 7.37 7.34 7.33 7.33 7.33 7.33 7.32 7.32 7.31 7.31 7.30 7.30 7.30 7.29 7.29 7.29 7.28 7.28 7.27 7.27 7.27 7.26 7.26 CDCl3 7.26 7.25 7.25 7.24 7.23 7.23 7.23 7.23 7.22 7.22 7.22 7.21 7.21 7.21 7.20 4.87 3.63
1H-NMR, CDCl3, 400 MHz
5
3
O
1
OMe
S
2'
4'
rac-S-1
10.67
1.00
3.05
1H (ppm)

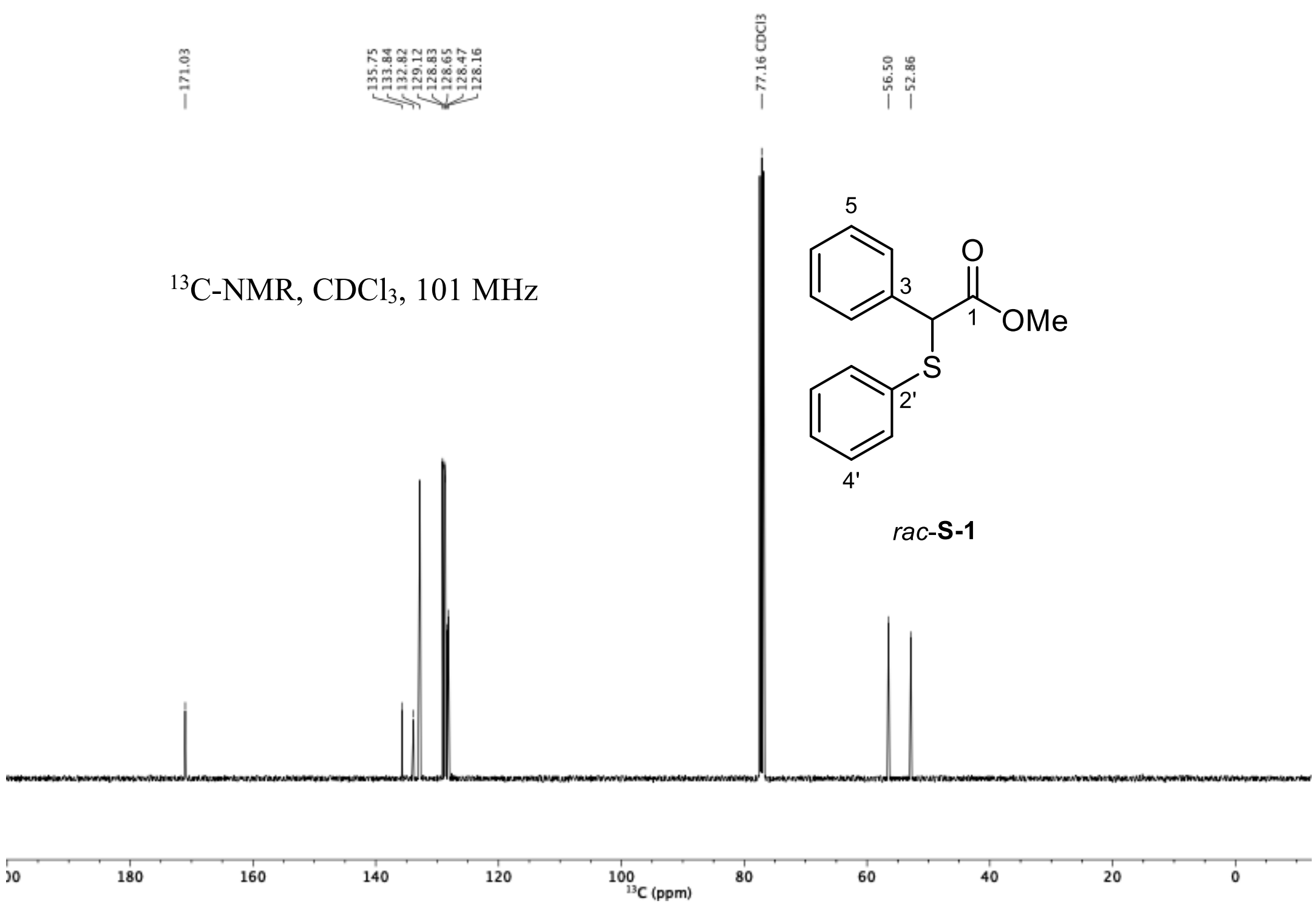

171.03
135.75 133.84 132.82 129.12 128.83 128.65 128.47 128.16
77.16 CDCl3
56.50
52.86
13C-NMR, CDCl3, 101 MHz
5
3
O
1
OMe
S
2'
4'
rac-S-1
13C (ppm)

S17. HPLC Traces

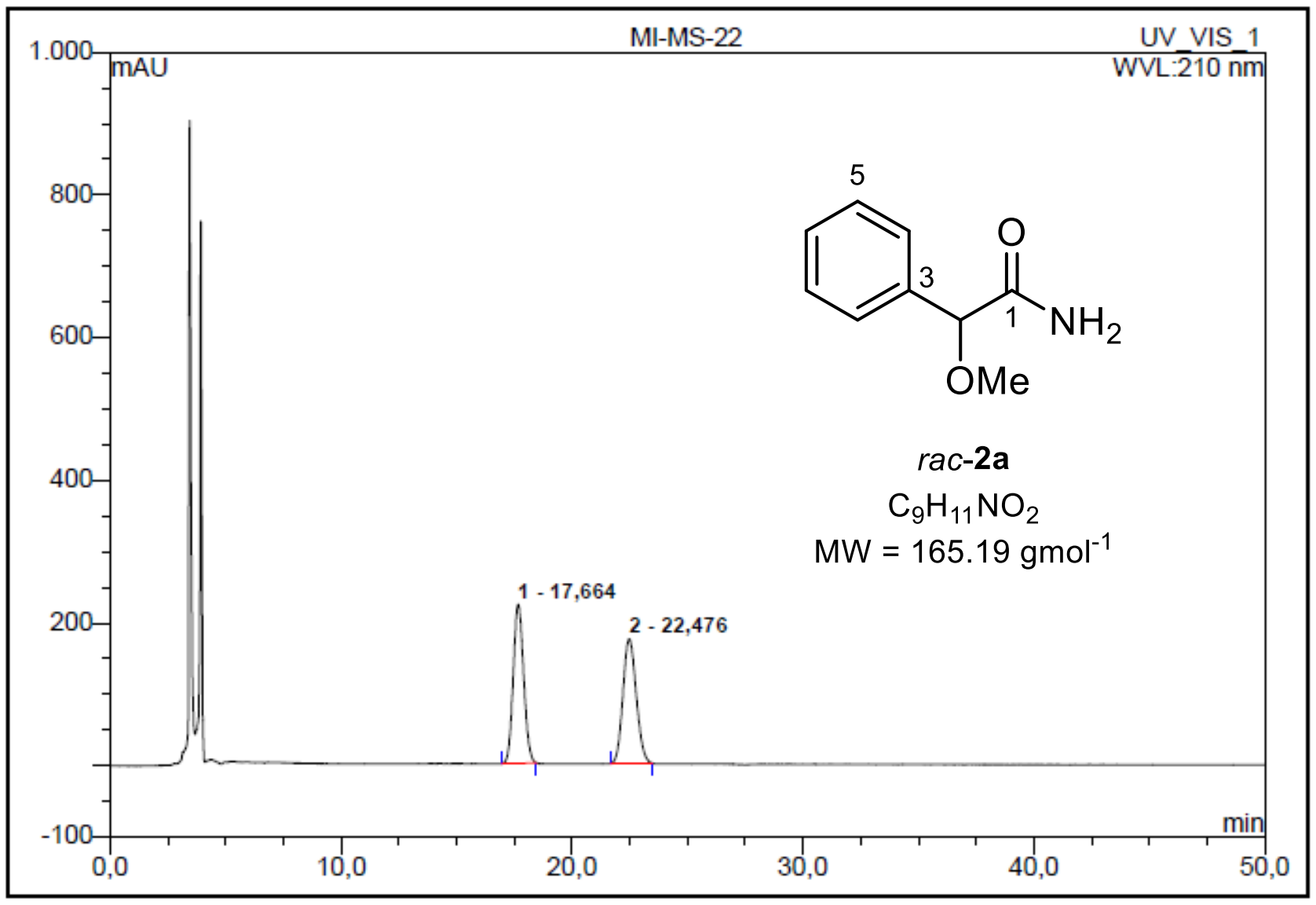

No.	Ret.Time min	Peak Name	Height mAU	Area mAU*min	Rel.Area %	Amount	Type
1	17,66	n.a.	222,715	116,866	50,09	n.a.	BMB
2	22,48	n.a.	174,568	116,454	49,91	n.a.	BMB
Total:			397,283	233,320	100,00	0,000	

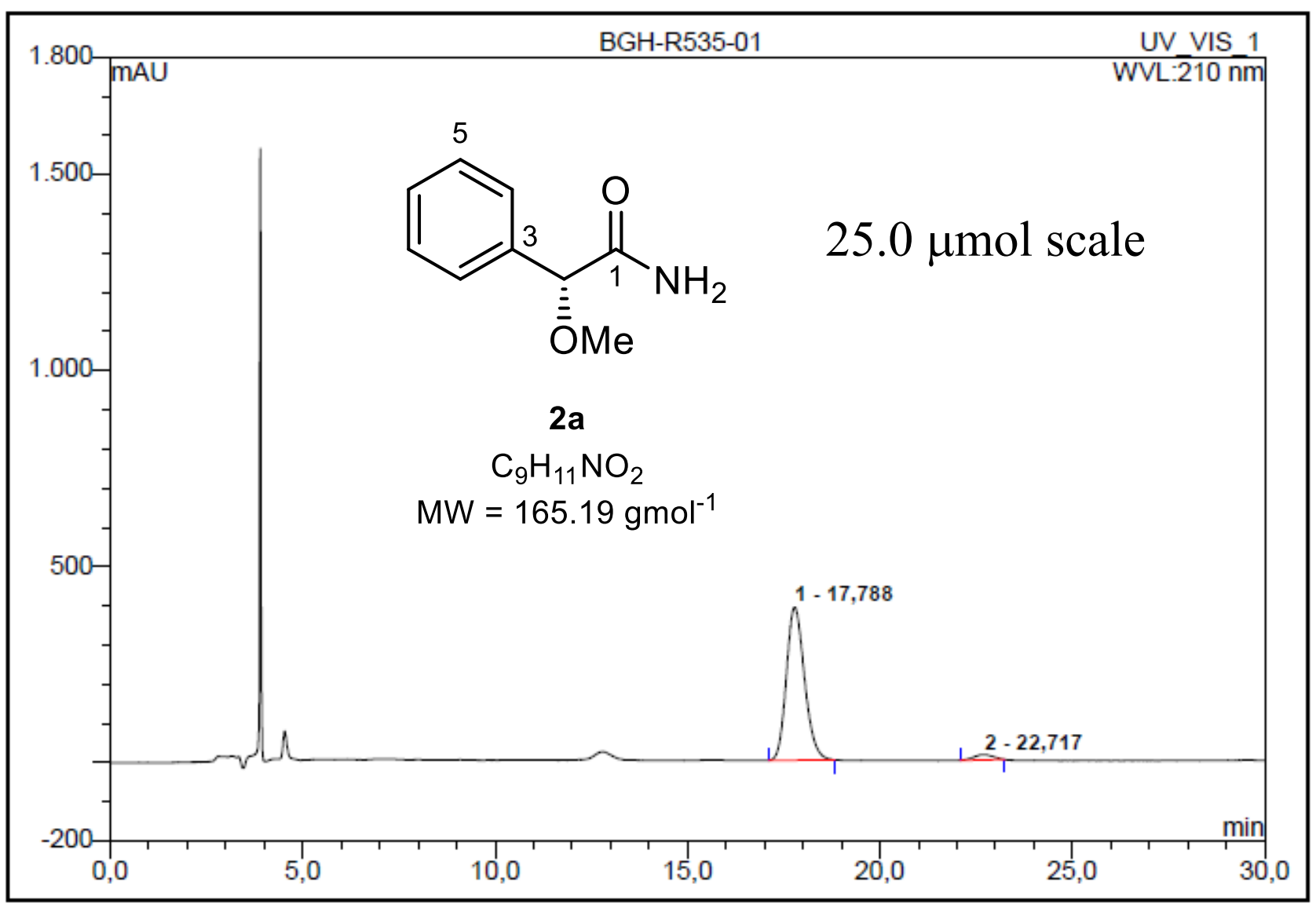

No.	Ret.Time min	Peak Name	Height mAU	Area mAU*min	Rel.Area %	Amount	Type
1	17,79	n.a.	390,353	213,907	96,50	n.a.	BMB
2	22,72	n.a.	13,206	7,759	3,50	n.a.	BMB*
Total:			403,559	221,666	100,00	0,000	

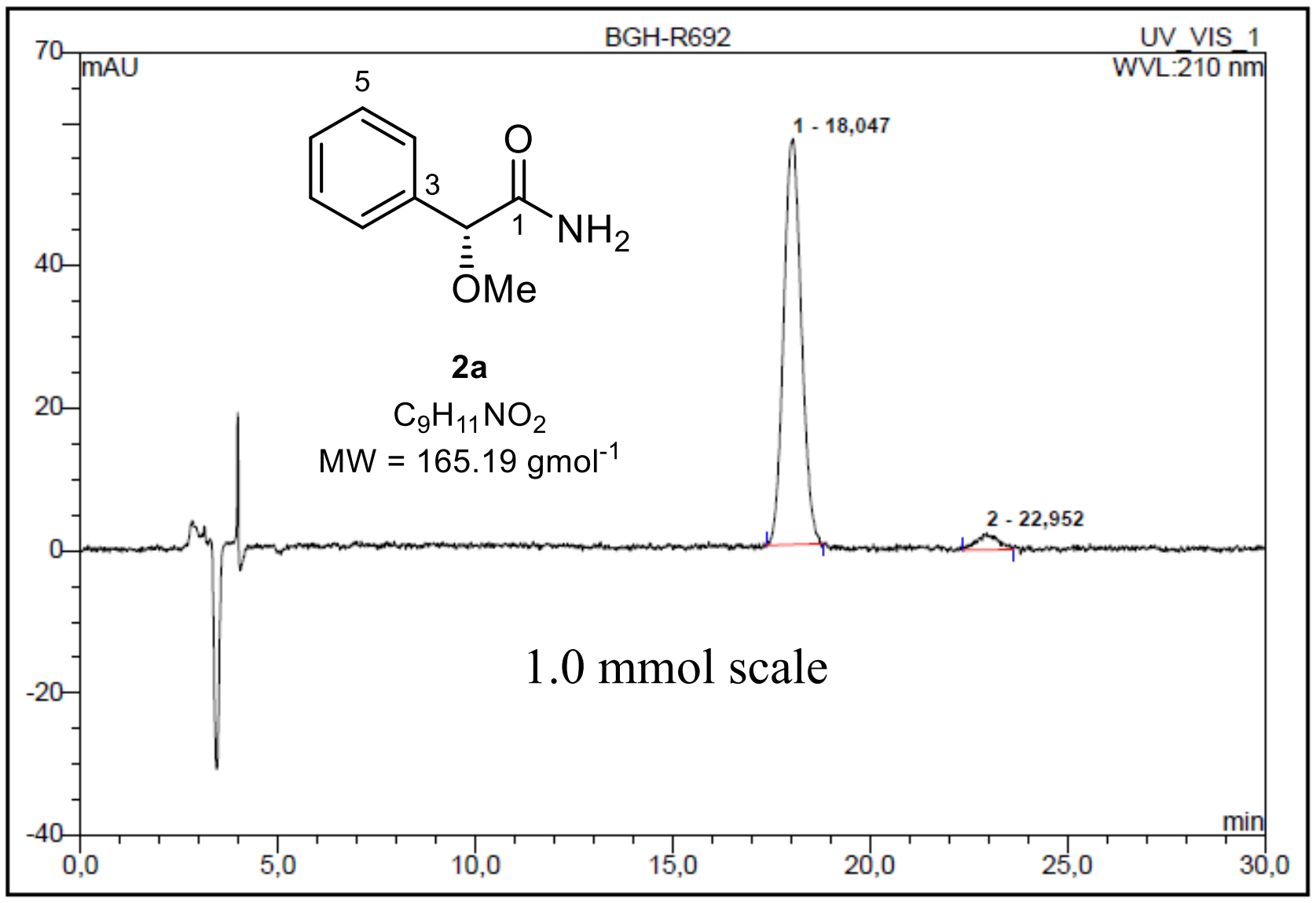

No.	Ret.Time min	Peak Name	Height mAU	Area mAU*min	Rel.Area %	Amount	Type
1	18,05	n.a.	57,043	29,512	95,41	n.a.	BMB*
2	22,95	n.a.	2,428	1,419	4,59	n.a.	BMB*
Total:			59,471	30,931	100,00	0,000	

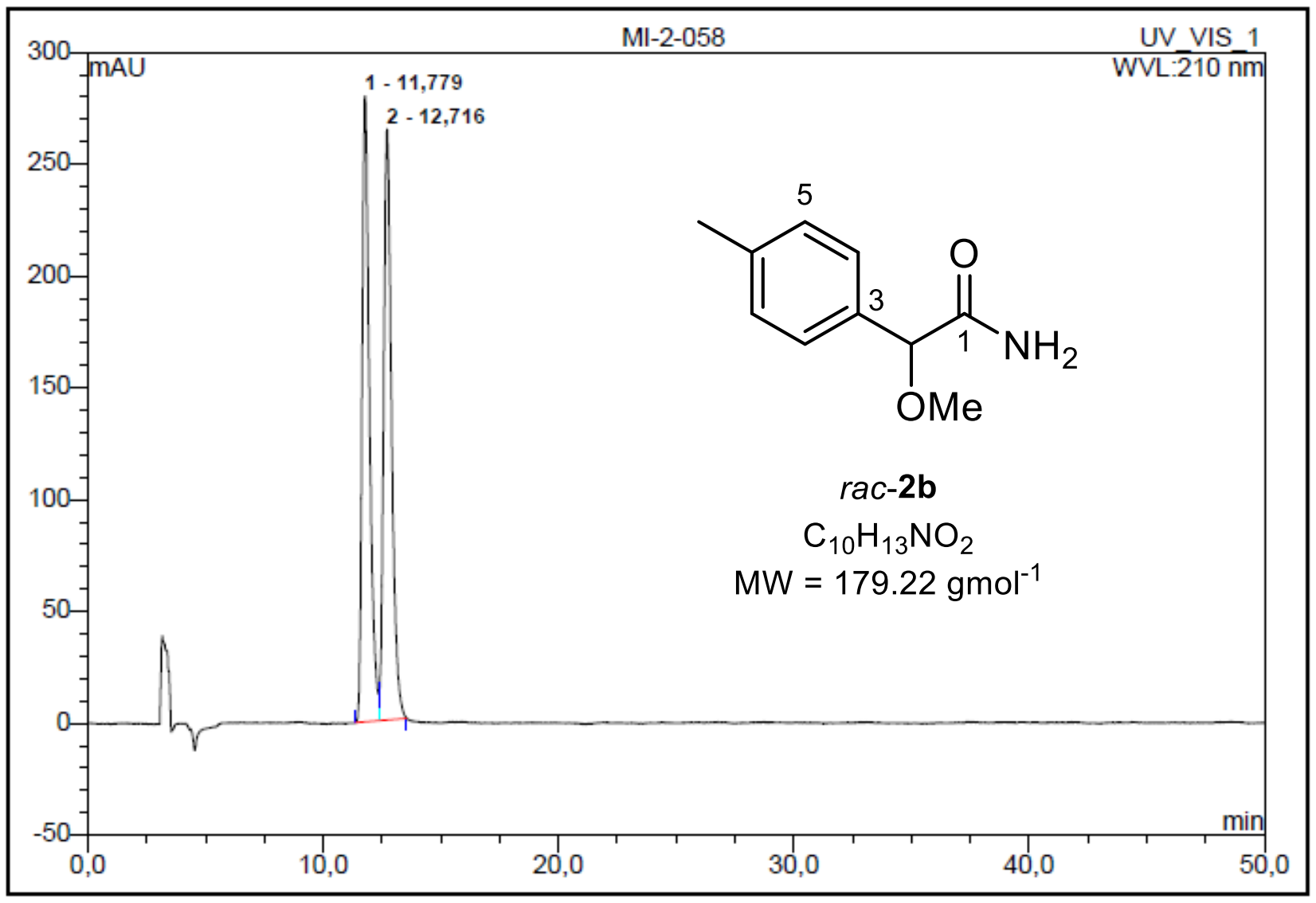

No.	Ret.Time min	Peak Name	Height mAU	Area mAU*min	Rel.Area %	Amount	Type
1	11,78	n.a.	279,830	101,368	49,61	n.a.	BM
2	12,72	n.a.	264,150	102,943	50,39	n.a.	MB
Total:			543,980	204,311	100,00	0,000	

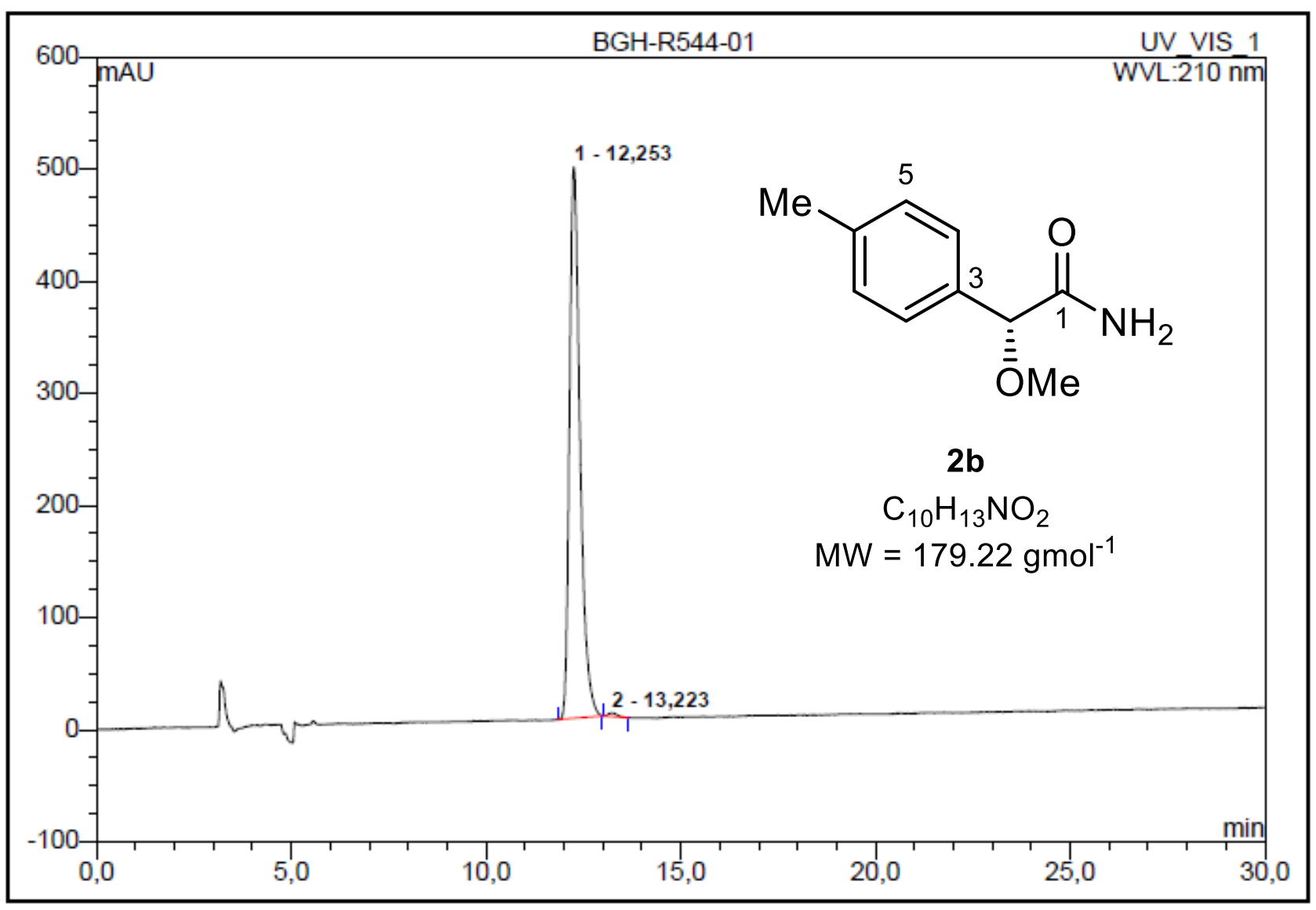

No.	Ret.Time min	Peak Name	Height mAU	Area mAU*min	Rel.Area %	Amount	Type
1	12,25	n.a.	491,783	154,066	99,42	n.a.	BMB*
2	13,22	n.a.	3,184	0,896	0,58	n.a.	BMB*
Total:			494,966	154,962	100,00	0,000	

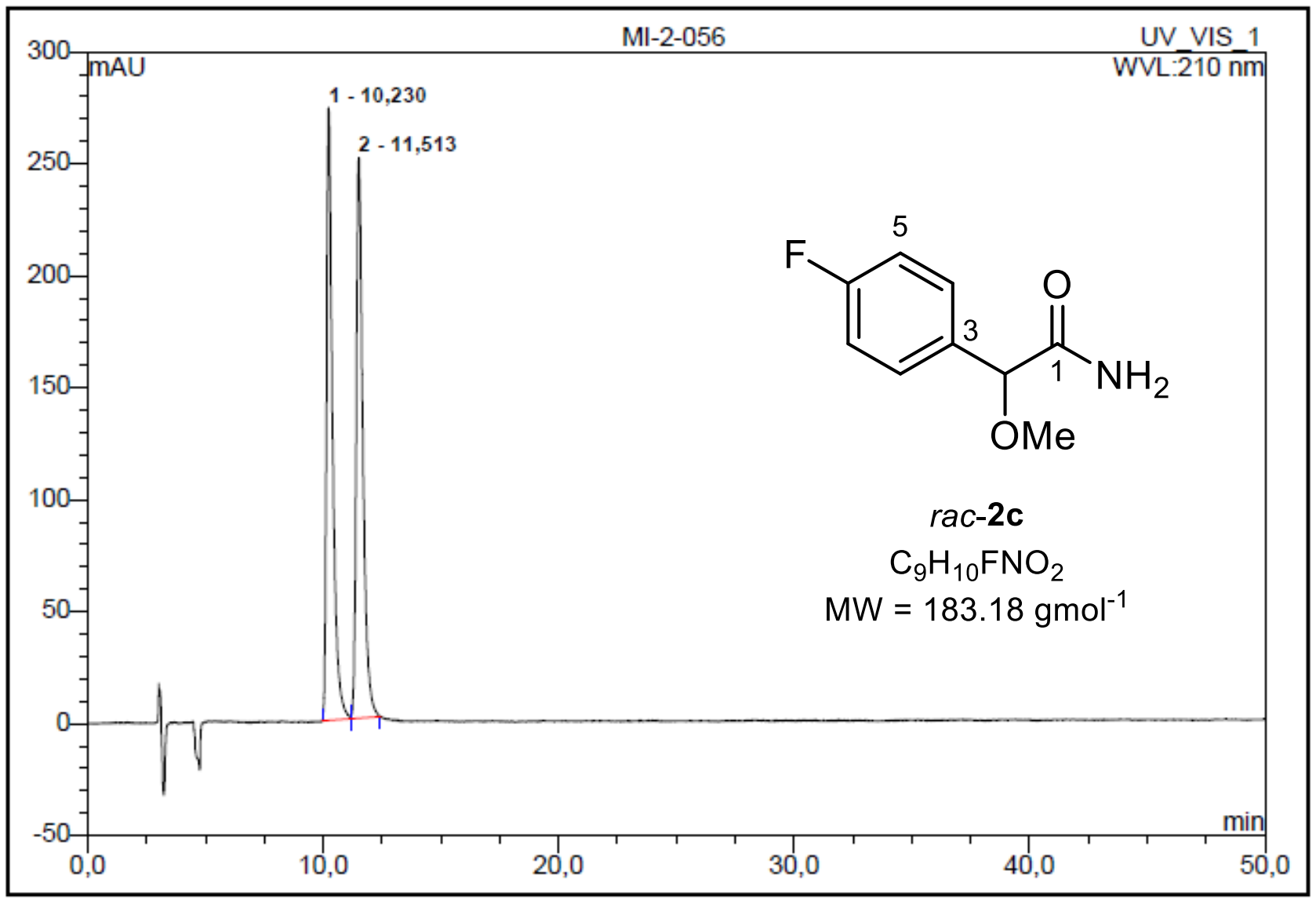

No.	Ret.Time min	Peak Name	Height mAU	Area mAU*min	Rel.Area %	Amount	Type
1	10,23	n.a.	273,659	81,911	50,22	n.a.	BM
2	11,51	n.a.	250,368	81,190	49,78	n.a.	MB
Total:			524,027	163,102	100,00	0,000	

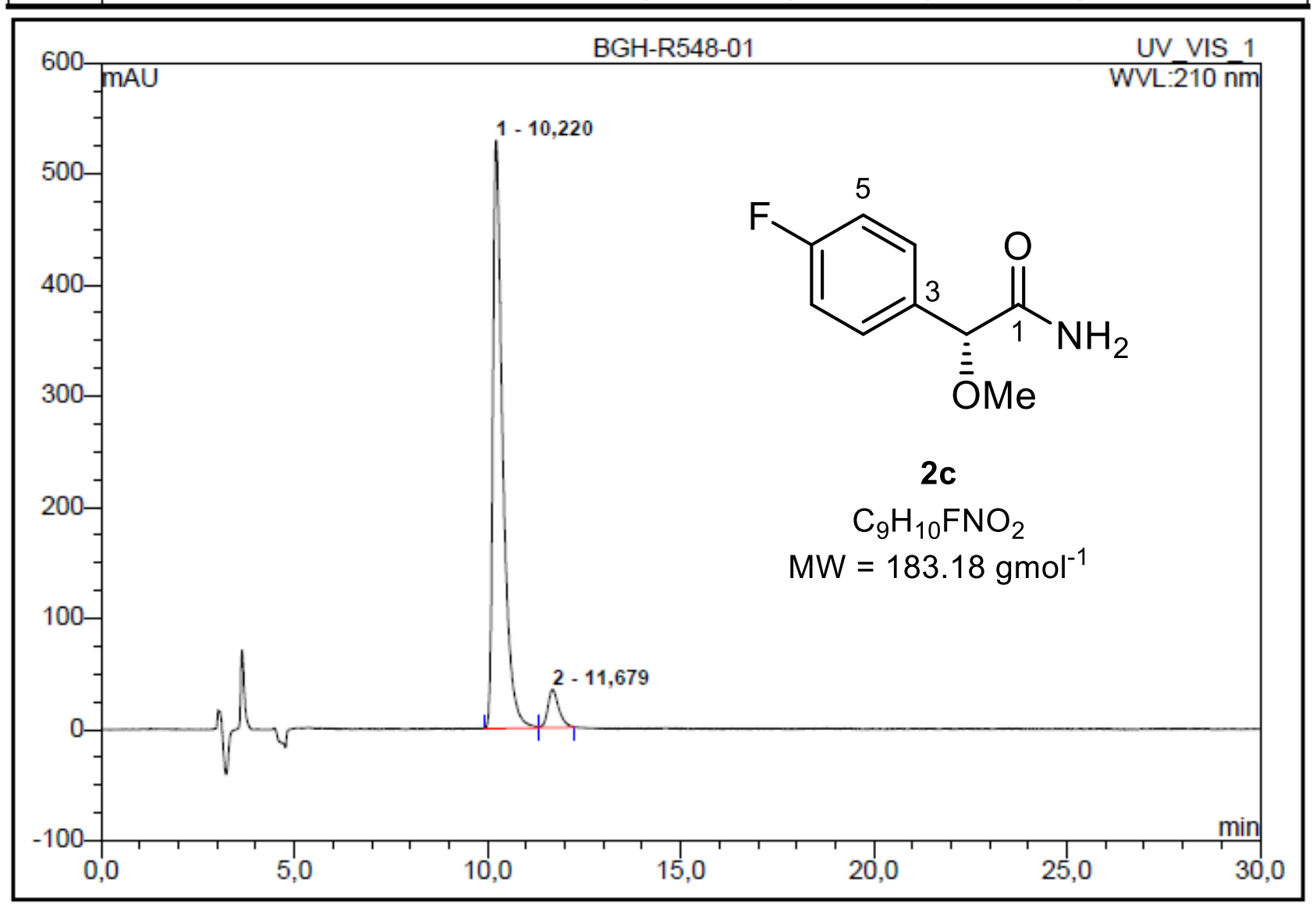

No.	Ret.Time min	Peak Name	Height mAU	Area mAU*min	Rel.Area %	Amount	Type
1	10,22	n.a.	529,193	156,914	93,32	n.a.	BM
2	11,68	n.a.	34,254	11,236	6,68	n.a.	MB
Total:			563,447	168,149	100,00	0,000	

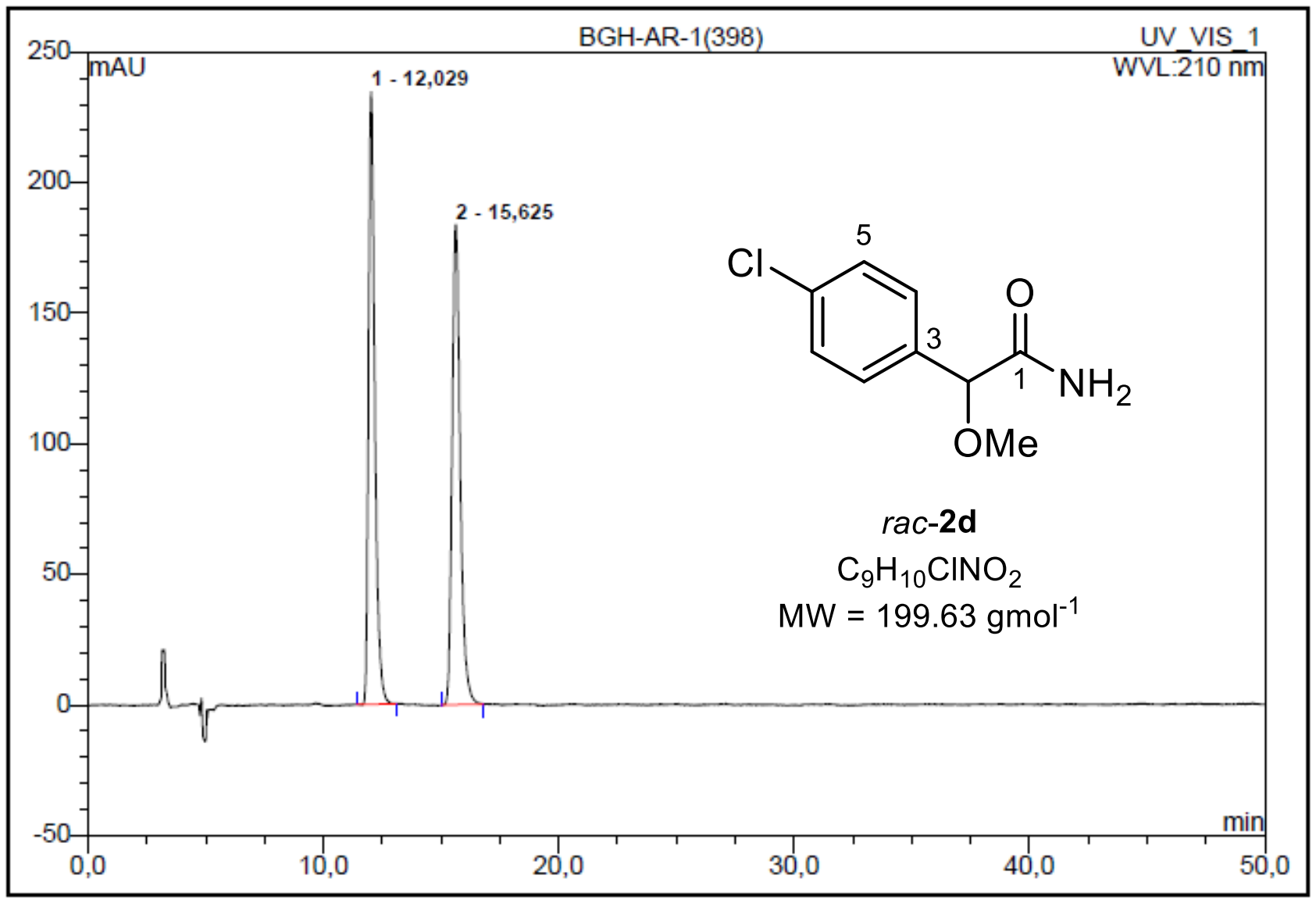

No.	Ret.Time min	Peak Name	Height mAU	Area mAU*min	Rel.Area %	Amount	Type
1	12,03	n.a.	234,372	72,447	49,87	n.a.	BMB*
2	15,62	n.a.	183,687	72,833	50,13	n.a.	BMB*
Total:			418,059	145,280	100,00	0,000	

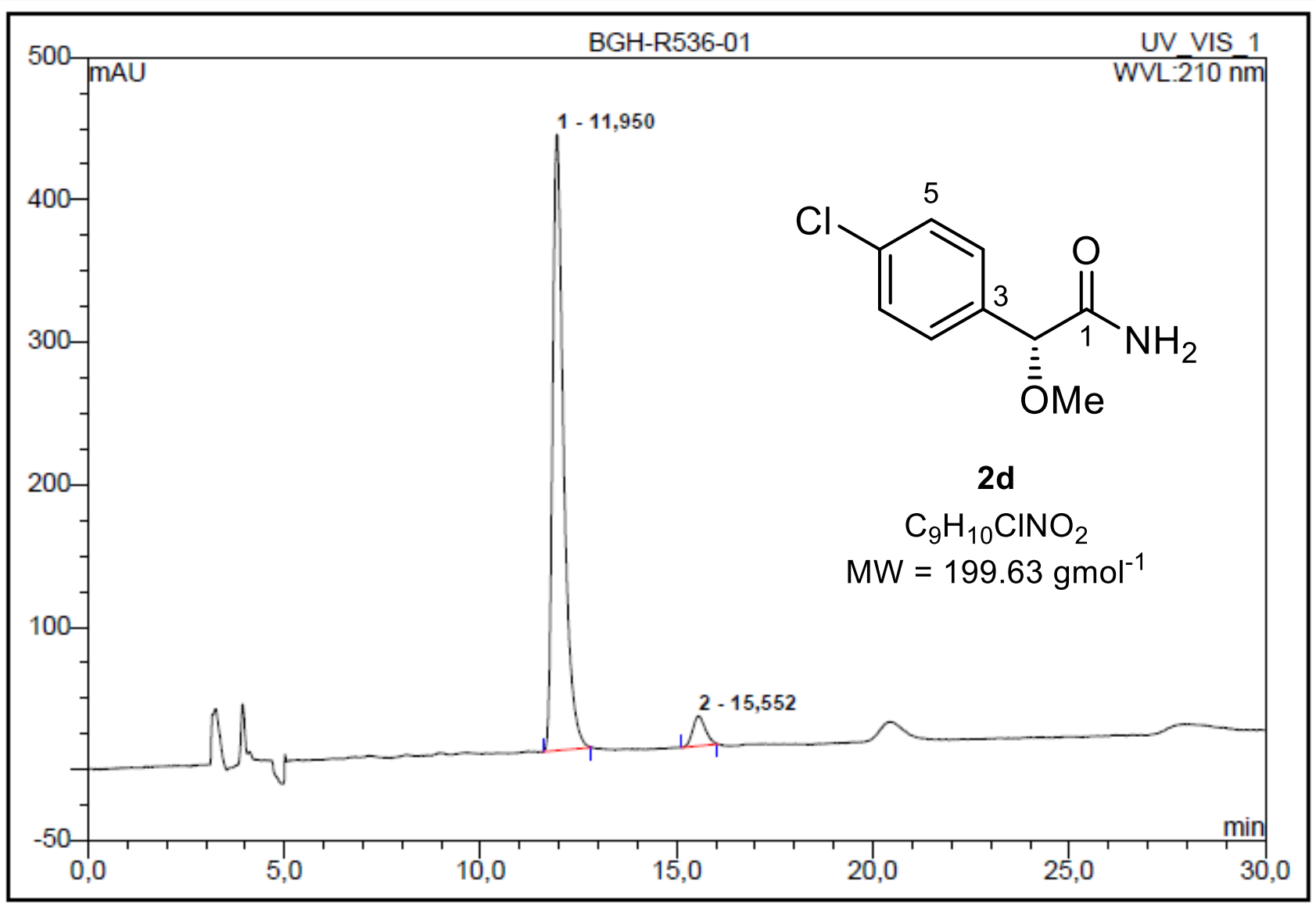

No.	Ret.Time min	Peak Name	Height mAU	Area mAU*min	Rel.Area %	Amount	Type
1	11,95	n.a.	432,486	141,052	94,77	n.a.	BMB
2	15,55	n.a.	21,204	7,789	5,23	n.a.	BMB*
Total:			453,691	148,841	100,00	0,000	

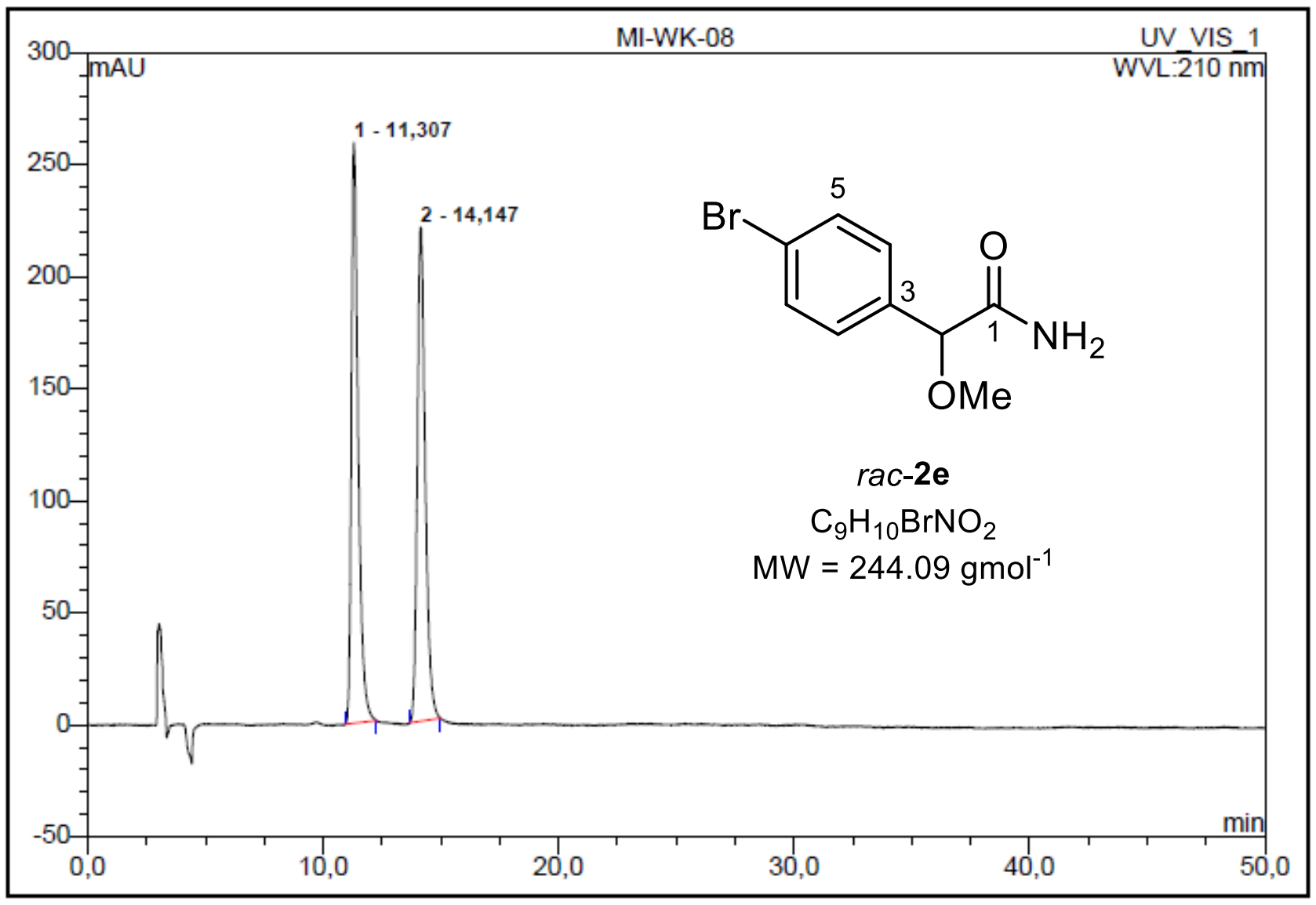

No.	Ret.Time min	Peak Name	Height mAU	Area mAU*min	Rel.Area %	Amount	Type
1	11,31	n.a.	259,159	86,596	50,30	n.a.	BMB
2	14,15	n.a.	220,468	85,577	49,70	n.a.	BMB
Total:			479,627	172,174	100,00	0,000	

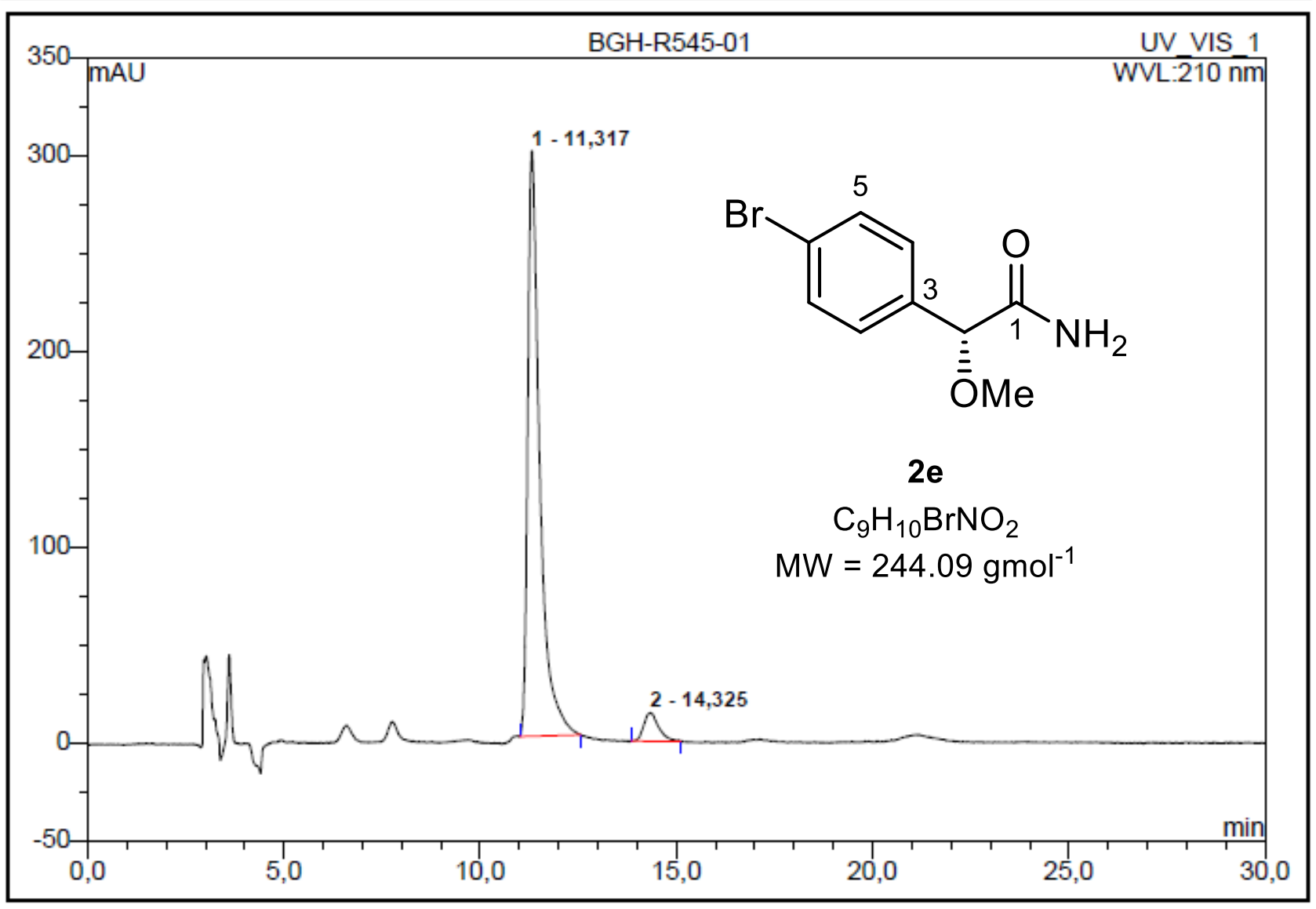

No.	Ret.Time min	Peak Name	Height mAU	Area mAU*min	Rel.Area %	Amount	Type
1	11,32	n.a.	298,815	106,449	94,41	n.a.	BMB
2	14,32	n.a.	14,460	6,300	5,59	n.a.	BMB*
Total:			313,275	112,749	100,00	0,000	

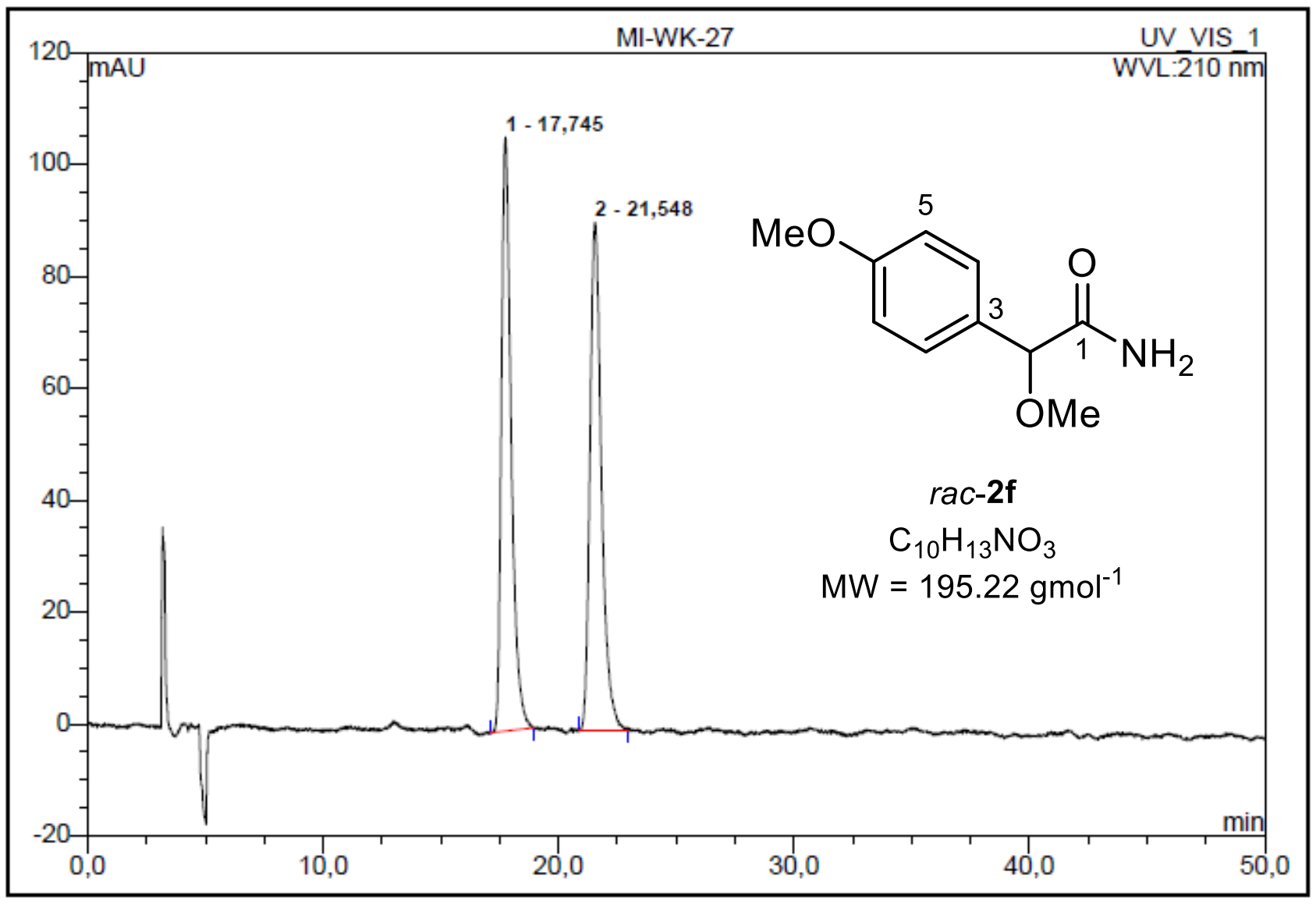

No.	Ret.Time min	Peak Name	Height mAU	Area mAU*min	Rel.Area %	Amount	Type
1	17,74	n.a.	106,125	51,046	49,75	n.a.	BMB*
2	21,55	n.a.	90,846	51,555	50,25	n.a.	BMB*
Total:			196,971	102,601	100,00	0,000	

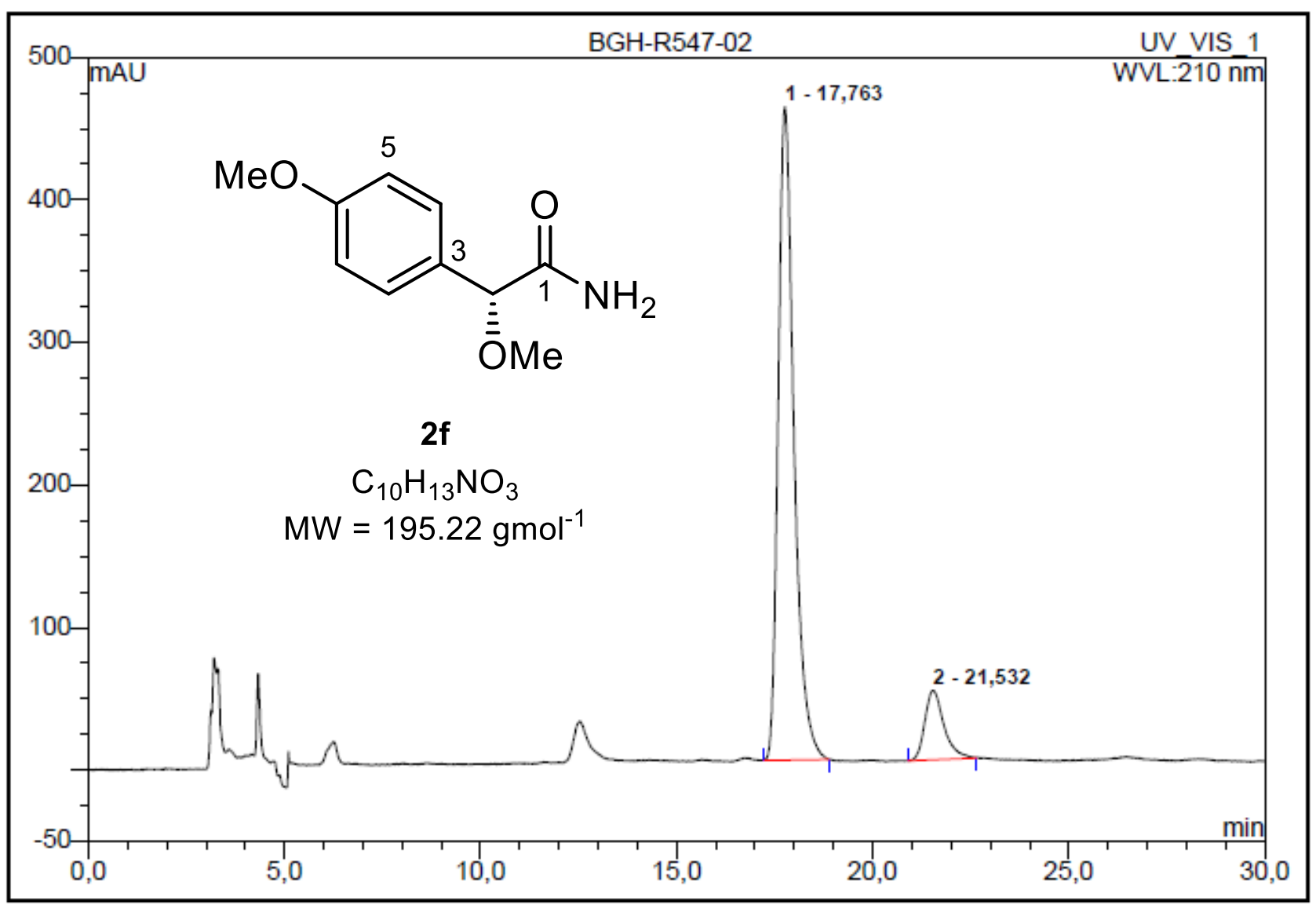

No.	Ret.Time min	Peak Name	Height mAU	Area mAU*min	Rel.Area %	Amount	Type
1	17,76	n.a.	458,293	213,600	88,62	n.a.	BMB
2	21,53	n.a.	48,522	27,429	11,38	n.a.	BMB*
Total:			506,815	241,029	100,00	0,000	

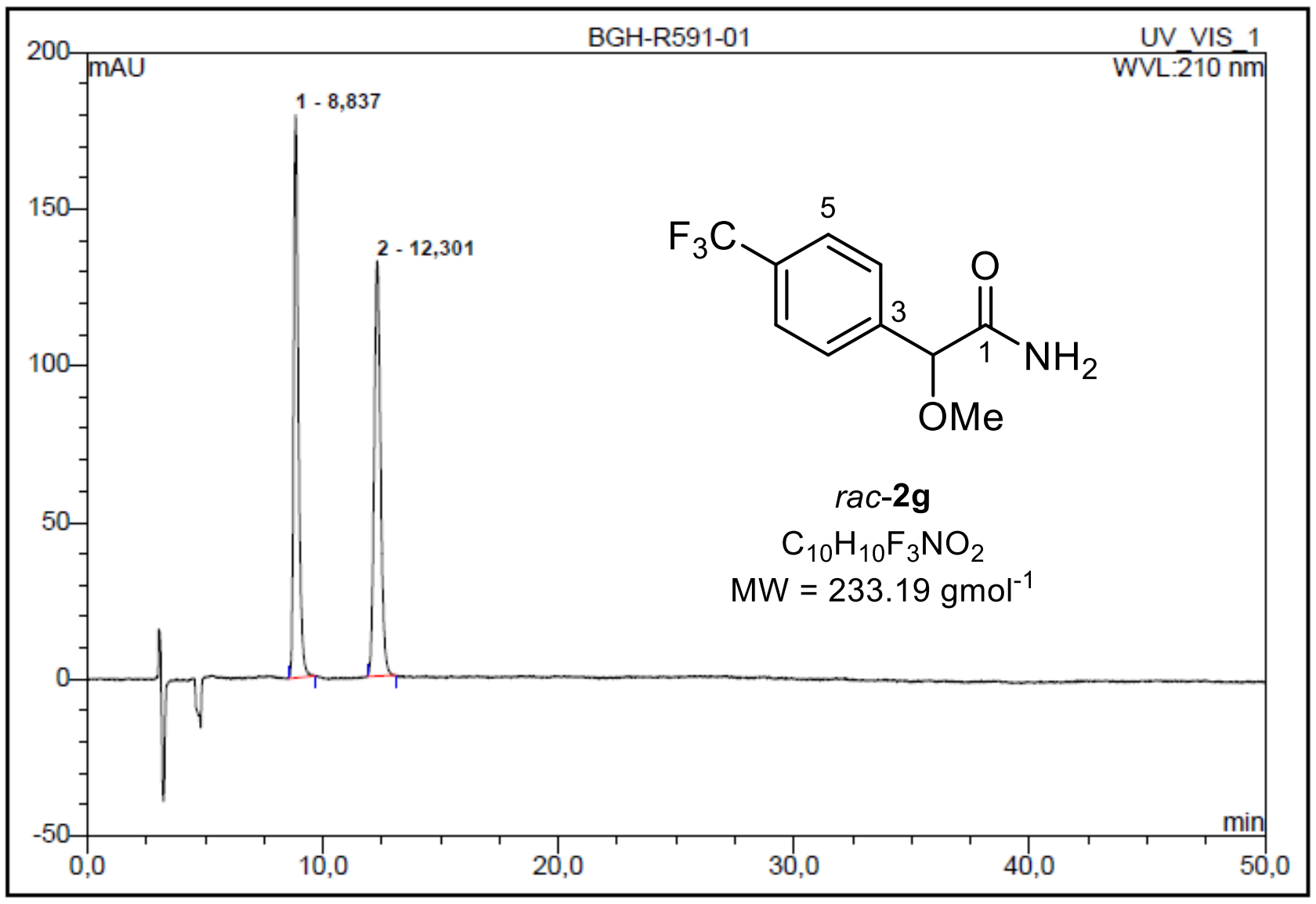

No.	Ret.Time min	Peak Name	Height mAU	Area mAU*min	Rel.Area %	Amount	Type
1	8,84	n.a.	179,521	42,063	50,31	n.a.	BMB*
2	12,30	n.a.	132,499	41,546	49,69	n.a.	BMB*
Total:			312,020	83,609	100,00	0,000	

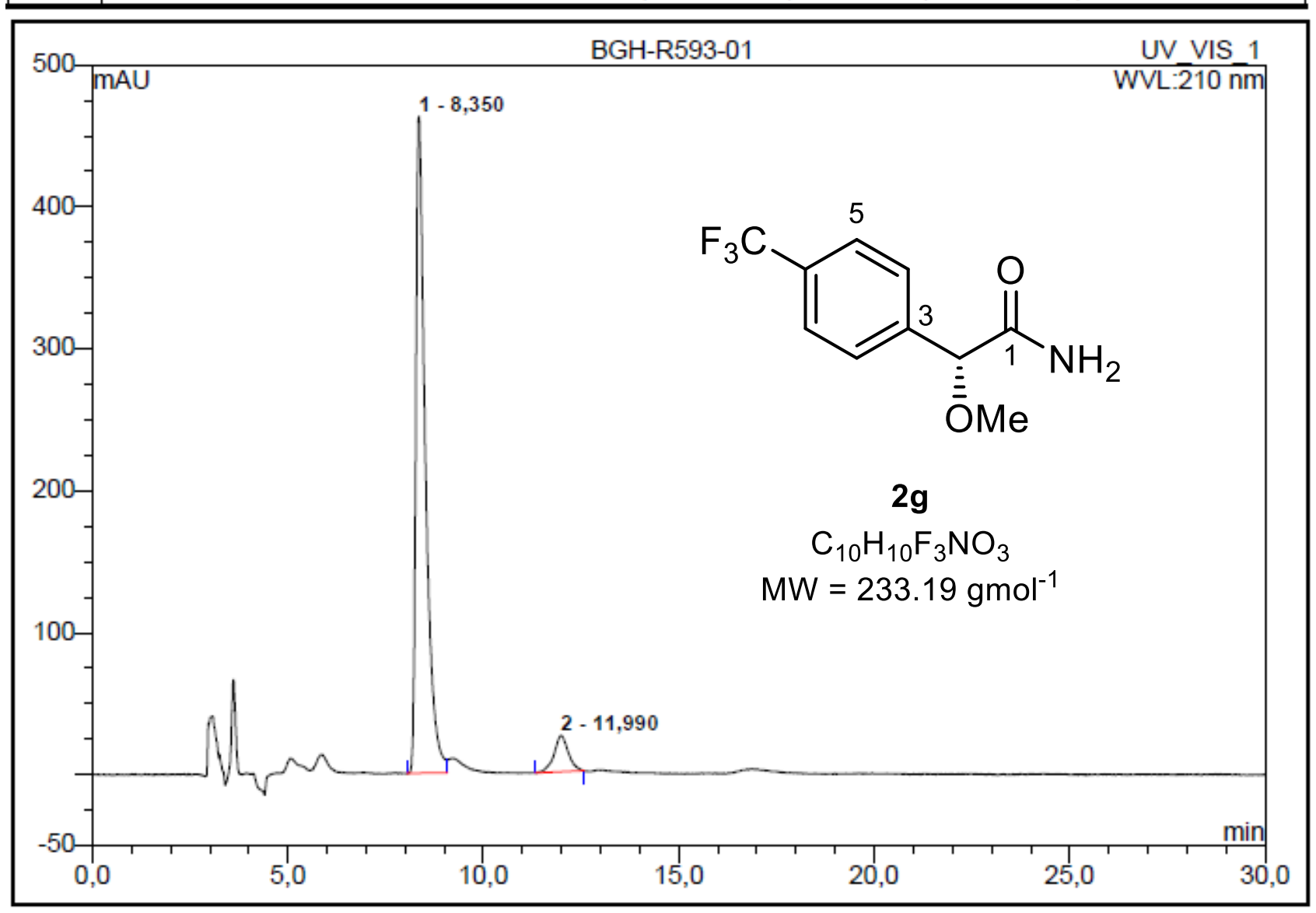

No.	Ret.Time min	Peak Name	Height mAU	Area mAU*min	Rel.Area %	Amount	Type
1	8,35	n.a.	462,837	131,621	92,36	n.a.	BM *
2	11,99	n.a.	25,483	10,883	7,64	n.a.	BMB*
Total:			488,320	142,505	100,00	0,000	

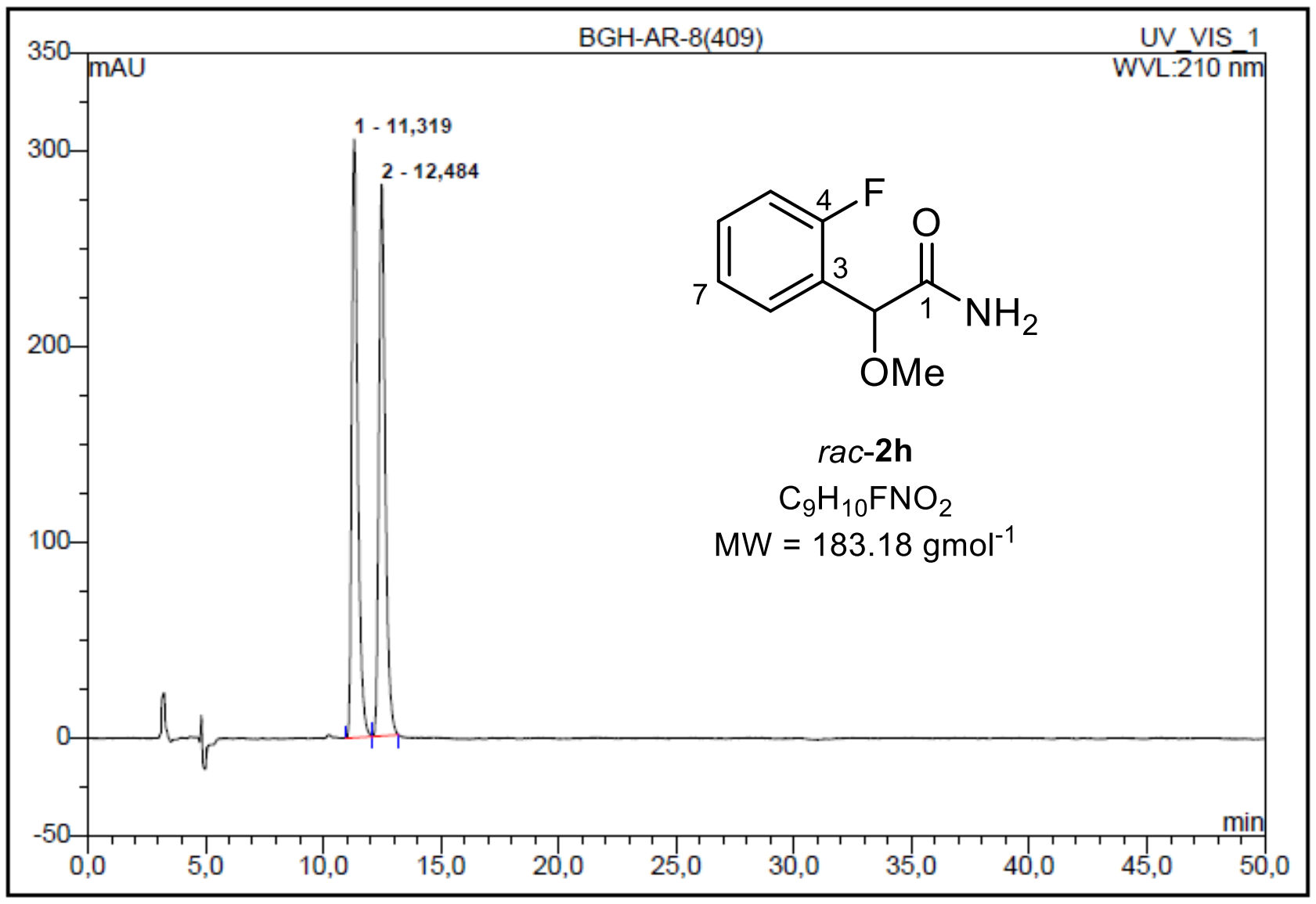

No.	Ret.Time min	Peak Name	Height mAU	Area mAU*min	Rel.Area %	Amount	Type
1	11,32	n.a.	305,786	89,619	50,24	n.a.	BM
2	12,48	n.a.	281,524	88,764	49,76	n.a.	MB
Total:			587,311	178,383	100,00	0,000	

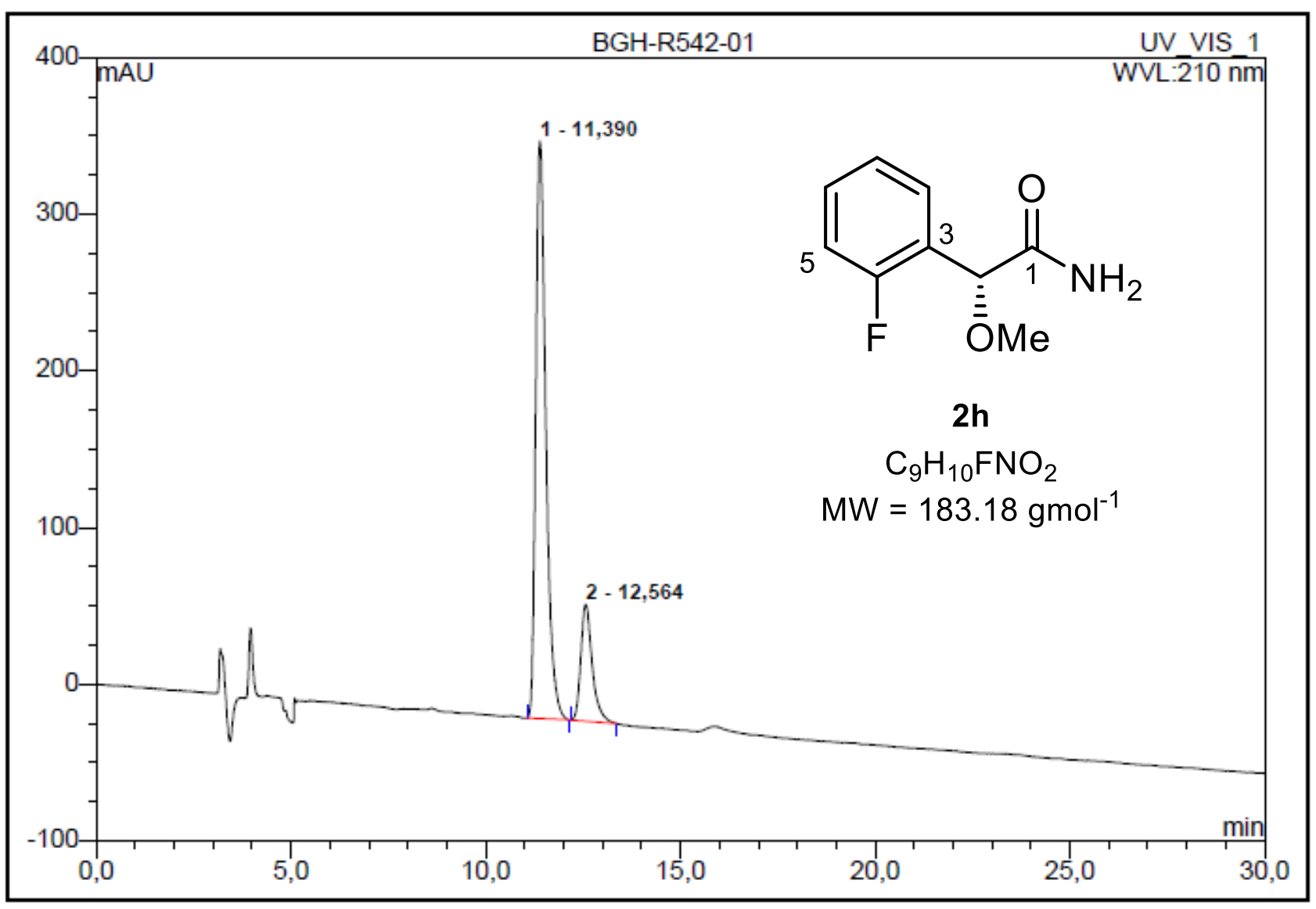

No.	Ret.Time min	Peak Name	Height mAU	Area mAU*min	Rel.Area %	Amount	Type
1	11,39	n.a.	368,417	110,195	81,27	n.a.	BMB
2	12,56	n.a.	74,472	25,404	18,73	n.a.	BMB
Total:			442,889	135,599	100,00	0,000	

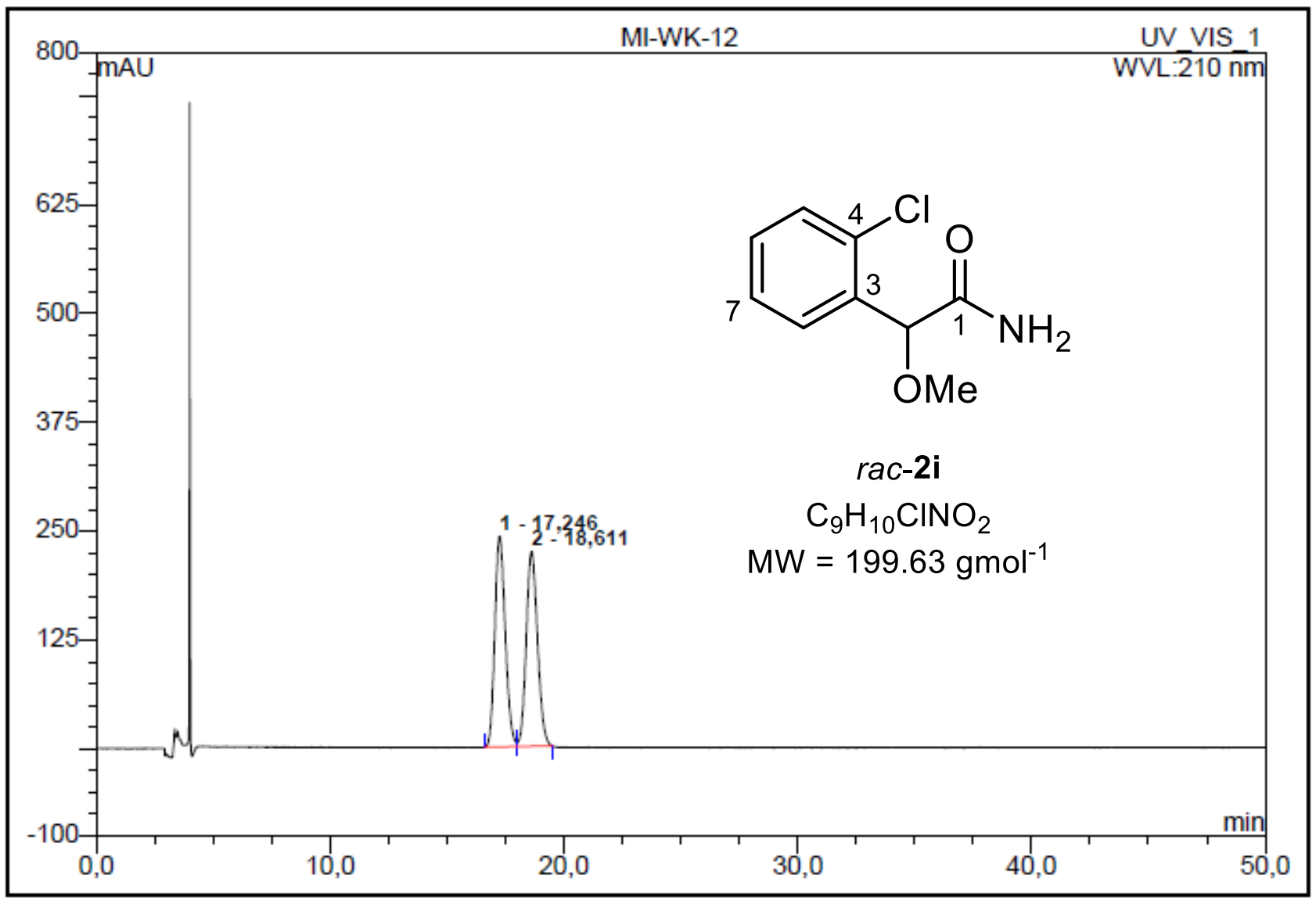

No.	Ret.Time min	Peak Name	Height mAU	Area mAU*min	Rel.Area %	Amount	Type
1	17,25	n.a.	241,566	125,437	50,07	n.a.	BM
2	18,61	n.a.	223,486	125,086	49,93	n.a.	MB
Total:			465,052	250,523	100,00	0,000	

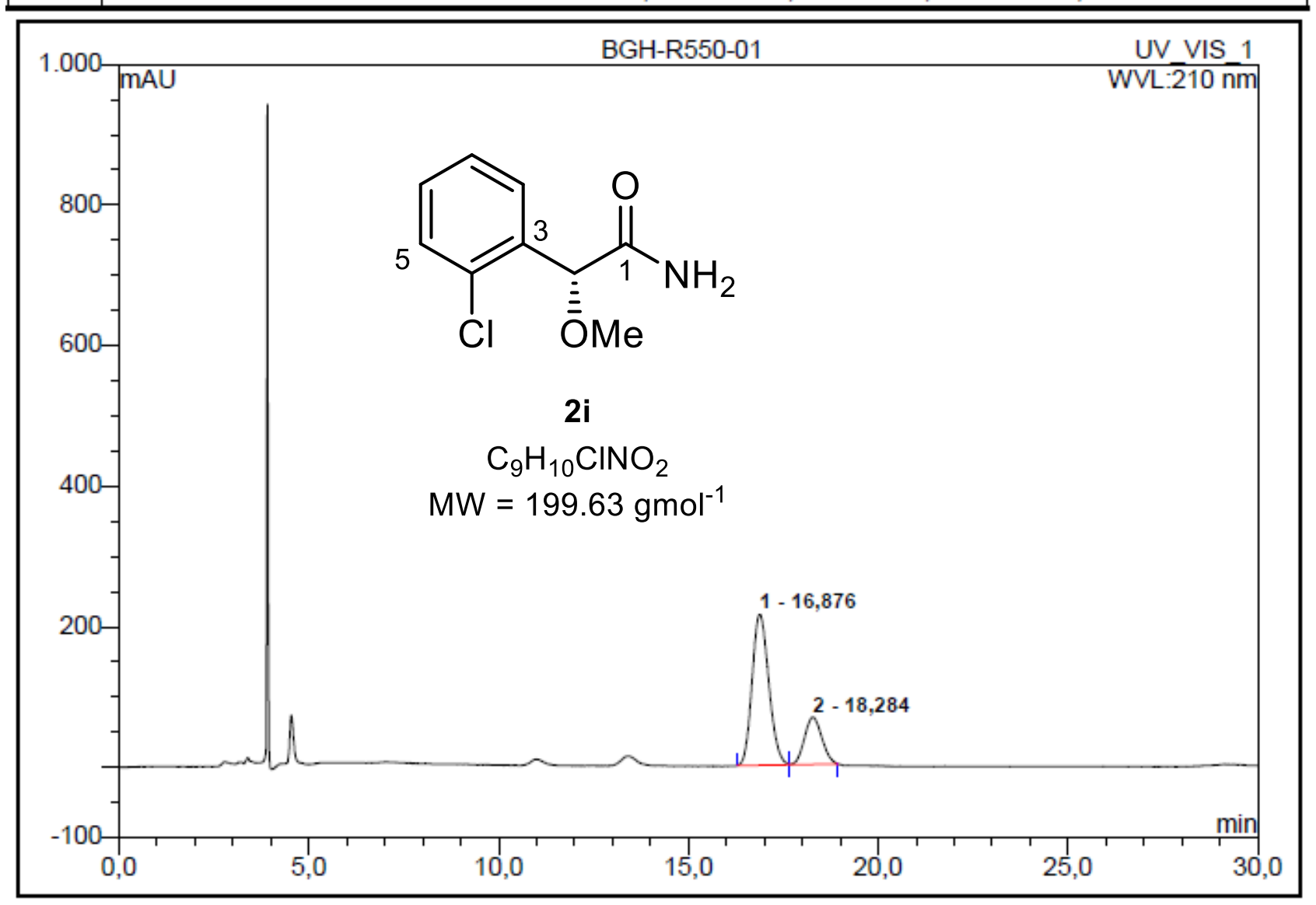

No.	Ret.Time min	Peak Name	Height mAU	Area mAU*min	Rel.Area %	Amount	Type
1	16,88	n.a.	214,805	109,899	75,45	n.a.	BM
2	18,28	n.a.	67,205	35,760	24,55	n.a.	MB
Total:			282,010	145,659	100,00	0,000	

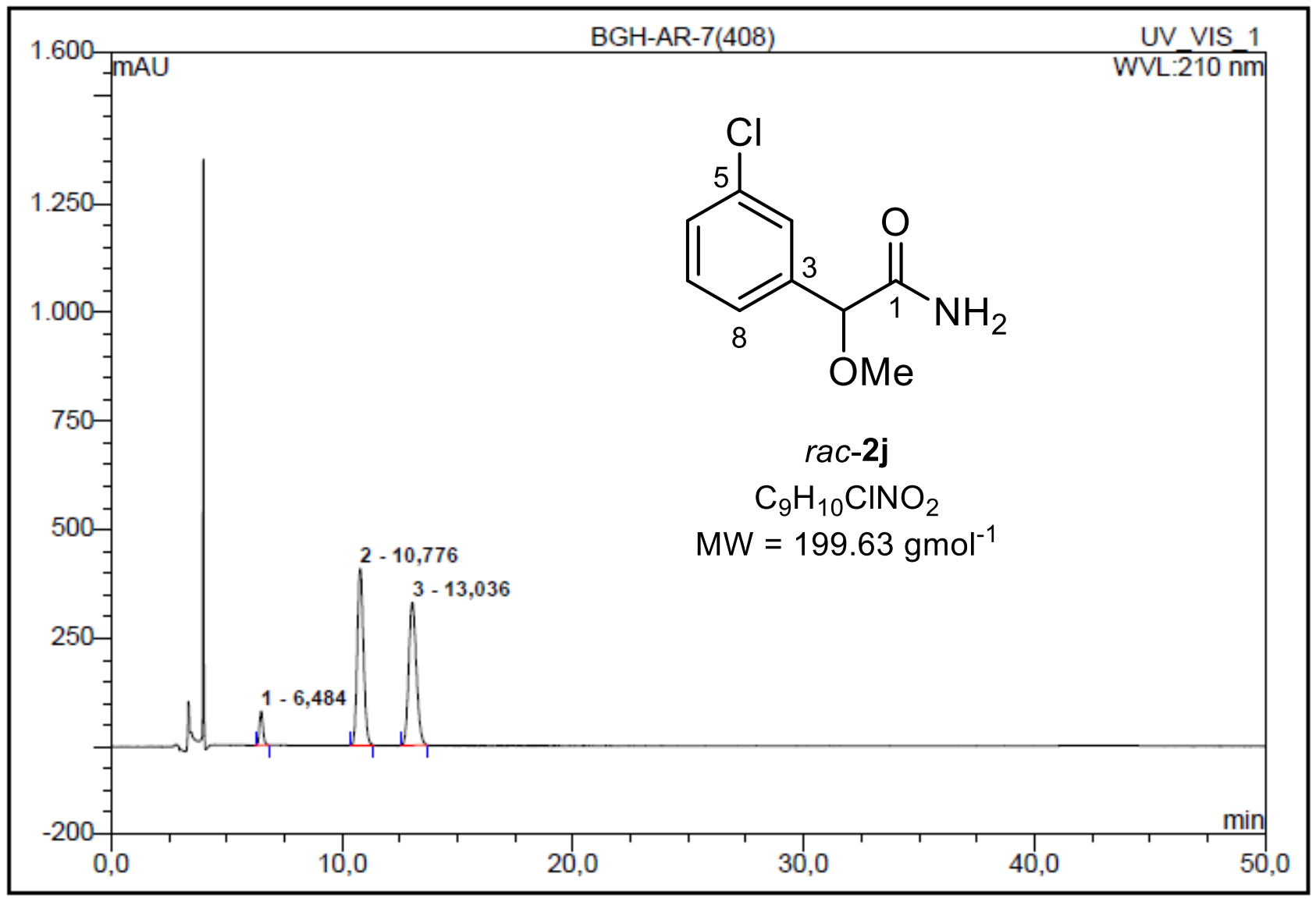

No.	Ret.Time min	Peak Name	Height mAU	Area mAU*min	Rel.Area %	Amount	Type
1	6,48	n.a.	78,233	13,998	5,21	n.a.	BMB
2	10,78	n.a.	407,687	127,604	47,50	n.a.	BMB
3	13,04	n.a.	328,207	127,054	47,29	n.a.	BMB
Total:			814,127	268,656	100,00	0,000	

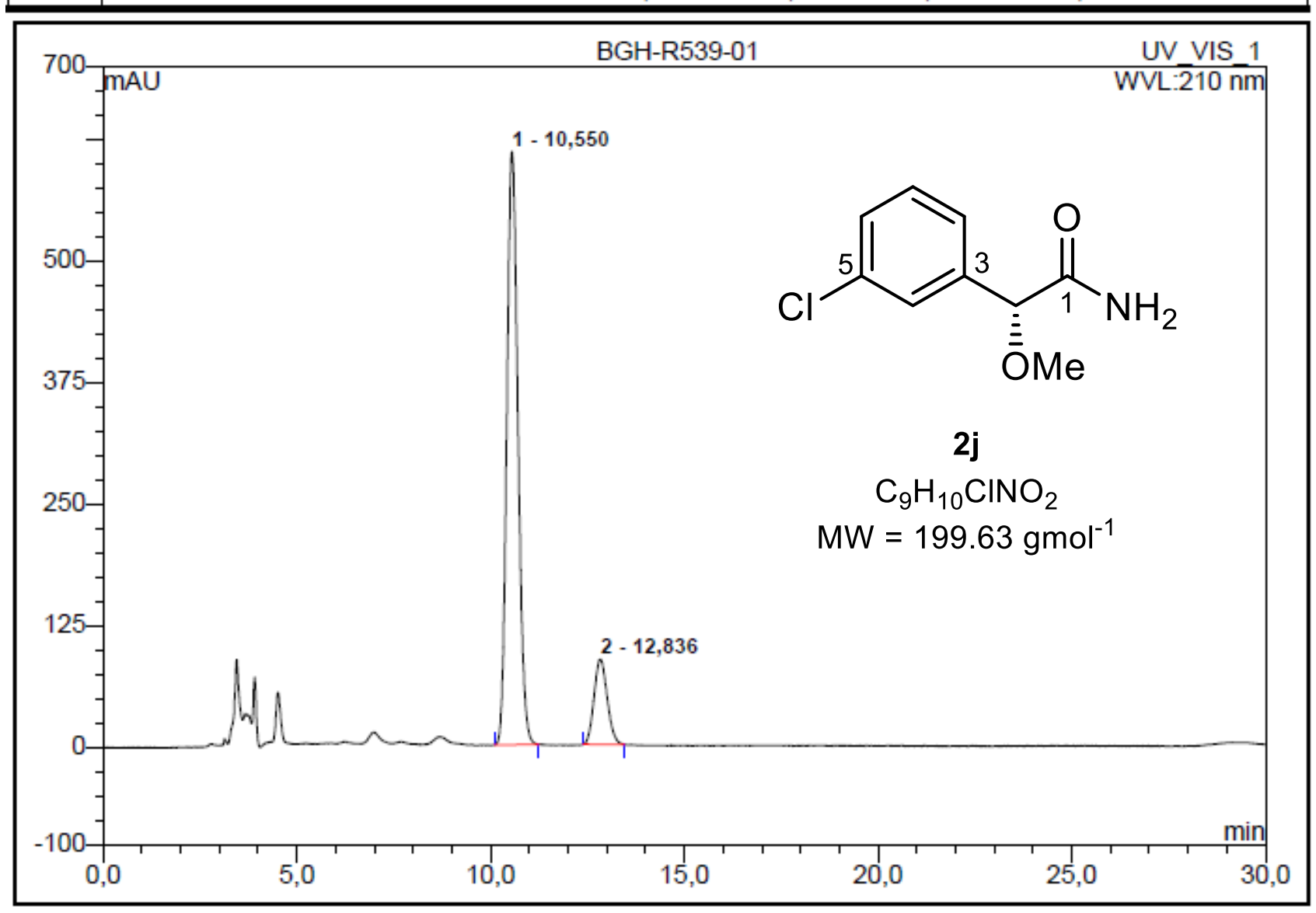

No.	Ret.Time min	Peak Name	Height mAU	Area mAU*min	Rel.Area %	Amount	Type
1	10,55	n.a.	608,851	201,732	85,41	n.a.	BMB
2	12,84	n.a.	87,669	34,447	14,59	n.a.	BMB
Total:			696,520	236,178	100,00	0,000	

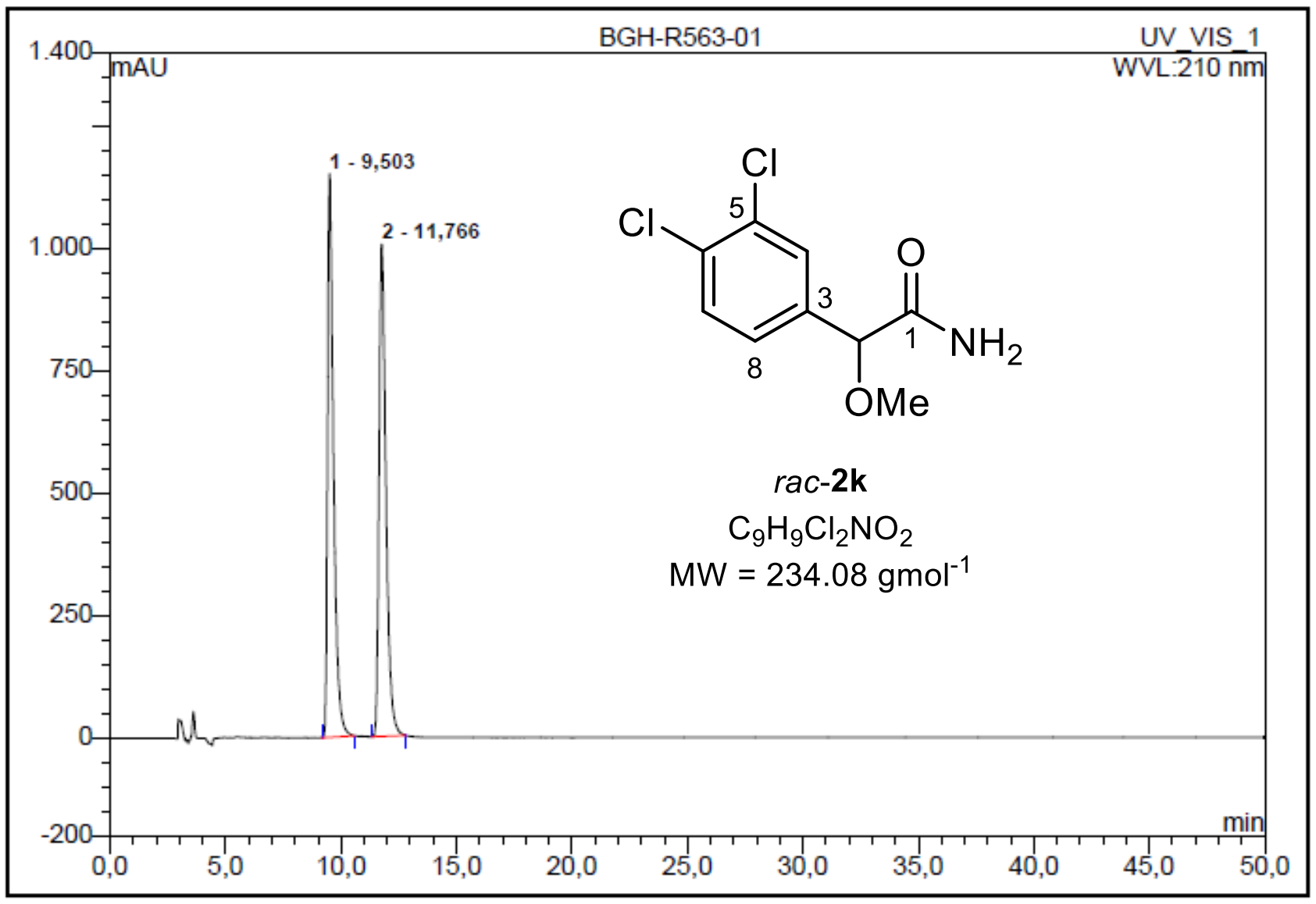

No.	Ret.Time min	Peak Name	Height mAU	Area mAU*min	Rel.Area %	Amount	Type
1	9,50	n.a.	1149,238	348,562	49,87	n.a.	BMB
2	11,77	n.a.	1003,984	350,413	50,13	n.a.	BMB
Total:			2153,222	698,975	100,00	0,000	

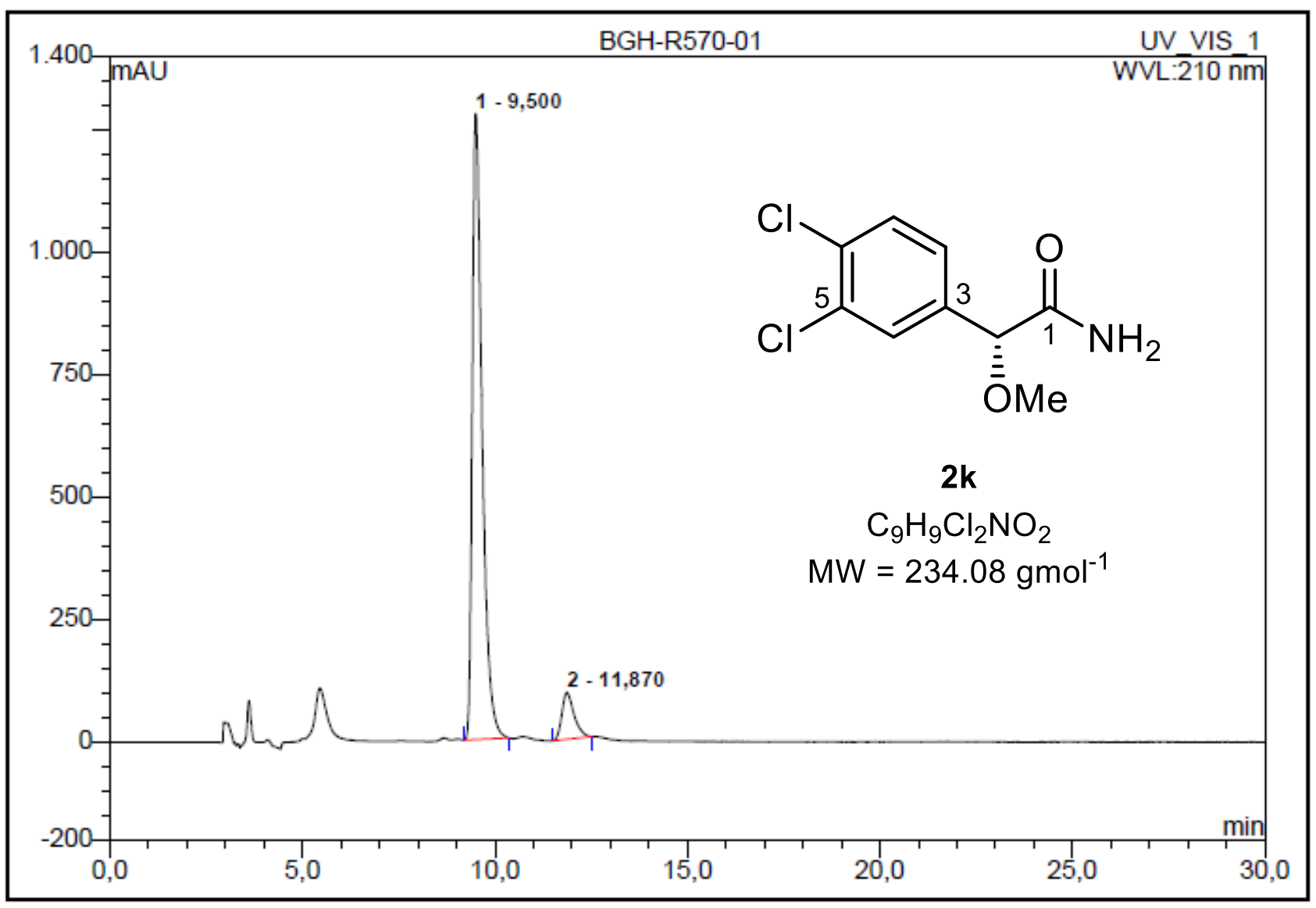

No.	Ret.Time min	Peak Name	Height mAU	Area mAU*min	Rel.Area %	Amount	Type
1	9,50	n.a.	1276,819	376,370	91,45	n.a.	BMB
2	11,87	n.a.	95,821	35,182	8,55	n.a.	BMB
Total:			1372,640	411,553	100,00	0,000	

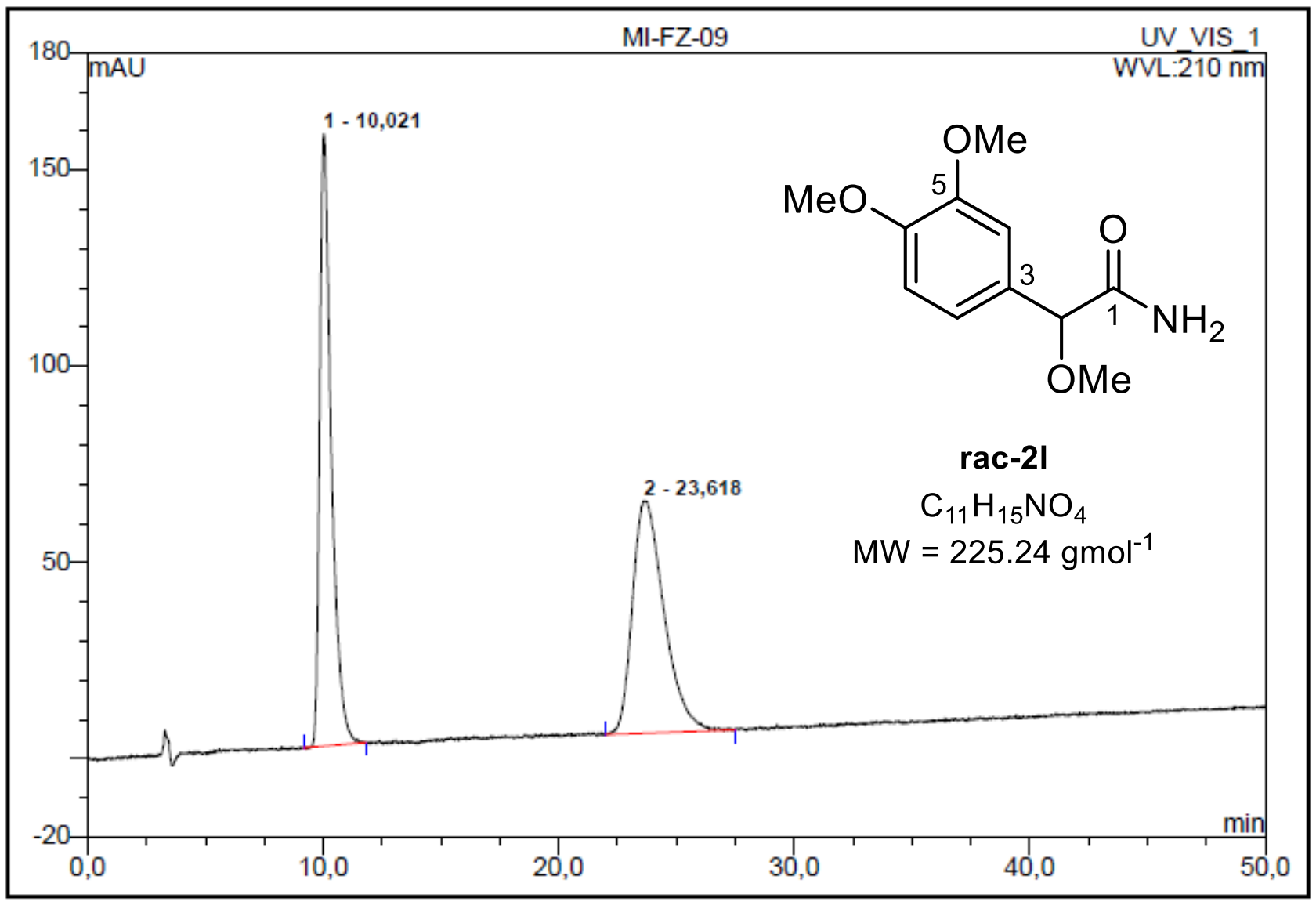

No.	Ret.Time min	Peak Name	Height mAU	Area mAU*min	Rel.Area %	Amount	Type
1	10,02	n.a.	155,897	88,983	49,18	n.a.	BMB*
2	23,62	n.a.	59,200	91,957	50,82	n.a.	BMB*
Total:			215,097	180,940	100,00	0,000	

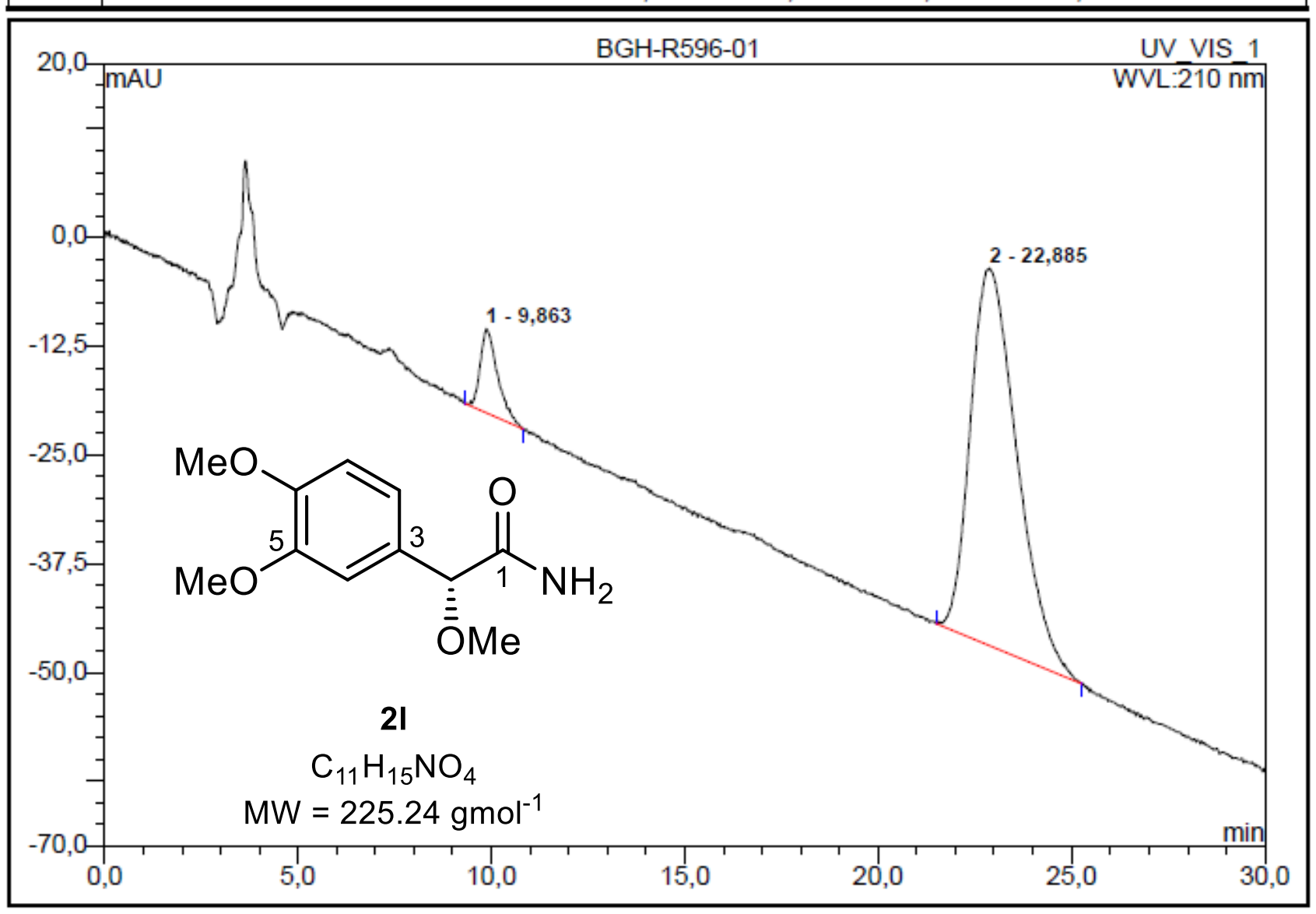

No.	Ret.Time min	Peak Name	Height mAU	Area mAU*min	Rel.Area %	Amount	Type
1	9,86	n.a.	9,684	5,071	7,72	n.a.	BMB*
2	22,88	n.a.	43,405	60,623	92,28	n.a.	BMB*
Total:			53,089	65,694	100,00	0,000	

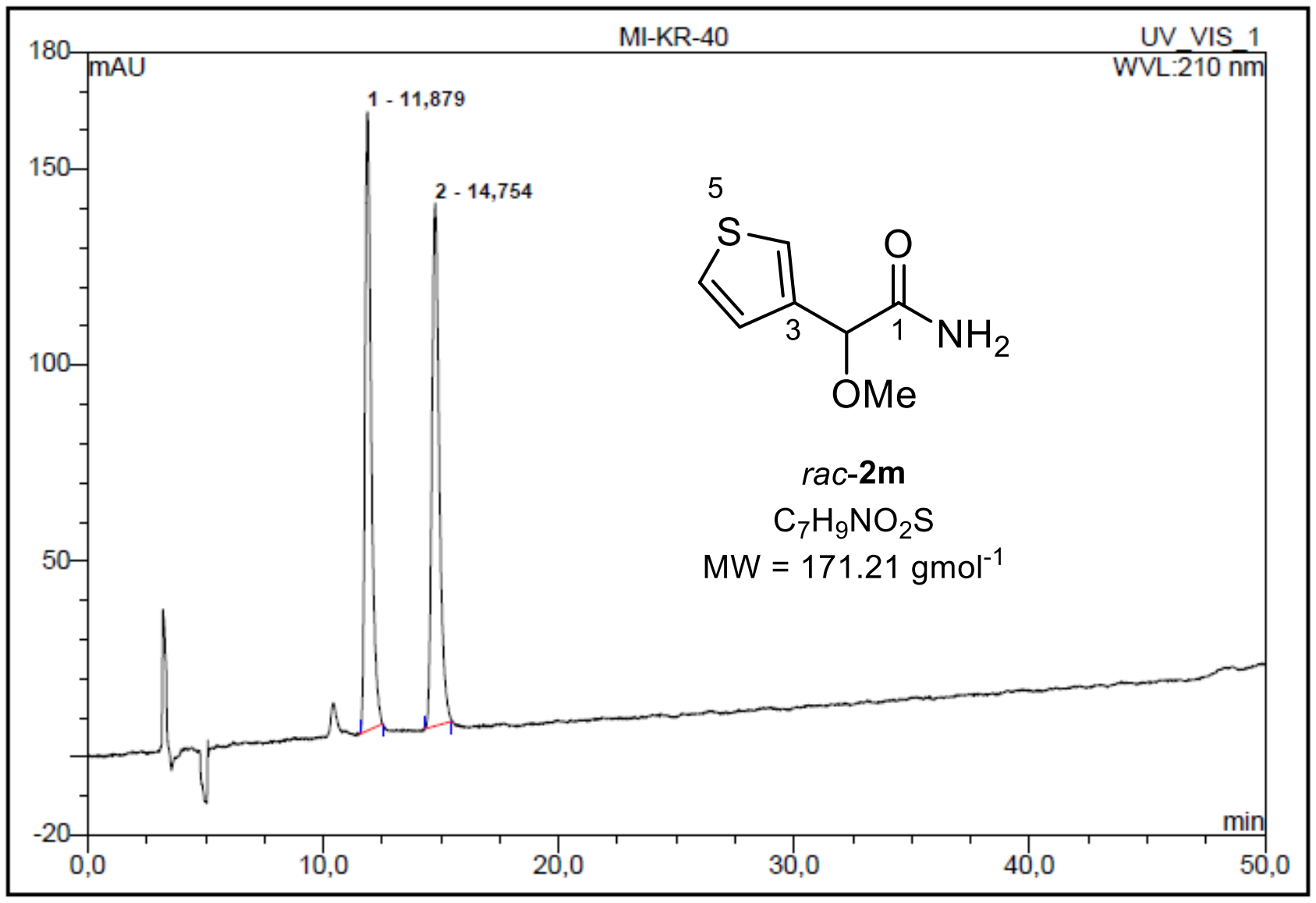

No.	Ret.Time min	Peak Name	Height mAU	Area mAU*min	Rel.Area %	Amount	Type
1	11,88	n.a.	157,883	48,104	49,94	n.a.	BMB
2	14,75	n.a.	133,360	48,215	50,06	n.a.	BMB
Total:			291,243	96,319	100,00	0,000	

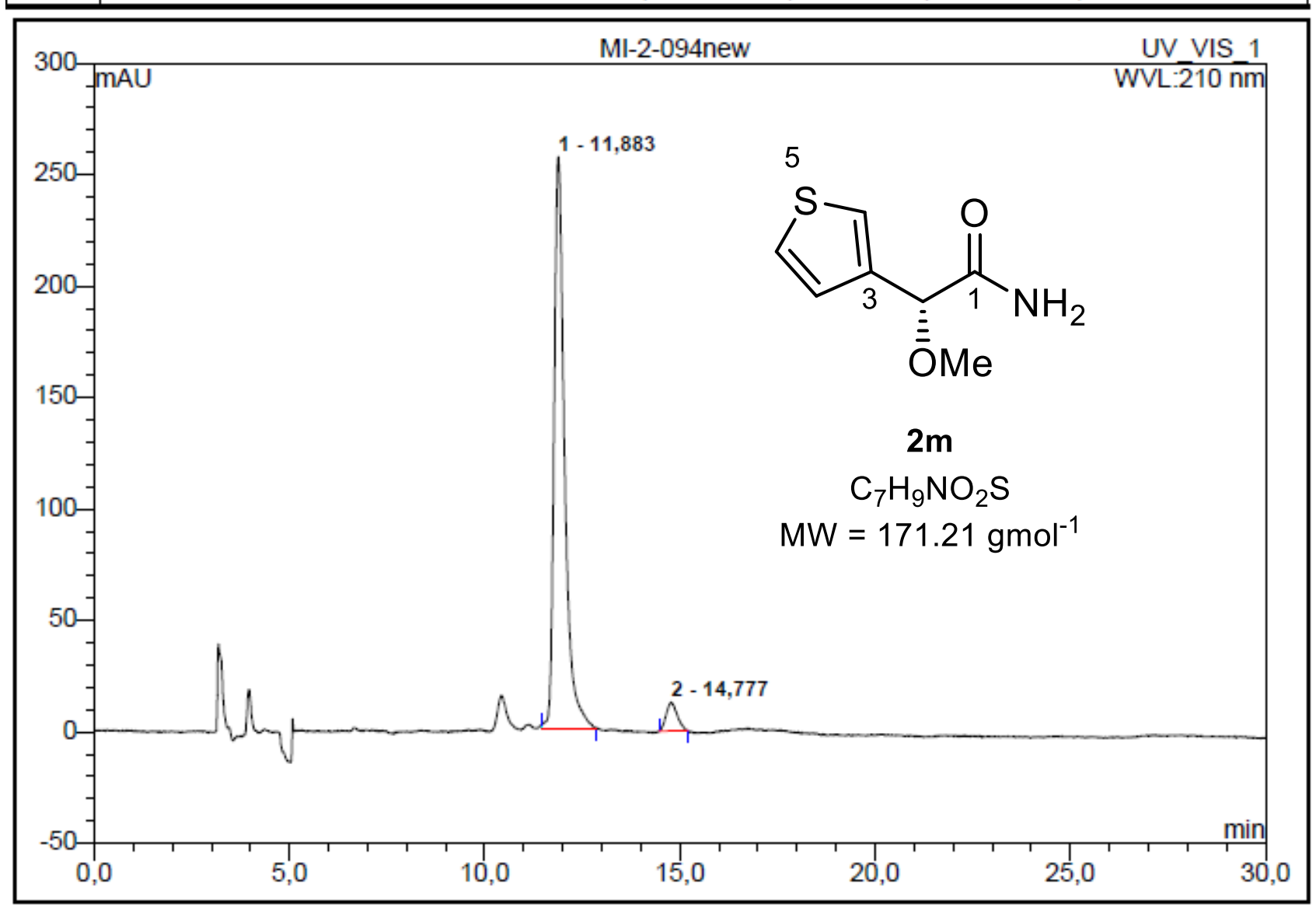

No.	Ret.Time min	Peak Name	Height mAU	Area mAU*min	Rel.Area %	Amount	Type
1	11,88	n.a.	256,392	79,673	95,07	n.a.	MB*
2	14,78	n.a.	12,735	4,136	4,93	n.a.	BMB*
Total:			269,127	83,809	100,00	0,000	

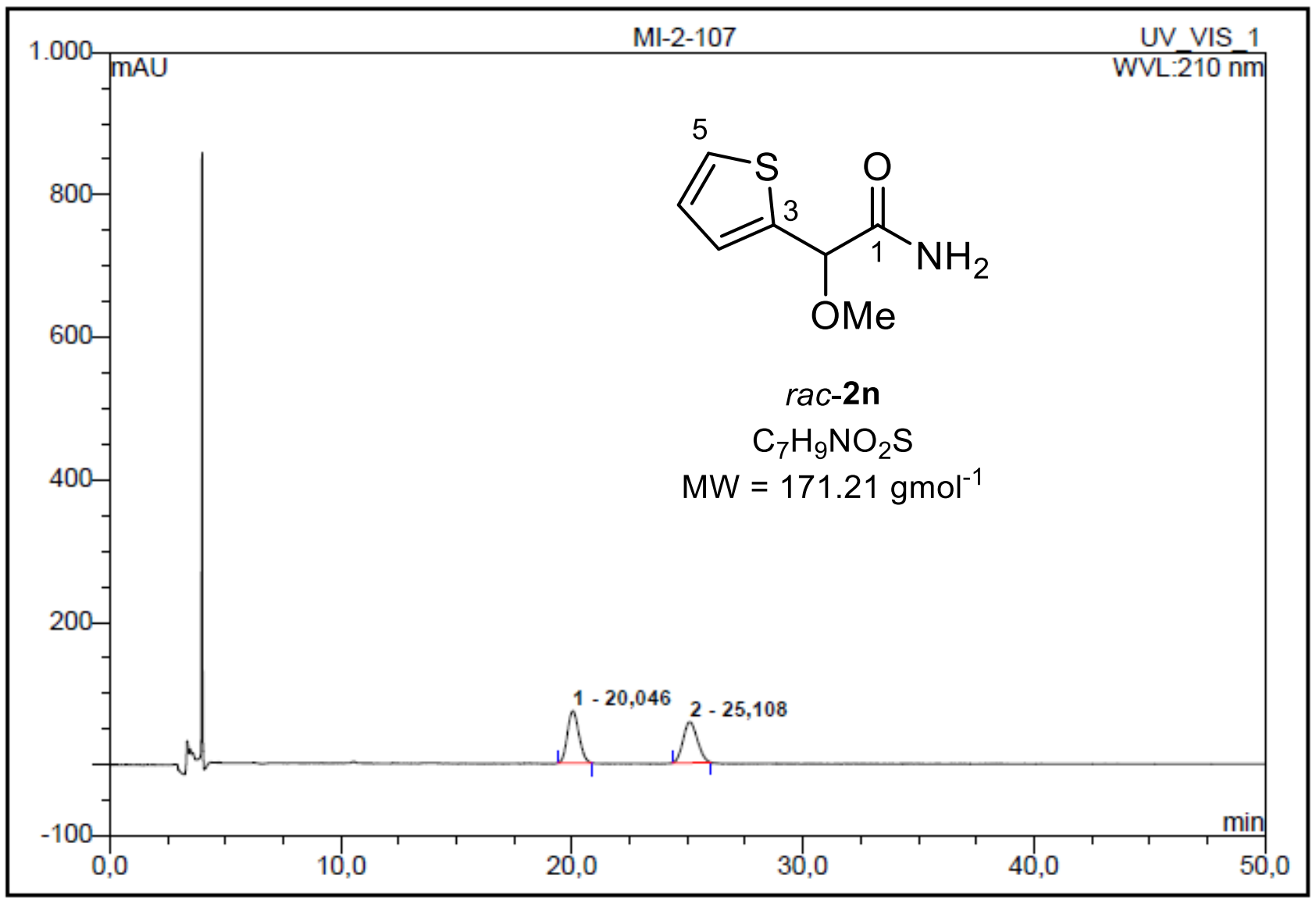

No.	Ret.Time min	Peak Name	Height mAU	Area mAU*min	Rel.Area %	Amount	Type
1	20,05	n.a.	73,281	42,036	50,67	n.a.	BMB
2	25,11	n.a.	57,122	40,919	49,33	n.a.	BMB
Total:			130,403	82,955	100,00	0,000	

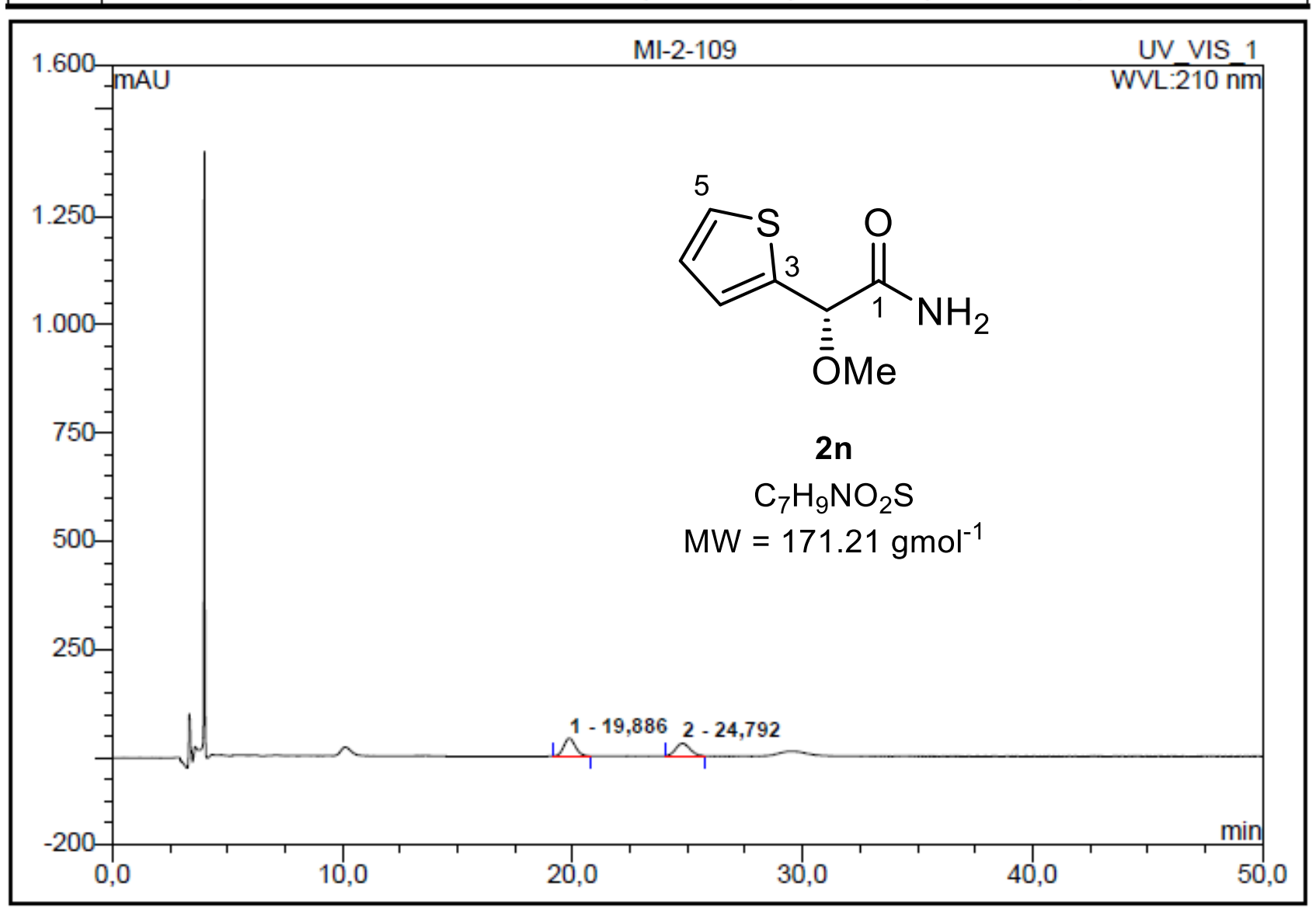

No.	Ret.Time min	Peak Name	Height mAU	Area mAU*min	Rel.Area %	Amount	Type
1	19,89	n.a.	40,679	23,430	52,62	n.a.	BMB*
2	24,79	n.a.	29,542	21,095	47,38	n.a.	BMB*
Total:			70,221	44,525	100,00	0,000	

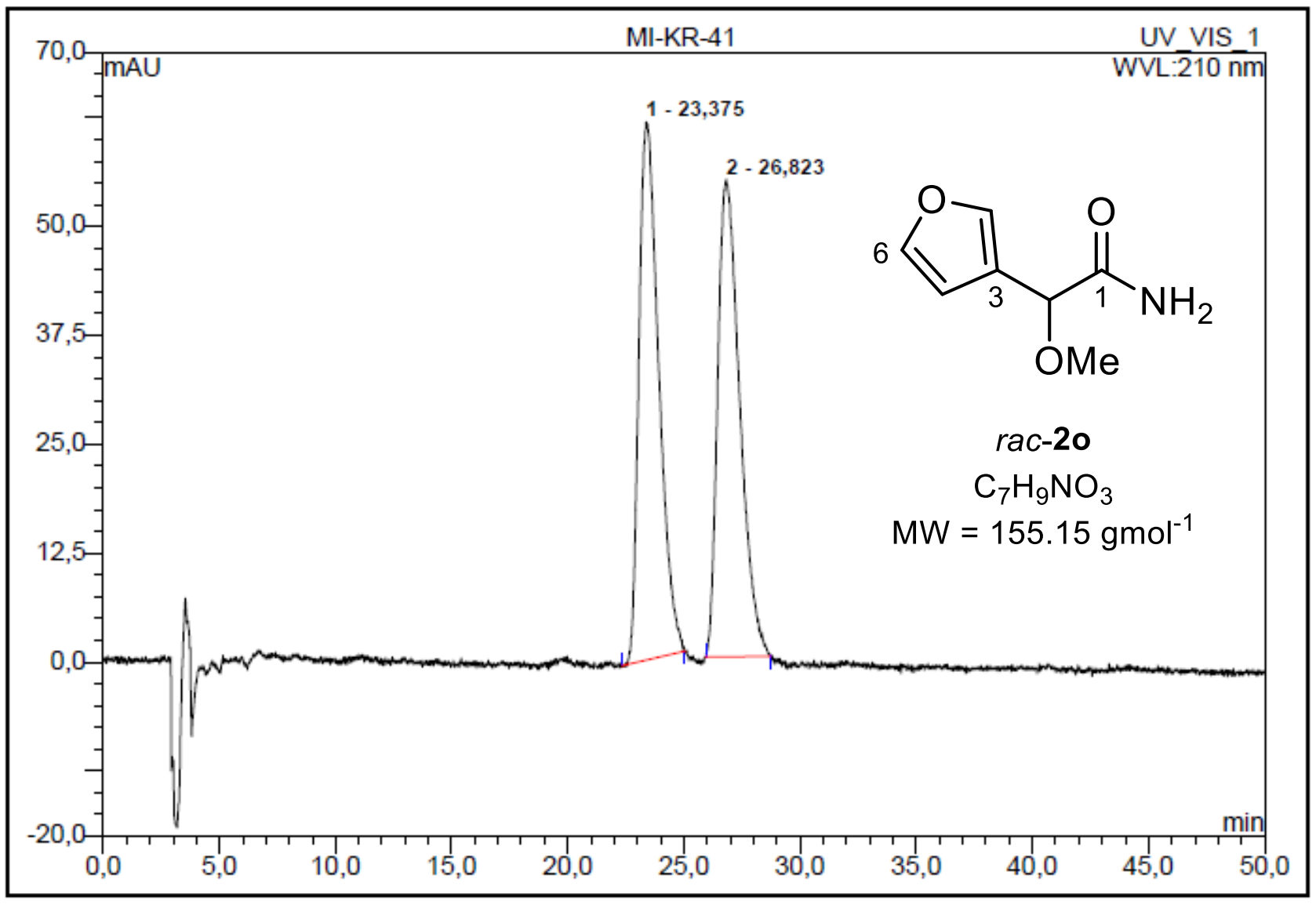

No.	Ret.Time min	Peak Name	Height mAU	Area mAU*min	Rel.Area %	Amount	Type
1	23,37	n.a.	61,779	61,112	50,36	n.a.	BMB*
2	26,82	n.a.	54,792	60,239	49,64	n.a.	BMB*
Total:			116,571	121,351	100,00	0,000	

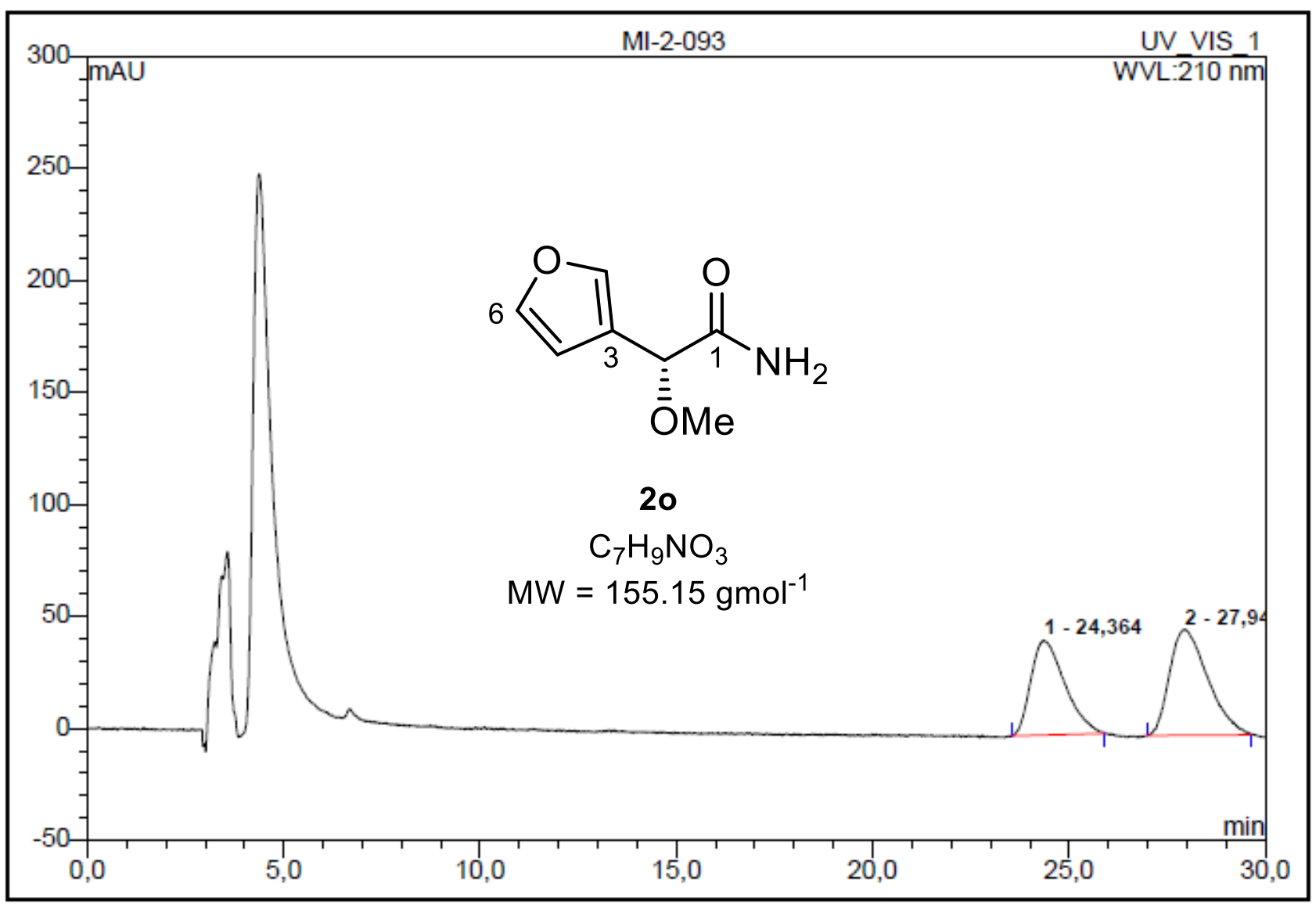

No.	Ret.Time min	Peak Name	Height mAU	Area mAU*min	Rel.Area %	Amount	Type
1	24,36	n.a.	42,238	42,415	44,32	n.a.	BMB*
2	27,95	n.a.	47,318	53,279	55,68	n.a.	BMB*
Total:			89,556	95,694	100,00	0,000	

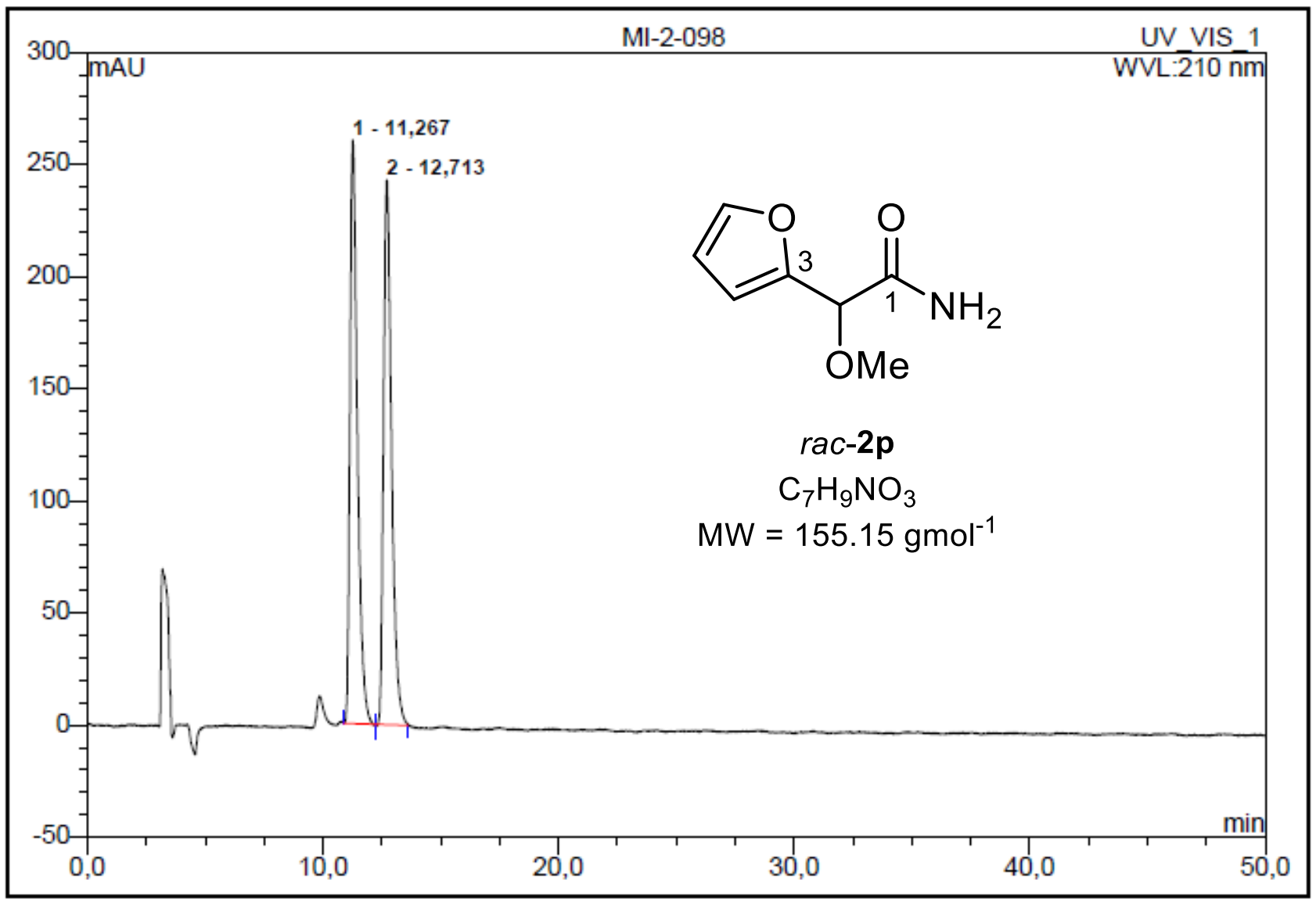

No.	Ret.Time min	Peak Name	Height mAU	Area mAU*min	Rel.Area %	Amount	Type
1	11,27	n.a.	260,167	94,585	49,82	n.a.	BM
2	12,71	n.a.	242,883	95,273	50,18	n.a.	MB
Total:			503,050	189,858	100,00	0,000	

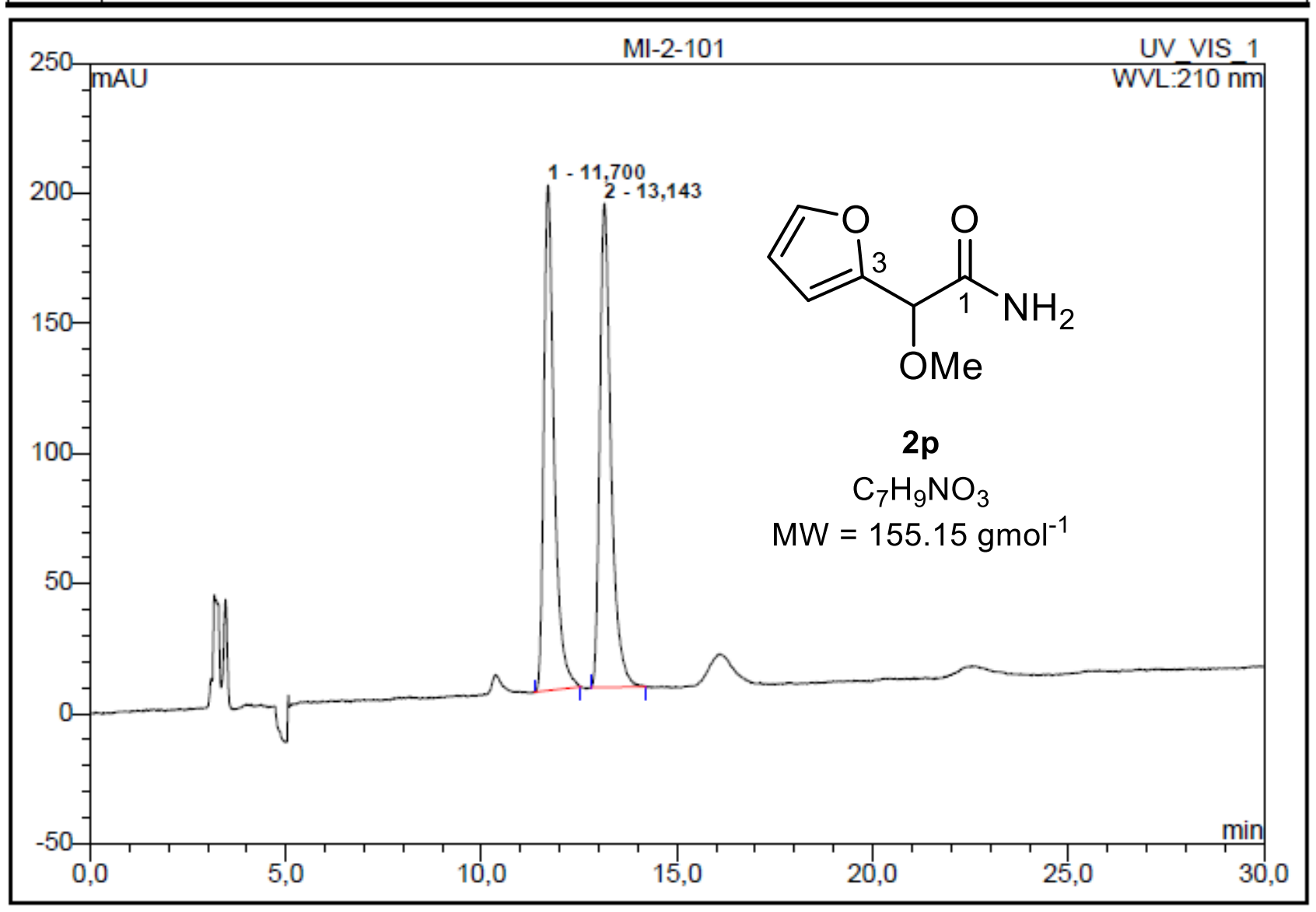

No.	Ret.Time min	Peak Name	Height mAU	Area mAU*min	Rel.Area %	Amount	Type
1	11,70	n.a.	194,134	59,267	48,87	n.a.	BMB*
2	13,14	n.a.	185,897	62,012	51,13	n.a.	BMB*
Total:			380,030	121,279	100,00	0,000	

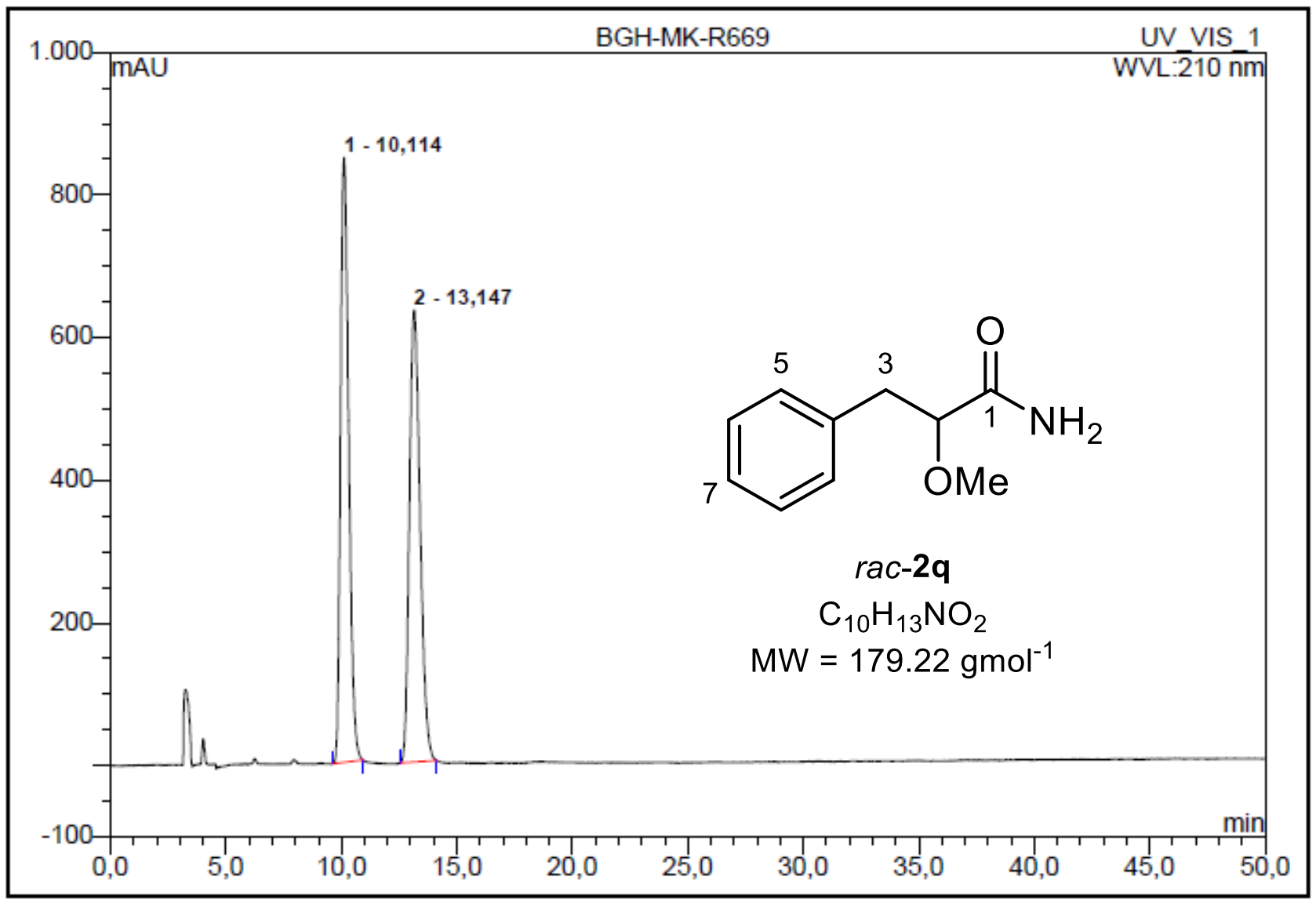

No.	Ret.Time min	Peak Name	Height mAU	Area mAU*min	Rel.Area %	Amount	Type
1	10,11	n.a.	847,864	349,330	50,68	n.a.	BMB
2	13,15	n.a.	633,628	340,002	49,32	n.a.	BMB
Total:			1481,492	689,332	100,00	0,000	

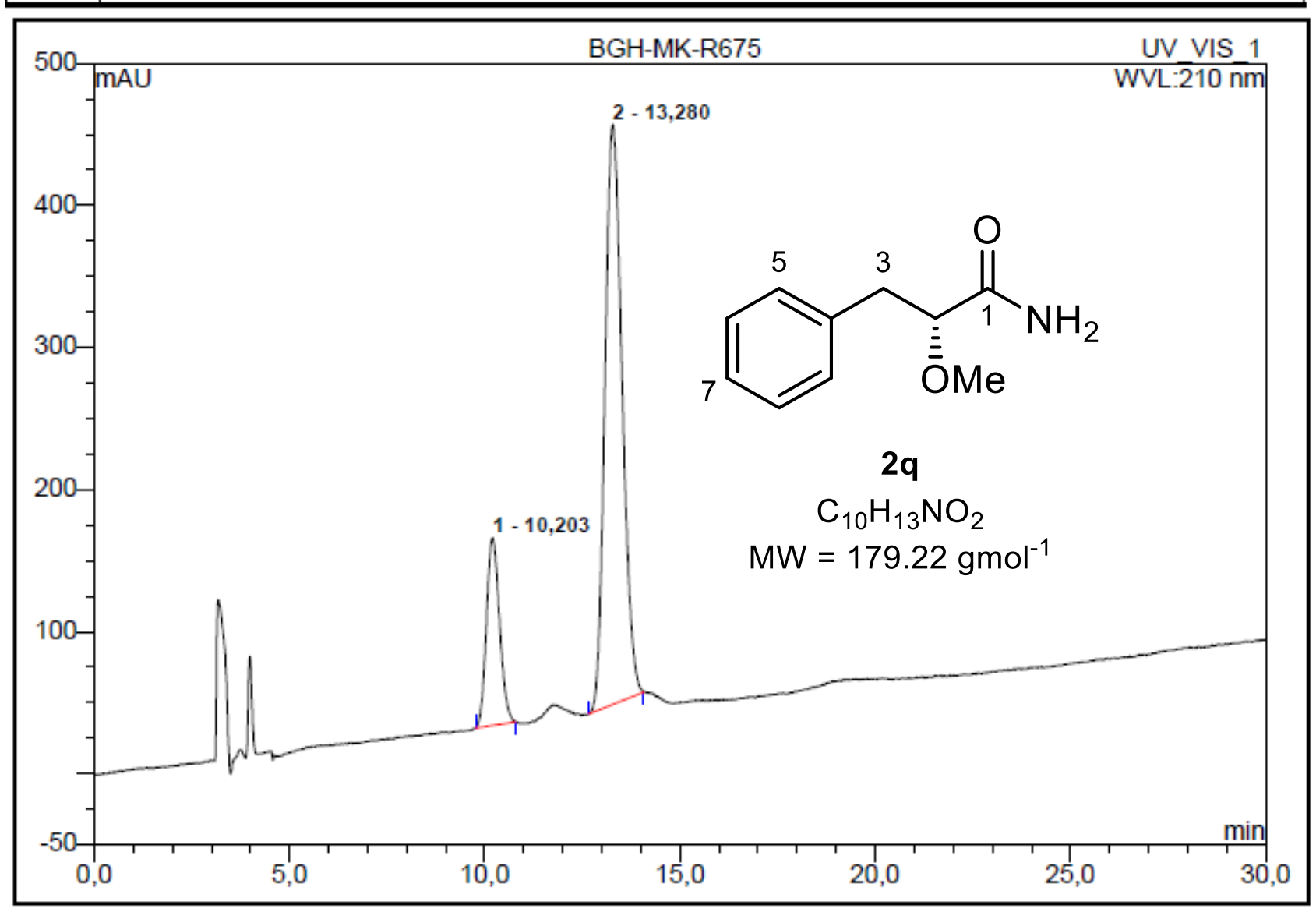

No.	Ret.Time min	Peak Name	Height mAU	Area mAU*min	Rel.Area %	Amount	Type
1	10,20	n.a.	132,295	51,525	19,87	n.a.	BMB
2	13,28	n.a.	408,207	207,749	80,13	n.a.	BMB
Total:			540,503	259,274	100,00	0,000	

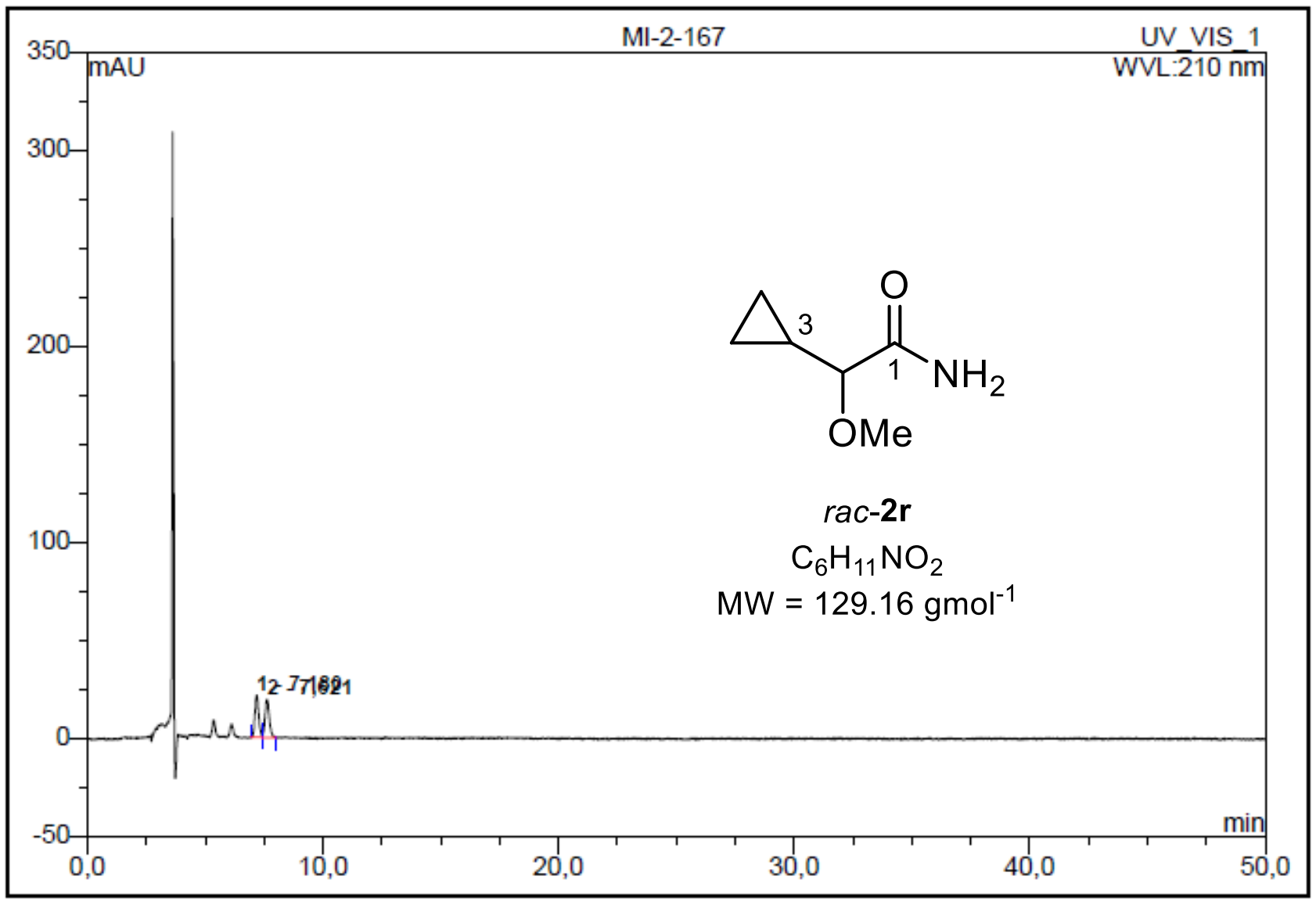

No.	Ret.Time min	Peak Name	Height mAU	Area mAU*min	Rel.Area %	Amount	Type
1	7,18	n.a.	21,326	3,992	49,21	n.a.	BM *
2	7,62	n.a.	19,446	4,120	50,79	n.a.	MB*
Total:			40,772	8,112	100,00	0,000	

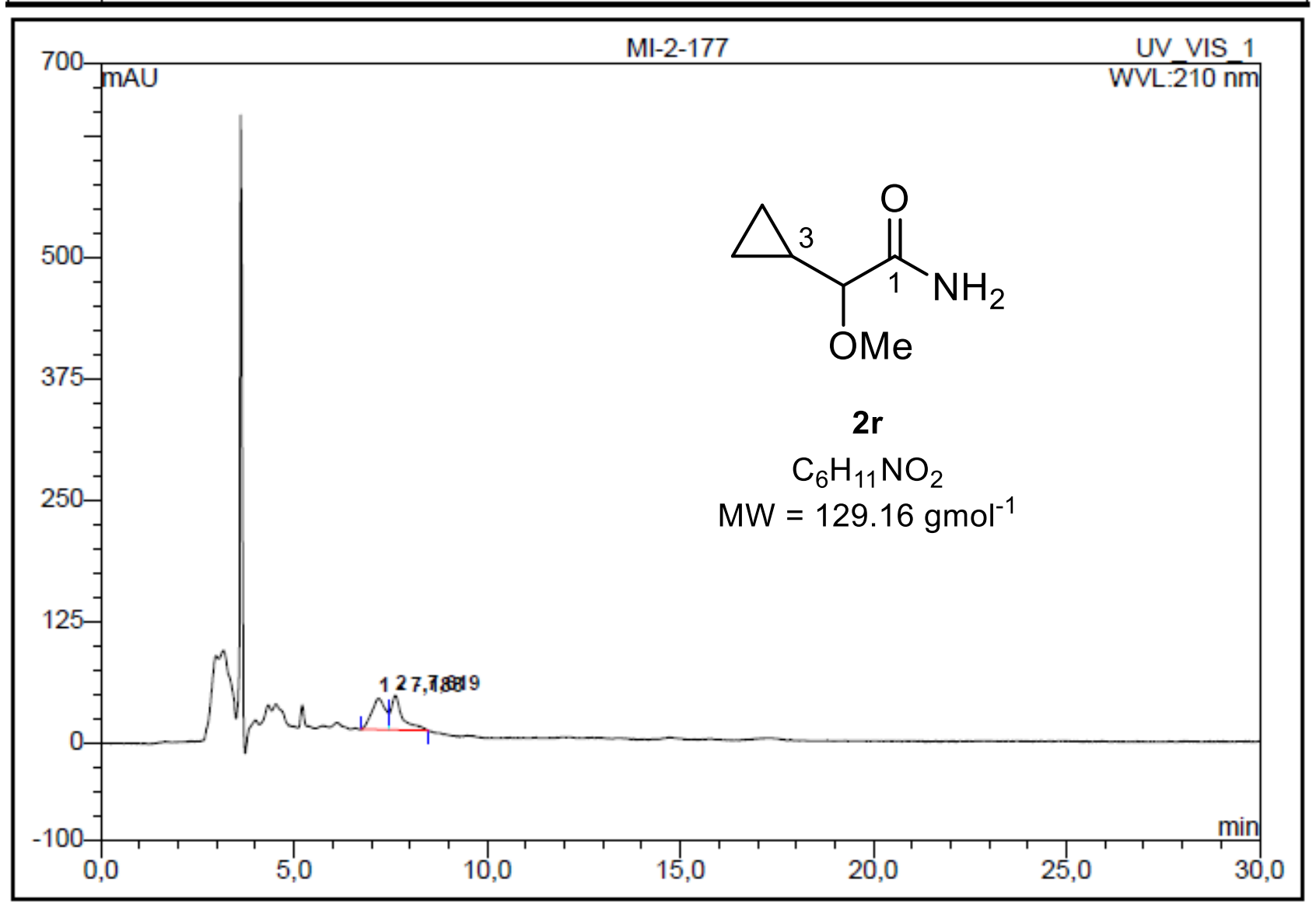

No.	Ret.Time min	Peak Name	Height mAU	Area mAU*min	Rel.Area %	Amount	Type
1	7,19	n.a.	32,287	12,975	51,12	n.a.	BM *
2	7,62	n.a.	35,462	12,405	48,88	n.a.	MB*
Total:			67,749	25,380	100,00	0,000	

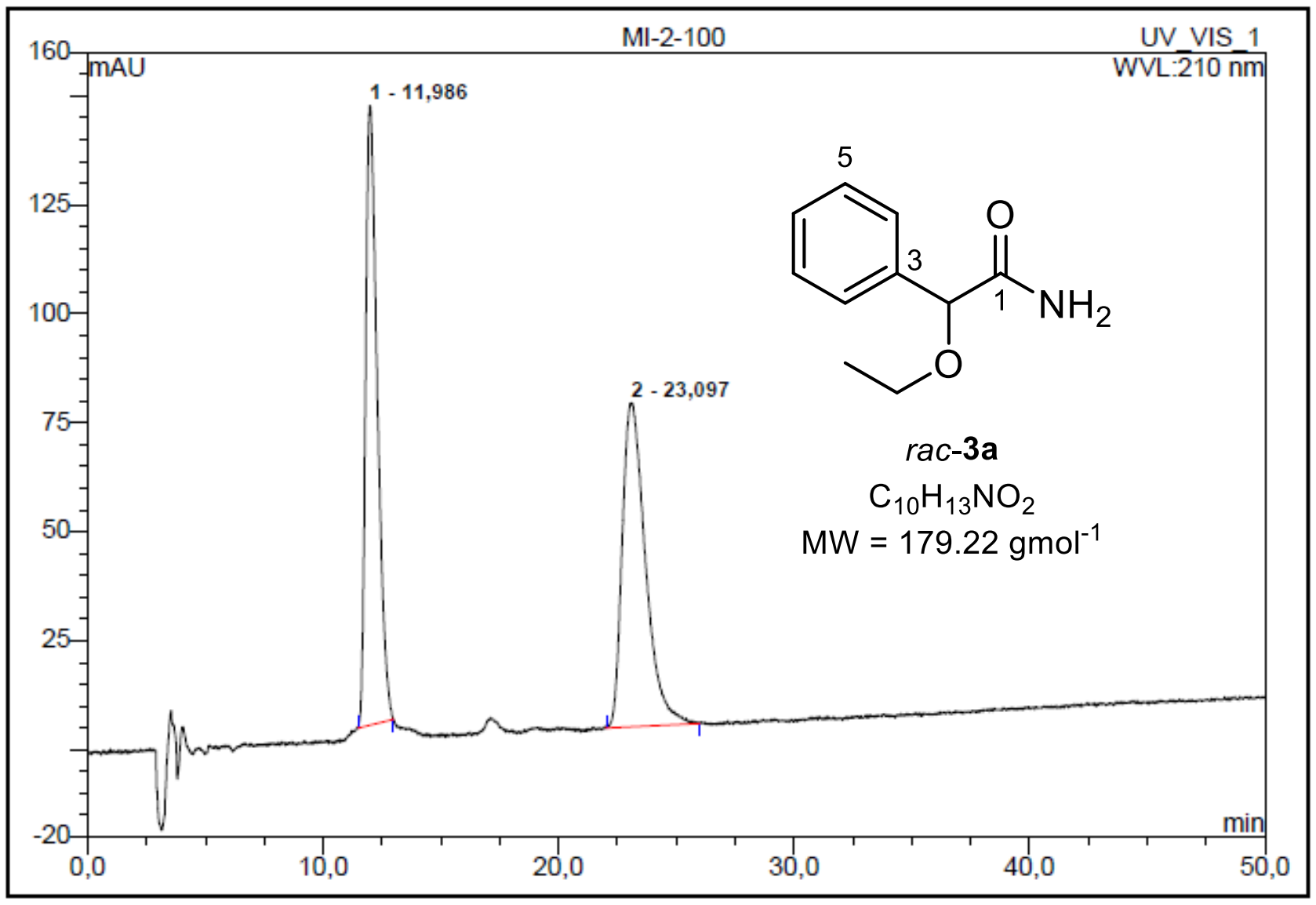

No.	Ret.Time min	Peak Name	Height mAU	Area mAU*min	Rel.Area %	Amount	Type
1	11,99	n.a.	142,117	82,096	49,09	n.a.	BMB
2	23,10	n.a.	74,357	85,130	50,91	n.a.	BMB*
Total:			216,474	167,226	100,00	0,000	

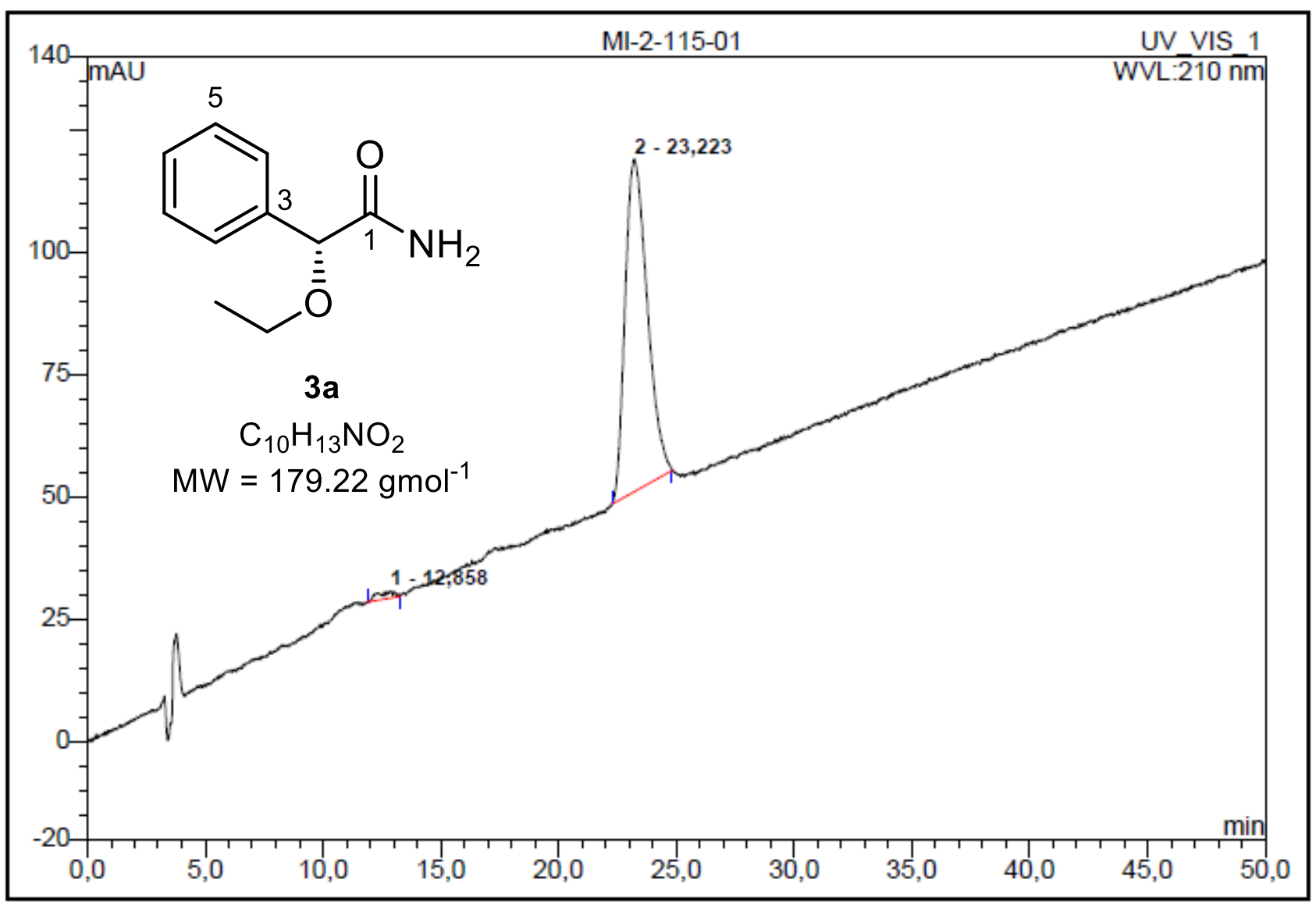

No.	Ret.Time min	Peak Name	Height mAU	Area mAU*min	Rel.Area %	Amount	Type
1	12,86	n.a.	1,407	1,194	1,59	n.a.	BMB*
2	23,22	n.a.	67,935	73,857	98,41	n.a.	BMB
Total:			69,342	75,052	100,00	0,000	

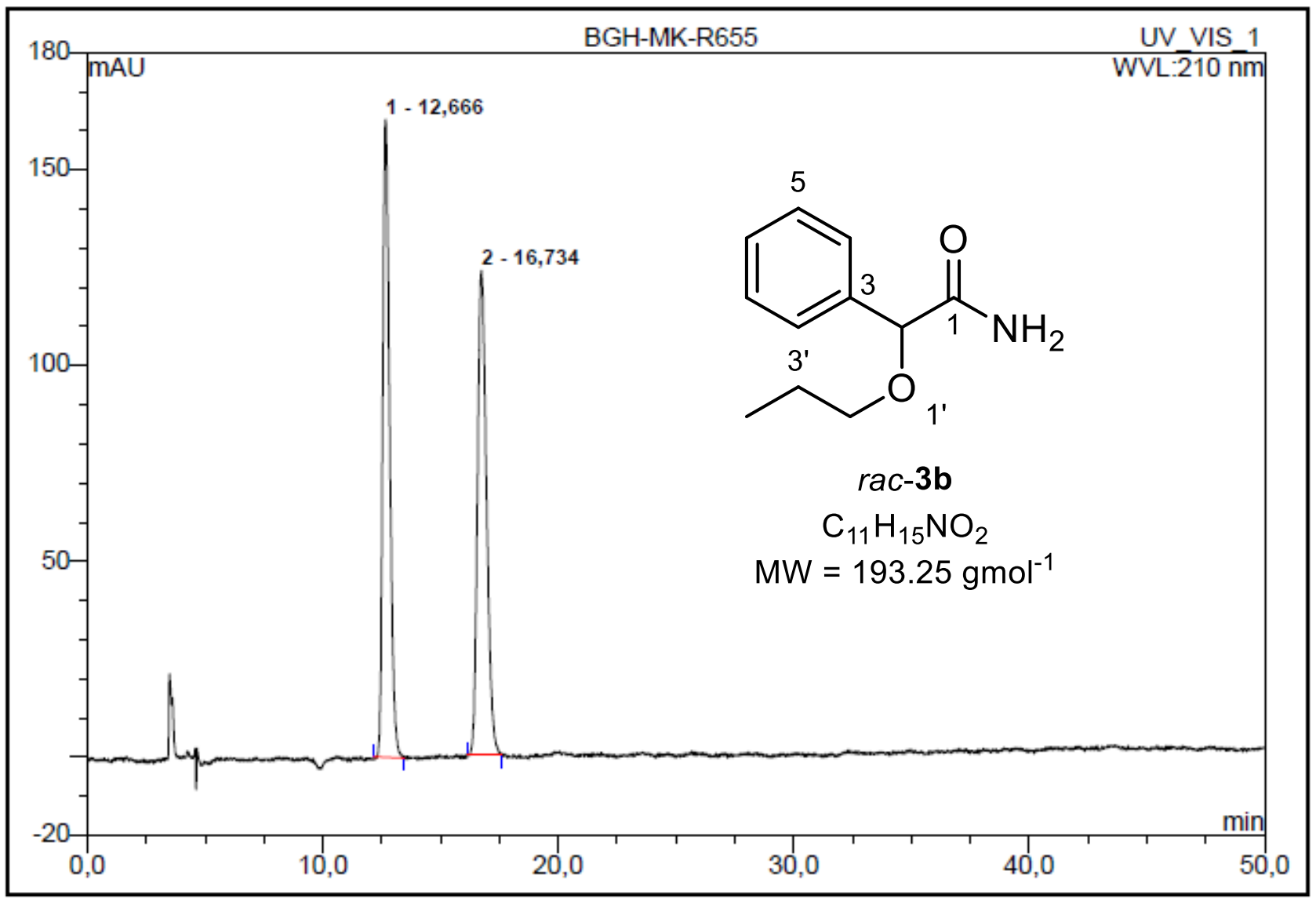

No.	Ret.Time min	Peak Name	Height mAU	Area mAU*min	Rel.Area %	Amount	Type
1	12,67	n.a.	162,886	55,020	49,84	n.a.	BMB*
2	16,73	n.a.	123,816	55,367	50,16	n.a.	BMB*
Total:			286,702	110,387	100,00	0,000	

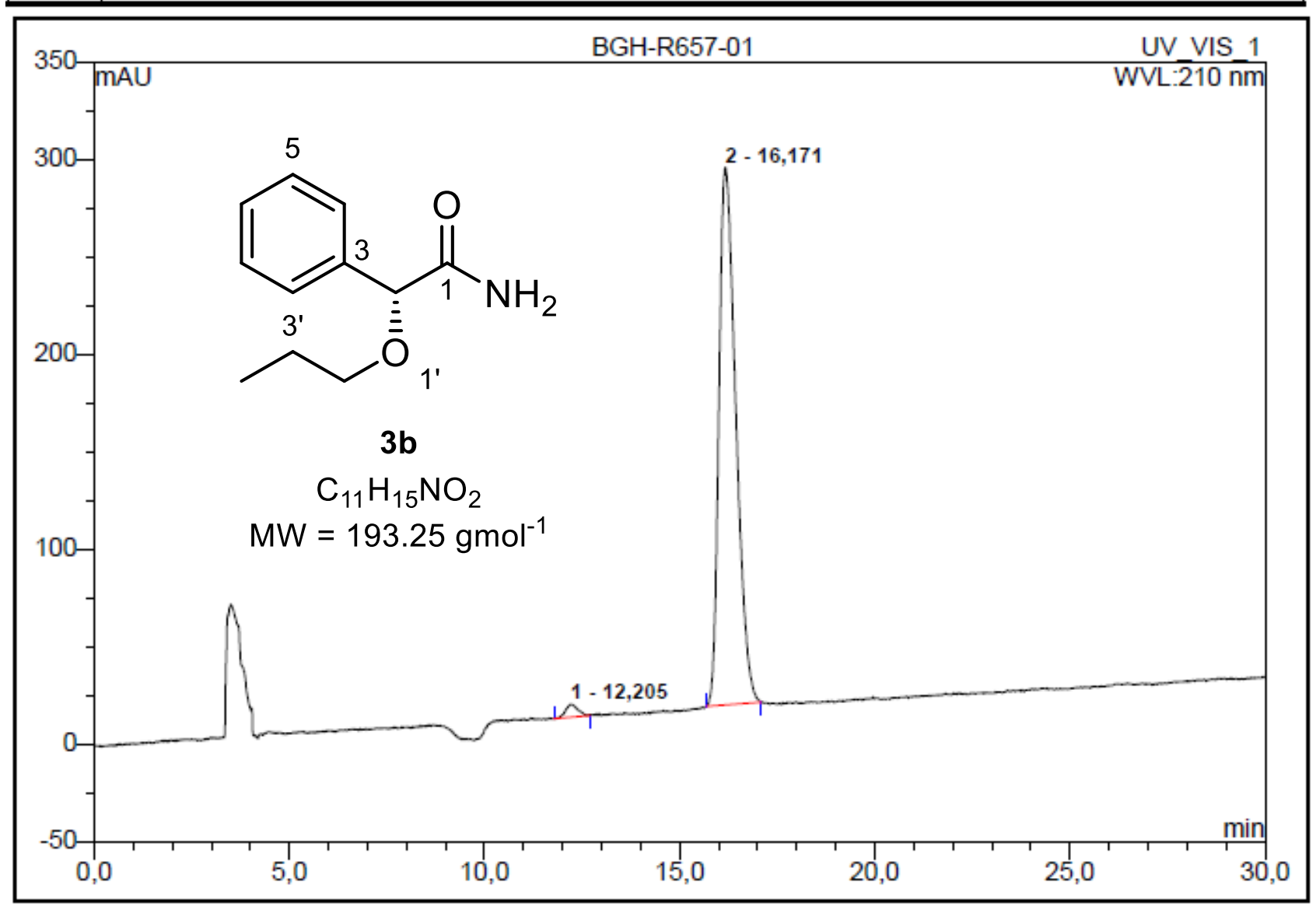

No.	Ret.Time min	Peak Name	Height mAU	Area mAU*min	Rel.Area %	Amount	Type
1	12,21	n.a.	6,572	2,386	1,70	n.a.	BMB*
2	16,17	n.a.	275,525	137,867	98,30	n.a.	BMB
Total:			282,097	140,253	100,00	0,000	

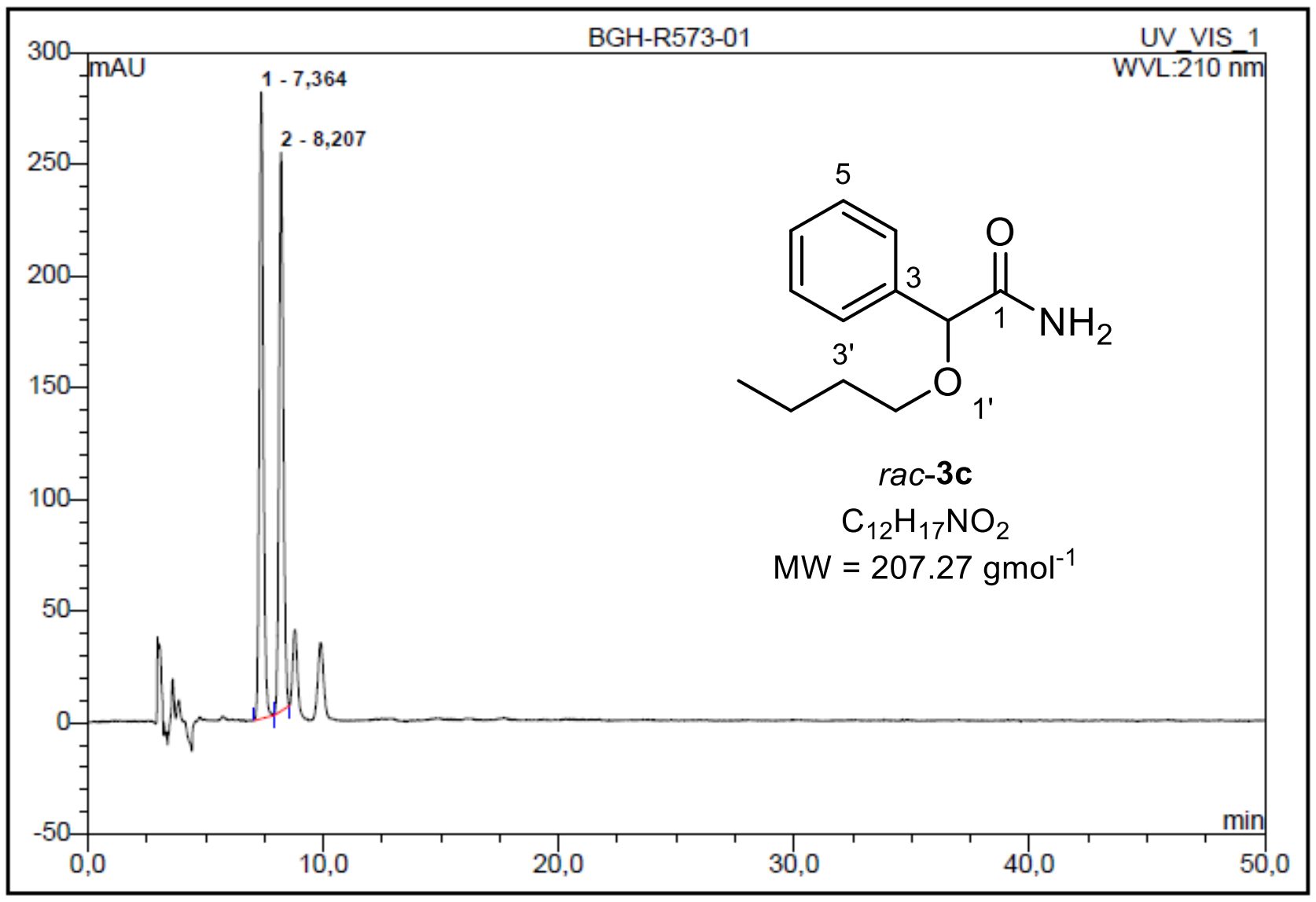

No.	Ret.Time min	Peak Name	Height mAU	Area mAU*min	Rel.Area %	Amount	Type
1	7,36	n.a.	280,649	56,111	50,98	n.a.	BMB*
2	8,21	n.a.	249,993	53,950	49,02	n.a.	BMB*
Total:			530,641	110,061	100,00	0,000	

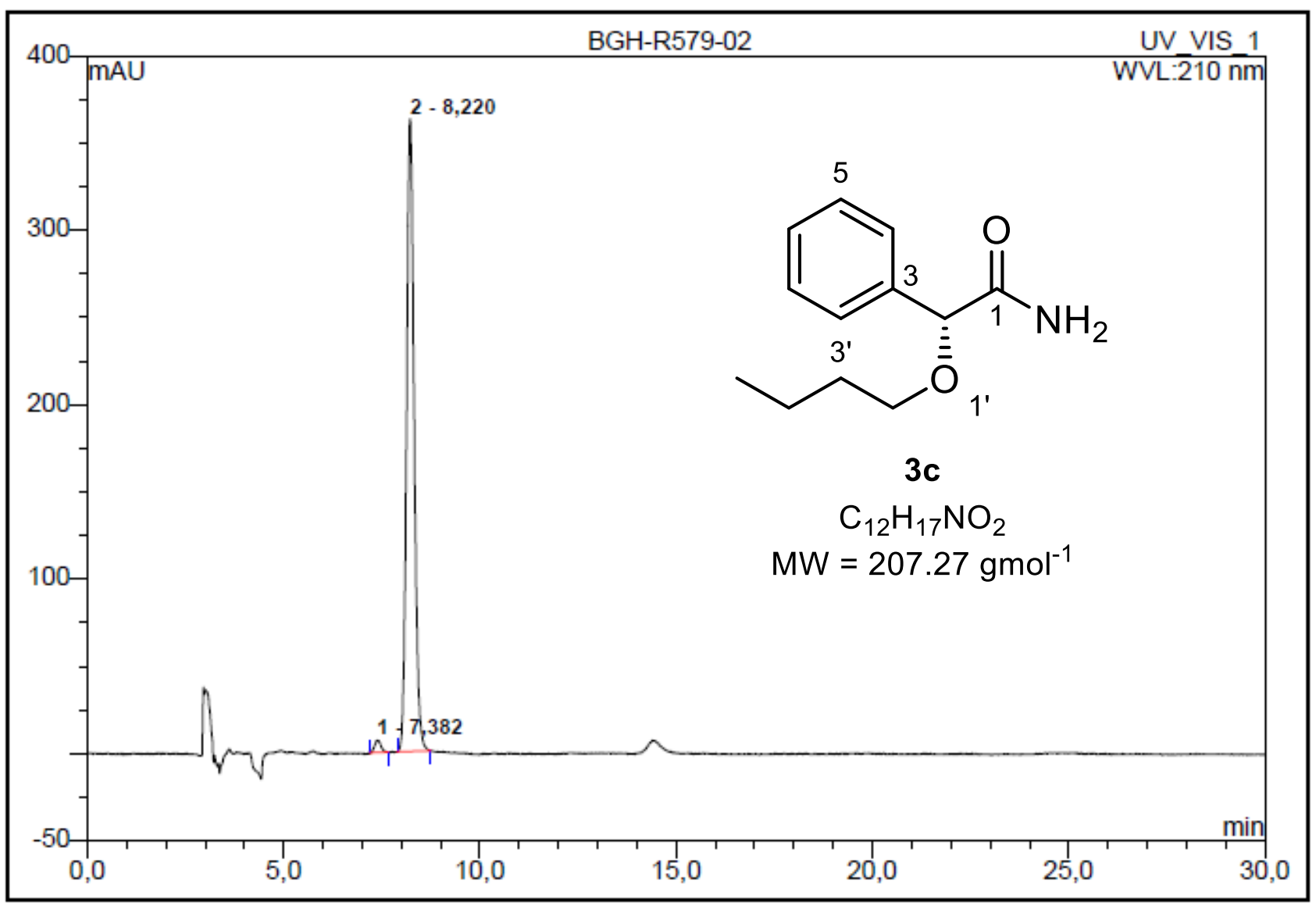

No.	Ret.Time min	Peak Name	Height mAU	Area mAU*min	Rel.Area %	Amount	Type
1	7,38	n.a.	7,014	1,329	1,62	n.a.	BMB*
2	8,22	n.a.	362,539	80,730	98,38	n.a.	BMB*
Total:			369,553	82,059	100,00	0,000	

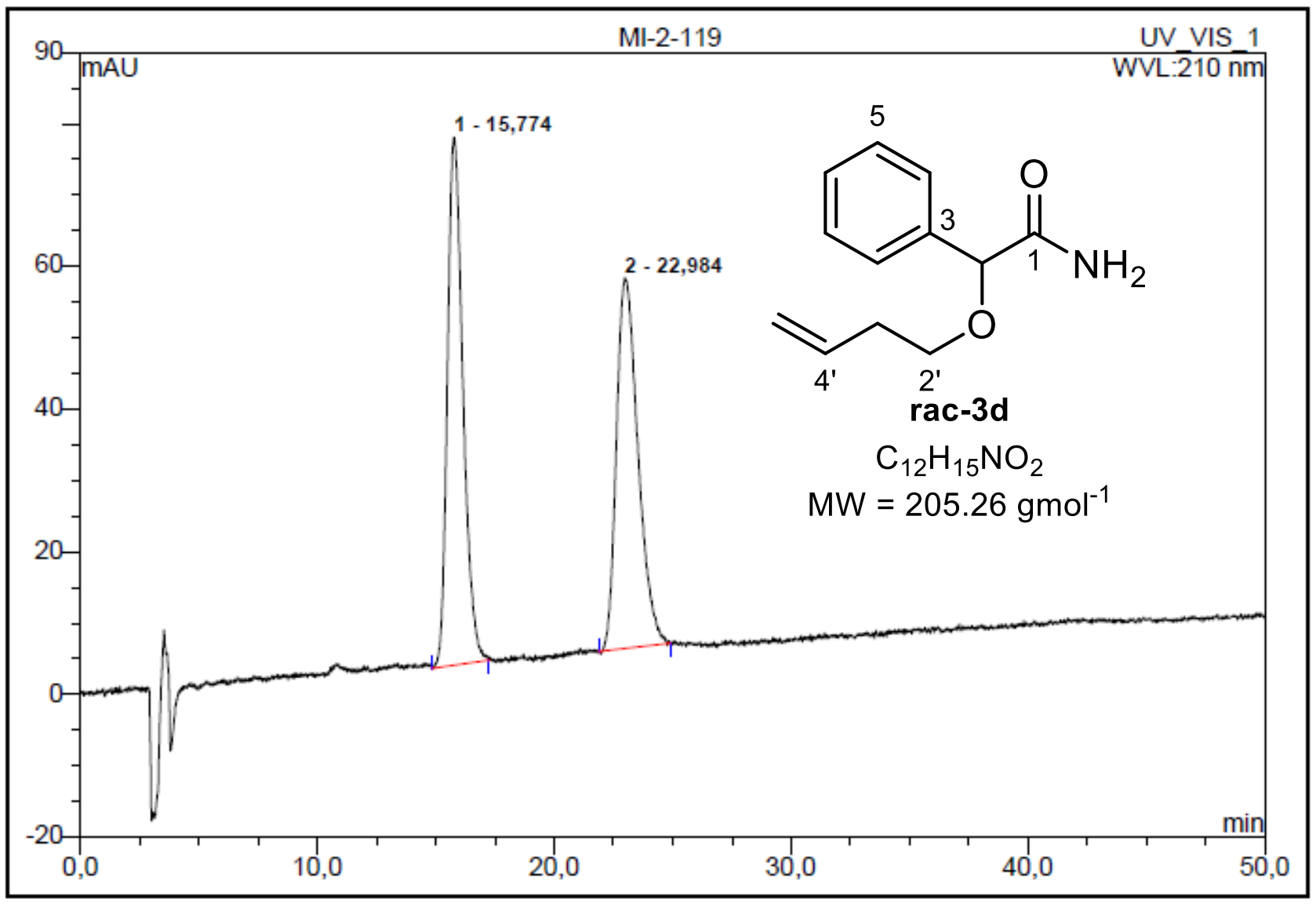

No.	Ret.Time min	Peak Name	Height mAU	Area mAU*min	Rel.Area %	Amount	Type
1	15,77	n.a.	73,985	56,072	50,71	n.a.	BMB*
2	22,98	n.a.	51,960	54,500	49,29	n.a.	BMB*
Total:			125,945	110,573	100,00	0,000	

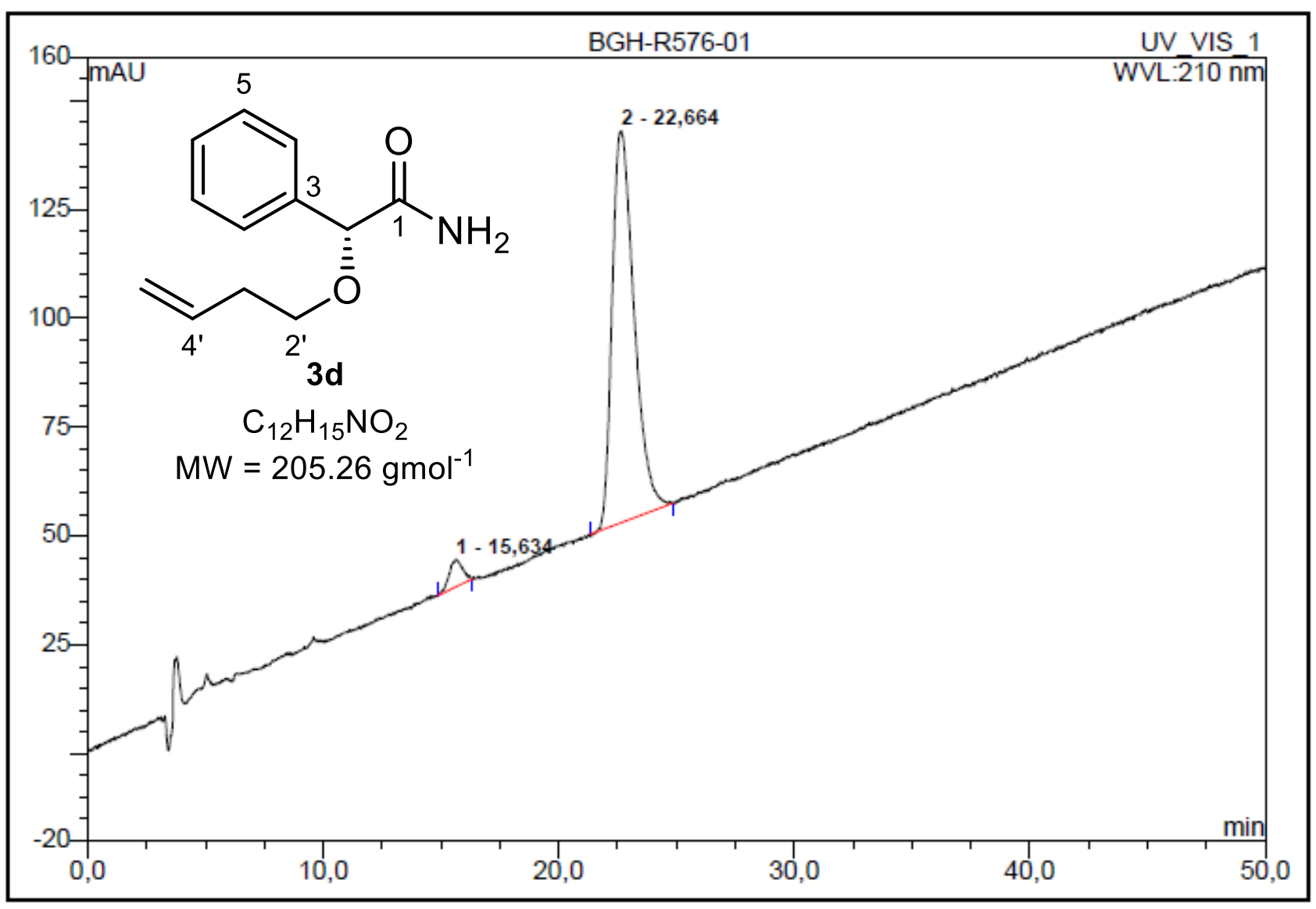

No.	Ret.Time min	Peak Name	Height mAU	Area mAU*min	Rel.Area %	Amount	Type
1	15,63	n.a.	6,378	4,101	4,05	n.a.	BMB*
2	22,66	n.a.	89,979	97,119	95,95	n.a.	BMB*
Total:			96,357	101,220	100,00	0,000	

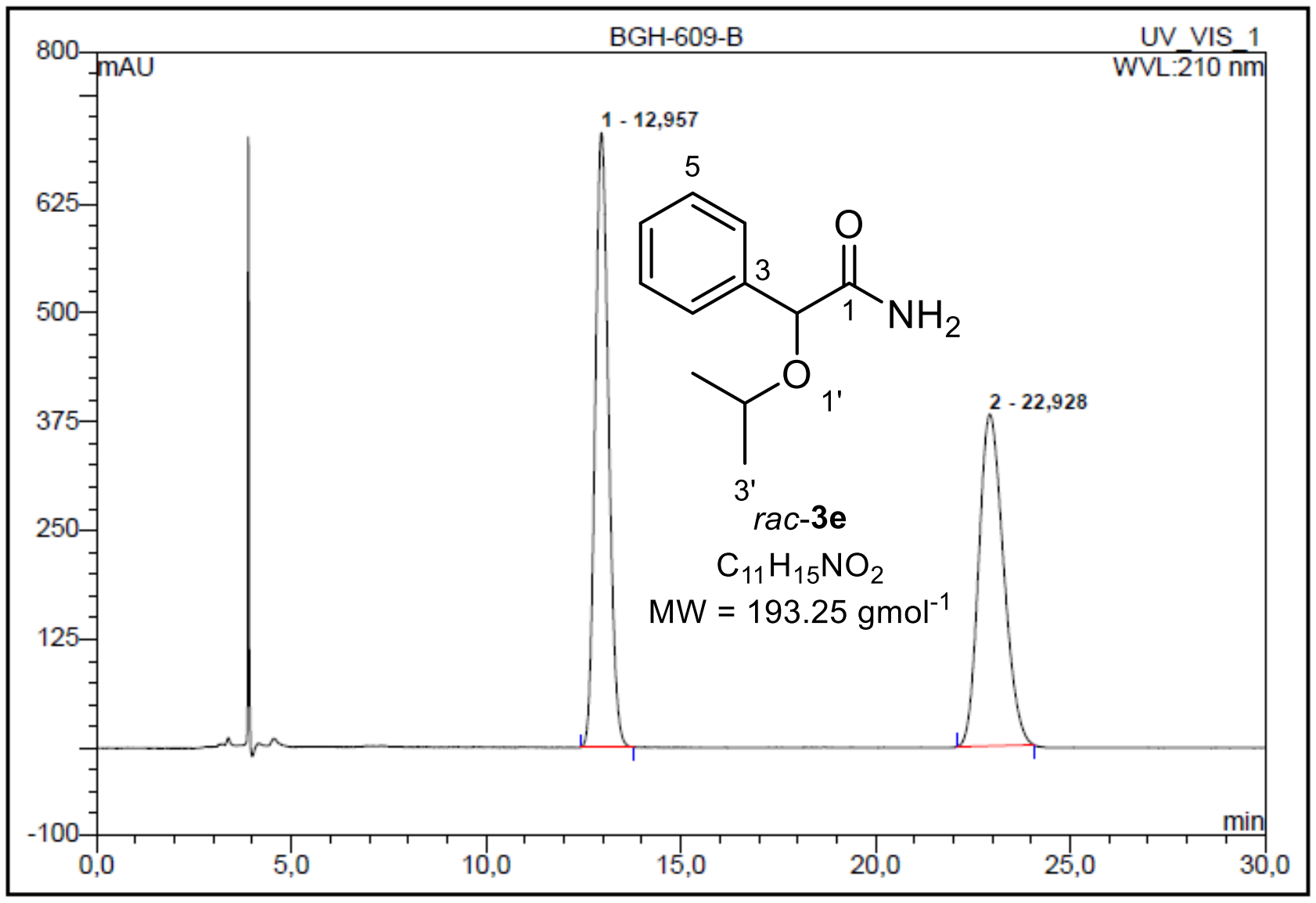

No.	Ret.Time min	Peak Name	Height mAU	Area mAU*min	Rel.Area %	Amount	Type
1	12,96	n.a.	706,009	282,561	50,20	n.a.	BMB
2	22,93	n.a.	381,084	280,264	49,80	n.a.	BMB
Total:			1087,092	562,825	100,00	0,000	

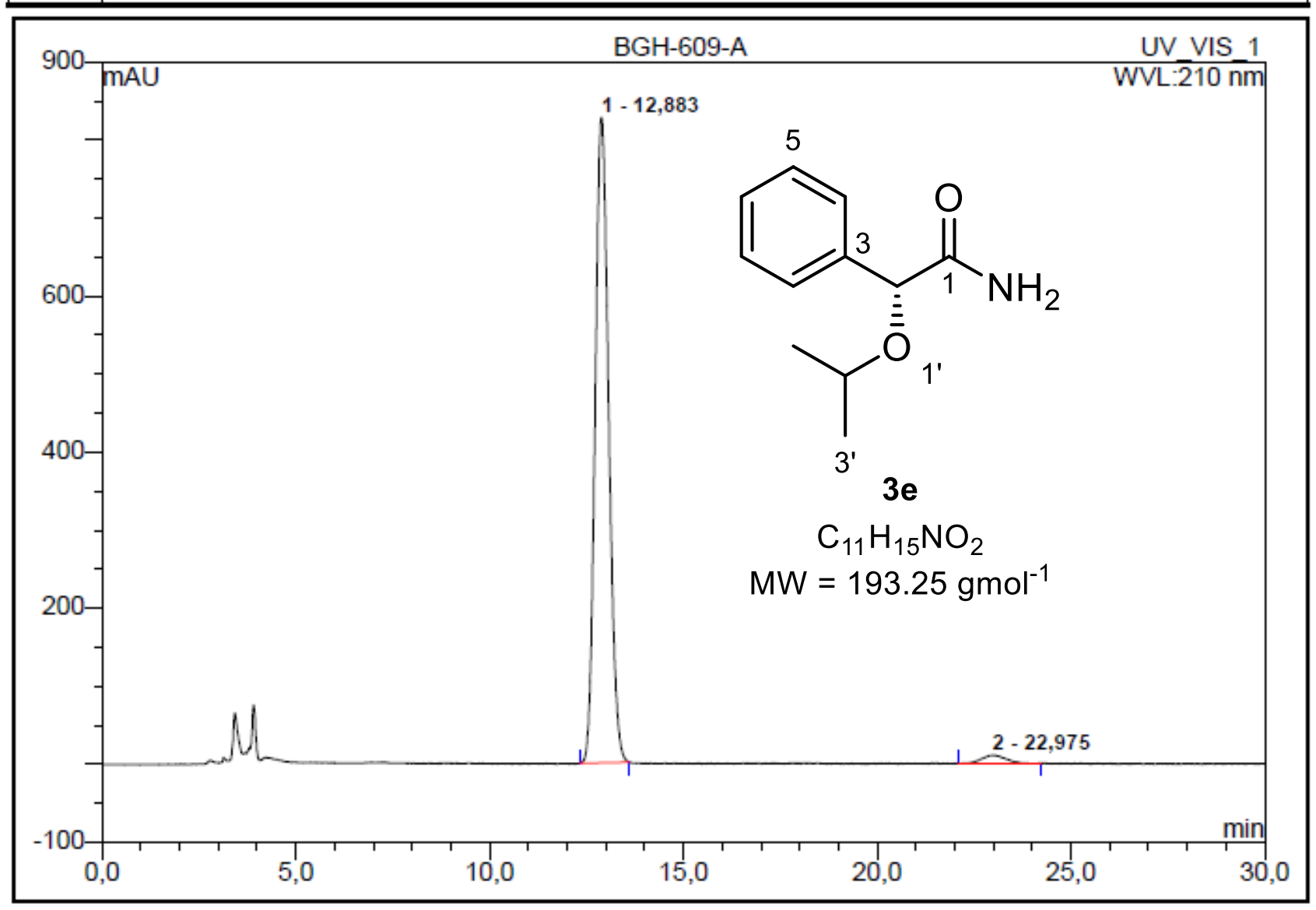

No.	Ret.Time min	Peak Name	Height mAU	Area mAU*min	Rel.Area %	Amount	Type
1	12,88	n.a.	826,744	333,947	97,92	n.a.	BMB
2	22,98	n.a.	10,141	7,098	2,08	n.a.	BMB*
Total:			836,884	341,046	100,00	0,000	

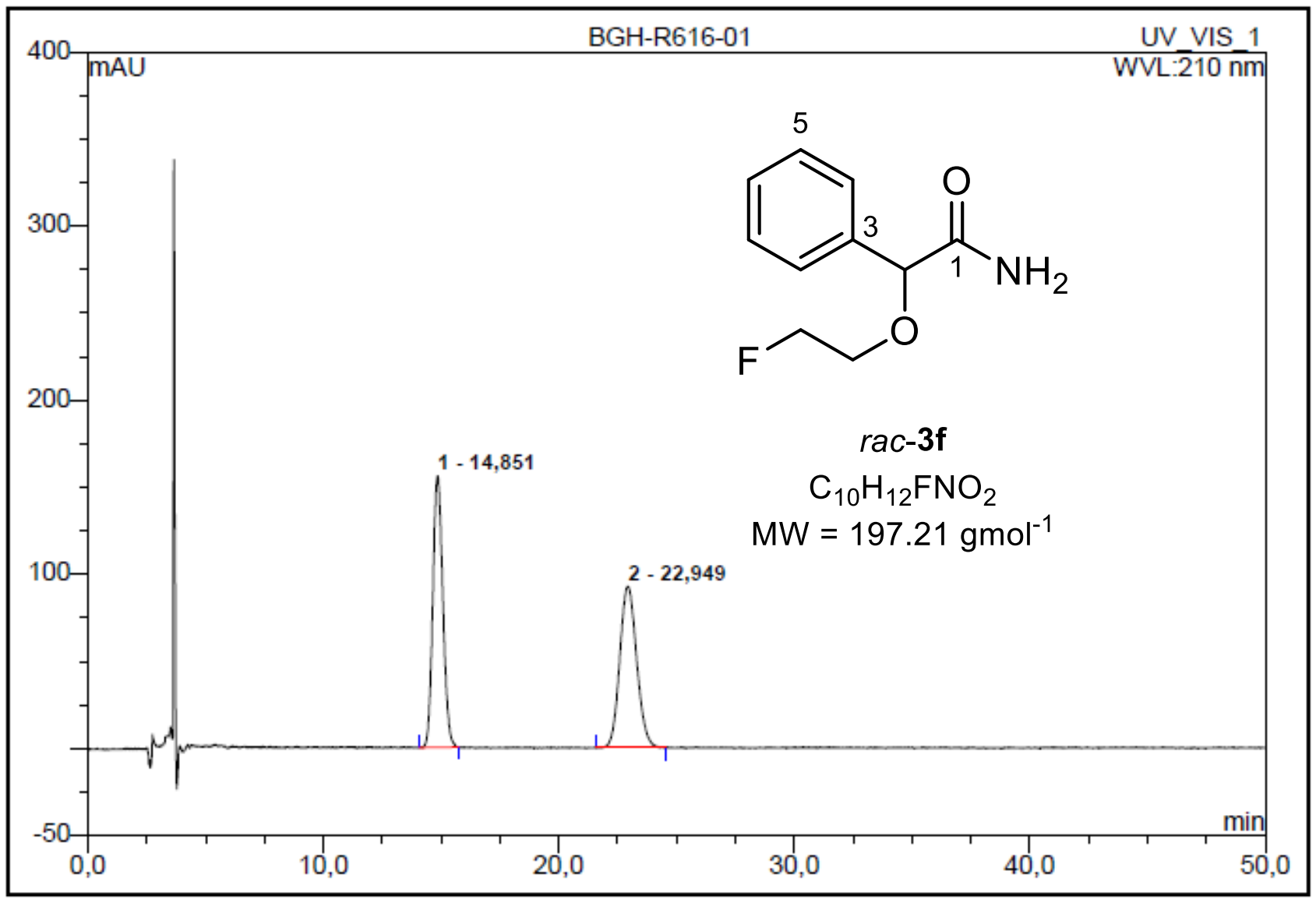

No.	Ret.Time min	Peak Name	Height mAU	Area mAU*min	Rel.Area %	Amount	Type
1	14,85	n.a.	155,966	77,027	49,88	n.a.	BMB*
2	22,95	n.a.	92,748	77,406	50,12	n.a.	BMB*
Total:			248,714	154,433	100,00	0,000	

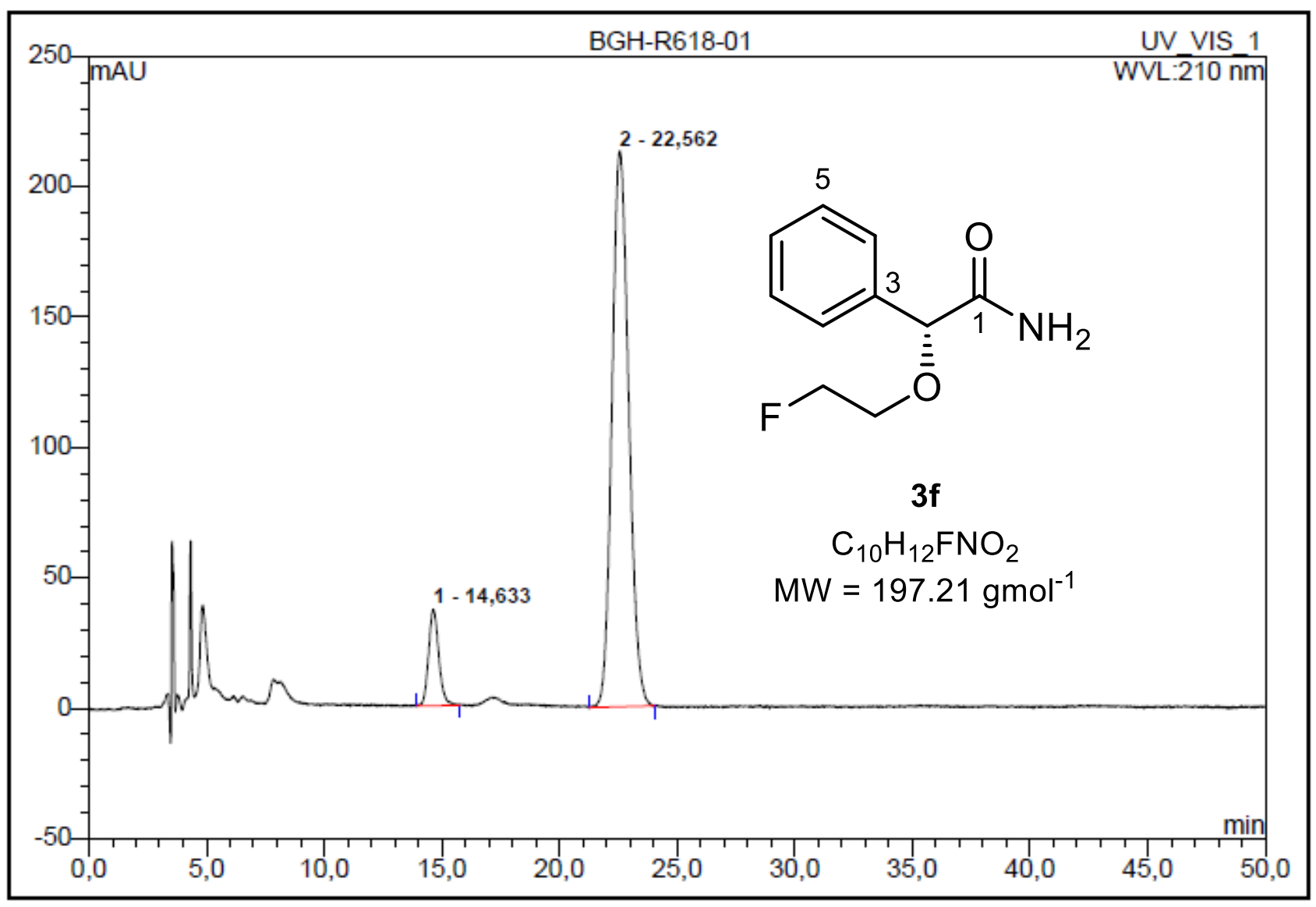

No.	Ret.Time min	Peak Name	Height mAU	Area mAU*min	Rel.Area %	Amount	Type
1	14,63	n.a.	36,888	18,631	9,60	n.a.	BMB*
2	22,56	n.a.	212,895	175,500	90,40	n.a.	BMB*
Total:			249,783	194,131	100,00	0,000	

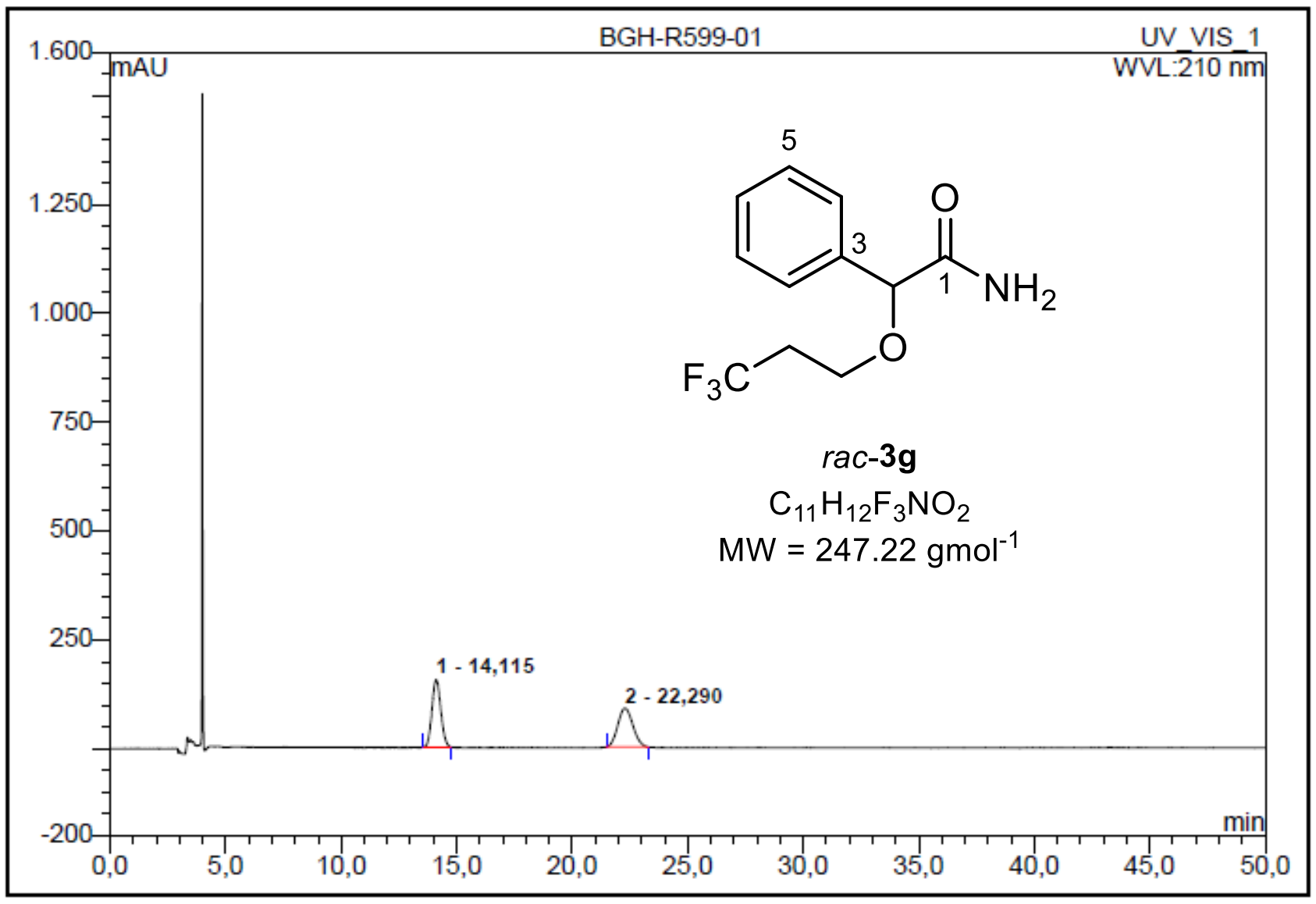

No.	Ret.Time min	Peak Name	Height mAU	Area mAU*min	Rel.Area %	Amount	Type
1	14,12	n.a.	155,594	69,412	50,77	n.a.	BMB
2	22,29	n.a.	88,793	67,299	49,23	n.a.	BMB
Total:			244,387	136,711	100,00	0,000	

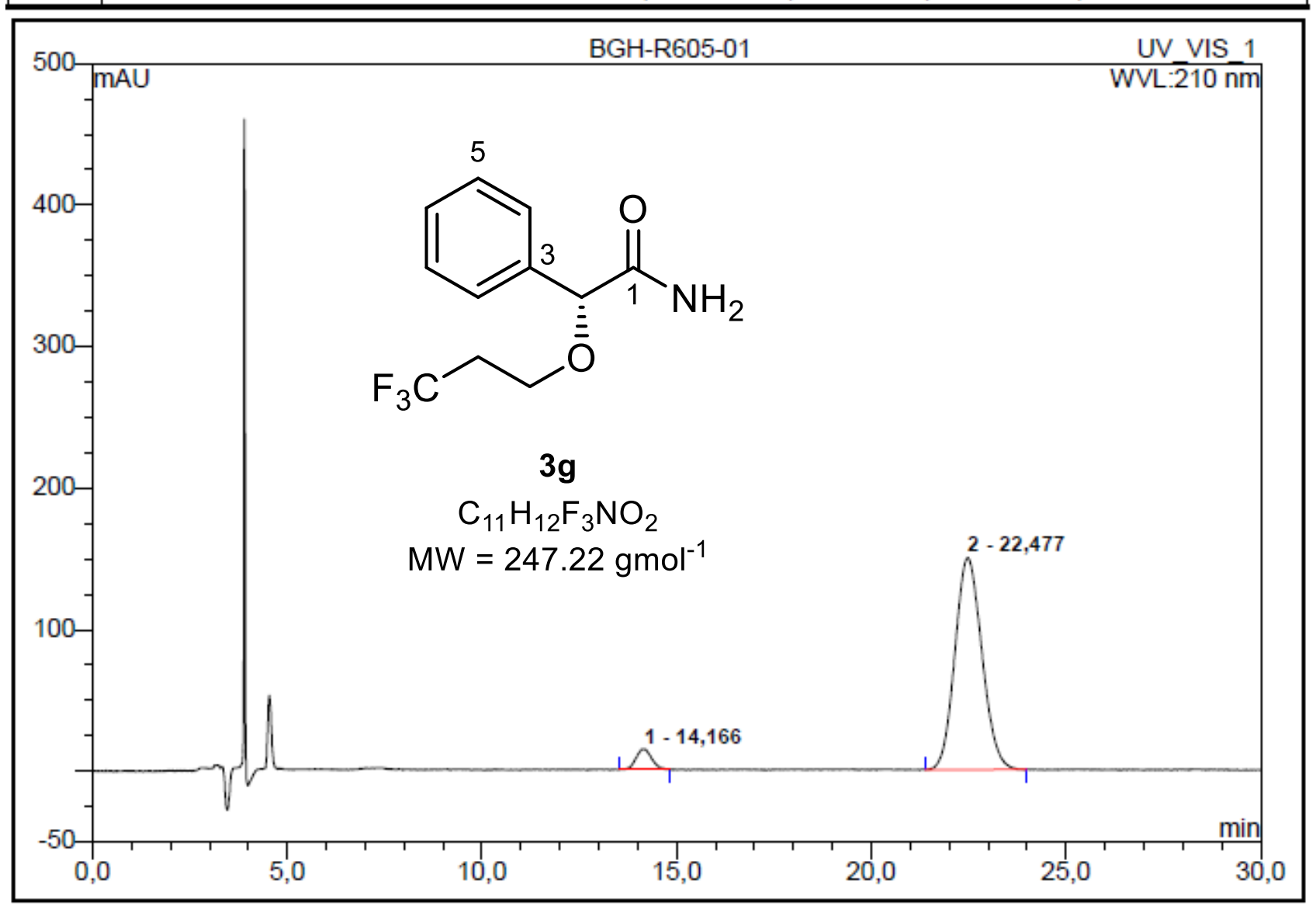

No.	Ret.Time min	Peak Name	Height mAU	Area mAU*min	Rel.Area %	Amount	Type
1	14,17	n.a.	14,564	6,774	5,32	n.a.	BMB*
2	22,48	n.a.	149,855	120,570	94,68	n.a.	BMB*
Total:			164,419	127,343	100,00	0,000	

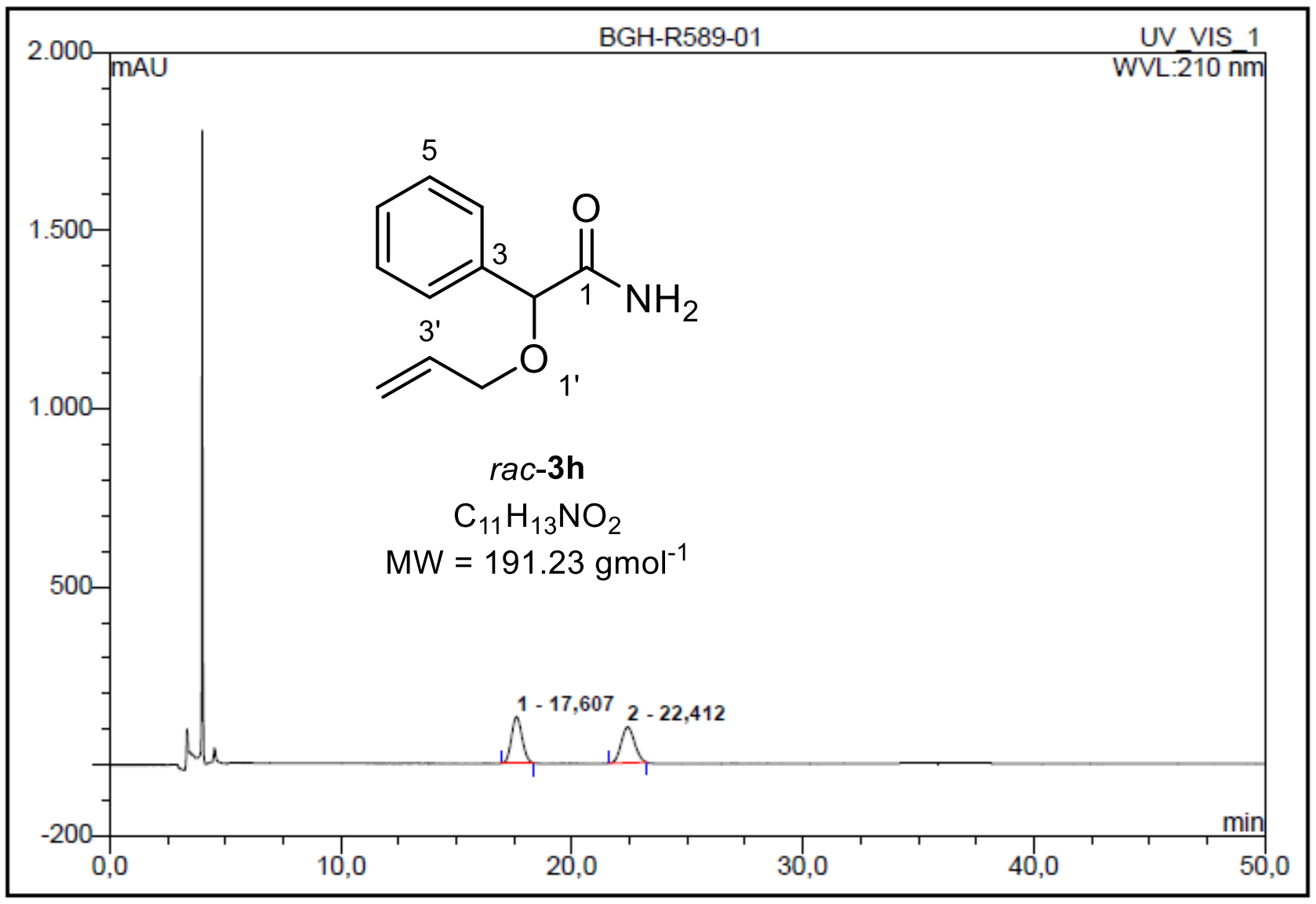

No.	Ret.Time min	Peak Name	Height mAU	Area mAU*min	Rel.Area %	Amount	Type
1	17,61	n.a.	131,216	70,442	51,09	n.a.	BMB
2	22,41	n.a.	100,395	67,426	48,91	n.a.	BMB
Total:			231,611	137,868	100,00	0,000	

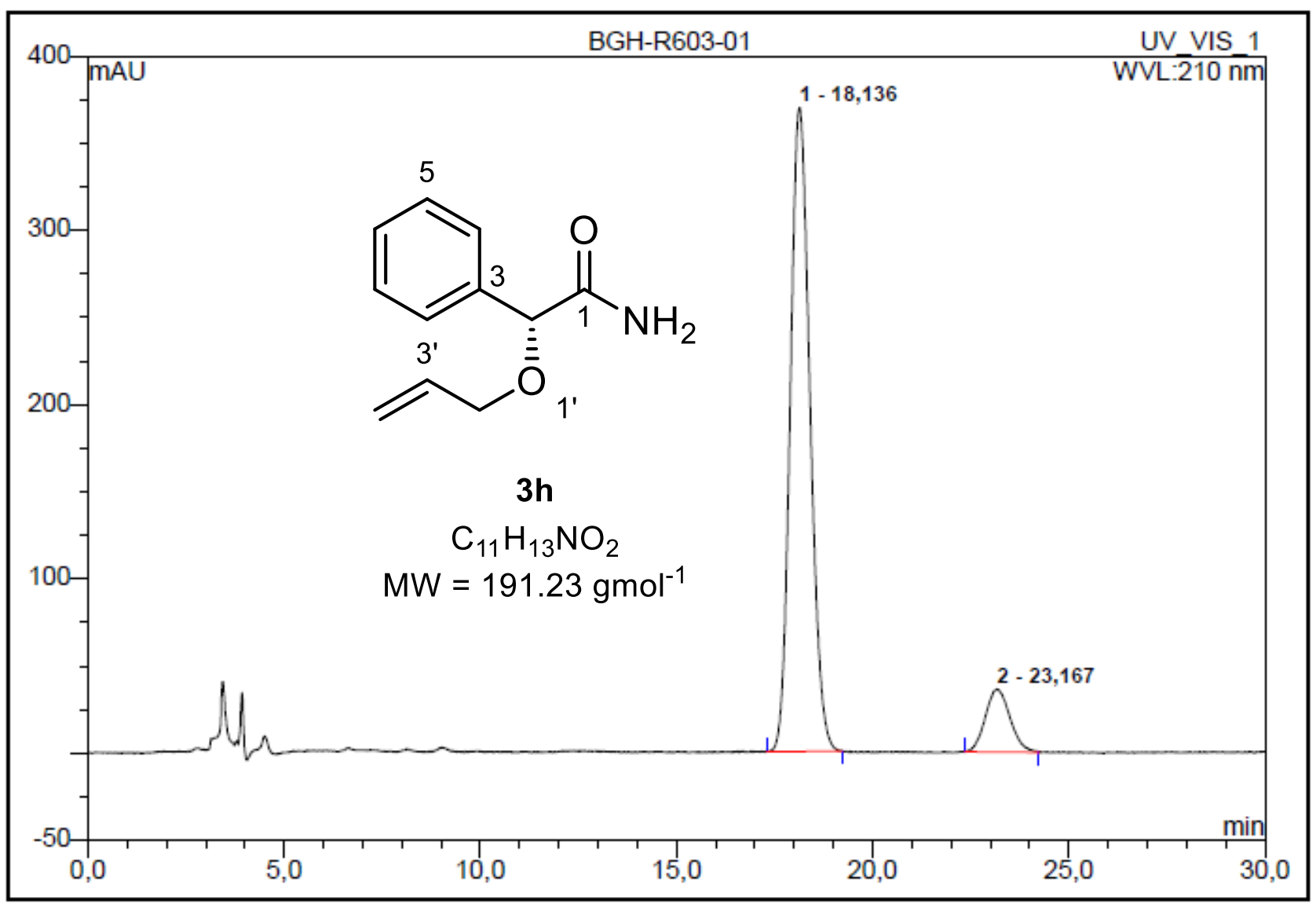

No.	Ret.Time min	Peak Name	Height mAU	Area mAU*min	Rel.Area %	Amount	Type
1	18,14	n.a.	369,579	214,484	89,10	n.a.	BMB*
2	23,17	n.a.	36,166	26,241	10,90	n.a.	BMB*
Total:			405,745	240,725	100,00	0,000	

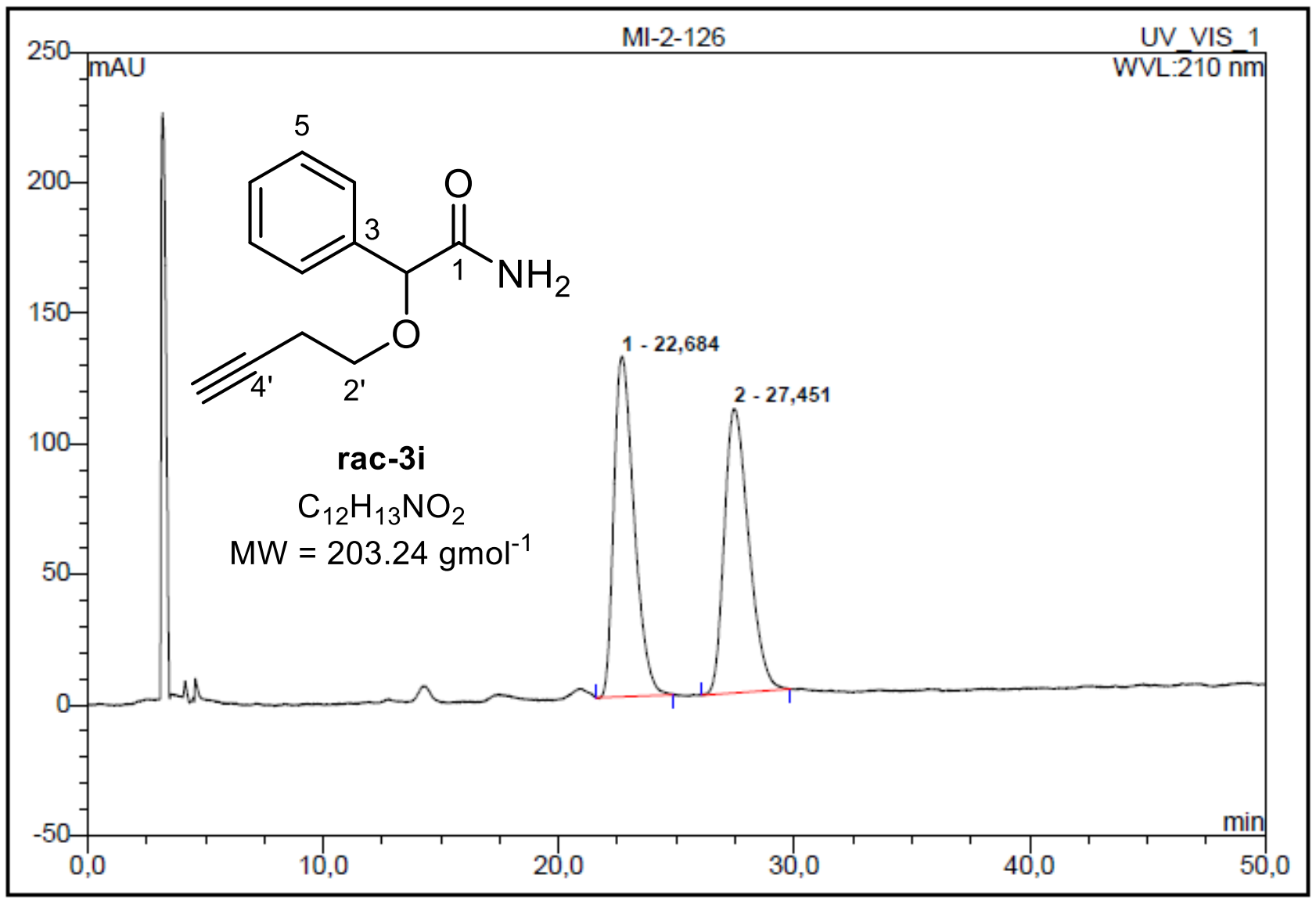

No.	Ret.Time min	Peak Name	Height mAU	Area mAU*min	Rel.Area %	Amount	Type
1	22,68	n.a.	130,201	130,109	49,79	n.a.	BMB*
2	27,45	n.a.	108,995	131,220	50,21	n.a.	BMB*
Total:			239,196	261,329	100,00	0,000	

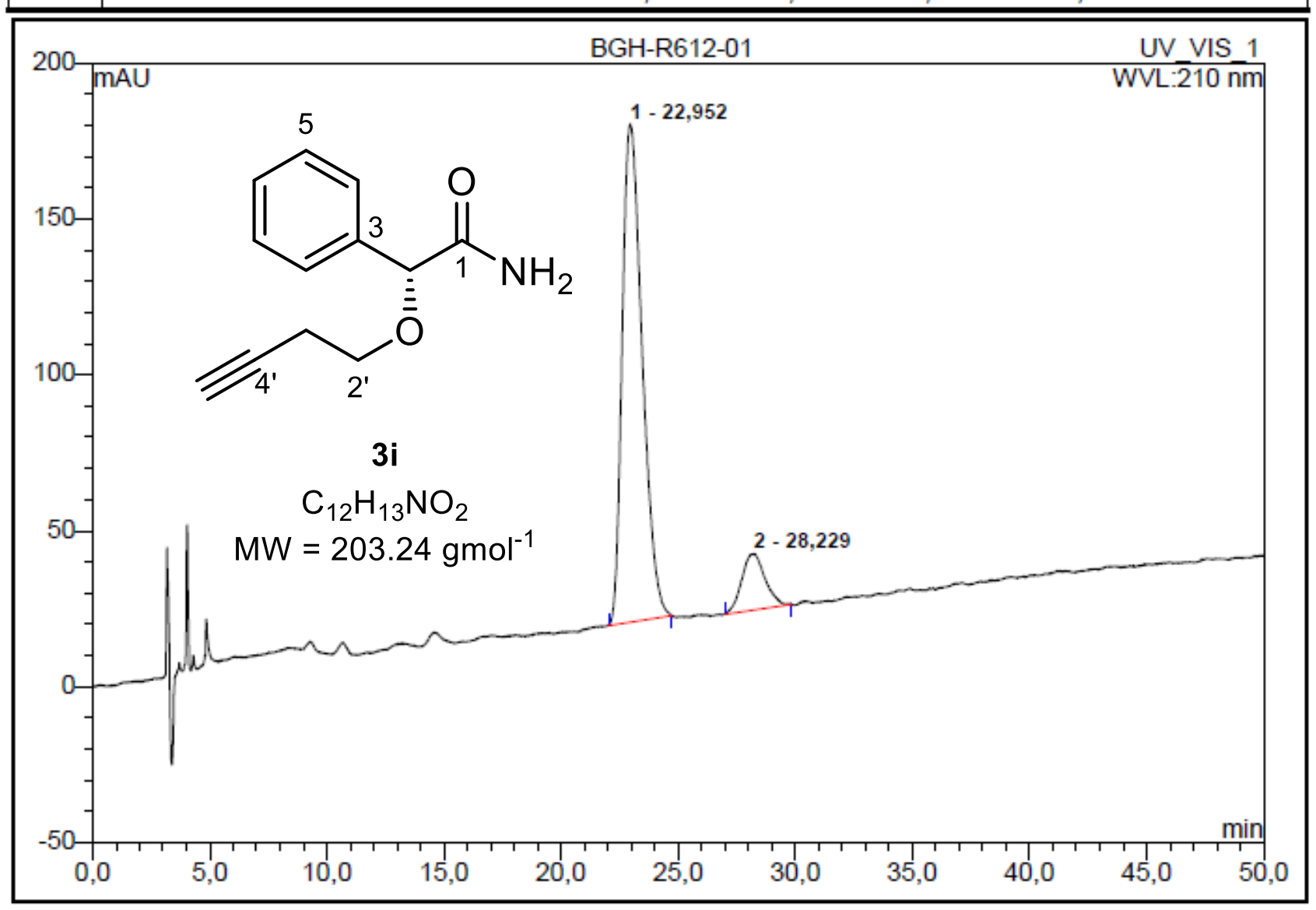

No.	Ret.Time min	Peak Name	Height mAU	Area mAU*min	Rel.Area %	Amount	Type
1	22,95	n.a.	159,760	157,753	88,31	n.a.	BMB
2	28,23	n.a.	18,081	20,873	11,69	n.a.	BMB*
Total:			177,841	178,627	100,00	0,000	

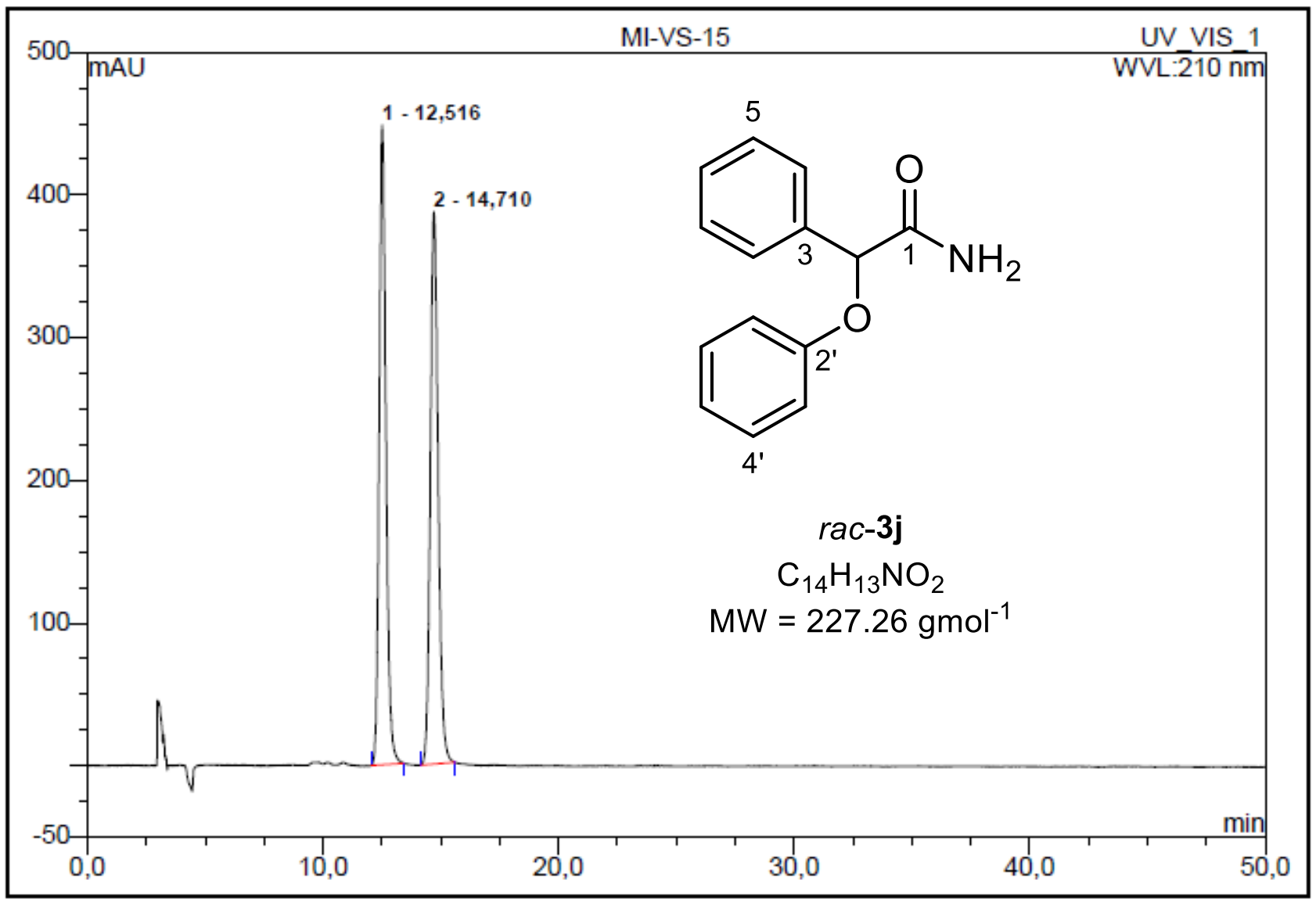

No.	Ret.Time min	Peak Name	Height mAU	Area mAU*min	Rel.Area %	Amount	Type
1	12,52	n.a.	448,098	150,098	50,08	n.a.	BMB
2	14,71	n.a.	386,965	149,628	49,92	n.a.	BMB
Total:			835,063	299,727	100,00	0,000	

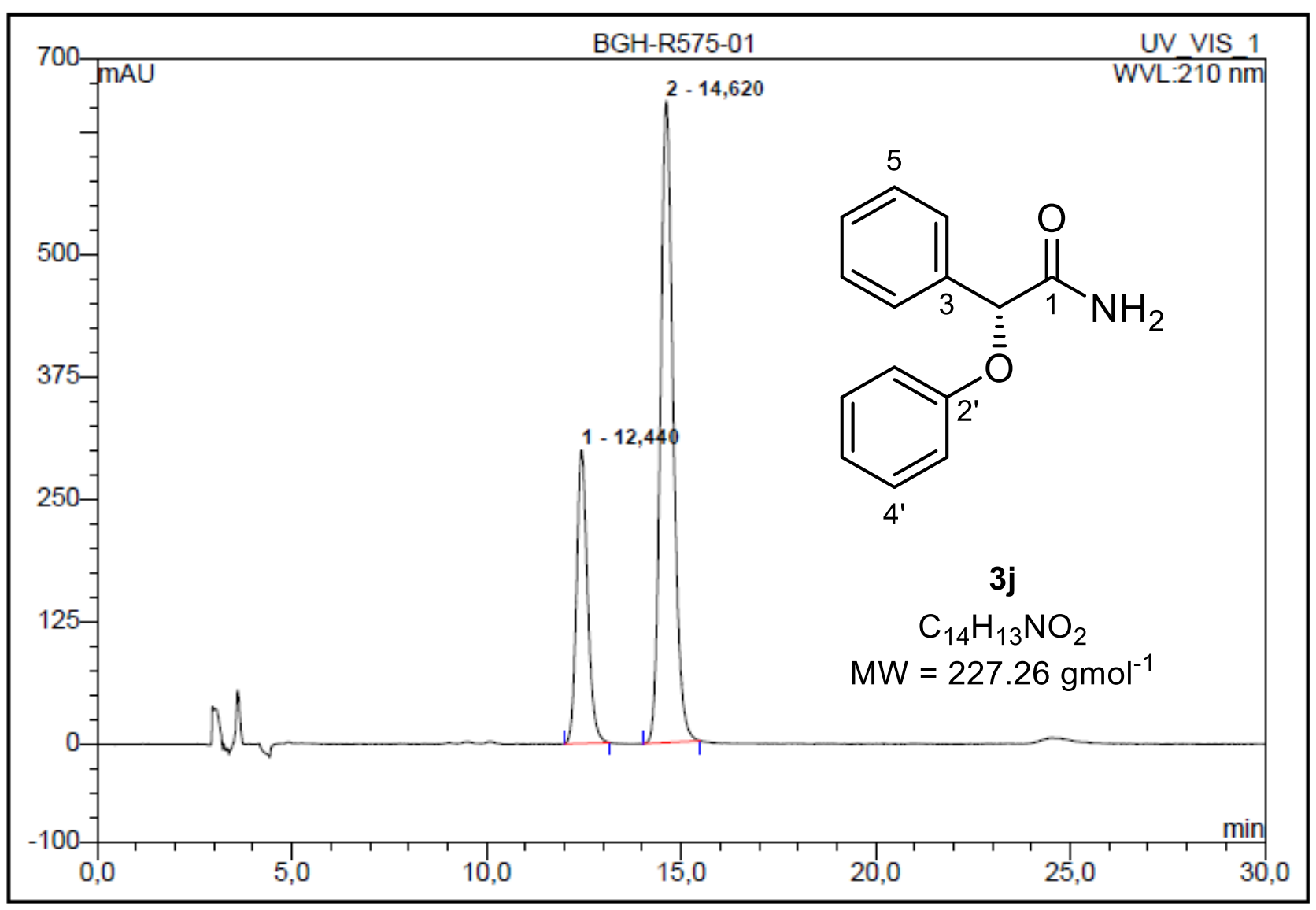

No.	Ret.Time min	Peak Name	Height mAU	Area mAU*min	Rel.Area %	Amount	Type
1	12,44	n.a.	298,958	98,193	28,24	n.a.	BMB
2	14,62	n.a.	654,790	249,462	71,76	n.a.	BMB
Total:			953,748	347,655	100,00	0,000	

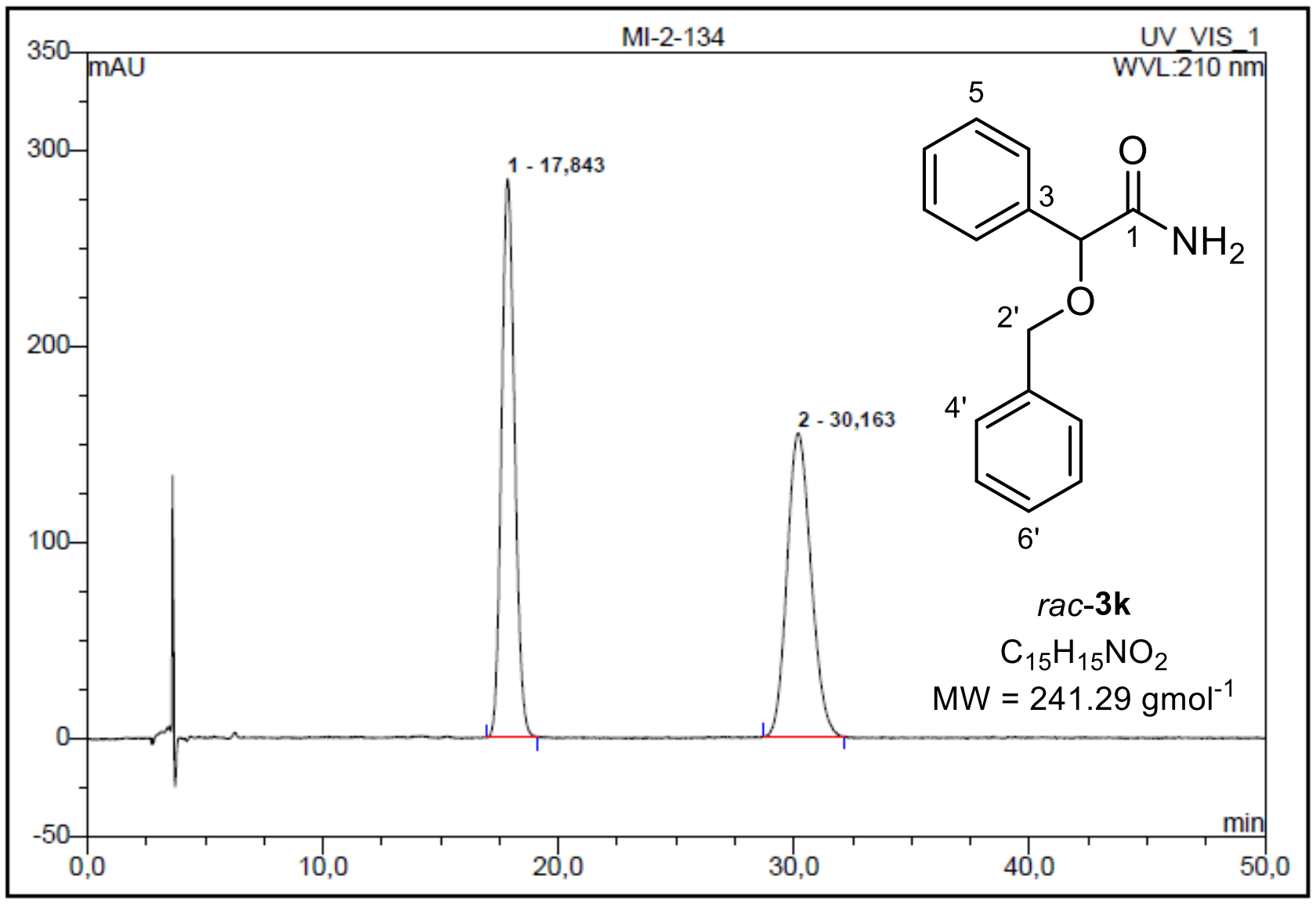

No.	Ret.Time min	Peak Name	Height mAU	Area mAU*min	Rel.Area %	Amount	Type
1	17,84	n.a.	284,943	183,968	50,23	n.a.	BMB*
2	30,16	n.a.	154,669	182,282	49,77	n.a.	BMB*
Total:			439,613	366,250	100,00	0,000	

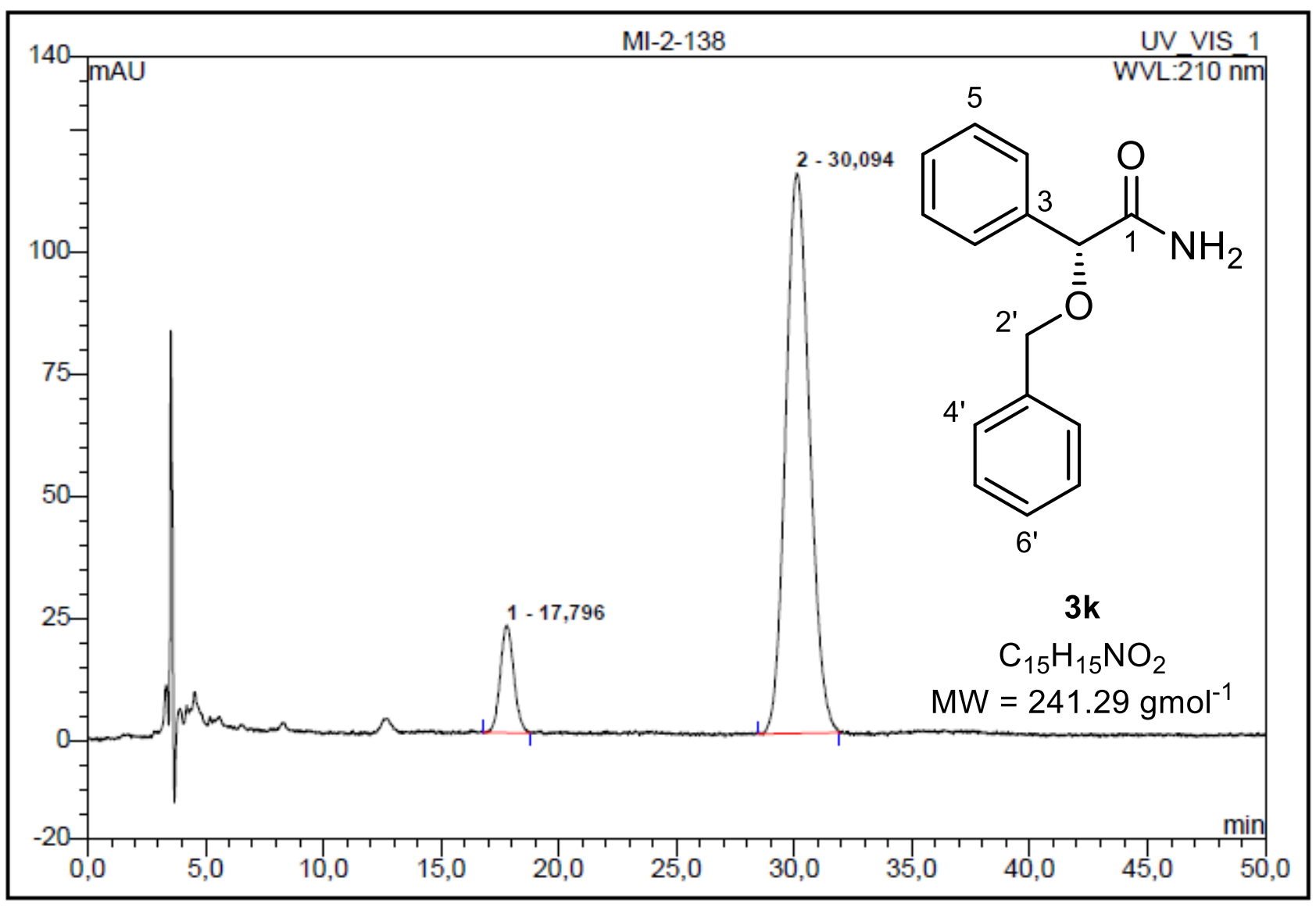

No.	Ret.Time min	Peak Name	Height mAU	Area mAU*min	Rel.Area %	Amount	Type
1	17,80	n.a.	22,037	14,683	9,84	n.a.	BMB*
2	30,09	n.a.	114,550	134,585	90,16	n.a.	BMB*
Total:			136,587	149,268	100,00	0,000	

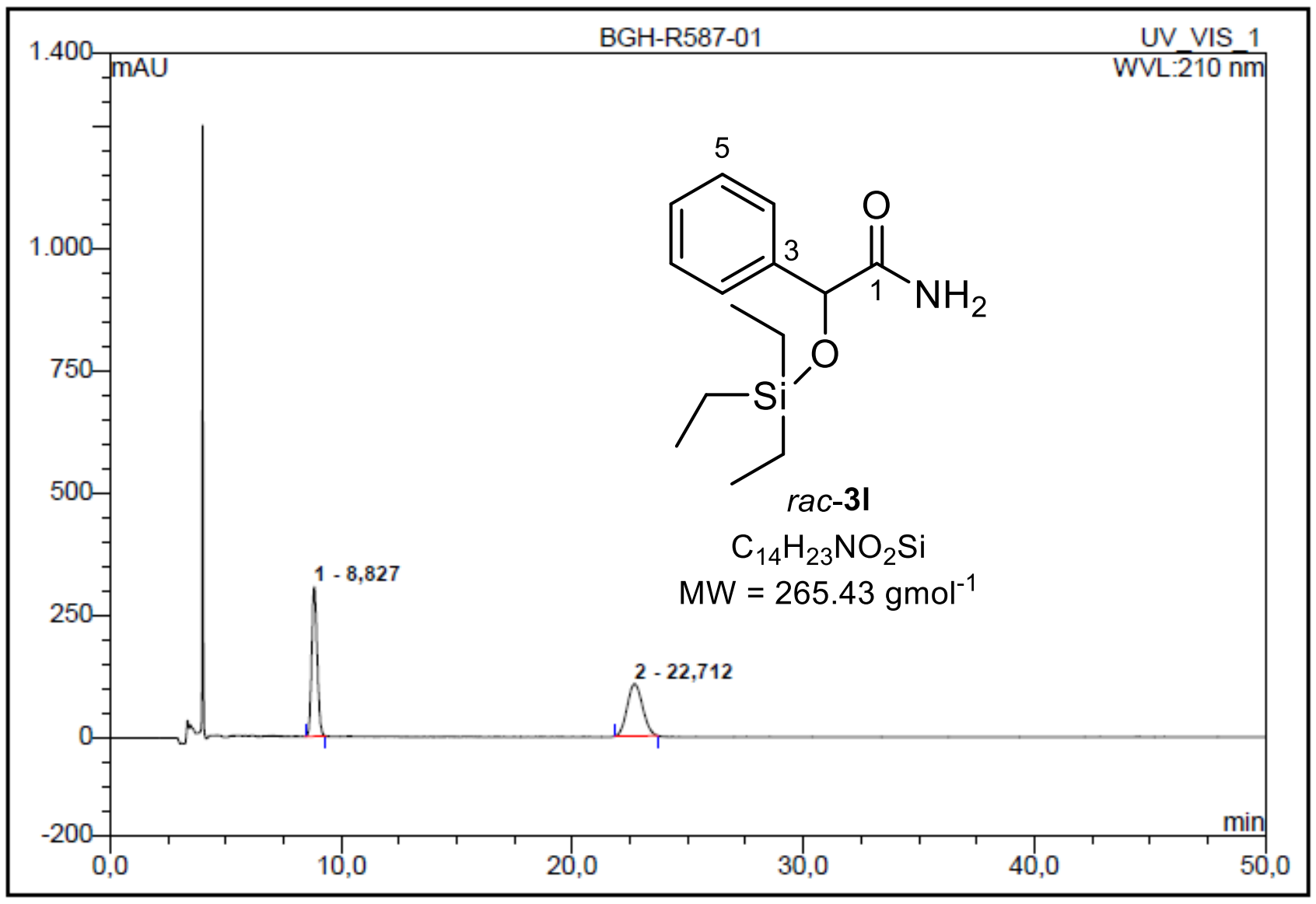

No.	Ret.Time min	Peak Name	Height mAU	Area mAU*min	Rel.Area %	Amount	Type
1	8,83	n.a.	305,382	85,170	50,77	n.a.	BMB
2	22,71	n.a.	106,810	82,579	49,23	n.a.	BMB
Total:			412,192	167,749	100,00	0,000	

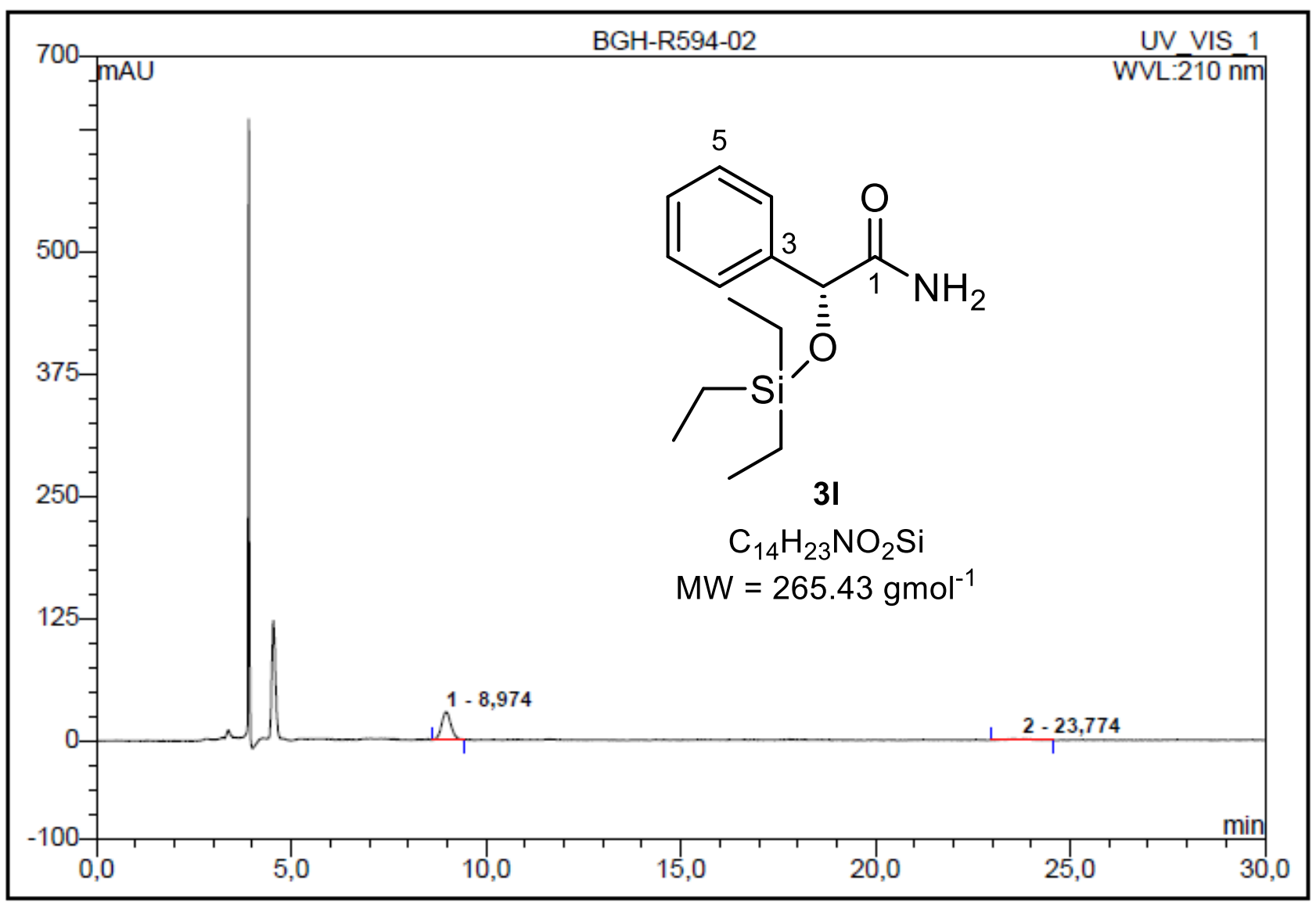

No.	Ret.Time min	Peak Name	Height mAU	Area mAU*min	Rel.Area %	Amount	Type
1	8,97	n.a.	28,076	7,705	89,50	n.a.	BMB*
2	23,77	n.a.	1,472	0,904	10,50	n.a.	BMB*
Total:			29,548	8,609	100,00	0,000	

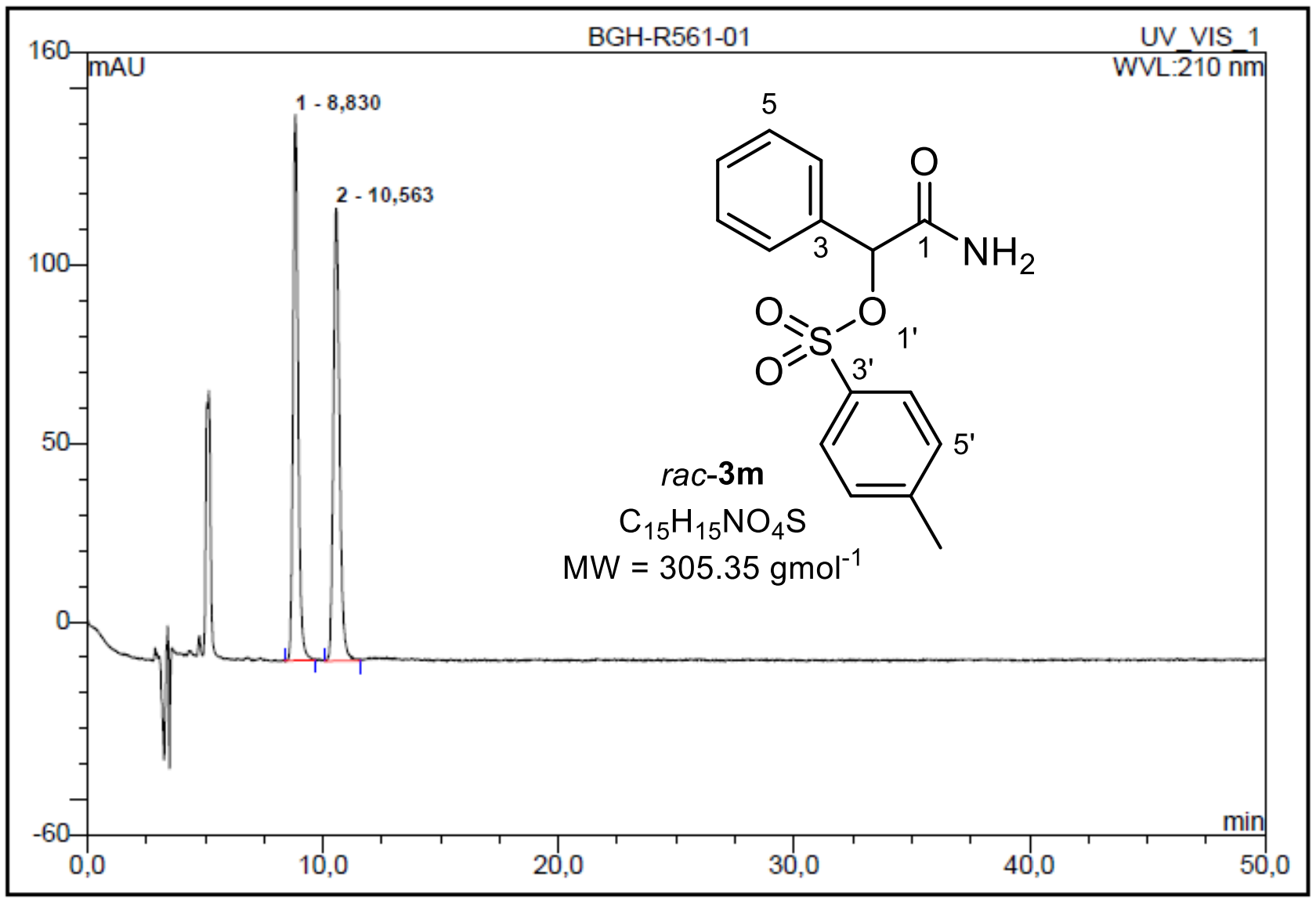

No.	Ret.Time min	Peak Name	Height mAU	Area mAU*min	Rel.Area %	Amount	Type
1	8,83	n.a.	153,222	38,460	49,99	n.a.	BMB*
2	10,56	n.a.	127,070	38,481	50,01	n.a.	BMB*
Total:			280,292	76,941	100,00	0,000	

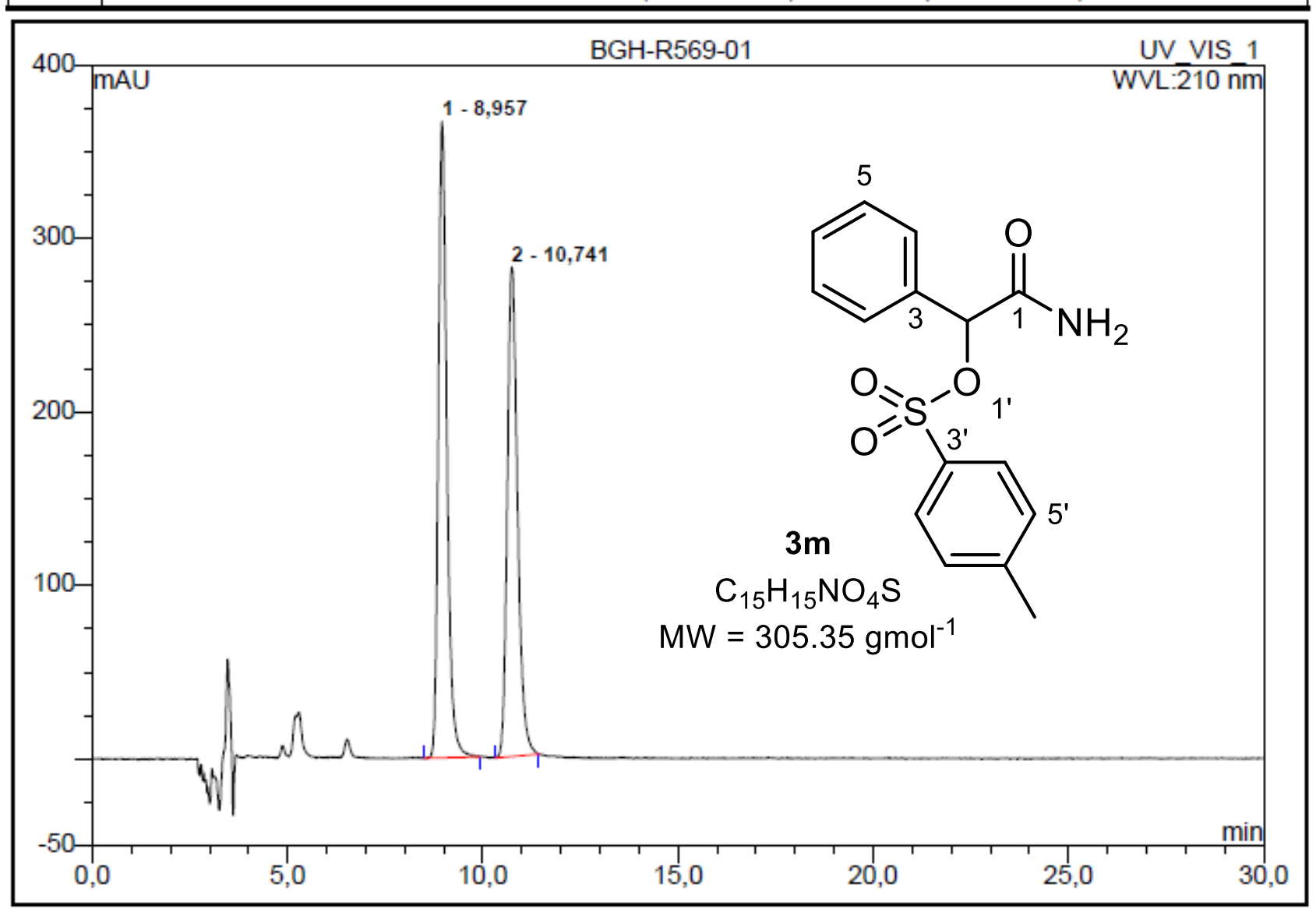

No.	Ret.Time min	Peak Name	Height mAU	Area mAU*min	Rel.Area %	Amount	Type
1	8,96	n.a.	366,868	92,947	52,07	n.a.	BMB*
2	10,74	n.a.	282,160	85,550	47,93	n.a.	BMB
Total:			649,028	178,497	100,00	0,000	

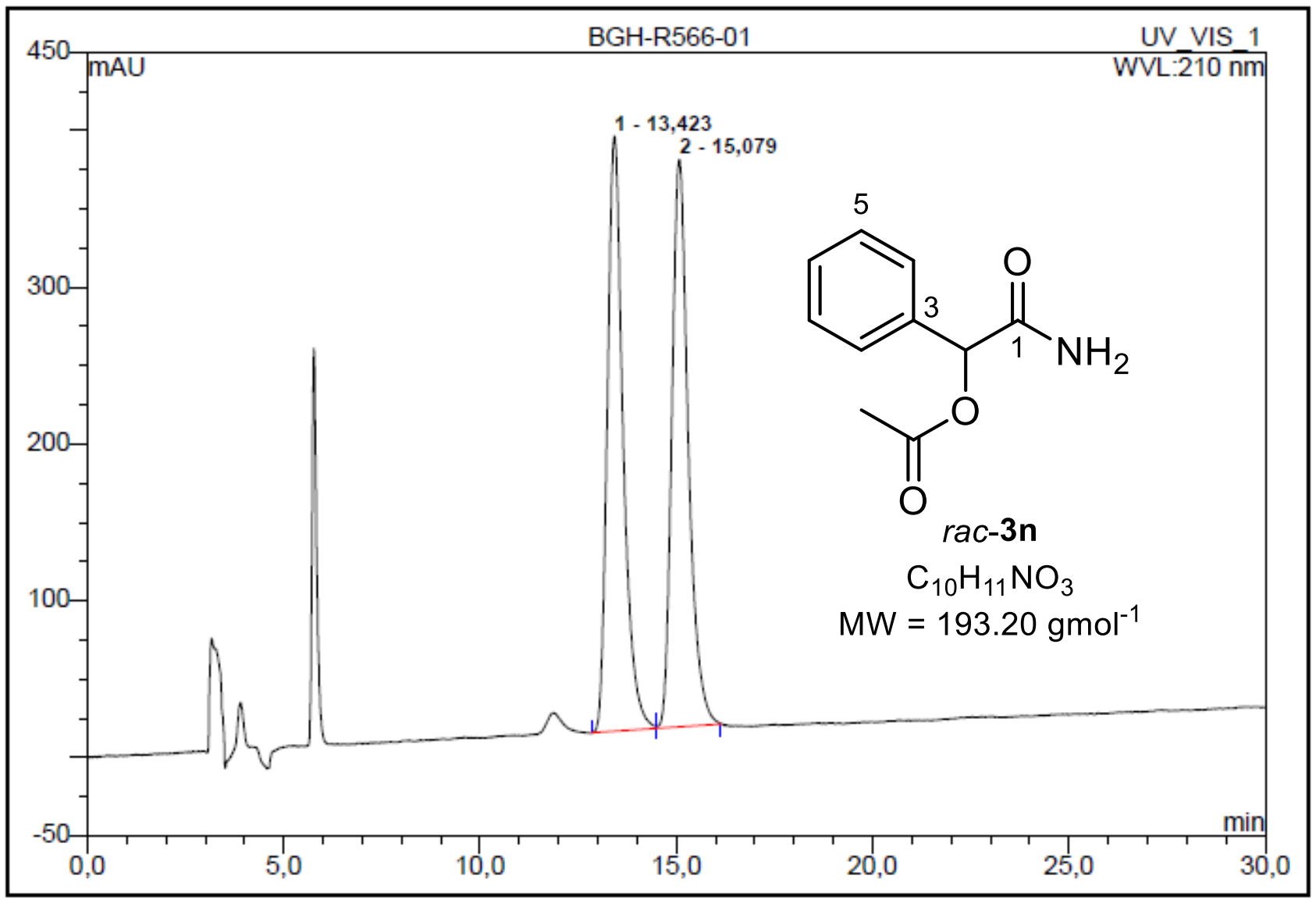

No.	Ret.Time min	Peak Name	Height mAU	Area mAU*min	Rel.Area %	Amount	Type
1	13,42	n.a.	379,466	177,080	50,19	n.a.	BM
2	15,08	n.a.	361,891	175,760	49,81	n.a.	MB
Total:			741,357	352,840	100,00	0,000	

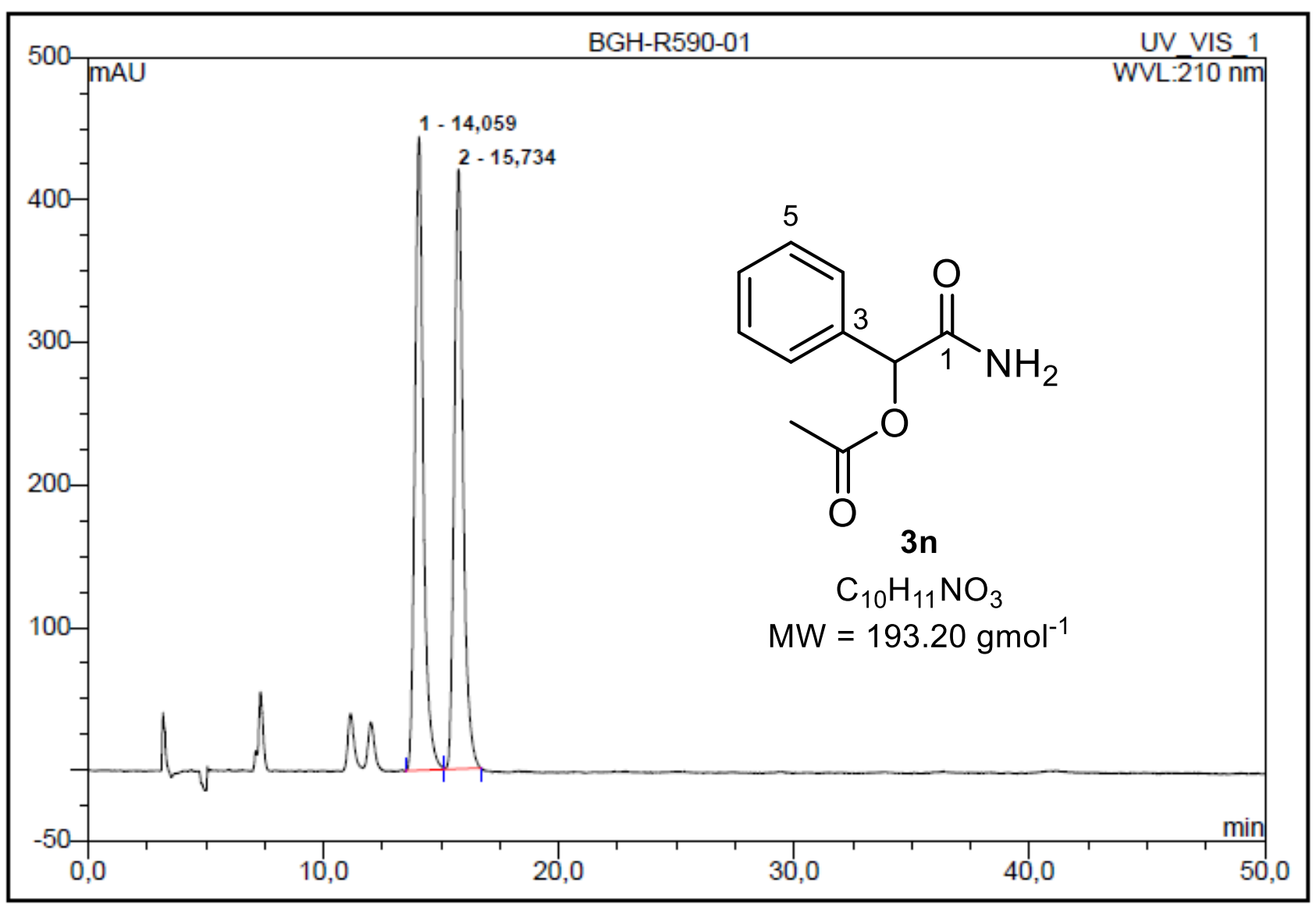

No.	Ret.Time min	Peak Name	Height mAU	Area mAU*min	Rel.Area %	Amount	Type
1	14,06	n.a.	444,538	185,745	50,33	n.a.	BM
2	15,73	n.a.	420,717	183,294	49,67	n.a.	MB
Total:			865,255	369,039	100,00	0,000	

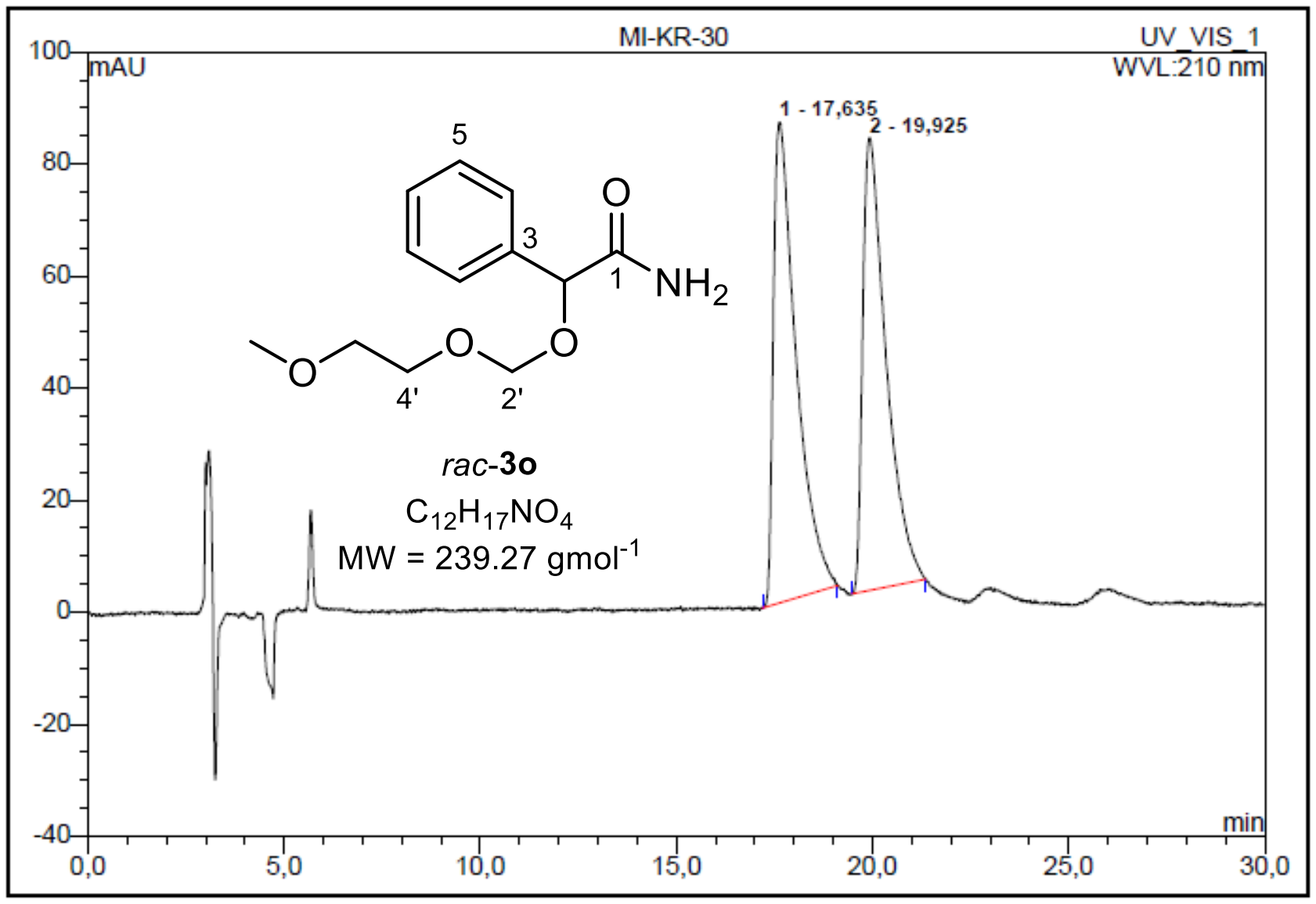

No.	Ret.Time min	Peak Name	Height mAU	Area mAU*min	Rel.Area %	Amount	Type
1	17,64	n.a.	85,758	57,736	50,86	n.a.	BMB*
2	19,92	n.a.	80,807	55,784	49,14	n.a.	BMB
Total:			166,565	113,520	100,00	0,000	

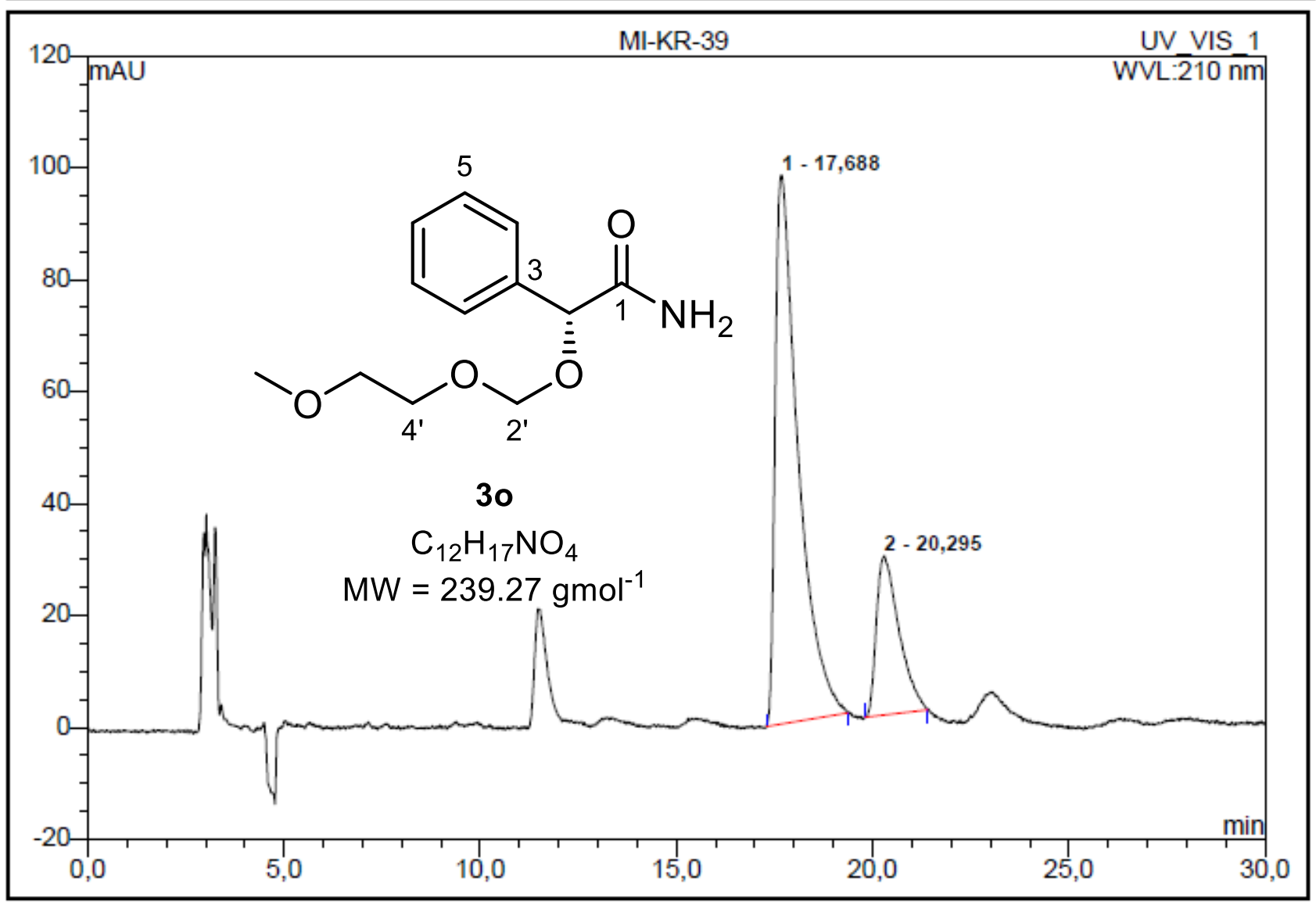

No.	Ret.Time min	Peak Name	Height mAU	Area mAU*min	Rel.Area %	Amount	Type
1	17,69	n.a.	98,026	66,766	77,92	n.a.	BMB*
2	20,29	n.a.	28,398	18,917	22,08	n.a.	BMB*
Total:			126,424	85,684	100,00	0,000	

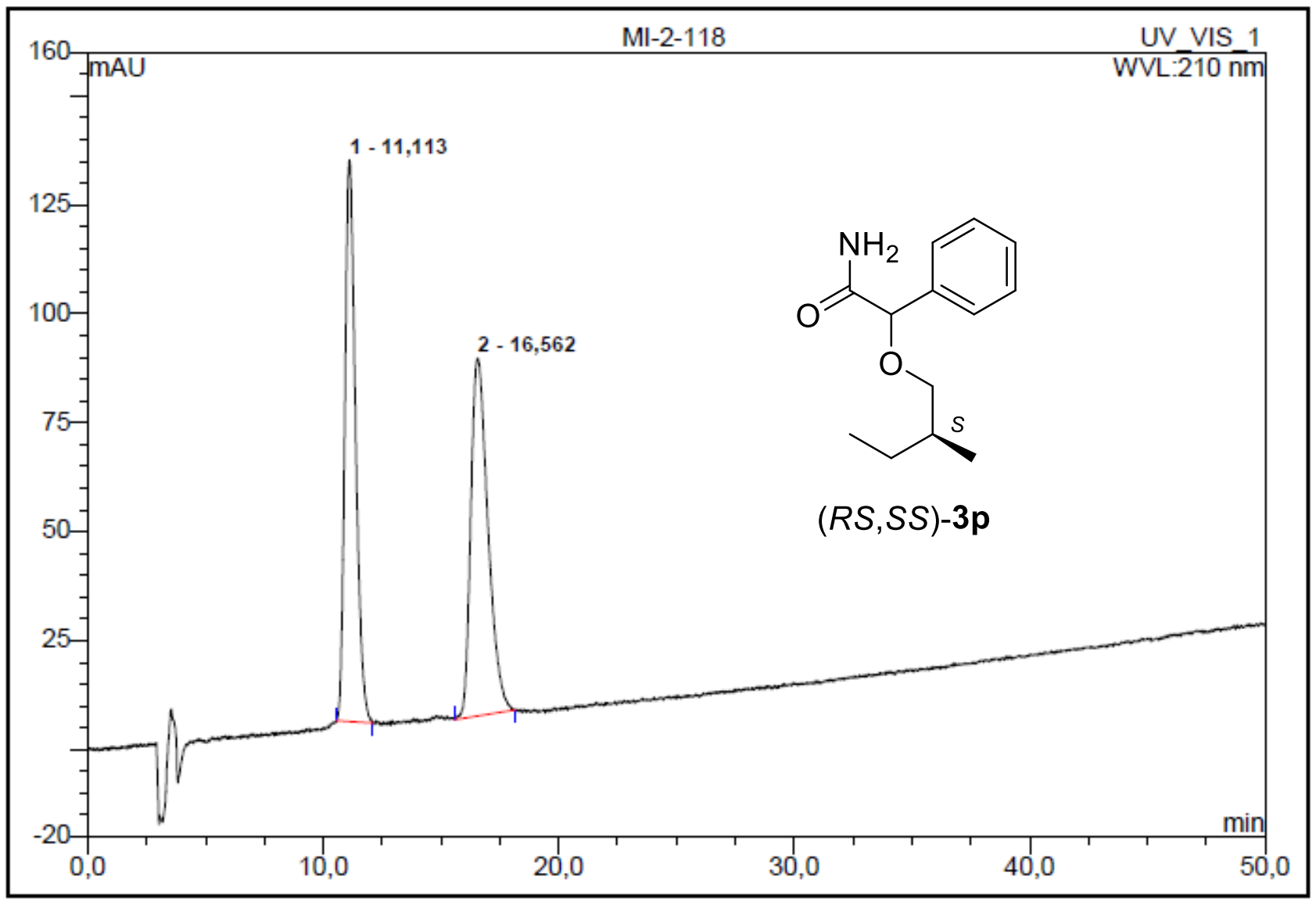

No.	Ret.Time min	Peak Name	Height mAU	Area mAU*min	Rel.Area %	Amount	Type
1	11,11	n.a.	128,881	67,833	49,46	n.a.	BMB*
2	16,56	n.a.	82,069	69,303	50,54	n.a.	BMB*
Total:			210,950	137,136	100,00	0,000	

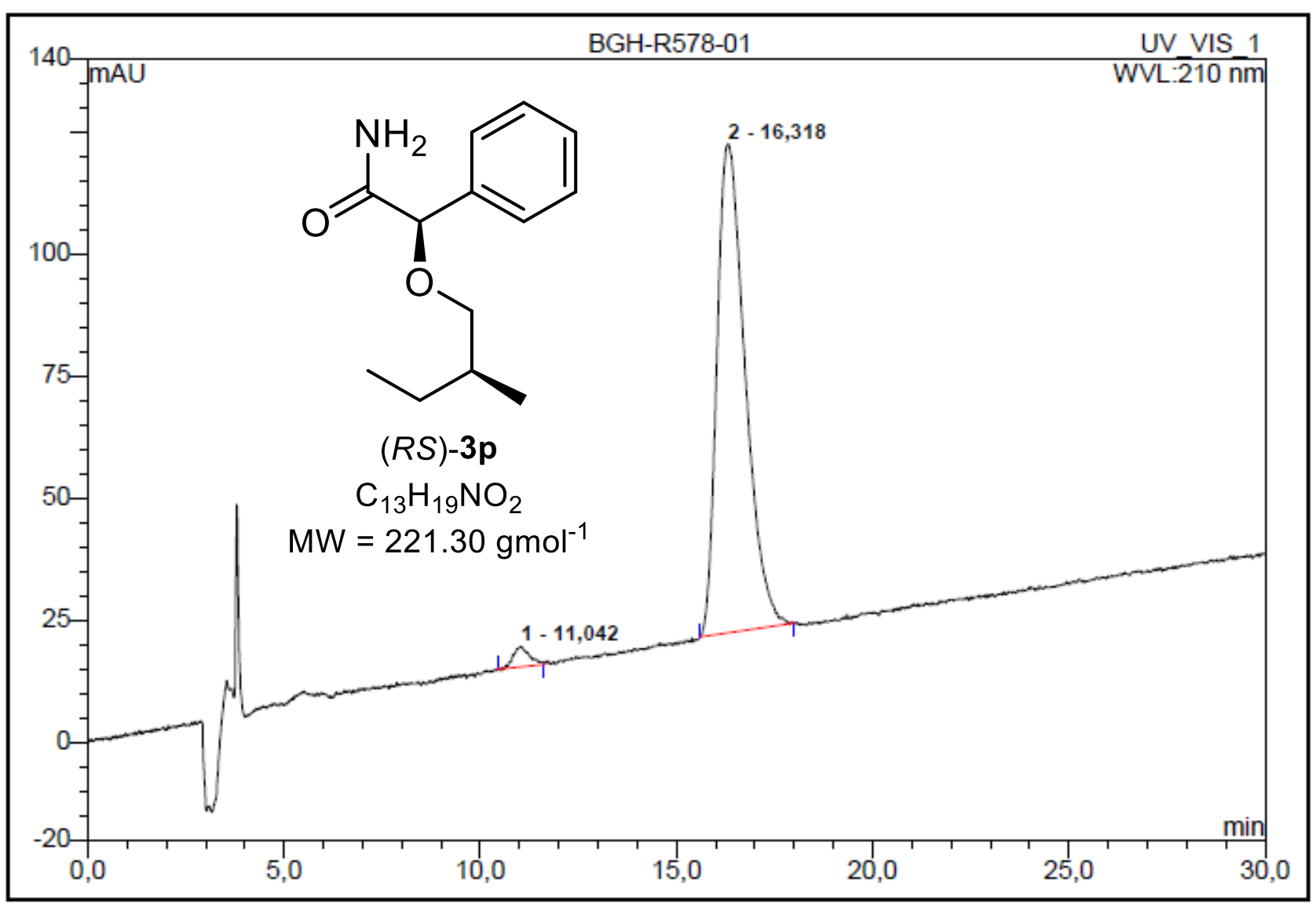

No.	Ret.Time min	Peak Name	Height mAU	Area mAU*min	Rel.Area %	Amount	Type
1	11,04	n.a.	4,150	1,960	2,30	n.a.	BMB*
2	16,32	n.a.	100,066	83,187	97,70	n.a.	BMB*
Total:			104,215	85,147	100,00	0,000	

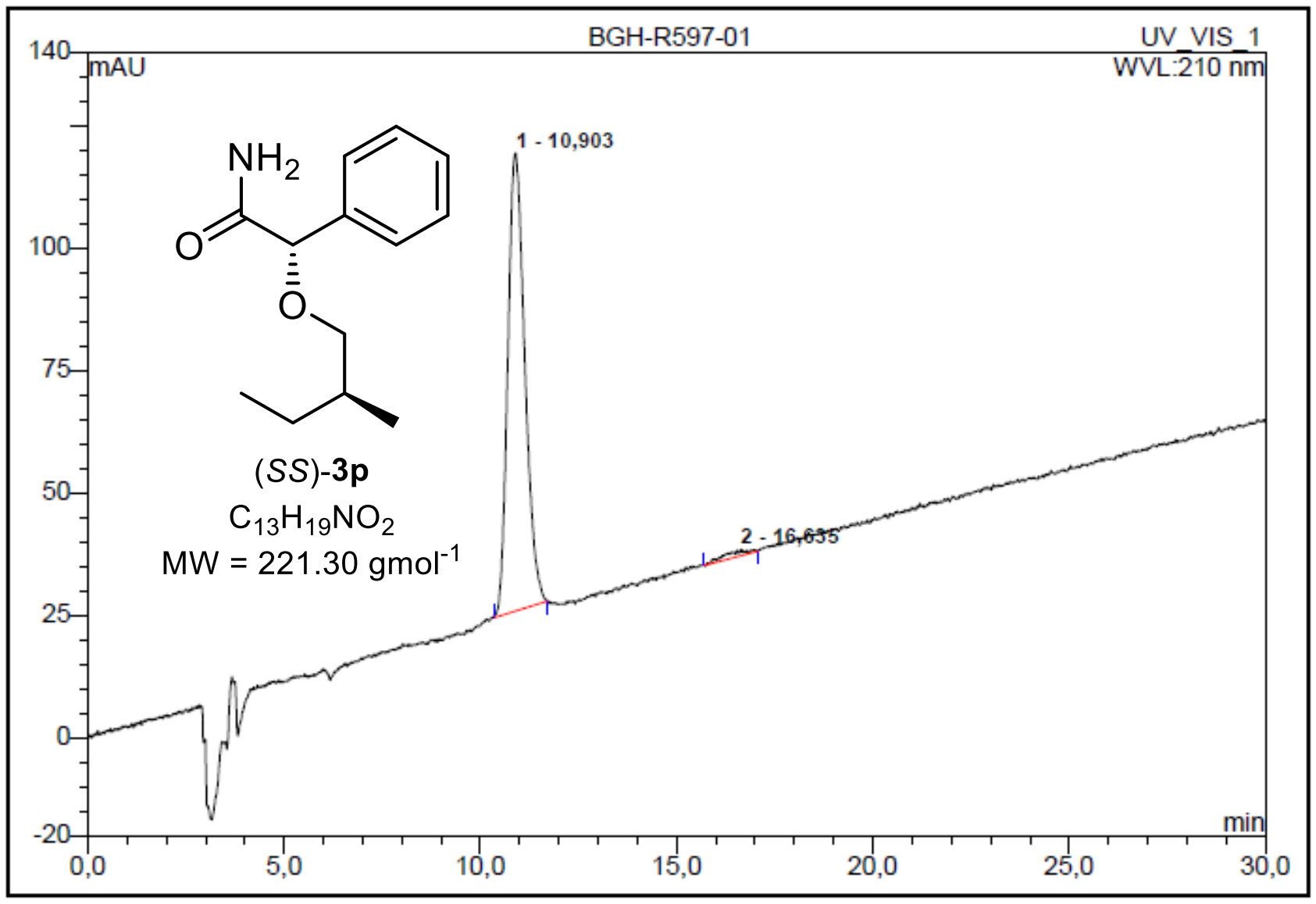

No.	Ret.Time min	Peak Name	Height mAU	Area mAU*min	Rel.Area %	Amount	Type
1	10,90	n.a.	93,405	46,796	98,15	n.a.	BMB
2	16,63	n.a.	1,372	0,882	1,85	n.a.	BMB*
Total:			94,777	47,678	100,00	0,000	

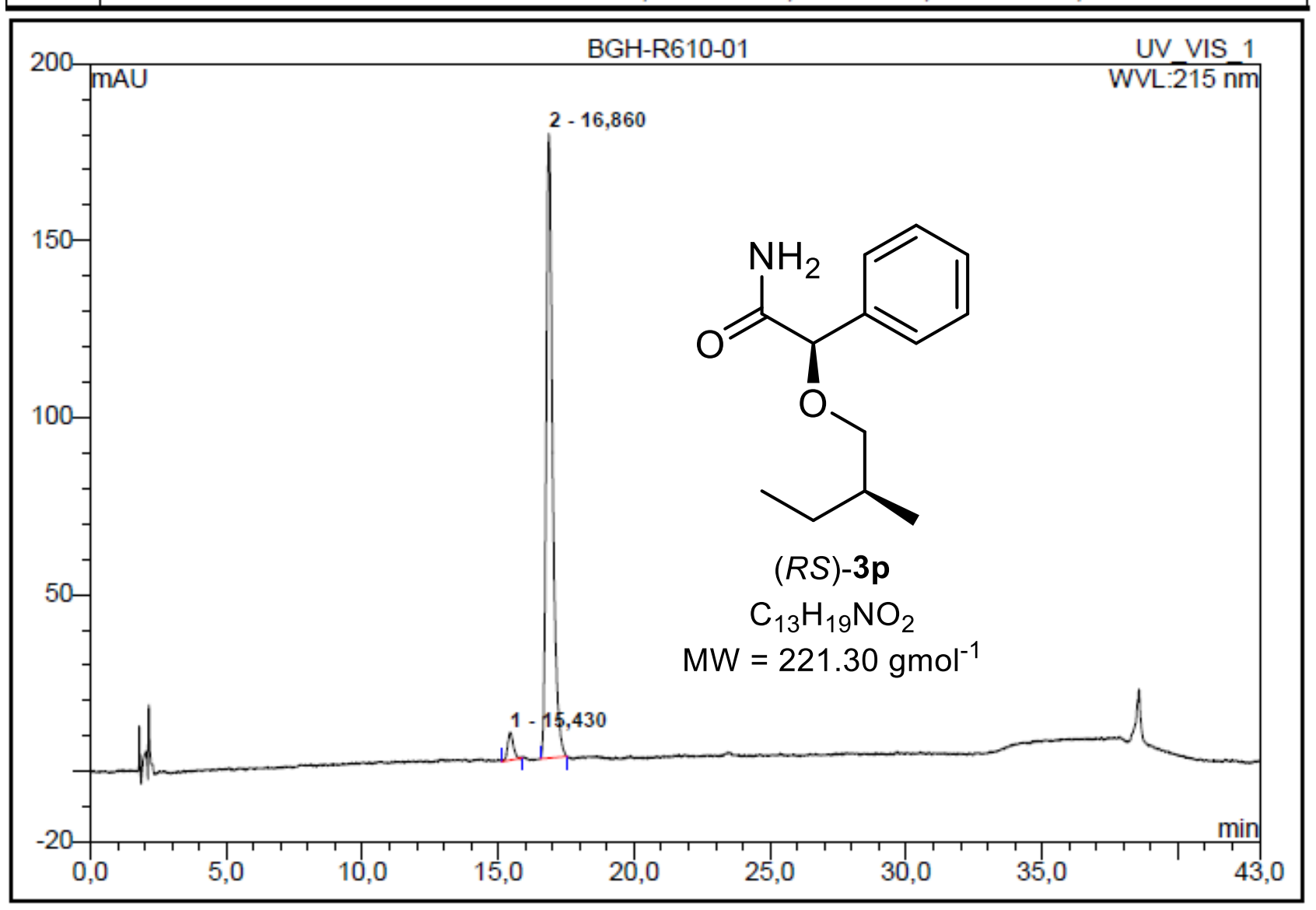

No.	Ret.Time min	Peak Name	Height mAU	Area mAU*min	Rel.Area %	Amount	Type
1	15,43	n.a.	7,876	1,881	3,72	n.a.	BMB*
2	16,86	n.a.	176,448	48,726	96,28	n.a.	BMB
Total:			184,324	50,607	100,00	0,000	

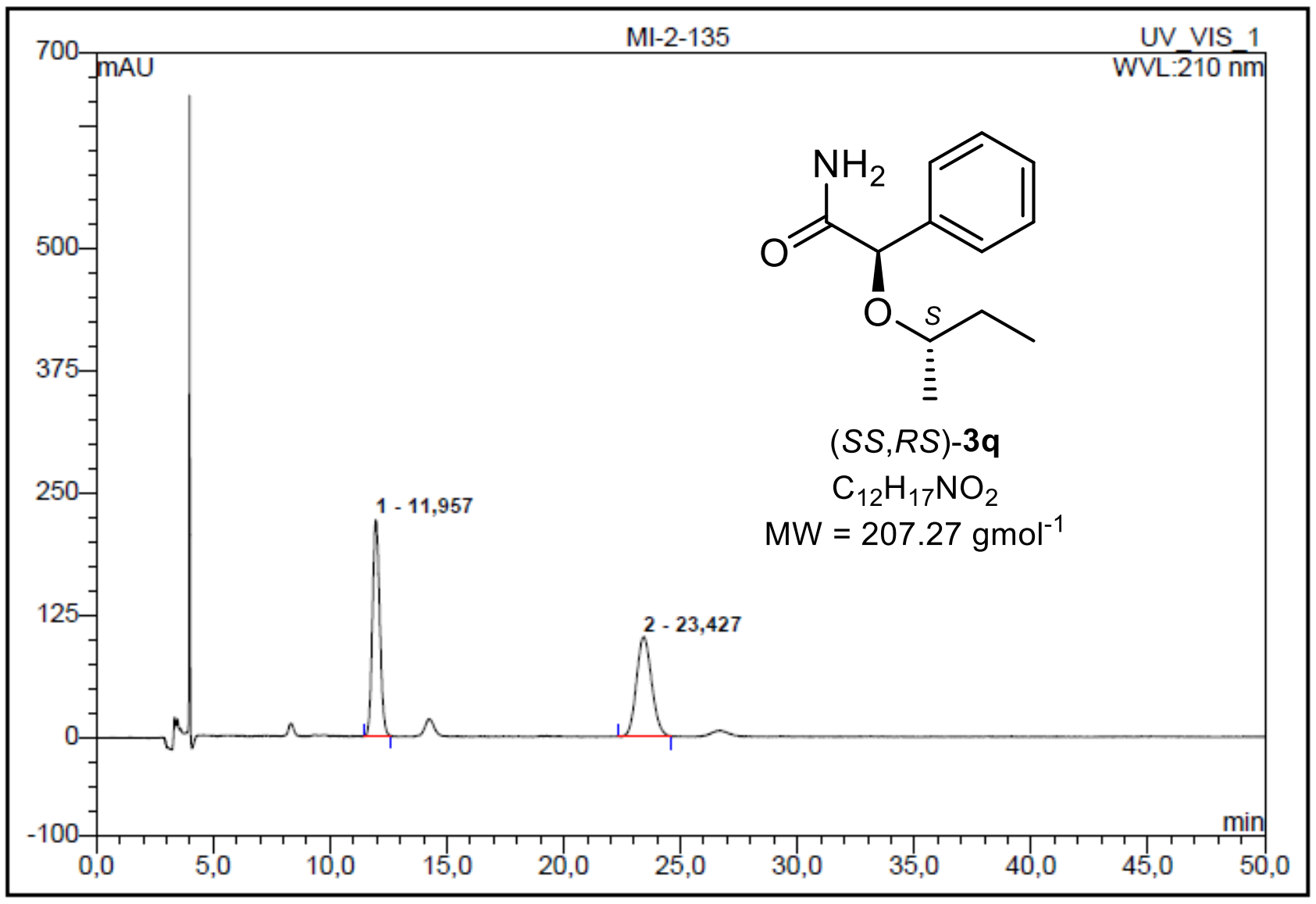

No.	Ret.Time min	Peak Name	Height mAU	Area mAU*min	Rel.Area %	Amount	Type
1	11,96	n.a.	220,893	79,875	51,00	n.a.	BMB
2	23,43	n.a.	101,896	76,750	49,00	n.a.	BMB*
Total:			322,789	156,625	100,00	0,000	

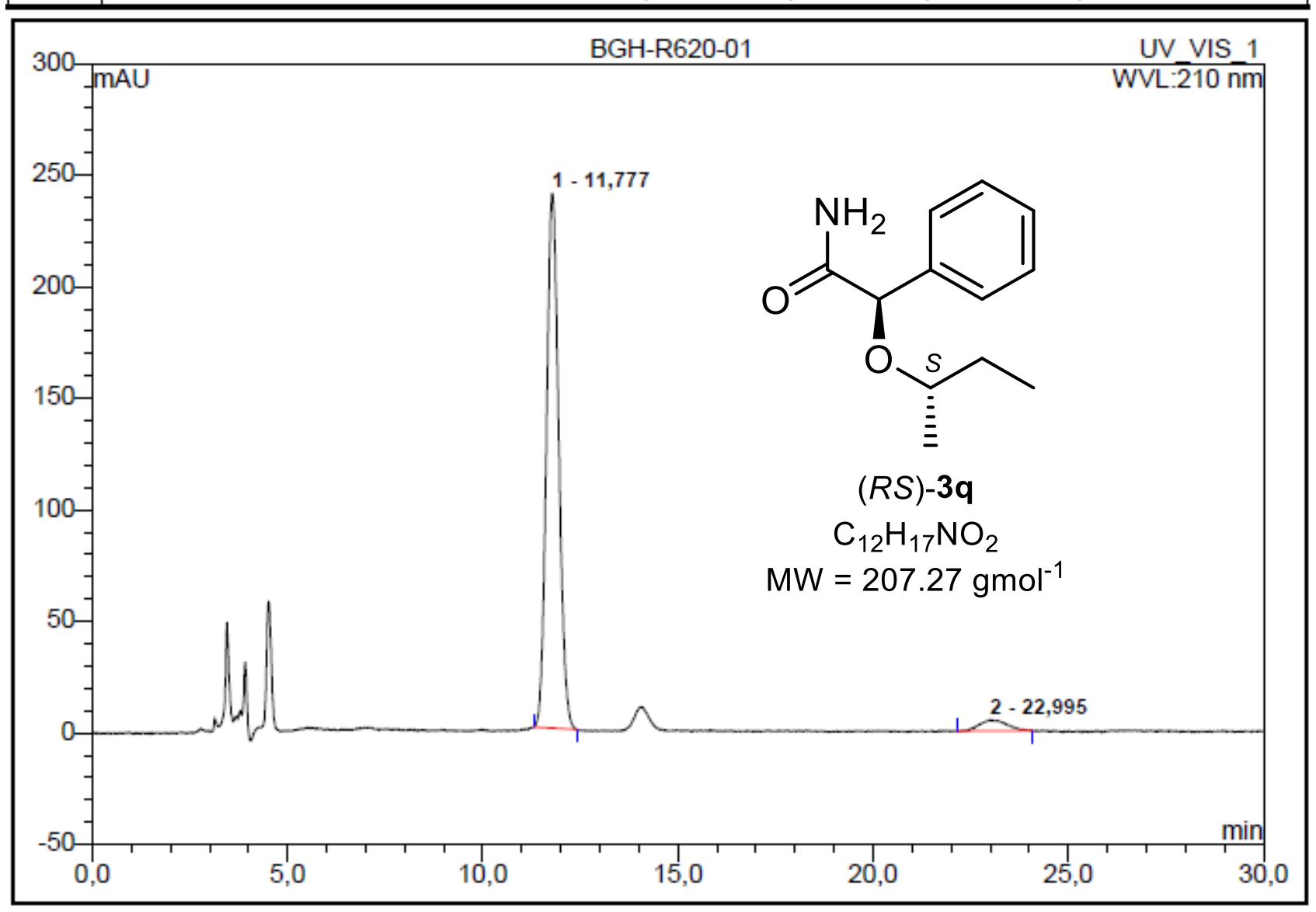

No.	Ret.Time min	Peak Name	Height mAU	Area mAU*min	Rel.Area %	Amount	Type
1	11,78	n.a.	239,844	87,186	95,82	n.a.	BMB
2	22,99	n.a.	4,982	3,802	4,18	n.a.	BMB*
Total:			244,826	90,988	100,00	0,000	

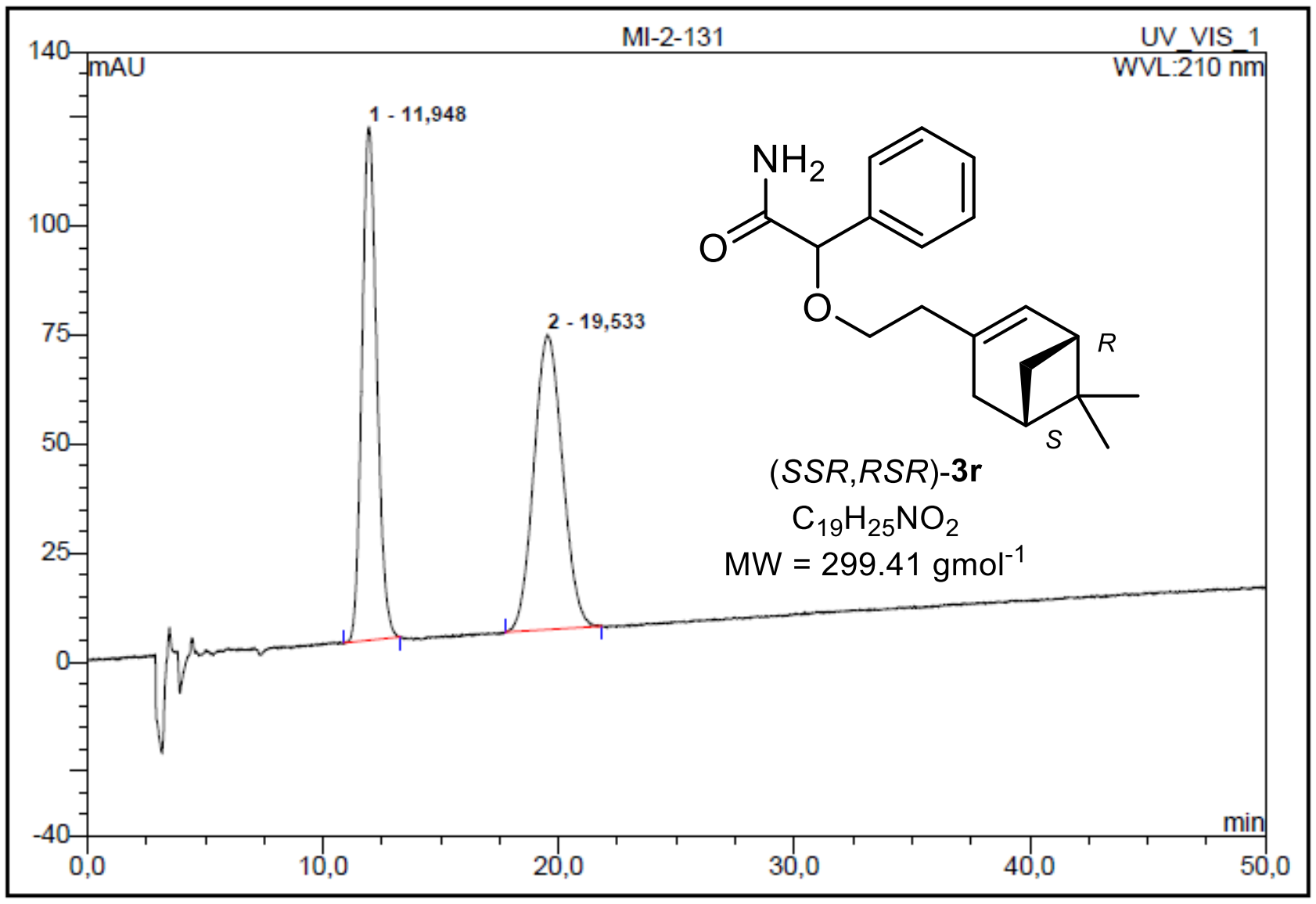

No.	Ret.Time min	Peak Name	Height mAU	Area mAU*min	Rel.Area %	Amount	Type
1	11,95	n.a.	117,837	86,161	47,47	n.a.	BMB*
2	19,53	n.a.	67,737	95,338	52,53	n.a.	BMB*
Total:			185,574	181,499	100,00	0,000	

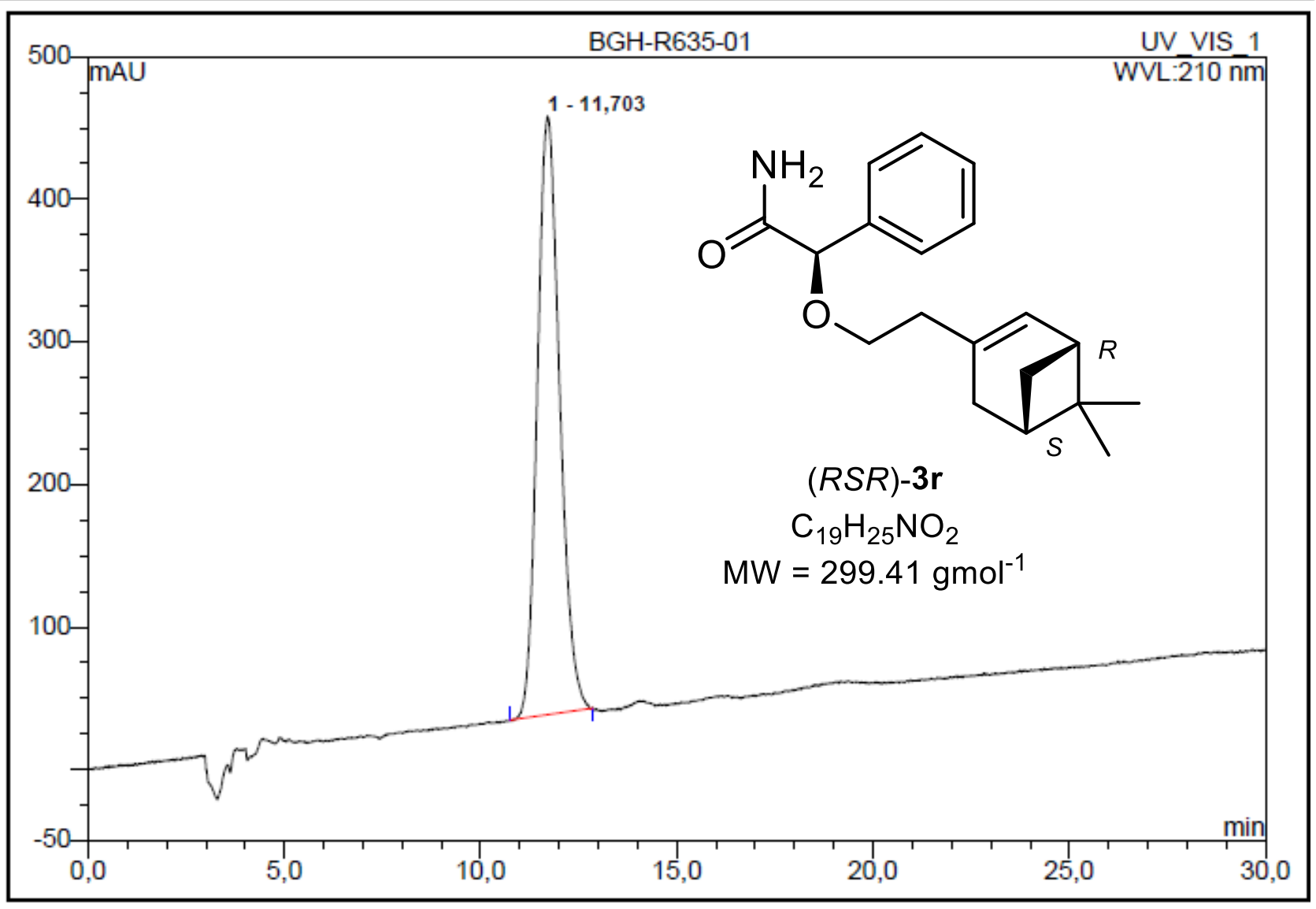

No.	Ret.Time min	Peak Name	Height mAU	Area mAU*min	Rel.Area %	Amount	Type
1	11,70	n.a.	419,929	277,329	100,00	n.a.	BMB*
Total:			419,929	277,329	100,00	0,000	

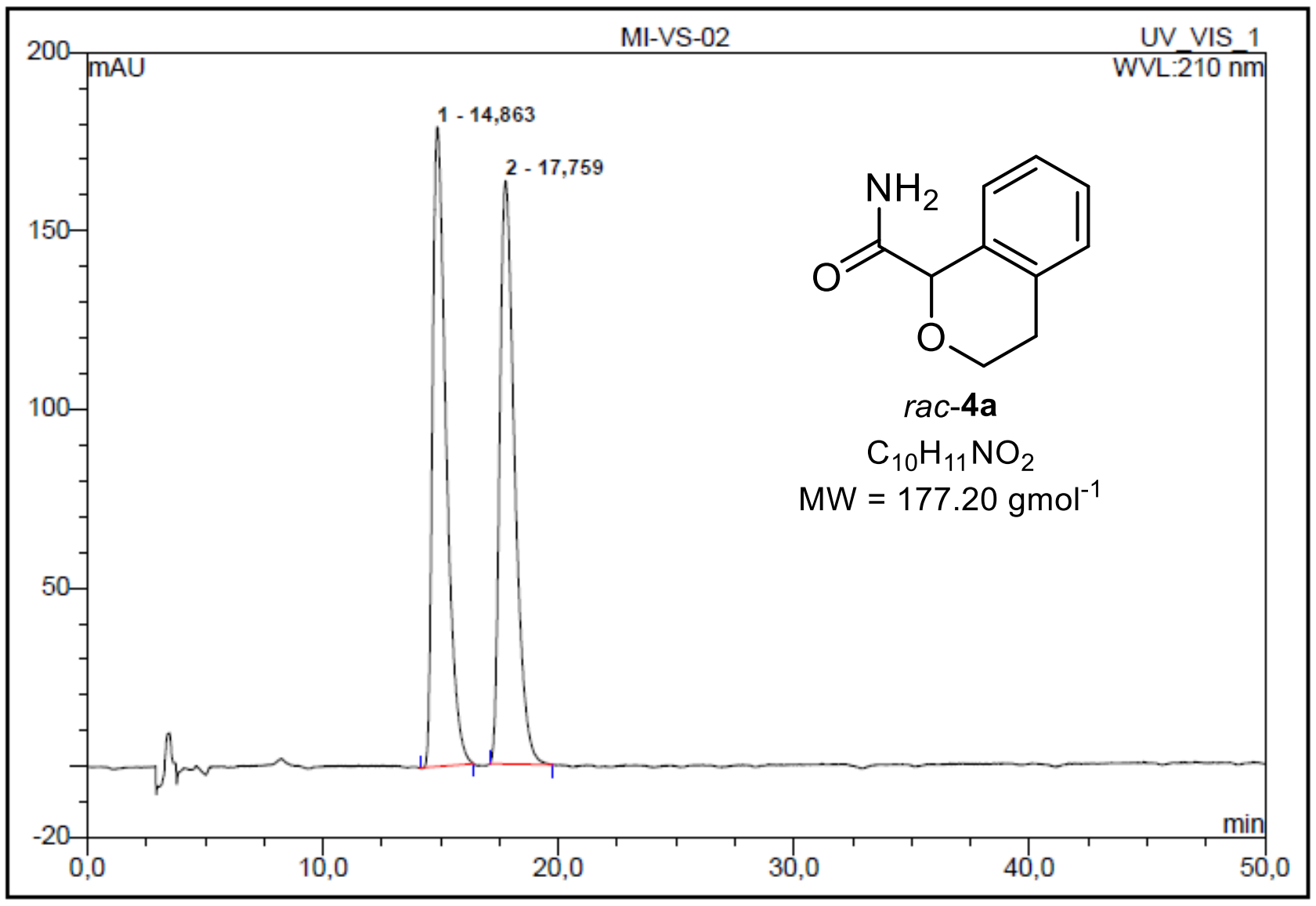

No.	Ret.Time min	Peak Name	Height mAU	Area mAU*min	Rel.Area %	Amount	Type
1	14,86	n.a.	179,226	119,998	49,96	n.a.	BMB*
2	17,76	n.a.	163,283	120,201	50,04	n.a.	BMB*
Total:			342,509	240,198	100,00	0,000	

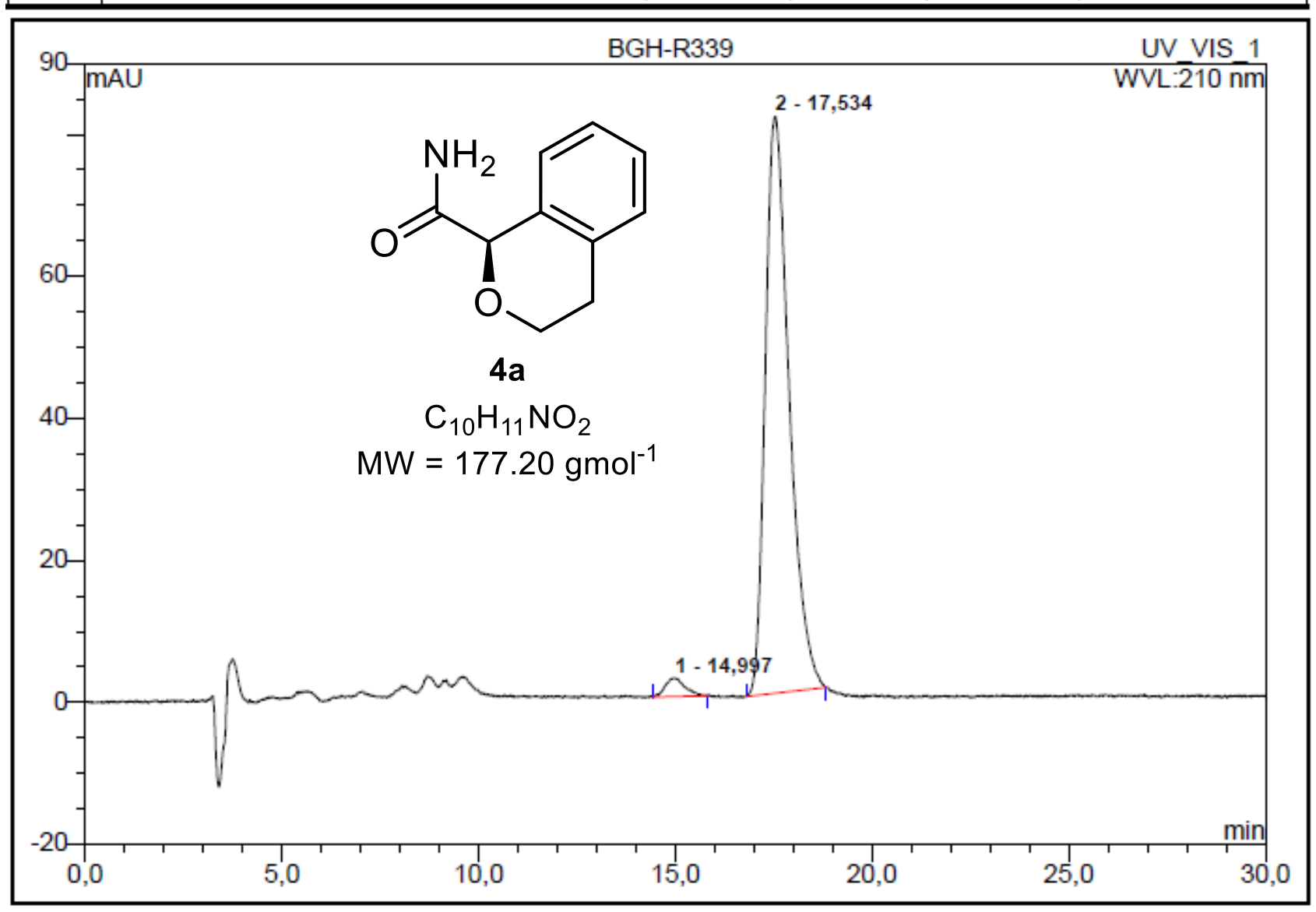

No.	Ret.Time min	Peak Name	Height mAU	Area mAU*min	Rel.Area %	Amount	Type
1	15,00	n.a.	2,621	1,504	2,56	n.a.	BMB*
2	17,53	n.a.	81,265	57,213	97,44	n.a.	BMB
Total:			83,886	58,717	100,00	0,000	

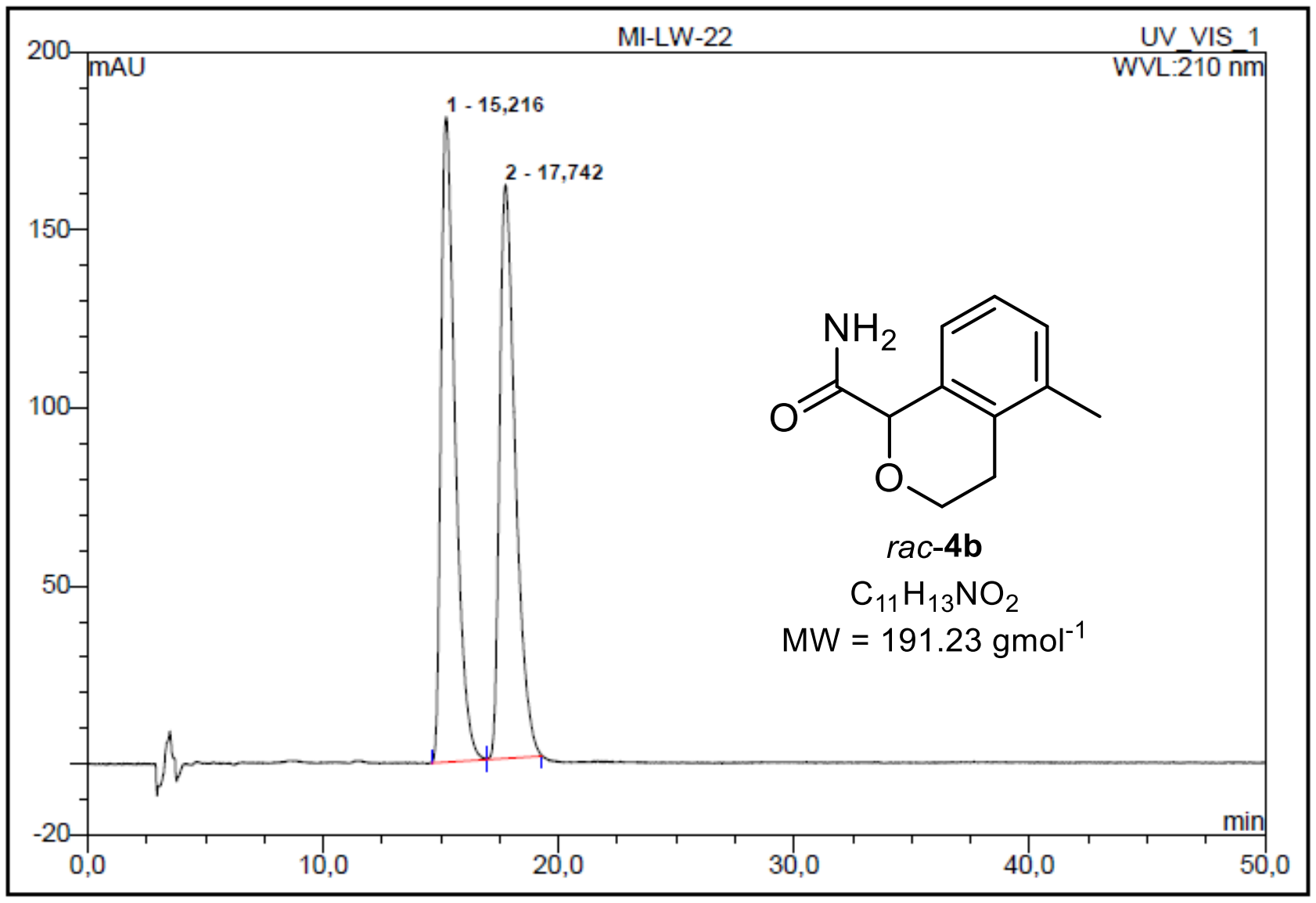

No.	Ret.Time min	Peak Name	Height mAU	Area mAU*min	Rel.Area %	Amount	Type
1	15,22	n.a.	181,295	127,269	50,37	n.a.	BM
2	17,74	n.a.	161,128	125,404	49,63	n.a.	MB
Total:			342,423	252,674	100,00	0,000	

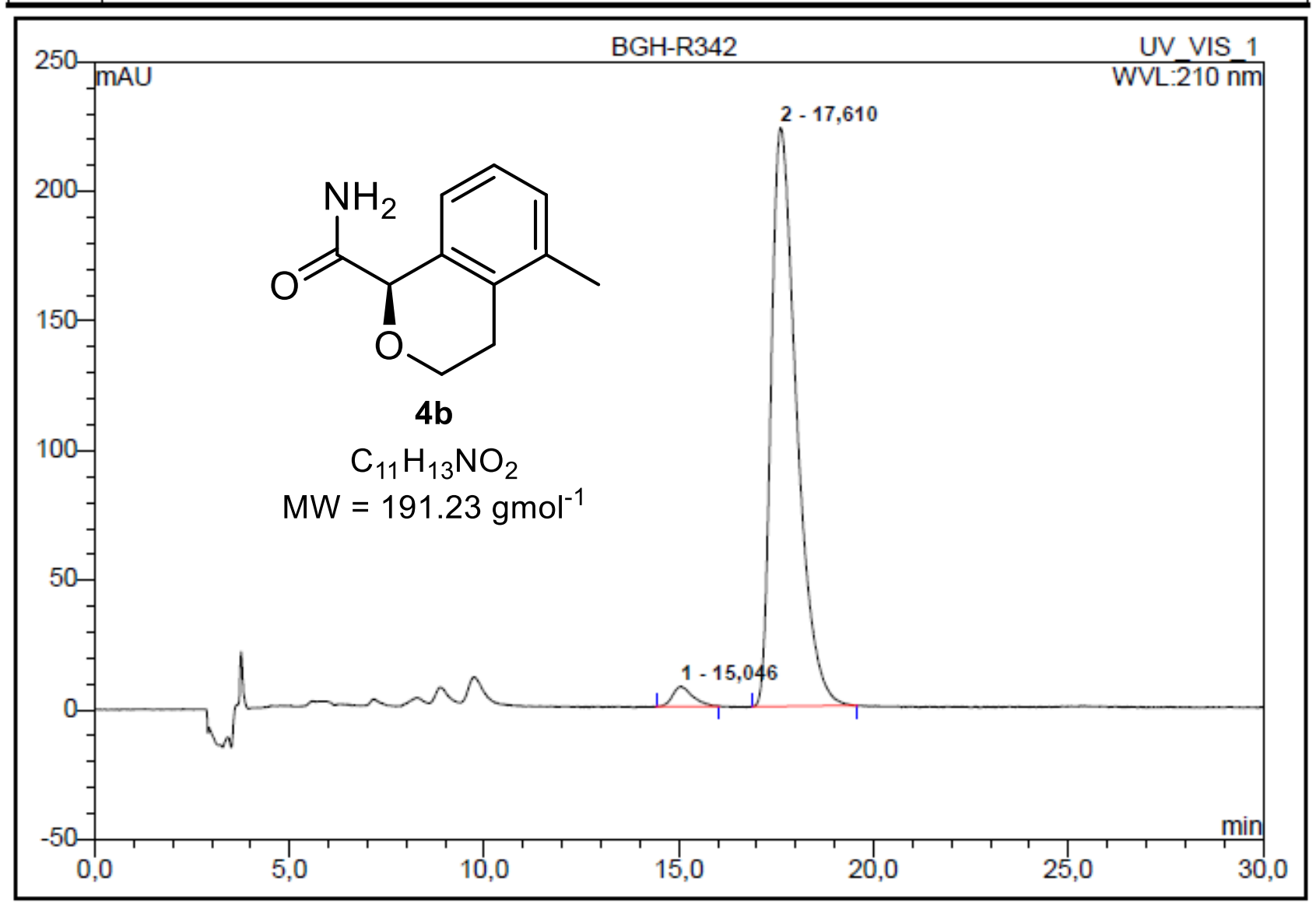

No.	Ret.Time min	Peak Name	Height mAU	Area mAU*min	Rel.Area %	Amount	Type
1	15,05	n.a.	7,666	4,596	2,66	n.a.	BMB*
2	17,61	n.a.	223,181	168,328	97,34	n.a.	BMB*
Total:			230,847	172,924	100,00	0,000	

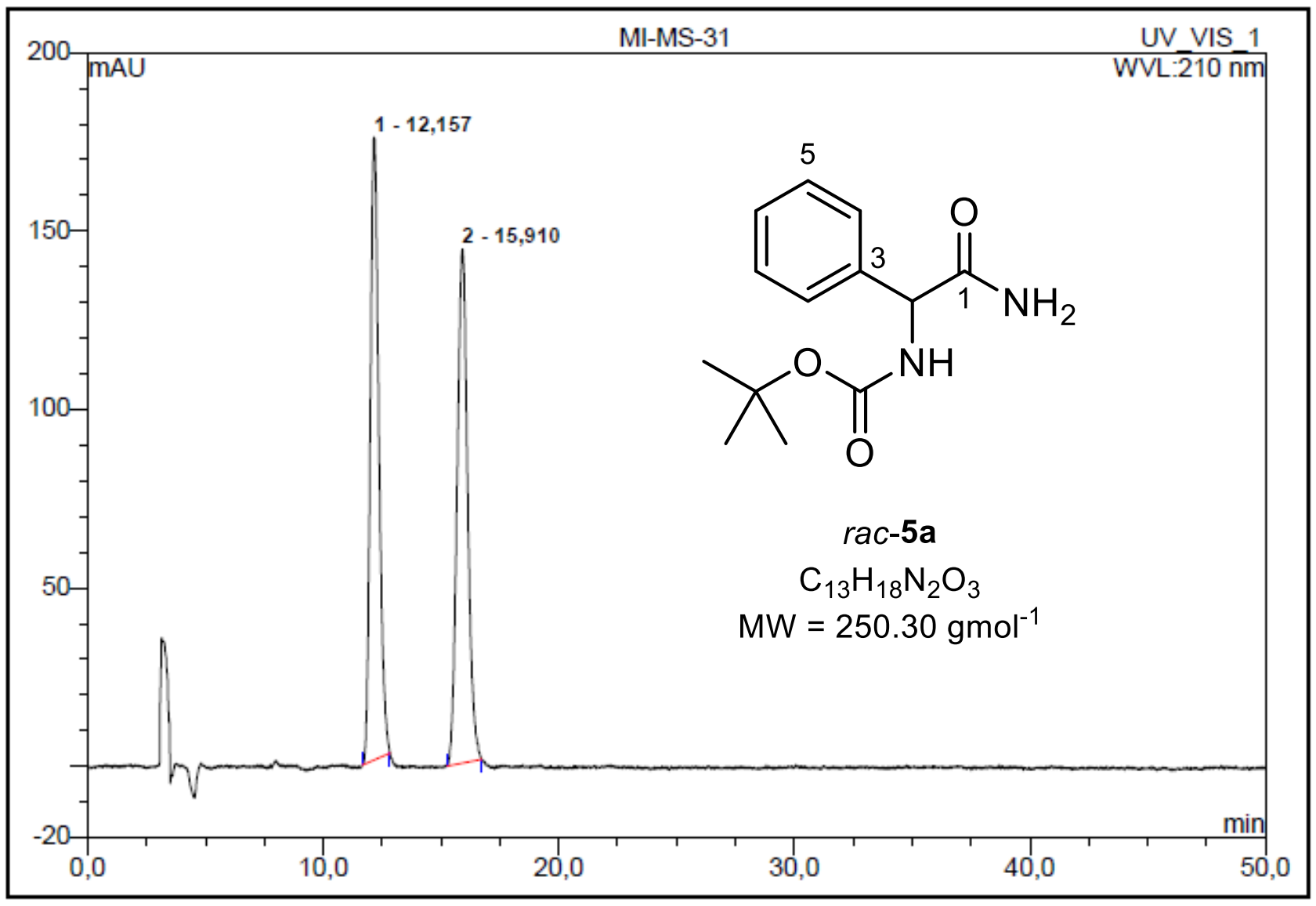

No.	Ret.Time min	Peak Name	Height mAU	Area mAU*min	Rel.Area %	Amount	Type
1	12,16	n.a.	174,579	73,678	49,69	n.a.	BMB
2	15,91	n.a.	143,976	74,608	50,31	n.a.	BMB
Total:			318,554	148,287	100,00	0,000	

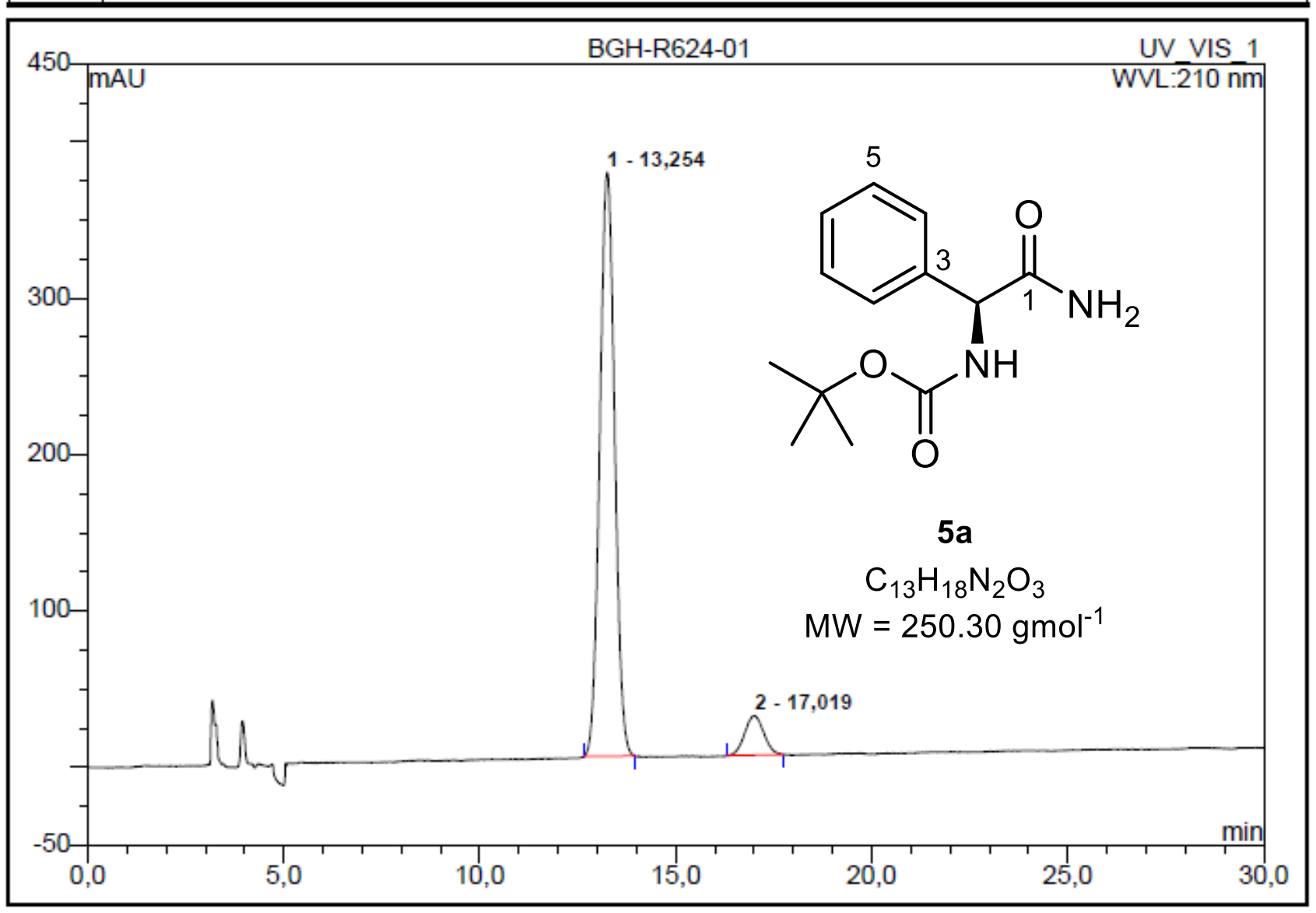

No.	Ret.Time min	Peak Name	Height mAU	Area mAU*min	Rel.Area %	Amount	Type
1	13,25	n.a.	373,242	164,486	92,03	n.a.	BMB
2	17,02	n.a.	25,228	14,239	7,97	n.a.	BMB*
Total:			398,470	178,725	100,00	0,000	

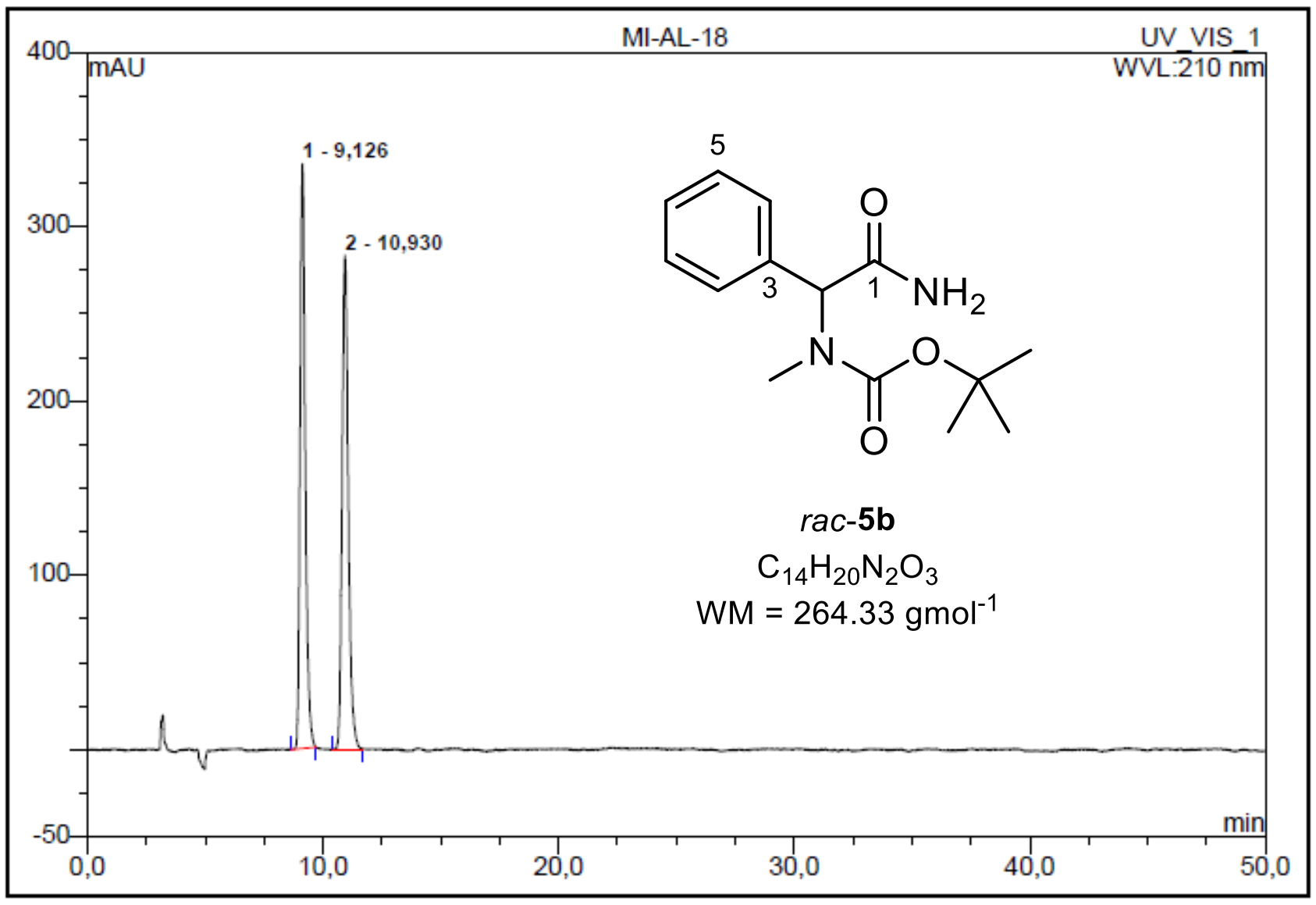

No.	Ret.Time min	Peak Name	Height mAU	Area mAU*min	Rel.Area %	Amount	Type
1	9,13	n.a.	335,520	85,910	49,72	n.a.	BMB*
2	10,93	n.a.	283,500	86,874	50,28	n.a.	BMB*
Total:			619,019	172,784	100,00	0,000	

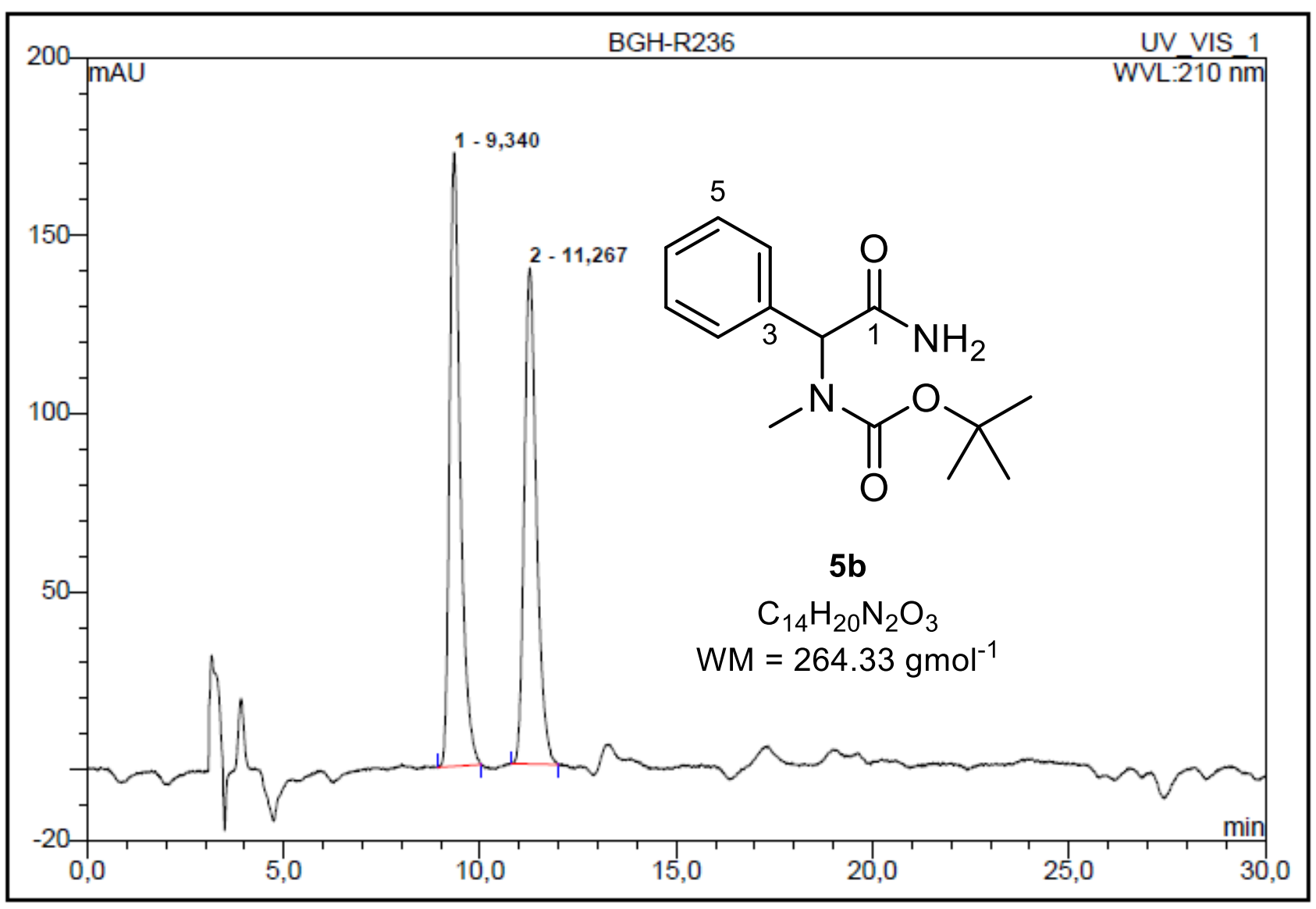

No.	Ret.Time min	Peak Name	Height mAU	Area mAU*min	Rel.Area %	Amount	Type
1	9,34	n.a.	172,393	54,551	52,15	n.a.	BMB*
2	11,27	n.a.	139,324	50,050	47,85	n.a.	BMB*
Total:			311,717	104,601	100,00	0,000	

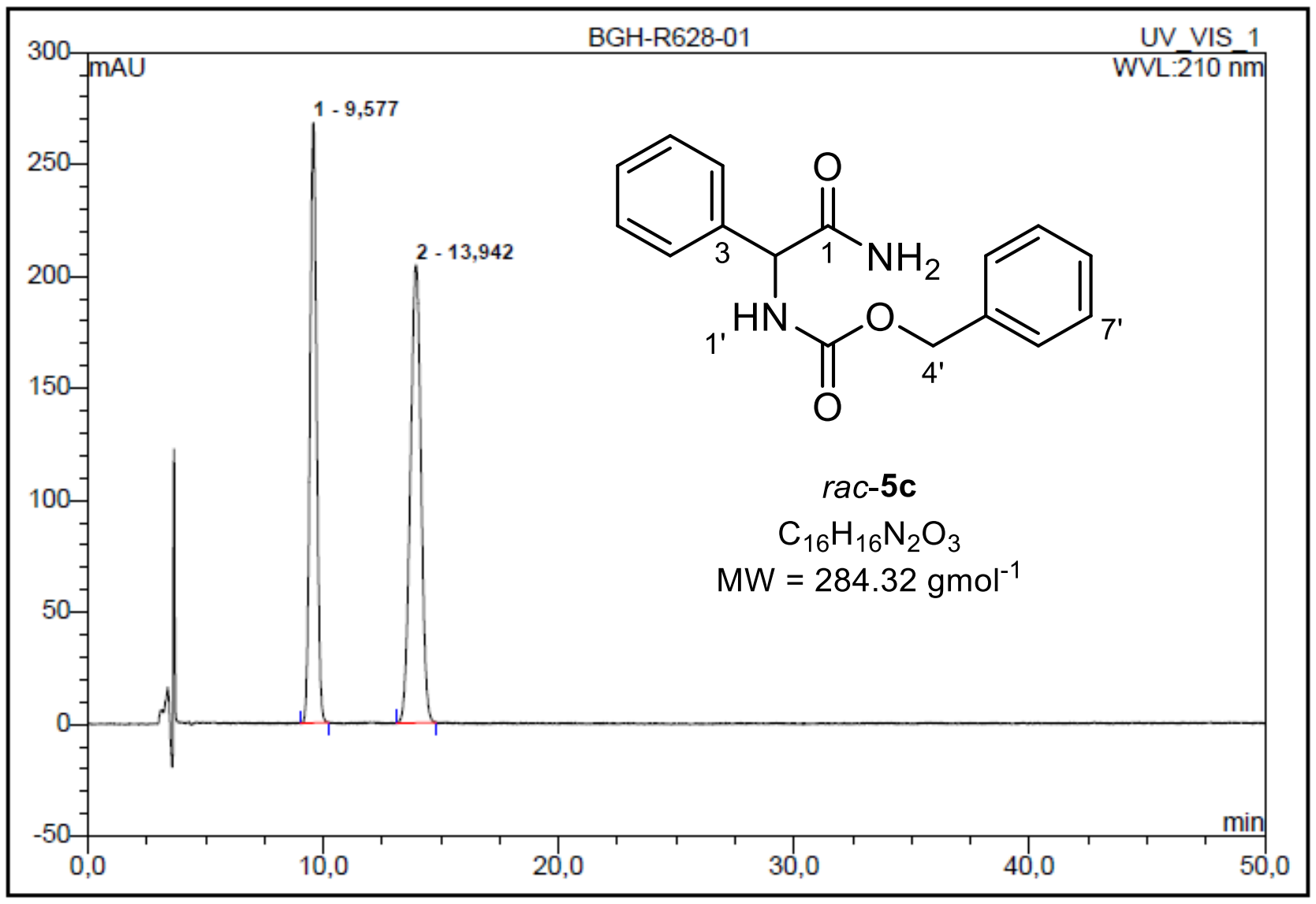

No.	Ret.Time min	Peak Name	Height mAU	Area mAU*min	Rel.Area %	Amount	Type
1	9,58	n.a.	268,133	87,614	44,95	n.a.	BMB*
2	13,94	n.a.	204,470	107,290	55,05	n.a.	BMB*
Total:			472,603	194,904	100,00	0,000	

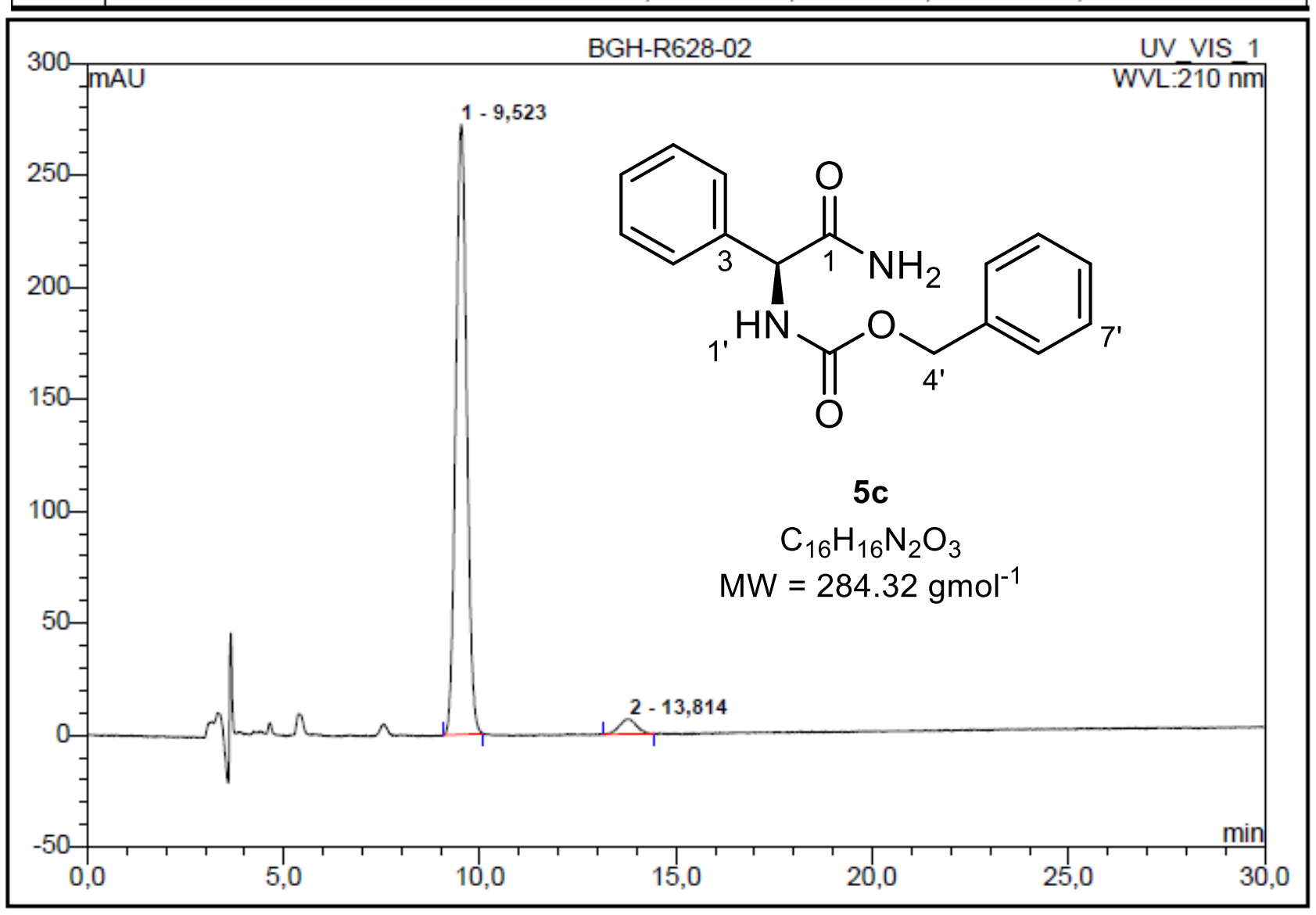

No.	Ret.Time min	Peak Name	Height mAU	Area mAU*min	Rel.Area %	Amount	Type
1	9,52	n.a.	272,417	88,094	96,23	n.a.	BMB
2	13,81	n.a.	6,727	3,449	3,77	n.a.	BMB*
Total:			279,144	91,544	100,00	0,000	

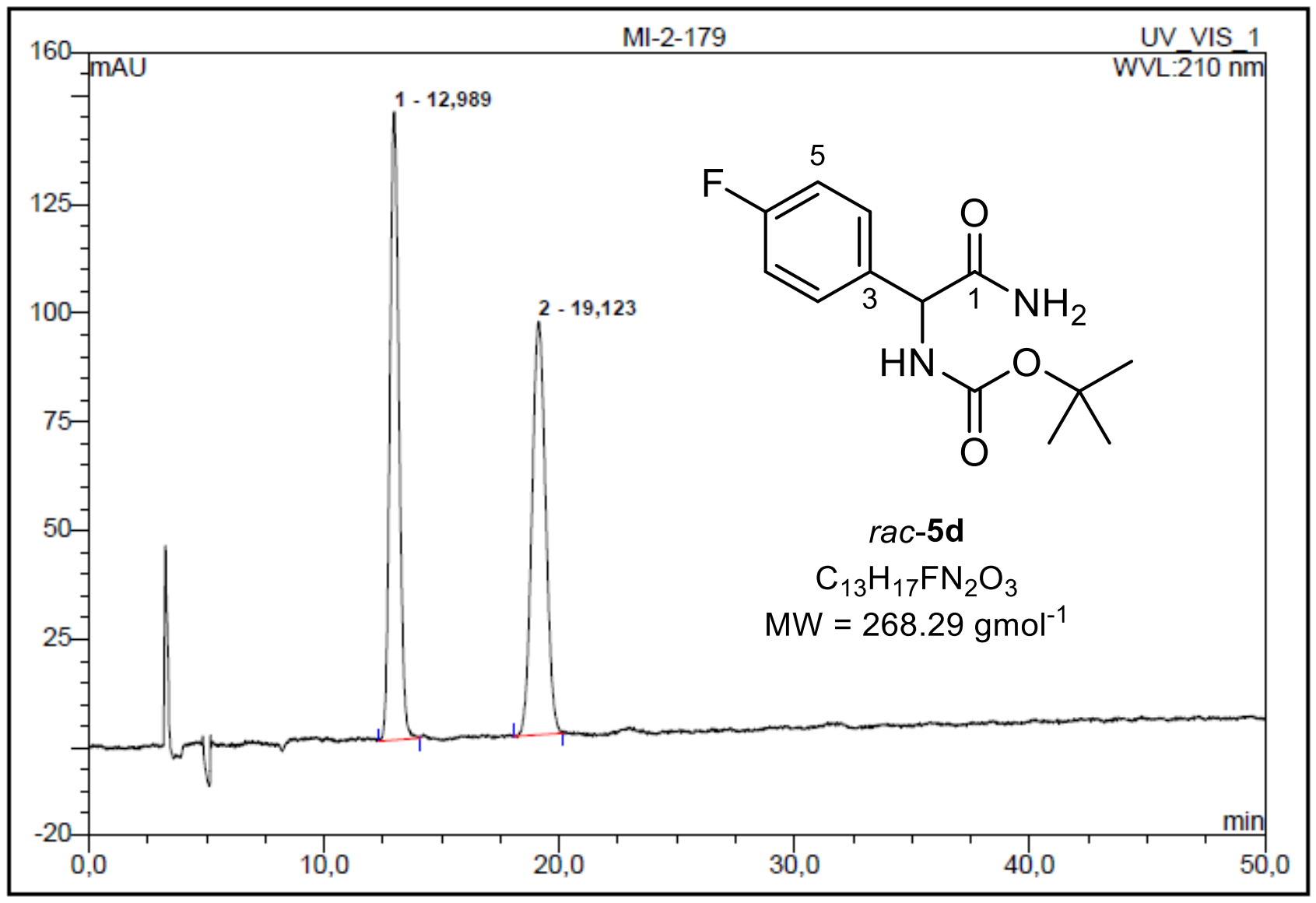

No.	Ret.Time min	Peak Name	Height mAU	Area mAU*min	Rel.Area %	Amount	Type
1	12,99	n.a.	144,568	64,791	50,28	n.a.	BMB*
2	19,12	n.a.	95,053	64,063	49,72	n.a.	BMB*
Total:			239,621	128,854	100,00	0,000	

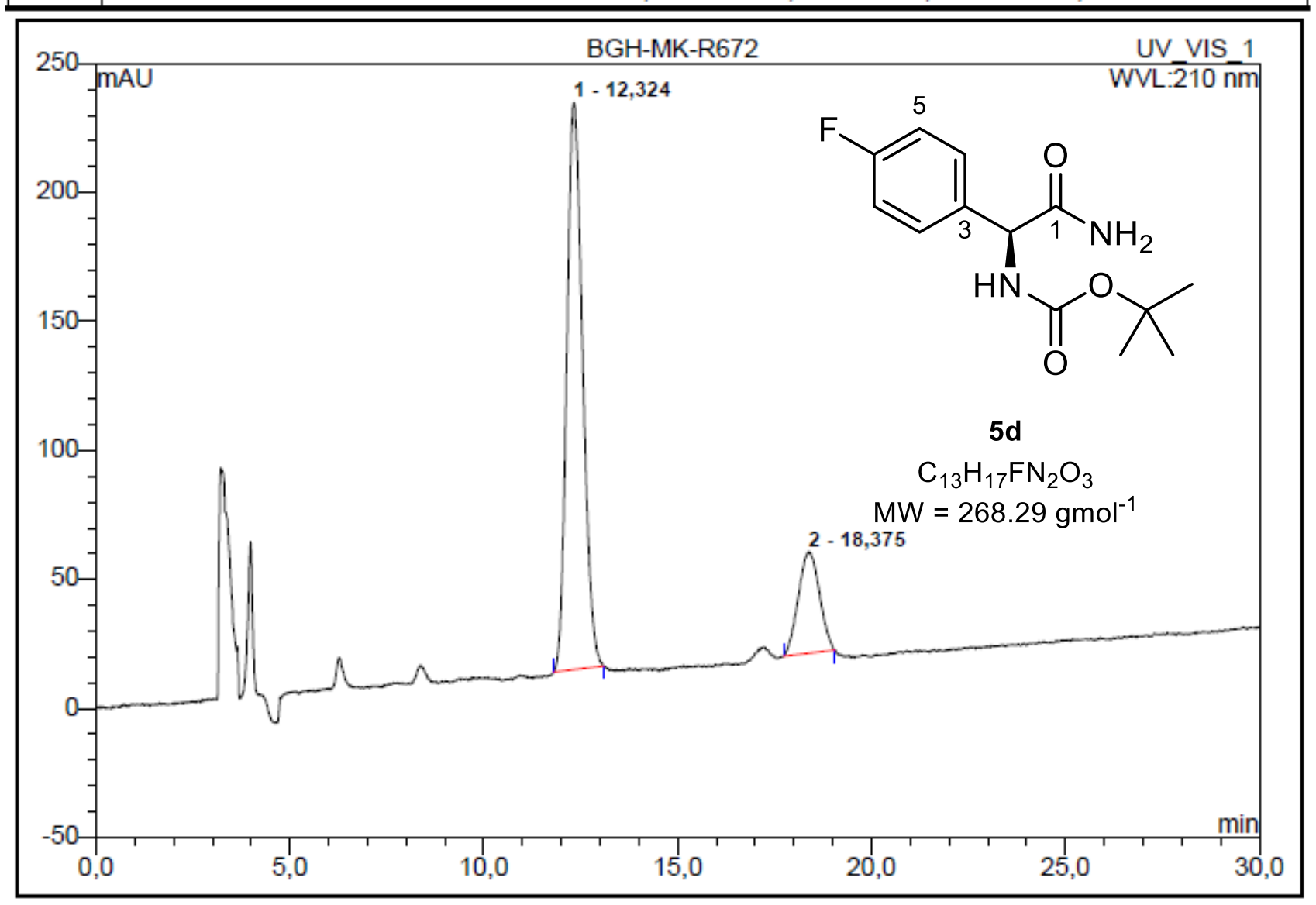

No.	Ret.Time min	Peak Name	Height mAU	Area mAU*min	Rel.Area %	Amount	Type
1	12,32	n.a.	219,681	103,949	80,81	n.a.	BMB
2	18,38	n.a.	39,251	24,688	19,19	n.a.	BMB
Total:			258,932	128,637	100,00	0,000	

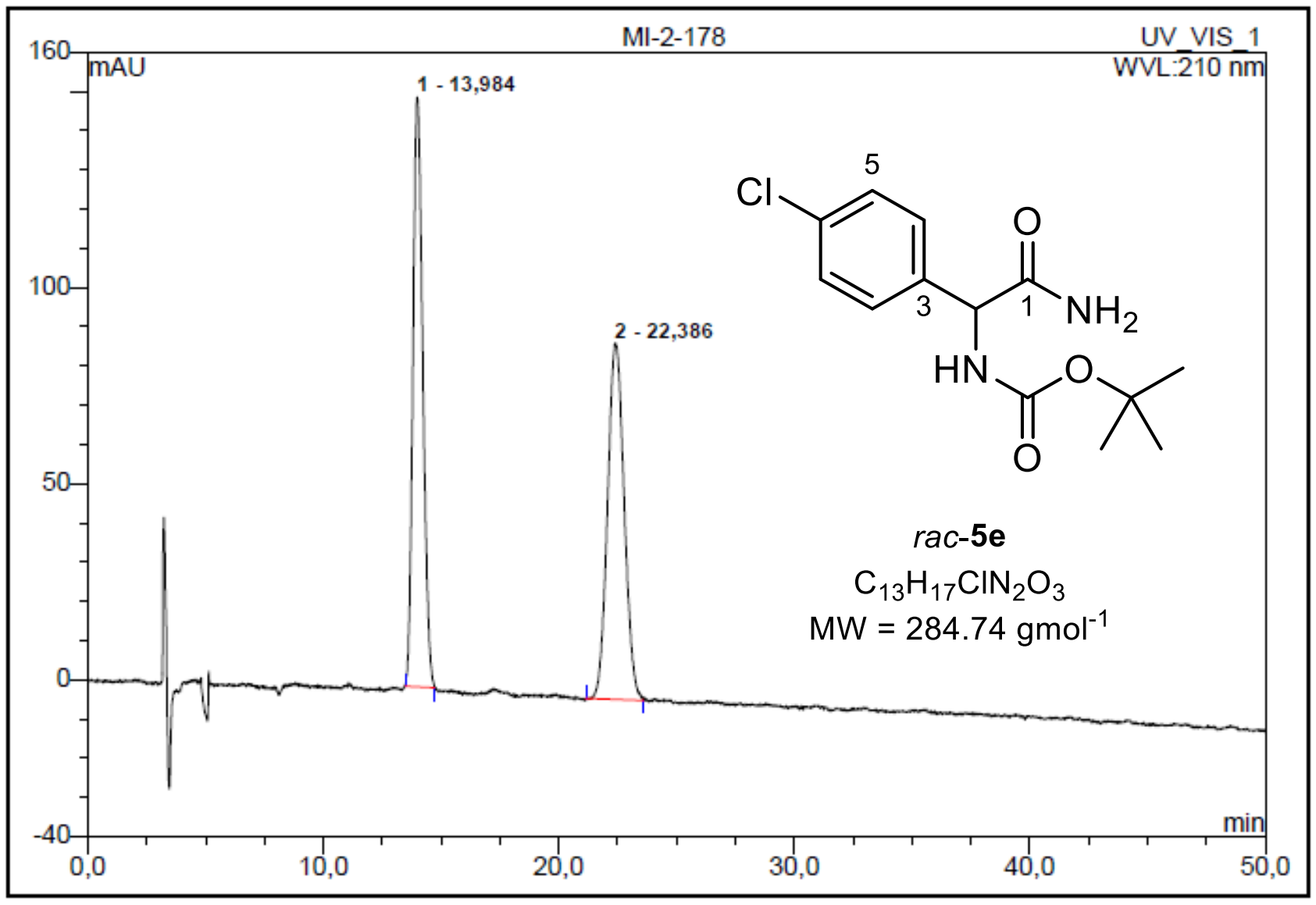

No.	Ret.Time min	Peak Name	Height mAU	Area mAU*min	Rel.Area %	Amount	Type
1	13,98	n.a.	150,284	73,136	49,63	n.a.	BMB
2	22,39	n.a.	90,865	74,235	50,37	n.a.	BMB*
Total:			241,149	147,371	100,00	0,000	

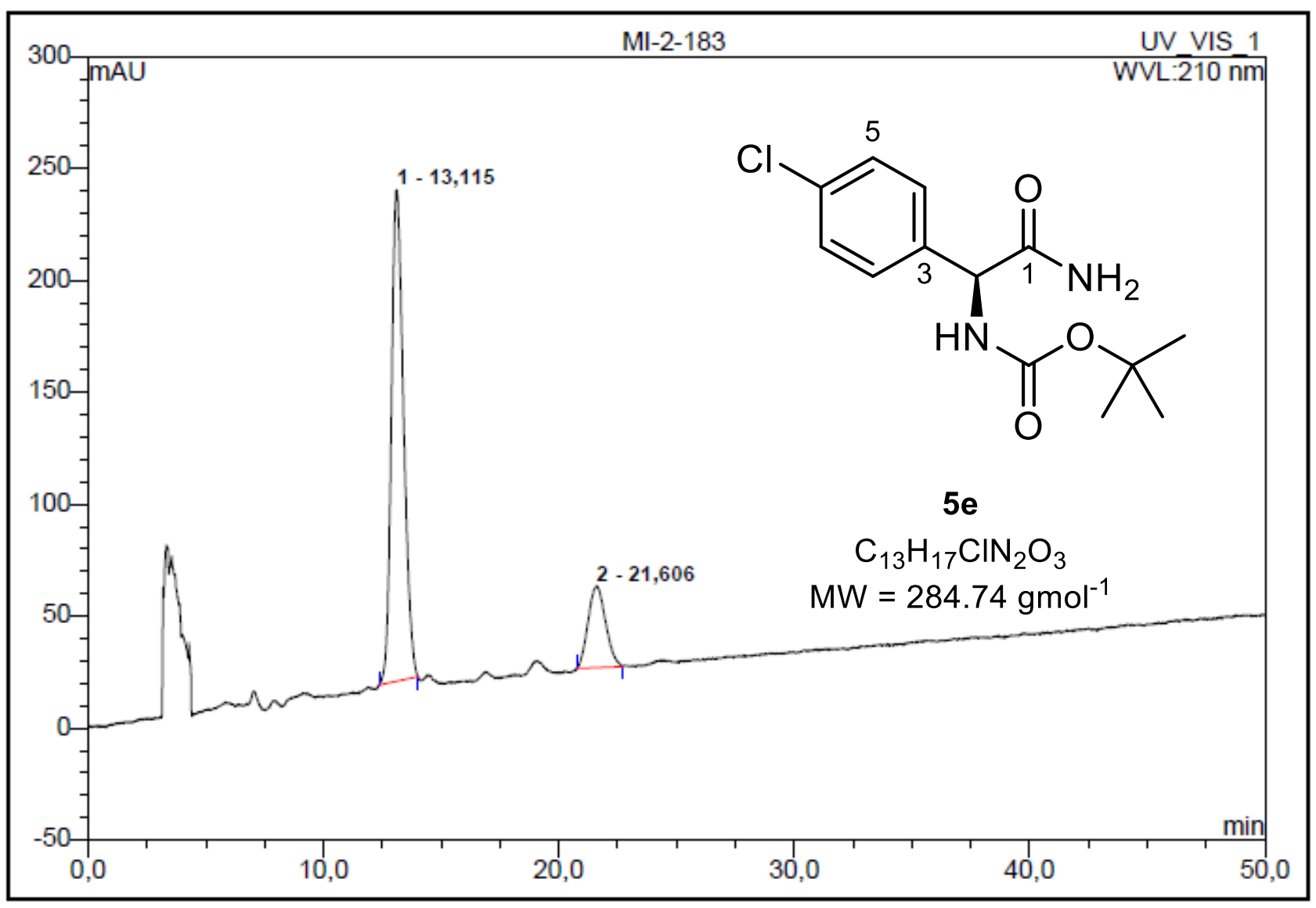

No.	Ret.Time min	Peak Name	Height mAU	Area mAU*min	Rel.Area %	Amount	Type
1	13,11	n.a.	219,544	131,232	80,70	n.a.	BMB
2	21,61	n.a.	36,424	31,394	19,30	n.a.	BMB
Total:			255,968	162,626	100,00	0,000	

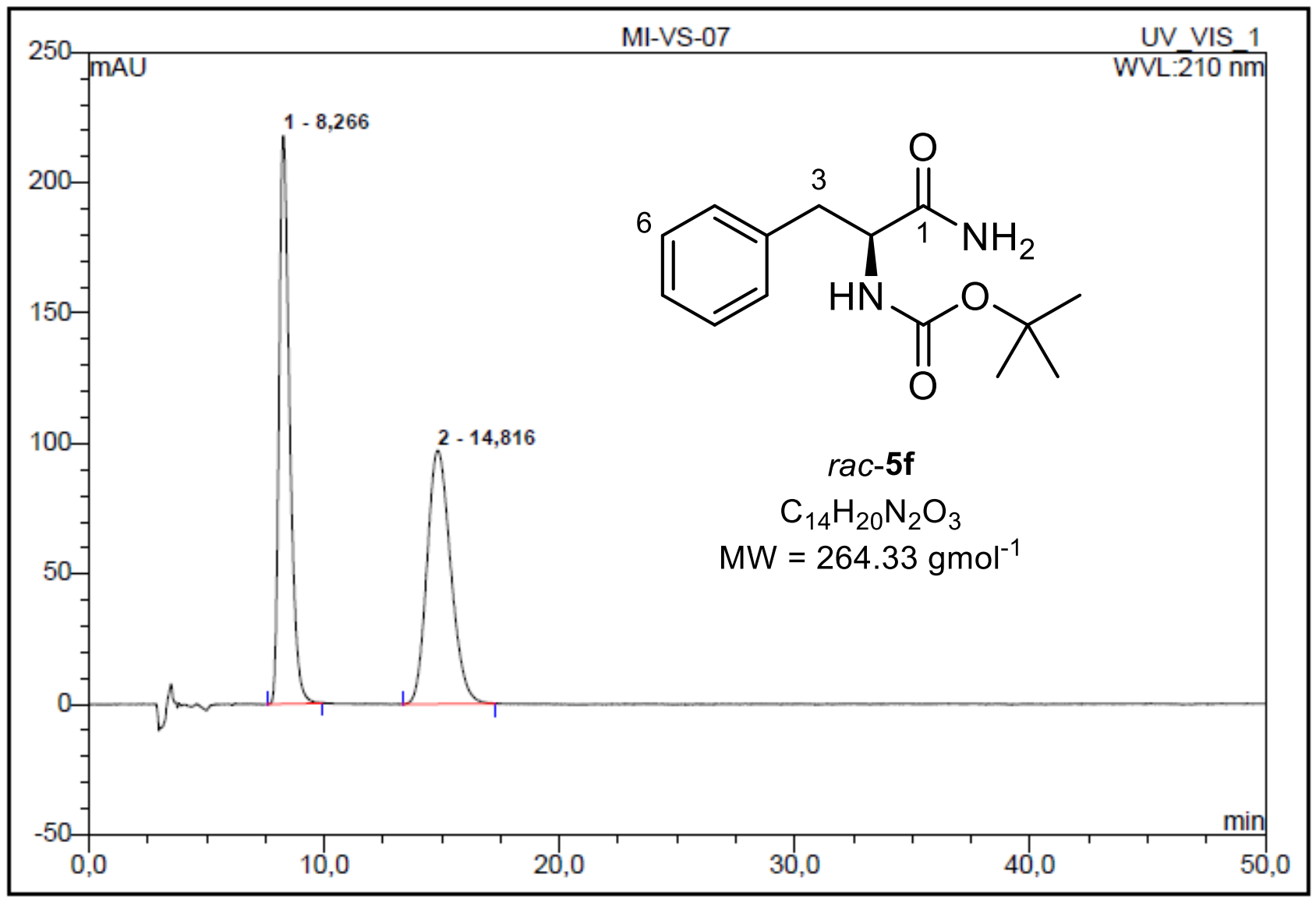

No.	Ret.Time min	Peak Name	Height mAU	Area mAU*min	Rel.Area %	Amount	Type
1	8,27	n.a.	217,879	113,859	50,09	n.a.	BMB*
2	14,82	n.a.	97,178	113,441	49,91	n.a.	BMB*
Total:			315,057	227,300	100,00	0,000	

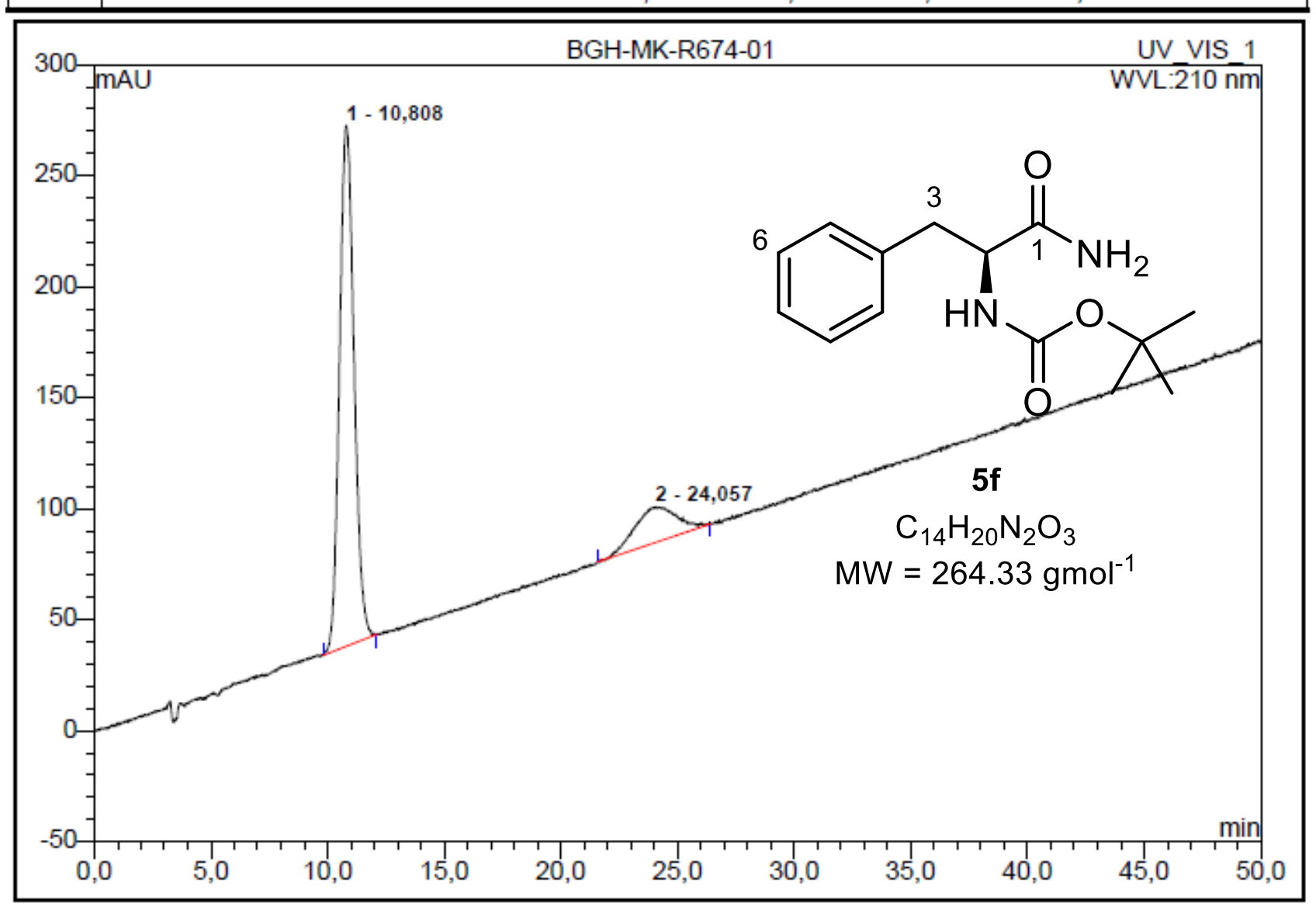

No.	Ret.Time min	Peak Name	Height mAU	Area mAU*min	Rel.Area %	Amount	Type
1	10,81	n.a.	234,650	169,700	83,70	n.a.	BMB*
2	24,06	n.a.	16,003	33,054	16,30	n.a.	BMB*
Total:			250,653	202,753	100,00	0,000	

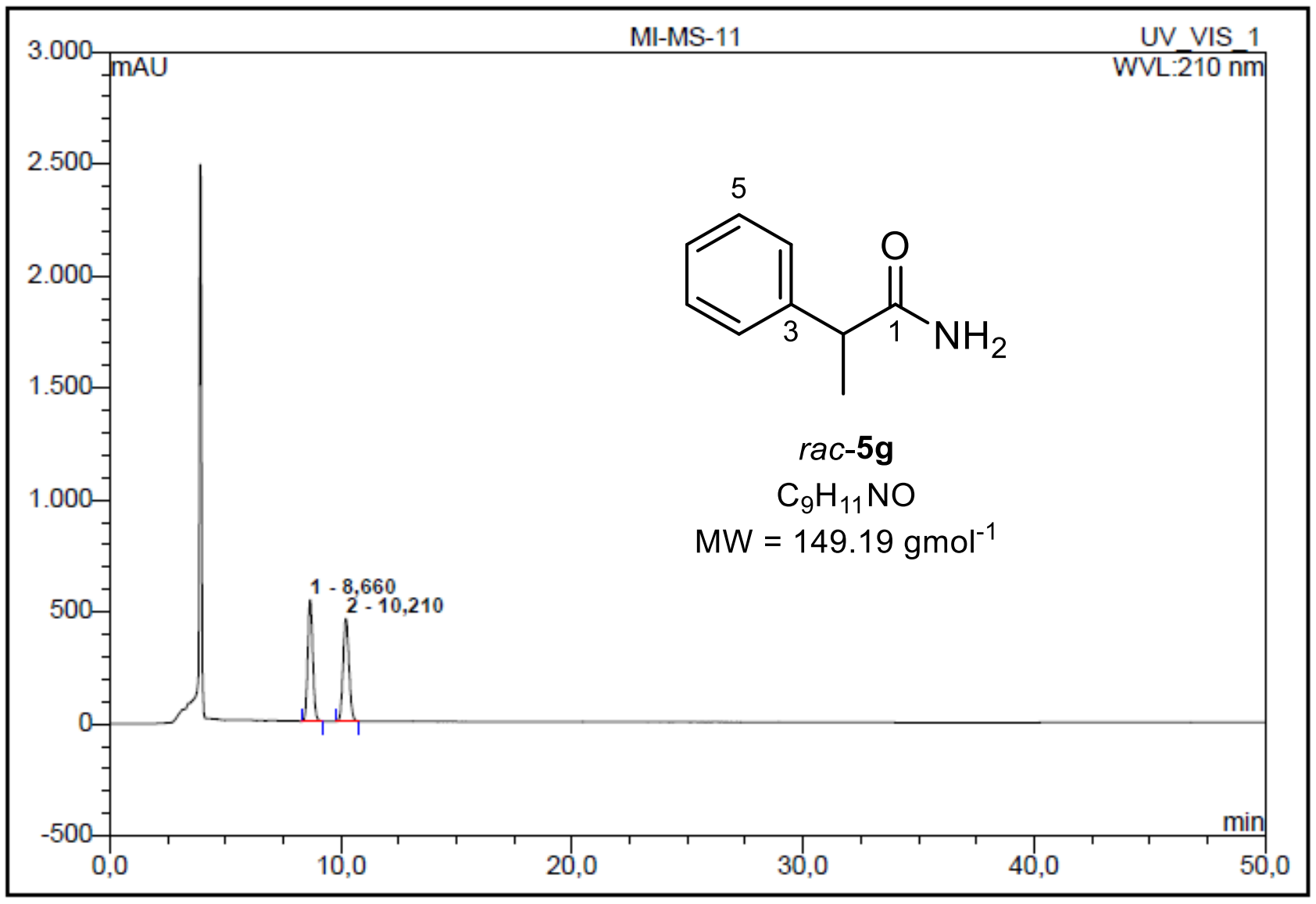

No.	Ret.Time min	Peak Name	Height mAU	Area mAU*min	Rel.Area %	Amount	Type
1	8,66	n.a.	539,596	137,017	50,06	n.a.	BMB
2	10,21	n.a.	457,355	136,673	49,94	n.a.	BMB
Total:			996,951	273,690	100,00	0,000	

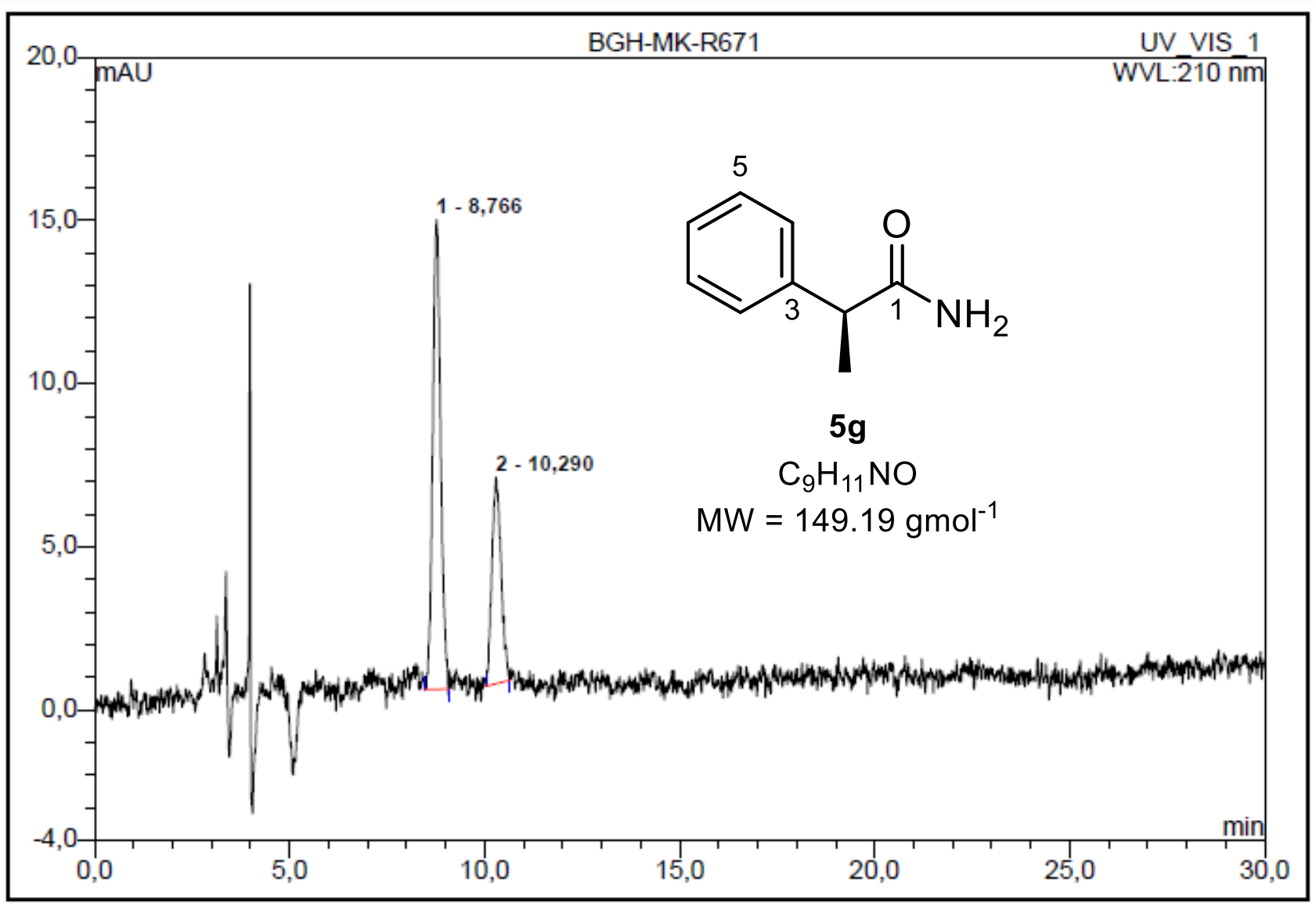

No.	Ret.Time min	Peak Name	Height mAU	Area mAU*min	Rel.Area %	Amount	Type
1	8,77	n.a.	14,395	3,383	66,15	n.a.	BMB*
2	10,29	n.a.	6,327	1,731	33,85	n.a.	BMB*
Total:			20,721	5,113	100,00	0,000	

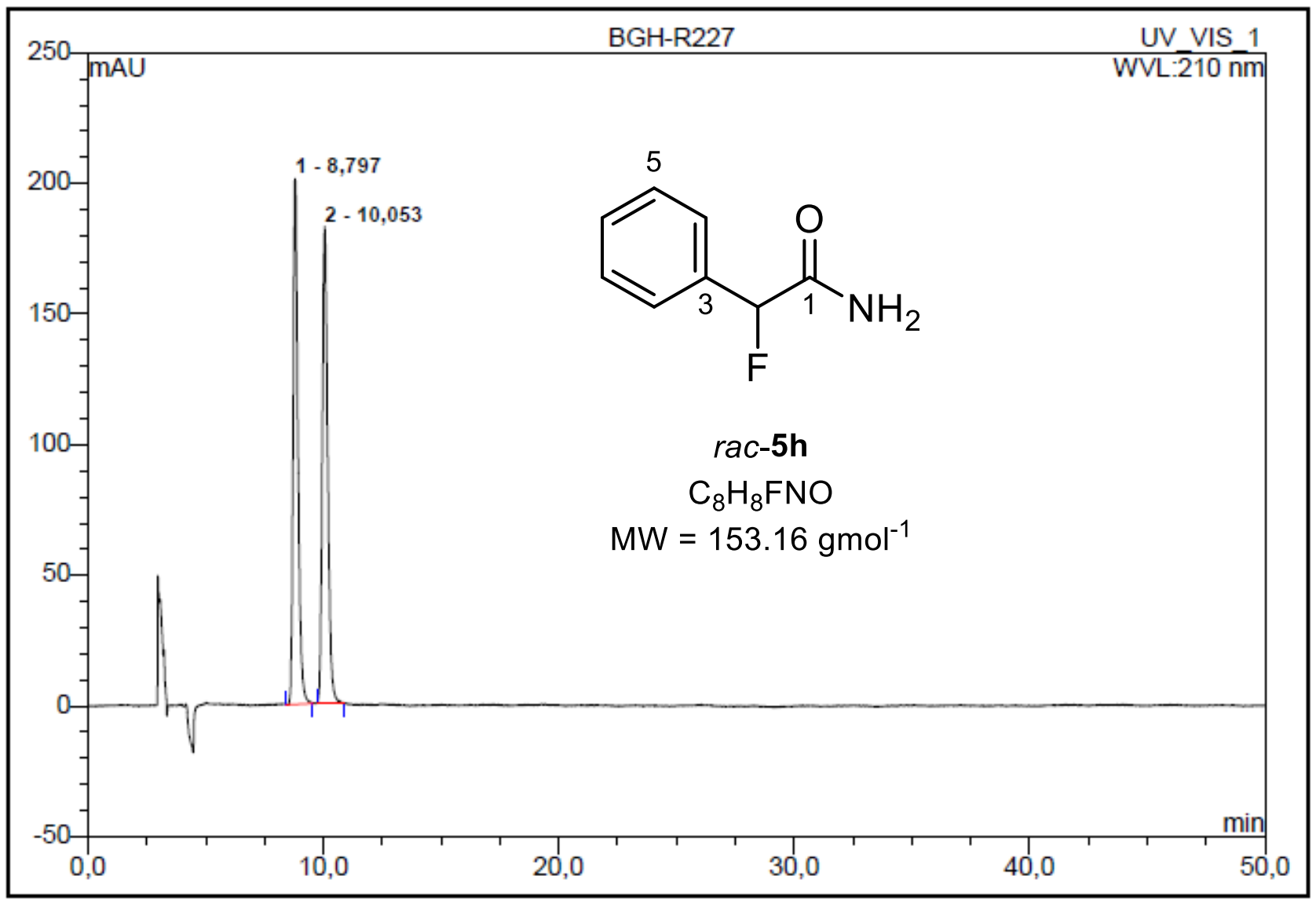

No.	Ret.Time min	Peak Name	Height mAU	Area mAU*min	Rel.Area %	Amount	Type
1	8,80	n.a.	201,049	48,101	50,04	n.a.	BMB*
2	10,05	n.a.	182,252	48,028	49,96	n.a.	BMB*
Total:			383,301	96,129	100,00	0,000	

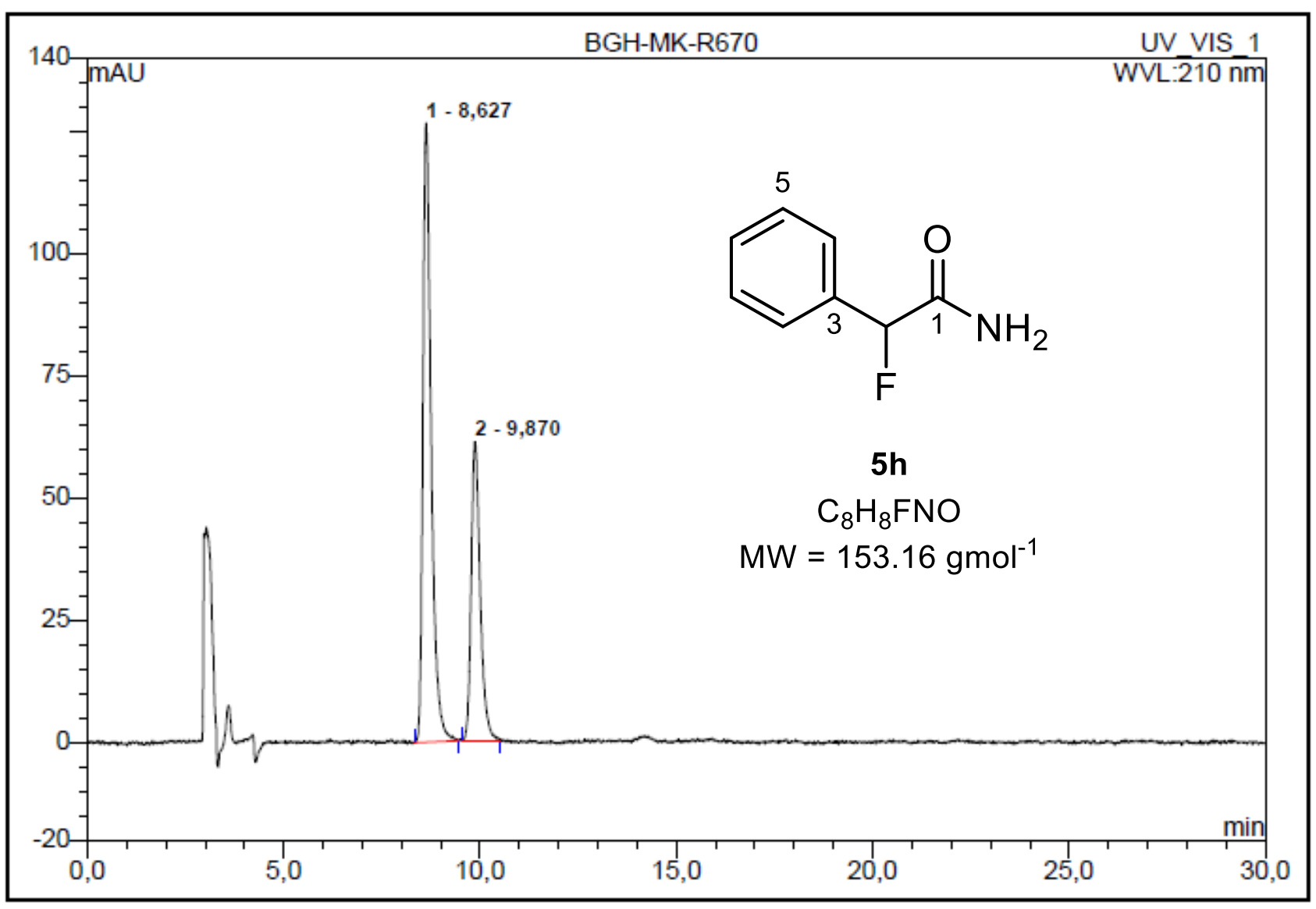

No.	Ret.Time min	Peak Name	Height mAU	Area mAU*min	Rel.Area %	Amount	Type
1	8,63	n.a.	126,480	30,883	65,66	n.a.	BMB*
2	9,87	n.a.	61,087	16,150	34,34	n.a.	BMB*
Total:			187,567	47,034	100,00	0,000	

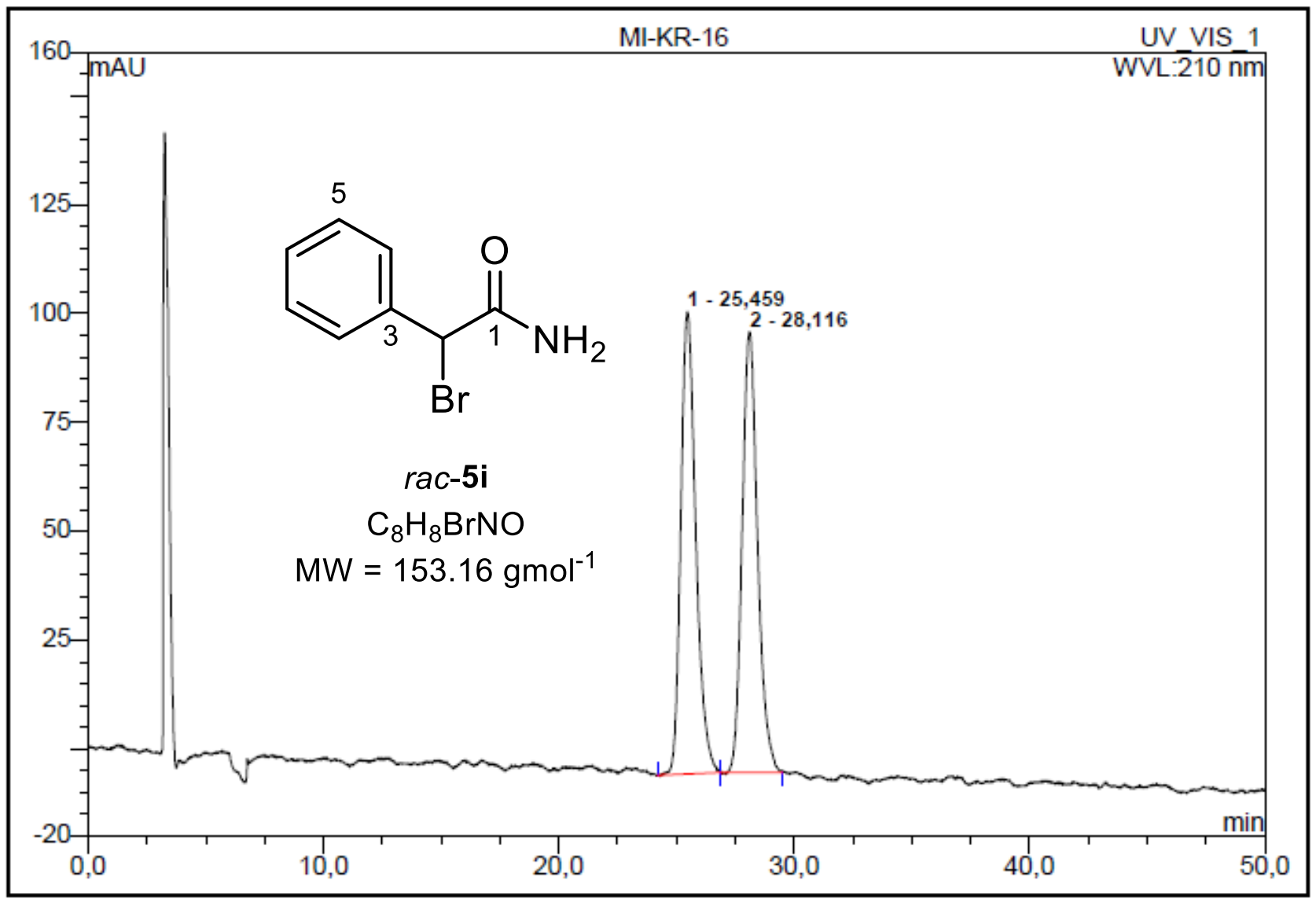

No.	Ret.Time min	Peak Name	Height mAU	Area mAU*min	Rel.Area %	Amount	Type
1	25,46	n.a.	106,136	78,492	50,43	n.a.	BMb*
2	28,12	n.a.	101,155	77,141	49,57	n.a.	bMB*
Total:			207,292	155,633	100,00	0,000	

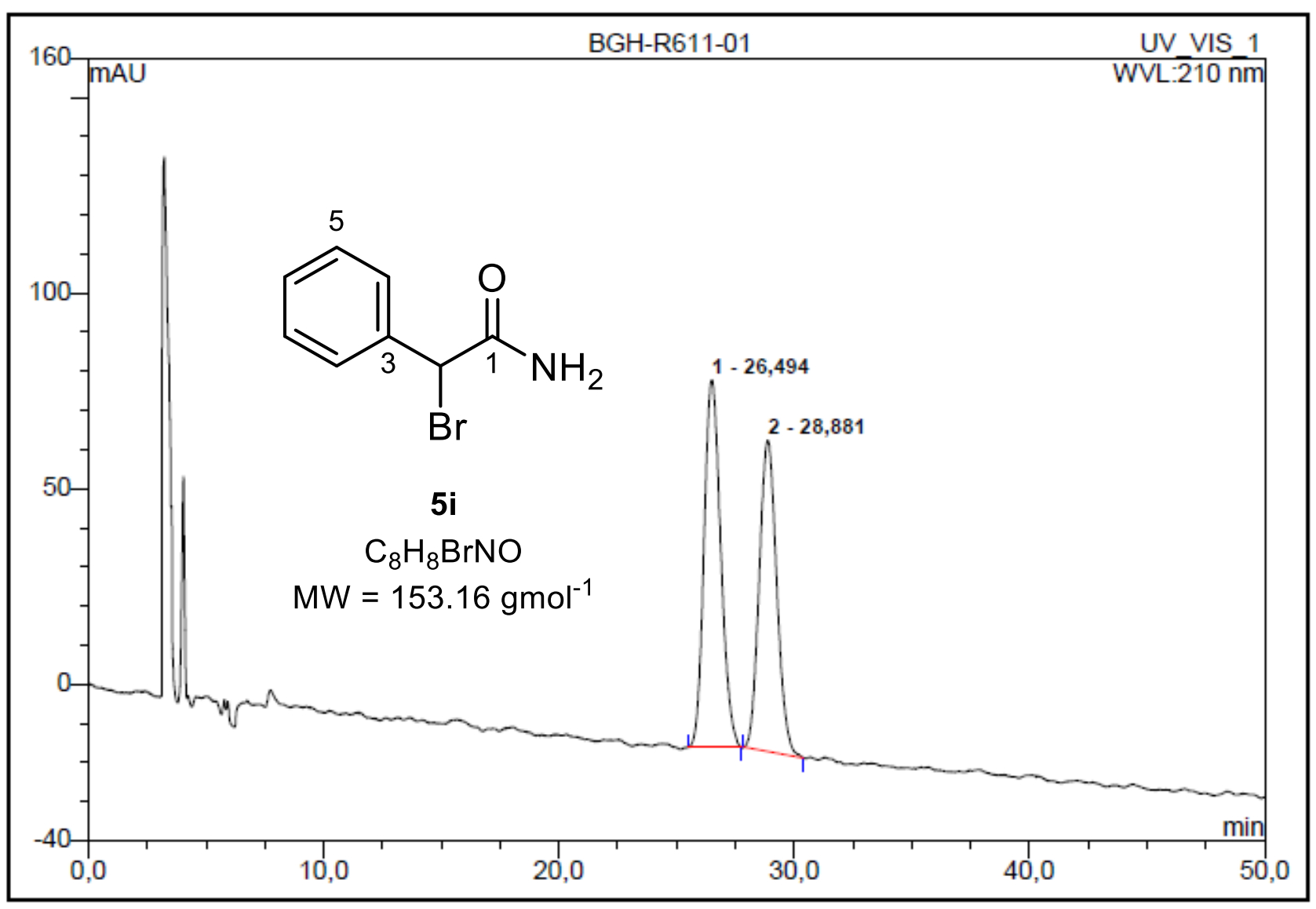

No.	Ret.Time min	Peak Name	Height mAU	Area mAU*min	Rel.Area %	Amount	Type
1	26,49	n.a.	93,833	77,037	52,92	n.a.	BMB
2	28,88	n.a.	79,566	68,539	47,08	n.a.	BMB*
Total:			173,399	145,576	100,00	0,000	

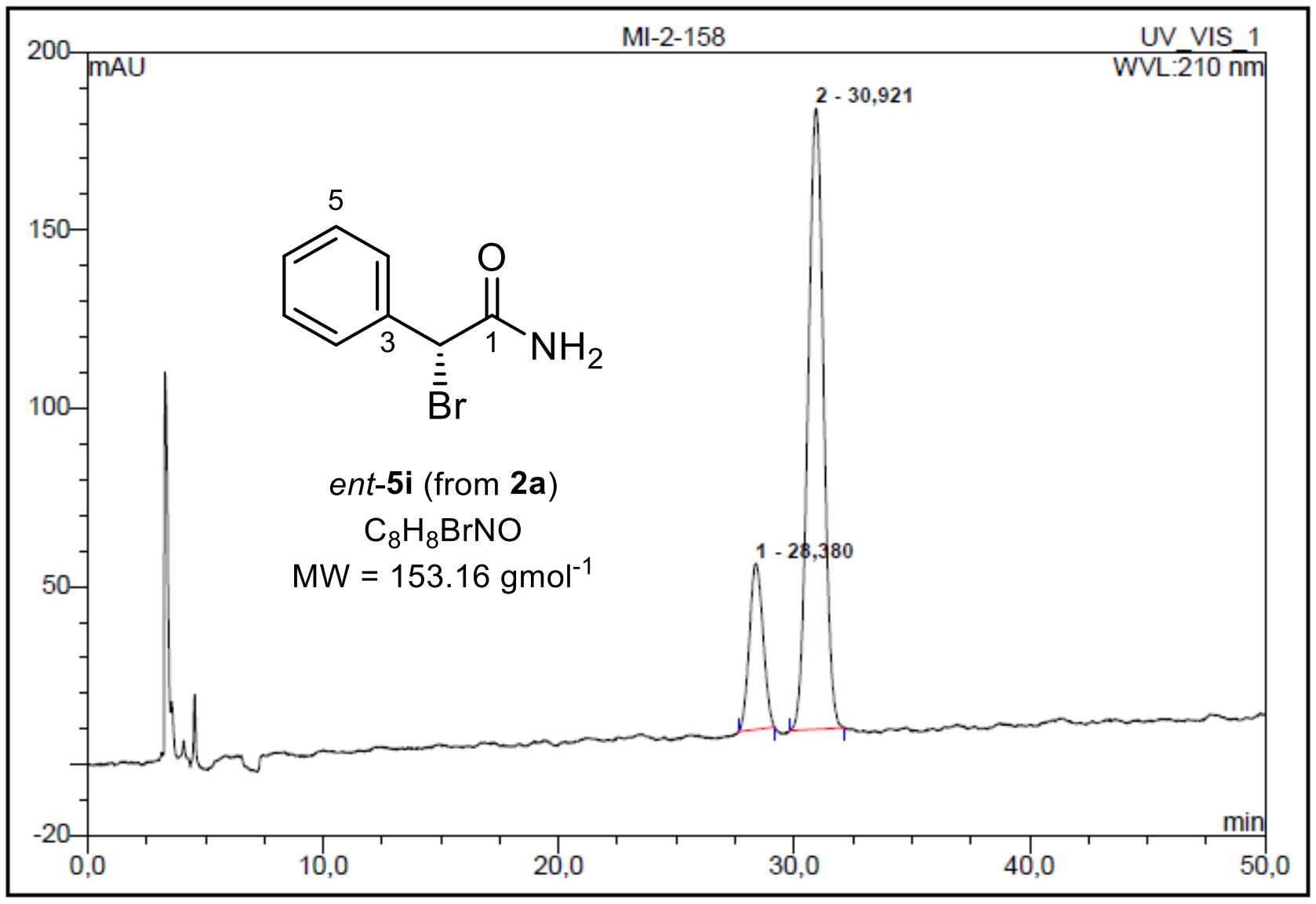

No.	Ret.Time min	Peak Name	Height mAU	Area mAU*min	Rel.Area %	Amount	Type
1	28,38	n.a.	46,545	31,319	19,04	n.a.	BMB*
2	30,92	n.a.	174,292	133,208	80,96	n.a.	BMB*
Total:			220,836	164,527	100,00	0,000	

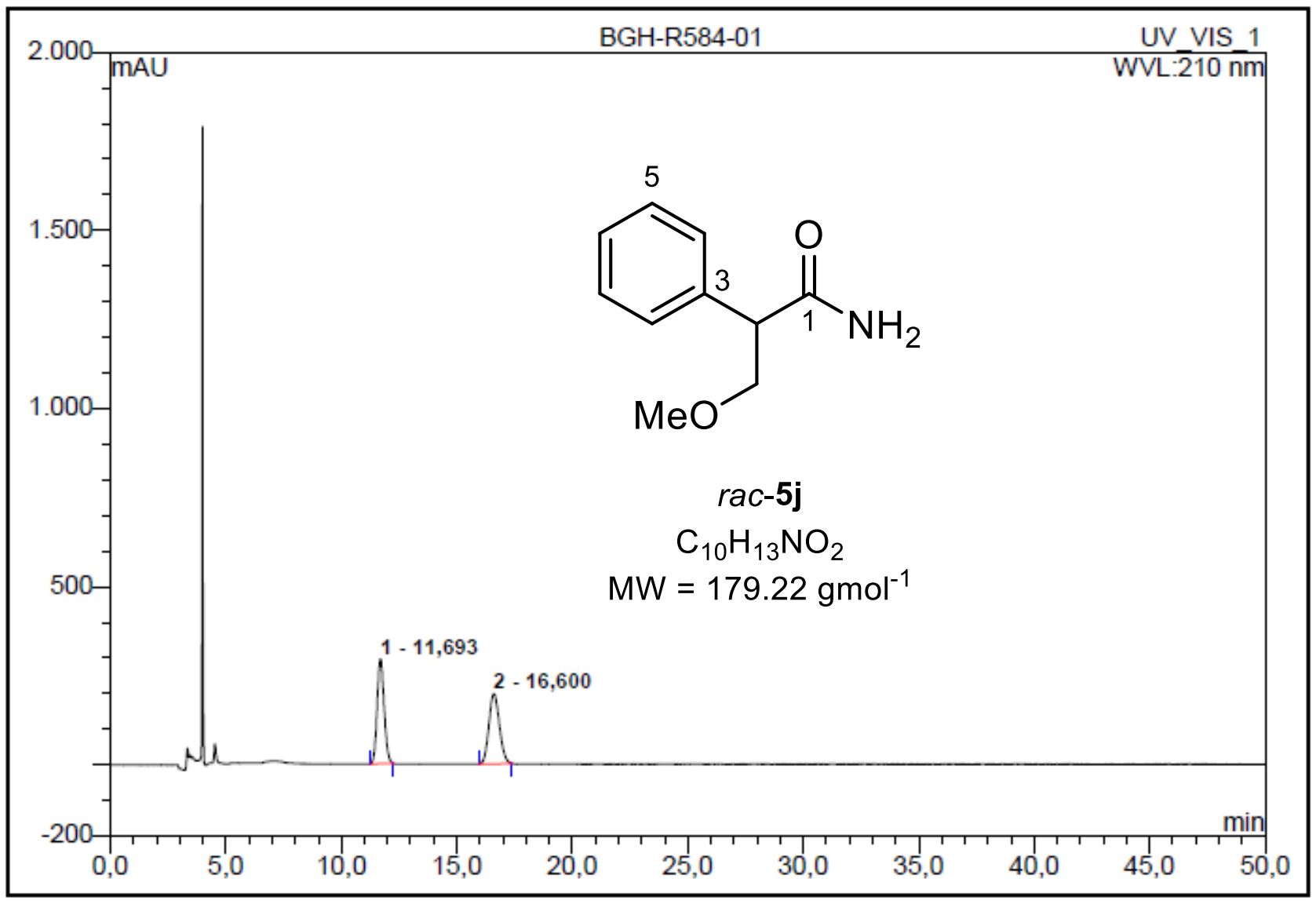

No.	Ret.Time min	Peak Name	Height mAU	Area mAU*min	Rel.Area %	Amount	Type
1	11,69	n.a.	293,226	103,997	49,99	n.a.	BMB
2	16,60	n.a.	196,434	104,028	50,01	n.a.	BMB
Total:			489,660	208,025	100,00	0,000	

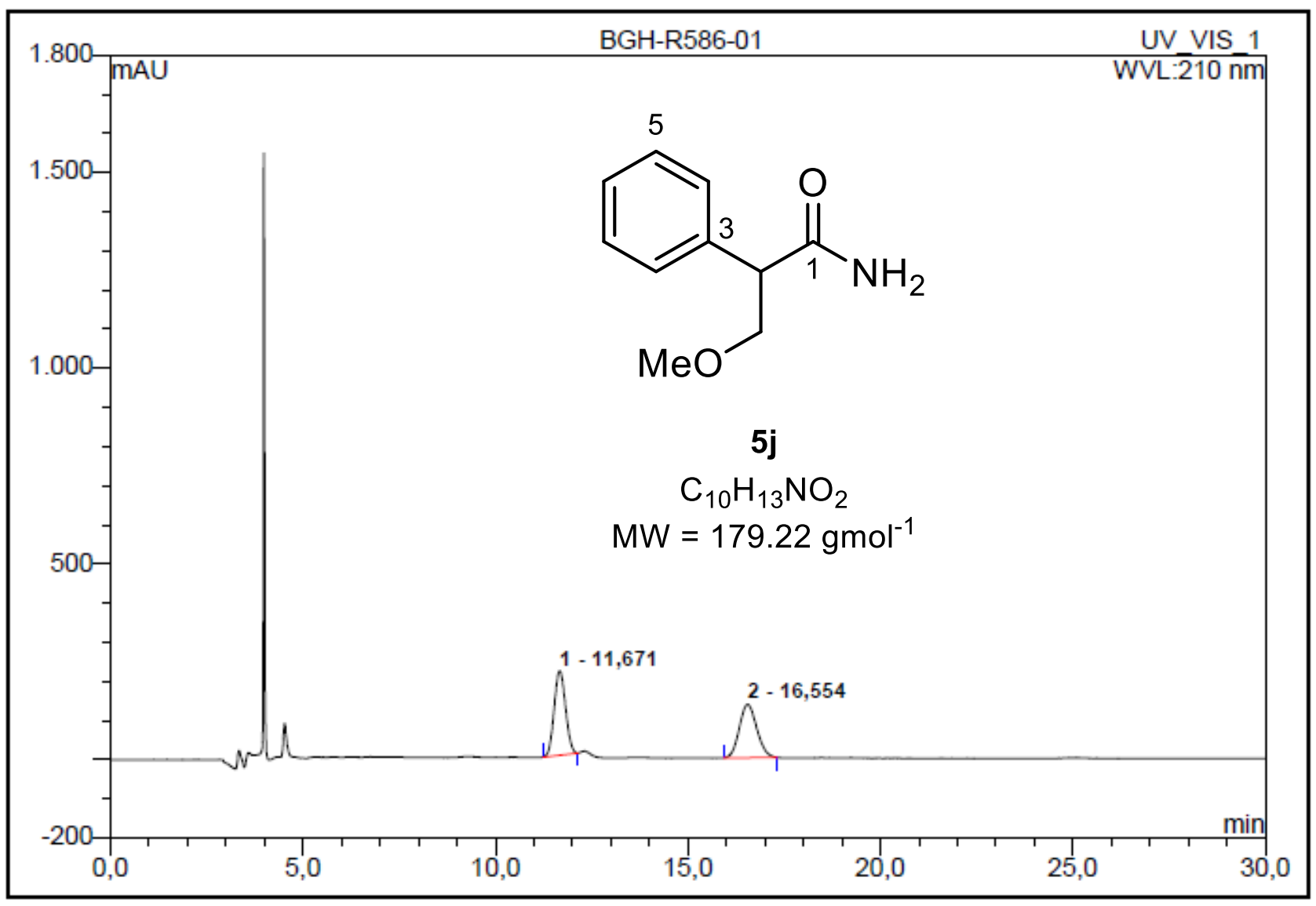

No.	Ret.Time min	Peak Name	Height mAU	Area mAU*min	Rel.Area %	Amount	Type
1	11,67	n.a.	215,460	73,722	50,77	n.a.	BMB
2	16,55	n.a.	136,143	71,488	49,23	n.a.	BMB
Total:			351,603	145,209	100,00	0,000	

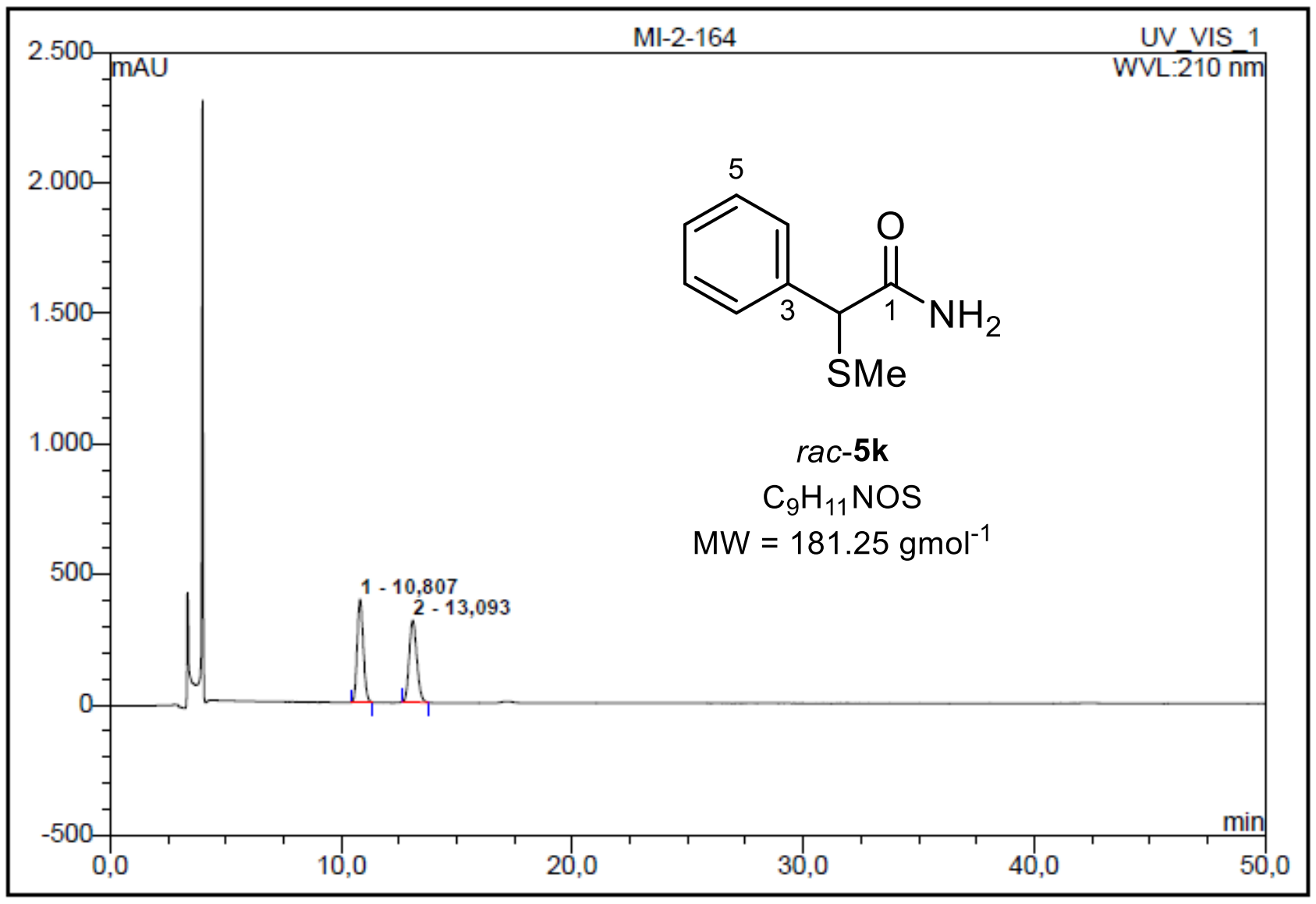

No.	Ret.Time min	Peak Name	Height mAU	Area mAU*min	Rel.Area %	Amount	Type
1	10,81	n.a.	394,170	121,745	50,23	n.a.	BMB
2	13,09	n.a.	312,895	120,618	49,77	n.a.	BMB
Total:			707,065	242,362	100,00	0,000	

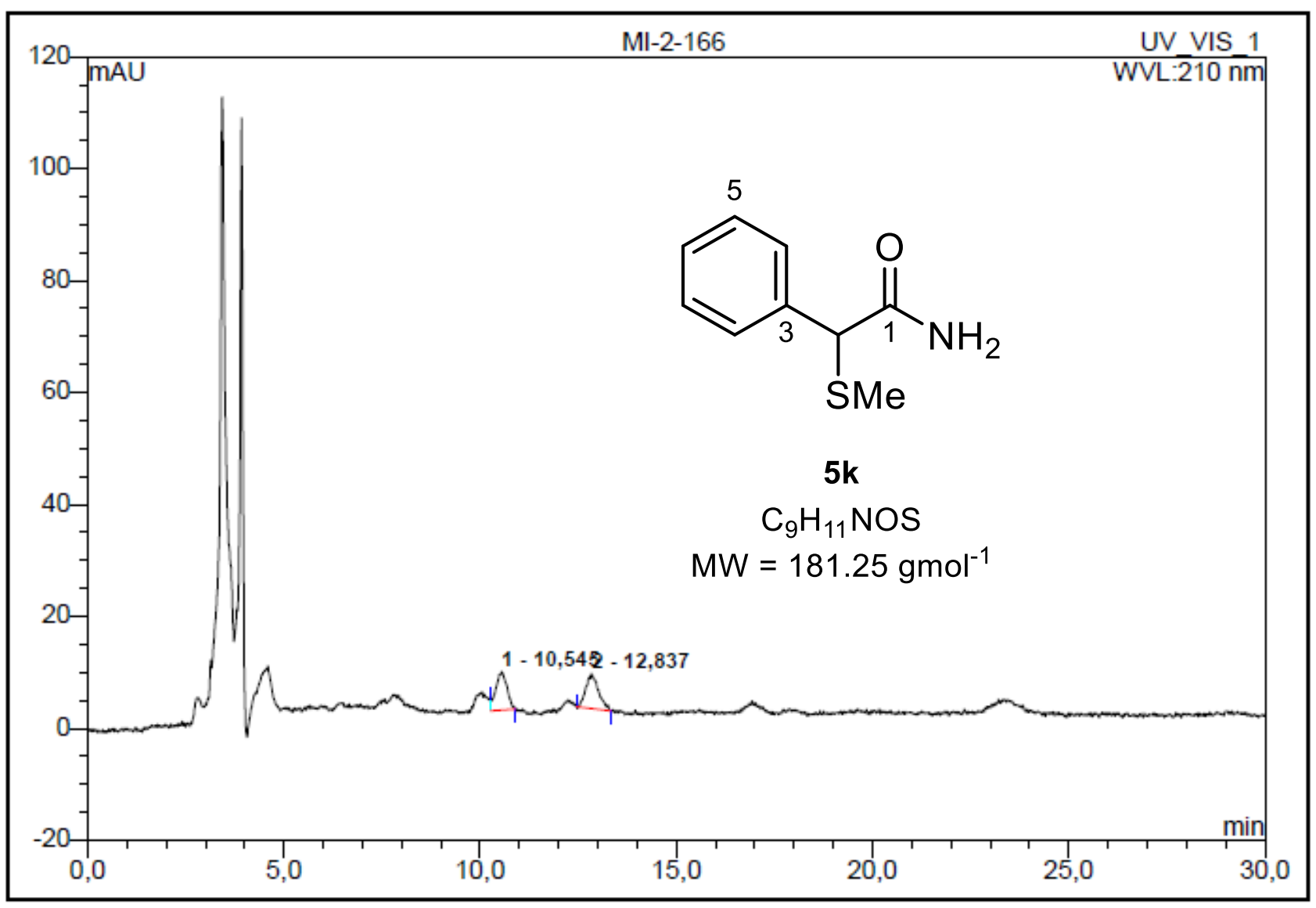

No.	Ret.Time min	Peak Name	Height mAU	Area mAU*min	Rel.Area %	Amount	Type
1	10,55	n.a.	6,855	2,271	49,79	n.a.	MB*
2	12,84	n.a.	6,221	2,290	50,21	n.a.	BMB*
Total:			13,076	4,561	100,00	0,000	

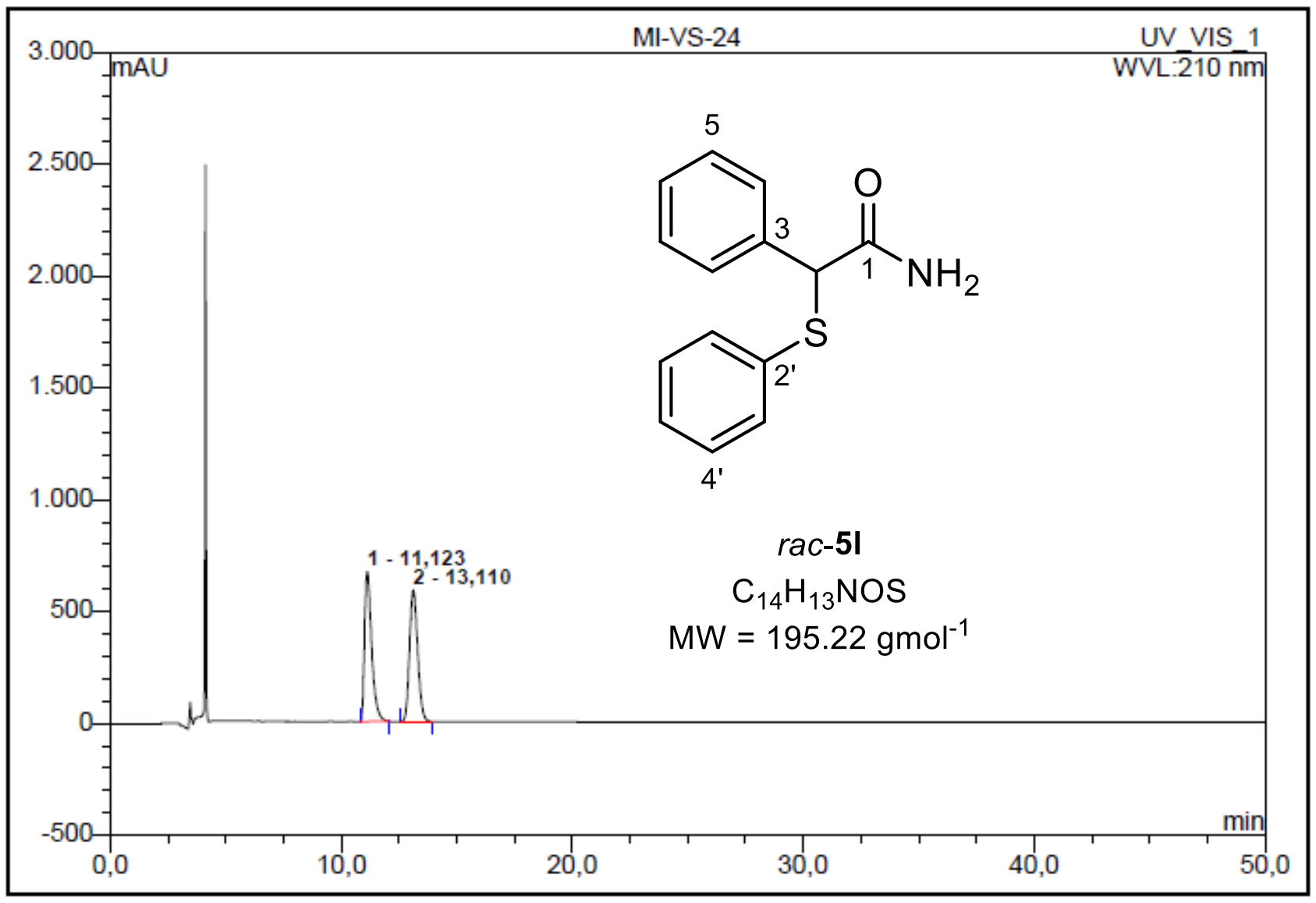

No.	Ret.Time min	Peak Name	Height mAU	Area mAU*min	Rel.Area %	Amount	Type
1	11,12	n.a.	669,866	240,973	49,92	n.a.	BMB
2	13,11	n.a.	587,008	241,770	50,08	n.a.	BMB
Total:			1256,874	482,743	100,00	0,000	

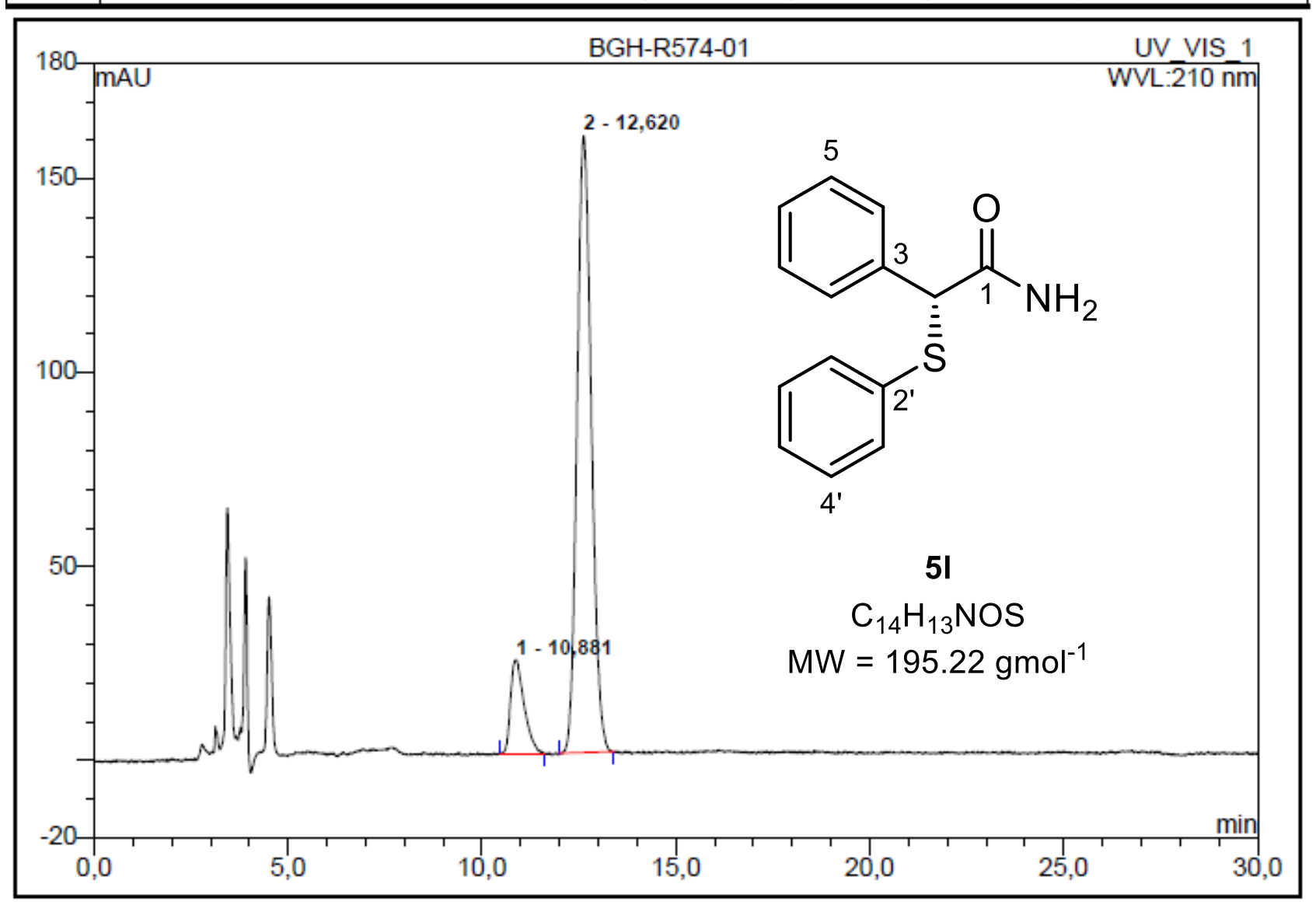

No.	Ret.Time min	Peak Name	Height mAU	Area mAU*min	Rel.Area %	Amount	Type
1	10,88	n.a.	24,098	9,812	12,61	n.a.	BMB*
2	12,62	n.a.	159,152	68,017	87,39	n.a.	BMB*
Total:			183,250	77,829	100,00	0,000	

S18. List of References

1 M. Plaza, J. Großkopf, S. Breitenlechner, C. Bannwarth and T. Bach, *J. Am. Chem. Soc.*, 2021, **143**, 11209-11217.

2 W. Reeve and C. W. Woods, *J. Am. Chem. Soc.*, 1960, **82**, 4062-4066.

3 A. McKenzie, *J. Chem. Soc., Trans.*, 1899, **75**, 753-770.

4 W. Jackson, H. Jacobs, G. Jayatilake, B. Matthews and K. Watson, *Aust. J. Chem.*, 1990, **43**, 2045-2062.

5 B. Ghosh, M. Iglhaut, D. Babushkina, M. Pauls, C. Bannwarth and T. Bach, *Angew. Chem. Int. Ed.*, 2026, **65**, e21436.

6 (a) S. Nakamura, R. Nakagawa, Y. Watanabe and T. Toru, *J. Am. Chem. Soc.*, 2000, **122**, 11340-11347. (b) D. Weinzierl, M. Piringer, P. Zebrowski, L. Stockhammer and M. Waser, *Org. Lett.*, 2023, **25**, 3126-3130.

7 M. Kimura, A. Kuboki and T. Sugai, *Tetrahedron: Asymmetry*, 2002, **13**, 1059-1068.

8 E. V. Bellale, D. S. Bhalerao and K. G. Akamanchi, *J. Org. Chem.*, 2008, **73**, 9473-9475.

9 N. Tka, J. Kraïem and B. B. Hassine, *Synth. Commun.*, 2013, **43**, 735-743.
